# Modeling COVID-19 scenarios for the United States

**DOI:** 10.1038/s41591-020-1132-9

**Published:** 2020-10-23

**Authors:** IHME COVID-19 Forecasting Team, Robert C. Reiner, Ryan M. Barber, James K. Collins, Peng Zheng, Christopher Adolph, James Albright, Catherine M. Antony, Aleksandr Y. Aravkin, Steven D. Bachmeier, Bree Bang-Jensen, Marlena S. Bannick, Sabina Bloom, Austin Carter, Emma Castro, Kate Causey, Suman Chakrabarti, Fiona J. Charlson, Rebecca M. Cogen, Emily Combs, Xiaochen Dai, William James Dangel, Lucas Earl, Samuel B. Ewald, Maha Ezalarab, Alize J. Ferrari, Abraham Flaxman, Joseph Jon Frostad, Nancy Fullman, Emmanuela Gakidou, John Gallagher, Scott D. Glenn, Erik A. Goosmann, Jiawei He, Nathaniel J. Henry, Erin N. Hulland, Benjamin Hurst, Casey Johanns, Parkes J. Kendrick, Apurva Khemani, Samantha Leigh Larson, Alice Lazzar-Atwood, Kate E. LeGrand, Haley Lescinsky, Akiaja Lindstrom, Emily Linebarger, Rafael Lozano, Rui Ma, Johan Månsson, Beatrice Magistro, Ana M. Mantilla Herrera, Laurie B. Marczak, Molly K. Miller-Petrie, Ali H. Mokdad, Julia Deryn Morgan, Paulami Naik, Christopher M. Odell, James K. O’Halloran, Aaron E. Osgood-Zimmerman, Samuel M. Ostroff, Maja Pasovic, Louise Penberthy, Geoffrey Phipps, David M. Pigott, Ian Pollock, Rebecca E. Ramshaw, Sofia Boston Redford, Grace Reinke, Sam Rolfe, Damian Francesco Santomauro, John R. Shackleton, David H. Shaw, Brittney S. Sheena, Aleksei Sholokhov, Reed J. D. Sorensen, Gianna Sparks, Emma Elizabeth Spurlock, Michelle L. Subart, Ruri Syailendrawati, Anna E. Torre, Christopher E. Troeger, Theo Vos, Alexandrea Watson, Stefanie Watson, Kirsten E. Wiens, Lauren Woyczynski, Liming Xu, Jize Zhang, Simon I. Hay, Stephen S. Lim, Christopher J. L. Murray

**Affiliations:** 1 Institute for Health Metrics and Evaluation, University of Washington, Seattle, WA USA; 2 Department of Health Metrics Sciences, School of Medicine, University of Washington, Seattle, WA USA; 3 Department of Political Science, University of Washington, Seattle, WA USA; 4 Center for Statistics and the Social Sciences, University of Washington, Seattle, WA USA; 5 Department of Applied Mathematics, University of Washington, Seattle, WA USA; 6 Queensland Centre for Mental Health Research, The University of Queensland, Brisbane, Queensland Australia; 7 School of Public Health, The University of Queensland, Brisbane, Queensland Australia; 8 Queensland Centre for Mental Health Research, Brisbane, Queensland Australia; 9 Big Data Institute, University of Oxford, Oxford, UK; 10 Department of Global Health, University of Washington, Seattle, WA USA; 11 QIMR Berghofer Medical Research Institute, Brisbane, Queensland Australia; 12 Henry M Jackson School of International Studies, University of Washington, Seattle, WA USA; 13 Policy and Epidemiology Group, Queensland Centre for Mental Health Research, Brisbane, Queensland Australia

**Keywords:** Infectious diseases, Health policy

## Abstract

We use COVID-19 case and mortality data from 1 February 2020 to 21 September 2020 and a deterministic SEIR (susceptible, exposed, infectious and recovered) compartmental framework to model possible trajectories of severe acute respiratory syndrome coronavirus 2 (SARS-CoV-2) infections and the effects of non-pharmaceutical interventions in the United States at the state level from 22 September 2020 through 28 February 2021. Using this SEIR model, and projections of critical driving covariates (pneumonia seasonality, mobility, testing rates and mask use per capita), we assessed scenarios of social distancing mandates and levels of mask use. Projections of current non-pharmaceutical intervention strategies by state—with social distancing mandates reinstated when a threshold of 8 deaths per million population is exceeded (reference scenario)—suggest that, cumulatively, 511,373 (469,578–578,347) lives could be lost to COVID-19 across the United States by 28 February 2021. We find that achieving universal mask use (95% mask use in public) could be sufficient to ameliorate the worst effects of epidemic resurgences in many states. Universal mask use could save an additional 129,574 (85,284–170,867) lives from September 22, 2020 through the end of February 2021, or an additional 95,814 (60,731–133,077) lives assuming a lesser adoption of mask wearing (85%), when compared to the reference scenario.

## Abstract (web summary)

A modeling study using case and mortality data from the first 8 months of the COVID-19 pandemic in the United States explores five potential future scenarios of social distancing mandates and mask use at the state level, with projections of the course of the epidemic through winter 2021.

## Main

The zoonotic origin of the novel severe acute respiratory syndrome coronavirus 2 (SARS-CoV-2)^1^ first reported in Wuhan, China^2^, and the global spread of the coronavirus disease 2019 (COVID-19; https://covid19.who.int/)^3^ promises to be a defining global health event of the twenty-first century^4^. This pandemic has already resulted in extreme societal, economic and political disruption across the world and in the United States (https://www.economist.com/united-states/2020/03/14/tracking-the-economic-impact-of-covid-19-in-real-time/)^5,6^. The establishment of SARS-CoV-2 and its rapid spread in the United States has been dramatic (https://www.thinkglobalhealth.org/article/updated-timeline-coronavirus/). Since the first case in the United States was identified on 20 January 2020 (ref. ^7^; first death on 6 February 2020: https://www.sccgov.org/sites/covid19/Pages/press-release-04-21-20-early.aspx), SARS-CoV-2 has spread to every state and has resulted in more than 28.2 million cases and 199,213 deaths as of 21 September 2020 (https://coronavirus.jhu.edu/map.html)^7,8^.

**Fig. 1:**
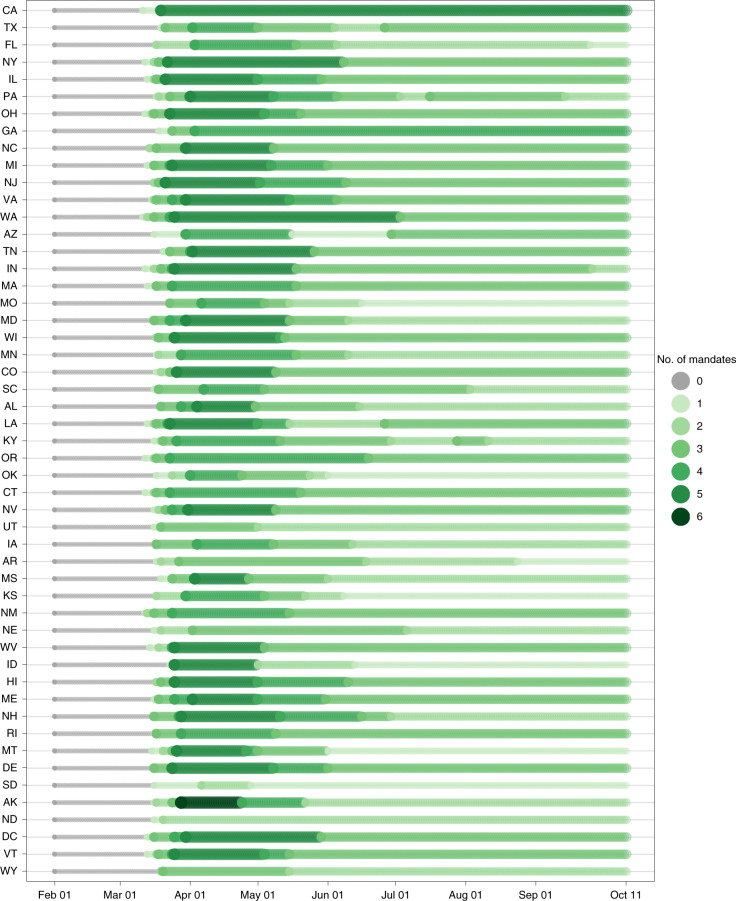
Number of social distancing mandates by US state from 1 February 2020 to 22 September 2020. States are ordered by decreasing population size on the *y* axis. Source data

There remains no approved vaccine for the prevention of SARS-CoV-2 infection, and few pharmaceutical options for the treatment of COVID-19 are available^9–11^. The most optimistic scientists do not predict the availability of new vaccines or therapeutics before 2021 (refs. ^12–15^). Non-pharmaceutical interventions (NPIs) are, therefore, the only available policy levers to reduce transmission^16^. Several NPIs have been put in place across the United States in response to the epidemic (Fig. 1), including the dampening of transmission through the wearing of face masks and social distancing mandates (SDMs) aimed at reducing contacts through school closures, restrictions of gatherings, stay-at-home orders and the partial or full closure of nonessential businesses. Increased testing and isolation of infected individuals and their contacts will also have had an impact^17^. These NPIs are credited with a reduction in viral transmission^18,19^, along with a host of other environmental, behavioral and social determinants postulated to affect the course of the epidemic at the state level.

In the United States, decisions to implement SDM or require mask use are generally made at the state level by government officials. These executives need to balance net losses from the societal turmoil, economic damage and indirect effects on health caused by NPIs with the direct benefits to human health of controlling the epidemic. Disease control has often been operationally defined in this pandemic context as the restriction of infections to below a specified level at which health services are not overwhelmed by demand and the loss of human health and life is consequently minimized^20^.

In the first months of the SARS-CoV-2 outbreak in the United States, states enacted restrictive SDMs intended to reduce transmission (by limiting human-to-human contact)^5^, while there was conflicting advice on the use of masks (https://www.npr.org/sections/goatsandsoda/2020/04/10/829890635/why-there-so-manydifferent-guidelines-for-face-masks-for-the-public/). At that early stage, relatively simple statistical models of future risk were sufficient to capture the general patterns of transmission^21^. As different behavioral responses to SDMs emerged and, more importantly, as some states began to relax SDMs (Fig. 1), a modeling approach that directly quantified transmission and could be used to explore these developing scenarios was necessary. As states varied in their actions to remove and reinstate SDMs (Fig. 1) or began to issue mandatory mask-use orders (https://www.cnn.com/2020/06/19/us/states-face-mask-coronavirus-trnd/index.html) amid resurgences of COVID-19 (https://www.nytimes.com/2020/07/01/world/coronavirus-updates.html), a clear need for evidence-based assessments of the possible effect of the NPI options available to decision-makers became apparent.

There is now growing evidence that face masks can considerably reduce the transmission of respiratory viruses like SARS-CoV-2, thereby limiting the spread of COVID-19 (refs. ^22–24^). We updated a recently published review^24^ to generate a new meta-analysis (Supplementary Information) of peer-reviewed studies and preprints to assess the effectiveness of masks at preventing respiratory viral infections in humans^25^. This analysis indicated a reduction in infection (from all respiratory viruses) for mask wearers by 40% (relative risk = 0.60, 95% uncertainty interval (UI) = 0.46–0.80)) relative to controls^25^. This is suggestive of a considerable population health benefit to mask use with great potential for uptake in the United States, where the national average for self-reported mask wearing was 49% as of 21 September 2020 (https://covid19.healthdata.org/; Supplementary Information).

Here we provide a state-level descriptive epidemiological analysis of the introduction of SARS-CoV-2 infection across the United States, from the first recorded case through to 21 September 2020. We use these observations to learn about epidemic progression and thereby model the first wave of transmission using a deterministic SEIR compartmental framework^26,27^. This observed, process-based understanding of how NPIs affect epidemiological processes is then used to make inferences about the future trajectory of COVID-19 and how different combinations of existing NPIs might affect this course. Five SEIR-driven scenarios, along with covariates that inform them, were then projected through to 28 February 2021 (Methods). We use these scenarios as a sequence of experiments to describe a range of model outputs, including *R*_*effective*_ (the change over time in the average number of secondary cases per infectious case in a population where not everyone is susceptible^26–28^), infections, deaths and hospital demand outcomes, which might be expected from plausible boundaries of the policy options available the fall and winter of 2020 (see Methods and Supplementary Information for an extended rationale on scenario construction).

We established three boundary scenarios. First, we forecast the expected outcomes if states continue to remove SDMs at the current pace of ‘mandate easing’, with resulting increases in population mobility and number of person-to-person contacts. This is an alternative scenario to the more probable situation where states are expected to respond to an impending health crisis by reinstating some SDMs. In the second, ‘plausible reference’ scenario, we model the future progress of the pandemic assuming that states would once again shut down social interaction and some economic activity at a threshold for the daily death rate of 8 deaths per million population—the 90th percentile of the observed distribution of when states previously implemented SDMs (Fig. 1 and Supplementary Information). This scenario assumes reinstatement of SDMs for 6 weeks. In addition, newly available data on mask efficacy enabled the exploration of a third, ‘universal mask-use’ scenario to investigate the potential population-level benefits of increased mask use in addition to the same threshold-driven reinstatement of SDMs. In this best-case scenario model, ‘universal’ was defined as 95% of people wearing masks in public, based on the highest observed coverage of mask use globally (in Singapore) during the COVID-19 pandemic to date (Supplementary Information). Two derivative scenarios were also included to assist understanding, nuance and policy resolution around the three boundary scenarios. The first scenario, termed ‘plausible reference + 85% mask use’, modeled less than universal mask use in public (85%) in the presence of reinstatement of SDMs. The second was a scenario of universal mask use (95%) in the absence of any NPIs (termed ‘mandate easing + universal mask use’). Details and results for these additional scenarios are in the Supplementary Information. In addition, sensitivity analyses and detailed diagnostics are provided to help users calibrate the effects of the covariates used in the models on the scenarios discussed (Supplementary Information).

## Results

### Observed COVID-19 patterns

**Fig. 2:**
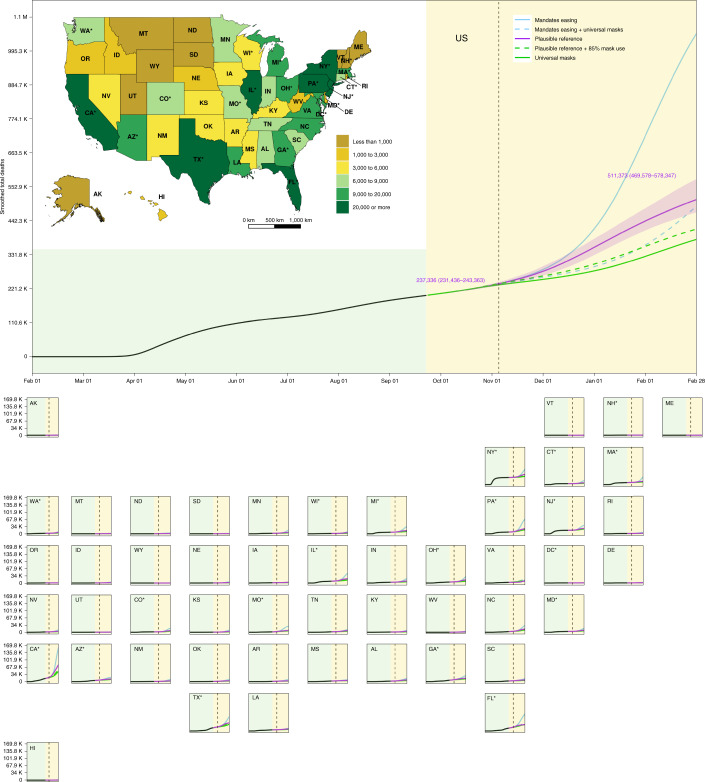
Cumulative deaths from 1 February 2020 to 28 February 2021. The inset map displays the cumulative deaths under the plausible reference scenario on 28 February 2021. A light-yellow background separates the observed and predicted part of the time series, before and after 22 September 2020. The dashed vertical line identifies 3 November 2020. Solid lines represent boundary scenarios and dashed lines represent derivative scenarios. Numbers are the means and UIs for the plausible reference scenario on the highlighted dates. An asterisk indicates states with population centers exceeding 2 million persons. UIs are shown for only the plausible reference scenario. Source data

**Fig. 3:**
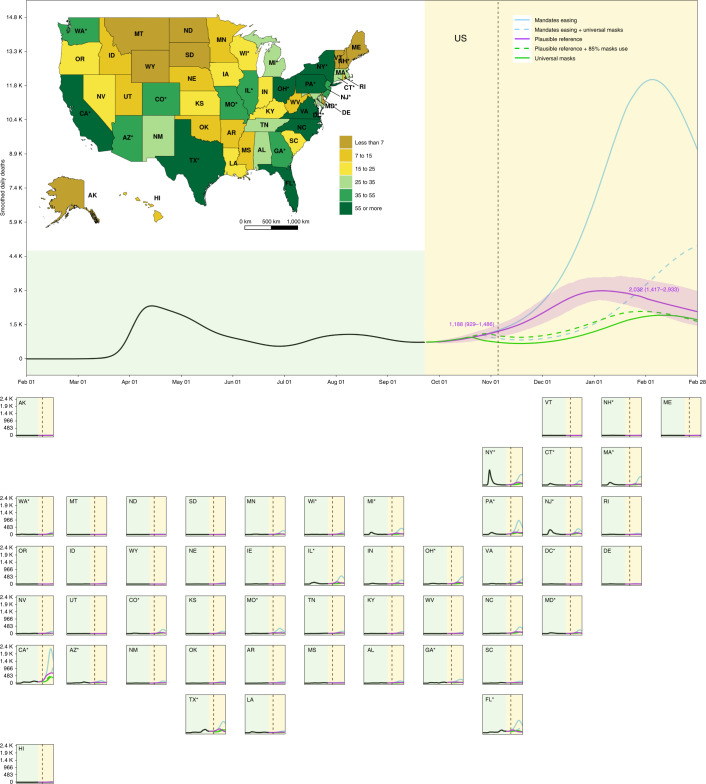
Daily deaths from 1 February 2020 to 28 February 2021. The inset map displays the daily deaths under the plausible reference scenario on 28 February 2021. A light-yellow background separates the observed and predicted part of the time series, before and after 22 September 2020. The dashed vertical line identifies 3 November 2020. Solid lines represent boundary scenarios and dashed lines represent derivative scenarios. Numbers are the means and UIs for the plausible reference scenario on the highlighted dates. An asterisk indicates states with population centers exceeding 2 million persons. UIs are shown for only the plausible reference scenario. Source data

**Fig. 4:**
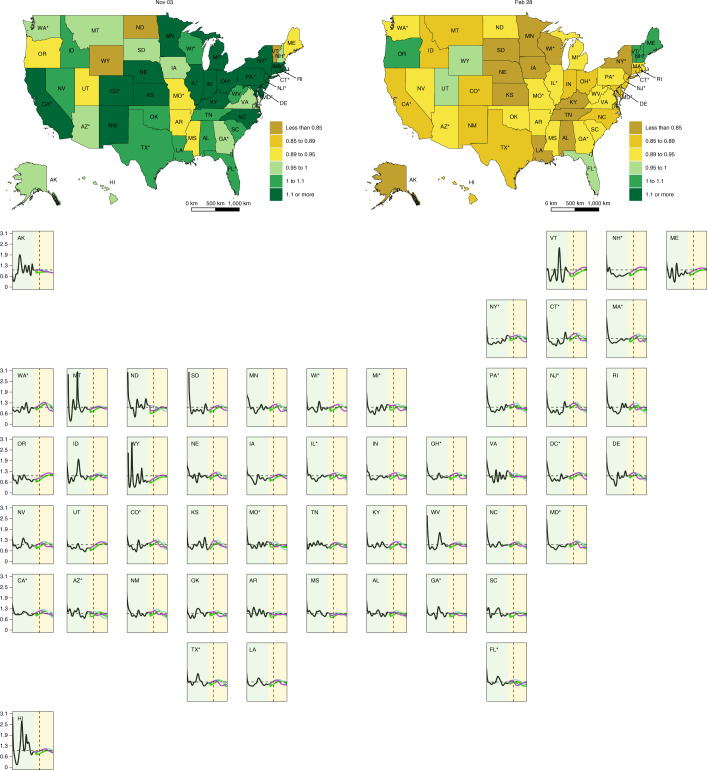
Time series for values of *R*_*effective*_ by US state. Inset maps display the value of *R*_*effective*_ on 3 November 2020 and 28 February 2021; time series of *R*_*effective*_ are presented for each state as separate panels. A light-yellow background separates the observed and predicted part of the time series, before and after 22 September 2020. The dashed vertical line identifies 3 November 2020. An asterisk indicates states with population centers exceeding 2 million persons. Source data

**Extended Data Fig. 1:**
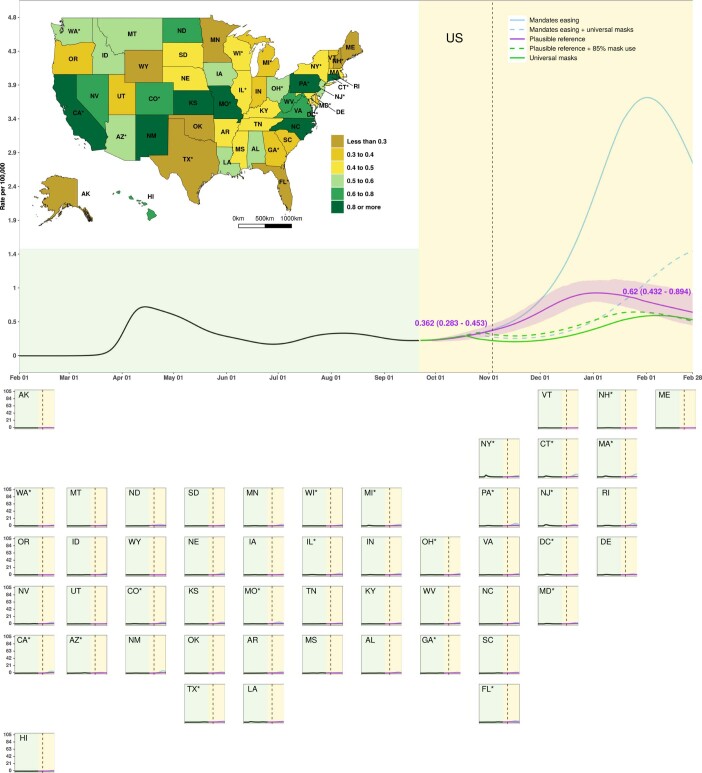
Estimated daily COVID-19 death rate (per 100,000 population) by state for all five scenarios. The inset map displays the estimated daily deaths from COVID-19 death per 100,000 population by state on 28 February 2021. The light yellow background separates the observed and predicted part of the time series, before and after 21 September 2020. The dashed vertical line identifies 03 November 2020. Numbers are the means and uncertainty interval (UI) for the plausible reference scenario on dates highlighted. Source data

**Extended Data Fig. 2:**
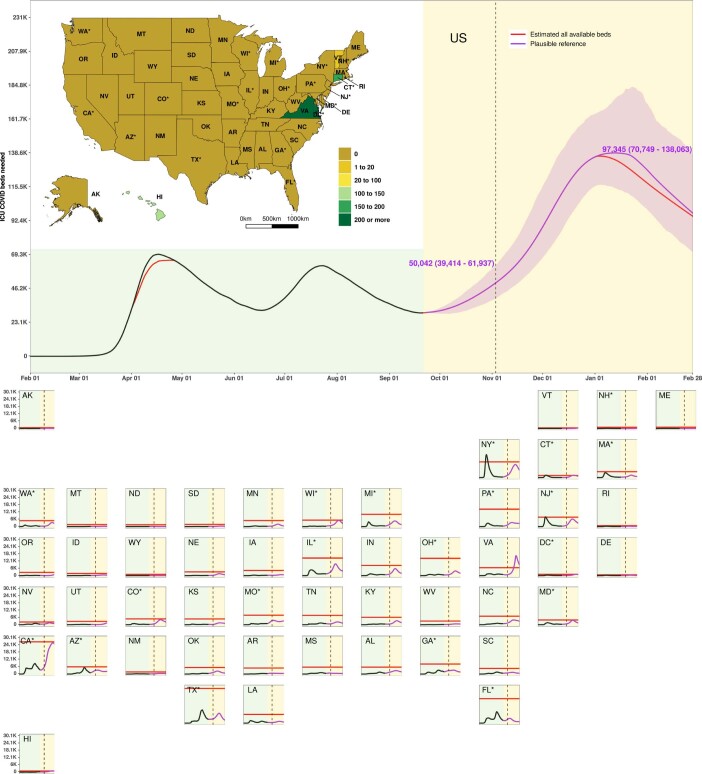
Estimated total hospital beds needed for COVID-19 patients by state from 01 February 2020 to 28 February, 2021, under the plausible reference scenario. The inset map displays the estimated peak number of all COVID-19 beds above capacity by state between 22 September 2020 and 28 February 2021. The light yellow background separates the observed and predicted part of the time series, before and after 21 September 2020. The dashed vertical line identifies 03 November 2020. Numbers are the means and uncertainty interval (UI) for the plausible reference scenario on dates highlighted. Source data

**Extended Data Fig. 3:**
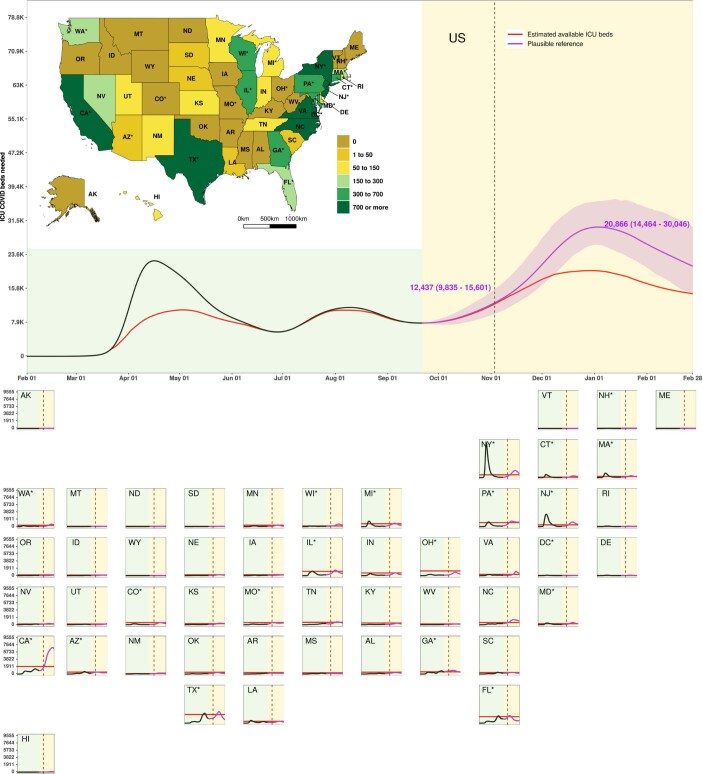
Estimated total ICU beds needed for COVID-19 patients by state from 01 February 2020 to 28 February 2021, under the plausible reference scenario. The inset map displays the estimated peak number of all ICU COVID-19 beds above capacity by state between 22 September 2020 and 28 February 2021. The light yellow background separates the observed and predicted part of the time series, before and after 21 September 2020. The dashed vertical line identifies 03 November 2020. Numbers are the means and uncertainty interval (UI) for the plausible reference scenario on dates highlighted. Source data

The COVID-19 epidemic has progressed unevenly across states. Since the first death was recorded in the United States in early February 2020, cumulative through 21 September 2020, 199,213 deaths from COVID-19 have been reported in the United States (Fig. 2); a sixth of those (16.6%) occurred in New York alone. Washington and California issued the first sets of state-level mandates on 11 March 2020, prohibiting gatherings of 250 people or more in certain counties, and by 23 March 2020, all 50 states initiated some combination of SDMs (Fig. 1). The highest levels of daily deaths at the state level between February and September of 2020 occurred in New York, New Jersey and Texas at 998, 311 and 220 deaths per day, respectively (Fig. 3 and Extended Data Fig. 1). On 21 September 2020, the highest level of daily deaths was in Florida at 101 deaths per day. A critical policy need at this stage of the modeling was the forecasting of hospital resource demands in the US states with the worst effective transmission rates (Virginia, New York and Missouri; Fig. 4). The highest peak demand was observed as 8,380 hospital intensive care unit (ICU) beds in New York (estimated initial hospital ICU bed availability of 718) on April 10 and 2,786 hospital ICU beds in New Jersey (estimated initial hospital ICU bed availability of 466) on April 21; demand for hospital ICU beds had receded to within initial capacity levels across the United States by 21 September 2020 (Extended Data Fig. 3). Hospital resource demands (all bed capacity) had been exceeded in the period before 21 September 2020 in three states (New York, New Jersey and Connecticut; Extended Data Figs. 2 and 3).

### Predicted COVID-19 patterns

**Extended Data Fig. 4:**
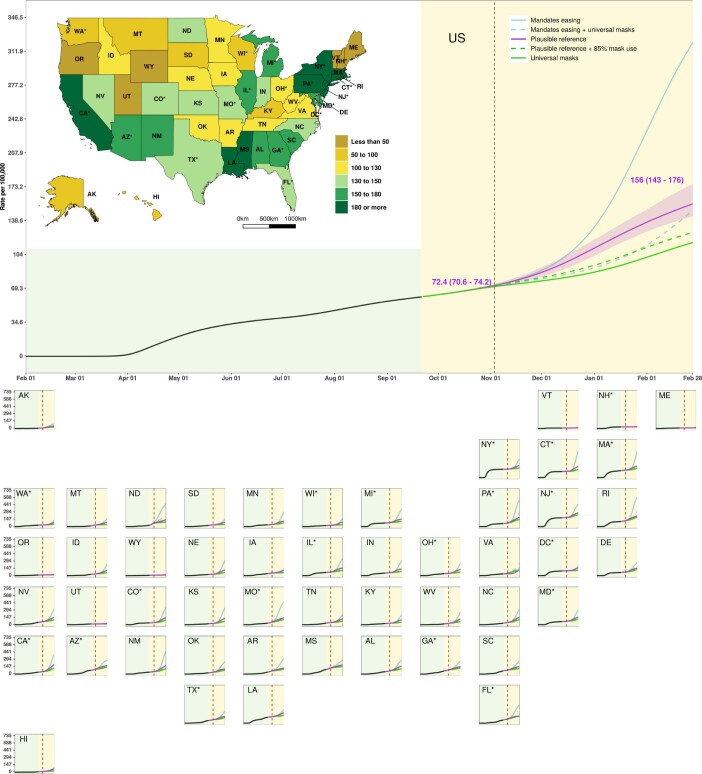
Estimated cumulative deaths from COVID-19 per 100,000 population from 01 February 2020 to 28 February 2021, by state, for all five scenarios. The inset map displays the estimated cumulative deaths per 100,000 population under the plausible reference scenario on 28 February 2021. The light yellow background separates the observed and predicted part of the time series, before and after 21 September 2020. The dashed vertical line identifies 03 November 2020. Numbers are the means and uncertainty interval (UI) for the plausible reference scenario on dates highlighted. The UIs are shown only for the plausible reference scenario. Source data

**Extended Data Fig. 5:**
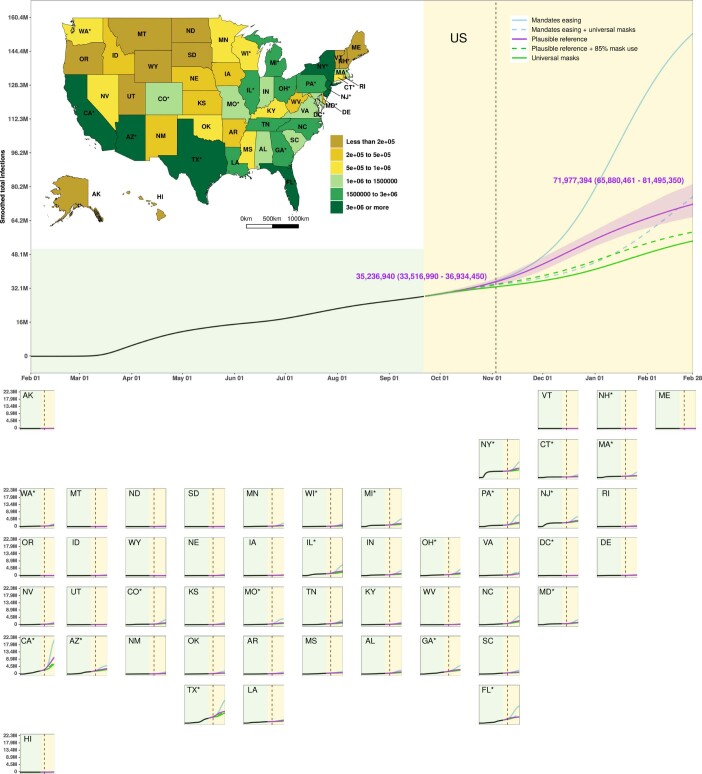
Estimated cumulative infections from SARS-CoV-2 from 01 February 2020 to 28 February 2021, by state, for all five scenarios. The inset map displays the estimated cumulative infections under the plausible reference scenario on 28 February 2021. The light yellow background separates the observed and predicted part of the time series, before and after 21 September 2020. The dashed vertical line identifies 03 November 2020. Numbers are the means and uncertainty interval (UI) for the plausible reference scenario on dates highlighted. The UIs are shown only for the plausible reference scenario. Source data

**Extended Data Fig. 6:**
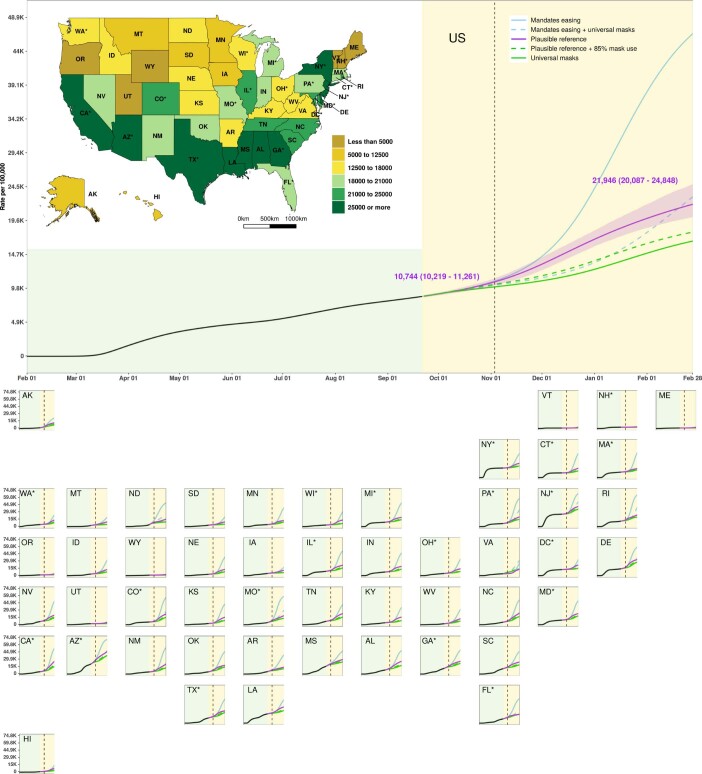
Estimated cumulative SARS-CoV-2 infection rate (per 100,000 population) by state, for all five scenarios. The inset map displays the estimated cumulative infections from COVID-19 per 100,000 population by state on 28 February 2021. The light yellow background separates the observed and predicted part of the time series, before and after 21 September 2020. The dashed vertical line identifies 03 November 2020. Numbers are the means and uncertainty interval (UI) for the plausible reference scenario on dates highlighted. The UIs are shown only for the plausible reference scenario. Source data

**Extended Data Fig. 7:**
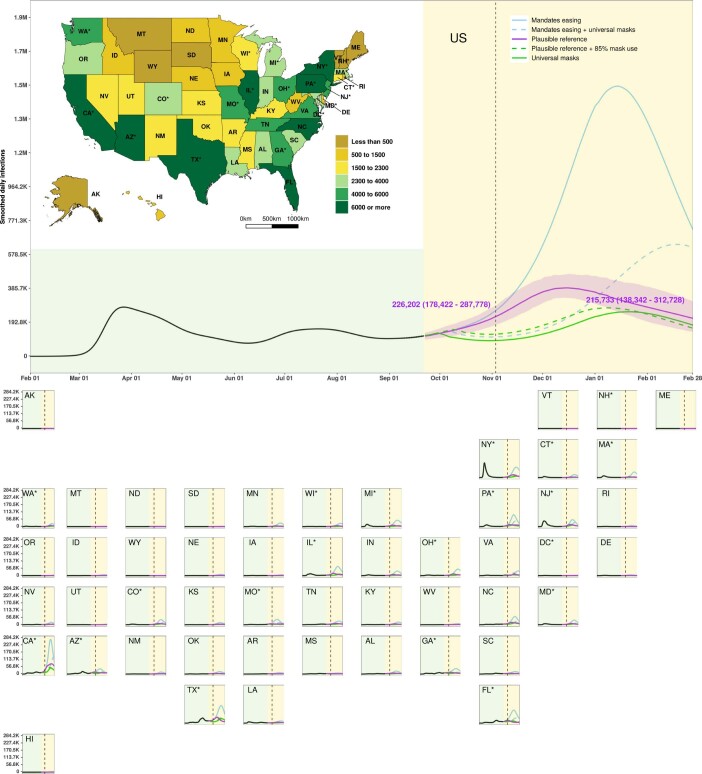
Estimated daily infections from SARS-CoV-2 from 01 February 2020 to 28 February 2021 by state, for all five scenarios. The inset map displays the estimated daily infections under the plausible reference scenario on 28 February 2021. The light yellow background separates the observed and predicted part of the time series, before and after 21 September 2020. The dashed vertical line identifies 03 November 2020. Numbers are the means and uncertainty interval (UI) for the plausible reference scenario on dates highlighted. The UIs are shown only for the plausible reference scenario. Source data

**Extended Data Fig. 8:**
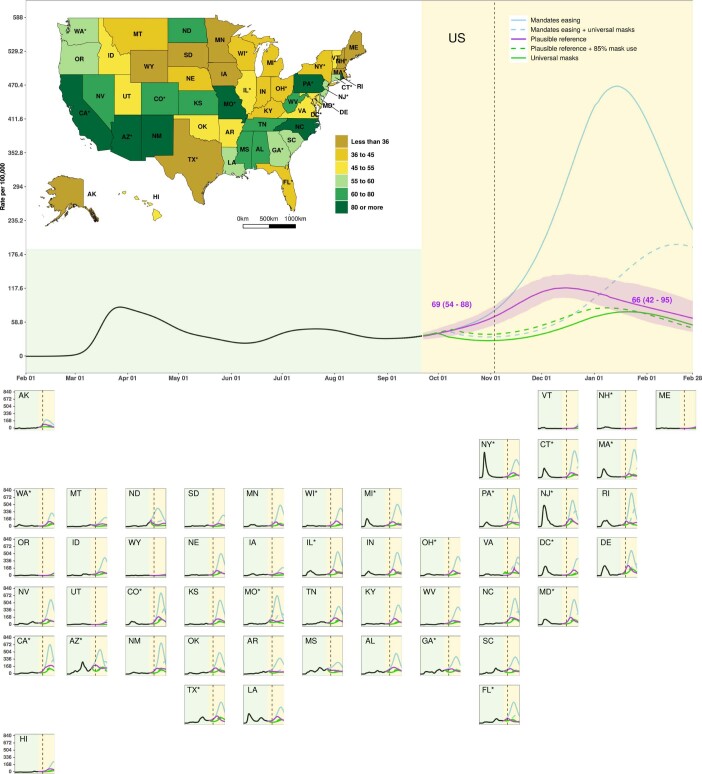
Estimated daily SARS-CoV-2 infection rate (per 100,000 population) by state, for all five scenarios. The inset map displays the estimated daily infections from COVID-19 per 100,000 population by state on 28 February 2021. The light yellow background separates the observed and predicted part of the time series, before and after 21 September 2020. The dashed vertical line identifies 03 November 2020. Numbers are the means and uncertainty interval (UI) for the plausible reference scenario on dates highlighted. The UIs are shown only for the plausible reference scenario. Source data

Under a boundary scenario where states continue with removal of SDMs (mandate easing), our model projections show that cumulative total deaths across the United States could reach 1,053,206 (759,693–1,452,397) by 28 February 2021 (Fig. 2 and Table 1). At the state level, contributions to that death toll would be heterogeneously distributed across the United States. Approximately one-third of the deaths projected from 22 September 2020 to 28 February 2021 in this scenario would occur across just three states: California (146,501 (84,828–221,194) deaths), Florida (66,943 (40,826–96,282) deaths) and Pennsylvania (62,352 (30,318–93,164) deaths). The highest cumulative death rates (per 100,000) from 22 September 2020 to 28 February 2021 are predicted to occur in Rhode Island (605.1 (428.1–769.0) deaths per 100,000)), Massachusetts (561.4 (315.8–901.3) deaths per 100,000), Connecticut (547.8 (209.3–978.2) deaths per 100,000) and Pennsylvania (541.1 (294.7–778.3) deaths per 100,000; Extended Data Fig. 4 and Table 1). By the US national election on 3 November 2020, a total of five states are predicted to exceed a threshold of daily deaths of 8 deaths per million (Fig. 3), and a total of 40 states would have an *R*_*effective*_ greater than one (Fig. 4). By 28 February 2021, a total of 45 states are predicted to exceed that threshold under this scenario, and all states would reach an *R*_*effective*_ of greater than one before the end of February 2021 (Table 1 and Fig. 4). This scenario results in an estimated total of 152,775,751 (115,305,817–199,130,145) infections across the United States by the end of February 2021 (Extended Data Fig. 5). The highest infection levels in states relative to their population size are estimated to occur in Arizona (71.2% (61.5–80.8%) infected), New Jersey (68.2% (47.5–84.1%) infected) and Rhode Island (65.5% (50.0–79.7%) infected; Extended Data Fig. 6). Further results for projected hospital resource-use needs are presented in Extended Data Figs. 2 and 3, and forecasted infections under this scenario are available in Extended Data Figs. 7 and 8.

**Table 1:** Cumulative deaths from 22 September 2020 through 28 February 2021, maximum estimated daily deaths per million, date of maximum daily deaths and estimated *R*_*effective*_ on 28 February 2021 for three boundary scenarios

	Mandate-easing scenario				Reference scenario				Universal mask-use scenario			
Location	Cumulative deaths through 28 February 2021	Maximum estimated daily deaths per million	Date of maximum daily deaths	Estimated R_*effective*_ on 28 February 2021	Cumulative deaths through 28 February 2021	Maximum estimated daily deaths per million	Date of maximum daily deaths	Estimated R_*effective*_ on 28 February 2021	Cumulative deaths through 28 February 2021	Maximum estimated daily deaths per million	Date of maximum daily deaths	Estimated R_*effective*_ on 28 February 2021
United States	1,053,206 (759,693–1,452,397)	36.8 (22.2–58.2)	2/1/21	NA	511,373 (469,578–578,347)	9 (7.7–10.8)	1/3/21	NA	381,798 (336,479–421,953)	5.7 (4.1–7.2)	2/8/21	NA
California	161,608 (99,935–236,301)	57.4 (29.4–97.7)	1/30/21	0.72 (0.56–0.83)	79,721 (59,551–115,314)	17.1 (10–31)	2/9/21	0.87 (0.83–0.91)	47,604 (33,315–59,781)	10.3 (4.9–14.2)	2/3/21	0.84 (0.78–0.90)
Florida	80,372 (54,255–109,711)	38.9 (20.5–65.5)	1/19/21	0.75 (0.63–0.83)	30,321 (26,885–34,509)	8.5 (4.2–13.6)	11/22/20	1 (0.84–1.13)	29,813 (23,434–32,599)	6.7 (0.9–11.2)	1/8/21	0.77 (0.61–1.03)
New York	74,392 (42,508–124,731)	35.1 (8.4–80.2)	2/13/21	0.82 (0.57–1)	48,225 (42,312–58,216)	9.4 (4.1–17.4)	1/18/21	0.81 (0.67–0.95)	39,185 (34,507–48,002)	4.8 (1.1–12.1)	2/28/21	0.93 (0.63–1.20)
Pennsylvania	70,325 (38,291–101,138)	72.4 (25.4–141)	1/18/21	0.68 (0.51–0.83)	24,401 (19,274–35,847)	11.9 (6.6–18.5)	12/11/20	0.91 (0.85–1.03)	18,636 (15,932–21,673)	8.9 (4–15.3)	1/12/21	0.85 (0.71–1.01)
Texas	68,869 (48,205–95,486)	26.3 (15.2–44.6)	2/6/21	0.76 (0.65–0.87)	38,266 (35,595–41,524)	8.9 (3.5–14.1)	1/1/21	0.85 (0.73–0.99)	31,945 (23,134–38,995)	6.6 (2.2–12)	2/14/21	0.78 (0.6–1.1)
Illinois	45,452 (27,394–66,503)	41.3 (17.9–73.4)	1/27/21	0.74 (0.59–0.84)	20,231 (18,473–23,034)	10.7 (6.7–15.9)	12/18/20	0.91 (0.83–1)	14,427 (11,603–17,696)	5.9 (2.5–10.8)	2/27/21	0.87 (0.61–1.09)
New Jersey	41,116 (26,328–53,040)	42.4 (10.3–86.9)	1/18/21	0.67 (0.48–0.87)	25,375 (21,966–31,245)	12.5 (4.0–21.9)	12/27/20	0.85 (0.76–0.93)	22,074 (17,978–24,938)	8.6 (1.6–17.2)	1/21/21	0.8 (0.64–1.06)
Massachusetts	37,421 (21,047–60,070)	82.2 (34.3–151.1)	2/13/21	0.76 (0.56–0.92)	15,265 (14,410–16,616)	11.1 (5.6–18.7)	12/28/20	0.95 (0.83–1.09)	12,853 (10,640–14,578)	8.1 (2.3–15.3)	2/15/21	0.77 (0.61–1.19)
Michigan	35,052 (17,757–56,639)	42.1 (14.5–82.2)	2/2/21	0.77 (0.59–0.91)	14,723 (13,425–17,959)	9 (4.0–16.1)	12/22/20	0.91 (0.77–1.07)	10,857 (8,509–14,137)	5.2 (1.4–11.4)	2/28/21	0.89 (0.59–1.15)
North Carolina	33,376 (20,637–48,331)	41.4 (21.9–70.3)	1/31/21	0.73 (0.59–0.84)	15,841 (12,988–20,712)	11.4 (7.0–16)	12/28/20	0.87 (0.83–0.95)	11,311 (7,628–14,094)	8.8 (4.1–13.7)	2/1/21	0.81 (0.72–1.02)
Ohio	31,340 (15,582–59,015)	41.3 (16.4–78)	2/18/21	0.84 (0.67–0.97)	14,726 (12,604–17,230)	10.6 (6.0–15.7)	1/5/21	0.86 (0.78–0.95)	8,760 (6,455–11,897)	5.4 (1.7–10.5)	2/28/21	0.93 (0.66–1.1)
Georgia	28,981 (20,312–39,881)	23.7 (13–40.3)	1/30/21	0.77 (0.66–0.87)	16,137 (12,811–19,435)	7.1 (1.7–12.3)	12/18/20	0.9 (0.77–1.04)	13,406 (10062–16,710)	5.5 (2.8–9.6)	10/17/20	0.85 (0.63–1.05)
Missouri	28,823 (17,420–38,804)	55.4 (24.5–95.2)	1/9/21	0.68 (0.53–0.79)	8,916 (6,562–13,898)	9.1 (3.1–16.2)	11/7/20	0.92 (0.79–1.12)	6,468 (4,236–7,732)	6 (2–13.3)	10/19/20	0.84 (0.63–1.11)
Indiana	20,541 (12,126–31,620)	38.9 (17.4–69.1)	2/1/21	0.75 (0.60–0.86)	8,999 (8,203–10,209)	10.7 (6.2–16.2)	12/24/20	0.88 (0.80–0.97)	6,037 (4,606–7,925)	5.4 (1.9–10.4)	2/28/21	0.89 (0.6–1.09)
Connecticut	20,236 (7,729–36,134)	77.8 (14.5–173.2)	2/6/21	0.76 (0.49–1)	8,440 (6,677–12,584)	14.1 (5.3–24.2)	1/8/21	0.88 (0.78–0.93)	6,085 (4,796–7,707)	7.2 (1.3–16.4)	2/15/21	0.86 (0.66–1.2)
Arizona	20,061 (16,934–23,195)	22.3 (12.1–36.8)	1/4/21	0.69 (0.57–0.78)	12,641 (10,609–15,248)	8.1 (3.8–13.4)	11/27/20	0.93 (0.77–1.12)	10,512 (8,022–12,062)	6.4 (0.3–12.6)	2/1/21	0.79 (0.61–1.15)
Colorado	19,048 (8,823–28,900)	50.8 (16.2–104.1)	1/27/21	0.68 (0.47–0.86)	7679 (5827–11,652)	12.8 (5.9–20.9)	1/4/21	0.85 (0.79–0.95)	5,170 (3,001–6,984)	8.2 (2.4–15.2)	2/6/21	0.77 (0.63–1.08)
Maryland	18,909 (10,802–29,780)	39.8 (16–73.1)	2/3/21	0.74 (0.56–0.86)	9,374 (8,224–11,424)	11.7 (7.4–17.4)	1/3/21	0.87 (0.79–0.95)	7,175 (5,684–8,566)	7.6 (4–13.1)	2/13/21	0.76 (0.62–1.03)
Minnesota	17,074 (8,030–31,654)	45.3 (17–89.5)	2/9/21	0.78 (0.59–0.91)	6,140 (5,551–6,945)	9.9 (5.3–17.1)	12/29/20	0.85 (0.72–0.99)	4,384 (3,031–5,868)	5.5 (1.9–11.3)	2/14/21	0.82 (0.55–1.05)
Virginia	15,369 (4,481–39,116)	39.2 (4.7–96.7)	2/28/21	0.72 (0.55–0.85)	9,588 (3,883–21,963)	16.3 (1.60–54.4)	1/25/21	0.89 (0.80–1.04)	10,472 (7,005–14,528)	7 (1.1–12.6)	1/14/21	0.86 (0.69–1.09)
Alabama	14,326 (8,692–20,493)	30.1 (13.3–55.5)	1/19/21	0.73 (0.59–0.83)	7,967 (6,485–10,627)	9.9 (4.1–15.1)	12/30/20	0.85 (0.79–0.98)	5,327 (3,501–7,137)	5.3 (0.9–9.7)	2/12/21	0.86 (0.68–1.02)
South Carolina	13,695 (9,516–18,801)	25.3 (14.5–42.5)	2/4/21	0.79 (0.68–0.89)	7,718 (6,769–8,644)	7.9 (2.8–12.7)	12/17/20	0.91 (0.79–1.05)	5,812 (4,408–7,709)	4.8 (0.9–9)	2/28/21	0.91 (0.64–1.06)
Tennessee	13,202 (5,011–21,540)	22.6 (5.1–42.9)	2/6/21	0.79 (0.64–1)	7,819 (5,011–9,577)	8.5 (2.1–14.3)	1/3/21	0.84 (0.72–0.95)	5,207 (2,969–7,608)	5.1 (0.7–10.1)	2/17/21	0.88 (0.66–1.06)
Louisiana	12,841 (9,085–17,601)	23.8 (12.8–40.3)	2/13/21	0.8 (0.68–0.95)	9,546 (8,523–10,628)	9.8 (5.2–14.4)	1/11/21	0.83 (0.75–0.92)	7,347 (6,144–9,052)	5.1 (1.2–9.3)	2/28/21	0.92 (0.68–1.07)
Nevada	10,950 (6,285–16,934)	46.2 (22.3–83.1)	2/8/21	0.76 (0.60–0.89)	4,812 (4,137–5,995)	10.3 (4.8–16.3)	1/1/21	0.90 (0.82–1.02)	3,389 (2,267–4,468)	7.1 (2.5–12.7)	2/20/21	0.82 (0.65–1.12)
Kansas	10,345 (1,683–19,571)	51.9 (3.9–116.2)	1/28/21	0.76 (0.52–1.05)	3,906 (1,683–6,711)	12.3 (2.2–22)	1/8/21	0.84 (0.69–0.96)	2,147 (892–3,295)	6.4 (0.6–13.2)	1/27/21	0.84 (0.66–1.07)
New Mexico	10,135 (5,818–15,160)	67.6 (31.2–120.1)	1/28/21	0.69 (0.52–0.82)	3,798 (3,035–5,683)	12.6 (7.2–17.8)	12/26/20	0.88 (0.84–0.95)	2,424 (1,796–3,068)	9.2 (4.8–13.7)	2/5/21	0.79 (0.69–0.95)
Wisconsin	10,132 (3,850–22,064)	29.7 (8.8–62.3)	2/22/21	0.87 (0.66–1.04)	5,265 (3,850–6,278)	9 (3.7–15.3)	1/18/21	0.79 (0.66–0.92)	2,409 (1,674–3,896)	3.1 (0.7–8.2)	2/28/21	0.98 (0.66–1.12)
Arkansas	10,061 (4,186–16,701)	34 (11.3–64.9)	2/2/21	0.81 (0.67–0.97)	3,805 (2,983–4,848)	6.8 (2–13.2)	10/25/20	0.93 (0.73–1.14)	2,999 (1,914–4,109)	6.1 (2.1–12.6)	10/17/20	0.88 (0.63–1.05)
Oklahoma	10,040 (5,661–15,338)	29.4 (13.3–53.5)	1/24/21	0.74 (0.59–0.85)	4,101 (3,446–5,304)	8.5 (2.9–14.4)	12/17/20	0.91 (0.78–1.05)	3,234 (1,998–4,188)	5.5 (1.6–10.7)	2/1/21	0.81 (0.58–1.01)
Washington	9,814 (5,067–17,772)	20.5 (7.9–39.3)	2/18/21	0.97 (0.87–1.12)	6,246 (4,705–8,574)	7.9 (4.4–12.3)	1/27/21	0.89 (0.7–1.1)	3,695 (2,716–5,024)	3.6 (1–6.7)	2/25/21	0.95 (0.90–1.03)
Kentucky	9,145 (2,956–17,672)	29.9 (6.6–62.8)	2/11/21	0.81 (0.61–1.03)	4,452 (2,956–5,660)	9.4 (2.6–16.5)	1/7/21	0.82 (0.66–0.95)	2,310 (1,470–3,798)	4 (0.6–9.7)	2/28/21	0.93 (0.62–1.09)
Mississippi	8,579 (6,359–11,950)	17.4 (9.1–29.8)	1/29/21	0.8 (0.71–0.9)	5,589 (4,524–7,006)	8.1 (3.5–17.7)	10/21/20	0.90 (0.76–1.07)	4,675 (3,911–5,813)	7.8 (3.5–16.6)	10/17/20	0.91 (0.67–1.02)
Rhode Island	6,319 (4,470–8,029)	75.2 (36.7–122.2)	1/17/21	0.66 (0.52–0.77)	2,317 (2,043–2,774)	12.2 (7.9–17.6)	11/30/20	0.94 (0.85–1.1)	1,946 (1,793–2,063)	9 (4.2–14.7)	1/10/21	0.84 (0.70–1.01)
Nebraska	5,915 (1,913–11,379)	47.1 (11.1–97.1)	2/5/21	0.77 (0.57–0.96)	2,156 (1,615–2,854)	10.8 (4.2–17.4)	12/31/20	0.84 (0.71–0.95)	1,298 (710–1,862)	5.8 (1.2–11.9)	2/8/21	0.82 (0.62–1.04)
West Virginia	5,071 (1,441–10,594)	41.6 (9.8–86.3)	2/17/21	0.86 (0.66–1.05)	2,153 (1,374–3,073)	9.6 (3.7–14.8)	1/10/21	0.89 (0.76–0.99)	1302 (630–2,030)	6.2 (1.4–11.3)	2/21/21	0.89 (0.71–1.11)
Iowa	4,510 (1,887–11,237)	18.4 (2.7–51)	2/28/21	0.94 (0.79–1.1)	3,232 (1,887–4,193)	6.5 (1.4–13.1)	2/1/21	0.82 (0.66–1.08)	1,910 (1,482–3,042)	2.8 (1.3–5.3)	10/6/20	0.95 (0.89–1.03)
Idaho	4,144 (899–9,028)	37.5 (4.1–84.6)	2/19/21	0.85 (0.63–1.09)	1,823 (899–2,521)	8 (1.6–15.2)	1/17/21	0.86 (0.68–1.08)	1,110 (600–1,951)	4.9 (0.7–11.4)	2/27/21	0.89 (0.64–1.10)
North Dakota	3,254 (1,813–4,744)	42 (14.9–81)	12/27/20	0.74 (0.61–0.84)	994 (623–1,639)	14.4 (5.2–30.4)	10/20/20	0.93 (0.79–1.13)	661 (464–951)	13.2 (4.8–27.1)	10/18/20	0.93 (0.66–1.07)
Delaware	2,559 (1,722–3,370)	27.1 (12.2–45.3)	1/16/21	0.72 (0.58–0.82)	1,476 (1,316–1,754)	10.9 (6.2–15.9)	12/18/20	0.89 (0.82–0.95)	1,222 (963–1,375)	7.1 (3.1–12.7)	1/24/21	0.77 (0.63–1)
Montana	1,671 (387–4,215)	21 (2.5–51.7)	2/27/21	0.92 (0.77–1.08)	922 (387–1,272)	6.2 (0.8–12)	1/25/21	0.88 (0.71–1.09)	450 (249–900)	4.2 (1.7–9.7)	10/6/20	0.96 (0.85–1.05)
Hawaii	1,576 (389–4,692)	22.9 (2.4–75.3)	2/28/21	0.99 (0.84–1.15)	1,035 (389–1,676)	7.7 (1.7–13.1)	2/2/21	0.86 (0.71–1.08)	448 (244–891)	3.1 (0.6–9)	2/28/21	0.98 (0.74–1.14)
South Dakota	1,370 (436–3,479)	25.8 (3.6–66)	2/28/21	0.92 (0.74–1.1)	765 (436–933)	6.5 (1.4–13.6)	1/17/21	0.80 (0.60–1.07)	408 (284–750)	3.2 (2.2–4.4)	10/1/20	0.95 (0.60–1.08)
District of Columbia	1,339 (863–2,450)	29.3 (8.9–67)	2/28/21	0.94 (0.74–1.09)	1,039 (863–1,220)	10 (4.8–16.3)	2/2/21	0.79 (0.68–0.89)	723 (659–862)	3.2 (0.6–9.2)	2/28/21	1.07 (0.72–1.25)
Oregon	1,104 (666–2,065)	4.1 (0.4–14.4)	2/28/21	1.09 (0.96–1.29)	1,096 (666–2,065)	3.9 (0.4–12.4)	2/28/21	1.06 (0.75–1.27)	661 (600–806)	0.7 (0.7–0.7)	9/22/20	1.02 (0.96–1.12)
Utah	959 (506–3,110)	5.3 (0.3–34.5)	2/28/21	1.07 (0.93–1.25)	891 (506–2,180)	3.5 (0.3–12.4)	2/28/21	1 (0.60–1.24)	518 (472–687)	0.4 (0.3–0.6)	9/29/20	1.01 (0.95–1.12)
Alaska	733 (74–2,524)	9.3 (0.2–46.7)	12/27/20	0.81 (0.58–0.97)	406 (74–1174)	4.4 (0.2–16.7)	12/9/20	0.83 (0.62–0.97)	250 (67–707)	2 (0.1–11.7)	12/10/20	0.81 (0.63–0.96)
New Hampshire	539 (451–810)	2.5 (0.2–11.6)	2/28/21	1.07 (0.95–1.27)	539 (451–810)	2.5 (0.2–11.2)	2/28/21	1.05 (0.75–1.26)	447 (440–466)	0.2 (0.2–0.2)	9/22/20	0.98 (0.93–1.07)
Maine	279 (149–769)	3 (0–17.2)	2/28/21	1.07 (0.96–1.25)	274 (149–755)	2.6 (0–12.1)	2/28/21	1.04 (0.73–1.23)	161 (145–202)	0.3 (0.3–0.3)	9/22/20	0.99 (0.91–1.09)
Vermont	119 (66–426)	5.1 (0.2–40.3)	2/28/21	1.14 (0.91–1.45)	113 (66–370)	3.6 (0.2–19.3)	2/28/21	1.06 (0.79–1.37)	69 (63–87)	0.4 (0.1–1.8)	2/28/21	0.99 (0.76–1.32)
Wyoming	115 (53–446)	2.4 (0–18.3)	2/28/21	0.99 (0.84–1.13)	108 (53–387)	1.6 (0–9.2)	2/28/21	0.97 (0.71–1.13)	65 (53–104)	0.7 (0.7–0.7)	9/22/20	0.92 (0.7–1.02)

Results for two additional derivative scenarios are available in the Supplementary Information. NA, not applicable.

When we modeled the future course of the epidemic assuming that states will once again shut down social interaction and economic activity when daily deaths reach a threshold of 8 deaths per million (plausible reference scenario), the projected cumulative death toll across the United States is forecast to be lower than that under the mandate-easing scenario, with 511,373 (469,578–578,347) deaths by 28 February 2021 (Fig. 2). Thus, across the 45 states that are projected to exceed daily deaths of 8 deaths per million under the mandate-easing scenario by the end of February 2021 (Table 1), the reinstatement of SDMs under the plausible reference scenario could save 541,738 (281,283–886,373) lives. This scenario also results in 80,798,356 (47,333,280–121,526,052) fewer estimated infections across the United States by the end of February 2021 (Extended Data Fig. 5) compared with the mandate-easing scenario, with the highest rates of infections estimated to occur in Arizona (46.2% (38.8–55.9%) infected), New Jersey (41.1% (35.1–50.8%) infected) and Louisiana (33.3% (29.9–37.4%) infected) (Extended Data Fig. 6). As with the previous scenario, even with the reinstatement of SDMs when daily deaths exceed 8 per million population, all states would reach an *R*_*effective*_ greater than one before the end of the February 2021 (Fig. 4 and Table 1). Further results for hospital resource-use needs are presented in Extended Data Figs. 2 and 3 and forecast infections by state under this scenario are presented in Extended Data Figs. 7 and 8.

The universal mask-use scenario where the population of each state was assumed to adopt and maintain a 95% level of mask use in public (Methods)—in addition to states reinstating SDM if a threshold daily death rate of 8 deaths per million population was exceeded—resulted in the lowest projected cumulative death toll across US states, with a total of 381,798 (336,479–421,953) cumulative deaths by 28 February 2021 (Fig. 2 and Table 1). Under this scenario, on 3 November 2020, no states will have exceeded a daily death rate of 8 deaths per million (Fig. 3), although 47 states are still estimated to exceed an *R*_*effective*_ of 1.0 at some point in the projected period, and three states would have an *R*_*effective*_ greater than 1.0 on 28 February 2021 (Fig. 4). Through the end of the February 2021, the daily death rate is forecast to exceed 8 deaths per million in nine states (California, Colorado, Massachusetts, New Jersey, New Mexico, North Carolina, North Dakota, Pennsylvania and Rhode Island; Table 1) saving 129,574 (85,284–170,867) lives when compared to the plausible reference scenario and 671,407 (376,883–1,046,250) lives when compared to the mandate-easing scenario. Universal mask use combined with threshold-driven implementation of SDM results in 17,408,352 (11,278,442–23,291,371) fewer estimated infections across the United States by the end of February 2021 compared with the plausible reference scenario, and 98,106,708 (59,908,817–142,318,907) fewer estimated infections compared to the mandate-easing scenario (Extended Data Fig. 5). The highest infection rates under the 95% mask-use scenario are estimated to occur in Arizona (38.1% (28.0–43.3%) infected), New Jersey (35.7% (30.2–41.0%) infected) and Delaware (28.2% (23.3–31.1%) infected) (Extended Data Fig. 6). Further results for hospital resource-use needs are presented in Extended Data Figs. 2 and 3, and forecast infections under this scenario are available in Extended Data Figs. 7 and 8.

To provide additional policy nuance to the three boundary scenarios, we also examined plausible reference + 85% mask use and mandate-easing + universal mask-use scenarios (Figs. 2–4, Extended Data Figs. 1 and 4–8 and Supplementary Information). In brief, the plausible reference + 85% mask-use scenario saves a considerable number of lives at the national level (95,814 (60,731–133,077) over and above the reference scenario, but is not as effective as the plausible reference + universal mask-use scenario. Although not surprising, this does help to confirm that any additional coverage that can be achieved through mask use will save lives. The mandate-easing + universal mask-use scenario reveals substantial lives saved (20,936 (0–102,507)) over the plausible reference scenario, even in the absence of reinstatement of SDMs at the daily threshold of 8 deaths per one million population, underscoring the potential effects that increased levels of mask adoption could have while minimizing the deleterious economic repercussions of other NPIs.

Two out-of-sample (OOS) model assessments were conducted for two different time intervals of the modeling period to investigate the strength of evidence behind each of the covariate drivers of SARS-CoV-2 transmission intensity. Full details of these sensitivity analyses are shown at the national level in the Supplementary Information. These analyses indicate that care needs to be taken in interpreting the strength of these relationships, which show variability in time and space. For example, our OOS tests indicate that over some time frames, pneumonia mortality seasonality was either the most or least useful covariate, despite in-sample tests having consistently shown this to be an important predictor. Since pneumonia seasonality is one of the leading covariates driving expected increases in COVID-19 deaths in the fall and winter, it is important to be aware of this uncertainty when assessing the forecasts. It is critical to note, however, that even when we completely remove this covariate from our model, sensitivity analyses show a forecast of over 100,000 deaths from COVID-19 by the end of winter (101,615 (81,479–126,295) additional deaths; Supplementary Information). Since this covariate complexity makes it difficult to generalize the effects of this uncertainty, we provide extensive diagnostics for the covariate relationships in each of the states with examples of how to interpret these findings (Supplementary Information).

### Model performance

The models presented here have been evaluated for OOS predictive validity using standard tests and metrics in an ongoing fashion and in a publicly available framework^21^. These SEIR models have consistently produced among the most accurate forecasts observed across models compared^21^. For example, for models released in June, the Institute for Health Metrics and Evaluation (IHME) SEIR model had the lowest median absolute percentage error (MAPE) at 10 weeks of forecasting at 20.2%, compared to 32.6% across models. We have included new sets of model and covariate diagnostics with worked descriptions for the most populous states (Supplementary Information and Supplementary Data 1–4) for transparent evaluation of our model performance. We emphasize that these are forecasts of possible futures, which are subject to many model assumptions and sources of data variability.

## Discussion

We have delimited three possible future scenarios of the course of the COVID-19 epidemic in the United States, at the state level—mandate-easing, plausible reference and universal mask-use scenarios—to help frame and inform a national discussion on what actions could be taken during the fall of 2020 and the public health, economic and political influences that these decisions will have for the rest of the winter (here defined as the end of February 2021). To help us understand the policy nuances of these boundary scenarios, two derivative scenarios (plausible reference + 85% mask use and mandate easing + universal mask use) were also explored. In addition, selected sensitivity analyses were conducted for the covariates used in the models, so that their influence could be better understood.

Under all scenarios evaluated here, the United States is likely to face a continued public health challenge from the COVID-19 pandemic through 28 February 2021 and beyond, with populous states in particular potentially facing high levels of illness, deaths and ICU demands as a result of the disease. The implementation of SDMs as soon as individual states reach a threshold of 8 daily deaths per million could dramatically ameliorate the effects of the disease; achieving near-universal mask use could delay, or in many states, possibly prevent, this threshold from being reached and has the potential to save the most lives while minimizing damage to the economy. National and state-level decision-makers can use these forecasts of the potential health benefits of available NPIs, alongside considerations of economic and other social costs, to make more informed decisions on how to confront the COVID-19 pandemic at the local level. Our findings indicate that universal mask use, a relatively affordable and low-impact intervention, has the potential to serve as a priority life-saving strategy in all US states. Our derivative scenarios suggest that this likely remains true at sub-universal levels of mask coverage and at universal mask coverage in the absence of any other NPIs.

New epidemics, resurgences and second waves are not inevitable. Several countries, such as South Korea, Germany and New Zealand have sustained reductions in COVID-19 cases over time (https://covid19.healthdata.org/). Early indications that seasonality may play a role in transmission, with increased spread during colder winter months as is seen with other respiratory viruses^29–32^, highlight the importance of taking action both before and during the pneumonia season in the United States. While it is yet unclear if COVID-19 seasonality will follow the pattern of related coronaviruses^32^ and parallel that of pneumonia seasonality, the sometimes strong associations observed in these forecasts indicate that increased government vigilance is prudent. Moreover, given the potential sensitivity of the model to effects of seasonality, a substantial winter effect cannot be ruled out. This effect would be against a background of more widespread and prevalent COVID-19 infection than experienced in the first wave.

Mask use has emerged as a contentious issue in the United States with only 49% of US residents reporting that they ‘always’ wear a mask in public as of 21 September 2020 (https://covid19.healthdata.org/). Regardless, toward the end of 2020, masks could help to contain a second wave of resurgence while reducing the need for frequent and widespread implementation of SDMs. Although 95% mask use across the population may seem a high threshold to achieve and maintain, on a neighborhood scale this level has already been observed in areas of New York (https://www.nytimes.com/2020/08/20/nyregion/nyc-face-masks.html); and on a state level, reported mask use has exceeded 60% in Virginia, Florida and California (see Supplementary Information for related methods). In countries where mask use has been widely adopted, such as Singapore, South Korea, Hong Kong, Japan and Iceland among others, transmission has declined and, in some cases, halted (https://covid19.healthdata.org/). These examples serve as additional natural experiments^33^ of the likely effects of masks and support the assumptions and findings from the universal mask-use scenario in our study. The potential life-saving benefit of increasing mask use in the coming fall and winter cannot be overstated. It is likely that US residents will need to choose between higher levels of mask use or risk the frequent redeployment of more stringent and economically damaging SDMs; or, in the absence of either measure, face a reality of a rising death toll^34^. Longer term, the future of COVID-19 in the United States will be determined by the deployment of an efficacious vaccine and the evolution of herd immunity^35^.

**Extended Data Fig. 9:**
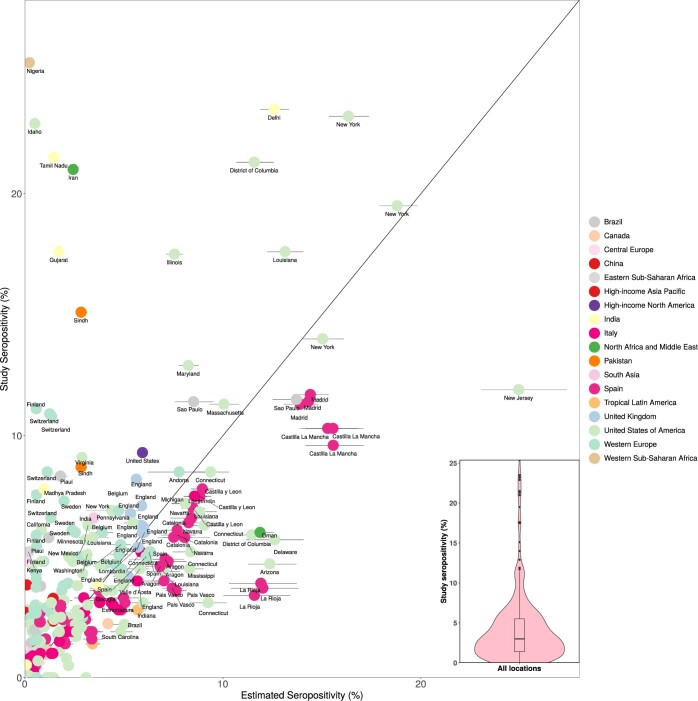
Modeled SARS-CoV-2 infection prediction totals compared with survey-derived seroprevalence rates in select locations. Modeled SARS-CoV-2 infection prediction totals compared with survey-derived seroprevalence rates in select locations globally. The scatter plots show locations colour coded by country; horizontal bars are the 95% confidence interval in the modeled estimates. The inset violin plot of the measured seropositivity data show the predominantly low values seropositivity estimates (below 5%) recorded in this global sample. Source data

This work represents the outputs of a class of models that aim to abstract the disease transmission process in populations to a level that is tractable for understanding, and, in this case, that can be used for prediction. A clear limitation of any such modeling exercise is that it will be constrained by data (disease and relevant covariates), the model of understanding developed and the length of time available to the model to learn/train the important dynamics. We have therefore tried to benchmark our model against alternative models of the COVID-19 pandemic and fully document our predictive performance with a range of measures^21^. In addition, we have provided all the data and model code to enable full reproducibility and increased transparency, provided sensitivity analyses to some of our core assumptions; and presented a range of likely futures^36^ in the form of mandate-easing, plausible reference and universal mask-use scenarios (as well as two derivative scenarios thereof) for decision-makers to review. In addition, triangulation of other outputs of the SEIR model, such as the proportion of the population that are affected, are also provided and tested against independent data, in this case seroprevalence surveys (Extended Data Fig. 9). Finally, because uncertainty compounds with increased distance into the future predicted, the data, model and its assumptions will be iteratively updated as the pandemic continues to unfold (https://www.latimes.com/opinion/story/2020-07-10/covid-forecast-deaths-ihme-washington/).

We wish to reiterate to decision-makers that there are a multitude of limitations in any modeling study of this type^26,27^; an extended description of the limitations specific to this study is provided (Methods). Specifically, (1) these models are approximations of real-world scenarios, and we have simplified many aspects of the epidemiological process of disease transmission to make these models computationally feasible; (2) these models are driven strongly by mortality data with all of its fidelity and recording imperfections; (3) these models are also informed by a wealth of other data types that each have differential availability, as well as detection and measurement bias issues for which we can never fully calibrate; (4) these models make particular assumptions about covariates, including seasonality, that while evidence-based and explicitly stated, are subject to sensitivity analyses because their effects could be substantial; and (5) our knowledge of this dynamic pandemic improves daily, so there should be no expectation that this modeling framework is final or that the data that drive it are fixed. While acknowledging all of these policy-relevant limitations, we take care to note that our publicly released model comparison framework^21^ supports the robust, iterative and objective evaluation of our modeling approach. This is especially valuable as the complexities of the pandemic response require that our modeling efforts remain agile to epidemiological and societal developments and that we continue to reevaluate and post updates weekly (https://covid19.healthdata.org/). Finally, it is especially important for decision-makers that we emphasize that we are not forecasting a future, but rather a range of outcomes that we believe are more probable given the scenarios tested, based on the data observed so far and our model assumptions. These forecasts are best considered as helpful guides, rather than definitive maps.

## Methods

Our analysis strategy supports two main and interconnected objectives: (1) to generate forecasts of COVID-19 deaths, infections and hospital resource needs for all US states; and (2) to explore alternative scenarios on the basis of changes in state-enforced SDMs or population-level mask use. The modeling approach to achieve this is summarized in the Supplementary Information and can be divided into four stages: (1) identification and processing of COVID-19 data, (2) exploration and selection of key drivers or covariates, (3) modeling deaths and cases across three boundary scenarios of SDMs in US states using an SEIR framework and (4) modeling health service utilization as a function of forecast infections and deaths within those scenarios. This study complies with the Guidelines for Accurate and Transparent Health Estimates Reporting statement (Supplementary Information).

### Data identification and processing

IHME forecasts include data from local and national governments, hospital networks and associations, the World Health Organization, third-party aggregators and a range of other sources. Data sources and corrections are described in detail in the Supplementary Information and in the data availability statement. Briefly, daily confirmed case and death numbers due to COVID-19 are collated from the Johns Hopkins University data repository; we supplement and correct this dataset as needed to improve the accuracy of our projections and adjust for reporting-day biases (Supplementary Information). Testing data are obtained from Our World in Data (https://ourworldindata.org/), The COVID Tracking Project (https://covidtracking.com/) and supplemented with data from additional government websites (Supplementary Information). Social distancing data are obtained from a number of different official and open sources, which vary by state (Supplementary Information). Mobility data are obtained from Facebook Data for Good (https://dataforgood.fb.com/docs/covid19/), Google (https://www.google.com/covid19/mobility/), SafeGraph (https://www.safegraph.com/dashboard/covid19-shelter-in-place/) and Descartes Labs (https://www.descarteslabs.com/mobility/; Supplementary Information). Mask-use data are obtained from the Facebook Global Symptom Survey (in collaboration with the University of Maryland Social Data Science Center), the Kaiser Family Foundation, YouGov COVID-19 Behavioural Tracker survey (https://today.yougov.com/covid-19/) and PREMISE (https://www.premise.com/covid-19/; Supplementary Information). Specific sources for data on licensed bed and ICU capacity and average annual utilization in the United States are detailed in the Supplementary Information.

Before modeling, observed cumulative deaths are smoothed using a spline-based smoothing algorithm with randomly placed knots^37^. Uncertainty is derived from bootstrapping and resampling of the observed deaths. The time series of case data is used as a leading indicator of death based on an infection fatality ratio (IFR) and a lag from infection to death. These smoothed estimates of observed deaths by location are then used to create estimated infections based on an age distribution of infections and on age-specific IFRs. The age-specific infections were collapsed into total infections by day and state and used as data inputs in the SEIR model. Detailed descriptions of data smoothing and transformation steps are provided in the Supplementary Information.

### Covariate selection

Covariates for the compartmental transmission SEIR model are predictors of the *β* parameter in the model that affects the transition from the susceptible to exposed state; specifically, *β* represents the contact rate multiplied by the probability of transmission per contact. Covariates were evaluated on the basis of biological plausibility and on the impact on the results of the SEIR model. Given limited empirical evidence of population-level predictors of SARS-CoV-2 transmission, biologically plausible predictors of pneumonia such as population density (percentage of the population living in areas with more than 1,000 individuals per square kilometer), tobacco smoking prevalence, population-weighted elevation, lower respiratory infection mortality rate and particulate matter air pollution were considered. These covariates are representative at a population level and are time invariant. Location-specific estimates for these covariates are derived from the Global Burden of Disease Study 2019 (refs. ^38–40^). Time-varying covariates include pneumonia excess mortality seasonality, diagnostic tests administered per capita, population-level mobility and personal mask use. These are described below.

#### Pneumonia seasonality

We used weekly pneumonia mortality data from the National Center for Health Statistics Mortality Surveillance System (https://gis.cdc.gov/grasp/fluview/mortality.html) from 2013 to 2019 by US state. Pneumonia deaths included all deaths classified by the full range of the International Classification of Disease codes in J12–J18.9. We pooled data over available years for each state and found the weekly deviation from the annual, state-specific mean mortality due to pneumonia. We then fit a seasonal pattern using a Bayesian meta-regression model with a flexible spline and assumed annual periodicity (Supplementary Information). For locations outside the United States, we used vital registration data where available. Locations without vital registration data had weekly pneumonia seasonality predicted based on latitude from a model pooling all available data (Supplementary Information).

#### Testing per capita

We considered diagnostic testing for active SARS-CoV-2 infections as a predictor of the ability for a state to identify and isolate active infections. We assumed that higher rates of testing were negatively associated with SARS-CoV-2 transmission. Our primary sources for US testing data were compiled by the COVID Tracking Project (Supplementary Information). Unless testing data existed before the first confirmed case in a state, we assumed that testing was non zero after the date of the first confirmed case. Before producing predictions of testing per capita, we smoothed the input data by using the same smoothing algorithm used for smoothing daily death data before modeling (previously described). Testing per capita projections for unobserved future days were based on linearly extrapolating the mean day-over-day difference in daily tests per capita for each location. We put an upper limit on diagnostic tests per capita of 500 per 100,000 based on the highest observed rates in June 2020.

#### Social distancing mandates

SDMs were not used as direct covariates in the transmission model. Rather, SDMs were used to predict population mobility (see below), which was subsequently used as a covariate in the transmission model. We collected the dates of state-issued mandates enforcing social distancing, as well as the planned or actual removal of these mandates. The measures that we included in our model were: (1) severe travel restrictions, (2) closing of public educational facilities, (3) closure of nonessential businesses, (4) stay-at-home orders and (5) restrictions on gathering size. Generally, these came from state government official orders or press releases.

To determine the expected change in mobility due to SDMs, we used a Bayesian, hierarchical meta-regression model with random effects by location on the composite mobility indicator to estimate the effects of social distancing policies on changes in mobility (Supplementary Information).

#### Mobility

We used four data sources on human mobility to construct a composite mobility indicator. Those sources were Facebook, Google, SafeGraph and Descartes Labs (Supplementary Information). Each source takes a slightly different approach to capturing mobility, so before constructing a composite mobility indicator, we standardized these different data sources (Supplementary Information). Briefly, this first involved determining the change in a baseline level of mobility for each location by data source. Then, we determined a location-specific median ratio of change in mobility for each pairwise comparison of mobility sources, using Google as a reference and adjusting the other sources by that ratio. The time series for mobility was estimated using a Gaussian process regression model using the standardized data sources to get a composite indicator for change in mobility for each location day.

We calculated the residuals between our predicted composite mobility time series and input composite time series, and then applied a first-order random walk to the residuals. The random walk was used to predict residuals from 1 January 2020 to 1 January 2021, which were then added to the mobility predictions to produce a final time series with uncertainty: ‘past’ changes in mobility from 1 January 2020 to 28 September 2020 and projected mobility from 28 September 2020 to 1 January 2021.

#### Masks

We performed a meta-analysis of 40 peer-reviewed scientific studies in an assessment of mask effectiveness for preventing respiratory viral infections (Supplementary Information). The studies were extracted from a preprint publication^24^. In addition, we considered all articles from a second meta-analysis^23^ and one supplemental publication^41^. These studies included both persons working in health care and the general population, especially family members of those with known infections. The studies indicate overall reductions in infections due to masks preventing exhalation of respiratory droplets containing viruses, as well as some prevention of inhalation by those uninfected. The resulting meta-regression calculated log-transformed relative risks and corresponding log-transformed standard errors based on raw counts and used a continuity correction for studies with zero counts in the raw data (0.001). We included additional specifications and characteristics to account for differences in the characteristics of individual studies and to identify important factors impacting mask effectiveness (Supplementary Information).

We used MR-BRT (meta-regression, Bayesian, regularized and trimmed), a meta-regression tool developed at the Institute for Health Metrics and Evaluation (Supplementary Information), to perform a meta-analysis that considered the various characteristics of each study. We accounted for between-study heterogeneity and quantified remaining between-study heterogeneity into the width of the UI. We also performed various sensitivity analyses to verify the robustness of the modeled estimates and found that the estimate of the effectiveness of mask use did not change significantly when we explored four alternative analyses, including changing the continuity correction assumption, using odds ratio versus relative risk from published studies, using a fixed-effects versus a mixed-effects model and including studies without information on covariates.

We estimated the proportion of people who self-reported always wearing a face mask when outside in public for both US and global locations using data from PREMISE (US), the Kaiser Family Foundation (US), YouGov (non-US) and Facebook (non-US) surveys (Supplementary Information). We used the same smoothing model as for COVID-19 deaths and testing per capita to produce estimates of observed mask use. This smoothing process averaged each data point with its neighbors. The level of mask use starting on 21 September 2020 (the last day of processed and analyzed data) was assumed to be flat. Among states without state-specific data, a within-the-US regional average was used.

### Deterministic modeling framework

Model specification is summarized in a schematic with additional details provided in the Supplementary Information. To fit and predict disease transmission dynamics, we include a SEIR component in our multistage model. In particular, the population of each location is tracked through the following system of differential equations:

$$\begin{array}{l}\frac{{dS}}{{dt}} = - \beta \left( t \right)\frac{{S\left( {I_1 + I_2} \right)^\alpha }}{N}\\ \frac{{dE}}{{dt}} = \beta \left( t \right)\frac{{S\left( {I_1 + I_2} \right)^\alpha }}{N} - \sigma E\\ \frac{{dI_1}}{{dt}} = \sigma E - \gamma _1I_1\\ \frac{{dI_2}}{{dt}} = \gamma _1I_1 - \gamma _2I_2\\ \frac{{dR}}{{dt}} = \gamma _2I_2\end{array}$$

where *α* represents a mixing coefficient to account for imperfect mixing within each location, *σ* is the rate at which infected individuals become infectious, *γ*_1_ is the rate at which infectious people transition out of the presymptomatic phase and *γ*_2_ is the rate at which individuals recover. This model does not distinguish between symptomatic and asymptomatic infections but has two infectious compartments (*I*_1_ and *I*_2_) to allow for interventions that would avoid focus on those who could not be symptomatic; *I*_1_ is thus the presymptomatic compartment.

Using the next-generation matrix approach, we can directly calculate both the basic reproductive number under control (*R*_*c*_(*t*)) and the effective reproductive number (*R*_*effective*_(*t*)) as (Supplementary Information):

$$R_c\left( t \right) = \alpha \times \beta \left( t \right) \times \left( {I_1\left( t \right) + I_2\left( t \right)} \right)^{\alpha - 1} \times \left( {\frac{1}{{\gamma _1}} + \frac{1}{{\gamma _2}}} \right)$$ and

$$R_{effective}\left( t \right) = R_c\left( t \right) \times \frac{{S\left( t \right)}}{N}$$

By allowing *β*(*t*) to vary in time, our model is able to account for increases in transmission intensity as human behavior shifts over time (for example, changes in mobility, adding or removing SDMs and changes in population mask use). Briefly, we combine data on cases (correcting for trends in testing), hospitalizations and deaths into a distribution of trends in daily deaths.

To fit this model, we resampled 1,000 draws of daily deaths from this distribution for each state (Supplementary Information). Using an estimated IFR by age and the distribution of time from infection to death (Supplementary Information), we then used the daily deaths to generate 1,000 distributions of estimated infections by day from 10 January to 21 September 2020. We then fit the rates at which infectious individuals may come into contact and infect susceptible individuals (denoted as *β*(*t*)) as a function of a number of predictors that affect transmission. Our modeling approach acts across the overall population (that is, no assumed age structure for transmission dynamics), and each location is modeled independently of the others (that is, we do not account for potential movement between locations).

We detail the SEIR fitting algorithm in the Supplementary Information. Briefly, for each draw, we first fit a smooth curve to our estimates of daily new infections. Then, sampling *γ*_2_, *σ* and *α* from defined ranges from the literature (Supplementary Information) and using $\gamma _1 = \frac{1}{2}$, we then sequentially fit the *E*, *I*_1_, *I*_2_ and *R* components in the past. We then algebraically solve the above system of differential equations for *β*(*t*).

The next stage of our model fit relationships between past changes in *β*(*t*) and covariates described above: mobility, testing, masks, pneumonia seasonality and others. The time-varying covariates were forecast from 28 September to 28 February 2021 (Supplementary Information). The fitted regression was then used to estimate future transmission intensity *β*_*pred*_(*t*). The final future transmission intensity is then an adjusted version of *β*_*pred*_(*t*) based on the average fit over the recent past (where the window of averaging varies by draw from 2 to 4 weeks; Supplementary Information).

Finally, we used the future estimated transmission intensity to predict future transmission (using the same parameter values for all other SEIR parameters for each draw). In a reversal of the translation of deaths into infections, we then used the estimated daily new infections to calculate estimated daily deaths (again using the location-specific IFR). We also used the estimated trajectories of each SEIR compartment to calculate *R*_*c*_ and *R*_*effective*_.

A final step to take predicted infections and deaths and a hospital-use microsimulation to estimate hospital resource need for each US state is described in the Supplementary Information and the results are presented online (https://covid19.healthdata.org/).

### Forecasts/scenarios

Policy responses to COVID-19 can be supported by the evaluation of the impacts of various scenarios of those options, against a background of a business-as-usual assumption, to explore fully the potential impact of policy levers available. Additional details are available in the Supplementary Information.

We estimate the trajectory of the epidemic by state under a mandate-easing scenario that models what would happen in each state if the current pattern of easing SDMs continues and new mandates are not implemented. This should be thought of as a worst-case scenario where, regardless of how high the daily death rate becomes, SDMs will not be reintroduced and behavior (including population mobility and mask use) will not vary before 28 February 2021. In locations where the number of cases is rising, this leads to very high numbers of cases by the end of the year.

As a more plausible scenario, we use the observed experience from the first phase of the pandemic to predict the likely response of state and local governments during the second phase. This plausible reference scenario assumes that in each location the trend of easing SDMs will continue at its current trajectory until the daily death rate reaches a threshold of 8 deaths per million. If the daily death rate in a location exceeds that threshold, we assume that SDMs will be reintroduced for a 6-week period. The choice of threshold (of a daily rate of 8 deaths per million) represents the 90th percentile of the distribution of daily death rate at which US states implemented their mandates during the first months of the COVID-19 pandemic. We selected the 90th percentile rather than the 50th percentile to capture an anticipated increased reluctance from governments to reinstate mandates because of the economic effects of the first set of mandates. In locations that do not exceed the threshold of a daily death rate of 8 per million, the projection is based on the covariates in the model and the forecasts for these to 28 February 2021. In locations where the daily death rate exceeded 8 per million at the time of running our final model (21 September 2020), we assumed that mandates would be introduced within 7 days.

The scenario of universal mask use models what would happen if 95% of the population in each state always wore a mask when they were in public. This value was chosen to represent the highest observed rate of mask use in the world so far during the COVID-19 pandemic (Supplementary Information). In this scenario, we also assumed that if the daily death rate in a state exceeds 8 deaths per million, SDMs will be reintroduced for a 6-week period.

Two additional, derivative scenarios were included to assist understanding and policy resolution of these main framework scenarios: a less comprehensive mask-wearing scenario of 85% public use of masks and a scenario of universal mask use in the absence of any additional NPIs. The less comprehensive mask-wearing scenario evaluated what would happen if 85% of the population in each state always wore a mask when they were in public. As with the universal mask-use scenario, we also assumed that if the daily death rate in a state exceeds 8 deaths per million, SDMs will be reintroduced for a 6-week period. For completeness, we also evaluated universal mask use by 95% of the population in a scenario that assumes no implementation of other NPIs at any threshold value of daily deaths—the results from this scenario, which did not differ notably from the more probable version where states respond to rising numbers of daily COVID-19 deaths by reinstating SDM, are provided in the Supplementary Information and Figs. 2–4. SEIR model vetting plots for scenarios of 95% mask use with mandates (Supplementary Data 1), 95% mask use without mandates (Supplementary Data 2) and 85% mask use with mandates (Supplementary Data 3), as well as detailed regression diagnostics (Supplementary Data 4) and the spatial distribution of select covariates (Supplementary Data 5) are available in the Supporting Information. All scenarios assume an increase in mobility associated with the opening of schools across the country.

### Model validation

OOS predictive performance for IHME SEIR models has been assessed against subsequently observed trends in an ongoing fashion and compared to other publicly available COVID-19 mortality forecasting models in a publicly available framework^21^. The IHME SEIR model described here has consistently demonstrated high accuracy, as measured by a low MAPE, when compared to models from other groups. For example, among models released in June, at 10 weeks of extrapolation, the IHME SEIR model had the lowest MAPE of any observed forecasting group at 20.2%, compared to an average of 32.6% across groups. Numerous other aspects of predictive performance are assessed in our publicly available framework^21^.

The increasing number of population-based serology surveys conducted also provides a unique opportunity to cross-validate our forecasts with modeled epidemiological outcomes. In Extended Data Fig. 9, we compare these serology surveys (such as the Spanish ENE-COVID study^42^) to our estimated population seropositivity, time indexed to the date that the survey was conducted. In general, across the varied locations that have been reported globally, we note a high degree of agreement between the estimated and surveyed seropositivity. As more serology studies are conducted and published, especially in the United States, this will allow an ongoing and iterative assessment of model validity. Two sensitivity analyses were conducted; the first assessed the importance of specific model assumptions on OOS predictive validity, while the second assessed the robustness of our conclusions to these same model assumptions (Supplementary Information).

### Limitations

Epidemics progress based on complex nonlinear and dynamic biological and social processes that are difficult to observe directly and at scale. Mechanistic models of epidemics, formulated either as ordinary differential equations or as individual-based simulation models, are a useful tool for conceptualizing, analyzing or forecasting the time course of epidemics. In the COVID-19 epidemic, effective policies and the responses to those policies have changed the conditions supporting transmission from one week to the next, with the effects of policies realized typically after a variable time lag. Each model approximates an epidemic, and whether used to understand, forecast or advise, there are limitations on the quality and availability of the data used to inform it and the simplifications chosen in model specification. It is unreasonable to expect any model to do everything well, so each model makes compromises to serve a purpose, while maintaining computational tractability.

One of the largest determinants of the quality of a model is the corresponding quality of the input data. Our model is anchored to daily COVID-19-related deaths, as opposed to daily COVID-19 case counts, due to the assumption that death counts are a less biased estimate of true COVID-19-related deaths than COVID-19 case counts are of the true number of SARS-CoV-2 infections. Numerous biases such as treatment-seeking behavior, testing protocols (such as only testing those who have traveled abroad) and differential access to care greatly influence the utility of case count data. Moreover, there is growing evidence that inapparent and asymptomatic individuals are infectious, as well as individuals who eventually become symptomatic and are infectious before the onset of any symptoms. As such, our primary input data for our model are counts of deaths; death data can likewise be fallible, however, and where available, we combine death data, case data and hospitalization data to estimate COVID-19 deaths.

Beyond the basic input data, a large number of other data sources with their own potential biases are incorporated into our model. Testing, mobility and mask use are all imperfectly measured and may or may not be representative of the practices of those that are susceptible and/or infectious. Moreover, any forecast of the patterns of these covariates is associated with a large number of assumptions (Supplementary Information), and as such, care must be taken in the interpretation of estimates farther into the future, as the uncertainty associated with the numerous submodels that go into these estimates increases in time. Moreover, although our time-invariant covariates are simpler to estimate, some of them may be more associated with disease outcome than transmission potential, and thus their impact on the model may be more muted.

For practical purposes, our transmission model has made a large number of simplifying assumptions. Key among these is the exclusion of movement between locations (for example, importation) and the absence of age structure and mixing within location (for example, we assume a well-mixed population). It is clear that there are large, super-spreader-like events that have occurred throughout the COVID-19 pandemic, and our current model is unable to fully capture these dynamics. Another important assumption to note is that of the relationship between pneumonia seasonality and SARS-CoV-2 seasonality. To date, across both the Northern and Southern Hemispheres, there is a strong association between COVID-19 cases and deaths and general seasonal patterns of pneumonia deaths (Supplementary Information). Our forecasts to the end of February 2021 are immensely influenced by the assumption that this relationship will maintain throughout the year and that SARS-CoV-2 seasonality will be well approximated by pneumonia seasonality. While we assess this assumption to the extent possible (Supplementary Information), we have not yet experienced a full year of SARS-CoV-2 transmission, and as such cannot yet know if this assumption is valid. Additionally, our model attempts to account for some of the associated uncertainties in the process but does not fully capture all levels of uncertainty. Future iterations should track uncertainties that arise from more complex processes such as demographic stochasticity. There is also uncertainty (and unidentifiability) surrounding a number of the parameters of the transmission model. Here we have chosen to incorporate this lack of knowledge by drawing key transmission parameters from plausible distributions and then presenting the average result across these potential realities. As more information becomes available, we hope to tune these parameters to each location in turn.

Finally, the model presented herein is not the first model our team has developed to predict current and future transmission of SARS-CoV-2. As the outbreak has progressed, we have attempted to adapt our modeling framework to both the changing epidemiological landscape, as well as the increase in data that could be useful to inform a model. Changes in the dynamics of the outbreak overwhelmed both the initial purpose and some key assumptions of our first model, requiring evolution in our approach. While the current SEIR formulation is a more flexible framework (and thus less likely to need complete reconfiguration as the outbreak progresses further), we fully expect the need to adapt our model to accommodate future shifts in patterns of SARS-CoV-2 transmission. Incorporating movement within and without locations is one example, but resolving our model at finer spatial scales, as well as accounting for differential exposure and treatment rates across sexes and races are other dimensions of transmission modeling that we currently do not account for but expect will be necessary additions in the coming months. As we have done before, we will continually adapt, update and improve our model based on need and predictive validity.

### Reporting Summary

Further information on research design is available in the Nature Research Reporting Summary linked to this article.

## Online content

Any methods, additional references, Nature Research reporting summaries, source data, extended data, supplementary information, acknowledgements, peer review information; details of author contributions and competing interests; and statements of data and code availability are available at 10.1038/s41591-020-1132-9.

## Supplementary information

Supplementary Information Supplementary Text on data and methods, Supplementary Model descriptions, Supplementary Figs. 1–12 and Supplementary Tables 1–12.

Reporting Summary

Supplementary Data 1 Appendix 1: SEIR model vetting plots for the scenario of 95% mask use with mandates.

Supplementary Data 2 Appendix 2: SEIR model vetting plots for the scenario of 95% mask use without mandates.

Supplementary Data 3 Appendix 3: SEIR model vetting plots for the scenario of 85% mask use with mandates.

Supplementary Data 4 Appendix 4: detailed SEIR regression diagnostics.

Supplementary Data 5 Appendix 5: spatial distribution of selected covariates.

## Data Availability

Results specific to the model run for this publication are accessible for each state (http://ghdx.healthdata.org/record/ihme-data/united-states-covid-19-scenarios-2020-2021). The estimates viewable in our online tool (https://covid19.healthdata.org/) will be iteratively updated as new data are incorporated and will ultimately supersede the results in this paper. The findings of this study are supported by data available in public online repositories and data that are available upon request from the data provider; non-publicly available data were used under license for the current study but can be made available with permission of the data provider; contact information is provided where applicable. Data citations for COVID-19 metrics (cases, hospitalizations and deaths) include the COVID-19 Repository by the Center for Systems Science and Engineering at Johns Hopkins University (cases and deaths; https://github.com/CSSEGISandData/COVID-19) and the COVID Tracking Project (hospitalizations; https://covidtracking.com/data/api). State-level datasets were replaced in the following locations, using the following sources: Alaska hospitalizations from https://coronavirus-response-alaska-dhss.hub.arcgis.com/; Delaware cases and deaths from https://www.dhss.delaware.gov/dhss/dph/index.html; Hawaii cases and deaths from https://health.hawaii.gov/coronavirusdisease2019/what-you-should-know/current-situation-in-hawaii/; Illinois cases and deaths from https://dph.illinois.gov/covid19/covid19-statistics; Indiana cases and deaths from https://www.coronavirus.in.gov/2393.htm; Kentucky cases and deaths from https://govstatus.egov.com/kycovid19; Maryland cases and deaths from https://coronavirus.maryland.gov/; Nebraska cases and deaths from http://dhhs.ne.gov/Pages/Coronavirus.aspx; New York cases and deaths from https://github.com/nychealth/coronavirus-data and https://covid19tracker.health.ny.gov/views/NYS-COVID19-Tracker/NYSDOHCOVID-19Tracker-Map?%3Aembed=yes&%3Atoolbar=no&%3Atabs=n; North Carolina cases and deaths from https://covid19.ncdhhs.gov/dashboard; and Washington cases, hospitalizations and deaths from https://www.doh.wa.gov/Emergencies/COVID19/DataDashboard. The timing of mandate implementation for each state was derived from a preprint study^43^ and supplemented with ad hoc additional resources available at http://ghdx.healthdata.org/record/ihme-data/united-states-covid-19-scenarios-2020-2021. The mobility covariate was constructed using data from Google Community Mobility Reports (https://www.google.com/covid19/mobility/); Facebook Data for Good Disease Prevention Maps (https://dataforgood.fb.com/tools/disease-prevention-maps/; with access coordinated via diseaseprevmaps@fb.com); SafeGraph Shelter in Place Index (https://www.safegraph.com/dashboard/covid19-shelter-in-place?s=US&d=09-13-2020&t=counties&m=index; with access coordinated through the SafeGraph COVID-19 Data Consortium via https://www.safegraph.com/covid-19-data-consortium/); and Descartes Labs (https://github.com/descarteslabs/DL-COVID-19). The testing covariate was constructed using data from the COVID Tracking Project (https://covidtracking.com/data/api/). State-level datasets for the testing covariate were replaced in Washington, using https://www.doh.wa.gov/Emergencies/COVID19/DataDashboard. Mask-use data were obtained from Premise COVID-19 Global Impact Survey (https://www.premise.com/the-dos-and-donts-of-conducting-surveys-during-covid-19/; with access coordinated through info@premise.com); the Facebook (COVID) Symptom Survey (with access coordinated through University of Maryland Joint Program in Survey Methodology via admin-C19survey-fb@umd.edu); and the YouGov COVID-19 Behavioural Tracker Survey (https://github.com/YouGov-Data/covid-19-tracker). Pneumonia seasonality estimates, particulate matter air pollution estimates, lower respiratory infection country-specific mortality rate estimates and smoking estimates were generated by the Global Burden of Disease study (http://ghdx.healthdata.org/record/ihme-data/united-states-covid-19-scenarios-2020-2021/). Altitude was sourced from the National Oceanic and Atmospheric Administration National Centers for Environmental Information Global Land One-km Base Elevation Project (https://www.ngdc.noaa.gov/mgg/topo/globe.html) and population data were obtained from WorldPop Population Counts (https://www.worldpop.org/project/list/). These sources are further detailed in the Supplementary Information^44–51^. Source data are provided with this paper.

## Source data

Source Data Fig. 1 Statistical source data. Source Data Fig. 2 Statistical source data. Source Data Fig. 3 Statistical source data. Source Data Fig. 4 Statistical source data. Source Data Extended Data Fig. 1 Statistical source data. Source Data Extended Data Fig. 2 Statistical source data. Source Data Extended Data Fig. 3 Statistical source data. Source Data Extended Data Fig. 4 Statistical source data. Source Data Extended Data Fig. 5 Statistical source data. Source Data Extended Data Fig. 6 Statistical source data. Source Data Extended Data Fig. 7 Statistical source data. Source Data Extended Data Fig. 8 Statistical source data. Source Data Extended Data Fig. 9 Statistical source data.

## Code availability

All code used for these analyses was custom created for this study and is publicly available online (https://github.com/ihmeuw/covid-model-seiir-pipeline/ and https://github.com/ihmeuw/covid-model-deaths-spline/).

Analyses were carried out using R version 3.6.1, Python 3.8 and R-INLA version 20.01.29.9000. All maps presented in this study are generated by the authors using RStudio (R Version 3.6.3) and ArcGIS Desktop 10.6, and no permissions were required to publish them. Administrative boundaries were retrieved from the Database of Global Administrative Areas. Land cover was retrieved from the online Data Pool, courtesy of the NASA Earth Observing System Data and Information System Land Processes Distributed Active Archive Center, United States Geological Survey Earth Resources Observation and Science Center.
