# Supplementary material for: Modeling COVID-19 scenarios for the United States
Source: Nat Med. 2020 Oct 23;27(1):94–105. doi: 10.1038/s41591-020-1132-9 (PMC7806509; doi:10.1038/s41591-020-1132-9)
Supplement: Supplementary file 1 — Supplementary Text on data and methods, Supplementary Model descriptions, Supplementary Figs. 1–12 and Supplementary Tables 1–12. [file 41591_2020_1132_MOESM1_ESM.pdf]

nature research

https://doi.org/10.1038/s41591-020-1132-9

Supplementary information

Modeling COVID-19 scenarios for the United States

In the format provided by the authors and unedited

Supplementary Information: COVID-19 scenarios for the United States

1 Table of Contents

1 Table of Contents ... *2*

Guidelines for Accurate and Transparent Health Estimates Reporting (GATHER) ... *6*

2 Methods overview ... *6*

3 Death, case, and hospital quantities ... *7*

3.1 Basic COVID-19 epidemiological data ... **7**

3.2 Detailed COVID-19 epidemiological data ... **8**

3.3 Data preparation ... **8**

3.4 COVID-19 hospital utilisation data ... **10**

3.5 Modelling past deaths using random knot combination splines (RKCS) ... **11**

3.5.1 Data and model overview ... 11
3.5.2 Deaths as a function of reported cases and hospitalisations ... 11
3.5.3 Fitting final deaths curve with uncertainty using all epidemiological data inputs ... 11
3.5.4 Day-of-week ensemble ... 12

3.6 Estimating infections from deaths ... **12**

4 COVID-19 covariates ... *13*

4.1 Social distancing mandates ... **14**

4.1.1 Data processing ... 14
4.1.2 Use in SEIR-fit ... 15
4.1.3 Forecasting mandates ... 15

4.2 Mobility ... **15**

4.2.1 Data processing ... 15
4.2.2 Use in SEIR-fit ... 17
4.2.3 Forecasting mobility ... 18

4.3 Testing *per capita* ... **18**

4.3.1 Data processing ... 18
4.3.2 Use in SEIR-fit ... 18
4.3.3 Forecasting testing ... 18

4.4 Mask effectiveness and use ... **19**

4.4.1 Data processing ... 19
4.4.2 Use in SEIR-fit ... 20
4.4.3 Forecasting mask use ... 20
4.4.4 Estimation of mask use effect size ... 20

4.5 Pneumonia seasonality ... **21**

4.6 Time-invariant covariates ... **22**

4.6.1 Lower Respiratory Infection Mortality ... 22
4.6.2 Altitude ... 22
4.6.3 Smoking ... 22

4.6.4 Ambient particulate matter pollution 22
4.6.5 Population density 23
4.6.6 Demography 23
4.6.7 Altitude 23

5 Intermediate quantity modelling 23

5.1 Mortality rate by age estimation 23

5.2 Infection fatality ratio 23

5.3 Infection to death duration 24

5.4 Hospitalisations to death ratio 24

6 Fitting and predicting transmission dynamics 24

6.1 SEIR-fit 24

6.1.1 Model formulation 24
6.1.2 Basic reproductive number under control and the effective reproductive number 25
6.1.3 Fitting $\beta(t)$ 26

6.2 β regression 27

6.2.1 Overview 27
6.2.2 Fitted regression coefficients 27
6.2.3 Regression diagnostics 28

6.3 β adjustments 28

6.4 SEIR-predict 29

6.5 State-specific β regression results 30

6.5.1 Exemplar 1: California 30
6.5.2 Exemplar 2: New York 31
6.5.3 Exemplar 3: Texas 32

7 Final data combination and summarization 33

7.1 Scenarios 33

8 Hospital resource use microsimulation 34

9 Sensitivity analyses 35

9.1 OOS predictive validity analyses 35

9.1.1 8-week OOS analyses 36
9.1.1.1 Analyses 1 36
9.1.1.2 Analyses 2 36
9.1.2 4-week OOS analyses 37
9.1.2.1 Analyses 1 37
9.1.2.2 Analyses 2 37
9.1.3 Mobility and the changing transmission landscape 38

9.2 Assessment of future predictions 38

9.2.1 Analyses 1 38

9.2.2 Analyses 2 39

Supplementary Figures 41

SI Figure 1. Schematic representation of modelling process. Numbers correspond to Supplementary Information sections detailing each portion. 41

SI Figure 2 Time trends and relationship with deaths for cumulative cases and hospitalisations in Florida. 42

SI Figure 3 Estimated death curve in cumulative and ln(daily) in Florida. Samples from refit model are shown in ln(daily) plot. 43

SI Figure 4. Trends of the number of mandates (out of 6) on for each location in the modelling hierarchy 44

SI Figure 5. Time trends of the average number of mandates "on" for each region of the world. 45

SI Figure 6. MR-BRT model of the US pattern of seasonality of logit ratio vs week. 46

SI Figure 7. Eight week out-of-sample predictive validity (July 4 model run) 47

SI Figure 8. Eight-week out-of-sample predictive validity (August 21 model run) 48

SI Figure 9. Four-week out-of-sample predictive validity (July 4 model run) 49

SI Figure 10. Four-week out-of-sample predictive validity (August 21 model run) 50

SI Figure 11. Fitted coefficient on mobility for the 8-week and 4-week out-of-sample and full "production run" versions of the model (July 4 model run) 51

SI Figure 12. Fitted coefficient on mobility for the 8-week and 4-week out-of-sample and full "production run" versions of the model (August 21 model run) 52

Supplementary Tables 53

SI Table 1. GATHER compliance 53

SI Table 2. Sources for epidemiological data by state 56

SI Table 3. Alternate data repositories for state-level reporting 59

SI Table 4. Ad hoc corrections made to the Johns Hopkins dataset 60

SI Table 5. Data sources by state for COVID-19-specific hospitalisations 61

SI Table 6. Covariates summary table 62

SI Table 7. Date of enactment, repeal, and source by state for social distancing mandates 63

SI Table 8. Listing of source of testing data for locations not present within Our World in Data global dataset 64

SI Table 9. Infection fatality ratio data sources 69

SI Table 10. Mortality rate estimation data sources 73

SI Table 11. β regression coefficient constraints 86

SI Table 12 Cumulative deaths 21 September 2020 through 28 February 2021, maximum estimated daily deaths per million population, date of maximum daily deaths, and estimated $R_{effective}$ on 28 February 2021 for two additional derivative scenarios. 87

Guidelines for Accurate and Transparent Health Estimates Reporting (GATHER)

Please see Supplemental Information [SI] Table 1 for details on how this study meets the Guidelines for Accurate and Transparent Health Estimates Reporting (GATHER).

2 Methods overview

Supplementary Information [SI] Figure 1 presents a schematic representation of the modelling process. The estimation of past and current deaths blends data on reported COVID-19 deaths, COVID-19 hospitalisations, and COVID-19 cases and testing rates; there are various biases (described in SI section 2) associated with reported COVID-19 deaths that must be overcome before the next step of the modelling process. By using COVID-19 cases and testing rates (and hospitalisation data where available) as leading indicators of deaths, we can extend our estimates of deaths beyond that of the available death data (in particular, by up to eight days past the last death data point; details are given in SI Section 2.5). From estimates of past and current daily deaths, we calculate past and current daily new SARS-CoV-2 infections using age-specific mortality rates, age-specific infection fatality rates, and estimates of the average time from infection to death (details are given in SI Section 4.3).

The primary model for estimating future infections and deaths is a mechanistic compartmental model. Specifically, the fraction of each location's population that is susceptible (S), infected but not infectious (exposed, E), infectious (I_1, I_2), and recovered (R), forming an SEIR model. Temporal variations in past transmission intensity is captured through the time-varying parameter $\beta(t)$ (details are given in SI Section 5). The association between the time-varying transmission intensity and a number of covariates is assessed in a multivariate mixed effects regression across all locations simultaneously (details are given in SI Section 5). Each of the covariates is then forecast into the future, with certain covariates forecast multiple times corresponding to unique future scenarios (details are given in SI Sections 5, 6). The forecast covariate values and the fitted regression model are then used to estimate future transmission intensity; the future transmission intensity is then used in the SEIR framework to estimate future infections. Finally, reversing the process that estimated past infections from past deaths, future deaths are estimated from future infections (details are given in SI Section 5.4).

The final component of the modelling approach uses past, current, and future infections and deaths to estimate hospitalisations, including estimates of ICU usage and invasive ventilation need (details are given in SI Section 7).

The estimation of past and current deaths model produces uncertainty. From this uncertainty, we generate 1,000 draws of past and current deaths for each location. The remaining steps of the process described above in brief and below in detail are done by draw, accumulating uncertainty in the subsequent steps (e.g., a separate regression connecting location-specific time-varying transmission intensity to covariates is conducted for each draw).

3 Death, case, and hospital quantities

Our sources of COVID-19 data come from a wide range of both governmental, non-profit, and volunteer organisations. In all instances, we aim to best reflect what information is being reported by each location with respect to the various COVID-19 measures. Given the various data requirements for the model, we collated the following information:

- Basic COVID-19 epidemiological data (cases and deaths by location and date)
- COVID-19 hospital utilisation data (cumulative hospitalisations or admissions data)
- Detailed COVID-19 epidemiological data (age and sex stratified data, time interval between symptom onset and clinical outcome, length of stay in hospital etc.)
- Covariate data (discussed in SI Section 3), including testing rates or data describing behaviours relevant to COVID-19 transmission (e.g. mask use and general mobility)

Such data collection processes naturally reflect the messy nature of daily data collection and processing – throughout the pandemic we have seen data systems fail and days of non-report that can lead to misleading artefacts in time series that hinder modelling. Where feasible to track (such as state-level cases and deaths) we track multiple sources of data which either allows us to replace erroneous data should one system fail or identify artefacts in common and seek out a specific resolution.

All data sources are described in more detail below, as well as specific fixes and corrections required for each data type.

3.1 Basic COVID-19 epidemiological data

Sources for the epidemiological data used in our model are listed by state in SI Table 2. As a first pass, given their global data collection efforts, we used the Johns Hopkins University CSSE data collection system, which uses a variety of primarily web-scraping and text parsing approaches to periodically capture reported case and death numbers. Across the pandemic, we have seen many times when data reporting mechanisms have either broken down or have been paused (e.g. for weekends) and consequently induce artefacts in daily case and death time series. For the US states and territories, we supplement JHU routine collection in two ways (a) using *The COVID-19 Tracking Project* (www.covidtracking.com) archive of historical data that captures screenshots of state COVID-19 dashboards several times throughout the day, allowing more flexibility in those locations where data updates were delayed and (b) manual extraction and verification of state dashboards and tracking of press-releases and footnotes of known issues and days off.

For some states, due to repeated inconsistencies between state reports and JHU time series, we have completely replaced the automated time series with a human curated alternative, supported by a library of screen captures and downloaded epidemiological bulletins and summaries (SI Table 3). Where there are only intermittent discrepancies or regular known artefacts (e.g. Oregon not reporting on weekends starting late May/early June), we have a separate mechanism that replaces erroneous values (SI Table 4). General sources for these data are listed by state in SI Table 2.

3.2 Detailed COVID-19 epidemiological data

From a number of locations, far more than just total cases and deaths are reported, allowing us to inform a variety of key parameters with data collated from across the world. Not all locations report every piece of data however, so these data tend to be fragmented in space and time.

We currently source data stratified by age from 40 locations. Where feasible, this dataset continues to be updated so it most accurately reflects the current state of affairs. Supplemental Information Section 4 describes how these available data are subset and used in the relevant analysis for both mortality rates and infection-fatality rates. In addition, we track duration and length of stay data to inform hospital utilisation statistics. To inform this, we use a mix of reported summary statistics, as well as survival analysis of individual line list data. In short, we use the Global Line List (https://github.com/beoutbreakprepared/nCoV2019) together with publicly available, de-identified individual patient data from Ohio State, USA; Mexico; Ceara State and Rio de Janeiro State, Brazil, to estimate the distribution of days from onset of symptoms to death from COVID-19.

3.3 Data preparation

While global compilers of data on cases and deaths expedite collection of data across multiple countries and locations, for a variety of reasons these more-automated compilers can be incorrect. Similarly, even where these aggregators are faithfully documenting what is reported, local issues (such as laboratories not releasing information to state officials in a timely way) introduce a variety of artefacts into the data that have no epidemiological relevance, but reflect issues in the data generation and reporting process instead. Wherever possible, we adjust for these issues to better reflect the state-of-the-art knowledge of the epidemiological situation. Where an anomaly is identified, we cross-reference with state Department of Health dashboards, or other data aggregators (such as *The COVID Tracking Projec*t; www.covidtracking.com) to identify the source of the discrepancy. News reports and press releases are consulted when, rather than a data collection error, a reporting issue is noted, or the date when probable cases and deaths were first added to the official tally introducing a large spike in daily deaths and cases.

For some locations, due to repeated inconsistencies between Johns Hopkins data and state-level reporting, we have manually undertaken our own extraction, or sourced an alternate repository of data (SI Table 3).

Ad-hoc corrections made to the Johns Hopkins dataset are described in SI Table 4 Where artefacts are identified, the indicated cases and deaths are redistributed in the preceding time period proportionate to the daily patterning of cases and deaths. The following redistribution steps took place:

- Alabama – 11th July, 3627 hospitalizations – Change in hospital data processing;
- Alabama – 24th September, 195 cases – Backlog of test results from a laboratory;
- Alabama – 25th September, 1594 cases – Backlog of test results from a laboratory;
- Alaska – 25th August, 2 deaths – Two deaths added from prior time periods;
- Arizona – 17th September, 577 cases – Inclusion of those determined positive via antigen testing;
- Arizona – 18th September, 764 cases – Inclusion of those determined positive via antigen testing;
- Arkansas – 16th September, 139 deaths – Addition of probable deaths;
- Connecticut – 24th July, 440 cases – Testing backlog received from out-of-state lab;
- Delaware – 23rd June, 67 deaths – Deaths from a prior period were reported on this day;

- Delaware – 24th July, 49 deaths – Reporting lags;
- Florida – 1st September, 4620 cases – Backlog of test results from Quest Diagnostics
- Hawaii – 18th September, 12 deaths – Addition of deaths from an elder care facility outbreak
- Illinois – 4th September, 3558 cases – Backlog in timely reporting;
- Iowa – 27th August, 1450 cases – Addition of cases tested positive from antigen tests;
- Kentucky – 24th July, 360 hospitalizations – Anomalous reporting; assumed to be due to reporting lags;
- Kentucky – 1st August, 463 hospitalizations – Anomalous reporting; assumed to be due to reporting lags;
- Louisiana – 21st May, 682 cases – Louisiana reported a backlog of positive tests;
- Louisiana – 9th September, 690 cases – Backlog of cases reported dating from 6th August to 4th September;
- Maryland – 14th April, 64 cases – First day of reported probable cases;
- Massachusetts – 1st June, 3,514 cases - Massachusetts added probable deaths and cases on 1st June;
- Massachusetts – 1st June, 141 deaths - Massachusetts added probable deaths and cases on 1st June;
- Massachusetts – 2nd June, 110 cases – Massachusetts continued to add probable cases;
- Michigan – 5th June, 5014 cases – Michigan started reporting probable cases;
- Michigan – 5th June, 239 deaths – Michigan started reporting probable cases;
- Michigan – 9th September, 63 deaths – Batch of probable deaths reporting on this day;
- Missouri – 5th September, 56 deaths – Deaths reported in this period actually occurred between June and August
- Missouri – 23rd September, 64 deaths – Missouri provided updated timelines for deaths reported in prior time periods;
- Missouri – 26th September, 20 deaths – Missouri provided updated timelines for deaths reported in prior time periods;
- Montana – 24th September, 40 hospitalizations – Anomalous reporting; assumed to be due to reporting lags;
- Nebraska – 12th August, 127 hospitalizations – Anomalous reporting; assumed to be due to reporting lags;
- New Hampshire – 15th July, 74 hospitalizations – Anomalous reporting; assumed to be due to reporting lags;
- New Jersey – 25th June, 1,854 deaths – Probable deaths included for the first time;
- New Jersey – 8th July, 91 deaths – increase in probable deaths due to reporting lags;
- Ohio – 15th September, 67 deaths – Backlog of deaths reported on this day;
- South Carolina – 16th July, 52 deaths – Reporting lags;
- South Carolina – 11th September, 1487 cases – USC delayed in uploading results spanning 22nd August to 8th September;
- South Carolina – 13th September, 1364 cases – Continued anomalous reporting due to college reporting backlogs;
- Virginia – 15th September, 88 deaths - Anomalous reporting; assumed to be due to reporting lags;
- Virginia – 16th September, 36 deaths - Anomalous reporting; assumed to be due to reporting lags;

- Virginia – 17th September, 27 deaths - Anomalous reporting; assumed to be due to reporting lags;
- Virginia – 18th September, 20 deaths - Anomalous reporting; assumed to be due to reporting lags;
- Virginia – 19th September, 32 deaths - Anomalous reporting; assumed to be due to reporting lags;
- Virginia – 20th September, 16 deaths - Anomalous reporting; assumed to be due to reporting lags;
- Wyoming – 9th April, 73 cases – Day Wyoming first reported probable cases.

For the following locations, we removed the total associated number of cases or deaths from the preceding time period proportionate to the daily patterning of cases and deaths:

- Arkansas – 14th August, 1251 cases – Decrease due to removal of out of state resident cases
- Delaware – 24th July, 151 cases – Case series revised;
- Indiana – 20th August, 81 hospitalizations – Unexplained decrease; retained 7-day daily average and removed rest
- Louisiana – 18th June 1,666 cases – 1,666 cases were identified as duplicates and total was revised;
- Maine – 2nd June, 7 hospitalizations – Unexplained decrease, retained 7-day daily average and removed rest;
- Massachusetts – 1st September, 106 hospitalizations – Unexplained decrease, retained 7-day daily average and removed rest;
- Massachusetts – 2nd September, 7983 cases - Unexplained decrease; retained 7-day daily average and removed rest;
- Nebraska – 18th August, 48 hospitalizations – Unexplained decrease; retained 7-day daily average and removed rest;
- North Dakota – 25th May, 82 cases – 82 positive results were considered inconclusive and asked to be re-tested due to a lab experiencing a recent malfunction on two pieces of lab equipment.
- Oregon– 3rd September, 22 hospitalizations – Unexplained decrease; retained 7-day daily average and removed rest;
- Rhode Island – 24th September, 208 cases – Removal of duplicated cases from case count;
- Wyoming – 5th September, 4 hospitalizations – Unexplained decrease; retained 7-day daily average and removed rest;

3.4 COVID-19 hospital utilisation data

Our model also estimates numbers of individuals in hospital and in intensive care. Hospitalisation data therefore gets used in two ways: (i) as a leading predictor of daily deaths (ii) as a statistic used to define the number of hospitalisations that result in deaths.

Data for these metrics were collected from the respective state departments of health and associated dashboards. It is important to note that hospitalisation data is typically reported in one of three formats: (i) the cumulative total of all hospitalisations to date (ii) the daily admission of newly hospitalised patients (which if a series is inclusive of the first day of admissions, a cumulative total can be recapitulated) and (iii) as a census statistic that reports the number of individuals currently in-hospital.

Our current modelling framework takes advantage of data reported in formats (i) and (ii). Sources for these data are listed by state in SI Table 5.

3.5 Modelling past deaths using random knot combination splines (RKCS)

3.5.1 Data and model overview

To derive infections from deaths and the infection fatality rate (SI Section 2.6) for use in the transmission model, we first perform a series of spline regressions using IHME's customised meta-regression tool MR-BRT. MR-BRT ("meta-regression—Bayesian, regularised, trimmed") is a trimmed constrained mixed-effects model that provides an easy interface for formulating and solving common linear and nonlinear mixed effects models. It is open source, and its core computational kernel uses the mixed effects package LimeTr (https://github.com/zhengp0/limetr) and the spline package XSpline (https://github.com/zhengp0/xspline). For the statistical models and algorithmic features underlying MR-BRT, a published technical report is available[37].

We use MR-BRT functionality that allows the user to specify a number of potential knot combinations to be randomly generated and runs separate models for each combination, which are then evaluated for performance and combined using those scores to create a weighted composite of the sub-models. We use 40 combinations in each of the subsequently described model stages, which are run separately by location.

The estimates obtained from MR-BRT smooth the trend in reported deaths and leverage patterns in reported case and hospital admissions data where available to make short term forecasts of deaths. Deaths and cases by day were available for every location; hospital admissions data were also available for 35 states. Before merging with deaths for modelling we account for the lag between hospital admissions or reporting of cases and death based on the Global Line List (https://github.com/beoutbreakprepared/nCoV2019) by shifting dates for these measures forward in time eight days.

3.5.2 Deaths as a function of reported cases and hospitalisations

In the first stage we model the cumulative death rate with either the cumulative case rate or the cumulative hospital admission rate as independent variable. Where data for both of these variables are available, a separate model is run for each. We use a cubic spline with one knot per 12 data points, but with the rightmost interval forced to be linear rather than cubic. We also fix the rightmost interior knot such that the right segment contains four data points, and we constrain the curve such that cumulative deaths monotonically increase along with cumulative cases/hospitalisations. Because of the shift window, we have eight days of case and hospitalisation data that extend past the last day of death data used to fit the model – by linearly extrapolating the tail of the fitted curve we produce projections of deaths that correspond to the additional eight days of case or hospitalisation data, in addition to our in-sample fit. These death estimates capture the trend in cases or hospitalisations while effectively accounting for changing case- and hospitalisation-fatality ratios due to variation in exogenous factors such as age, pattern of cases, and testing rates. SI Figure 2 illustrates the fit for Florida, showing the time series of cumulative cases and hospitalisations, as well as the fit to deaths.

3.5.3 Fitting final deaths curve with uncertainty using all epidemiological data inputs

Using deaths estimated as a function of cases and hospitalisations from the model described above, in addition to observed deaths, we then fit a second stage model using cumulative deaths from all three

sources with time (in days) as the independent variable. We inflate the standard error of the first stage death estimates by a factor of two so that they are not as influential as the observed deaths. Once again, we use a cubic spline with a linear right tail, and a constraint to be monotonically increasing over time. We also fix the rightmost interior knot in such a manner that the linear rightmost segment contains four days of reported deaths – and thus 12 days of estimated deaths from cases and hospitalisations.

With the resultant curve, we calculate the robust standard deviation of residuals in log daily death space which we use to independently sample death rates by day, resulting in uncorrelated time series draws representative of the observed noise in the data. We refit models to each of these log daily deaths time series, giving us smooth estimates of death with uncertainty for the full range of dates with observed deaths and extending out to an 8-day projection. We use the same knots samples as the cumulative model, once again with a linear right tail. In this model, we add Gaussian priors on the 3rd derivative of the cubic segments – a stronger prior of $\mathcal{N}(0,\ 10^{-4})$ on the left-tail segment, and a "dampening" prior of $\mathcal{N}(0, 0.01)$ on the remaining interior segments. The first of these permits non-linear growth early on in the outbreak while controlling for erratic behaviour in cubic splines at the terminus, and the second serves to reduce volatility that would suggest implausible fluctuations in transmission in the downstream model. Additionally, if fewer than 21 deaths have occurred in the past week we include a strong prior $\mathcal{N}(0,\ 10^{-8})$ on the slope of the rightmost segment, forcing it to be flatter. This mitigates the phenomenon of subtle changes of the linear death rate trend in settings with small numbers of deaths being projected as exponential growth in the non-linear transmission model. SI Figure 3 shows the cumulative point estimate and ln(daily) samples of death curves for Florida.

3.5.4 Day-of-week ensemble

In addition to stochasticity in the day-to-day reporting of these indicators, there is also bias that can be traced to the day of week on which the report falls – in general, Sunday and Monday tend to be underreported, with compensating overreporting Tuesday through Saturday. While this is generally true, the day of week pattern varies by state. This means that a model run on data reported on a Monday can tend to over-emphasize or create the illusion of declining trends, while the opposite can be true of models run on Saturday-reported data. To address this we run seven models, each using data up to the most recent reporting for a given day of the week – so, for results based on data reported on Monday, 28 September, we run a model using data up through Tuesday, 22 September, and additional models for each day up through 28 September. The predictions of the linear right tail for each model extend to the most recent day predicted in the final model, 6 September. We use 142 samples from each of the past days models and 148 from the most recent day, resulting in 1000 draws for each location.

3.6 Estimating infections from deaths

Conditioning on the death draws produced in SI Section 2.5 and the Infection Fatality Rate (IFR) and age-specific mortality rate (MR) calculated in SI Sections 4.2 and 4.1, daily infections are inferred by stratifying all-age deaths into age-specific deaths, using the age-specific IFR to determine the number of infections that would have led to this quantity of age-deaths, and then backshifting the infections in time to account for the lag between infection and deaths.

For each of the $j \in 1, \ldots, 1000$ cumulative death draws time-series, CD^j, one infection-to-death lag, l^j is randomly sampled from a discrete uniform distribution on 17 to 21 days.

For each lowest-level location, loc:

1. Daily deaths time-series, $DD^j(loc)$, are generated by differencing the cumulative deaths time-series, $CD^j(loc)$.
2. The mortality probabilities, $MP_{ageBin_i}(loc)$, for an individual in this location belonging to each 5-year age bins, $ageBin_i$, is calculated:

$$MP_{ageBin_i}(loc) = \frac{MR_{ageBin_i}(loc) \times Pop_{ageB\ \ i}(loc)}{\Sigma_i(MR_{ageB\ \ i}(loc) \times Pop_{ageBi\ \ i}(loc)},$$

where $Pop_{ageBi\ \ i}(loc)$, is the total population for that $ageBin_i$ at loc. If this is not available, we resort to using the parent location's population.

3. The expected age-specific daily deaths time-series, $DD^j_{ageBi\ \ i}(loc)$, is calculated by stratifying the all-age deaths using the age-specific mortality probabilities, $MP_{ageBi\ \ i}(loc)$:

$$DD^j_{ageBin_i}(loc) = MP_{ageBi\ \ i}(loc) \times DD^j(loc).$$

4. The expected age-specific daily infections time-series, $DI^j_{ageB\ \ i}(loc)$, are calculated from the age-specific IFR and daily deaths:

$$DI^j_{ageB\ \ i}(loc) = DD^j_{ageB\ \ i}(loc)/IFR_{ageB\ \ i}(loc)$$

5. The date of the infection time-series is taken to be the date of the death time series shifted back by l^j days.
6. The all-age daily infection time-series is prepared for the SEIR model by summing the infections across all age groups:

$$DI^j(loc) = \Sigma_i DI^j_{ageBi\ \ i}(loc).$$

This process yields 1000 draws of daily new infections across all modelled locations.

$$MR_{ageB\ \ i}(loc)/\Sigma_i MR_{ageB\ \ i}(loc) A_D H: D_{A_D}$$

4 COVID-19 covariates

Covariates for the compartmental transmission SEIR model are predictors of the β parameter in the model that affect the transition from Susceptible to Infected states. Covariates were evaluated on the basis of biologic plausibility and on the impact on the results of the SEIR model. Given limited empirical evidence of population-level predictors of SARS-CoV-2 transmission, biologically plausible predictors of pneumonia such as population density (percentage of the population living in areas with more than 1000 individuals per square kilometer), tobacco smoking prevalence, population-weighted elevation, lower respiratory infection mortality rate, and particulate matter air pollution were considered. These

covariates are representative at a population-level and are time invariant. Spatially resolved estimates for these covariates are derived from the Global Burden of Disease Study 2019 (http://ghdx.healthdata.org/record/ihme-data/gbd-2015-covariates-1980-2015). Time varying covariates include seasonality of pneumonia excess mortality, diagnostic tests per capita, population-level mobility, and personal mask use. These are described in the following sections and summarised in SI Table 6.

4.1 Social distancing mandates

A wide array of social distancing mandates have been implemented across the 50 states, some presenting as recommendations, others as requirements, some presenting fragmented updates that escalate over a few days or weeks, others as discrete events where a state transitions from no measures to full implementation of strict social distancing measures. To allow for comparability across different geographies, we collected and collated these mandates focusing on four components of social distancing, with six tiers of implementation. We only included those orders that were direct restrictions and had a legal basis for enforcement; executive orders that were only “recommendations” or that “urged” or “encouraged” citizens were therefore excluded. In all instances, we were interested in documenting the date of enactment, not the date of proclamation.

4.1.1 Data processing

Following New Zealand’s Alert Level system we identified four key sectors: stay-at-home measures intended to restrict the number of direct contacts any individuals may have, business and workplace closures intended to minimise transmission among employees and with customers, educational closures intended to protect students and staff, and internal travel restrictions intended to limit the amount of non-essential movement taken by individuals. For stay-at-home measures we considered two strata: the date at which any restrictions on the gathering of people took place, and the date at which a full stay-at-home order was mandated, with interactions between households restricted. For business closures we considered two strata: the date at which the first restrictions applied to businesses were enacted, and the date on which all non-essential businesses were mandated to close. “Non-essential” is an inherently local distinction – rather than provide an exhaustive list of businesses that must have been closed to qualify, we followed local guidance. The necessary component, however, was clear exhaustive local guidance as to what businesses were essential, with an emphasis that all other businesses are non-essential and therefore closed.

In the last few months we have seen the de-escalation of these social distancing measures and have tracked the dates on which prior restrictions have been repealed. Additionally, some states are re-imposing social distancing measures in recent weeks, which we also track and incorporate into the model. We identified legislation that was the antithesis of the closure orders that proceeded them. Consequently, should an executive order requiring people to stay at home be relaxed so that different households could interact, or that individuals could leave home for non-essential reasons, these orders would be associated with the date of relaxation. For an executive order to be repealed, it must be repealed across the entirety of the population affected – states that were following a phased process that varied county by county were only considered to have repealed the strictest mandates once all counties had the social distancing measures relaxed.

We used two key approaches for populating the US mandate database – (i) cross-referencing the resource compiled by the University of Washington Political Sciences Department tracking state-level executive orders[43] and (ii) supplementation of these efforts by direct searches of state legislature websites, and governor websites. Supplemental Information Table 7 provides the date of enactment and repeal by state for each the six tracked measures, as well as linking to the source used to verify (see also SI Figure 4 and SI Figure 5). Global mandates were tracked via a combination of using the World Health Organization's Public Health Social Mandates database, supplemented by specific local searches of government websites and news resources.

4.1.2 Use in SEIR-fit

After analyzing the time trends of mandate imposition around the world, we noticed that initial mandate imposition occurred within a two-to-three week period during March for most of the world, indicating that mandate imposition had more to do with global pressure to enact mandates and less to do with the outbreak size in a specific location.

Rather than model each mandate individually, we looked at the mandate imposition trend in aggregate across five of the six IHME mandates: stay at home order, educational facilities closed, all non-essential businesses closed, partial business closure, and any gathering restriction. Specifically, we fit a quasibinomial model (mgcv R package) on the proportion of five mandates implemented at a given time as a function of location and date. The regression has a location specific intercept and a spline on day with six knots.

$$Proportion\ of\ mandates\ implemented \sim location + s(day, k = 6)$$

4.1.3 Forecasting mandates

The probability that five mandates will be "on" during any given day declines towards zero over time. To ensure that mandate forecasts align with observed data, we multiplicatively intercept shift the forecast to the start at the most recent observed data for mandate status.

4.2 Mobility

To better understand and predict disease transmission, we estimate human movement relative to baseline movement patterns prior to the COVID-19 pandemic.

4.2.1 Data processing

These data come from mobile phone users. We used four primary resources to gauge the changes in relative mobility of populations within each state: Google Community Mobility Reports (https://www.google.com/covid19/mobility/), Facebook Data for Good (https://dataforgood.fb.com/docs/covid19/), Safegraph (https://www.safegraph.com/dashboard/covid19-shelter-in-place), and Descartes Laboratories (https://www.descarteslabs.com/mobility/). Each of these sources have different definitions of mobility. For example, the data from Google reports distance traveled to six categories of locations relative to daily values from 03 January 3 to 06 February, 2020. SafeGraph reports the percent of devices that do not leave "home" relative to a baseline period of 08 February to 14 February, 2020.

Google data are reported as a percentage difference in attendance to certain destinations compared to the median value from the 5-week period 03 January to 06 February, 2020. The reports are stratified by

six destinations: “Retail & recreation”, “Grocery and pharmacy”, “Parks”, “Transit stations”, “Workplaces”, and “Residential”. We took the average of the percentage change in the “Retail & recreation”, “Transit stations”, and “Workplaces” since these three destinations represent activities most strongly affected by the social distancing measures. No further processing is undertaken prior to modelling.

Descartes Laboratories release mobility statistics at state and county levels. They provide a mobility index (of values normalised for the weeks of 17 February to 07 March, 2020) that represents the maximum Haversine (great circle) distance from the initial starting points reported by devices. The top 10% of their data is removed due to possible inclusion of outlier data due to poor GPS recording. The index is reported from 01 March, 2020 through to three days prior to-date. The index is transformed by subtracting 100 from the m50_index value.

Safegraph data release a number of measures that allow for a stay-at-home metric to be calculated. Data is reported from January 1st through to three days prior to-date, derived from GPS reports from anonymous mobile devices. These are used to determine a nighttime location for each device over a six week period. Devices are aggregated by home census block group. For modelling we determine an index representing the percent difference between the number of devices that flagged as having not stayed within their home range as compared to the mean number of devices that stayed within their home range over a baseline reference period (08 February and 14 February, 2020). To calculate the number of devices that stay within home range, for each census block group, we determine the ratio of devices that never leave home to the total number of devices. Using the associated FIPS codes, we can aggregate to the various analysis locations (whether counties, or states, or territories) by taking the device-weighted mean of the census block group ratios.

Facebook Data for Good datasets are determined location-by-location or as geographic ranges. Facebook tracks the aggregate patterns of movement of Facebook users with location history turned on over a period of several hours. For this analysis, we receive patterns of movement reported by location-specific administrative regions, which vary based upon the geographic range of the dataset (which could span neighborhoods of a city, different cities, or districts, counties, or states). For each, a baseline period for future comparison is developed by considering the prior 45 days of Facebook user activity. Subsequent to the date of initiation, all future days of reporting cross-reference their own baseline activity period. For each dataset, we used latitude and longitude for a given location to match it to one of our modelled geographies using a spatial overlay. Where latitudes and longitudes were missing or did not accurately represent a location, we manually assigned a model geography by name. Using the start location from out modelled geographies, we find the mean percent change in mobility for all trips starting from that location on a given day and at a given time (0800, 1200, or 1600). We weight this mean by the number of users who normally take this trip (n_baseline). Given the variable baseline periods, we must transform Facebook data so that it is comparable to other sources – given the much broader geographic coverage of Google Community Mobility reports, we calculate the mean percent change in Google data for 45 days preceding the first day of Facebook data and apply this to the Facebook percentage change. Where the Facebook data starting date occurred before or at the same time as the Google data, no transformation was necessary. Where Facebook data was present after the initiation of Google’s time series, we calculated a baseline for Facebook using the mean percent change in Google data over the 45 days prior. To adjust Facebook data, we calculated the absolute value change

for the estimated Facebook baseline, added the difference between Google and Facebook values, and divided by the Google baseline. This resulted in a new mean percent change that was consistent with the baseline from Google and the other mobility datasets, rather than the Facebook dataset specific timings, some of which cross-reference a baseline period well into the lockdown period.

There are several steps to smooth and standardise the data. We observe strong patterns in mobility by the day of the week. The data from Google is already corrected for these day-of-week patterns. For all other sources we calculate a 7-day rolling mean to account for weekly trends.

4.2.2 Use in SEIR-fit

To account for differences in time coverage between sources we calculate the median ratio between each available pair of sources for each location across the time series. In locations where we are missing the time series for a given source, we impute based on all other sources and the median ratio in that location over time.

In the US, we calculate the indicator based on all four sources, and in the rest of the world, we calculate the indicator based on Google and Facebook data. Because the sources tend to provide systematically different estimates, and when a given location is missing data from a component source, we impute values for the missing source based on the available source(s) and the global median ratio(s) with the missing source.

After all missing dates and sources have been imputed, we average across sources and take a 5-day rolling mean using Gaussian process regression to smooth over time. For locations where we are missing data early in the time series, we use Holt smoothing back in time, linear damped with phi = 0.9 to create a full time series from 01 January, 2020 through the most recent available date of data. In sub-national and national locations where we are missing data, we impute the national and regional averages respectively.

Once we have generated a full location/time series dataset of mobility, we fit a linear regression using an open source mixed effects solver SLIME (https://github.com/zhengp0/SLIME/) to determine the effects of social distancing mandates in each location. SLIME provides functionality to incorporate bounds and a Gaussian prior to the total effects $(\beta_i + \mu_{il})$, which is important for guiding the regression finding the correct coefficients. We calculate mobility as:

$$Mobility_{lt} = (\beta_1 + \mu_{1l})SD1_{lt} + (\beta_2 + \mu_{2l})SD2_{lt} + (\beta_3 + \mu_{3l})SD3_{lt} + (\beta_4 + \mu_{4l})PSD1_{lt} + (\beta_5 + \mu_{5l})PSD3_{lt} + (\beta_6 + \mu_{6l})Anticipate_{lt} + e_{lt}$$

Where $Mobility_{lt}$ is the percent change from baseline for a given location (*l*) and time (*t*), $SD1_{lt}$, $SD2_{lt}$, $SD3_{lt}$, $PSD1_{lt}$, and $PSD3_{lt}$ are indicator variables for five social distancing mandates—stay at home order, school closures, essential business closures, restricted gathering order, and partial business closures—set to be 1 when a policy is implemented in a given location (*l*) and time (*t*) and 0 otherwise. $Anticipate_{lt}$ is an indicator variable that is set to 1 beginning 7 days before the first mandate is implemented to account for reduced mobility prior to policy intervention. $(\beta_i + \mu_{il})$ estimates the location-specific (*l*) effect of each mandate (*i*) and e_{lt} is the residual error. We set a prior on the variance of all random effects of 0.001 and set a bound such that $(\beta_i + \mu_{il})$ must be negative for all locations (*l*) and mandates (*i*).

4.2.3 Forecasting mobility

For each location we forecast mobility based on the location-specific estimated effects ($\beta_i + \mu_{il}$) and any mandates that have been lifted or announced. Beyond the period for which we have documented policies, we use the mandate forecast model described in section 3.1.3, which estimates the location-specific proportion of mandates implemented. We assign an equal probability of being lifted to each of the mandates that are still implemented such that the total proportion of mandates implemented is equivalent to the predicted proportion. In the United States the model restricts the date of school reopening to no earlier than August 15, 2020. We do not allow mobility projections to go above zero, the baseline mobility prior to the Covid-19 pandemic.

4.3 Testing *per capita*

Testing for COVID-19 can impact the epidemic both directly and indirectly. Directly, a positive test result alerts an individual to their need to self-isolate and for their contacts to quarantine. Indirectly, higher levels of testing ensure that policy makers and healthcare professionals have accurate information when making decisions about social distancing mandates and resource allocation.

4.3.1 Data processing

Data on the number of tests administered were sourced from a combination of direct reports from government health authorities; *The COVID Tracking Projec*t for the United States, except for Washington State; and *Our World in Data* for all locations that were present in their database that we had not sourced from direct reports, supplemented by additional country resources when missing. Sources for these data are detailed in SI Table 8.

4.3.2 Use in SEIR-fit

When both daily and cumulative data were present on the same date for a given location, we gave preference to the cumulative data. When there were daily data reported in between gaps in cumulative data reports, we added the daily data to the preceding cumulative value to fill in the missing cumulative data. Dates where only positive tests were reported were dropped. Cumulative data preceded by days of no reports was shifted to the midpoint of the missing interval and scaled to equal the average daily tests over the interval. In locations where the date of the first confirmed case preceded the date of the first reported tests, we utilised the same approach of shifting to the midpoint of the interval and setting the level to the average daily tests over the interval. We then aggregated to weekly intervals and linearly interpolated the weekly data with knots placed at the middle of each week. Finally, we smoothed the weekly interpolated data using ten iterations of smoothing with a uniform kernel and a three-day bandwidth.

4.3.3 Forecasting testing

We projected levels of daily testing per capita using the location-specific mean daily difference in testing per capita for locations with data; in effect assuming that future growth in daily testing per capita will match past increases in testing. For locations that were missing testing data, we predicted the daily increase in testing per capita using the Socio-demographic Index, a composite used widely in the GBD study and reflecting income, education, and fertility. We did not allow testing *per capita* to increase in perpetuity, instead we capped the maximum daily tests at 500 per 100,000 people.

4.4 Mask effectiveness and use

We performed a meta-analysis of 40 peer reviewed scientific studies to assess mask effectiveness for preventing respiratory viral infections. We updated systematic reviews and meta-regressions of the individual benefits of mask use. We used MR-BRT (see SI section 2.5 for additional information on MR-BRT) to perform a novel meta-analysis. We also analyzed survey data on the levels and trends of mask use. This analysis sought to estimate the proportion of people who self-reported always wearing a facemask when outside their homes.

4.4.1 Data processing

To identify variation in mask use across both US and global locations during the timeframe of the current COVID-19 pandemic, we combined survey data from multiple sources. Our covariate for mask use is the proportion of adults that self-report always wearing a mask when outside their homes. For US estimates (all 50 states and Washington, D.C.), we analyzed volunteer survey data from PREMISE (https://www.premise.com/covid-19/) a crowd-sourcing data collection and analytics platform. The PREMISE survey asked respondents a variety of questions about behaviours, sentiments, and attitudes vis-a-vis COVID-19. We make use of the following: "When you leave your home do you typically wear a face mask (SELECT_ONE)" with responses "Yes, always; Yes, sometimes; No never". Respondents were also asked about their reasons for not wearing a mask. The latitude and longitude for each respondent was also provided. The date range of data incorporated in this analysis was 23 April 2020 to 21 September 2020.

We evaluated the maximum level of mask usage observed globally during the COVID-19 pandemic and used this value as an upper bound for what could be achieved in the United States (see SI Section 6.1 on scenario development). To evaluate mask usage in countries other than the US, we used volunteer survey data collected through the Facebook app, the social networking platform with more than 2 billion global users, as part of its COVID-19 symptom survey and Data for Good program (https://dataforgood.fb.com/docs/covid19/). COVID-19 symptom survey data are collected via the Facebook app by the University of Maryland (non-US) and Carnegie Mellon University (US). For the non-US estimates, we analyzed aggregated data hosted by the University of Maryland's Joint Program in Survey Methodology. While the principal focus of the survey is on self-reporting of COVID-19-related symptoms experienced by the sampled user and members of their household, we make use of responses to the following question: "In the last 7 days, how often did you wear a mask when in public?" to which there are the following responses "All of the time; Most of the time; About half of the time; Sometimes; Never; I have not been in public during the last 7 days". For this survey, daily responses are received and processed so that all respondents from the same geography are combined into one day-specific response, and then the proportion of responses for each option are determined from this composite. Facebook data have two- to three-day lags and are updated twice per week. The date of the first set of data from Facebook that we analyzed is April 23, 2020 and these continue to be updated twice per week through 28th September 2020 (https://dataforgood.fb.com/docs/covid19/). Lastly, we used the YouGov COVID-19 Behavioural Tracker survey (https://today.yougov.com/covid-19).
YouGov surveys cover 29 countries and have interviewed around 21,000 individuals each week since 1 March and up until 28th September 2020. From YouGov, we use the following question: "Thinking about the last 7 days, have you worn a face mask outside your home (e.g. when on public transport, going to a

supermarket, going to a main road)" with responses "Always", "Frequently", "Sometimes", "Rarely", and "Not at all". Respondents for "Always" were the numerator in our proportion.

4.4.2 Use in SEIR-fit

We used a smoothing model to produce estimates of observed mask use. This smoothing process averages each data point with its neighbors. Projections of mask use past the observed survey data were flat at the value of the last observed, smoothed model.

4.4.3 Forecasting mask use

Mask use by location is projected forward at a constant level from the last date of observed mask use data (21 September, 2020) through 28 February, 2021.

4.4.4 Estimation of mask use effect size

We conducted a meta-analysis to determine the efficacy of masks in reducing transmission of respiratory viruses by extracting data studies from two published meta-analyses and one additional article – these analyses are reported in greater detail in a forthcoming preprint[25]. The resulting meta-regression calculated log-transformed relative risks and corresponding log-transformed standard errors based on raw counts and used a continuity correction for studies with zero counts in the raw data (0.001). Whereas the other meta-analyses reported one outcome per study, we extracted all relevant outcomes per study. Additionally, we included additional specifications and characteristics to account for differences in characteristics of individual studies and to identify important factors impacting mask effectiveness. These include the type of population using masks (general population versus healthcare population), country of study (Asian countries versus non-Asian countries), type of mask (paper/cloth or non-descript versus medical masks and N95 masks), type of control group (no use versus infrequent use), type of disease (SARS-CoV 1 or 2 versus H1N1/influenza/other respiratory pathogens), and type of diagnosis (clinical versus laboratory). The geographic locations of the studies included: China, Singapore, Hong Kong, Thailand, Vietnam, Saudi Arabia, South Korea, Canada, Germany, and a multinational airline flight. The region with the largest proportion of studies was Southeast Asia, where 24 of 40 studies were conducted. More than half the observations (36 or 65 observations, or 55%) were of SARS-CoV 1 or 2, six of which examined SARS-CoV 2. One observation studied cloth masks, 19 studied non-descript masks, and 44 looked at surgical, medical, or N95 masks; 52 observations were diagnosed via laboratory methods. We pooled "other" masks and cloth masks so that they could represent the range of the most common masks that members of the general public might wear in a non-medical setting. Moreover, 18 observations were in the general population, while 47 examined healthcare workers or healthcare settings. We were particularly interested in studies of the general population (i.e. non-healthcare setting), including households, student populations, and airplanes. With regards to control groups, 49 observations considered "no use" as the control, while 14 considered infrequent use and one looked at pre-/-post/study design. The re-extraction and inclusion of articles not included in the other meta-analyses resulted in 65 rows of data from 41 papers. We retained one additional study from the unavailable papers for sensitivity analyses; one paper was excluded due to our inability to recreate 2x2 table and the reported odds ratio; two papers were excluded due to incorrect control groups. We calculated relative risks and corresponding log-transformed standard errors based on raw counts where available.

We used MR-BRT (SI Section 2.5) to perform a meta-analysis that considered the various characteristics of each study. Our MR-BRT analysis used random effects with gamma terms accounting for between-study heterogeneity and quantified remaining between-study heterogeneity into the width of the uncertainty interval. We considered several analyses, but in the end settled on univariate models, an intercept-only model, and several multivariate models. Our analysis considered intercept-only model, all univariate associates, and multivariate models, as well as sub-analyses for key variables like population and mask types. We also performed various sensitivity analyses to verify the robustness of the modelled estimates and found that the estimate of the effectiveness of mask use did not change significantly when we explored four alternative analyses, including changing the continuity correction assumption, using odds ratio versus relative risk from published studies, using a fixed effects versus a mixed effects model, and including studies without covariate information.

Our analysis suggested a reduction in infection (from all respiratory viruses), for all mask-wearers, by at least one-third (Relative Risk = 0.65 (0.47-0.92)) relative to controls. The intercept only model has a point estimate of 0.48 (0.42-0.56) for all users, medical or otherwise. For all non-medical mask users, we estimate the reduction in infection is 0.57 (0.45-0.74) via univariate regression, and 0.65 (0.47-0.92) via bivariate regression.

4.5 Pneumonia seasonality

Pneumonia is one of the main clinical syndromes associated with respiratory SARS-CoV-2 infection and its seasonality is marked in many locations, particularly those far from the equator. This could be due to climatic variation (relative humidity, average air temperature) or due to human behaviour (greater time spent indoors). We modelled the ratio of pneumonia deaths in a given week to the average weekly pneumonia deaths by location. As such, ratios above 1 indicate that more pneumonia deaths than the yearly average occur in that week, and ratios below 1 indicate that fewer deaths than the yearly average occur. For a map showing these estimated ratios for each US state on the final week of the year, see Appendix 5 Figure 3.

For locations where we have weekly vital registration data for pneumonia deaths, we used the data to directly model this ratio. For the United States, we used weekly pneumonia mortality data from the National Center for Health Statistics Mortality Surveillance System from 2013 to 2019 by each state. Pneumonia deaths include all deaths classified by the full range of ICD codes in J12 - J18.9. To account for uncertainty in vital registration data and model type, all ratios were estimated 1000 times in the meta-regression model. The proportion of deaths in each week was calculated as the weekly number of deaths over the annual number of deaths in a location. The standard error was calculated using the formula for binomial variance:

$$variance = \sqrt{\frac{\frac{weekly\ death\ count}{annual\ death\ count} * (1 - \frac{weekly\ death\ count}{annual\ death\ count})}{annual\ death\ count}}$$

For locations without data on pneumonia deaths, the strategy included additional models and calculations to generate estimates for all locations. We modelled the global seasonality trend pooling all pneumonia deaths data, calculated the amplitude of the seasonality time series in specific locations to model and predict the relationship between amplitude and latitude, and then used the estimated amplitude values by latitude to manipulate the amplitude of the global pattern. As such all locations without data have the same general seasonality pattern (higher in October to April in Northern

Hemisphere; higher in April to October in Southern Hemisphere), but the amplitude varies by location, depending on the latitude.

To preserve the cyclical trend of the pneumonia deaths in the model, the same 52 weeks of data were triplicated, and added to the beginning and the end of the time series. We then modelled the logit ratio of weekly deaths to annual deaths (shown in SI Figure 6) in a meta-regression tool developed at the Institute for Health Metrics and Evaluation called MR-BRT (Meta-Regression, Bayesian, Regularised, Trimmed) (see section 2.5 for additional detail). The meta-regression used a cubic spline on week and 1% trimming of the data inputs.

4.6 Time-invariant covariates

4.6.1 Lower Respiratory Infection Mortality

In the transmission model, the mortality rate due to lower respiratory infections (LRI) is captured as the location-specific age-standardised mortality death rate in the population 15 years or older. The 15+ years age-standardised LRI death rate is assumed to represent transmission of respiratory communicable diseases among adults.

Estimates of the LRI mortality rate come from the Global Burden of disease study, and methods for estimation are described elsewhere[38,39,44]. Briefly, we used vital registration and verbal autopsy data in a Bayesian ensemble model which uses out of sample validity to produce a variety of plausible models which are weighted based on their performance in the final ensemble. Estimates are produced for each age, sex, year, and location. For this analysis, we used the age-standardised rate for both sexes by location in the year 2019 (most recent complete year of estimates). For a map showing the distribution of lower respiratory infection mortality across states, see Appendix 5 Figure 2.

4.6.2 Altitude

The incidence and severity of lower respiratory infections, including pneumonia, is greater at higher elevation[45–47]. Altitude and humidity are believed to be a predictor of transmission and several studies have found greater mortality due to pneumonia at higher elevations, possibly due to decreased oxygen concentration at higher altitudes. The proportion of the population living below 100 meters above sea-level by country was obtained from the Global Burden of Disease study (http://ghdx.healthdata.org/record/ihme-data/gbd-2015-covariates-1980-2015).

4.6.3 Smoking

The adult (15+ years) age-standardised tobacco smoking prevalence in 2019 was used as a covariate. This covariate is from the Global Burden of Disease study 2019[48] and described in detail there. Briefly, we estimated the prevalence of current smokers (daily or occasional) using individual-level and aggregated available survey data. The prevalence was modelled using Space-time Gaussian Process Regression to produce smoothed estimates by space, time, age, and sex. For this analysis, we used age-standardised prevalence among both sexes. Smoking prevalence is location-specific. For a map showing the distribution of smoking prevalence across US states, see Appendix 5 Figure 4.

4.6.4 Ambient particulate matter pollution

Ambient particulate matter pollution is a covariate from the Global Burden of Disease study 2019[48] and is defined as the population-weighted mean exposure to air particles with an aerodynamic diameter less than 2.5 micrometers per cubic meter of air. Input data for this model come from satellite observations,

ground measurements, land use data, and chemical transport model simulations. Estimates are produced on a geospatial resolution and aggregated to the national level by population-weighting. This covariate is location-specific. For a map showing the distribution of particulate air pollution by US state, see Appendix 5 Figure 1.

4.6.5 Population density

Population density per pixel was calculated using *Worldpop* total population rasters and an area raster and is represented as the percentage of the population living in areas denser than 1,000 people per square kilometer (km^2). By country, we determined the proportion of the population living in discrete categories of density and aggregated categories less than 1000 per km^2 for this analysis, using 2020 estimates to approximate population.

4.6.6 Demography

Demographic data on state populations, namely the age structure of the population, is used in estimating the age-specific mortality rate calculated in SI section 4.1 to stratify all-age deaths into age-specific deaths. Age distributions were obtained from the Global Burden of Disease study[40,48,49].

4.6.7 Altitude

Altitude is captured as the proportion of the population living below a given threshold of sea level. For the sake of this analysis, we incorporated altitude as the proportion of the population living below 100 meters above sea-level by state; this value was derived from the Global Burden of Disease study (http://ghdx.healthdata.org/record/ihme-data/gbd-2015-covariates-1980-2015).

5 Intermediate quantity modelling

5.1 Mortality rate by age estimation

To determine the age pattern of mortality for each location, we assembled available data from multiple global locations (SI Table 10) and fit a hierarchical meta-regression model[37]. The dependent variable was logit-transformed deaths divided by population. We employed a cascading spline structure to capture the non-linear effect of age, borrowing information from levels higher in the cascade to inform the shape of the age effect in relatively data sparse regions. The first stage of the cascade was a model fit on all data, with random intercepts by location. The estimated spline coefficients from this global model were passed as Bayesian priors to the subsequent region-specific models, and the region-specific coefficients were passed as priors to location-specific models. For a given in-sample or out-of-sample location, the model from most detailed geographical level was used to make predictions. Finally, we divided predictions by the minimum location-specific value to obtain age-specific relative mortality ratios.

5.2 Infection fatality ratio

We estimated infection fatality ratios (IFR) using random effects meta-analysis, modeling the dependent variable as logit-transformed deaths divided by infections. To calculate the dependent variable, age-specific observations from seroprevalence studies (see SI Table 9) were multiplied by population to obtain an estimate of infections. For each population represented by a seroprevalence observation, a corresponding estimate of deaths was obtained by splitting all-age deaths into age group-specific deaths based on the population's age distribution and predicted age pattern of mortality. The model included

random intercepts by study and a spline to estimate the non-linear effect of age. The spline method allows for the estimation of a continuous age effect from observations recorded as age groups.

5.3 Infection to death duration

To estimate the time from infection to death, we brought together two distinct sources of information: published studies of time from infection to symptoms and individual patient data on time from symptom onset to death. Due to a paucity of data on the time from infection to symptom onset, we used the median time reported from a single source (5.1 days) for the first part of this duration and added it to a distribution for the second derived by pooling data from the Global Line List (https://github.com/beoutbreakprepared/nCoV2019); Ohio, USA (https://coronavirus.ohio.gov/wps/portal/gov/covid-19/dashboards); Rio de Janeiro State, Brazil (http://painel.saude.rj.gov.br/monitoramento/covid19.html); Ceara State, Brazil (https://indicadores.integrasus.saude.ce.gov.br/indicadores/indicadores-coronavirus/coronavirus-ceara); and Mexico. This pooled dataset included data on 5,125 individuals, with a median time from onset of symptoms to death of 11 days. Informed by this, we use a uniform distribution over 17 to 21 days of lag between infection and death.

5.4 Hospitalisations to death ratio

To determine hospitalisation, we use cumulative hospital to cumulative deaths ratios estimated directly from hospitalisation and mortality data in the US and Europe through July 2020. We assembled data on COVID-19 hospitalisations from a number of countries and US states as detailed in SI Table 5. We analyzed hospitalisation to death ratios using random effects meta-analysis. We used the location-specific random effect in the estimate for locations with data. In the absence of data we used the corresponding pooled effect for other countries.

As the hospitalisation to death ratios are for all-ages only, to estimate the age-pattern of the hospitalisation to death ratio, we used the age distribution of hospitalisation to death ($H\colon D$) in the US to estimate the age-distribution for other countries and states:

$$H\colon D_{ageBin}(\mathrm{loc}) = \frac{H\colon D_{ageBin}(US)\ * H\colon D_{allAge}(loc)}{(H\colon D_{ageBin}(US) * D_{ageBin}(loc))/D_{allAge}(loc)}$$

6 Fitting and predicting transmission dynamics

6.1 SEIR-fit

6.1.1 Model formulation

To project the full time-series of deaths and infections to the future, we use a transmission model with the following compartments: susceptible, exposed, infected, and removed (SEIR). In particular, each location's population is tracked through the following system of differential equations:

$$\frac{dS}{dt} = -\beta(t)\frac{S(I_1 + I_2)^\alpha}{N}$$
$$\frac{dE}{dt} = \beta(t)\frac{S(I_1 + I_2)^\alpha}{N} - \sigma E$$
$$\frac{dI_1}{dt} = \sigma E - \gamma_1 I_1$$
$$\frac{dI_2}{dt} = \gamma_1 I_1 - \gamma_2 I_2$$
$$\frac{dR}{dt} = \gamma_2 I_2$$

where α represents a mixing coefficient to account for imperfect mixing within each location, σ is the rate at which infected individuals become infectious, γ_1 is the rate at which infectious people transition out of the pre-symptomatic phase, and γ_2 is the rate at which individuals recover. This model does not distinguish between symptomatic and asymptomatic infections but has two infectious compartments (I_1 and I_2) to allow for interventions that would avoid focus on those who could not be symptomatic. I_1 is thus the pre-symptomatic compartment.

6.1.2 Basic reproductive number under control and the effective reproductive number

In this section, we derive the time-varying basic reproductive number under control, $R_c(t)$, and the time-varying effective reproductive number, $R_{eff}(t)$. For a compartmental model with static coefficients, we can calculate the basic reproductive number as the largest singular value of the next generation operator

$$R_c = \lambda_{max}(FV^{-1})$$

where F is the Jacobian of the vector of appearance rates for compartments that actively possess the virus (E, I_1, and I_2 in our case), and $V = V^- + V^+$ is the Jacobian of the vector of transport rates of the individuals between these compartments. Both Jacobians are evaluated at the state of disease-free equilibrium (i.e., when $S = N$). The appearance and transport rate vectors for our SEIR model formulation are:

$$f = \begin{pmatrix} \beta\frac{S(I_1 + I_2)^\alpha}{N} \\ 0 \\ 0 \end{pmatrix}, \qquad v = \begin{pmatrix} \sigma E \\ \gamma_1 I_1 - \sigma E \\ \gamma_2 I_2 - \gamma_1 I_2 \end{pmatrix}.$$

We can then directly calculate the Jacobians at disease-free equilibrium:

$$F = \begin{pmatrix} 0 & \alpha\beta(I_1 + I_2)^{\alpha-1} & \alpha\beta(I_1 + I_2)^{\alpha-1} \\ 0 & 0 & 0 \\ 0 & 0 & 0 \end{pmatrix}$$

$$V = \begin{pmatrix} \sigma & 0 & 0 \\ -\sigma & \gamma_1 & 0 \\ 0 & -\gamma_1 & \gamma_2 \end{pmatrix} \Rightarrow V^{-1} = \begin{pmatrix} \frac{1}{\sigma} & 0 & 0 \\ \frac{1}{\gamma_1} & \frac{1}{\gamma_1} & 0 \\ \frac{1}{\gamma_2} & \frac{1}{\gamma_2} & \frac{1}{\gamma_2} \end{pmatrix}$$

Thus, the next generation operator is

$$FV^{-1} = \begin{pmatrix} \alpha\beta(I_1 + I_2)^{\alpha-1}\left(\frac{1}{\gamma_1} + \frac{1}{\gamma_2}\right) & \alpha\beta(I_1 + I_2)^{\alpha-1}\left(\frac{1}{\gamma_1} + \frac{1}{\gamma_2}\right) & \alpha\beta(I_1 + I_2)^{\alpha-1} \cdot \frac{1}{\gamma_2} \\ 0 & 0 & 0 \\ 0 & 0 & 0 \end{pmatrix}$$

which yields

$$R_c = \alpha\beta(I_1 + I_2)^{\alpha-1}\left(\frac{1}{\gamma_1} + \frac{1}{\gamma_2}\right)$$

6.1.3 Fitting $\beta(t)$

We denote the new daily infections output from the previous step as:

$$f(t) \approx \beta(t)S(I_1 + I_2)^{\alpha}$$

For each draw we take as constant the parameters governing the transmission dynamics other than $\beta(t)$ (i.e., α, σ, γ_1, and γ_2). These parameter values are drawn from distributions based on existing literature and can be found in SI Table 11.

With a known $f(t)$, we can solve a single simple linear ODE to get $E(t)$:

$$\frac{dE}{dt} = -f(t) - \sigma E$$

This ODE can be solved in closed form using integrating factors, or numerically. In practice we use the 4th order Runge-Kutta method (RK-4). However, it is useful to solve it in 'closed form' using the integration factor approach. Defining

$$v(t) = \int \sigma dt = \sigma t,$$

we have the closed form solution

$$E(t) = \exp(-\sigma t) \int_0^t -f(\tau) \exp(\sigma\tau)\, d\tau + C \exp(-\sigma t)\,, \quad C = E(0)$$

Having obtained $E(t)$, we repeat the process, solving for $I_1(t)$ and $I_2(t)$:

$$\frac{dI_1}{dt} + \gamma_1 I_1 = \sigma E(t)$$

$$
\begin{aligned}
&I_1(t) = \exp(-\gamma_1 t)\left(\int_0^t -f(\tau)\exp(\sigma\tau)\,d\tau\right) + I_1^0 \exp(-\gamma_1 t) \\
&= F_1(t) + I_1^0 \exp(-\gamma_1 t) \\
&\frac{dI_2}{dt} + \gamma_2 I_2 = \gamma_1 I_1(t) \\
&I_2(t) = \exp(-\gamma_2 t)\left(\int_0^t \gamma_1 I_1(\tau)\exp(\gamma_2\tau)\,d\tau\right) \\
&= \exp(-\gamma_2 t)\left(\int_0^t \gamma_1\left(F_1(\tau) + I_1^0\exp(-\gamma_1\tau)\right)\ \exp(\gamma_2\tau)\,d\tau\right) \\
&= \exp(-\gamma_2 t)\left(\int_0^t \gamma_1\, F_1(\tau)\exp(\gamma_2\tau)\,d\tau\right) + \frac{I_1^0}{\gamma_2-\gamma_1}\left(\exp(-\gamma_1 t) - \exp(-\gamma_2 t)\right) \\
&= F_2(t) + \frac{I_1^0}{\gamma_2-\gamma_1}\left(\exp(-\gamma_1 t) - \exp(-\gamma_2 t)\right)
\end{aligned}
$$

where $E(t)$ is known when solving I_1, and then $I_1(t)$ is known when solving for I_2. While useful for formulation to think of the exact solutions, the integrals must still be solved numerically. We therefore solve all the differential equations using Runge-Kutta order 4. With $f(t)$ in hand, we also obtain $S(t)$ by simple integration and subtraction. Having solved for $S(t)$, $I_1(t)$, and $I_2(t)$, we then have:

$$\beta(t) = \frac{Nf(t)}{S(t)\big(I_1(t) + I_2(t)\big)^{\alpha}}$$

6.2 β regression

6.2.1 Overview

With $\beta_f(t)$ fit to the data, we next perform a log-linear regression using the open source mixed effects solver SLIME (https://github.com/zhengp0/SLIME) to determine the strength of the relationship between $\beta_f(t)$ and the various covariates. All covariates are assumed to have fixed effects while the intercept is allowed to vary by location. For location l, the regression is calculated as:

$$\ln\big(\beta_{p,l}\big) = \alpha_{0,l} + \boldsymbol{X}_l\boldsymbol{\alpha}$$

such that the mean squared error between $\beta_{p,l}$ and $\beta_{f,l}$ (our fit from the previous stage) is minimised by location l. $\alpha_{0,l}$ is the random intercept for location l, $\boldsymbol{X}_l$ is a matrix with a column for each covariate in the regression and a row for each day, and $\boldsymbol{\alpha}$ is the coefficient indicating the strength of the relationship between $\log\beta$ and the covariate. Several coefficients in the model are bounded as described in their corresponding sections, while others are only constrained by directional bounds. As noted in (previous sections), not all covariates are time varying. These non-time varying covariates are used to explain some of the location specific variance otherwise absorbed into the random intercept. The time-variance and bounds of the coefficients are denoted in SI Table 11. A sensitivity analysis removing the constraints was conducted and described in SI Section 8. Using the fitted α and the forecasted covariates, we produce, by draw, estimates of future transmission intensity $\beta_p(t)$.

6.2.2 Fitted regression coefficients

By draw, across all locations, each coefficient is approximated with a point estimate. Uncertainty in estimated coefficients, therefore, is calculated by looking at the distribution of these point estimates

across draws. Boxplots of these distributions are plotted by location in panels I through Q of even-numbered figures in Appendices 1-3; summary statistics are described in SI Table 6. In addition to sharing the same coefficients for the location or location time varying coefficients, each location has a fitted random effect $\alpha_{0,l}$ with distributions derived similarly to those of the fined effects. Boxplots of these distributions are plotted by location in panel R of even-numbered figures in Appendices 1-3.

6.2.3 Regression diagnostics

Although the model is fit in log-β space, it may be of more practical importance to compare the fit of the β regression in natural space. To this end, we compare, by draw and by location, the time-varying input β from SI Section 2.6 (Appendix 4 figures, panel A) with the time-varying fitted β predicted from the β regression (Appendix 4 figures, panel B). By draw, we then take the difference between these to calculate residual estimates (again, in natural space, not in log space) (Appendix 4 figures, panel C). As with the estimates of the coefficients from the β regression, the uncertainty displayed in each panel is based on the distribution of the values across draws. As expected, the uncertainty in the β residuals is narrower than that of either the input β trajectories or the fitted β trajectories.

To quantify the performance of the regression, we then calculated the root mean squared error (RMSE) associated with these residuals. Our infection estimation model has difficulties accurately quantifying the distribution of infection over the first few days of a location's outbreak which thus corresponds with unreasonable variation in β values over this time. As such, and due to this period's relative minor impact on the β regression overall, we illustrate our RMSE by calculating the square root of the average squared residual from April 1st to the present across locations (Appendix 4 figures, panel D). Once again, uncertainty is based on the distribution of RMSE across draws. While there is draw level differences in α, γ_1 and γ_2 that impact how β translates into R_c, at the mean level of each quantity, an error in estimating β of 0.125 translates into an error in estimating R_c of 0.5. Of course, as the outbreak progresses, an error of 0.125 in β would then result in a smaller and smaller error in R_{eff} (directly proportional to the fraction of the population that remains susceptible. The large majority of states have median RMSEs less than 0.125 (41/53 states, counting the three sub-regions of Washington state separately), with notable exceptions in states with very low transmission (e.g., Montana and Hawaii). Finally, to assess the potential for changes in model performance through time, we alter the starting window across which we calculate the RMSE from April 1st forward in time a by week up through August 1st (each time taking the RMSE up through the present) (Appendix 4 figures, panel E). While there is some variation in states through time, again the performance of the model remains consistent as we focus more and more on the recent past model fit, with most median RMSEs below 0.125. We note that New York has an increase in RMSE over time, but as they have experienced a substantial outbreak, R_{eff} would be substantially smaller than R_c and thus this error would have a diminished impact.

6.3 β adjustments

To ensure continuity from our fitted β_f from SEIR-fit to the predicted β_F into the future, we shift the predicted β_p. Generally speaking, we shift β_p towards β_f by first ensuring that on the day of transition, say T, $\beta_F(T) = \beta_f(T)$. Then, over a window of time we slowly transition from the hard adjustment based on the residual at time T, we shift by the average residual between β_f and β_p over a window of time in the past. More specifically, define $r(t)$ as

$$r(t) = \log\left(\frac{\beta_f(t)}{\beta_p(t)}\right), \quad t \leq T$$

and $\beta_{F_1}(t)$ and $\beta_{F_2}(t)$ as:

$$\beta_{F_1}(t) = \exp\big(r(T)\big)\,\beta_p(t), \quad t \geq T$$
$$\beta_{F_2}(t) = \exp\left(\frac{1}{n}\sum_{i=1}^{n} r(T - i + 1)\right)\beta_p(t), \quad t \geq T$$

and transition weights $w(t)$ as

$$w(t) = \begin{cases} \dfrac{M - (t - T)}{M}, & T \leq t \leq T + M \\ 0, & otherwise \end{cases}$$

Then, for a given n and M, we define $\beta_F(t)$ as

$$\beta_F(t) = w(t)\beta_{F_1}(t) + \big(1 - w(t)\big)\beta_{F_2}(t)$$

Based on out-of-sample tests similar to those described in the sensitivity analyses for optimal values of M and n, we found that the optimal n was 42 and the optimal M was for M to be, by draw, drawn from a uniform distribution of windows from 7 to 28 days.

6.4 SEIR-predict

The general format of our predictions is relatively simple: we take the final predicted β_F and run our system of ODEs forward in time using our fitted compartment values at time T as the initial conditions of the second SEIR model.

There are however a number of simplifications made within our modelling formulation. First, we ignore the potential for importation which may be more likely in larger, more dense locations. Second, we assume a well-mixed population which may be more egregious in smaller, less dense locations. As two intermediate solutions for this, we introduce two correction factors. In each location we only use one or the other correction factor, and the use and magnitude of the correction is based on OOS predictive validity dropping 8 weeks of data and comparing the predicted outbreak to the observed one. The first correction factor allows for the addition of a small number of additional infections above and beyond those from the interaction between I_1 and I_2 and S. These can be envisaged as individuals traveling outside the location, becoming infected, and returning as exposed individuals. The second correction factor removes a small fraction of exposed individuals from the E compartment and moves them directly to the recovered compartment. Our model acts on the fraction of individuals who are infectious, exposed, etc, and the results of allowing for fractional infectious individuals (and no possibility for truly 'zero' infections) can alter the dynamics for small locations. These corrections can be mathematically described using θ^+ and θ^- for the importation correction and the small location correction, respectively. Again, each location receives only one of these and they alter the SEIR model formulation for prediction as:

$$\frac{dS}{dt} = -\beta(t)\frac{S(I_1 + I_2)^{\alpha}}{N} - \theta^{+}S$$
$$\frac{dE}{dt} = \beta(t)\frac{S(I_1 + I_2)^{\alpha}}{N} - \sigma E + \theta^{+}S - \theta^{-}E$$
$$\frac{dI_1}{dt} = \sigma E - \gamma_1 I_1$$
$$\frac{dI_2}{dt} = \gamma_1 I_1 - \gamma_2 I_2$$
$$\frac{dR}{dt} = \gamma_2 I_2 + \theta^{-}E$$

With these correction factors identified, we can then run our ODEs forward (again using the Runge-Kutta 4 algorithm), to have a complete time-series of infections through the end of the year.

6.5 State-specific β regression results

With the state-specific static and time-varying covariates, the fitted coefficients, and the state-specific intercept, we calculate fitted values of β. In Appendices 1 through 3, we display, by state, first the values of each of the covariates (though time where appropriate) and then the resultant fitted beta. Within each covariate panel, we combine the values of the covariates that were used in the regression as well as the predicted values of the covariates across the various scenarios. Using the predicted values of the covariates, as well as the residual averaging described in Section 5.3, we estimate future values of β inpanel B.

There are numerous diagnostics that we provide for each state (and each scenario). Here we describe how these diagnostics can aid in the interpretation of our model for three exemplar settings: California, New York, and Texas.

6.5.1 Exemplar 1: California

Beginning with Appendix 1 Figure 12, panel G displays the resultant smooth estimate of daily deaths, here based on observed daily deaths and observed daily cases. The final red spline represents the mean of 1,000 draws with the lighter shaded red representing the 95% uncertainty interval. Draws from this distribution are then back-cast into draws of daily infections. These are the input to the calculation of $\beta(t)$ in SI section 5.1.3.

Each of the time-varying covariates for the β regression are plotted in the top row of Appendix 1 Figure 12, with the part of these covariates that goes into the regression indicated by those to the left of the dashed line (which represents the last day of estimated daily infections based on data). There are numerous versions of mobility and mask use (all only different in the future) corresponding to the respective scenarios (e.g., 'Mask Use Best' here is the mask covariate for the scenario where mask use goes up to 95%). The fitted value of the corresponding coefficients for each covariate (static and time-varying) is displayed in panels I through R). Here, the uncertainty is based on the distribution of the draw level mean estimates. Each of the sub-panel boxplots is identical across panels because this diagnostic comparison is comparing multiple scenarios from the same base model run (we frequently use these same diagnostics to compare one week's run to the last). Here, for example, we can see the average effect of mobility is to increase log beta by 0.095 for each 1% increase towards normal movement. We

can also see the effect of the bounds on coefficients such as mask use in that the model frequently would prefer a larger impact of masks but is bounded by our *a priori* assumption (as described in the mask use section 3.4).

Appendix 1 Figure 11 shows the resultant β fit from the regression (panel B before the dashed line). This is then used to forecast new infections in the SEIR model and the corresponding R_{eff} is plotted in panel A. A deeper diagnostic on this β regression is shown in Appendix 4 Figure 5, analogous to panels C and D in Appendix 1 Figure 11. In the detailed diagnostic,draw level difference are calculated between the 'observed' β (the β that came out of the β fitting described in SI Section 5.1.3) and the predicted β from the β regression. This difference is displayed, by draw, in panel C, and the overall distribution of draw-level root-mean squared errors (RMSEs) from April 1 to present is shown in panel D. Here we see the median RMSE is 0.09, with a range from 0.03 to 0.2. Finally, in panel E, we display the changes in the RMSE as we shorten the range of residuals we use to calculate it. For example, we see that if we only look at the residuals over the last few weeks, the range of RMSEs grows substantially, but the median is less than 0.125.

Going back to Appendix 1 Figure 11, panel D shows the calculated beta residual mean used in the adjustment of β as described in Section 5.3. Using this adjustment, and the forecasted covariates we get our final forecasted β (panel B after the dashed line). Here we see that the model was systematically under-estimating by about 0.26 (thus giving us a positive residual beta) and thus our β estimate from our regression was increased correspondingly. This final β is then re-run through the SEIR model to estimate daily infections (panel E) and using the IFR and infection-to-death delay we estimate daily deaths. These daily deaths (in death rate space) are used in the re-implementation scenario. We can see in California, we predict a mandate re-implementation would be justified on October 15th (based on the red line dropping on that day in panel E) but its effect on deaths is not seen until October 25th (based on the red line dropping on that day in panel F). Finally, results by day are aggregated to given cumulative cases by scenario and drawn and displayed on panels G and H, respectively).

Digging into the results for California, we see a large predicted increase in infections and deaths later in the year (even in the mask scenario, Appendix 1 Figure 11, panels E and F). Going to the covariates in Appendix 1 Figure 12, we see that mask use is already at 62%, and testing is already at 0.00325, so there is not much room for improvement. Conversely, mobility was never estimated to be extremely low and is projected to only be 15% below normal by the end of the year in the mandate easing scenario. Thus, the main time-varying covariate that changes in the remainder of 2020 is the pneumonia seasonality covariate, and it is this covariate that drives our estimated increases.

6.5.2 Exemplar 2: New York

Following the same path through the diagnostics for New York as we did for California, we start with Appendix 1 Figure 68, panel G, and the estimated daily deaths. In this panel, reported data on daily deaths, daily infections, and daily hospitalisations are combined and fit to a spline to produce 1,000 draws of estimated daily deaths. We can see the huge peak in deaths observed in mid-April and the substantial and sustained decline since that point.

Regarding the covariates, shown in Appendix 1 Figure 68, panels A through F, we also see that mobility reached much lower levels in New York than California but has already returned to being only 27% below baseline. Testing rose dramatically in June and has stayed relatively stable; mask use also climbed

in the same time period to approximately 60%. Panels I through R of the same figure show coefficients fit to each covariate. Because most coefficients are based on a global fit, they remain the same across locations; the exception is the location-specific random intercept, shown in panel R.

Appendix 4 Figure 33 shows the 'observed' and predicted β for New York. Although our regression predicts a decline in transmission in New York, our covariates are unable to capture the steepness of this decline (as shown by the large negative residuals in late July and August; see panel C). This translates into a relatively large RMSE when we only focus on the last few weeks of data (panel E). This in turn translates into a relatively large adjustment based on the residual averaging for β; these residuals can be seen in Appendix 1 Figure 67, panel D. Our final β forecast (Appendix 1 Figure 67, panel B) does show a predicted increase in transmission intensity, but as can be seen from the corresponding R_{eff} plot (panel A), R_{eff} is substantially lower than 1 and our forecast expects relatively little transmission. This is, of course, both a function of the model fit and the relatively large fraction of the population that has already been exposed (estimated here to be 20%), reducing R_{eff}.

6.5.3 Exemplar 3: Texas

We chose Texas as our third exemplar because, like New York, it has already experienced substantial transmission, but like California we expect it to experience substantial transmission in the future.

Following the same path through the diagnostics as we did for California and New York, we start with Appendix 1 Figure 90, panel G, and the estimated daily deaths. There was evidence of an outbreak in May and June, but more worrying there is evidence of a larger outbreak beginning in July and ongoing. This is based on both daily death data and daily case data.

Reviewing model covariates in Appendix 1 Figure 90, panels A through F, we see that testing has actually come down in recent weeks and mask use appears to have stabilised since early August. With relatively high mobility, low testing, and the pneumonia seasonality at its low point now, it is not surprising that we expect more transmission in the future. Note here that the daily death rate is currently at a point where our mandate-reimposition scenario would expect mandates to be re-imposed (as can be seen from the precipitous drop in mobility, panel B).

From Appendix 4 Figure 44 we can see that, similar to New York but going in the other direction, our model did not expect the most recent outbreak in Texas (panel C, positive residuals from mid-June forward). Given our model missing this outbreak, it is not surprising that residual averaging adjustment (Appendix 1 Figure 89, panel D) is positive, indicating an upwards adjustment of or forecasted β. Combining this adjustment with an estimated R_{eff} greater than 1 (Appendix 1 Figure 89, panel A) with covariates that in aggregate do not reduce β leads to estimates of a continued outbreak in the mandates easing scenario (panel E). In the reference scenario, mandates are re-imposed reducing mobility and thus β, leading to an R_{eff} less than 1 and a decline in transmission. However, in that scenario (and the mask scenario) we do expect β to grow for the rest of the year and in both scenarios, R_{eff} eventually surpasses 1. As all covariates except for pneumonia seasonality are relatively constant in the last two months of the year, this increase can be clearly attributed to our seasonal driver. Texas is also a good example of the complex relationship between β and R_{eff}: although the β for the mandate easing scenario is the highest, the R_{eff} is the lowest in December (due to a depletion of susceptibles). It

is difficult to see, but within the reference scenario there is also a second re-imposition of mandates at the end of 2020 as deaths climb past the re-imposition threshold (Appendix 1 Figure 89, panels A, B, E; Appendix 1 Figure 90, panel B). This occurs so late in the year that its impact is not however seen in deaths themselves (Appendix 1 Figure 89, panel F).

7 Final data combination and summarization

The transmission model produces 1,000 full time series (including projections) of infections and deaths. We summarise draws into means and 95% UIs for reporting. To control for extreme values, the top 2.5% and bottom 2.5% of draws are dropped and replaced through random resampling of the remaining 950 draws. The summarised deaths and infections are then used as inputs to the hospital resource use microsimulation (see SI Section 7).

7.1 Scenarios

In all scenarios, schools are assumed to reopen on 15 August, 2020 and mobility is projected to increase as outlined in SI Sections 3.1 and 3.2. We estimate the likely bounds on the trajectory of the epidemic by state by investigating three main scenarios: (1) continued removal of social distancing mandates (2) reimposition of social distancing mandates after a threshold of daily deaths is reached, and (3) adoption of universal masking together with threshold induced mandate re-imposition.

The "mandates easing" scenario models what would happen in each state if the current pattern of lifting social distancing mandates continues and new mandates are not imposed; the model identifier for this scenario is 2020_08_21.04.

As a more plausible scenario, we use observations from the first phase of the pandemic to predict the likely response of state and local governments during the second phase. This plausible reference scenario assumes that in each location the trend of easing SDM will continue at its current trajectory until the daily death rate reaches a threshold of 8 deaths per million. If the daily death rate in a location exceeds that threshold, we assume that SDM will be reintroduced for a six-week period. The choice of threshold (of a rate of daily deaths of 8 per million) represents the 90th percentile of the distribution of daily death rate at which US states implemented their mandates during the first months of the COVID-19 pandemic. We selected the 90th percentile rather than the 50th percentile to capture an anticipated increased reluctance from governments to re-impose mandates because of the economic effects of the first set of mandates. In locations that do not exceed the threshold of a daily death rate of 8 per million, the projection is based on the covariates in the model and the forecasts for these to 28 February 2021. In locations were the daily death rate exceeded 8 per million at the time of our final model run for this manuscript (21 September, 2020), we are assuming that mandates will be introduced within 7 days. The model identifier for this scenario is 2020_08_22.01.

The final boundary scenario of universal mask wearing evaluates what would happen if 95% of the population in each state always wore a mask when they were in public. This value was chosen to represent the highest observed rate of mask use observed globally during the COVID-19 pandemic through June of 2020 (SI Section 3.4). In this scenario, we also assume that if the daily death rate in a state exceeds 8 deaths per million, SDMs will be reintroduced for a six-week period. The model identifier for this scenario is 2020_08_22.02.

Two additional, derivative scenarios were included to assist understanding and policy resolution of these main framework scenarios: a less comprehensive mask scenario of 85% public use of masks and a scenario of universal mask wearing in the absence of any additional NPIs. The less comprehensive

mask wearing scenario evaluates what would happen if 85% of the population in each state always wore a mask when they were in public. As with the universal mask scenario, we also assume that if the daily death rate in a state exceeds 8 deaths per million, SDMs will be reintroduced for a six-week period. The model identifier for this scenario is 2020_08_22.07. Universal mask use by 95% of the population was also evaluated in a scenario that assumes no imposition of other NPIs at any threshold value of daily deaths. The model identifier for this scenario is 2020_08_22.07. Cumulative deaths for 21 September 2020 through 28 February 2021, maximum estimated daily deaths per million population, date of maximum daily deaths, and estimated *Reffective* on 28 February 2021 for these additional derivative scenarios are available in Supplemental Information Table 12.

All scenarios presume an increase in mobility associated with the opening of schools across the country.

8 Hospital resource use microsimulation

The hospital use microsimulation is run for each projected death across time and across death-draws.

For each death, we:

1. Simulate the age of the deceased using normalised estimated mortality rates as the probability for belonging to that age. That is, we assign the death to $ageBin_i$ with probability $MR_{ageBin_i}(loc)/\Sigma_i MR_{ageBin_i}(loc)$. Call this A_D. See Section 5.1 for more details.

2. We determine how many days prior to death the deceased entered the hospital. Based on data from New York State we set this to be 6 days prior to death.

3. We assign the deceased to an ICU bed for their entire admittance period.

4. Based on A_D, we use $H\colon D_{A_D}$ to estimate the number of individuals of the same age group that would have entered the hospital on the same day as the deceased to result in 1 death in that age group on the date of death. This age-hospital-cohort will pass through the hospital and all are assumed to survive. See section 5.4 for further details.

5. For each individual in the age-hospital-cohort, they have a 6.3% chance of getting admitted to the ICU (see note below on derivation of 6.3%).

    a. Those that visit the ICU are assumed to have a hospital stay of 20 days, the middle 13 of which are in the ICU.

    b. Those that don't visit the ICU are released after eight days.

6. To determine ventilation use, we assume 85% of individuals in the ICU require invasive mechanical ventilation based on data from New York State.

By performing this simulation for each death, and each associated member of the age-hospital-cohort, we are able to summarise future hospital usage needs for general beds, ICU beds, and ventilators. Finally, using a combination of data sources, we compare the estimated number of general beds and ICU beds with availability.

Notes:

1. Based on hospital data from New York State up through Mar 31, 2020, the average ICU bed counts to hospital census was 25%. Given the assumptions about lengths of stay for those who die, those who recover, and their duration in the ICU, the conditional probability of a recovering patient going to the ICU was back calculated to be 6.3% to keep the long-term ICU usage at 25%. When possible, location-specific hospitalisation data is used to calculate the probability of a recovering patient going to the ICU. In the absence of data from a particular location, the mode is used and the ICU admission probability is calculated to be 8.8% for a recovering patient.

9 Sensitivity analyses

To assess the impact of our model formulation and associated assumptions on our future predictions, we have conducted a number of sensitivity analyses. Here we present two different types of model assessment: out-of-sample (OOS) predictive validity and assessment on future predictions. For both assessments, we consider the following five alternative model formulations: 1) the base model without the mask covariate; 2) the base model without the pneumonia seasonality covariate; 3) the base model without the mobility covariate; 4) the base model without the testing covariate; and 5) the model with all of the covariates, but without the constraints on the covariate coefficients other than directional constraints.

We conduct this sensitivity analysis periodically to assess changes in model performance through time. As discussed below for mobility, the fitted relationship between any individual covariate and observed transmission may change over time, either increasing or decreasing its perceived importance. Here we present the last two sets of sensitivity analyses. It is important to note that these regressions are fit globally to 400+ locations (although here we present how well the model fits in just the US.

9.1 OOS predictive validity analyses

For the OOS predictive validity analyses, we dropped a set number of weeks of data, re-ran the entire model fitting pipeline and then compared cases and deaths predicted by week compared to the cumulative US input death estimates for the publication run. In these experiments, we used the observed values of the covariates as opposed to forecasting based on the state of those covariates eight weeks ago. Here we present two of these analyses: dropping eight weeks of data and dropping four weeks of data. Analyses 1 was run based on all data accumulated by July 4th, while Analyses 2 was run based on all data accumulated through August 14th (associated with the models presented in the main text). On those dates, there were several states which did not have available data on the last few days before the cutoff. As such, using our knowledge on those dates as the observed universe, we did not have a full set of “ground truth” for the final week and the comparisons are thus across seven weeks and three weeks, respectively.

These two sets of our analyses highlight difficulties in capturing rapid changes in transmission intensity with covariates that might not vary at the same rate. In the first analyses, we generally overestimated, and the pneumonia seasonality covariate helped constrain the predicted (and not realised) increases in transmission. In the second analyses, the all models in the first sub-experiment missed the outbreak in mid-July and August and as such the covariate that was previously reining in transmission (pneumonia seasonality) appeared to exacerbate already overly low predictions. Interestingly, in the second sub-

experiment within the second analyses (which included some of that mid-July / August outbreak), all models appeared to have the information they needed to successfully predict the rest of the way.

9.1.1 8-week OOS analyses

9.1.1.1 Analyses 1

After dropping all data back to May 9th and rerunning the entire COVID pipeline (except covariate prediction) we found the inclusion (or rather exclusion) of any of the time-varying covariates had substantial influence on predictive validity (SI Figure 7). The worst performing model, by far, was the model that excluded pneumonia seasonality as a covariate. The model that excluded masks was also substantially inferior to the base model. Conversely, models that excluded mobility or testing out-performed the full model. Finally, as expected, a model that ignored biologically plausible maximum impacts of the covariates ('Unconstrained' in SI Figure 7) was also superior to the model with the constraints in place.

At the end of the 4th week of the predictions (June 6th), full model over-estimated deaths by 13,300 deaths. While every model assessed here over estimated deaths, the model without pneumonia seasonality over-estimated the cumulative number of deaths by 17,900 (34% higher than the full model), while the model without masks estimated 16,800 more deaths than observed (26% higher than the full model). The model without mobility only over-estimated cumulative deaths by 5,600 deaths (41% of the full model's error), while the model without testing over-estimated cumulative deaths by 8,700 deaths (65% of the full model's error). The unconstrained model performed similarly to that of the model without testing, over-estimating cumulative deaths by 8,100 (61% of the full model's error).

At the end of the 7th week of predictions (June 27th), both the magnitudes of the errors as well as the relative differences grew substantially. The full model over-estimated cumulative deaths by 48,700 deaths. The model without the pneumonia seasonality covariate produced massive estimated death counts, over-estimating by 83,900 deaths (172% the error of the full model). Excluding masks also continued to result in large over-estimations, predicting 71,100 more deaths than observed by June 27th (146% the error of the full model). The models that outperformed the full model increased their dominance. The model without testing predicted 20,400 more deaths than observed (50% the error of the full model), while the unconstrained model predicted 21,600 more deaths than observed (50% the error of the full model. Finally, the model without mobility tracked actual cumulative deaths well, and 'only' over-estimated by 9,900 deaths (26% the error of the full model).

9.1.1.2 Analyses 2

After dropping all data back to June 23rd and rerunning the entire COVID pipeline (except covariate prediction) we again found the inclusion (or rather exclusion) of any of the time-varying covariates had substantial influence on predictive validity (SI Figure 8). In this OOS analyses, the model predicted fewer infections and deaths than observed across all experiments and the runs that included the pneumonia seasonality covariate (which from July to August would contributed to lowering transmission intensity performed worse than the run which dropped this covariate. The experiment without the testing covariate also did better than the full model (or the unconstrained model). Again, based on the understanding of transmission from the beginning of the outbreak to June 23rd, we would have expected both increases in testing and decreases due to seasonality to result in fewer infections and death than

were observed (that is to say, the outbreak in mid-July to August was unexpected based on what we had seen from the beginning of the outbreak up to June 23rd.

At the end of the 4th week of the predictions (July 22nd), all models were still performing well, with all errors less than 2,200 deaths (SI Figure 8) compared to an observed cumulative number of deaths of 142,278. The model that excluded pneumonia seasonality outperformed the full model with a slight overestimation of the outbreak (212 more deaths than observed) and the no-testing and no-masks models also had closer estimates of deaths than the full model, underestimating by 754 and 1,064 deaths respectively. The full model performed better than the unconstrained model underestimating by 1,257 deaths compared to underestimating by 1,900 deaths and the model that removed mobility performed the worst, underestimating by 2,148 deaths.

In the exercise where we forecasted the June 23rd model forward 7 weeks (August 11th), all models underestimated the magnitude of the outbreak by at least 13,340 deaths. The 'best' model was again the one that dropped the pneumonia seasonality. The rest of the models were all very comparable with underestimations that missed between 13,340 deaths (for the model that dropped testing) and 18,555 deaths (for the model without mobility).

9.1.2 4-week OOS analyses

9.1.2.1 Analyses 1

After dropping all data back to June 6th and rerunning the entire COVID pipeline (except covariate prediction) we again found the inclusion (or rather exclusion) of any of the time-varying covariates had substantial influence on predictive validity (SI Figure 9). Again, the worst performing model, by far, was the model that excluded pneumonia seasonality as a covariate. Interestingly, the next worst model was the one that excluded testing. The model that excluded masks was again inferior to the base model. The models that excluded mobility or removed constraints were again superior to the full model, but the superiority was diminished relative to the same forecast horizon comparisons in the 8-week analyses.

At the end of the 3rdweek of the predictions (June 27th), full model over-estimated deaths by 5,000 deaths. Again, every model assessed here over estimated cumulative US deaths. The model without pneumonia seasonality over-estimated the cumulative number of deaths by 6,100 (23% higher than the full model), the model without testing over-estimated cumulative deaths by 5,300 (7% higher than the full model, and the model without masks estimated 5,100 more deaths than observed (4% higher than the full model). The model without mobility over-estimated cumulative deaths by 3,100 deaths (62% of the full model's error), while the unconstrained model over-estimated cumulative deaths by 4,000 deaths (80% of the full model's error).

9.1.2.2 Analyses 2

After dropping all data back to July 21st and re-running the pipeline (SI Figure 10) we see marked differences in performance compared to the 8-week OOS of Analyses 2. Here every model appears to have the information needed to capture the increases seen in the past 4 weeks. From July 21st to August 14th, there were an estimated 14,514 deaths, and all experiments were able to produce estimates within 1,000 (though all were underestimates). The full model and the full model without constraints underestimated the most, missing the observed number of deaths by 990 and 985 respectively, with the

model that dropped mobility being third worst underestimating deaths by 968 deaths. The models that dropped either masks, pneumonia seasonality, or testing all performed similarly with each underestimating by fewer than 500 deaths (482, 380, and 381, respectively). It should be noted that although not displayed in the figure diagnostic, in the 4th week all models continued to perform well, but the relative rankings were almost inverted (with the full model and the full model without constraint performing the best and the model without pneumonia seasonality performing the worst, albeit still quite well).

9.1.3 Mobility and the changing transmission landscape

While the OOS analysis strongly supports the inclusion of covariates that track masks and pneumonia seasonality (as the exclusion of these covariates greatly degraded the model's predictive performance), there was also a strong indication that the mobility covariate was detrimental to the performance of the model. While these results certainly warrant further investigation, there are several clear observations that can already be made. As has been noted elsewhere, while declines in mobility strongly correlated with declines in transmission early in the outbreak, the correlation was more muted as mobility returned to normal levels[51]. One plausible hypothesis for this is that human behaviour was altered during the course of the outbreak and a measured level of 'mobility' did not mean the same in February and March as it did in April and beyond. As an example, mask usage had greatly increased over this time.

The current model formulation fits a regression to transmission intensity using the entire past of the outbreak, equally weighting each day's residual with each other day. As such, an 8-week OOS test would have relatively few days post-rebound of mobility and relatively many days when mobilities decline was strongly tied to that of transmission. Likewise, the 8-week OOS test would contain relatively few days that underscored the importance (and high usage) of masks. To dig a little deeper into this potential explanation for some of the superiority of the mobility-free model, we looked at the fitted coefficient on mobility for the 8-week and 4-week OOS full models and compared them to the production run. As can be seen in SI Figure 12, the effect of mobility on transmission intensity has decreased as more post-rebound data has entered the model. As has been seen over the weeks at the end of June and beginning of July, 2020, reductions in social distancing are coincident with increases in transmission. As such, it seems impractical to fully remove mobility from a model of COVID. That being said, the OOS assessment must be repeated continually to fully understand the impact of these covariates on model utility.

9.2 Assessment of future predictions

A different sort of sensitivity analysis that can complement the predictive validity assessment is one that investigates potential changes in the final conclusions of the study to altered assumptions. To this end, we re-predicted COVID transmission and deaths through the end of 2020 using all available data but using the altered model formulations described above. The OOS analyses illustrated the importance of investigating the impact of each covariate in the model predictions, as the mobility and mask covariates are directly tied to two of the three scenarios presented in this manuscript. Thus, the scenario we considered was the worst-case, mandate easing scenario.

9.2.1 Analyses 1

In the scenario where mandates are eased with no re-implementation, the estimated number of COVID deaths by October 1st was 184,900 from the full model. Although the pneumonia seasonality covariate indicates an increase in transmission risk in the fall, the model without pneumonia seasonality estimated

202,900 deaths on October 1st (10% larger than the full model). The model without testing and the model without masks were very similar to the full model, with 186,400 and 186,600 deaths respectively (both 1% larger than the full model). The model without mobility and the unconstrained model both produced lower estimated by October 1st, with 175,800 and 174,700 deaths, respectively (both 95% the magnitude of the full model). In general, there was agreement across all the models as to the magnitude of the loss of life due to COVID by the beginning of October.

Alternatively, there were substantial deviations across the model predictions from October through the remainder of the year. In the mandate-easing scenario, the full model predicts 399,900 deaths by January 1st. The model without pneumonia seasonality predicted a massive outbreak in the last 3 months of 2020, with and an estimated 845,200 total COVID deaths by January 1st (211% the full model value). This came from an almost doubling in total deaths in both November and December (November 1st : 273,500 cumulative deaths, December 1st: 445,800 cumulative deaths). Both the model without masks and the model without testing produced final death values 29% higher than those of the full model by January 1st (515,000 and 514,100 respectively). The unconstrained model continued to produce fewer deaths than the full model, but still ended estimating 369,400 deaths by January 1st (92% the full model). Just as the model without pneumonia seasonality estimated a massive outbreak in the last 3 months of 2020, the model without mobility estimated a large reduction in transmission over the last 3 months, estimating 49,600 deaths from October 1st to the end of the year. While this is a huge loss of life, it must be noted that the scenario used here is the worst-case scenario presented, and compared to the other models' estimates, 49,600 deaths is substantially smaller than other models. The final estimated death total is 225,400 which is 56% of the full models' estimates.

In the current COVID transmission landscape (where mandate re-imposition is being considered due to a resurgence in cases and deaths), it is possible that the effect of mobility (or any other covariate) will change once again. It remains critical to continually re-evaluate the inclusion (or exclusion) of any covariate as well as assess their impact.

9.2.2 Analyses 2

In the scenario where mandates are eased with no re-implementation, the estimated number of COVID deaths by October 1st was 222,753 from the full model. The model without pneumonia seasonality, the model without testing and the full model without constraints all predicted deaths within 500 of this (222,252, 223,137, and 222,425, respectively) while the model without masks or without mobility had deviated by more than 3,000 by October 1 (225,943 and 217,069, respectively).

The differences between models substantially increase from October 1st through the end of the year. The full model estimates 569,354 deaths by 1 January 2021 without any mandate re-imposition. Models without masks, testing, or constraints all produce much higher results (637,419 deaths, 713,618 deaths and 668,746 deaths, respectively). Conversely, models without mobility or pneumonia seasonality estimate substantially fewer deaths (399,206 and 332,491, respectively). As with the results of this sensitivity analysis for Analyses 1, it must be noted that these results are in the absence of any of the scenarios that control for future infections (e.g., mandate re-imposition or increases in mask use). The differences in those settings would naturally be smaller as the total number of infections would be reduced. Of course, we did not attempt that comparison as it would not work in the scenarios where the alteration occurs on a covariate that is dropped from the model. Nonetheless, these two analyses

underscore the need to repeatedly re-evaluate both the performance of the overall model as well as the utility of each covariate in the model to most accurately capture changes in the SARS-CoV-2 transmission landscape.

Supplementary Figures

SI Figure 1. Schematic representation of modelling process. Numbers correspond to Supplementary Information sections detailing each portion.

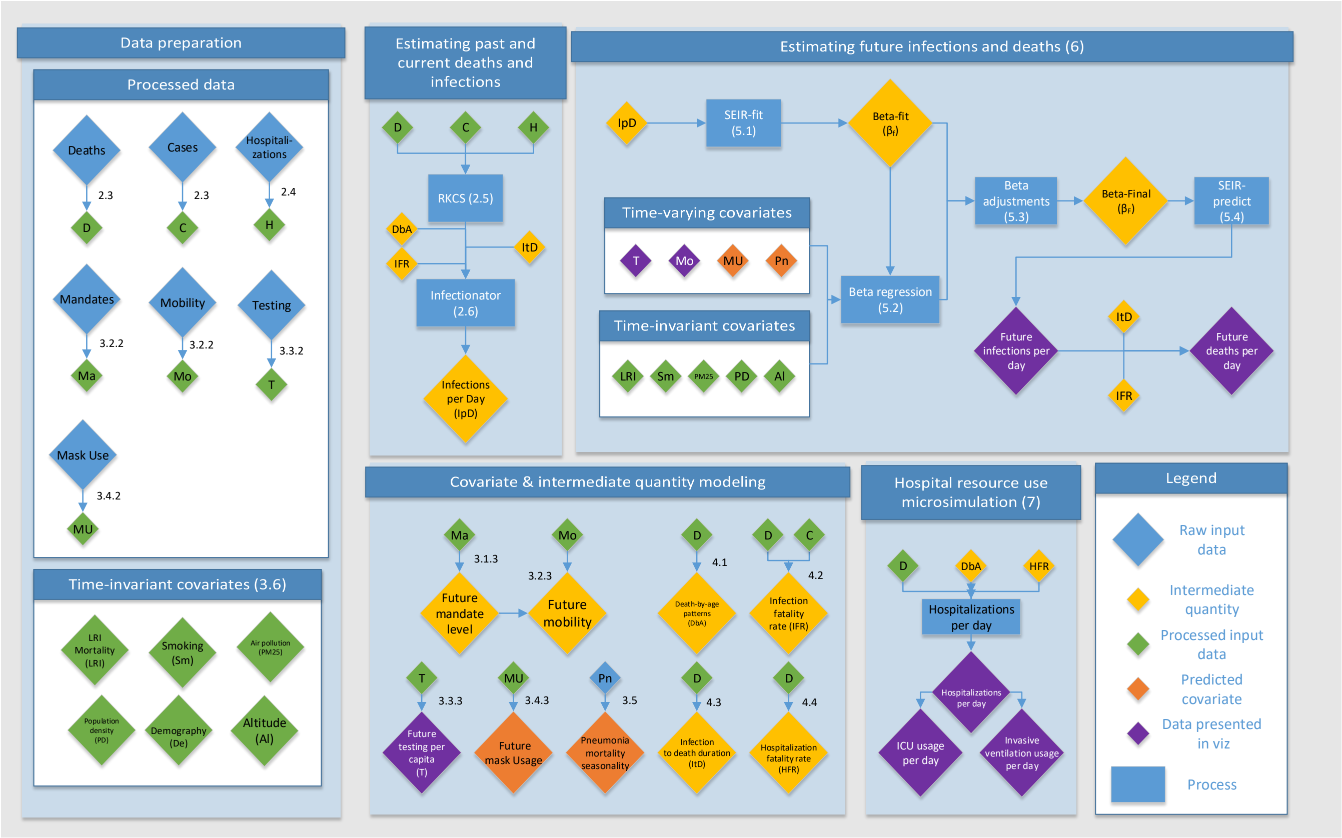

SI Figure 2 Time trends and relationship with deaths for cumulative cases and hospitalisations in Florida.

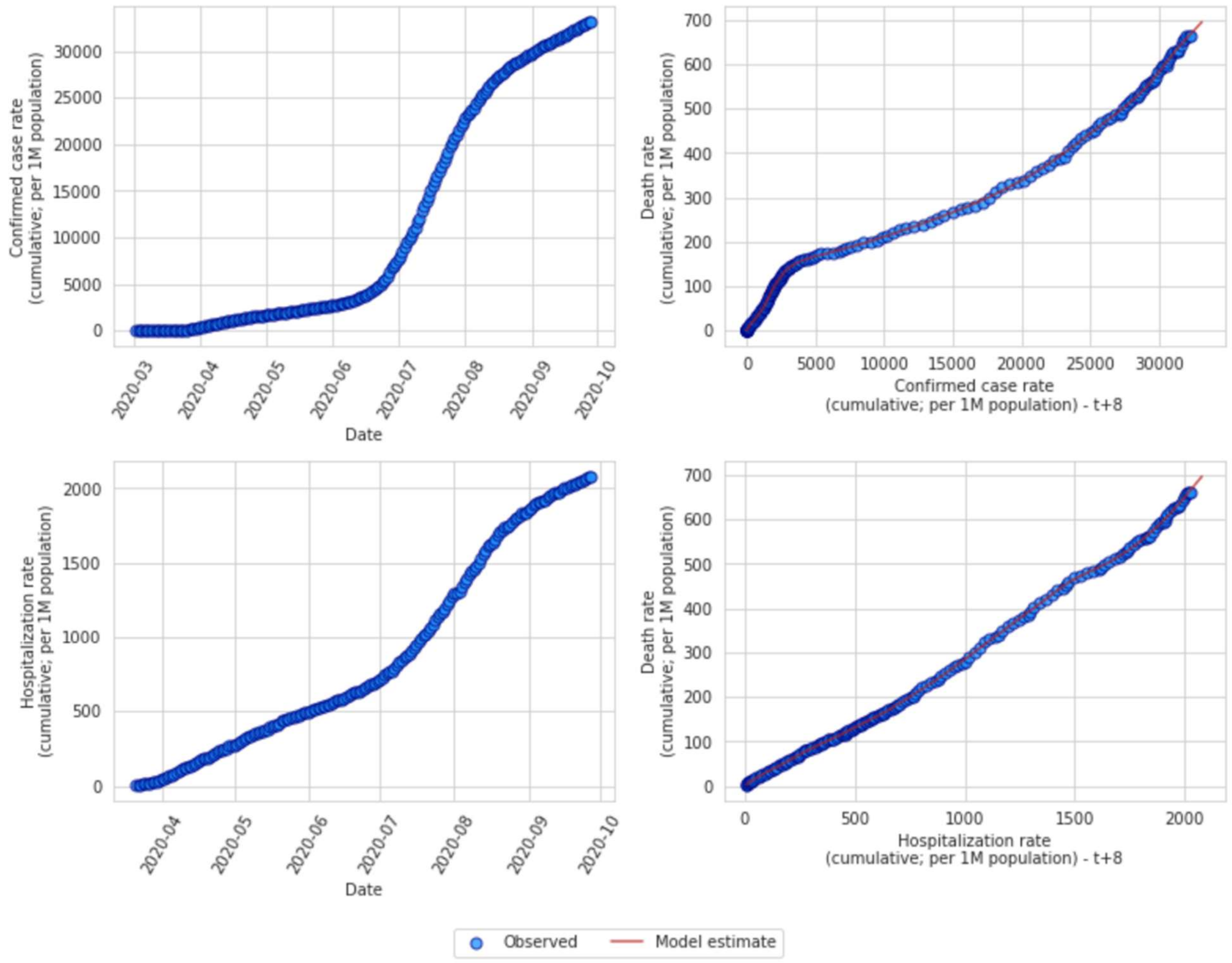

SI Figure 3 Estimated death curve in cumulative and ln(daily) in Florida. Samples from refit model are shown in ln(daily) plot.

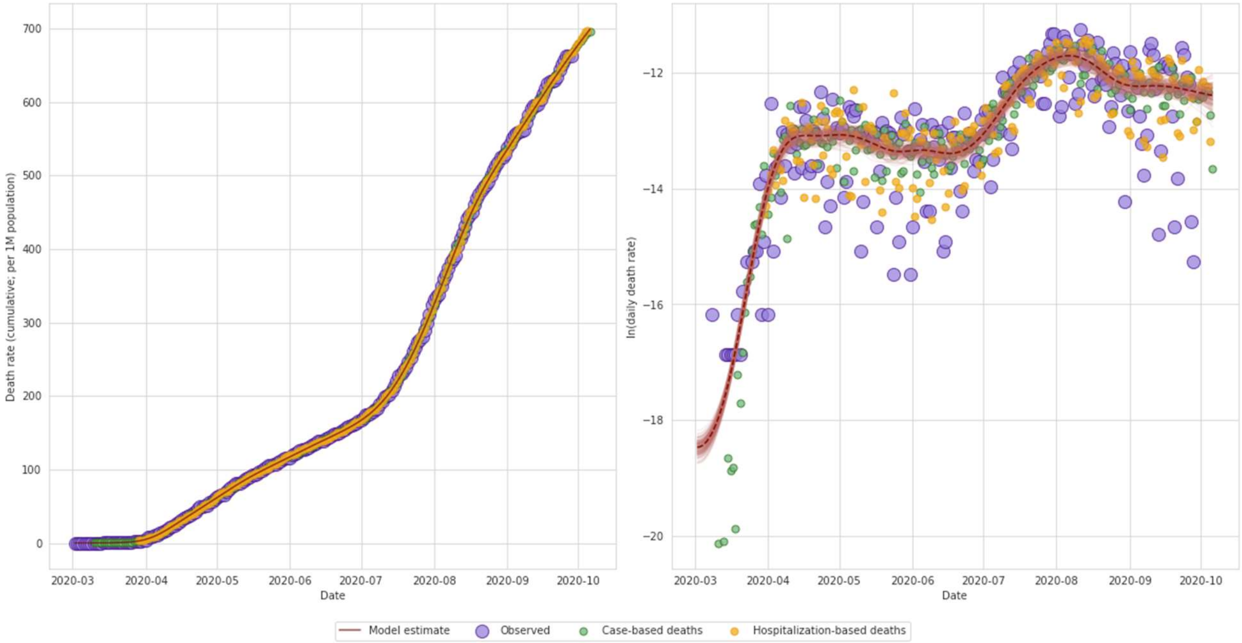

SI Figure 4. Trends of the number of mandates (out of 6) on for each location in the modelling hierarchy

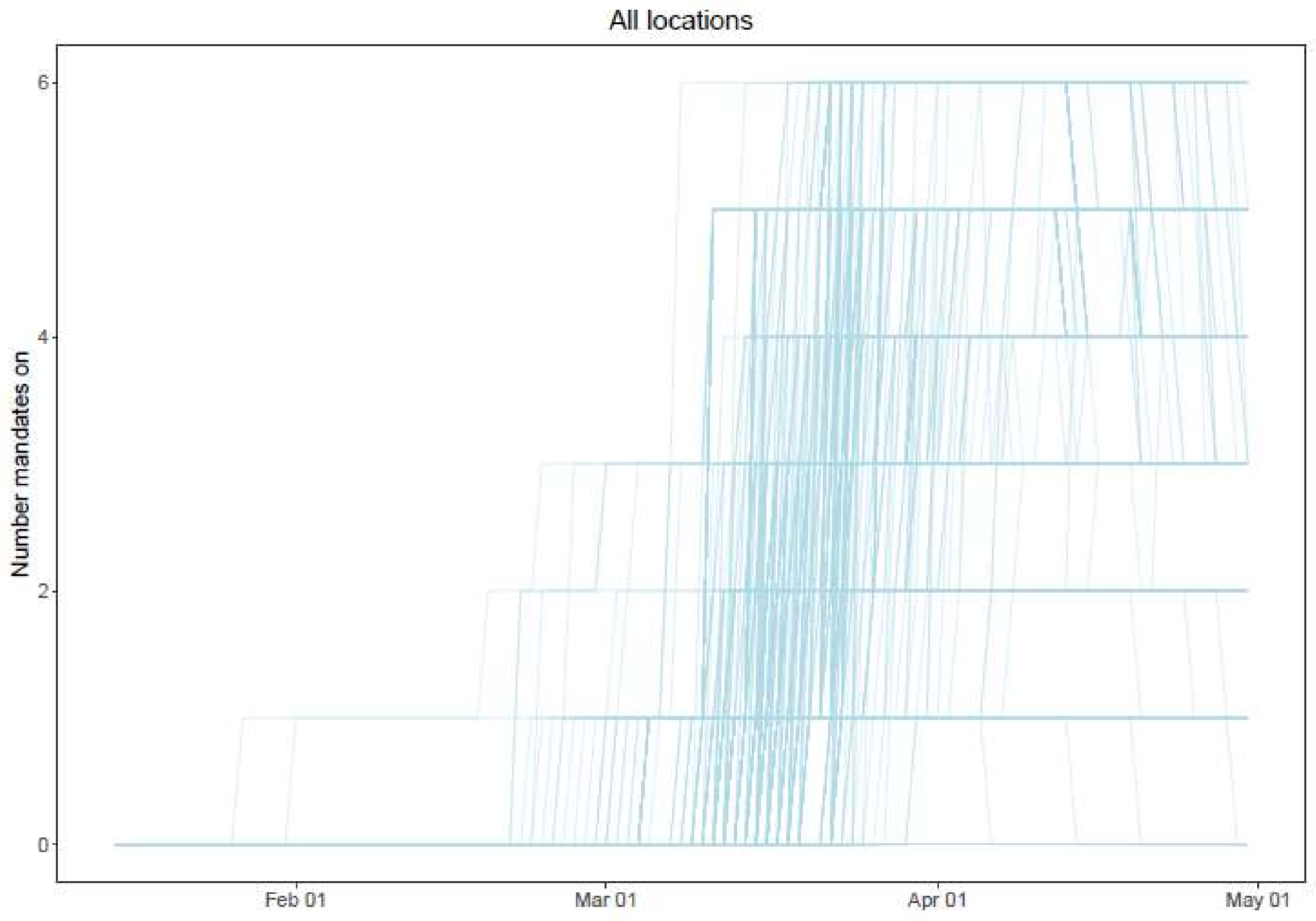

Each line represents a country, and the number of mandates implemented on a given date is shown on the Y axis. By the middle of March, most countries around the world had implemented at least 5 mandates.

SI Figure 5. Time trends of the average number of mandates “on” for each region of the world.

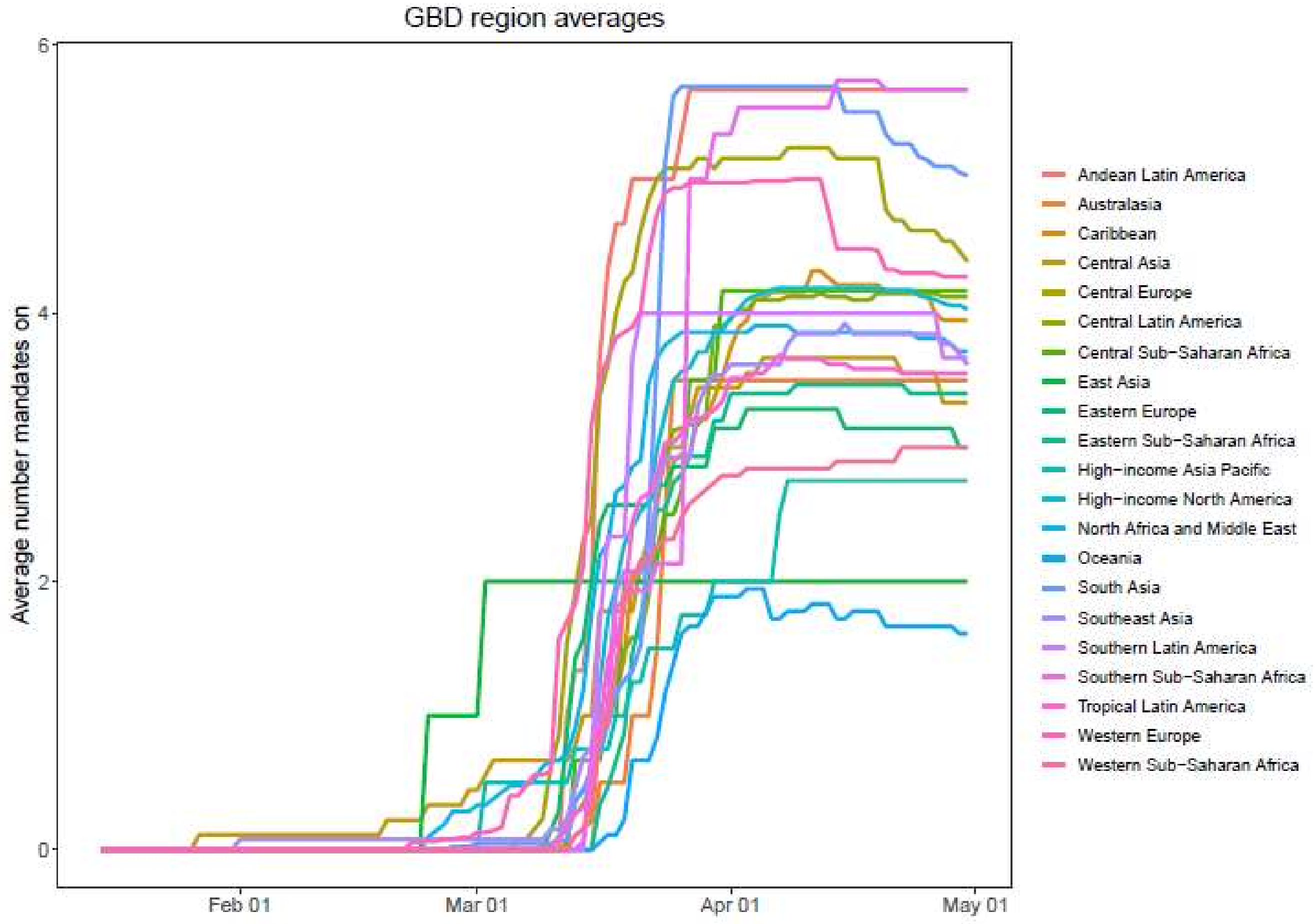

Each line represents a Global Burden of Disease region, and the average number of mandates implemented in all countries of that region is shown on the Y axis.

SI Figure 6. MR-BRT model of the US pattern of seasonality of logit ratio vs week.

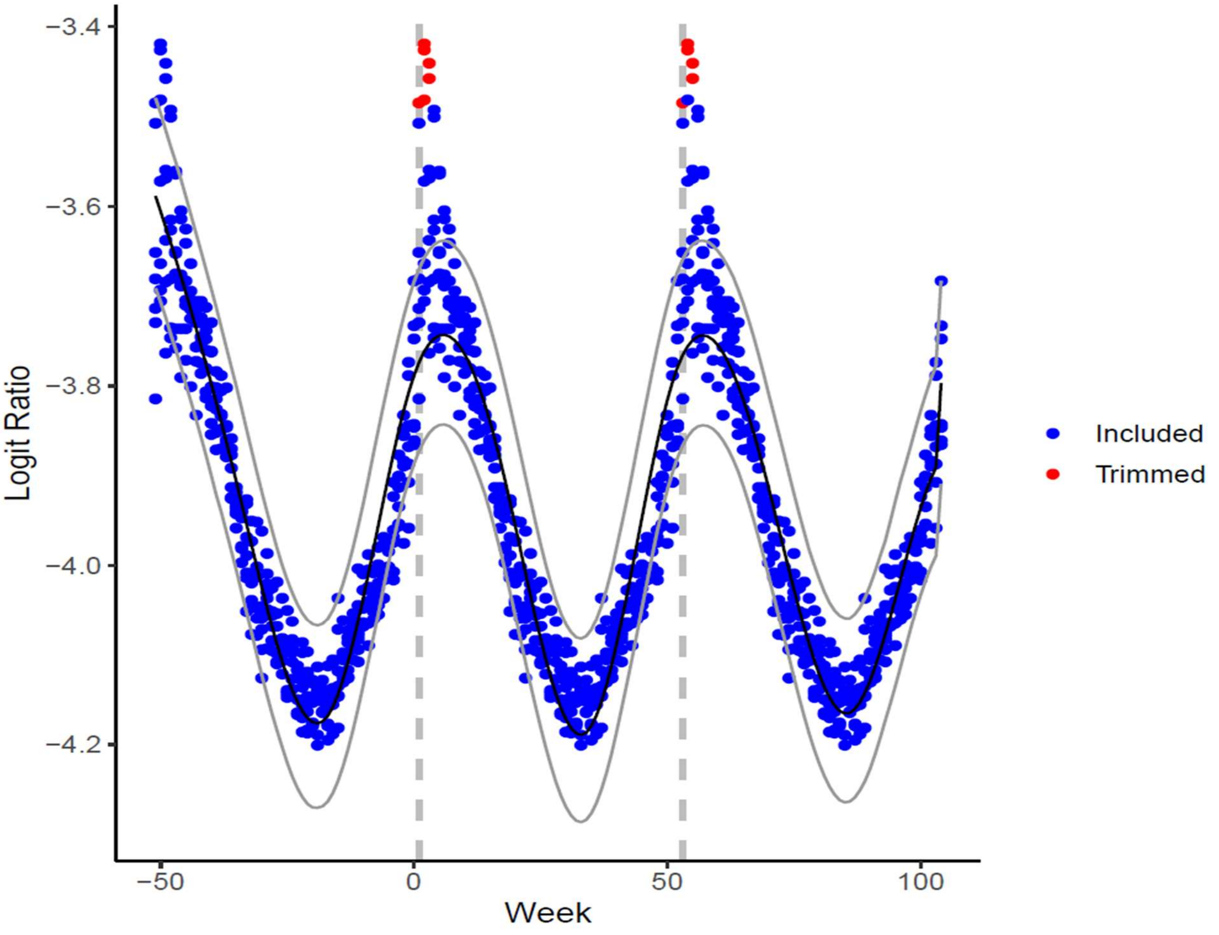

The black line shows the model estimates, the blue points are data included in the model, and the red points are data excluded from the model.

SI Figure 7. Eight week out-of-sample predictive validity (July 4 model run)

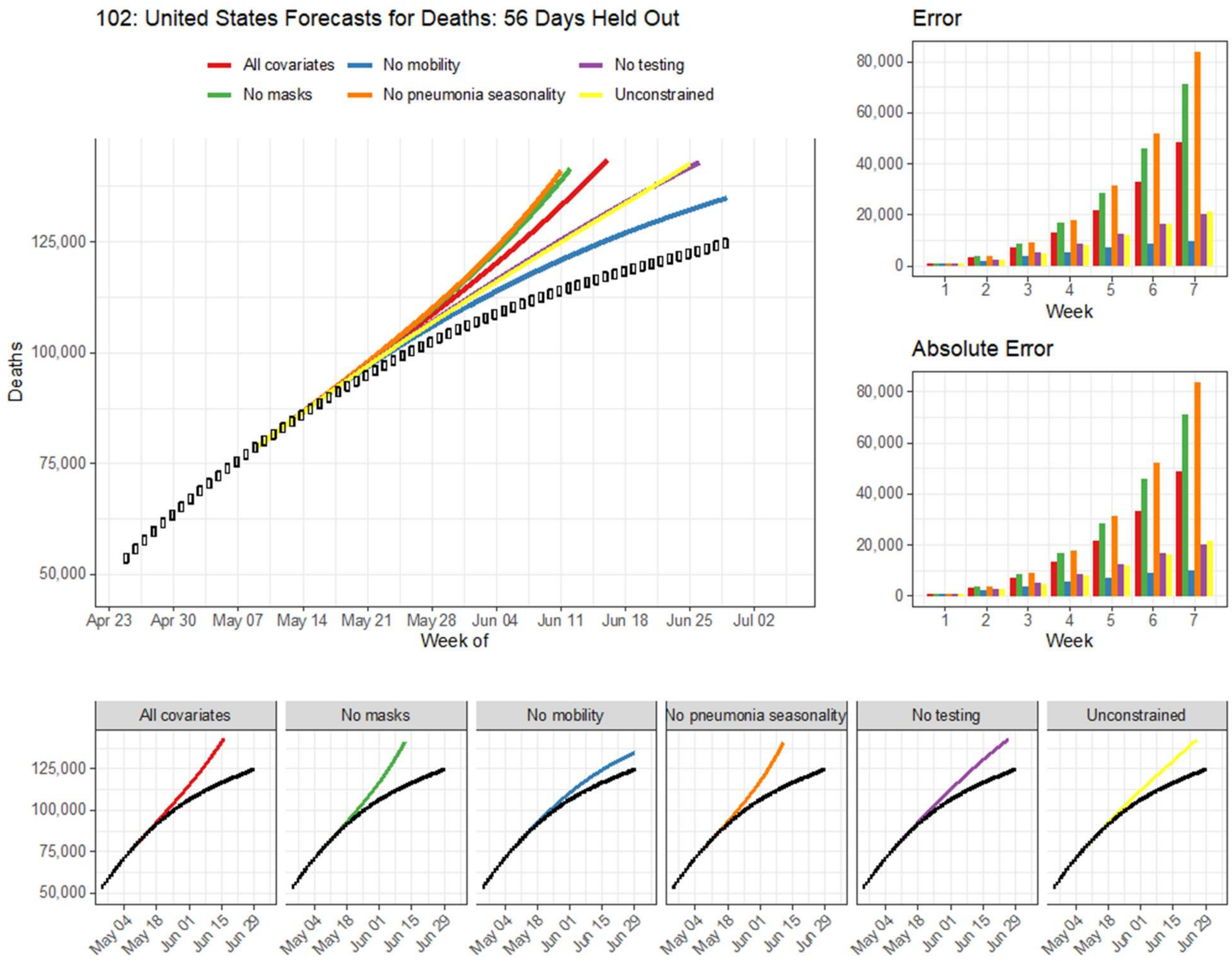

Figure panels display the results of fitting and predicting our model when holding out eight-weeks of death and case data, based on input data and covariates as of July 4. We ran our reference model (bottom row, red line) and models in which we dropped a single time varying covariate from the regression (no masks, no mobility, no pneumonia seasonality, and no testing per capita). Bayesian priors are used on the coefficients for these covariates and so in a last model we removed (‘unconstrained’) the priors. The predictions from these models are shown on the bottom row. The top left panel shows all these models compared to the observed cumulative deaths in the United States (white dots). The upper right panels show the out-of-sample error, binned by week, in both natural and absolute error.

SI Figure 8. Eight-week out-of-sample predictive validity (August 21 model run)

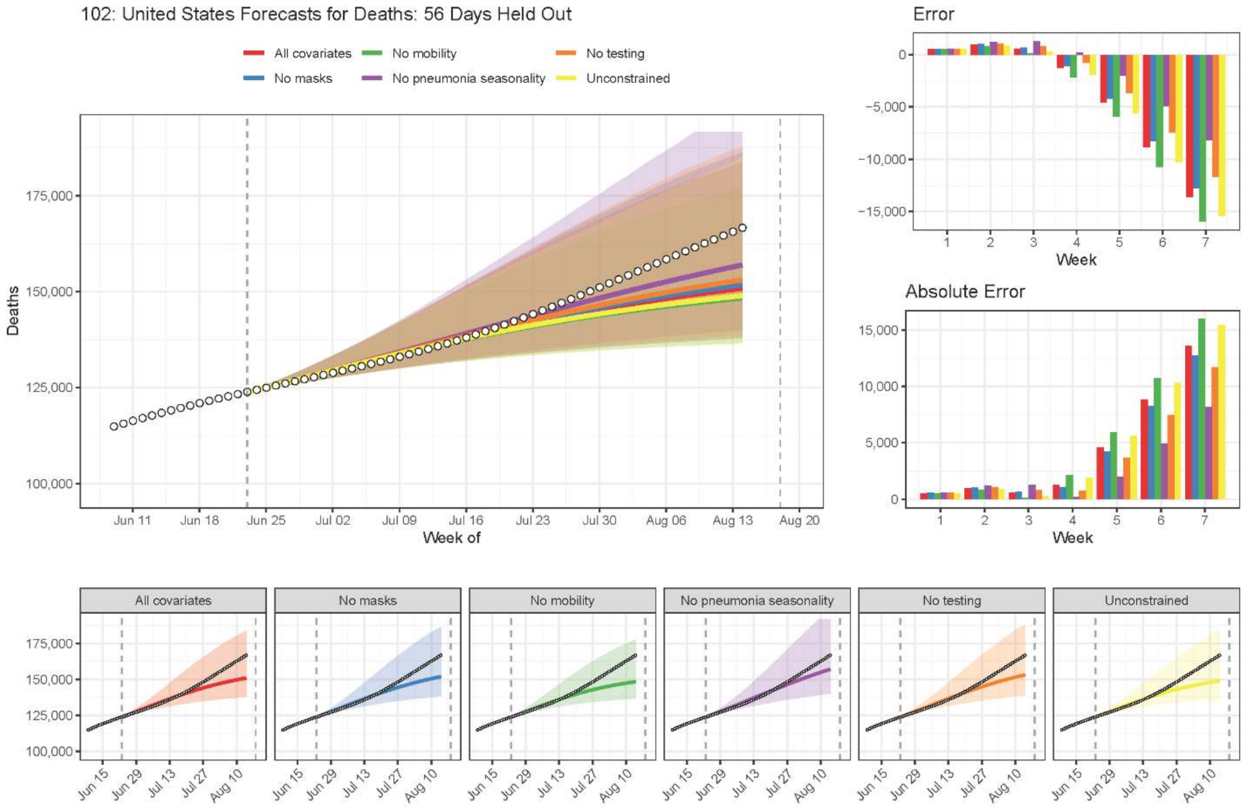

Figure panels display the results of fitting and predicting our model when holding out eight-weeks of death and case data, , based on input data and covariates as of August 21. We ran our reference model (bottom row, red line) and models in which we dropped a single time varying covariate from the regression (no masks, no mobility, no pneumonia seasonality, and no testing per capita). Bayesian priors are used on the coefficients for these covariates and so in a last model we removed ('unconstrained') the priors. The predictions from these models are shown on the bottom row. The top left panel shows all these models compared to the observed cumulative deaths in the United States (white dots). The upper right panels show the out-of-sample error, binned by week, in both natural and absolute error.

SI Figure 9. Four-week out-of-sample predictive validity (July 4 model run)

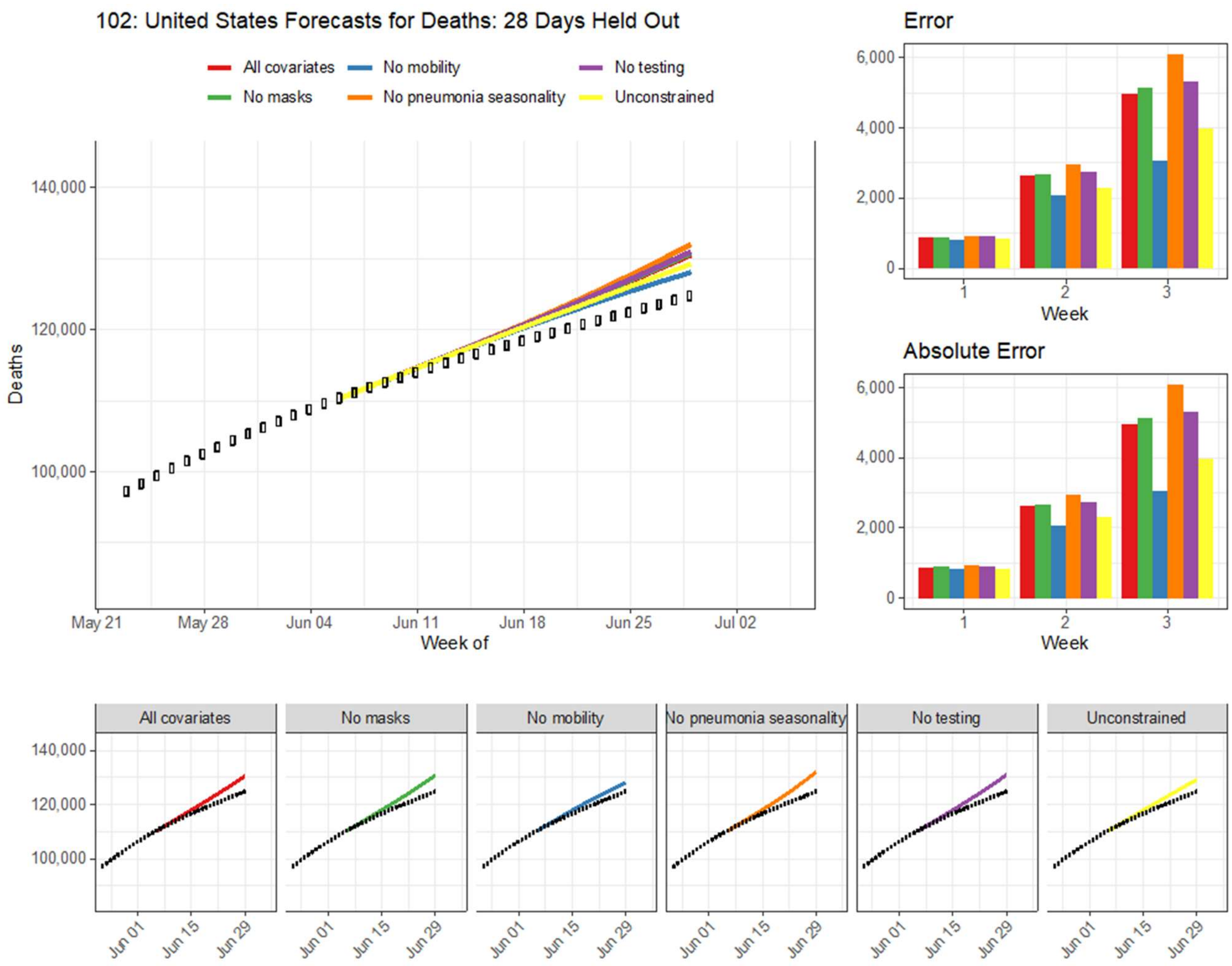

Figure panels display the results of fitting and predicting our model when holding out four weeks of death and case data, based on input data and covariates as of July 4. We ran our reference model (bottom row, red line) and models in which we dropped a single time varying covariate from the regression (no masks, no mobility, no pneumonia seasonality, and no testing per capita). Bayesian priors are used on the coefficients for these covariates and so in a last model we removed ('unconstrained') the priors. The predictions from these models are shown on the bottom row. The top left panel shows all these models compared to the observed cumulative deaths in the United States (white dots). The upper right panels show the out-of-sample error, binned by week, in both natural and absolute error.

SI Figure 10. Four-week out-of-sample predictive validity (August 21 model run)

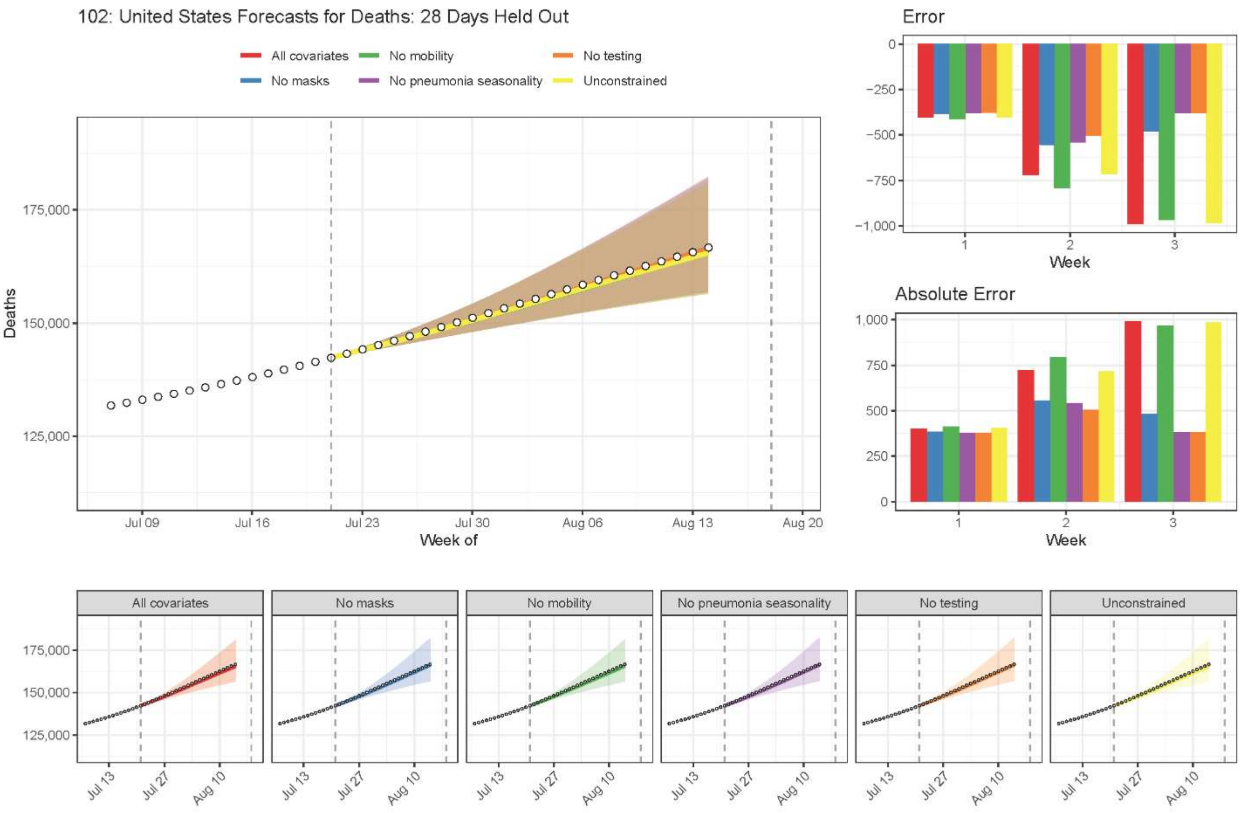

Figure panels display the results of fitting and predicting our model when holding out four weeks of death and case data, based on input data and covariates as of August 21. We ran our reference model (bottom row, red line) and models in which we dropped a single time varying covariate from the regression (no masks, no mobility, no pneumonia seasonality, and no testing per capita). Bayesian priors are used on the coefficients for these covariates and so in a last model we removed ('unconstrained') the priors. The predictions from these models are shown on the bottom row. The top left panel shows all these models compared to the observed cumulative deaths in the United States (white dots). The upper right panels show the out-of-sample error, binned by week, in both natural and absolute error.

SI Figure 11. Fitted coefficient on mobility for the 8-week and 4-week out-of-sample and full “production run” versions of the model (July 4 model run)

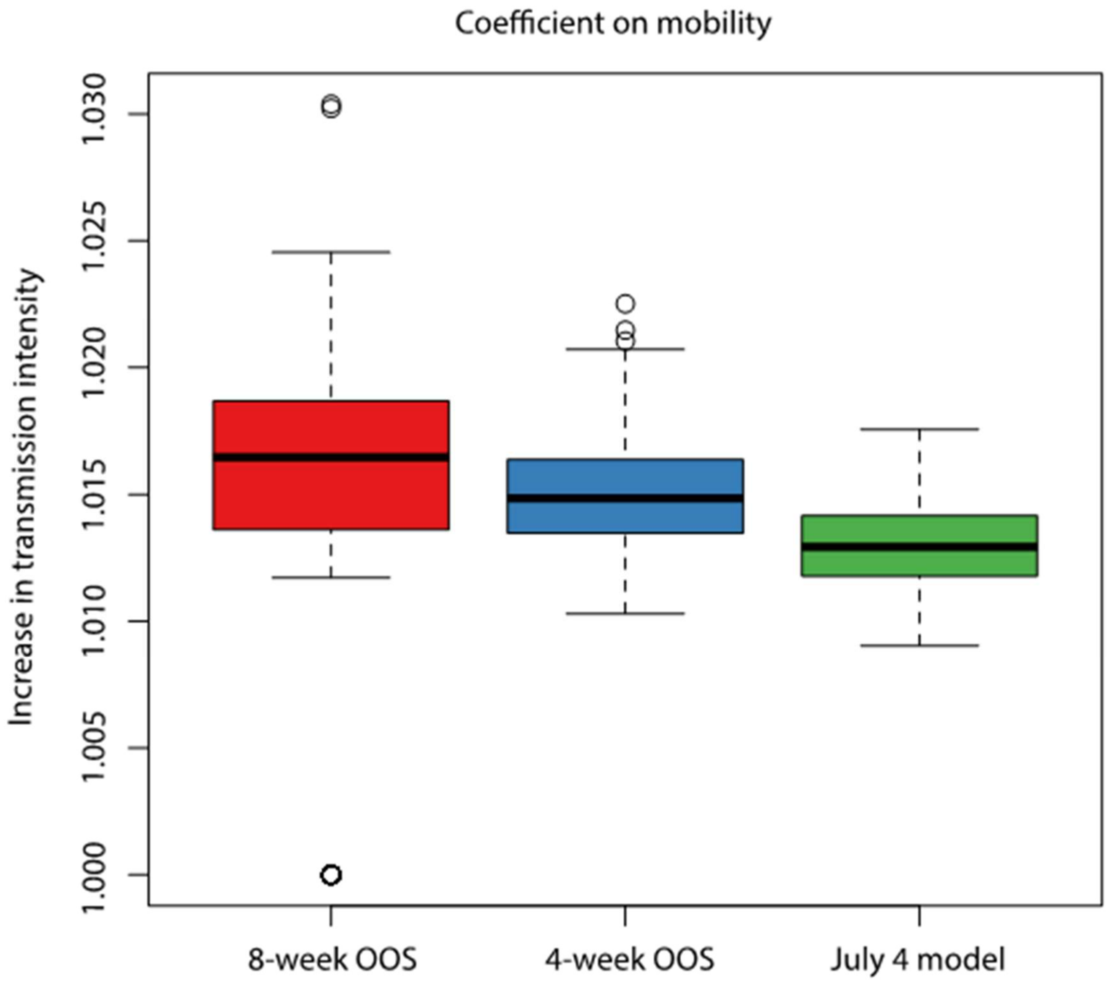

SI Figure 12. Fitted coefficient on mobility for the 8-week and 4-week out-of-sample and full "production run" versions of the model (August 21 model run)

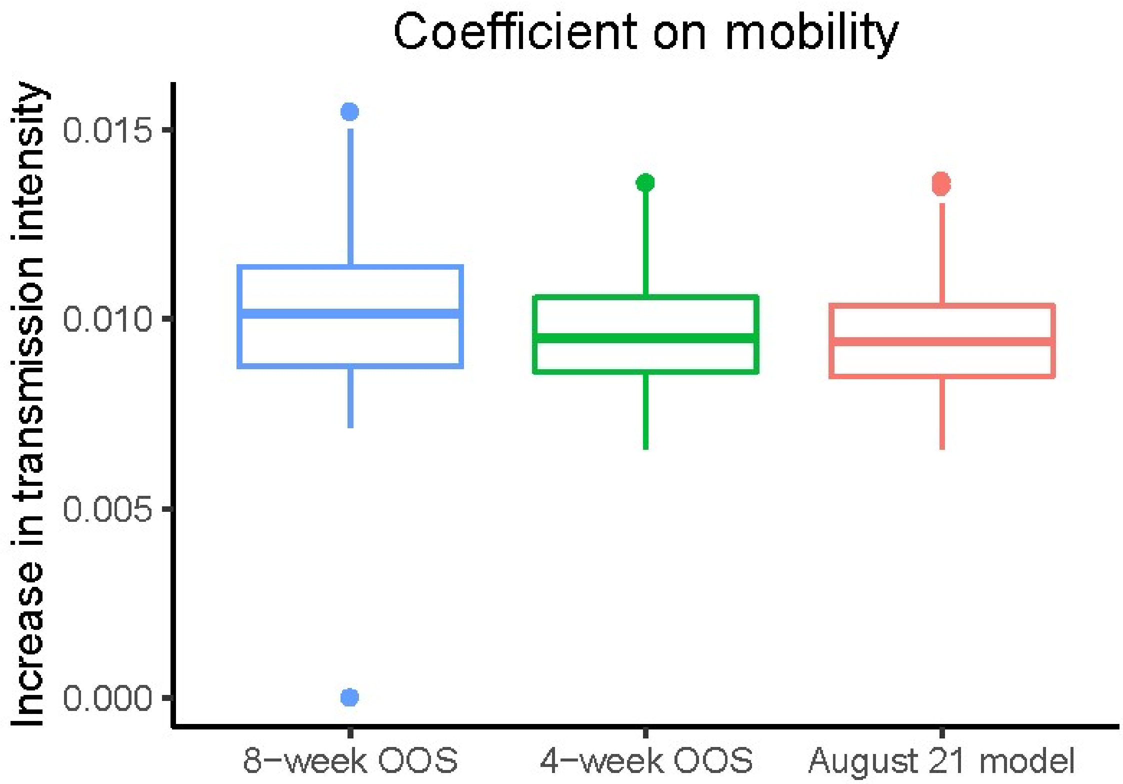

Supplementary Tables

SI Table 1. GATHER compliance

#	Checklist item	Location
**Objectives and funding**		
1	Define the indicators, populations, and time periods for which estimates were made.	Main manuscript
2	List the funding sources for the work.	Main manuscript
**Data Inputs**		
For all data inputs from multiple sources that are synthesised as part of the study:		
3	Describe how the data were identified and how the data were accessed.	SI Sections 2-3
4	Specify the inclusion and exclusion criteria. Identify all ad-hoc exclusions.	SI Sections 2-3
5	Provide information on all included data sources and their main characteristics. For each data source used, report reference information or contact name/institution, population represented, data collection method, year(s) of data collection, sex and age range, diagnostic criteria or measurement method, and sample size, as relevant.	SI Section 2, SI Tables 2-10<br><br>See data availability statement in main manuscript; SI Section 2, SI Tables 2-10
6	Identify and describe any categories of input data that have potentially important biases (e.g., based on characteristics listed in item 5).	SI Section 2, SI Tables 2-10
For data inputs that contribute to the analysis but were not synthesised as part of the study:		
7	Describe and give sources for any other data inputs.	See data availability statement in main manuscript; SI Section 2, SI Tables 2-10
For all data inputs:		

8	Provide all data inputs in a file format from which data can be efficiently extracted (e.g., a spreadsheet as opposed to a PDF), including all relevant meta-data listed in item 5. For any data inputs that cannot be shared due to ethical or legal reasons, such as third-party ownership, provide a contact name or the name of the institution that retains the right to the data.	See data availability statement in main manuscript; SI Section 2, SI Tables 2-10; data source files linked directly from the online version of the paper
**Data analysis**		
9	Provide a conceptual overview of the data analysis method. A diagram may be helpful.	Main manuscript, SI Figure 1, SI Section 2, SI Tables 2-10
10	Provide a detailed description of all steps of the analysis, including mathematical formulae. This description should cover, as relevant, data cleaning, data pre-processing, data adjustments and weighting of data sources, and mathematical or statistical model(s).	SI Sections 2, 4-7
11	Describe how candidate models were evaluated and how the final model(s) were selected.	SI Sections 2, 4-7
12	Provide the results of an evaluation of model performance, if done, as well as the results of any relevant sensitivity analysis.	SI Sections 2, 4-7
13	Describe methods for calculating uncertainty of the estimates. State which sources of uncertainty were, and were not, accounted for in the uncertainty analysis.	SI Sections 2, 4-7
14	State how analytic or statistical source code used to generate estimates can be accessed.	Code is provided in online repositories: https://github.com/ihmeuw/covid-model-seiir-pipeline<br>https://github.com/ihmeuw/covid-model-deaths-spline
**Results and Discussion**		

15	Provide published estimates in a file format from which data can be efficiently extracted.	SI Tables 2-10<br><br>Results specific to the model run for this publication are available for download (https://ihmecovid19storage.blob.core.windows.net/archive/2020-10-02/ihme-covid19.zip).
16	Report a quantitative measure of the uncertainty of the estimates (e.g. uncertainty intervals).	Main manuscript<br><br>Online data visualization tool: https://covid19.healthdata.org
17	Interpret results in light of existing evidence. If updating a previous set of estimates, describe the reasons for changes in estimates.	Main manuscript
18	Discuss limitations of the estimates. Include a discussion of any modelling assumptions or data limitations that affect interpretation of the estimates.	Main manuscript, SI Sections 4-5

SI Table 2. Sources for epidemiological data by state

Location	Source
United States of America, California	United States - California Department of Public Health Daily COVID-19 Updates 2020
United States of America, South Dakota	United States - South Dakota Department of Health Novel Coronavirus (COVID-19) Updates and Information 2020
United States of America, Texas	United States - Texas Department of State Health Service COVID-19 Fatalities Over Time by County
United States of America, West Virginia	United States - West Virginia Department of Health and Human Resources Coronavirus Disease (COVID-19) Cases 2020
United States of America, Minnesota	United States - Minnesota Department of Health Situation Update for Coronavirus Disease 2019 (COVID-19) 2020
United States of America, Texas	United States - Texas Department of State Health Service COVID-19 Cases Over Time by County
United States of America, Hawaii	United States - Hawaii Department of Health COVID-19 Current Situation 2020
United States of America, Florida	United States - Florida Division of Emergency Management COVID-19 Data Report 2020
United States of America, Maryland	United States - Maryland Department of Health COVID-19 Statistics 2020
United States of America, Alabama	United States - Alabama Department of Public Health COVID-19 Data and Surveillance 2020
United States of America, New Mexico	United States - New Mexico Department of Health 2019 Novel Coronavirus Disease (COVID-19) Updates 2020
United States of America, Nebraska	United States - Nebraska Department of Health and Human Services Coronavirus COVID-19 Cases 2020
United States of America, Delaware	United States - Delaware Division of Public Health Coronavirus Disease (COVID-19) Data Dashboard 2020
United States of America, Pennsylvania	United States - Pennsylvania Department of Health COVID-19 Cases 2020
United States of America, Maine	United States - Maine Division of Disease Surveillance Novel Coronavirus 2019 (COVID-19) Situation 2020
United States of America, New Jersey	United States - New Jersey Department of Health COVID-19 Data 2020
United States of America, Massachusetts	United States - Massachusetts Department of Public Health COVID-19 Cases, Quarantine and Monitoring 2020
United States of America, New Hampshire	United States - New Hampshire Department of Health and Human Services 2019 Novel Coronavirus (COVID-19) Summary Report 2020
United States of America, Oregon	United States - Oregon Health Authority COVID-19 Updates 2020
United States of America, Nevada	United States - Nevada Department of Health and Human Services COVID-19 (Coronavirus) Data 2020
United States of America, Connecticut	United States - Connecticut Department of Public Heath COVID-19 Update 2020
United States of America, Utah	United States - Utah Department of Health Overview of COVID-19 Surveillance 2020

Location	Source
United States of America, Colorado	United States - Colorado Department of Public Health and Environment COVID-19 Updates 2020
United States of America, Arizona	United States - Arizona Department of Health Services COVID-19 Data 2020
United States of America, Wyoming	United States - Wyoming Department of Health COVID-19 Map and Statistics 2020
United States of America, Montana	United States - Montana Department of Health and Human Services COVID-19 Cases 2020
United States of America, Oklahoma	United States - Oklahoma State Department of Health COVID-19 Current Situation 2020
United States of America, Michigan	United States - Michigan Department of Health and Human Services Coronavirus Data 2020
United States of America, Illinois	United States - Illinois Department of Public Health Coronavirus Disease 2019 (COVID-19) Statistics 2020
United States of America, Tennessee	United States - Tennessee Department of Health Epidemiology and Surveillance Data 2020
United States of America, New York City (as a subset of New York State)	United States - New York City Department of Health and Mental Hygiene Coronavirus Disease 2019 (COVID-19) Data 2020
United States of America, Georgia	United States - Georgia Department of Public Health COVID-19 Daily Status Report 2020
United States of America, Iowa	United States - Iowa Department of Public Health Novel Coronavirus (COVID-19) Cases 2020
United States of America, Wisconsin	United States - Wisconsin Department of Health Services COVID-19 Data 2020
United States of America, Louisiana	United States - Louisiana Department of Health Coronavirus (COVID-19) Information 2020
United States of America, Mississippi	United States - Mississippi State Department of Health Coronavirus Disease 2019 (COVID-19) Current Cases and Statistics 2020
United States of America, North Dakota	United States - North Dakota Department of Health Coronavirus Cases 2020
United States of America, Arkansas	United States - Arkansas Department of Health COVID-19 Status Updates 2020
United States of America, Idaho	United States - Idaho Division of Public Health COVID-19 Case Data 2020
United States of America, North Carolina	United States - North Carolina Department of Health and Human Services COVID-19 Dashboard 2020
United States of America, Missouri	United States - Missouri Department of Health and Senior Services COVID-19 Outbreak Data 2020
United States of America, Alaska	United States - Alaska Department of Public Health and Social Services Coronavirus Response Hub 2020
United States of America, South Carolina	United States - South Carolina Department of Health and Environmental Control COVID-19 Demographic Data by Case 2020

Location	Source
United States of America, Virginia	United States - Virginia Department of Health COVID-19 Cases 2020
United States of America, Ohio	United States - Ohio Department of Health COVID-19 Dashboard 2020
United States of America, Rhode Island	United States - Rhode Island Department of Health COVID-19 Data Tracker 2020
United States of America, Vermont	United States - Vermont Department of Health COVID-19 Data 2020
United States of America, Indiana	United States - Indiana COVID-19 Statewide Test, Case, and Death Trends 2020
United States of America, Kansas	United States - Kansas Department of Health and Environment Coronavirus Disease 2019 (COVID-19) Case Summary 2020
United States of America, Kentucky	United States - Kentucky Department for Public Health COVID-19 Dashboard 2020
United States of America, Washington	United States - Washington State Department of Health COVID-19 Cases by County and CDC Event Date 2020
United States of America, Washington	United States - Washington State Department of Health COVID-19 Deaths by County, Date, Age Group and Sex 2020

SI Table 3. Alternate data repositories for state-level reporting

Location	Source
Illinois	United States - Illinois Department of Public Health Coronavirus Disease 2019 (COVID-19) Statistics 2020
Maryland	United States - Maryland Department of Health COVID-19 Statistics 2020
Kentucky	United States - Kentucky Department for Public Health COVID-19 Dashboard 2020
Hawaii	United States - Hawaii Department of Health COVID-19 Current Situation 2020
Nebraska	United States - Nebraska Department of Health and Human Services Coronavirus COVID-19 Cases 2020
North Carolina	United States - North Carolina Department of Health and Human Services COVID-19 Dashboard 2020
Indiana	United States - Indiana COVID-19 Statewide Test, Case, and Death Trends 2020
Washington	United States - Washington State Department of Health COVID-19 Cases by County and CDC Event Date 2020; United States - Washington State Department of Health COVID-19 Deaths by County, Date, Age Group and Sex 2020
New York City (as a subset of New York State)	United States - New York City Department of Health and Mental Hygiene Coronavirus Disease 2019 (COVID-19) Data 2020

SI Table 4. Ad hoc corrections made to the Johns Hopkins dataset

A file containing SI Table 4 is hosted within the Global Health Data Exchange record (file name – IHME_USA_COVID_2020_2021_MANDATES_CLOSURES_REOPENING_Y2020M10D09.CSV) associated with this publication http://ghdx.healthdata.org/record/ihme-data/united-states-covid-19-scenarios-2020-2021

SI Table 5. Data sources by state for COVID-19-specific hospitalisations

Location	Data source
United States of America, Alaska	United States - Alaska Department of Public Health and Social Services Coronavirus Response Hub 2020
United States of America	The COVID Tracking Project

SI Table 6. Covariates summary table

	Covariate Name	Definition	Covariate Coefficient (baseline model)
Time varying	Social distancing mandates	Categorical classification of social distancing mandates imposition and lifting	N/A
	Mobility	Composite indicator of multiple mobility sources, expressed as percent reduction from "norm". Projected based on social distancing mandates	9.45E-3 (95% UI: 7.10E-3 to 1.21E-2)
	Testing per capita	Number of tests administered daily divided by population per location	-25.9 (-54.6 to -6.37)
	Mask Use	Percentage of the population who "always" wear a mask	-0.502 (-0.520 to -0.380)
	Pneumonia seasonality	Proportion of pneumonia deaths to total deaths by week of the calendar year; by location	1.035 (0.90 to 1.31)
Time invariant	LRI mortality	age 15+ age-standardised LRI mortality rate by location (time invariant, based on 2019 results)	5.89E-4 (0.0 to 4.65E-3)
	Altitude	Proportion population below 100 m altitude	1.38E-3 (0.0 to 8.97E-3)
	Population density	Percentage of population living in areas more dense than 1,000 ppl per square km	2.5E-6 (0.0 to 4.6E-6)
	Smoking	Smoking exposure per capita (time invariant) (Adult age-standardised [15+ years], both sexes)	1.38E-2 (0.0 to 0.102)
	Air pollution	Population-weighted annual mean PM2.5 exposure	2.00E-5 (0.0 to 1.38E-4)

SI Table 7. Date of enactment, repeal, and source by state for social distancing mandates

A file containing SI Table 7 is hosted within the Global Health Data Exchange record (file name – IHME_USA_COVID_2020_2021_RAW_DATA_CORRECTIONS_Y2020M10D09.CSV) associated with this publication http://ghdx.healthdata.org/record/ihme-data/united-states-covid-19-scenarios-2020-2021

SI Table 8. Listing of source of testing data for locations not present within Our World in Data global dataset

Location	Source	Tests reported
Albania	Albania Ministry of Health and Social Protection Updated Information on COVID-19 2020	Individuals tested
Angola	Angola Ministry of Health COVID-19 News 2020	Tests processed
Armenia	Armenia National Center for Disease Control Coronavirus Disease (COVID-19) Confirmed Cases by Days 2020	Cases + Negatives
Australia	Coronavirus (COVID-19) in Australia 2020	Tests processed
Barbados	Barbados Government Information Service COVID-19 Update 2020	Tests processed
Benin	Benin Coronavirus Information (COVID-19) 2020	Tests processed
Botswana	Botswana COVID-19 Updates 2020	Tests processed
Canadian subnationals	Canada Coronavirus Disease 2019 (COVID-19) Daily Epidemiology Update 2020	Individuals tested
Canadian subnationals	Canada Public Health Infobase Number of Total Cases of COVID-19 2020	Individuals tested
Comoros	Comoros Ministry of Health, Solidarity, Social Protection and Gender Promotion COVID-19 Press Release	Tests processed
Congo	Congo COVID-19 Epidemiological Situation 2020	Tests processed
Costa Rica	Costa Rica COVID-19 National Situation - Distance State University 2020	Tests processed
Cote d'Ivoire	Cote d'Ivoire COVID-19: Update on the Situation of Coronavirus Disease 2020	Tests processed
Cyprus	Cyprus Announcement of the Ministry of Health Regarding New Cases of COVID-19 Disease 2020	Tests processed
Djibouti	Djibouti COVID-19 Statistics 2020	Tests processed
Dominican Republic	Dominican Republic General Directorate of Epidemiology Coronavirus Disease 2019 (COVID-19) Special Bulletin 2020	Tests processed
Democratic Republic of the Congo	Democratic Republic of the Congo Multisectoral Committee on the Response to COVID-19 Bulletin 2020	Tests processed

Location	Source	Tests reported
Equatorial Guinea	Equatorial Guinea COVID-19 News - AhoraEG	Tests processed
Eswatini	Eswatini COVID-19 Dashboard 2020	Individuals tested
Gabon	Gabon COVID-19 Epidemiological Situation 2020	Tests processed
Gambia	Gambia COVID-19 Situational Outbreak Report 2020	Tests processed
Ghana	Ghana Health Service Coronavirus Disease (COVID-19) Updates 2020	Tests processed
Guinea	Guinea Ministry of Health COVID-19 Epidemiological Situation 2020	Individuals tested
Guinea Bissau	Guinea-Bissau INFOCOVID-19 Update 2020	Tests processed
Guyana	Guyana Ministry of Public Health COVID-19 Dashboard 2020	Individuals tested
Honduras	Honduras National Risk Management System COVID-19 Statement 2020	Tests processed
Italian subnationals	Italy COVID-19 Situation Monitoring - Department of Civil Protection	Tests processed
Japan	Japan Coronavirus Disease (COVID-19) Situation Report 2020 - Toyo Kazei Online	Tests processed
Madagascar	Madagascar Ministry of Public Health Coronavirus Situation 2020	Tests processed
Mali	Mali Ministry of Health and Social Affairs Communique on the Monitoring of Prevention and Response Actions to Coronavirus Disease 2020	Tests processed
Mauritania	Mauritania COVID-19 Situation Report 2020	Tests processed
Mauritius	Mauritius Ministry of Health and Wellness COVID-19 Statistics 2020	Tests processed
Mexico	Mexico General Directorate of Epidemiology COVID-19 Database 2020	Individuals tested
Moldova	Moldova Epidemiological Situation Due to Infection with the New Type of Coronavirus (COVID-19) 2020	Tests processed
Mozambique	Mozambique National Institute of Health COVID-19 Daily Surveillance Bulletin 2020	Individuals tested

Location	Source	Tests reported
Niger	Niger Ministry of Public Health General Secretariat COVID-19 Communications 2020	Tests processed
Pakistan - Sindh	Pakistan - Sindh COVID-19 Statistics 2020	Tests processed
Pakistan - Punjab	Pakistan - Punjab COVID-19 Statistics 2020	Tests processed
Pakistan - Khyber Pakhtunkhwa	Pakistan - Khyber Pakhtunkhwa COVID-19 Statistics 2020	Tests processed
Pakistan - Islamabad	Pakistan - Islamabad COVID-19 Statistics 2020	Tests processed
Pakistan - Gilgit-Baltistan	Pakistan - Gilgit-Baltistan COVID-19 Statistics 2020	Tests processed
Pakistan - Balochistan	Pakistan - Balochistan COVID-19 Statistics 2020	Tests processed
Pakistan - Azad Jammu and Kashmir	Pakistan - Azad Jammu and Kashmir COVID-19 Statistics 2020	Tests processed
Brazil - Pernambuco	COVID-19 in the World, in Brazil and in Pernambuco 2020	Tests processed
Peru	Peru Ministry of Health COVID-19 Situation 2020	Individuals tested
Saint Kitts	Saint Kitts and Nevis COVID-19 Situation Report 2020	Positives + Negatives
Brazil - Santa Catarina	Brazil - Santa Catarina Coronavirus Epidemiological Bulletin 2020	Tests processed
Brazil - Sergipe	Brazil - Sergipe Epidemiological Bulletin for Update on Coronavirus Disease 2019 (Covid-19) 2020	Tests processed
Sierra Leone	Sierra Leone Coronavirus Disease (COVID-19) Situational Report, April-May 2020	Tests processed
South Africa subnationals	South Africa National Institute for Communicable Diseases COVID-19 Weekly Epidemiological Brief 2020	Tests processed

Location	Source	Tests reported
Spain - Aragon	Spain - Aragon Open Data: Daily Facts and Figures About the Coronavirus 2020	Tests processed
Spain - Cantabria	Spain - Cantabria Epidemiological Situation of COVID-19 2020	Total PCR tests
Spain - Navarra	Spain - Navarra COVID-19 Tests Results Data 2020	Total PCR + Antibody Tests
Spain - Navarra	Spain - Navarra New COVID-19 Series Evolution Data 2020	Total PCR + Antibody Tests
Spain - Castile y Leon	Spain - Castile and León Open Data: Coronavirus Tests 2020	Total PCR tests
Spain	Spain Ministry of Health, Consumption, and Social Welfare Coronavirus Disease (COVID-19) Current Situation Update 2020	Total PCR tests
Togo	Coronavirus in Togo: Evolution in Graphics 2020	Tests processed
United States of America States (apart from Washington)	United States COVID Tracking Project API - Historic State Data 2020	Various
Washington	United States - Washington State Department of Health COVID-19 Tests by County and Specimen Collection Date 2020	Tests processed
Yemen	Yemen Covid-19 Daily Report for the Period from January to June 2020	Tests processed
Spain - La Rioja	Spain - La Rioja Epidemiological Situation of COVID-19 2020	Tests processed
Spain - Balearic Islands	Spain - Balearic Islands Ministry of Health and Consumption News About the Coronavirus COVID-19 2020	Tests processed
Spain - Asturias	Spain - Asturias Open Data: COVID-19 Evolution 2020	Tests processed
Georgia	Georgia National Center for Disease Control and Public Health COVID-19 Update 2020	Tests processed
Malawi	Malawi COVID-19 National Information Dashboard 2020	Tests processed

Location	Source	Tests reported
Cape Verde	Cape Verde COVID-19 Epidemiological Bulletin 2020	Tests processed
Namibia	Namibia COVID-19 National Statistics 2020	Individuals tested
Sudan	Sudan Health Observatory COVID-19 Situation and Updates 2020	Tests processed
Central African Republic	Central African Republic COVID-19 Daily Situation Report 2020	Individuals tested
Philippines	Philippines Department of Health COVID-19 Tracker 2020	Tests processed
Niger	Niger Ministry of Public Health General Secretariat COVID-19 Communications 2020	Tests processed
Brail - Minas Gerais	Brazil - Minas Gerais Coronavirus Epidemiological Bulletin 2020	Tests processed in public facilities
Brail - Rondonia	Brazil - Rondonia Daily Newsletter on Coronavirus 2020	Tests processed
Jamaica	Jamaica Ministry of Health and Wellness COVID-19 Update 2020	Tests processed
Jordan	Jordan Ministry of Health COVID-19 Updates 2020	Tests processed
Zambia	Zambia Coronavirus Disease (COVID-19) Outbreak Situation Report 2020	Tests processed
Indian subnationals	India COVID-19 Crowdsourced Patient Database: State Level Testing Data 2020	Various
Palestine	Palestine Ministry of Health COVID-19 Surveillance System 2020	Tests processed
Bermuda	Bermuda COVID-19 Update 2020	Tests processed
Lebanon	Lebanon Ministry of Public Health COVID-19 Surveillance Data 2020	Tests processed
Oman	Oman Ministry of Health COVID-19 Statement 2020	Tests processed
Guatemala	Guatemala Ministry of Public Health and Social Assistance COVID-19 Case Update 2020	Tests processed
Palau	Palau Ministry of Health Coronavirus Disease 2019 (COVID-19) Situation Report 2020	Tests processed

SI Table 9. Infection fatality ratio data sources

Location	Date	Source
Belgium	13th June 2020	Sereina et al. 2020 "Seroprevalence of IgG antibodies against SARS coronavirus 2 in Belgium: a serial prospective cross-sectional nationwide study of residual samples" medRxiv https://www.medrxiv.org/content/10.1101/2020.06.08.20125179v3
Belgium	4th July 2020	Sereina et al. 2020 "Seroprevalence of IgG antibodies against SARS coronavirus 2 in Belgium: a serial prospective cross-sectional nationwide study of residual samples" medRxiv https://www.medrxiv.org/content/10.1101/2020.06.08.20125179v3
Denmark	17th April 2020	Erikstrup et al. 2020 "Estimation of SARS-CoV-2 infection fatality rate by real-tme antibody screening of blood donors" medRxiv https://www.medrxiv.org/content/10.1101/2020.04.24.20075291v1.full.pdf
Spain	11th May 2020	Estudio ENE-COVID19 Segunda Ronda https://www.ciencia.gob.es/stfls/MICINN/Ministerio/FICHEROS/INFORME_SEGUNDA_RONDA.pdf
Spain	1st June 2020	Estudio ENE-COVID19 Segunda Ronda https://www.ciencia.gob.es/stfls/MICINN/Ministerio/FICHEROS/INFORME_SEGUNDA_RONDA.pdf
Spain	22nd June 2020	Estudio ENE-COVID19 Informe Final https://portalcne.isciii.es/enecovid19/informes/informe_final.pdf
Sweden	2nd May 2020	Folkhalsomyndigheten "Pavisning av antikroppar efter genomgangen covid-19 hos blodgivare (Delrapport 2)" https://www.folkhalsomyndigheten.se/publicerat-material/publikationsarkiv/p/pavisning-av-antikroppar-efter-genomgangen-covid-19-hos-blodgivare-delrapport-2/
Sweden	9th May 2020	Folkhalsomyndigheten "Pavisning av antikroppar efter genomgangen covid-19 hos blodgivare (Delrapport 2)" https://www.folkhalsomyndigheten.se/publicerat-material/publikationsarkiv/p/pavisning-av-antikroppar-efter-genomgangen-covid-19-hos-blodgivare-delrapport-2/
Sweden	16th May 2020	Folkhalsomyndigheten "Pavisning av antikroppar efter genomgangen covid-19 hos blodgivare (Delrapport 2)" https://www.folkhalsomyndigheten.se/publicerat-material/publikationsarkiv/p/pavisning-av-antikroppar-efter-genomgangen-covid-19-hos-blodgivare-delrapport-2/

Sweden	23rd May 2020	Folkhalsomyndigheten "Pavisning av antikroppar efter genomgangen covid-19 hos blodgivare (Delrapport 2)" https://www.folkhalsomyndigheten.se/publicerat-material/publikationsarkiv/p/pavisning-av-antikroppar-efter-genomgangen-covid-19-hos-blodgivare-delrapport-2/
Brazil	21st May 2020	Hallal et al. 2020 "Remarkable variability in SARS-CoV-2 antibodies across Brazilian regions: nationwide serological household survey in 27 states" medRxiv https://www.medrxiv.org/content/10.1101/2020.05.30.20117531v1.full.pdf
Kenya	16th June 2020	Uyoga et al. 2020 "Seroprevalence of anti-SARS-CoV-2 IgG antibodies in Kenyan blood donors" medRxiv https://www.medrxiv.org/content/10.1101/2020.07.27.20162693v1
Nigeria	30th June 2020	Majiya et al. 2020 "Seroprevalence of COVID-19 in Niger State" medRxiv https://www.medrxiv.org/content/10.1101/2020.08.04.20168112v1
California, United States	27th April	Havers et al. 2020 "Seroprevalence of Antibodies to SARS-CoV-2 in 10 Sites in the United States, March 23 – May 12 2020" JAMA Intern Med. https://jamanetwork.com/journals/jamainternalmedicine/fullarticle/2768834?guestAccessKey=7a5c32e6-3c27-41b3-b46c-43c4a38bbe00
Connecticut, United States	3rd May 2020	Havers et al. 2020 "Seroprevalence of Antibodies to SARS-CoV-2 in 10 Sites in the United States, March 23 – May 12 2020" JAMA Intern Med. https://jamanetwork.com/journals/jamainternalmedicine/fullarticle/2768834?guestAccessKey=7a5c32e6-3c27-41b3-b46c-43c4a38bbe00
Connecticut, United States	26th May 2020	US Centers for Disease Control "Commerical Laboratory Seroprevalence Survey Data" https://covid.cdc.gov/covid-data-tracker/#serology-surveillance
Connecticut, United States	17th June 2020	US Centers for Disease Control "Commerical Laboratory Seroprevalence Survey Data" https://covid.cdc.gov/covid-data-tracker/#serology-surveillance
Florida, United States	10th April 2020	Havers et al. 2020 "Seroprevalence of Antibodies to SARS-CoV-2 in 10 Sites in the United States, March 23 – May 12 2020" JAMA Intern Med. https://jamanetwork.com/journals/jamainternalmedicine/fullarticle/2768834?guestAccessKey=7a5c32e6-3c27-41b3-b46c-43c4a38bbe00

Florida, United States	24th April 2020	US Centers for Disease Control "Commerical Laboratory Seroprevalence Survey Data" https://covid.cdc.gov/covid-data-tracker/#serology-surveillance
Louisiana, United States	8th April 2020	Havers et al. 2020 "Seroprevalence of Antibodies to SARS-CoV-2 in 10 Sites in the United States, March 23 – May 12 2020" JAMA Intern Med. https://jamanetwork.com/journals/jamainternalmedicine/fullarticle/2768834?guestAccessKey=7a5c32e6-3c27-41b3-b46c-43c4a38bbe00
Minnesota, United States	12th May 2020	Havers et al. 2020 "Seroprevalence of Antibodies to SARS-CoV-2 in 10 Sites in the United States, March 23 – May 12 2020" JAMA Intern Med. https://jamanetwork.com/journals/jamainternalmedicine/fullarticle/2768834?guestAccessKey=7a5c32e6-3c27-41b3-b46c-43c4a38bbe00
Minnesota, United States	7th June 2020	US Centers for Disease Control "Commerical Laboratory Seroprevalence Survey Data" https://covid.cdc.gov/covid-data-tracker/#serology-surveillance
Minnesota, United States	27th June 2020	US Centers for Disease Control "Commerical Laboratory Seroprevalence Survey Data" https://covid.cdc.gov/covid-data-tracker/#serology-surveillance
Missouri, United States	26th April 2020	Havers et al. 2020 "Seroprevalence of Antibodies to SARS-CoV-2 in 10 Sites in the United States, March 23 – May 12 2020" JAMA Intern Med. https://jamanetwork.com/journals/jamainternalmedicine/fullarticle/2768834?guestAccessKey=7a5c32e6-3c27-41b3-b46c-43c4a38bbe00
Missouri, United States	30th March 2020	US Centers for Disease Control "Commerical Laboratory Seroprevalence Survey Data" https://covid.cdc.gov/covid-data-tracker/#serology-surveillance
Missouri, United States	20th June 2020	US Centers for Disease Control "Commerical Laboratory Seroprevalence Survey Data" https://covid.cdc.gov/covid-data-tracker/#serology-surveillance
New York, United States	1st April 2020	Havers et al. 2020 "Seroprevalence of Antibodies to SARS-CoV-2 in 10 Sites in the United States, March 23 – May 12 2020" JAMA Intern Med. https://jamanetwork.com/journals/jamainternalmedicine/fullarticle/2768834?guestAccessKey=7a5c32e6-3c27-41b3-b46c-43c4a38bbe00
New York, United States	28th April 2020	Rosenberg et al. 2020 "Cumulative incidence and diagnosis of SARS-CoV-2 Infection in New York medRxiv https://www.medrxiv.org/content/10.1101/2020.05.25.20113050v1.full.pdf

New York, United States	6th May 2020	US Centers for Disease Control "Commerical Laboratory Seroprevalence Survey Data" https://covid.cdc.gov/covid-data-tracker/#serology-surveillance
New York, United States	21st June 2020	US Centers for Disease Control "Commerical Laboratory Seroprevalence Survey Data" https://covid.cdc.gov/covid-data-tracker/#serology-surveillance
Pennsylvania, United States	25th April 2020	Havers et al. 2020 "Seroprevalence of Antibodies to SARS-CoV-2 in 10 Sites in the United States, March 23 – May 12 2020" JAMA Intern Med. https://jamanetwork.com/journals/jamainternalmedicine/fullarticle/2768834?guestAccessKey=7a5c32e6-3c27-41b3-b46c-43c4a38bbe00
Pennsylvania, United States	30th May 2020	US Centers for Disease Control "Commerical Laboratory Seroprevalence Survey Data" https://covid.cdc.gov/covid-data-tracker/#serology-surveillance
Pennsylvania, United States	20th June 2020	US Centers for Disease Control "Commerical Laboratory Seroprevalence Survey Data" https://covid.cdc.gov/covid-data-tracker/#serology-surveillance
Utah, United States	3rd May 2020	Havers et al. 2020 "Seroprevalence of Antibodies to SARS-CoV-2 in 10 Sites in the United States, March 23 – May 12 2020" JAMA Intern Med. https://jamanetwork.com/journals/jamainternalmedicine/fullarticle/2768834?guestAccessKey=7a5c32e6-3c27-41b3-b46c-43c4a38bbe00
Utah, United States	5th June 2020	US Centers for Disease Control "Commerical Laboratory Seroprevalence Survey Data" https://covid.cdc.gov/covid-data-tracker/#serology-surveillance
Washington, United States	1st April 2020	Havers et al. 2020 "Seroprevalence of Antibodies to SARS-CoV-2 in 10 Sites in the United States, March 23 – May 12 2020" JAMA Intern Med. https://jamanetwork.com/journals/jamainternalmedicine/fullarticle/2768834?guestAccessKey=7a5c32e6-3c27-41b3-b46c-43c4a38bbe00

SI Table 10. Mortality rate estimation data sources

Location	Date	Source
China	11th February 2020	"The Epidemiological Characteristics of an Outbreak of 2019 Novel Coronavirus Diseases (COVID-19) - China 2020" http://weekly.chinacdc.cn/en/article/id/e53946e2-c6c4-41e9-9a9b-fea8db1a8f51
Indonesia	17th August 2020	Komite Penanganan COVID-19 Dan Pemulihan Ekonomi Nasional https://covid19.go.id/peta-sebaran
Philippines	19th August 2020	Philippines Department of Health https://www.doh.gov.ph/2019-nCoV
Japan	8th May 2020	Ministry of Health, Labour and Welfare https://www.mhlw.go.jp/stf/houdou/houdou_list_202005.html
Republic of Korea	18th August 2020	KDCA https://www.cdc.go.kr/board/board.es?mid=a30402000000&bid=0030
Singapore	6th May 2020	Ministry of Health https://www.moh.gov.sg/news-highlights
Australia	18th August 2020	Department of Health https://www.health.gov.au/news/health-alerts/novel-coronavirus-2019-ncov-health-alert
New Zealand	19th August 2020	Ministry of Health https://www.health.govt.nz/our-work/diseases-and-conditions/covid-19-novel-coronavirus/covid-19-current-situation/covid-19-current-cases
Belgium	19th August 2020	Sciensano https://epistat.wiv-isp.be/covid/
Denmark	18th August 2020	Statens Serum Institut https://www.ssi.dk/sygdomme-beredskab-og-forskning/sygdomsovervaagning/c/covid19-overvaagning
France	19th August 2020	Sante Publique France https://geodes.santepubliquefrance.fr/#c=indicator&f=0&i=covid_hospit.dc&s=2020-03-26&t=a01&view=map2
Germany	19th August 2020	Robert Koch Institut https://www.rki.de/DE/Content/InfAZ/N/Neuartiges_Coronavirus/Situationsberichte/Gesamt.html
Greece	19th August 2020	EODY Greece https://eody.gov.gr/neos-koronaios-covid-19/
Iceland	18th June 2020	Directorate of Health https://www.covid.is/data

Ireland	19th August 2020	Health Protection Surveillance Centre https://www.hpsc.ie/a-z/respiratory/coronavirus/novelcoronavirus/casesinireland/epidemiologyofcovid-19inireland/
Italy	11th August 2020	Istituto Superiore do Sanita https://www.epicentro.iss.it/coronavirus/aggiornamenti
Netherlands	19th August 2020	RIVM https://www.rivm.nl/coronavirus-covid-19/grafieken
Norway	19th August 2020	NIPH https://www.fhi.no/en/id/infectious-diseases/coronavirus/daily-reports/daily-reports-COVID19/
Portugal	11th August 2020	Direcao-Geral de Saude https://covid19.min-saude.pt/
Spain	13th August 2020	ISCII https://www.isciii.es/QueHacemos/Servicios/VigilanciaSaludPublicaRENAVE/EnfermedadesTransmisibles/Paginas/InformesCOVID-19.aspx
Sweden	19th August 2020	Folkhalsomyndigheten https://www.folkhalsomyndigheten.se/smittskydd-beredskap/utbrott/aktuella-utbrott/covid-19/statistik-och-analyser/bekraftade-fall-i-sverige/
Switzerland	11th August 2020	Federal Office of Public Health https://www.bag.admin.ch/bag/en/home/krankheiten/ausbrueche-epidemien-pandemien/aktuelle-ausbrueche-epidemien/novel-cov/situation-schweiz-und-international.html
United Kingdom	7th August 2020	Office for National Statistics https://www.ons.gov.uk/peoplepopulationandcommunity/birthsdeathsandmarriages/deaths/datasets/weeklyprovisionalfiguresondeathsregisteredinenglandandwales
Argentina	28th July 2020	Ministerio de Salud https://www.argentina.gob.ar/salud/coronavirus-COVID-19
Chile	29th July 2020	MINSA https://www.minsal.cl/nuevo-coronavirus-2019-ncov/casos-confirmados-en-chile-covid-19/
Canada	19th August 2020	Government of Canada https://health-infobase.canada.ca/covid-19/epidemiological-summary-covid-19-cases.html

United States of America	24th June 2020	National Center for Health Statistics https://www.cdc.gov/nchs/nvss/vsrr/covid_weekly/index.htm
Haiti	17th August 2020	Ministere de la Sante Publique et de la Population https://www.mspp.gouv.ht/documentation/
Ecuador	3rd May 2020	Ministerio de Salud Publica https://www.salud.gob.ec/boletines-epidemiologicos-coronavirus-por-semanas/
Peru	11th August 2020	MINSA - https://www.dge.gob.pe/portalnuevo/covid-19/covid-cajas/situacion-del-covid-19-en-el-peru/
Colombia	14th August 2020	Instituto Nacional de Salud https://www.ins.gov.co/Paginas/Boletines-casos-COVID-19-Colombia.aspx#InplviewHash5872a312-02d0-4090-aa8a-7716dd9fc4df=Paged%3DTRUE-p_SortBehavior%3D0-p_FileLeafRef%3D2020%252d06%252d11%252exlsx-p_ID%3D116-PageFirstRow%3D91
Costa Rica	18th August 2020	Observatorio Geografico en Salud http://geovision.uned.ac.cr/oges/index.html#descargas
Guatemala	18th August 2020	MSPAS https://tablerocovid.mspas.gob.gt/
Mexico	19th August 2020	Gobierno de Mexico https://www.gob.mx/salud/documentos/lineamiento-estandarizado-para-la-vigilancia-epidemiologica-y-por-laboratorio-de-la-enfermedad-respiratoria-viral
Iraq	19th August 2020	World Health Organization https://app.powerbi.com/view?r=eyJrIjoiNjljMDhiYmItZTlhMS00MDlhLTg3MjItMDNmM2FhNzE5NmM4IiwidCI6ImY2MTBjMGI3LWJkMjQtNGIzOS04MTBiLTNkYzI4MGFmYjU5MCIsImMiOjh9
Lebanon	18th August 2020	MOPH - https://www.moph.gov.lb/ar/Pages/2/24870/%D9%81%D9%8A%D8%B1%D9%88%D8%B3-%D8%A7%D9%84%D8%AA%D8%A7%D8%AC%D9%8A-%D8%A7%D9%84%D9%85%D8%B3%D8%AA%D8%AC%D8%AF-2019
Afghanistan	18th August 2020	Ministry of Public Health http://covid.moph-dw.org/#/
Bangladesh	19th August 2020	IEDCR https://covid19bd.idare.io/

Republic of the Congo	28th July 2020	Ministere de la Sante, de la Population, de la Promotion de la Femme et de l’Integration de la Femme au Developpement http://sante.gouv.cg/
Kenya	27th July 2020	Ministry of Health https://www.health.go.ke/#1591180376422-52af4c1e-256b
Somalia	12th August 2020	WHO Somalia https://bmgf.maps.arcgis.com/apps/opsdashboard/index.html#/d0d9a939c5fa401caa3a7447e72b2017
South Africa	1st July 2020	Department of Health https://sacoronavirus.co.za/
Eswatini	19th August 2020	Wits University https://datastudio.google.com/embed/u/0/reporting/b847a713-0793-40ce-8196-e37d1cc9d720/page/2a0LB
Togo	19th August 2020	Gouvernement Togo https://covid19.gouv.tg/graph-evolution/
Gauteng, South Africa	23rd July 2020	Gauteng Health https://twitter.com/gautenghealth?lang=en
Western Cape, South Africa	19th August 2020	Western Cape Government https://coronavirus.westerncape.gov.za/covid-19-dashboard
Alabama, United States	18th August 2020	Alabama Public Health https://alpublichealth.maps.arcgis.com/apps/opsdashboard/index.html#/6d2771faa9da4a2786a509d82c8cf0f7
Alaska, United States	18th August 2020	Alaska Department of Health and Social Services https://coronavirus-response-alaska-dhss.hub.arcgis.com
Arizona, United States	25th August 2020	Arizona Department of Health Services https://www.azdhs.gov/preparedness/epidemiology-disease-control/infectious-disease-epidemiology/covid-19/dashboards/index.php
Arkansas, United States	8th August 2020	National Center for Health Statistics https://www.cdc.gov/nchs/nvss/vsrr/covid_weekly/index.htm
California, United States	8th August 2020	National Center for Health Statistics https://www.cdc.gov/nchs/nvss/vsrr/covid_weekly/index.htm
Colorado, United States	19th August 2020	Colorado Department of Health https://drive.google.com/drive/folders/1bBAC7H-pdEDgPxRuU_eR36ghzc0HWNf1

Connecticut, United States	17th August 2020	Ct Data https://data.ct.gov/Health-and-Human-Services/COVID-19-Cases-and-Deaths-by-Age-Group/ypz6-8qyf
Delaware, United States	24th August 2020	Delaware Environmental Public Health Tracking Network https://myhealthycommunity.dhss.delaware.gov/locations/state
District of Columbia, United States	8th August 2020	National Center for Health Statistics https://www.cdc.gov/nchs/nvss/vsrr/covid_weekly/index.htm
Florida, United States	17th August 2020	Florida Division of Disaster Management https://www.floridadisaster.org/covid19/covid-19-data-reports/
Georgia, United States	25th August 2020	Georgia Department of Public Health https://dph.georgia.gov/covid-19-daily-status-report
Idaho, United States	17th August 2020	Idaho Department of Health and Welfare https://public.tableau.com/profile/idaho.division.of.public.health#!/vizhome/DPHIdahoCOVID-19Dashboard/Home
Illinois, United States	25th August 2020	Illinois Department of Public Health http://www.dph.illinois.gov/covid19/covid19-statistics
Indiana, United States	17th August 2020	Indiana COVID-19 Data Report https://www.coronavirus.in.gov/2393.htm
Iowa, United States	8th August 2020	National Center for Health Statistics https://www.cdc.gov/nchs/nvss/vsrr/covid_weekly/index.htm
Kansas, United States	8th August 2020	National Center for Health Statistics https://www.cdc.gov/nchs/nvss/vsrr/covid_weekly/index.htm
Kentucky, United States	24th July 2020	Kentucky Department of Public Health https://kygeonet.maps.arcgis.com/apps/opsdashboard/index.html#/543ac64bc40445918cf8bc34dc40e334
Louisiana, United States	18th August 2020	Louisiana Department of Health https://ldh.la.gov/Coronavirus/
Maine, United States	8th August 2020	National Center for Health Statistics https://www.cdc.gov/nchs/nvss/vsrr/covid_weekly/index.htm
Maryland, United States	18th August 2020	Maryland Department of Health https://coronavirus.maryland.gov/

Massachusetts, United States	11th August 2020	Government of Massachusetts https://www.mass.gov/info-details/covid-19-response-reporting
Michigan, United States	17th August 2020	Michigan Department of Health and Human Services https://www.michigan.gov/coronavirus/0,9753,7-406-98163_98173---,00.html
Minnesota, United States	8th August 2020	Department of Health https://www.health.state.mn.us/diseases/coronavirus/stats/index.html
Mississippi, United States	17th August 2020	Mississippi State Department of Health https://msdh.ms.gov/msdhsite/_static/14,0,420.html
Missouri, United States	25th August 2020	Missouri Department of Health https://showmestrong.mo.gov/data/public-health/
Nebraska, United States	8th August 2020	National Center for Health Statistics https://www.cdc.gov/nchs/nvss/vsrr/covid_weekly/index.htm
Nevada, United States	8th August 2020	National Center for Health Statistics https://www.cdc.gov/nchs/nvss/vsrr/covid_weekly/index.htm
New Hampshire, United States	18th August 2020	New Hampshire Department of Health and Human Services https://www.nh.gov/covid19/
New Jersey, United States	30th July 2020	National Center for Health Statistics https://www.cdc.gov/nchs/nvss/vsrr/covid_weekly/index.htm
New Mexico, United States	8th August 2020	National Center for Health Statistics https://www.cdc.gov/nchs/nvss/vsrr/covid_weekly/index.htm
New York, United States	17th August 2020	New York State Department of Health https://covid19tracker.health.ny.gov/views/NYS-COVID19-Tracker/NYSDOHCOVID-19Tracker-Map?%3Aembed=yes&%3Atoolbar=no&%3Atabs=n
North Carolina, United States	18th August 2020	North Carolina Department of Health and Human Services https://covid19.ncdhhs.gov/dashboard
North Dakota, United States	8th August 2020	National Center for Health Statistics https://www.cdc.gov/nchs/nvss/vsrr/covid_weekly/index.htm

Ohio, United States	24th August 2020	Ohio Department of Health https://coronavirus.ohio.gov/wps/portal/gov/covid-19/dashboards
Oklahoma, United States	17th August 2020	Oklahoma State Department of Health https://coronavirus.health.ok.gov/
Oregon, United States	18th August 2020	Oregon Health Authority https://govstatus.egov.com/OR-OHA-COVID-19
Pennsylvania, United States	8th August 2020	National Center for Health Statistics https://www.cdc.gov/nchs/nvss/vsrr/covid_weekly/index.htm
Rhode Island, United States	8th August 2020	National Center for Health Statistics https://www.cdc.gov/nchs/nvss/vsrr/covid_weekly/index.htm
South Carolina, United States	18th August 2020	South Carolina Department of Health and Environmental Control https://scdhec.gov/infectious-diseases/viruses/coronavirus-disease-2019-covid-19/south-carolina-county-level-data-covid-19
South Dakota, United States	8th August 2020	National Center for Health Statistics https://www.cdc.gov/nchs/nvss/vsrr/covid_weekly/index.htm
Tennessee, United States	18th August 2020	Department of Health https://www.tn.gov/content/tn/health/cedep/ncov/data.html
Texas, United States	17th August 2020	Deaprtmetn of State Health Services https://www.dshs.texas.gov/coronavirus/
Utah, United States	8th August 2020	National Center for Health Statistics https://www.cdc.gov/nchs/nvss/vsrr/covid_weekly/index.htm
Vermont, United States	25th August 2020	Health Vermont https://www.healthvermont.gov/response/coronavirus-covid-19/current-activity-vermont
Virginia, United States	18th August 2020	Virginia Open Data Portal https://data.virginia.gov/browse
Washington, United States	18th August 2020	Washington State Department of Health https://www.doh.wa.gov/Emergencies/COVID19#CovidDataTables
West Virginia, United States	8th August 2020	National Center for Health Statistics https://www.cdc.gov/nchs/nvss/vsrr/covid_weekly/index.htm
Wisconsin, United States	11th August 2020	Wisconsin Department of Health Services https://www.dhs.wisconsin.gov/outbreaks/index.htm

Aguascalientes, Mexico	19th August 2020	Gobierno de Mexico https://www.gob.mx/salud/documentos/lineamiento-estandarizado-para-la-vigilancia-epidemiologica-y-por-laboratorio-de-la-enfermedad-respiratoria-viral
Baja California, Mexico	19th August 2020	Gobierno de Mexico https://www.gob.mx/salud/documentos/lineamiento-estandarizado-para-la-vigilancia-epidemiologica-y-por-laboratorio-de-la-enfermedad-respiratoria-viral
Baja California Sur, Mexico	19th August 2020	Gobierno de Mexico https://www.gob.mx/salud/documentos/lineamiento-estandarizado-para-la-vigilancia-epidemiologica-y-por-laboratorio-de-la-enfermedad-respiratoria-viral
Campeche, Mexico	19th August 2020	Gobierno de Mexico https://www.gob.mx/salud/documentos/lineamiento-estandarizado-para-la-vigilancia-epidemiologica-y-por-laboratorio-de-la-enfermedad-respiratoria-viral
Coahuila, Mexico	19th August 2020	Gobierno de Mexico https://www.gob.mx/salud/documentos/lineamiento-estandarizado-para-la-vigilancia-epidemiologica-y-por-laboratorio-de-la-enfermedad-respiratoria-viral
Colima, Mexico	19th August 2020	Gobierno de Mexico https://www.gob.mx/salud/documentos/lineamiento-estandarizado-para-la-vigilancia-epidemiologica-y-por-laboratorio-de-la-enfermedad-respiratoria-viral
Chiapas, Mexico	19th August 2020	Gobierno de Mexico https://www.gob.mx/salud/documentos/lineamiento-estandarizado-para-la-vigilancia-epidemiologica-y-por-laboratorio-de-la-enfermedad-respiratoria-viral
Chihuahua, Mexico	19th August 2020	Gobierno de Mexico https://www.gob.mx/salud/documentos/lineamiento-estandarizado-para-la-vigilancia-epidemiologica-y-por-laboratorio-de-la-enfermedad-respiratoria-viral
Mexico City, Mexico	19th August 2020	Gobierno de Mexico https://www.gob.mx/salud/documentos/lineamiento-estandarizado-para-la-vigilancia-

		epidemiologica-y-por-laboratorio-de-la-enfermedad-respiratoria-viral
Durango, Mexico	19th August 2020	Gobierno de Mexico https://www.gob.mx/salud/documentos/lineamiento-estandarizado-para-la-vigilancia-epidemiologica-y-por-laboratorio-de-la-enfermedad-respiratoria-viral
Guanajuato, Mexico	19th August 2020	Gobierno de Mexico https://www.gob.mx/salud/documentos/lineamiento-estandarizado-para-la-vigilancia-epidemiologica-y-por-laboratorio-de-la-enfermedad-respiratoria-viral
Guerrero, Mexico	19th August 2020	Gobierno de Mexico https://www.gob.mx/salud/documentos/lineamiento-estandarizado-para-la-vigilancia-epidemiologica-y-por-laboratorio-de-la-enfermedad-respiratoria-viral
Hidalgo, Mexico	19th August 2020	Gobierno de Mexico https://www.gob.mx/salud/documentos/lineamiento-estandarizado-para-la-vigilancia-epidemiologica-y-por-laboratorio-de-la-enfermedad-respiratoria-viral
Jalisco, Mexico	19th August 2020	Gobierno de Mexico https://www.gob.mx/salud/documentos/lineamiento-estandarizado-para-la-vigilancia-epidemiologica-y-por-laboratorio-de-la-enfermedad-respiratoria-viral
Mexico, Mexico	19th August 2020	Gobierno de Mexico https://www.gob.mx/salud/documentos/lineamiento-estandarizado-para-la-vigilancia-epidemiologica-y-por-laboratorio-de-la-enfermedad-respiratoria-viral
Michoacan de Ocampo, Mexico	19th August 2020	Gobierno de Mexico https://www.gob.mx/salud/documentos/lineamiento-estandarizado-para-la-vigilancia-epidemiologica-y-por-laboratorio-de-la-enfermedad-respiratoria-viral
Morelos, Mexico	19th August 2020	Gobierno de Mexico https://www.gob.mx/salud/documentos/lineamiento-estandarizado-para-la-vigilancia-epidemiologica-y-por-laboratorio-de-la-enfermedad-respiratoria-viral

Nayarit, Mexico	19th August 2020	Gobierno de Mexico https://www.gob.mx/salud/documentos/lineamiento-estandarizado-para-la-vigilancia-epidemiologica-y-por-laboratorio-de-la-enfermedad-respiratoria-viral
Nuevo Leon, Mexico	19th August 2020	Gobierno de Mexico https://www.gob.mx/salud/documentos/lineamiento-estandarizado-para-la-vigilancia-epidemiologica-y-por-laboratorio-de-la-enfermedad-respiratoria-viral
Oaxaca, Mexico	19th August 2020	Gobierno de Mexico https://www.gob.mx/salud/documentos/lineamiento-estandarizado-para-la-vigilancia-epidemiologica-y-por-laboratorio-de-la-enfermedad-respiratoria-viral
Puebla, Mexico	19th August 2020	Gobierno de Mexico https://www.gob.mx/salud/documentos/lineamiento-estandarizado-para-la-vigilancia-epidemiologica-y-por-laboratorio-de-la-enfermedad-respiratoria-viral
Queretaro, Mexico	19th August 2020	Gobierno de Mexico https://www.gob.mx/salud/documentos/lineamiento-estandarizado-para-la-vigilancia-epidemiologica-y-por-laboratorio-de-la-enfermedad-respiratoria-viral
Quintana Roo, Mexico	19th August 2020	Gobierno de Mexico https://www.gob.mx/salud/documentos/lineamiento-estandarizado-para-la-vigilancia-epidemiologica-y-por-laboratorio-de-la-enfermedad-respiratoria-viral
San Luis Potosi, Mexico	19th August 2020	Gobierno de Mexico https://www.gob.mx/salud/documentos/lineamiento-estandarizado-para-la-vigilancia-epidemiologica-y-por-laboratorio-de-la-enfermedad-respiratoria-viral
Sinaloa, Mexico	19th August 2020	Gobierno de Mexico https://www.gob.mx/salud/documentos/lineamiento-estandarizado-para-la-vigilancia-epidemiologica-y-por-laboratorio-de-la-enfermedad-respiratoria-viral
Sonora, Mexico	19th August 2020	Gobierno de Mexico https://www.gob.mx/salud/documentos/lineamiento-estandarizado-para-la-vigilancia-

		epidemiologica-y-por-laboratorio-de-la-enfermedad-respiratoria-viral
Tabasco, Mexico	19th August 2020	Gobierno de Mexico https://www.gob.mx/salud/documentos/lineamiento-estandarizado-para-la-vigilancia-epidemiologica-y-por-laboratorio-de-la-enfermedad-respiratoria-viral
Tamaulipas, Mexico	19th August 2020	Gobierno de Mexico https://www.gob.mx/salud/documentos/lineamiento-estandarizado-para-la-vigilancia-epidemiologica-y-por-laboratorio-de-la-enfermedad-respiratoria-viral
Tlaxcala, Mexico	19th August 2020	Gobierno de Mexico https://www.gob.mx/salud/documentos/lineamiento-estandarizado-para-la-vigilancia-epidemiologica-y-por-laboratorio-de-la-enfermedad-respiratoria-viral
Veracruz de Ignacio de al Llave, Mexico	19th August 2020	Gobierno de Mexico https://www.gob.mx/salud/documentos/lineamiento-estandarizado-para-la-vigilancia-epidemiologica-y-por-laboratorio-de-la-enfermedad-respiratoria-viral
Yucatan, Mexico	19th August 2020	Gobierno de Mexico https://www.gob.mx/salud/documentos/lineamiento-estandarizado-para-la-vigilancia-epidemiologica-y-por-laboratorio-de-la-enfermedad-respiratoria-viral
Zacatecas, Mexico	19th August 2020	Gobierno de Mexico https://www.gob.mx/salud/documentos/lineamiento-estandarizado-para-la-vigilancia-epidemiologica-y-por-laboratorio-de-la-enfermedad-respiratoria-viral
North Sumatra, Indonesia	17th August 2020	Komite Penanganan COVID-19 Dan Pemulihan Ekonomi Nasional https://covid19.go.id/peta-sebaran
South Sumatra, Indonesia	17th August 2020	Komite Penanganan COVID-19 Dan Pemulihan Ekonomi Nasional https://covid19.go.id/peta-sebaran
Jakarta, Indonesia	17th August 2020	Komite Penanganan COVID-19 Dan Pemulihan Ekonomi Nasional https://covid19.go.id/peta-sebaran

West Java, Indonesia	17th August 2020	Komite Penanganan COVID-19 Dan Pemulihan Ekonomi Nasional https://covid19.go.id/peta-sebaran
Central Java, Indonesia	17th August 2020	Komite Penanganan COVID-19 Dan Pemulihan Ekonomi Nasional https://covid19.go.id/peta-sebaran
East Java, Indonesia	17th August 2020	Komite Penanganan COVID-19 Dan Pemulihan Ekonomi Nasional https://covid19.go.id/peta-sebaran
Banten, Indonesia	17th August 2020	Komite Penanganan COVID-19 Dan Pemulihan Ekonomi Nasional https://covid19.go.id/peta-sebaran
West Nusa Tenggara, Indonesia	17th August 2020	Komite Penanganan COVID-19 Dan Pemulihan Ekonomi Nasional https://covid19.go.id/peta-sebaran
Central Kalimantan, Indonesia	17th August 2020	Komite Penanganan COVID-19 Dan Pemulihan Ekonomi Nasional https://covid19.go.id/peta-sebaran
South Kalimantan, Indonesia	17th August 2020	Komite Penanganan COVID-19 Dan Pemulihan Ekonomi Nasional https://covid19.go.id/peta-sebaran
East Kalimantan, Indonesia	17th August 2020	Komite Penanganan COVID-19 Dan Pemulihan Ekonomi Nasional https://covid19.go.id/peta-sebaran
North Sulawesi, Indonesia	17th August 2020	Komite Penanganan COVID-19 Dan Pemulihan Ekonomi Nasional https://covid19.go.id/peta-sebaran
South Sulawesi, Indonesia	17th August 2020	Komite Penanganan COVID-19 Dan Pemulihan Ekonomi Nasional https://covid19.go.id/peta-sebaran
North Maluku, Indonesia	17th August 2020	Komite Penanganan COVID-19 Dan Pemulihan Ekonomi Nasional https://covid19.go.id/peta-sebaran
Alagoas, Brazil	26th July 2020	Government of State of Alagoas http://www.alagoascontraocoronavirus.al.gov.br/
Amazonas, Brazil	8th July 2020	FVS Amazonas http://www.fvs.am.gov.br/publicacoes
Amapa, Brazil	5th August 2020	Governo do Estado http://painel.corona.ap.gov.br/
Ceara, Brazil	13th August 2020	Department of Health https://coronavirus.ceara.gov.br/boletins/
Paraiba, Brazil	19th August 2020	Department of Health https://superset.plataformatarget.com.br/superse

		t/explore_json/?form_data=%7B%22slice_id%22%3A1549%7D&csv=true
Parana, Brazil	12th August 2020	Governo do Estado https://www.saude.pr.gov.br/
Pernambuco, Brazil	26th July 2020	Open Data https://dados.seplag.pe.gov.br/apps/corona_dados.html
Santa Catarina, Brazil	26th July 2020	Government of Santa Catarina http://www.coronavirus.sc.gov.br/category/boletins/
Sergipe, Brazil	19th August 2020	Ministry of Health https://todoscontraocorona.net.br/
Delhi, India	20th May 2020	Government of Delhi http://health.delhigovt.nic.in/wps/wcm/connect/doit_health/Health/Home/Covid19/
Jharkhand, India	8th June 2020	DD News Jharkhand https://twitter.com/rnuddkranchi/status/1314231773610430464
Karnataka, India	5th August 2020	Department of Health and Family Welfare https://drive.google.com/file/d/1jfRJOMdvPRBl5wgZh1LElMvaofNAxJGY/view
Tamil Nadu, India	27th July 2020	Health and Family Welfare Department https://twitter.com/ANI/status/1314183102533570560
La Rioja, Spain	17th August 2020	Gobierno de La Rioja https://actualidad.larioja.org/coronavirus/datos

SI Table 11. β regression coefficient constraints

Covariate	Time-varying	Lower Bound	Upper Bound
Pneumonia Seasonality	Yes	0.9	1.31
Mobility	Yes	0	∞
Mask Use	Yes	-0.52	0
Testing	Yes	-80	0
Air Quality	No	0	∞
Smoking Prevalence	No	0	1
LRI Mortality	No	0	∞
Altitude	No	0	∞
Population Density	No	0	∞

SI Table 12 Cumulative deaths 21 September 2020 through 28 February 2021, maximum estimated daily deaths per million population, date of maximum daily deaths, and estimated $R_{effective}$ on 28 February 2021 for two additional derivative scenarios.

Location	less comprehensive mask use (85% of population wears masks and SDM re-imposed at daily death rate threshold of 8/million)				"Masks only" (95% of population wears masks, SDM are removed and not reinstated)			
	**Cumulative deaths through 28 February 2021**	**Maximum estimated daily deaths per million**	**Date of maximum daily deaths**	**Estimated $R_{effective}$ on 28 February 2021**	**Cumulative deaths through 28 February 2021**	**Maximum estimated daily deaths per million**	**Date of maximum daily deaths**	**Estimated $R_{effective}$ on 28 February 2021**
United States of America	415559 (377160-451197)	6.3 (4.7-7.6)	1/26/21	NA	490437 (379492-665753)	14.9 (8.3-27)	2/28/21	NA
California	53900 (42694-68160)	10.9 (6-15.1)	1/19/21	0.86 (0.81-0.91)	65335 (38380-122247)	27.3 (11.1-61.8)	2/28/21	0.95 (0.82-1.1)
Florida	28981 (23397-31802)	6.2 (0.8-10.9)	12/19/20	0.87 (0.66-1.07)	42096 (26825-65821)	16.8 (7.6-33.1)	2/26/21	0.91 (0.83-1.02)
New York	41540 (35559-48724)	5.6 (1.9-13.1)	2/18/21	0.86 (0.63-1.16)	41748 (34836-71491)	10.2 (1.5-42.9)	2/28/21	1.04 (0.85-1.21)
Pennsylvania	19430 (16909-22990)	8.8 (3.6-15.4)	12/31/20	0.9 (0.76-1.07)	37589 (17752-78473)	40 (13-86.8)	2/21/21	0.86 (0.65-1.02)
Texas	35414 (27254-40056)	7.3 (2.8-12.4)	1/28/21	0.72 (0.6-1.02)	35234 (24421-53967)	10.9 (4-22.4)	2/28/21	0.95 (0.84-1.09)
Illinois	16544 (13376-18728)	6.7 (3.1-11.1)	2/8/21	0.76 (0.61-1.03)	15364 (12085-22725)	8.4 (3.2-22.3)	2/28/21	0.98 (0.9-1.09)
New Jersey	22833 (19359-25729)	9.2 (2.2-17.6)	1/12/21	0.82 (0.66-1)	29090 (18368-45890)	25.5 (4.6-57.8)	2/17/21	0.84 (0.6-1.09)
Massachusetts	13782 (11657-14873)	8.8 (3.3-15.7)	2/2/21	0.74 (0.64-0.94)	14295 (10886-22980)	20.6 (4.6-69.9)	2/28/21	1.07 (0.95-1.25)

	less comprehensive mask use (85% of population wears masks and SDM re-imposed at daily death rate threshold of 8/million)				"Masks only" (95% of population wears masks, SDM are removed and not reinstated)			
Location	**Cumulative deaths through 28 February 2021**	**Maximum estimated daily deaths per million**	**Date of maximum daily deaths**	**Estimated $R_{effective}$ on 28 February 2021**	**Cumulative deaths through 28 February 2021**	**Maximum estimated daily deaths per million**	**Date of maximum daily deaths**	**Estimated $R_{effective}$ on 28 February 2021**
Michigan	12192 (9380-14492)	5.7 (1.5-11.9)	2/11/21	0.82 (0.59-1.1)	11974 (8798-22436)	9.4 (2.1-34.7)	2/28/21	1.02 (0.91-1.16)
North Carolina	12557 (9901-14941)	9.3 (4.6-14)	1/17/21	0.84 (0.75-0.96)	15217 (8140-28550)	21.1 (8-43.8)	2/28/21	0.93 (0.8-1.08)
Ohio	10500 (7225-13383)	6.7 (2.6-11.1)	2/21/21	0.84 (0.66-1.06)	9071 (6834-12840)	6 (2-15)	2/28/21	0.99 (0.91-1.11)
Georgia	14505 (11001-17038)	6 (2.8-10.9)	10/25/20	0.82 (0.64-1.02)	14994 (10683-22756)	8.3 (2.5-19)	2/28/21	0.94 (0.83-1.04)
Missouri	6493 (4601-8350)	7.9 (2.4-16.6)	10/27/20	0.92 (0.67-1.14)	13391 (5947-27818)	22.7 (7.3-48.2)	2/15/21	0.87 (0.69-1.01)
Indiana	7044 (5236-8395)	6.3 (2.7-11.2)	2/12/21	0.8 (0.6-1.05)	6353 (4823-9505)	6.9 (2.2-17.9)	2/28/21	0.98 (0.9-1.09)
Connecticut	6598 (5081-8190)	8.1 (1.9-16)	2/3/21	0.82 (0.65-1.12)	7666 (4869-19376)	26.6 (2.4-108.5)	2/28/21	1.05 (0.77-1.26)
Arizona	10769 (8350-12204)	6.9 (2.8-14)	10/26/20	0.85 (0.63-1.17)	12677 (9092-18126)	11.6 (5-21.9)	2/24/21	0.88 (0.73-1.02)
Colorado	5761 (3794-7460)	8.8 (2.9-16.4)	1/26/21	0.79 (0.66-1)	8013 (3357-19496)	25.7 (5.8-67.4)	2/28/21	0.92 (0.68-1.11)
Maryland	7906 (6588-9003)	8.5 (4.7-13.9)	1/30/21	0.75 (0.64-0.93)	8410 (5895-15574)	16.2 (5.5-44.4)	2/28/21	0.96 (0.83-1.08)
Minnesota	5079 (3499-6098)	6.5 (2.1-12)	2/6/21	0.74 (0.56-1.02)	4848 (3166-8773)	9.1 (2.5-27.3)	2/28/21	0.97 (0.89-1.08)
Virginia	10904 (7491-17325)	7.7 (2.2-18.1)	10/30/20	0.91 (0.73-1.1)	21863 (8446-46746)	28.7 (7.9-64.5)	2/17/21	0.87 (0.66-1.02)

Location	less comprehensive mask use (85% of population wears masks and SDM re-imposed at daily death rate threshold of 8/million)				"Masks only" (95% of population wears masks, SDM are removed and not reinstated)			
	Cumulative deaths through 28 February 2021	Maximum estimated daily deaths per million	Date of maximum daily deaths	Estimated $R_{effective}$ on 28 February 2021	Cumulative deaths through 28 February 2021	Maximum estimated daily deaths per million	Date of maximum daily deaths	Estimated $R_{effective}$ on 28 February 2021
Alabama	6049 (4240-7305)	6.3 (0.9-10.7)	1/27/21	0.83 (0.69-1)	6053 (3757-11137)	7.9 (2.1-19.1)	2/26/21	0.91 (0.81-1.01)
South Carolina	6580 (4906-8074)	5.7 (0.8-9.9)	2/17/21	0.85 (0.64-1.04)	6075 (4644-8213)	5.5 (1.6-12.1)	2/28/21	0.95 (0.88-1.05)
Tennessee	6060 (3190-8053)	5.9 (1-11.1)	2/8/21	0.83 (0.67-1.05)	5519 (3028-9374)	6.7 (0.8-17.7)	2/28/21	0.95 (0.86-1.06)
Louisiana	7997 (6447-9537)	6.3 (1.9-10.5)	2/19/21	0.84 (0.67-1.06)	7460 (6276-9338)	5.6 (1.4-12.9)	2/28/21	0.96 (0.87-1.07)
Nevada	3825 (2650-4632)	7.8 (2.9-13.5)	2/6/21	0.78 (0.66-1.08)	3843 (2397-7095)	13.3 (3.7-35)	2/28/21	1 (0.88-1.15)
Kansas	2443 (960-3654)	7.3 (0.7-14.4)	1/18/21	0.85 (0.68-1.05)	3222 (904-8955)	18.1 (0.8-61.3)	2/28/21	0.94 (0.79-1.1)
New Mexico	2689 (2224-3270)	9.8 (4.9-14.6)	1/20/21	0.83 (0.74-0.96)	3489 (1923-7809)	27.1 (9.3-71.2)	2/28/21	0.95 (0.79-1.1)
Wisconsin	2953 (1817-4880)	4.6 (1.2-10.8)	2/28/21	0.93 (0.62-1.11)	2484 (1769-3938)	3.3 (0.9-10)	2/28/21	0.99 (0.91-1.13)
Arkansas	3215 (2162-4138)	6.7 (2-13.2)	10/25/20	0.88 (0.64-1.07)	4073 (2017-7847)	10 (1.8-25.5)	2/28/21	0.94 (0.87-1.04)
Oklahoma	3649 (2538-4356)	6.3 (1.4-11.4)	1/18/21	0.77 (0.59-0.99)	3949 (2186-8115)	9.7 (3.1-24.5)	2/28/21	0.91 (0.8-1.02)
Washington	4340 (3080-5902)	4.6 (1.8-7.6)	2/17/21	0.96 (0.89-1.06)	4081 (2809-7034)	6 (1.3-18.7)	2/28/21	0.95 (0.9-1.04)
Kentucky	2804 (1577-4225)	5.1 (0.8-11)	2/20/21	0.87 (0.62-1.08)	2396 (1507-4106)	4.6 (0.7-14.4)	2/28/21	0.97 (0.89-1.1)
Mississippi	5019 (4120-5915)	8.1 (3.5-17.7)	10/21/20	0.89 (0.67-1.02)	5503 (4109-8215)	8.2 (3.5-17.5)	10/22/20	0.91 (0.83-1)

Location	less comprehensive mask use (85% of population wears masks and SDM re-imposed at daily death rate threshold of 8/million)				"Masks only" (95% of population wears masks, SDM are removed and not reinstated)			
	**Cumulative deaths through 28 February 2021**	**Maximum estimated daily deaths per million**	**Date of maximum daily deaths**	**Estimated $R_{effective}$ on 28 February 2021**	**Cumulative deaths through 28 February 2021**	**Maximum estimated daily deaths per million**	**Date of maximum daily deaths**	**Estimated $R_{effective}$ on 28 February 2021**
Rhode Island	1976 (1890-2085)	8.6 (4.1-13.7)	12/26/20	0.92 (0.76-1.1)	3368 (2064-5864)	43.2 (19.8-79.1)	2/27/21	0.87 (0.67-1.04)
Nebraska	1529 (822-1986)	6.7 (1.7-12.6)	1/28/21	0.78 (0.63-1.01)	1520 (736-3416)	10.4 (1.6-35.6)	2/28/21	0.94 (0.87-1.05)
West Virginia	1537 (727-2099)	6.8 (1.6-11.8)	2/6/21	0.86 (0.72-1.07)	1536 (654-3466)	11.3 (1.7-36.5)	2/28/21	1 (0.91-1.13)
Iowa	2142 (1522-3661)	2.8 (1.3-5.3)	10/6/20	0.94 (0.67-1.04)	1964 (1502-3106)	2.8 (1.3-5.1)	10/6/20	0.95 (0.9-1.04)
Idaho	1289 (634-2029)	5.5 (0.8-11.6)	2/16/21	0.85 (0.65-1.08)	1224 (609-2787)	7.7 (0.7-27.7)	2/28/21	0.98 (0.88-1.12)
North Dakota	724 (508-1122)	14.4 (5.2-30.4)	10/20/20	0.95 (0.7-1.09)	1546 (696-3004)	15.4 (5.2-33.5)	10/24/20	0.87 (0.77-0.96)
Delaware	1302 (1118-1409)	7.7 (3.6-13.2)	1/9/21	0.78 (0.65-0.94)	1474 (1001-2404)	13 (5.5-25.1)	2/25/21	0.9 (0.75-1.02)
Montana	543 (267-1082)	4.2 (1.7-9.7)	10/6/20	0.95 (0.7-1.06)	510 (256-1175)	4.1 (1.7-9.1)	10/6/20	0.96 (0.9-1.05)
Hawaii	576 (269-1121)	4.5 (0.8-10.6)	2/28/21	0.95 (0.72-1.13)	465 (253-923)	3.3 (0.6-11.3)	2/28/21	0.99 (0.88-1.15)
South Dakota	487 (300-848)	3.2 (2.2-4.4)	10/1/20	0.92 (0.6-1.08)	422 (292-753)	3.2 (2.2-4.4)	10/1/20	0.97 (0.91-1.09)
District of Columbia	791 (685-1007)	5.7 (1.6-12.9)	2/28/21	0.98 (0.69-1.21)	731 (666-857)	3.4 (0.8-10)	2/28/21	1.08 (0.96-1.25)
Oregon	701 (609-923)	0.7 (0.7-0.7)	9/22/20	1.03 (0.96-1.15)	666 (604-800)	0.7 (0.7-0.7)	9/22/20	1.02 (0.95-1.12)
Utah	549 (476-827)	0.5 (0-2.9)	2/28/21	1.02 (0.95-1.15)	520 (474-663)	0.4 (0.4-0.4)	9/28/20	1.01 (0.94-1.13)
Alaska	293 (68-815)	2.5 (0.1-13.3)	11/30/20	0.82 (0.6-0.96)	371 (67-1872)	3.8 (0.1-23.8)	1/21/21	0.81 (0.64-0.95)
New Hampshire	452 (441-480)	0.2 (0.2-0.2)	9/22/20	1 (0.94-1.09)	448 (441-463)	0.2 (0.2-0.2)	9/22/20	0.98 (0.92-1.07)

Location	less comprehensive mask use (85% of population wears masks and SDM re-imposed at daily death rate threshold of 8/million)				"Masks only" (95% of population wears masks, SDM are removed and not reinstated)			
	**Cumulative deaths through 28 February 2021**	**Maximum estimated daily deaths per million**	**Date of maximum daily deaths**	**Estimated $R_{effective}$ on 28 February 2021**	**Cumulative deaths through 28 February 2021**	**Maximum estimated daily deaths per million**	**Date of maximum daily deaths**	**Estimated $R_{effective}$ on 28 February 2021**
Maine	170 (146-237)	0.3 (0.3-0.3)	9/22/20	1.01 (0.93-1.12)	162 (145-205)	0.3 (0.3-0.3)	9/22/20	0.99 (0.9-1.1)
Vermont	72 (63-102)	0.6 (0.1-3.2)	2/28/21	1.02 (0.79-1.36)	69 (63-89)	0.4 (0.1-1.9)	2/28/21	0.99 (0.76-1.34)
Wyoming	70 (53-129)	0.7 (0.7-0.7)	9/22/20	0.93 (0.73-1.04)	65 (53-102)	0.7 (0.7-0.7)	9/22/20	0.92 (0.7-1.04)
