# Supplementary material for: Modeling COVID-19 scenarios for the United States
Source: Nat Med. 2020 Oct 23;27(1):94–105. doi: 10.1038/s41591-020-1132-9 (PMC7806509; doi:10.1038/s41591-020-1132-9)
Supplement: Supplementary file 2 — Reporting Summary [file 41591_2020_1132_MOESM2_ESM.pdf]

nature research

Corresponding author(s): SIMON I HAY

Last updated by author(s): Oct 9, 2020

Reporting Summary

Nature Research wishes to improve the reproducibility of the work that we publish. This form provides structure for consistency and transparency in reporting. For further information on Nature Research policies, see our Editorial Policies and the Editorial Policy Checklist.

Statistics

For all statistical analyses, confirm that the following items are present in the figure legend, table legend, main text, or Methods section.

n/a	Confirmed	
☐	☒	The exact sample size (*n*) for each experimental group/condition, given as a discrete number and unit of measurement
☐	☒	A statement on whether measurements were taken from distinct samples or whether the same sample was measured repeatedly
☒	☐	The statistical test(s) used AND whether they are one- or two-sided<br>*Only common tests should be described solely by name; describe more complex techniques in the Methods section.*
☒	☐	A description of all covariates tested
☐	☒	A description of any assumptions or corrections, such as tests of normality and adjustment for multiple comparisons
☐	☒	A full description of the statistical parameters including central tendency (e.g. means) or other basic estimates (e.g. regression coefficient) AND variation (e.g. standard deviation) or associated estimates of uncertainty (e.g. confidence intervals)
☒	☐	For null hypothesis testing, the test statistic (e.g. *F*, *t*, *r*) with confidence intervals, effect sizes, degrees of freedom and *P* value noted<br>*Give P values as exact values whenever suitable.*
☐	☒	For Bayesian analysis, information on the choice of priors and Markov chain Monte Carlo settings
☒	☐	For hierarchical and complex designs, identification of the appropriate level for tests and full reporting of outcomes
☒	☐	Estimates of effect sizes (e.g. Cohen's *d*, Pearson's *r*), indicating how they were calculated

Our web collection on statistics for biologists contains articles on many of the points above.

Software and code

Policy information about availability of computer code

Data collection	No primary data collection was carried out for this analysis. No software was used for primary data collection purposes.
Data analysis	All code used for these analyses was custom created for this study and is publicly available online (https://github.com/ihmeuw/covid-model-seiir-pipeline; https://github.com/ihmeuw/covid-model-deaths-spline). Analyses were carried out using R version 3.6.1, python 3.8, and using R-INLA v20.01.29.9000. All maps presented in this study are generated by the authors using RStudio (R Version 3.6.3) and ArcGIS Desktop 10.6 and no permissions are required to publish them.

For manuscripts utilizing custom algorithms or software that are central to the research but not yet described in published literature, software must be made available to editors and reviewers. We strongly encourage code deposition in a community repository (e.g. GitHub). See the Nature Research guidelines for submitting code & software for further information.

Data

Policy information about availability of data

All manuscripts must include a data availability statement. This statement should provide the following information, where applicable:

- Accession codes, unique identifiers, or web links for publicly available datasets
- A list of figures that have associated raw data
- A description of any restrictions on data availability

[TO BE UPDATED WITH FINAL TEXT]

Field-specific reporting

Please select the one below that is the best fit for your research. If you are not sure, read the appropriate sections before making your selection.

☒ Life sciences ☐ Behavioural & social sciences ☐ Ecological, evolutionary & environmental sciences

For a reference copy of the document with all sections, see nature.com/documents/nr-reporting-summary-flat.pdf

Life sciences study design

All studies must disclose on these points even when the disclosure is negative.

Sample size	Sample size was calculated as the number of unique data source-location pairs with observations of covid-19 epidemiological data. This sample size is reported in the Supplementary Information, section 2; we use 10,984 location-days of covid-19 epidemiological data.
Data exclusions	Reasons for data exclusion were pre-established and are described in supplementary information section 2. Principal epidemiological data streams were excluded and replaced with human curated alternatives based on the pre-established verification process described in supplementary information section 2.
Replication	This is an observational study using many months of reported COVID-19 epidemiological data, combined with covariate data, and could be replicated using publicly available code and data.
Randomization	There was no experimental design or group allocation involved in this study.
Blinding	There was no experimental design or group allocation involved in this study.

Reporting for specific materials, systems and methods

We require information from authors about some types of materials, experimental systems and methods used in many studies. Here, indicate whether each material, system or method listed is relevant to your study. If you are not sure if a list item applies to your research, read the appropriate section before selecting a response.

Materials & experimental systems

n/a	Involved in the study	
☒	☐	Antibodies
☒	☐	Eukaryotic cell lines
☒	☐	Palaeontology and archaeology
☒	☐	Animals and other organisms
☒	☐	Human research participants
☒	☐	Clinical data
☒	☐	Dual use research of concern

Methods

n/a	Involved in the study	
☒	☐	ChIP-seq
☒	☐	Flow cytometry
☒	☐	MRI-based neuroimaging
