# Supplementary material for: Modeling COVID-19 scenarios for the United States
Source: Nat Med. 2020 Oct 23;27(1):94–105. doi: 10.1038/s41591-020-1132-9 (PMC7806509; doi:10.1038/s41591-020-1132-9)
Supplement: Supplementary file 3 — Appendix 1: SEIR model vetting plots for the scenario of 95% mask use with mandates. [file 41591_2020_1132_MOESM3_ESM.pdf]

Appendix 1:
SEIR Model Vetting Plots
Scenario: 95% Mask Use With Mandates

Contents

1 United States of America: SEIR fit comparison 7

2 United States of America: Covariate fits and regression coefficients 8

3 Alabama: SEIR fit comparison 9

4 Alabama: Covariate fits and regression coefficients 10

5 Alaska: SEIR fit comparison 11

6 Alaska: Covariate fits and regression coefficients 12

7 Arizona: SEIR fit comparison 13

8 Arizona: Covariate fits and regression coefficients 14

9 Arkansas: SEIR fit comparison 15

10 Arkansas: Covariate fits and regression coefficients 16

11 California: SEIR fit comparison 17

12 California: Covariate fits and regression coefficients 18

13 Colorado: SEIR fit comparison 19

14 Colorado: Covariate fits and regression coefficients 20

15 Connecticut: SEIR fit comparison 21

16 Connecticut: Covariate fits and regression coefficients 22

17 Delaware: SEIR fit comparison 23

18 Delaware: Covariate fits and regression coefficients 24

19 District of Columbia: SEIR fit comparison 25

20 District of Columbia: Covariate fits and regression coefficients 26

21 Florida: SEIR fit comparison 27

22 Florida: Covariate fits and regression coefficients 28

23 Georgia: SEIR fit comparison 29

24 Georgia: Covariate fits and regression coefficients **30**

25 Hawaii: SEIR fit comparison **31**

26 Hawaii: Covariate fits and regression coefficients **32**

27 Idaho: SEIR fit comparison **33**

28 Idaho: Covariate fits and regression coefficients **34**

29 Illinois: SEIR fit comparison **35**

30 Illinois: Covariate fits and regression coefficients **36**

31 Indiana: SEIR fit comparison **37**

32 Indiana: Covariate fits and regression coefficients **38**

33 Iowa: SEIR fit comparison **39**

34 Iowa: Covariate fits and regression coefficients **40**

35 Kansas: SEIR fit comparison **41**

36 Kansas: Covariate fits and regression coefficients **42**

37 Kentucky: SEIR fit comparison **43**

38 Kentucky: Covariate fits and regression coefficients **44**

39 Louisiana: SEIR fit comparison **45**

40 Louisiana: Covariate fits and regression coefficients **46**

41 Maine: SEIR fit comparison **47**

42 Maine: Covariate fits and regression coefficients **48**

43 Maryland: SEIR fit comparison **49**

44 Maryland: Covariate fits and regression coefficients **50**

45 Massachusetts: SEIR fit comparison **51**

46 Massachusetts: Covariate fits and regression coefficients **52**

47 Michigan: SEIR fit comparison **53**

48 Michigan: Covariate fits and regression coefficients **54**

49 Minnesota: SEIR fit comparison **55**

50 Minnesota: Covariate fits and regression coefficients **56**

51 Mississippi: SEIR fit comparison **57**

52 Mississippi: Covariate fits and regression coefficients **58**

53 Missouri: SEIR fit comparison **59**

54 Missouri: Covariate fits and regression coefficients **60**

55 Montana: SEIR fit comparison **61**

56 Montana: Covariate fits and regression coefficients **62**

57 Nebraska: SEIR fit comparison **63**

58 Nebraska: Covariate fits and regression coefficients **64**

59 Nevada: SEIR fit comparison **65**

60 Nevada: Covariate fits and regression coefficients **66**

61 New Hampshire: SEIR fit comparison **67**

62 New Hampshire: Covariate fits and regression coefficients **68**

63 New Jersey: SEIR fit comparison **69**

64 New Jersey: Covariate fits and regression coefficients **70**

65 New Mexico: SEIR fit comparison **71**

66 New Mexico: Covariate fits and regression coefficients **72**

67 New York: SEIR fit comparison **73**

68 New York: Covariate fits and regression coefficients **74**

69 North Carolina: SEIR fit comparison **75**

70 North Carolina: Covariate fits and regression coefficients **76**

71 North Dakota: SEIR fit comparison **77**

72 North Dakota: Covariate fits and regression coefficients **78**

73 Ohio: SEIR fit comparison **79**

74 Ohio: Covariate fits and regression coefficients **80**

75 Oklahoma: SEIR fit comparison **81**

76 Oklahoma: Covariate fits and regression coefficients **82**

77 Oregon: SEIR fit comparison **83**

78 Oregon: Covariate fits and regression coefficients **84**

79 Pennsylvania: SEIR fit comparison **85**

80 Pennsylvania: Covariate fits and regression coefficients **86**

81 Rhode Island: SEIR fit comparison **87**

82 Rhode Island: Covariate fits and regression coefficients **88**

83 South Carolina: SEIR fit comparison **89**

84 South Carolina: Covariate fits and regression coefficients **90**

85 South Dakota: SEIR fit comparison **91**

86 South Dakota: Covariate fits and regression coefficients **92**

87 Tennessee: SEIR fit comparison **93**

88 Tennessee: Covariate fits and regression coefficients **94**

89 Texas: SEIR fit comparison **95**

90 Texas: Covariate fits and regression coefficients **96**

91 Utah: SEIR fit comparison **97**

92 Utah: Covariate fits and regression coefficients **98**

93 Vermont: SEIR fit comparison **99**

94 Vermont: Covariate fits and regression coefficients **100**

95 Virginia: SEIR fit comparison **101**

96 Virginia: Covariate fits and regression coefficients **102**

97 Washington: SEIR fit comparison **103**

98 Washington: Covariate fits and regression coefficients **104**

99 West Virginia: SEIR fit comparison **105**

100 West Virginia: Covariate fits and regression coefficients **106**

101 Wisconsin: SEIR fit comparison **107**

102 Wisconsin: Covariate fits and regression coefficients **108**

103 Wyoming: SEIR fit comparison **109**

104 Wyoming: Covariate fits and regression coefficients **110**

105 Spokane County: SEIR fit comparison **111**

106 Spokane County: Covariate fits and regression coefficients **112**

107 King and Snohomish Counties: SEIR fit comparison **113**

108 King and Snohomish Counties: Covariate fits and regression coefficients **114**

109 Washington except for King, Snohomish, and Spokane Counties: SEIR fit comparison **115**

110 Washington except for King, Snohomish, and Spokane Counties: Covariate fits and regression coefficients **117**

1 United States of America: SEIR fit comparison

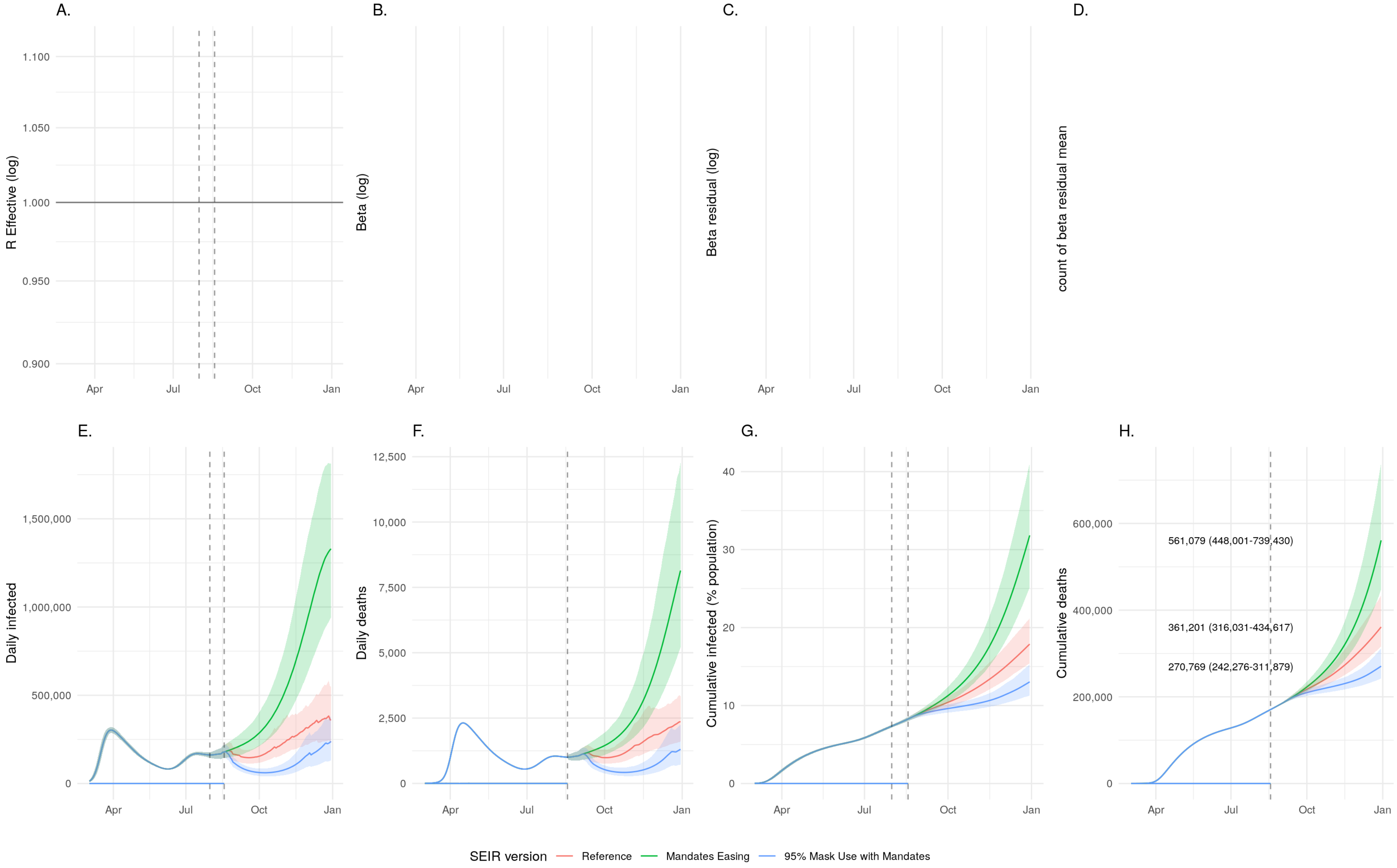

United States of America: SEIR fit comparison. Panels **A-D** display values that are not directly calculated for aggregate locations. **E**: predicted daily infections from each model through December 31. **F**: predicted daily deaths from each model through December 31. **G**: predicted cumulative infections through December 31, as a proportion of the total population. **H**: predicted cumulative deaths through December 31. In panels E, F, G, and H, reported death and infections are plotted alongside model predictions in light blue.

2 United States of America: Covariate fits and regression coefficients

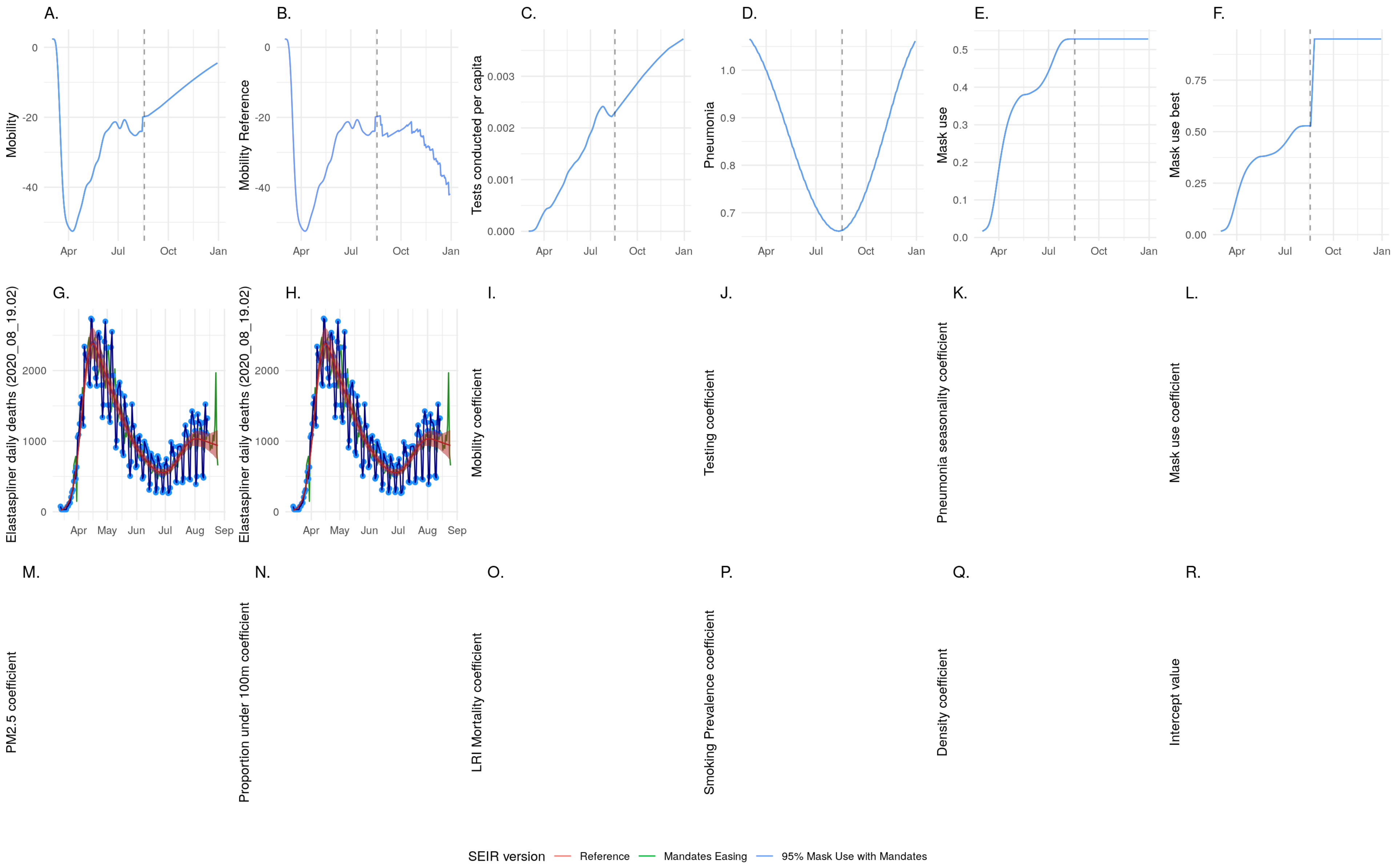

United States of America: Covariate fits and regression coefficients. A-F: Line plots showing predicted covariate time trends for **A)** mobility in the absence of additional mandates; **B)** mobility with additional mandates applied; **C)** diagnostic testing per capita; **D)** pneumonia seasonality; **E)** mask use per capita, and; **F)** mask use in a scenario where adherence increases to 95% of the population. **G-H**: COVID mortality data generated from reported daily deaths (blue); estimated based on reported hospitalizations (purple); estimated from reported cases (green); and via a spline fit through all available data types (red, 95% UI in pink). Panels **I-R** display coefficients for a regression fit to $log(beta)$, which is not applicable to aggregate locations.

3 Alabama: SEIR fit comparison

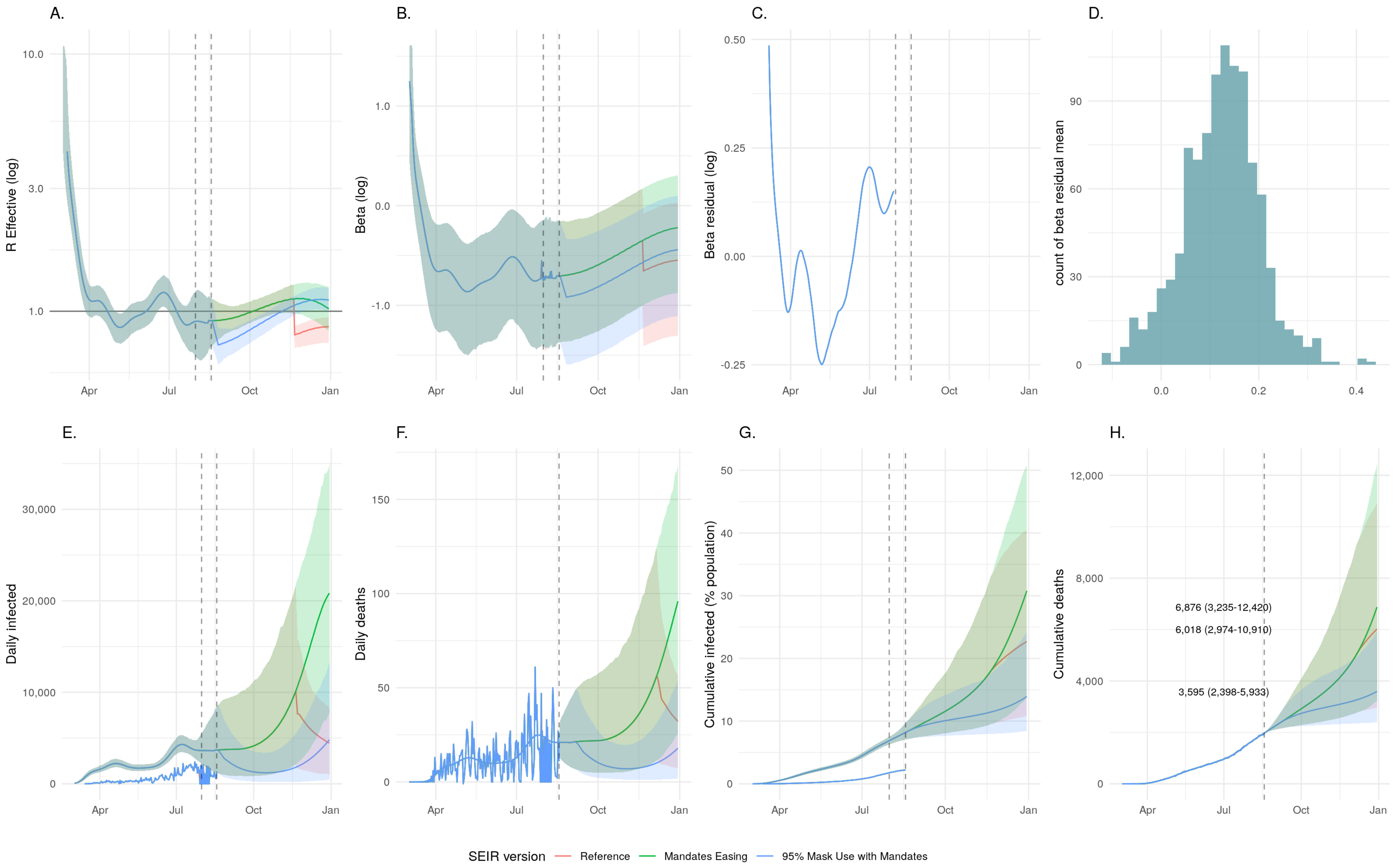

Alabama: SEIR fit comparison. **A**: predicted R effective for each model through December 31. **B**: predicted SEIR *beta* parameter. **C**: residual of predicted *beta* and the observed value calculated directly from infection data over time. **D**: histogram of residual values for *beta*. Panels A, B, C, and D are all displayed in log space, reflecting the space in which the SEIR model is fit. **E**: predicted daily infections from each model through December 31. **F**: predicted daily deaths from each model through December 31. **G**: predicted cumulative infections through December 31, as a proportion of the total population. **H**: predicted cumulative deaths through December 31. In panels E, F, G, and H, reported death and infections are plotted alongside model predictions in light blue.

4 Alabama: Covariate fits and regression coefficients

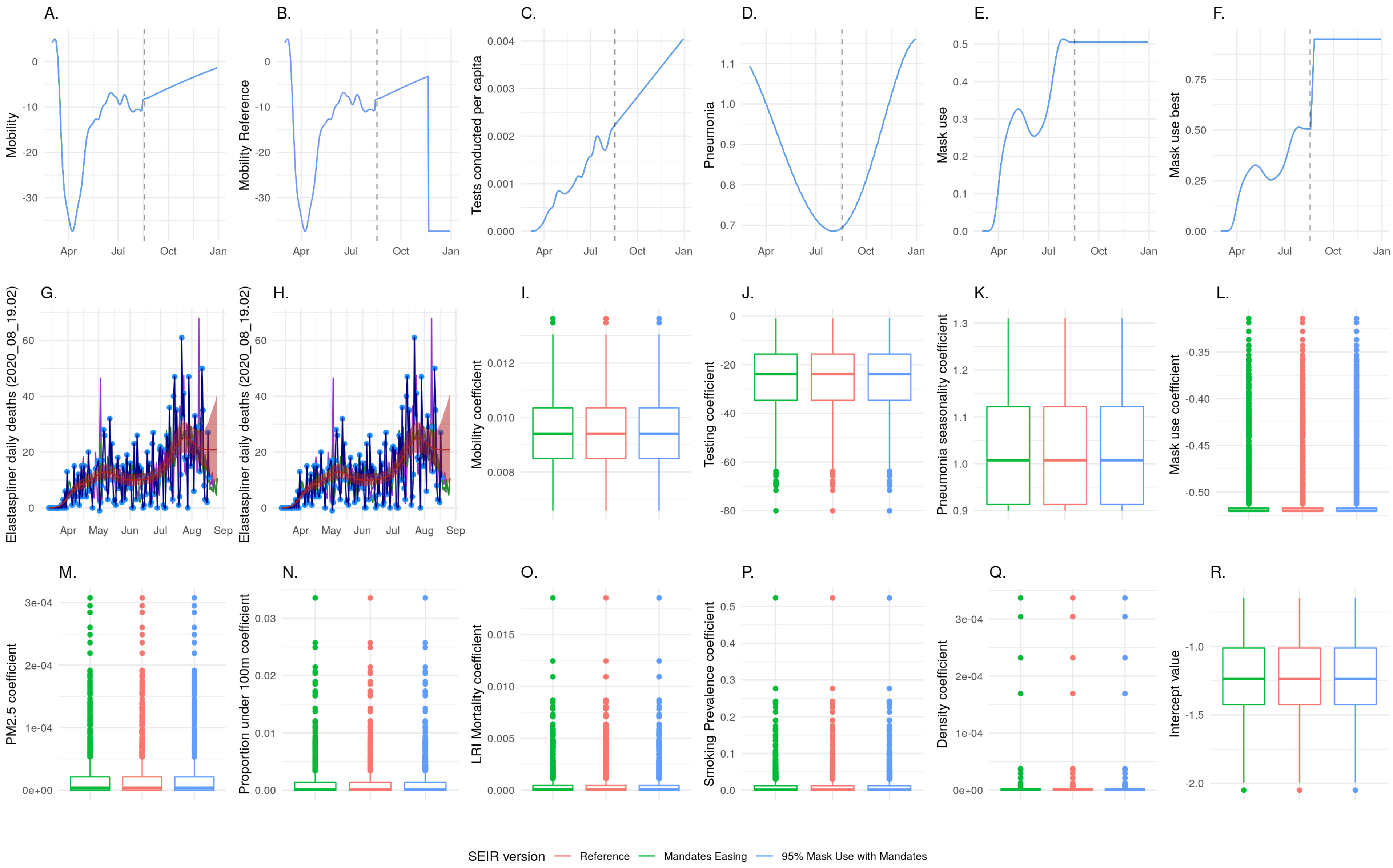

Alabama: Covariate fits and regression coefficients. **A-F**: Line plots showing predicted covariate time trends for **A)** mobility in the absence of additional mandates; **B)** mobility with additional mandates applied; **C)** diagnostic testing per capita; **D)** pneumonia seasonality; **E)** mask use per capita, and; **F)** mask use in a scenario where adherence increases to 95% of the population. **G-H**: COVID mortality data generated from reported daily deaths (blue); estimated based on reported hospitalizations (purple); estimated from reported cases (green); and via a spline fit through all available data types (red, 95% UI in pink). **I-R**: Box plots showing 1,000 draws of fixed effect coefficients in a multivariate regression fit to $log(beta)$.

5 Alaska: SEIR fit comparison

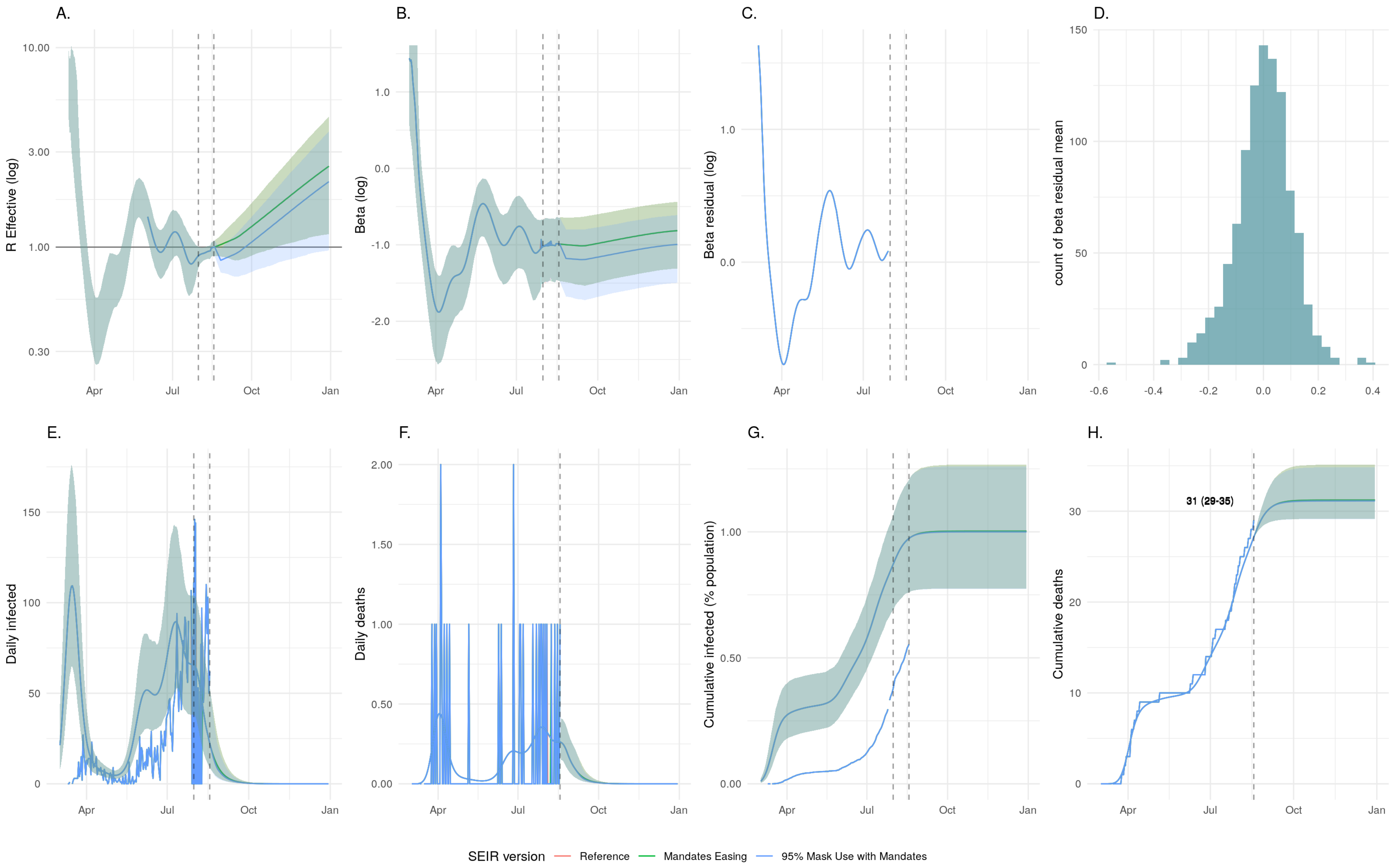

Alaska: SEIR fit comparison. **A**: predicted R effective for each model through December 31. **B**: predicted SEIR *beta* parameter. **C**: residual of predicted *beta* and the observed value calculated directly from infection data over time. **D**: histogram of residual values for *beta*. Panels A, B, C, and D are all displayed in log space, reflecting the space in which the SEIR model is fit. **E**: predicted daily infections from each model through December 31. **F**: predicted daily deaths from each model through December 31. **G**: predicted cumulative infections through December 31, as a proportion of the total population. **H**: predicted cumulative deaths through December 31. In panels E, F, G, and H, reported death and infections are plotted alongside model predictions in light blue.

6 Alaska: Covariate fits and regression coefficients

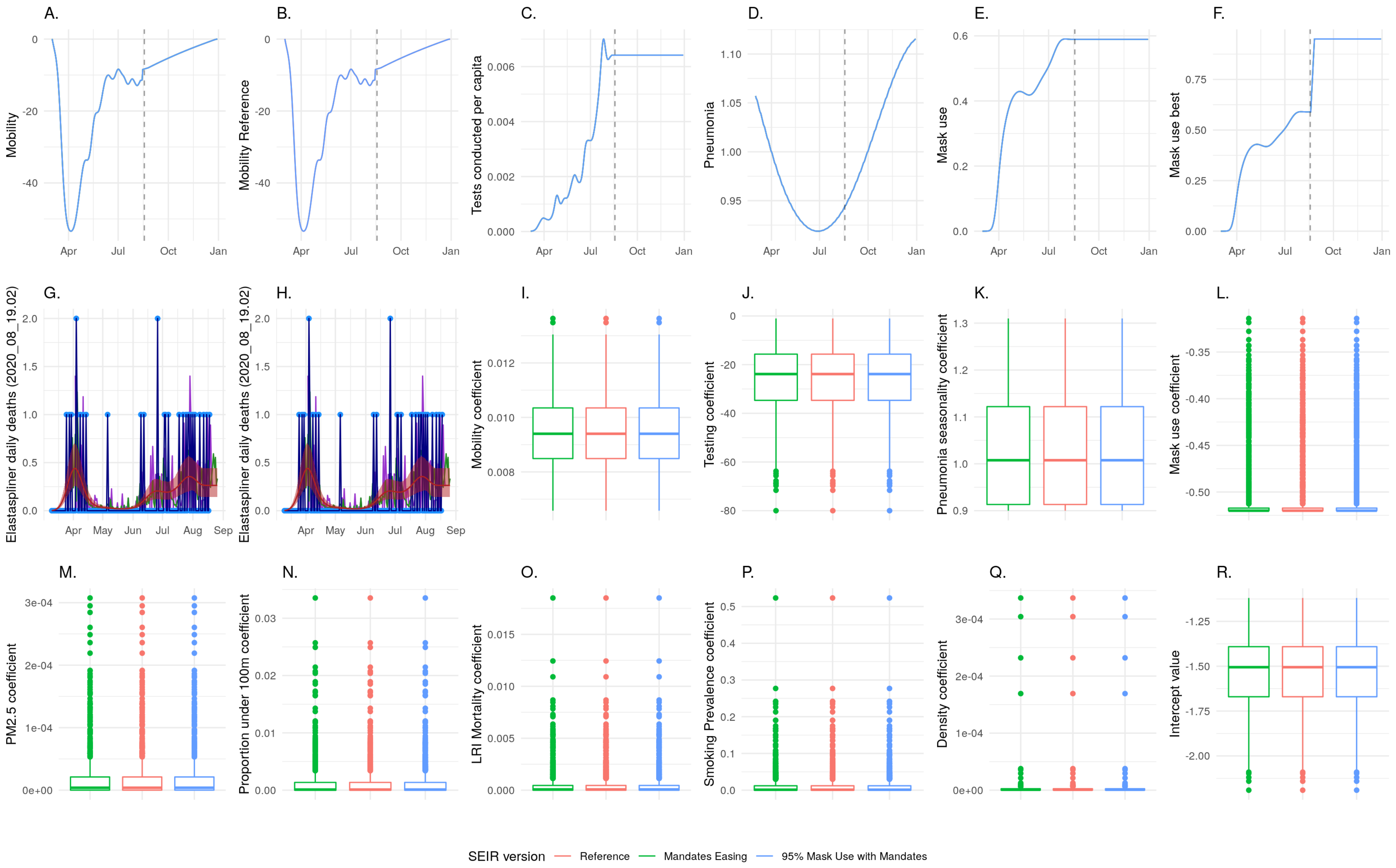

Alaska: Covariate fits and regression coefficients. **A-F**: Line plots showing predicted covariate time trends for **A)** mobility in the absence of additional mandates; **B)** mobility with additional mandates applied; **C)** diagnostic testing per capita; **D)** pneumonia seasonality; **E)** mask use per capita, and; **F)** mask use in a scenario where adherence increases to 95% of the population. **G-H**: COVID mortality data generated from reported daily deaths (blue); estimated based on reported hospitalizations (purple); estimated from reported cases (green); and via a spline fit through all available data types (red, 95% UI in pink). **I-R**: Box plots showing 1,000 draws of fixed effect coefficients in a multivariate regression fit to $log(beta)$.

7 Arizona: SEIR fit comparison

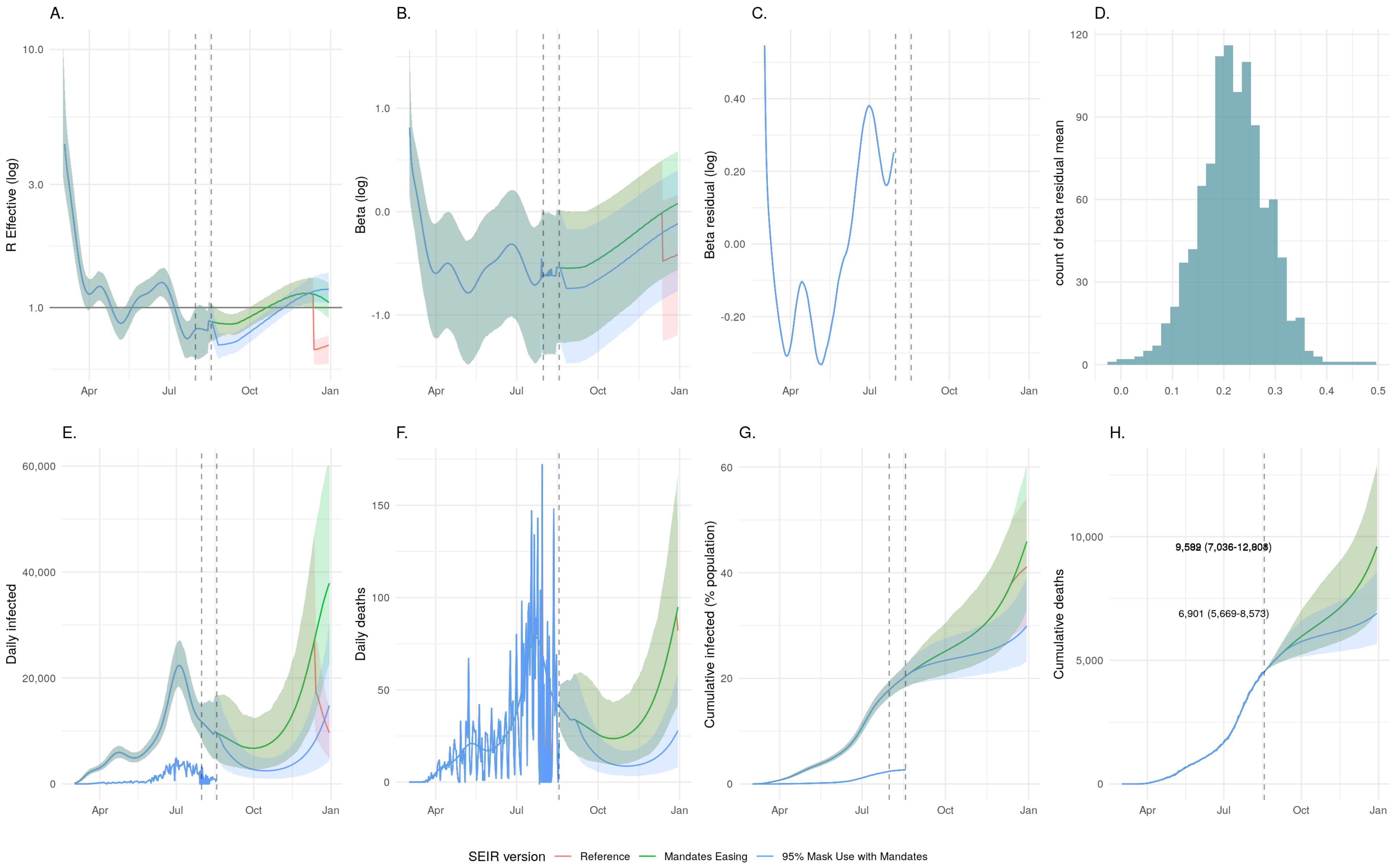

Arizona: SEIR fit comparison. A: predicted R effective for each model through December 31. **B**: predicted SEIR *beta* parameter. **C**: residual of predicted *beta* and the observed value calculated directly from infection data over time. **D**: histogram of residual values for *beta*. Panels A, B, C, and D are all displayed in log space, reflecting the space in which the SEIR model is fit. **E**: predicted daily infections from each model through December 31. **F**: predicted daily deaths from each model through December 31. **G**: predicted cumulative infections through December 31, as a proportion of the total population. **H**: predicted cumulative deaths through December 31. In panels E, F, G, and H, reported death and infections are plotted alongside model predictions in light blue.

8 Arizona: Covariate fits and regression coefficients

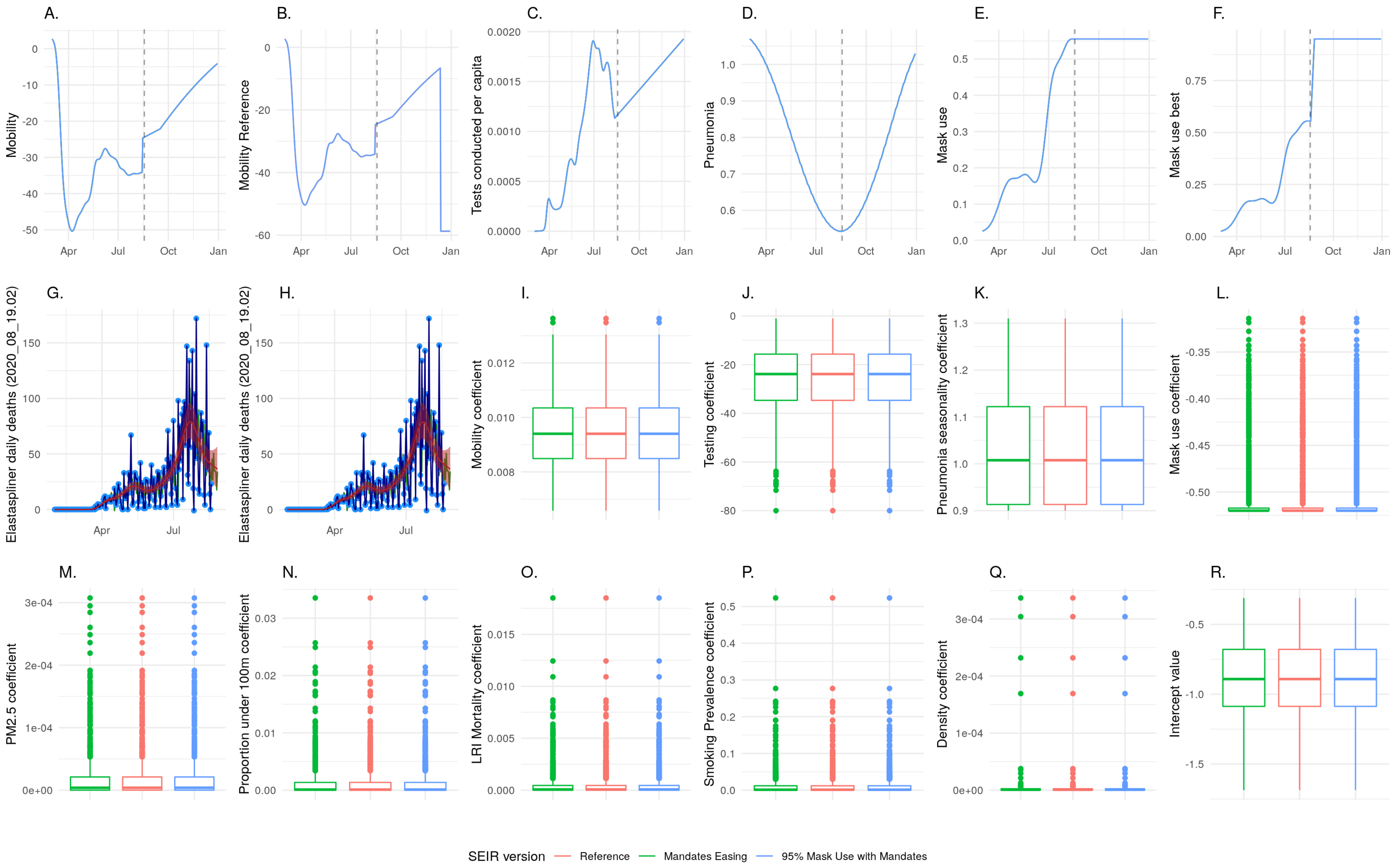

Arizona: Covariate fits and regression coefficients. **A-F**: Line plots showing predicted covariate time trends for **A)** mobility in the absence of additional mandates; **B)** mobility with additional mandates applied; **C)** diagnostic testing per capita; **D)** pneumonia seasonality; **E)** mask use per capita, and; **F)** mask use in a scenario where adherence increases to 95% of the population. **G-H**: COVID mortality data generated from reported daily deaths (blue); estimated based on reported hospitalizations (purple); estimated from reported cases (green); and via a spline fit through all available data types (red, 95% UI in pink). **I-R**: Box plots showing 1,000 draws of fixed effect coefficients in a multivariate regression fit to $log(beta)$.

9 Arkansas: SEIR fit comparison

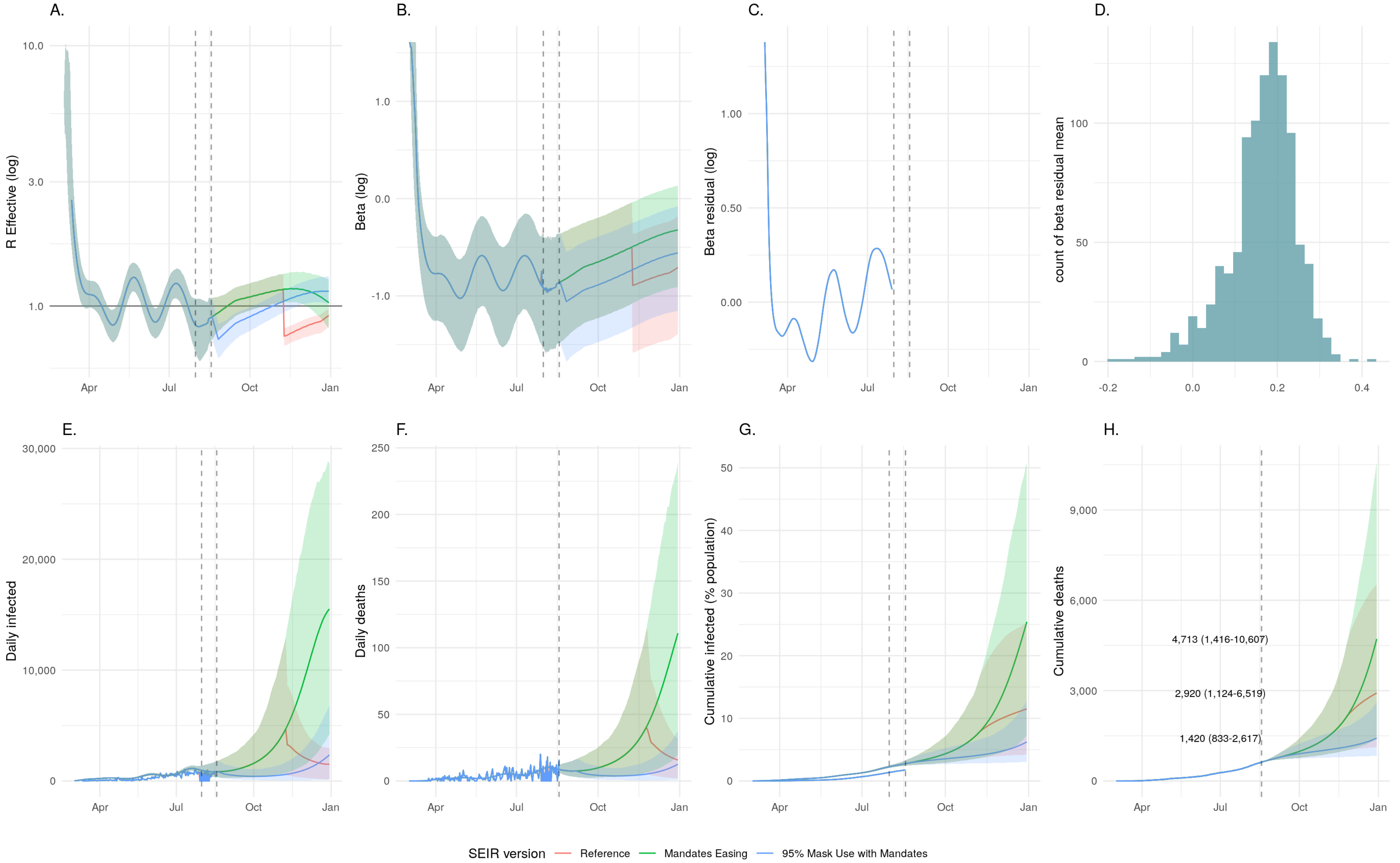

Arkansas: SEIR fit comparison. A: predicted R effective for each model through December 31. **B**: predicted SEIR *beta* parameter. **C**: residual of predicted *beta* and the observed value calculated directly from infection data over time. **D**: histogram of residual values for *beta*. Panels A, B, C, and D are all displayed in log space, reflecting the space in which the SEIR model is fit. **E**: predicted daily infections from each model through December 31. **F**: predicted daily deaths from each model through December 31. **G**: predicted cumulative infections through December 31, as a proportion of the total population. **H**: predicted cumulative deaths through December 31. In panels E, F, G, and H, reported death and infections are plotted alongside model predictions in light blue.

10 Arkansas: Covariate fits and regression coefficients

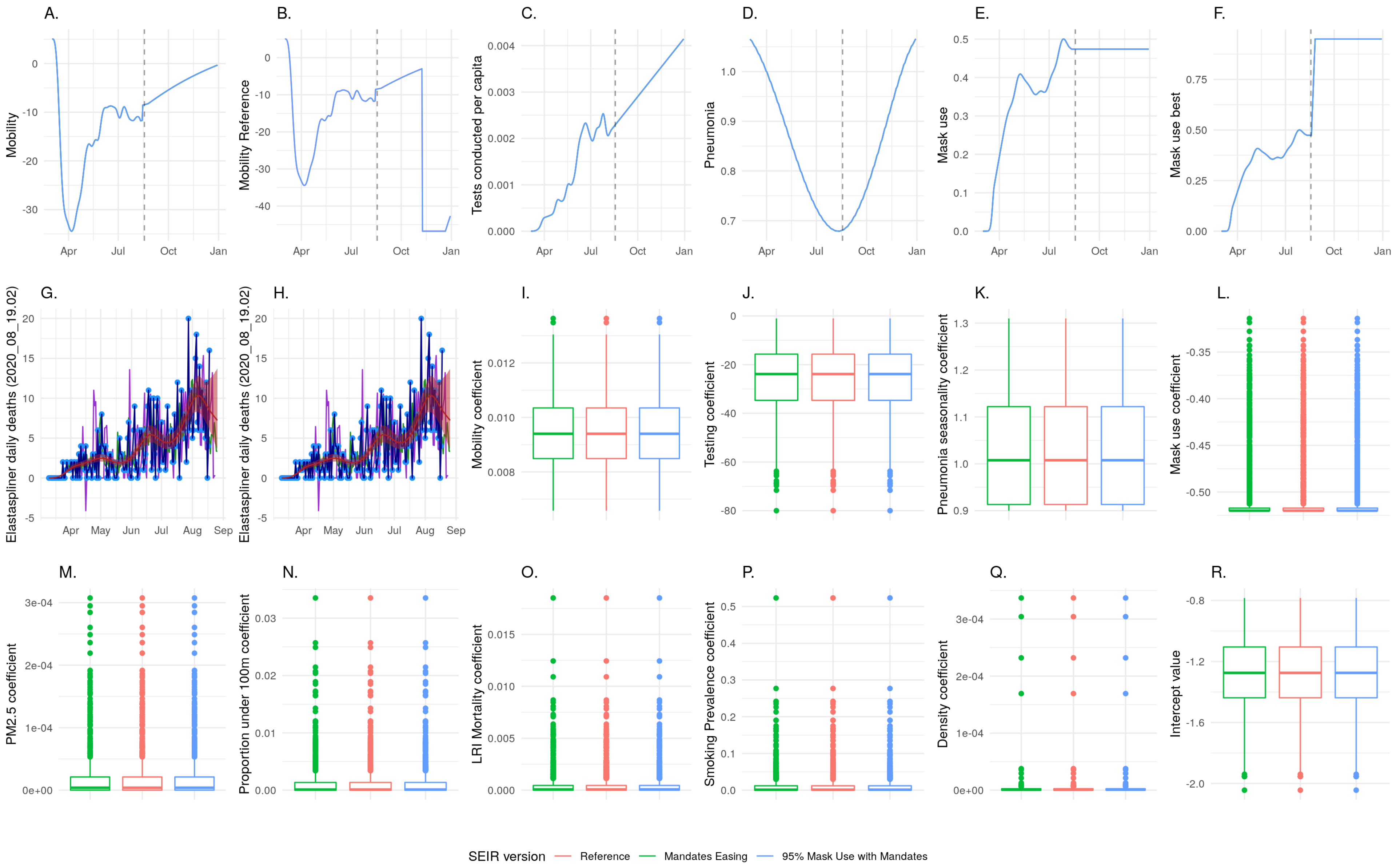

Arkansas: Covariate fits and regression coefficients. A-F: Line plots showing predicted covariate time trends for **A)** mobility in the absence of additional mandates; **B)** mobility with additional mandates applied; **C)** diagnostic testing per capita; **D)** pneumonia seasonality; **E)** mask use per capita, and; **F)** mask use in a scenario where adherence increases to 95% of the population. **G-H**: COVID mortality data generated from reported daily deaths (blue); estimated based on reported hospitalizations (purple); estimated from reported cases (green); and via a spline fit through all available data types (red, 95% UI in pink). **I-R**: Box plots showing 1,000 draws of fixed effect coefficients in a multivariate regression fit to $log(beta)$.

11 California: SEIR fit comparison

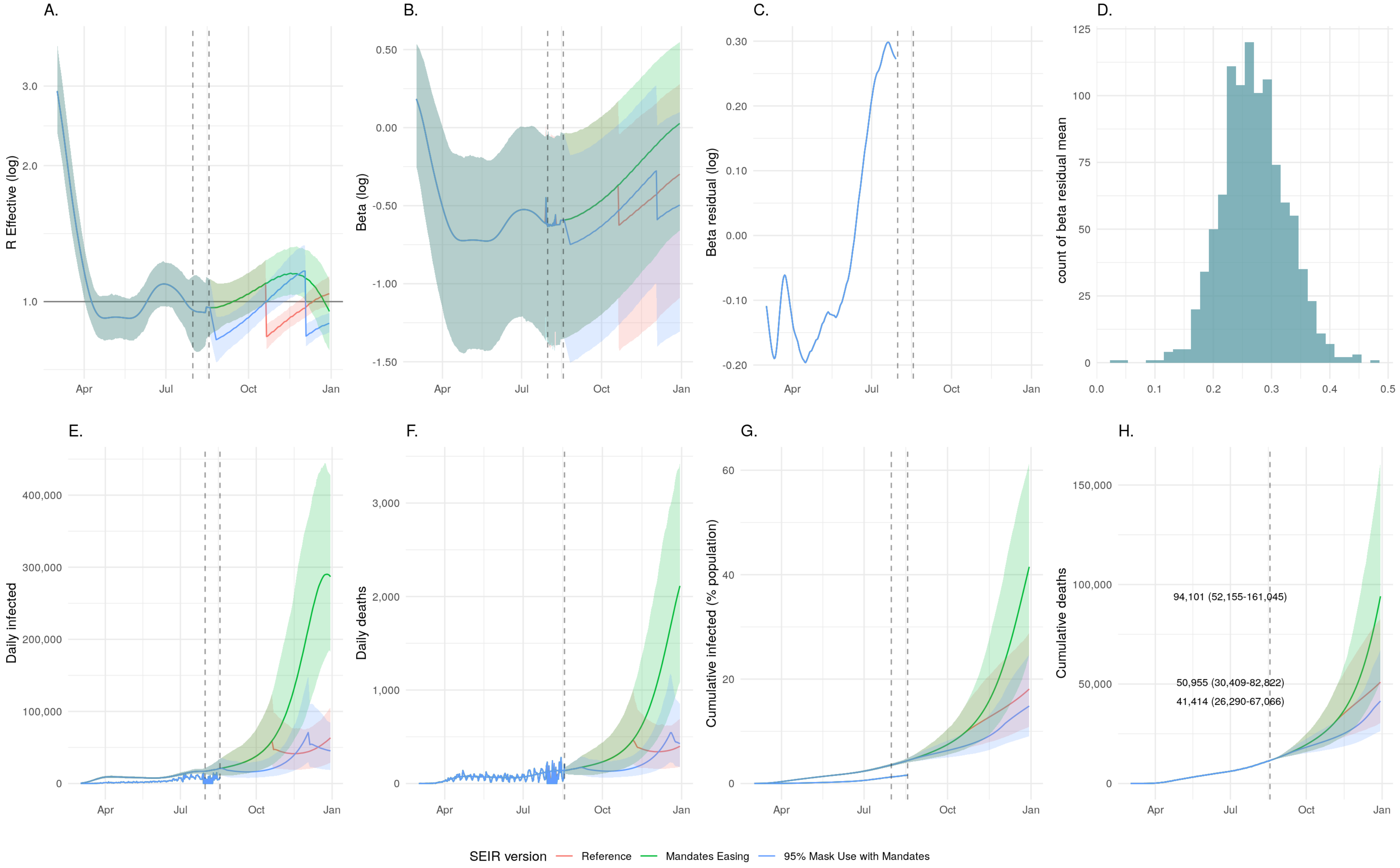

California: SEIR fit comparison. A: predicted R effective for each model through December 31. **B**: predicted SEIR *beta* parameter. **C**: residual of predicted *beta* and the observed value calculated directly from infection data over time. **D**: histogram of residual values for *beta*. Panels A, B, C, and D are all displayed in log space, reflecting the space in which the SEIR model is fit. **E**: predicted daily infections from each model through December 31. **F**: predicted daily deaths from each model through December 31. **G**: predicted cumulative infections through December 31, as a proportion of the total population. **H**: predicted cumulative deaths through December 31. In panels E, F, G, and H, reported death and infections are plotted alongside model predictions in light blue.

12 California: Covariate fits and regression coefficients

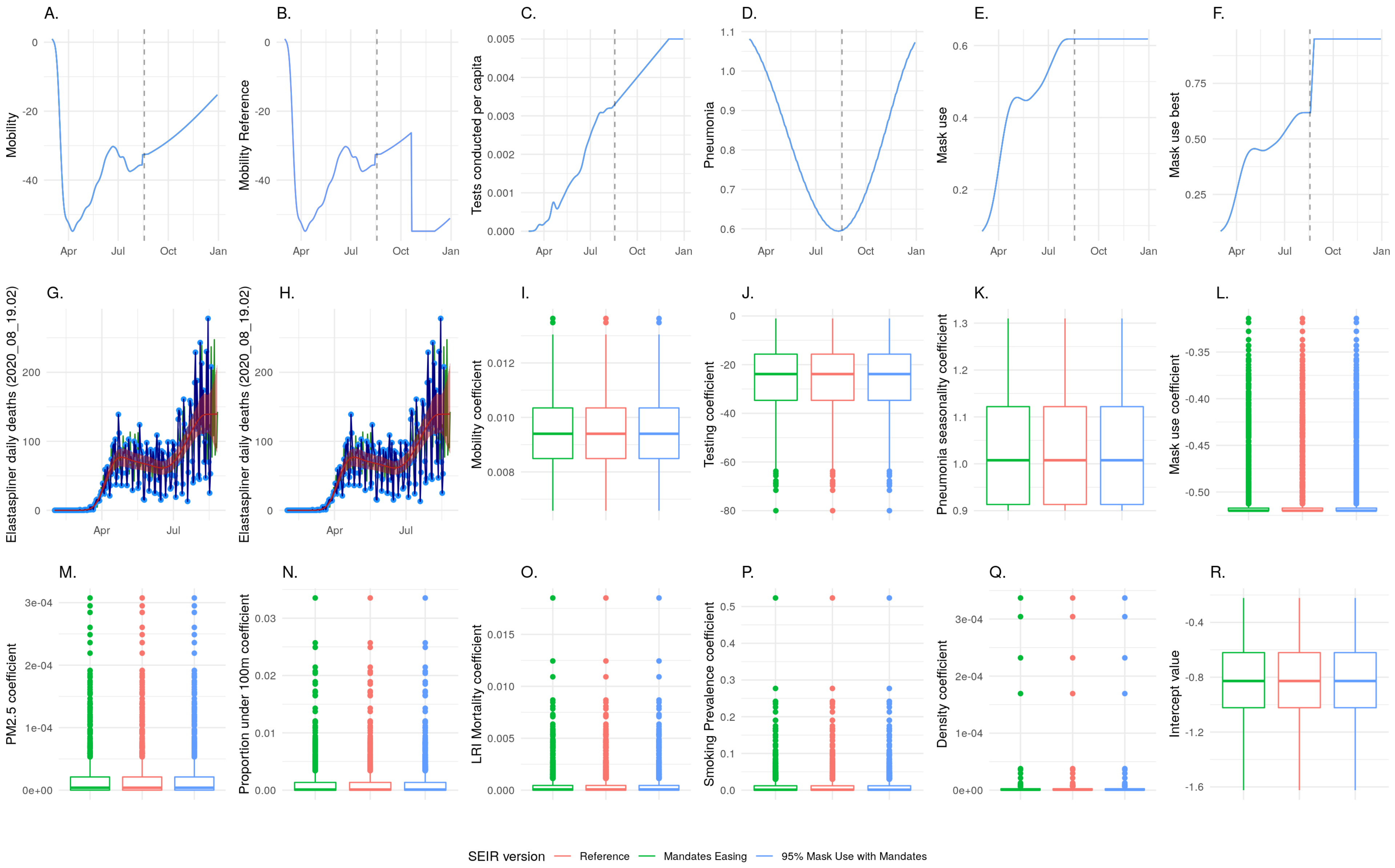

California: Covariate fits and regression coefficients. **A-F**: Line plots showing predicted covariate time trends for **A)** mobility in the absence of additional mandates; **B)** mobility with additional mandates applied; **C)** diagnostic testing per capita; **D)** pneumonia seasonality; **E)** mask use per capita, and; **F)** mask use in a scenario where adherence increases to 95% of the population. **G-H**: COVID mortality data generated from reported daily deaths (blue); estimated based on reported hospitalizations (purple); estimated from reported cases (green); and via a spline fit through all available data types (red, 95% UI in pink). **I-R**: Box plots showing 1,000 draws of fixed effect coefficients in a multivariate regression fit to $log(beta)$.

13 Colorado: SEIR fit comparison

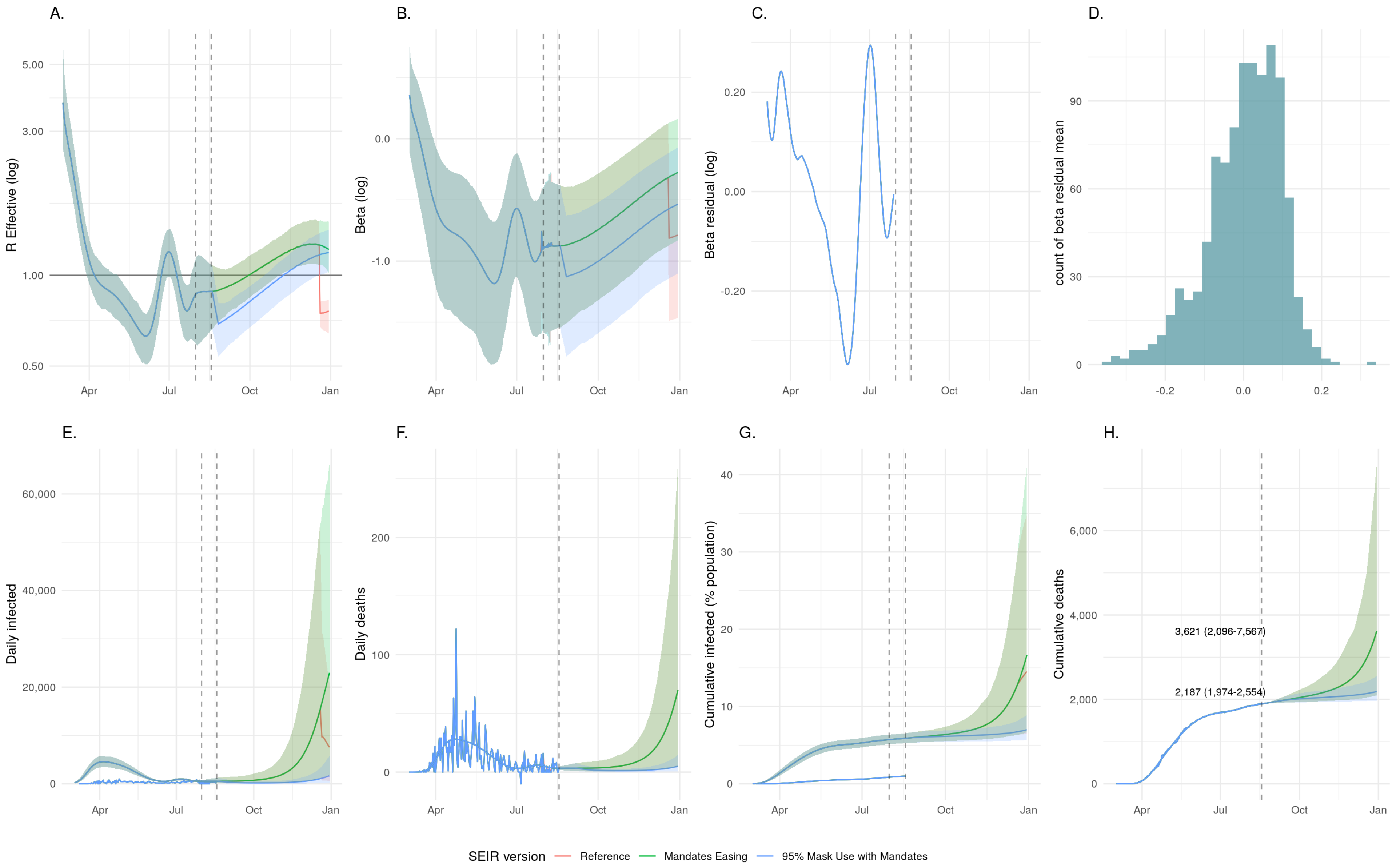

Colorado: SEIR fit comparison. **A**: predicted R effective for each model through December 31. **B**: predicted SEIR *beta* parameter. **C**: residual of predicted *beta* and the observed value calculated directly from infection data over time. **D**: histogram of residual values for *beta*. Panels A, B, C, and D are all displayed in log space, reflecting the space in which the SEIR model is fit. **E**: predicted daily infections from each model through December 31. **F**: predicted daily deaths from each model through December 31. **G**: predicted cumulative infections through December 31, as a proportion of the total population. **H**: predicted cumulative deaths through December 31. In panels E, F, G, and H, reported death and infections are plotted alongside model predictions in light blue.

14 Colorado: Covariate fits and regression coefficients

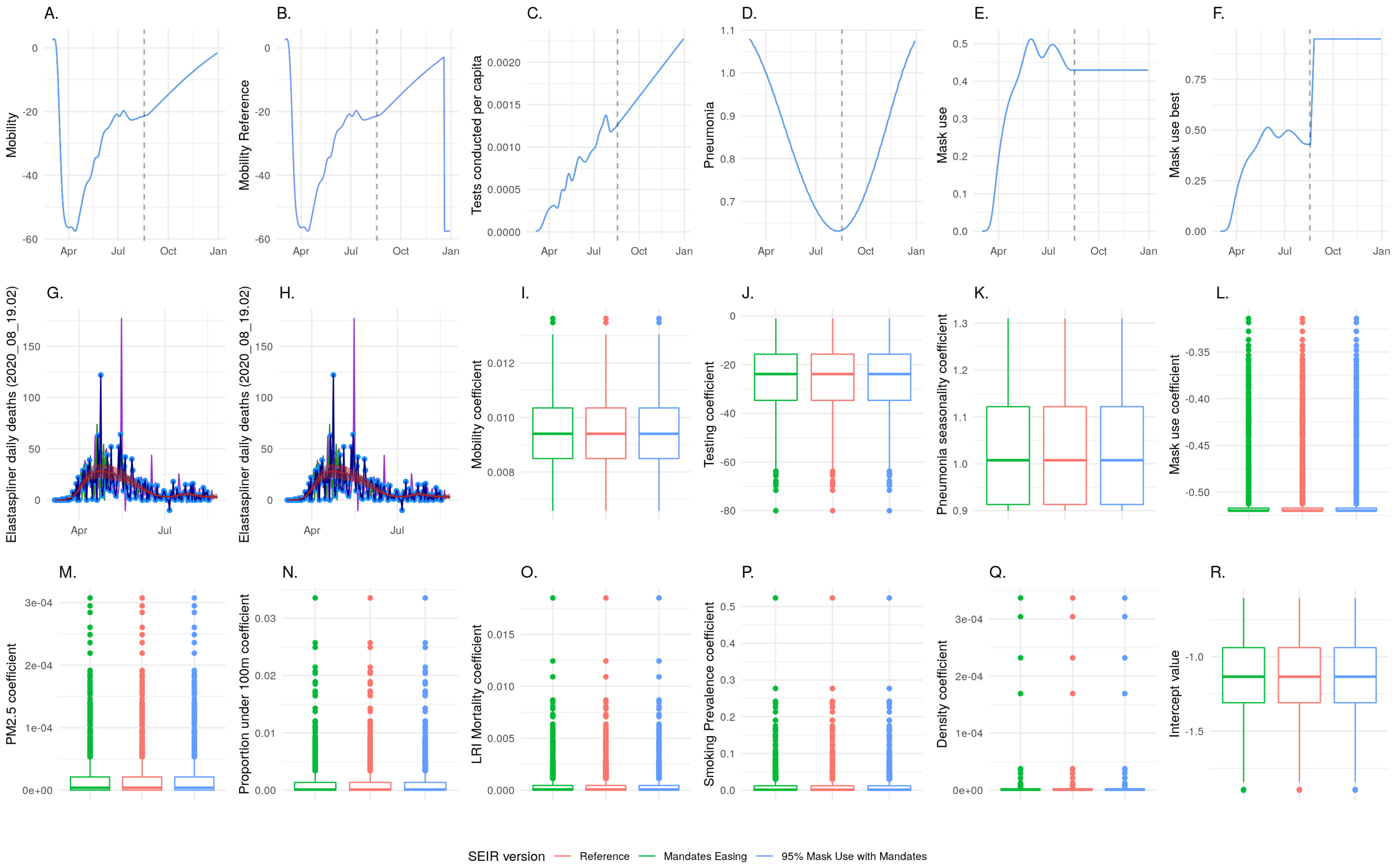

Colorado: Covariate fits and regression coefficients. A-F: Line plots showing predicted covariate time trends for **A)** mobility in the absence of additional mandates; **B)** mobility with additional mandates applied; **C)** diagnostic testing per capita; **D)** pneumonia seasonality; **E)** mask use per capita, and; **F)** mask use in a scenario where adherence increases to 95% of the population. **G-H**: COVID mortality data generated from reported daily deaths (blue); estimated based on reported hospitalizations (purple); estimated from reported cases (green); and via a spline fit through all available data types (red, 95% UI in pink). **I-R**: Box plots showing 1,000 draws of fixed effect coefficients in a multivariate regression fit to $log(beta)$.

15 Connecticut: SEIR fit comparison

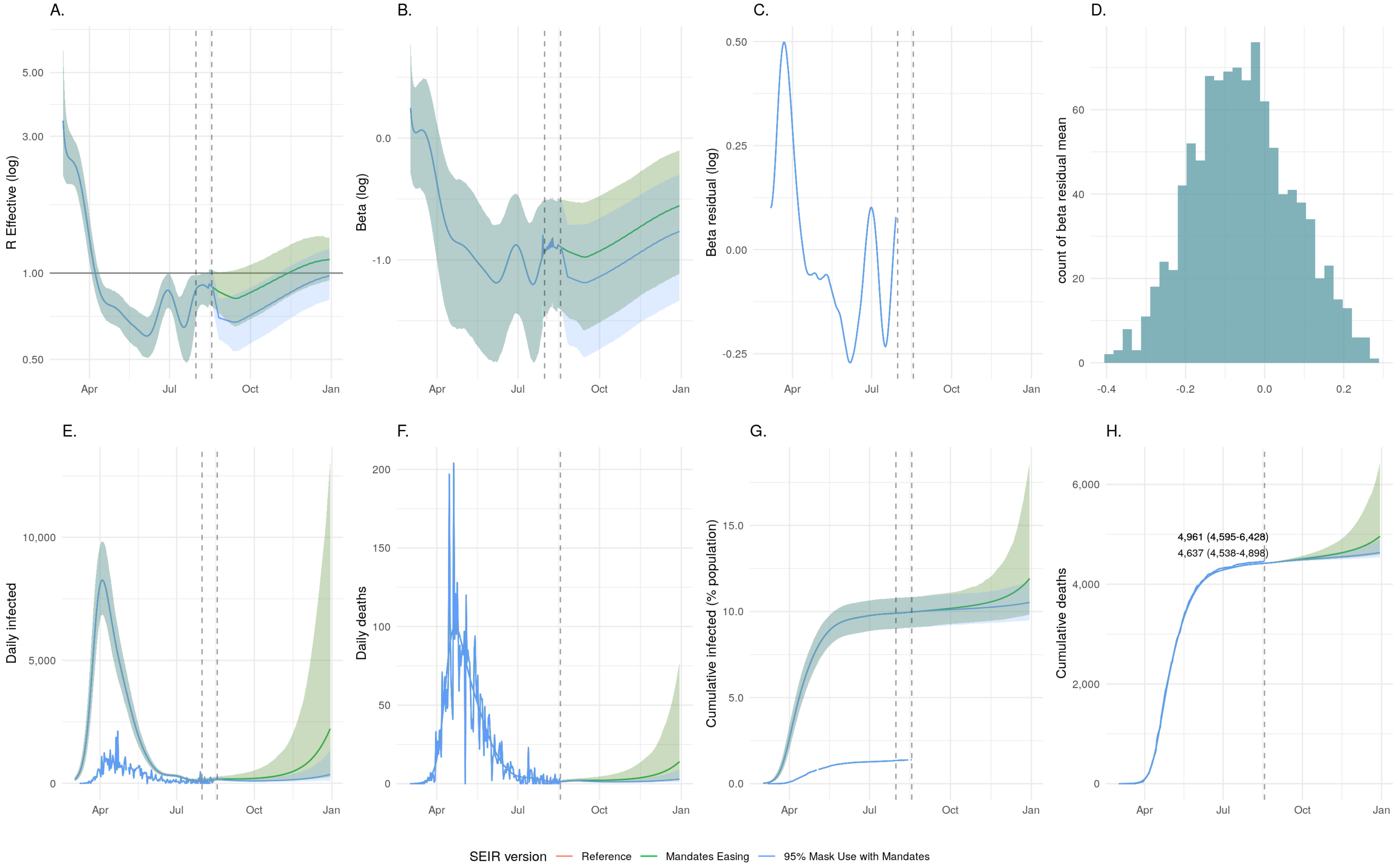

Connecticut: SEIR fit comparison. A: predicted R effective for each model through December 31. **B**: predicted SEIR *beta* parameter. **C**: residual of predicted *beta* and the observed value calculated directly from infection data over time. **D**: histogram of residual values for *beta*. Panels A, B, C, and D are all displayed in log space, reflecting the space in which the SEIR model is fit. **E**: predicted daily infections from each model through December 31. **F**: predicted daily deaths from each model through December 31. **G**: predicted cumulative infections through December 31, as a proportion of the total population. **H**: predicted cumulative deaths through December 31. In panels E, F, G, and H, reported death and infections are plotted alongside model predictions in light blue.

16 Connecticut: Covariate fits and regression coefficients

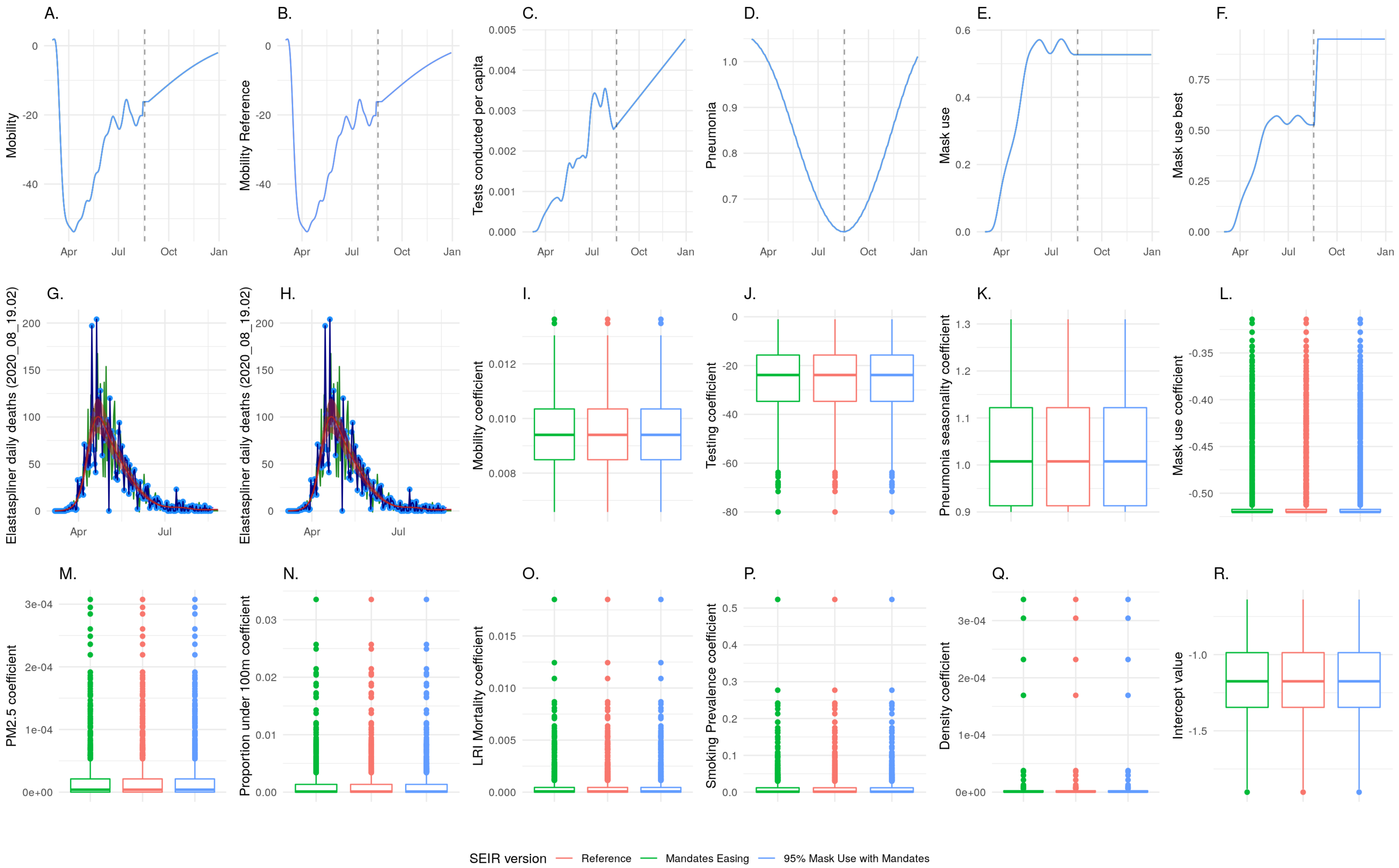

Connecticut: Covariate fits and regression coefficients. **A-F**: Line plots showing predicted covariate time trends for **A)** mobility in the absence of additional mandates; **B)** mobility with additional mandates applied; **C)** diagnostic testing per capita; **D)** pneumonia seasonality; **E)** mask use per capita, and; **F)** mask use in a scenario where adherence increases to 95% of the population. **G-H**: COVID mortality data generated from reported daily deaths (blue); estimated based on reported hospitalizations (purple); estimated from reported cases (green); and via a spline fit through all available data types (red, 95% UI in pink). **I-R**: Box plots showing 1,000 draws of fixed effect coefficients in a multivariate regression fit to $log(beta)$.

17 Delaware: SEIR fit comparison

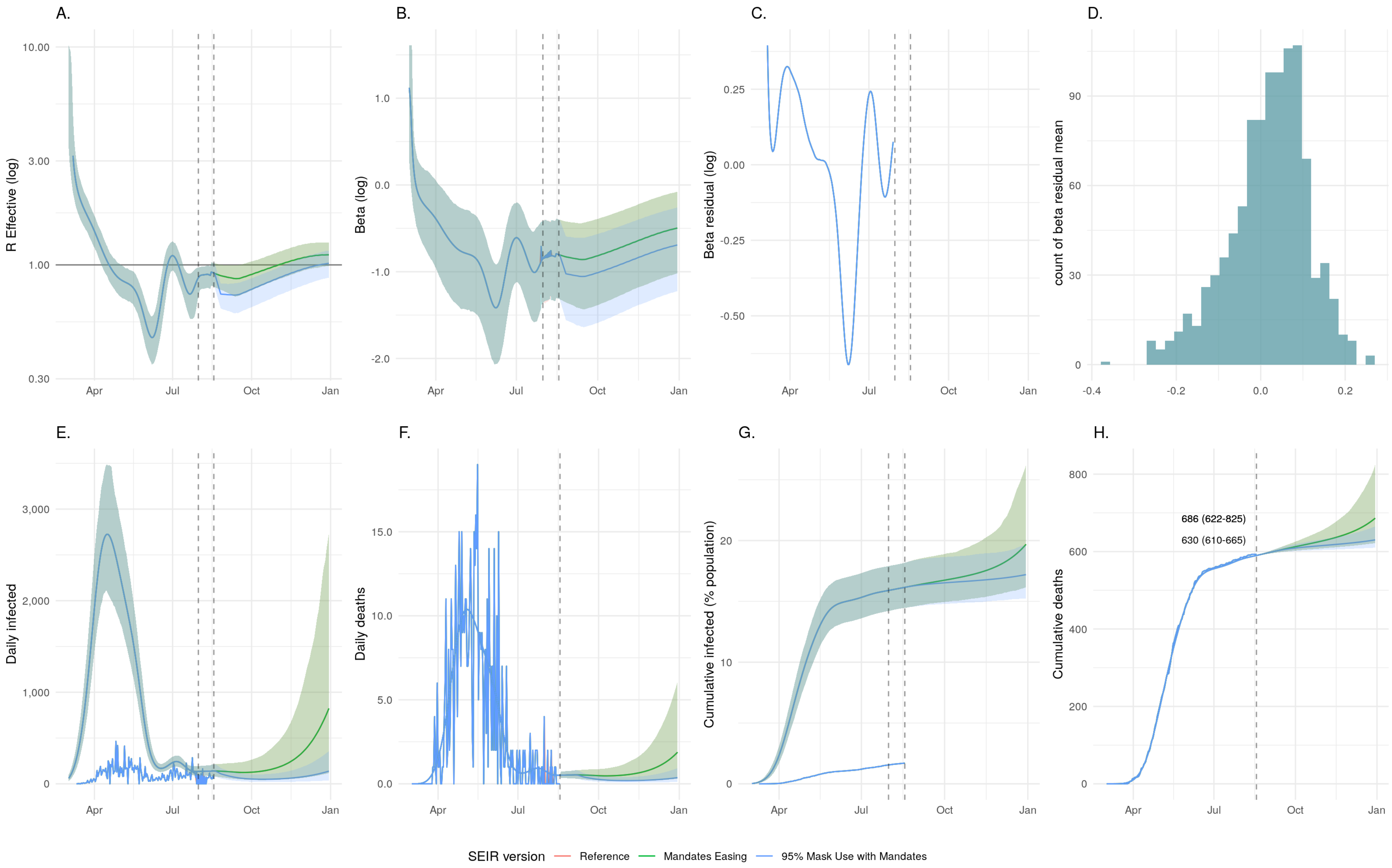

Delaware: SEIR fit comparison. **A**: predicted R effective for each model through December 31. **B**: predicted SEIR *beta* parameter. **C**: residual of predicted *beta* and the observed value calculated directly from infection data over time. **D**: histogram of residual values for *beta*. Panels A, B, C, and D are all displayed in log space, reflecting the space in which the SEIR model is fit. **E**: predicted daily infections from each model through December 31. **F**: predicted daily deaths from each model through December 31. **G**: predicted cumulative infections through December 31, as a proportion of the total population. **H**: predicted cumulative deaths through December 31. In panels E, F, G, and H, reported death and infections are plotted alongside model predictions in light blue.

18 Delaware: Covariate fits and regression coefficients

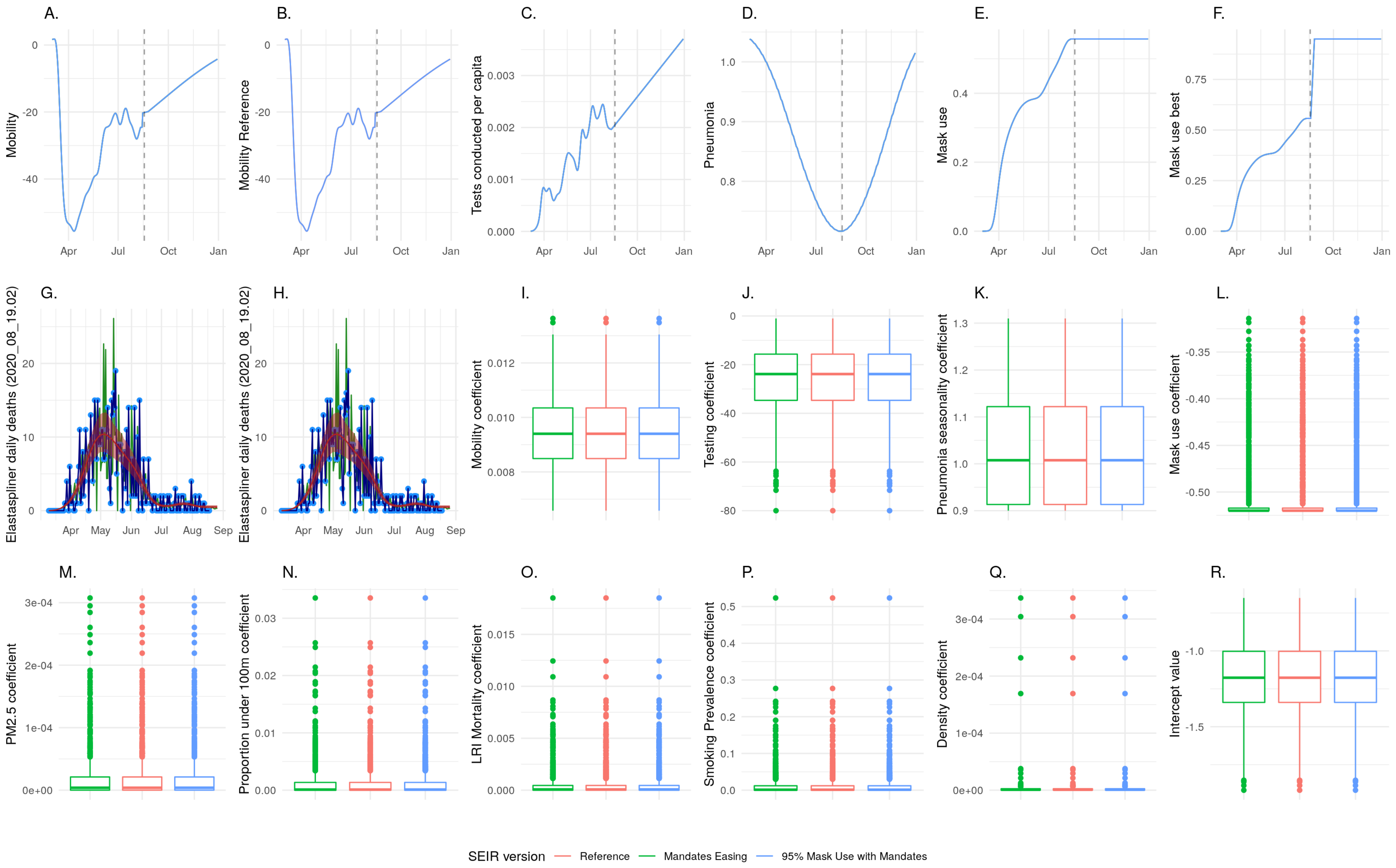

Delaware: Covariate fits and regression coefficients. A-F: Line plots showing predicted covariate time trends for **A)** mobility in the absence of additional mandates; **B)** mobility with additional mandates applied; **C)** diagnostic testing per capita; **D)** pneumonia seasonality; **E)** mask use per capita, and; **F)** mask use in a scenario where adherence increases to 95% of the population. **G-H**: COVID mortality data generated from reported daily deaths (blue); estimated based on reported hospitalizations (purple); estimated from reported cases (green); and via a spline fit through all available data types (red, 95% UI in pink). **I-R**: Box plots showing 1,000 draws of fixed effect coefficients in a multivariate regression fit to $log(beta)$.

19 District of Columbia: SEIR fit comparison

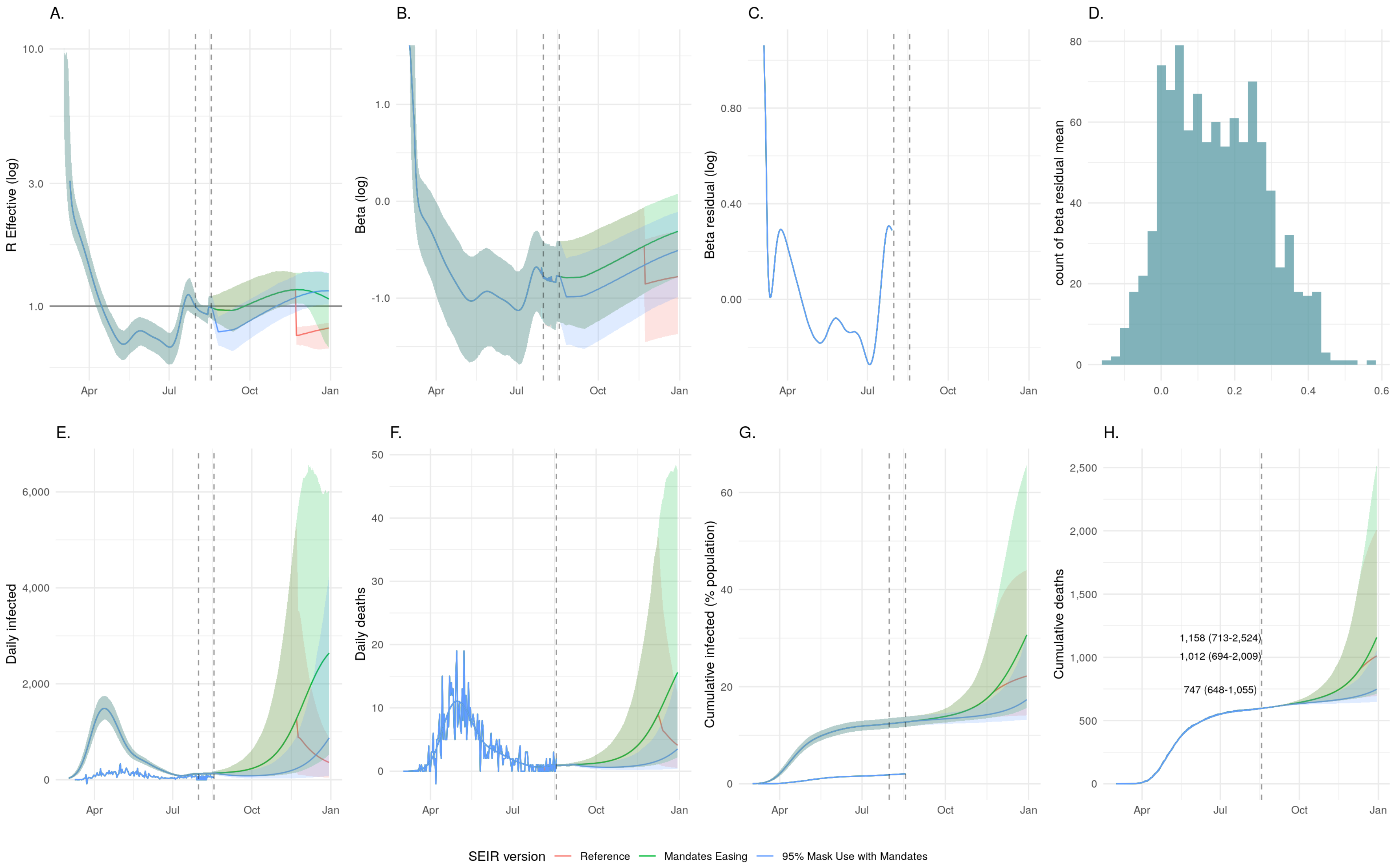

District of Columbia: SEIR fit comparison. **A**: predicted R effective for each model through December 31. **B**: predicted SEIR *beta* parameter. **C**: residual of predicted *beta* and the observed value calculated directly from infection data over time. **D**: histogram of residual values for *beta*. Panels A, B, C, and D are all displayed in log space, reflecting the space in which the SEIR model is fit. **E**: predicted daily infections from each model through December 31. **F**: predicted daily deaths from each model through December 31. **G**: predicted cumulative infections through December 31, as a proportion of the total population. **H**: predicted cumulative deaths through December 31. In panels E, F, G, and H, reported death and infections are plotted alongside model predictions in light blue.

20 District of Columbia: Covariate fits and regression coefficients

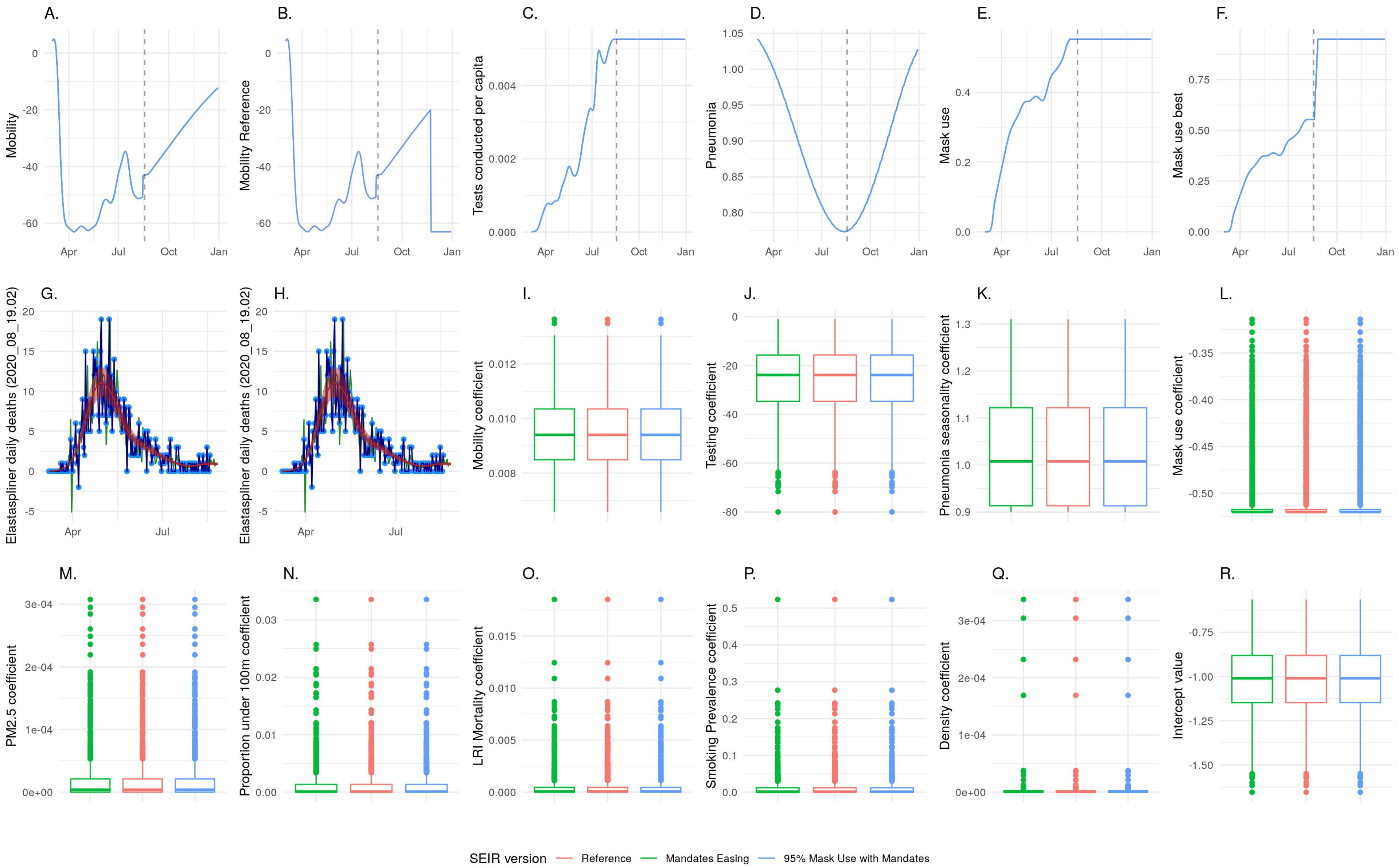

District of Columbia: Covariate fits and regression coefficients. A-F: Line plots showing predicted covariate time trends for **A)** mobility in the absence of additional mandates; **B)** mobility with additional mandates applied; **C)** diagnostic testing per capita; **D)** pneumonia seasonality; **E)** mask use per capita, and; **F)** mask use in a scenario where adherence increases to 95% of the population. **G-H**: COVID mortality data generated from reported daily deaths (blue); estimated based on reported hospitalizations (purple); estimated from reported cases (green); and via a spline fit through all available data types (red, 95% UI in pink). **I-R**: Box plots showing 1,000 draws of fixed effect coefficients in a multivariate regression fit to $log(beta)$.

21 Florida: SEIR fit comparison

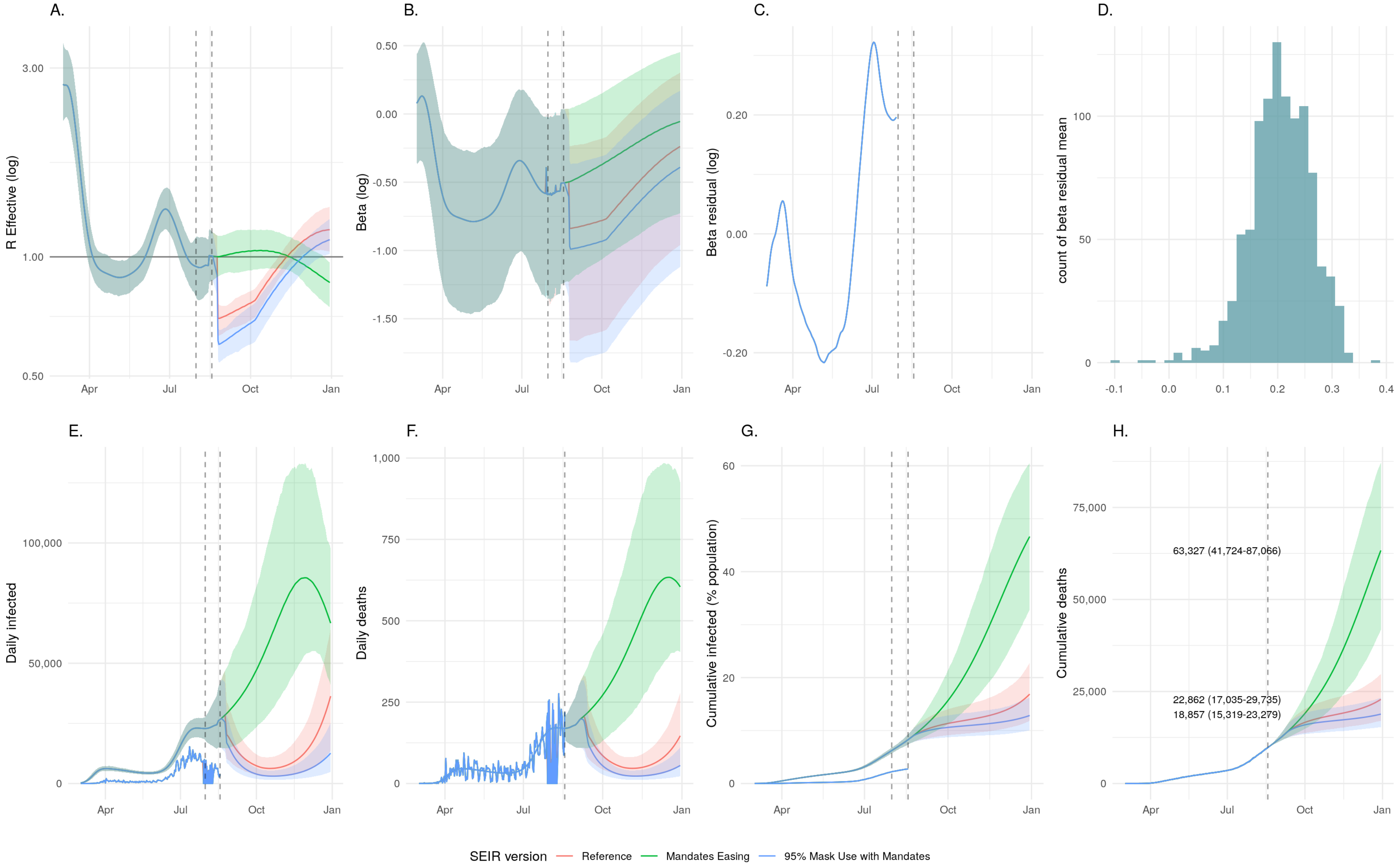

Florida: SEIR fit comparison. A: predicted R effective for each model through December 31. **B**: predicted SEIR *beta* parameter. **C**: residual of predicted *beta* and the observed value calculated directly from infection data over time. **D**: histogram of residual values for *beta*. Panels A, B, C, and D are all displayed in log space, reflecting the space in which the SEIR model is fit. **E**: predicted daily infections from each model through December 31. **F**: predicted daily deaths from each model through December 31. **G**: predicted cumulative infections through December 31, as a proportion of the total population. **H**: predicted cumulative deaths through December 31. In panels E, F, G, and H, reported death and infections are plotted alongside model predictions in light blue.

22 Florida: Covariate fits and regression coefficients

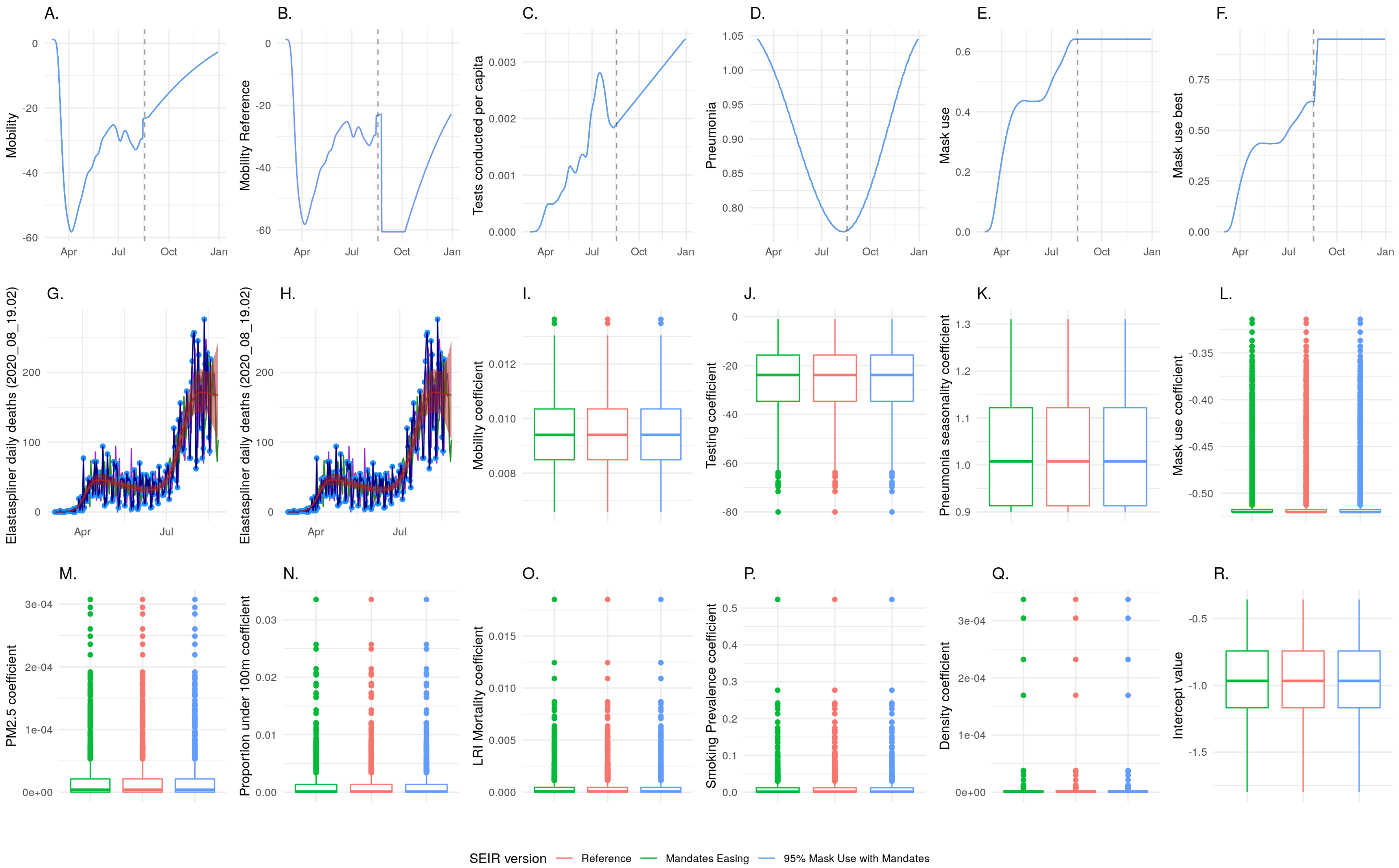

Florida: Covariate fits and regression coefficients. **A-F**: Line plots showing predicted covariate time trends for **A)** mobility in the absence of additional mandates; **B)** mobility with additional mandates applied; **C)** diagnostic testing per capita; **D)** pneumonia seasonality; **E)** mask use per capita, and; **F)** mask use in a scenario where adherence increases to 95% of the population. **G-H**: COVID mortality data generated from reported daily deaths (blue); estimated based on reported hospitalizations (purple); estimated from reported cases (green); and via a spline fit through all available data types (red, 95% UI in pink). **I-R**: Box plots showing 1,000 draws of fixed effect coefficients in a multivariate regression fit to $log(beta)$.

23 Georgia: SEIR fit comparison

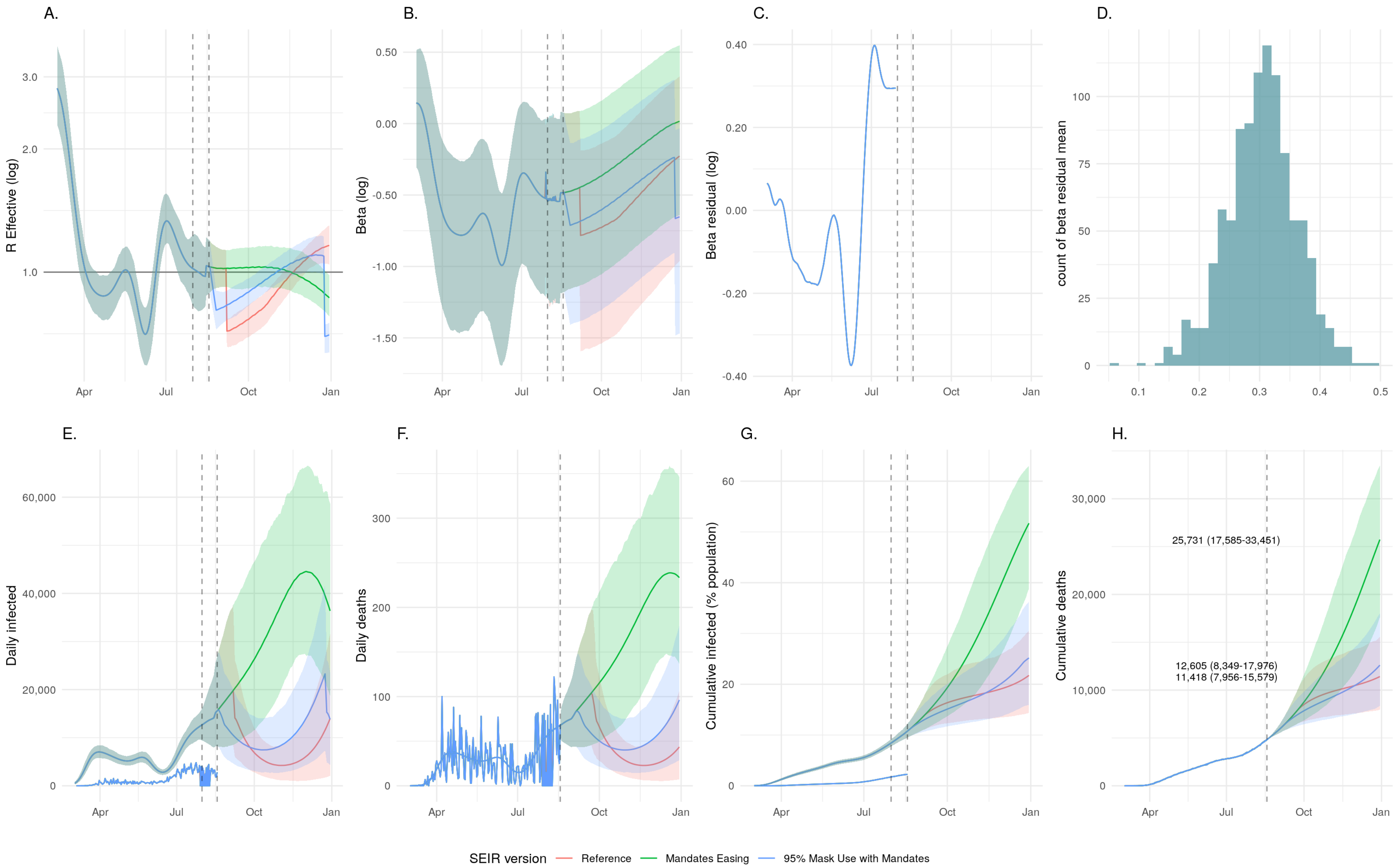

Georgia: SEIR fit comparison. A: predicted R effective for each model through December 31. **B**: predicted SEIR *beta* parameter. **C**: residual of predicted *beta* and the observed value calculated directly from infection data over time. **D**: histogram of residual values for *beta*. Panels A, B, C, and D are all displayed in log space, reflecting the space in which the SEIR model is fit. **E**: predicted daily infections from each model through December 31. **F**: predicted daily deaths from each model through December 31. **G**: predicted cumulative infections through December 31, as a proportion of the total population. **H**: predicted cumulative deaths through December 31. In panels E, F, G, and H, reported death and infections are plotted alongside model predictions in light blue.

24 Georgia: Covariate fits and regression coefficients

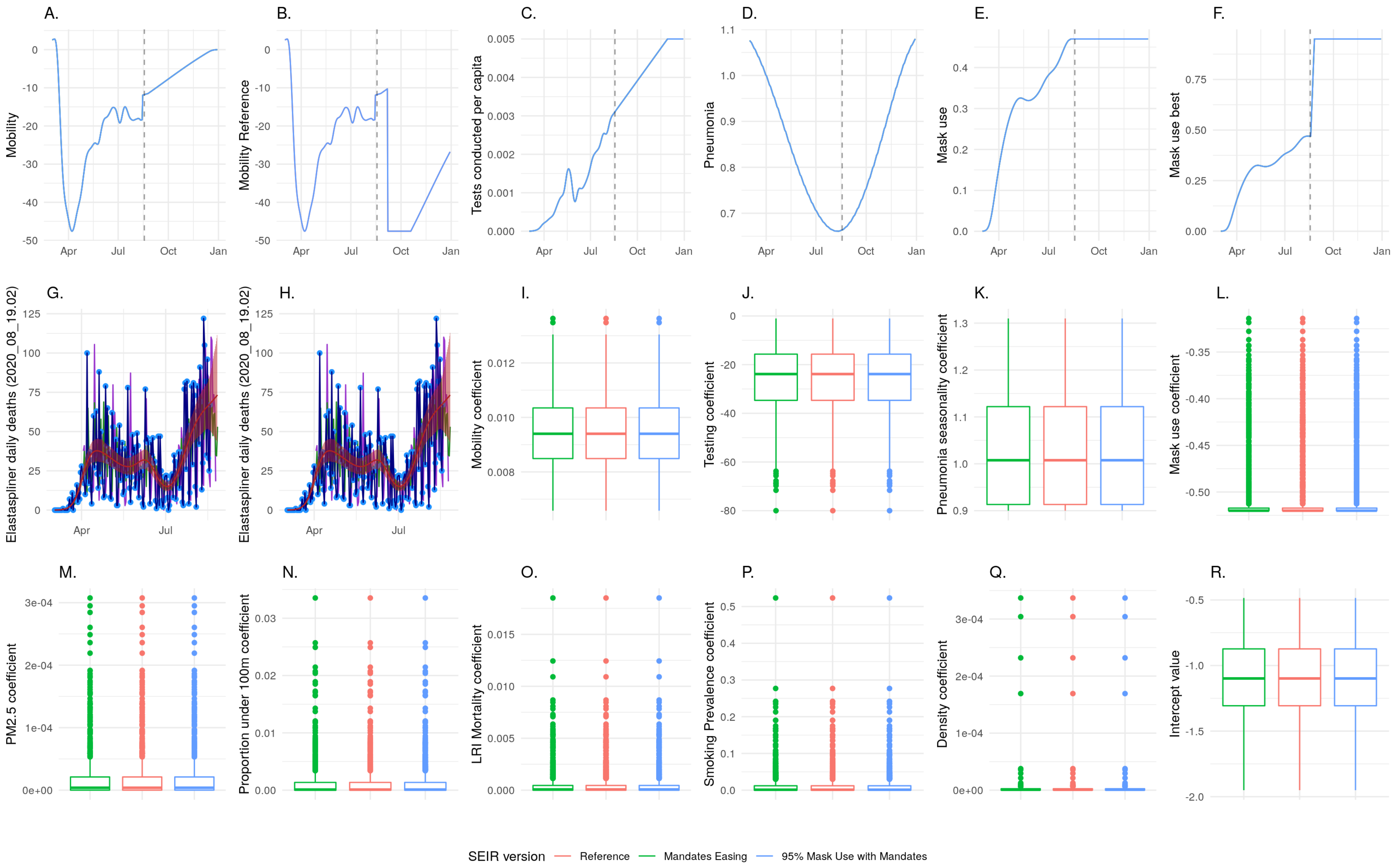

Georgia: Covariate fits and regression coefficients. A-F: Line plots showing predicted covariate time trends for **A)** mobility in the absence of additional mandates; **B)** mobility with additional mandates applied; **C)** diagnostic testing per capita; **D)** pneumonia seasonality; **E)** mask use per capita, and; **F)** mask use in a scenario where adherence increases to 95% of the population. **G-H**: COVID mortality data generated from reported daily deaths (blue); estimated based on reported hospitalizations (purple); estimated from reported cases (green); and via a spline fit through all available data types (red, 95% UI in pink). **I-R**: Box plots showing 1,000 draws of fixed effect coefficients in a multivariate regression fit to $log(beta)$.

25 Hawaii: SEIR fit comparison

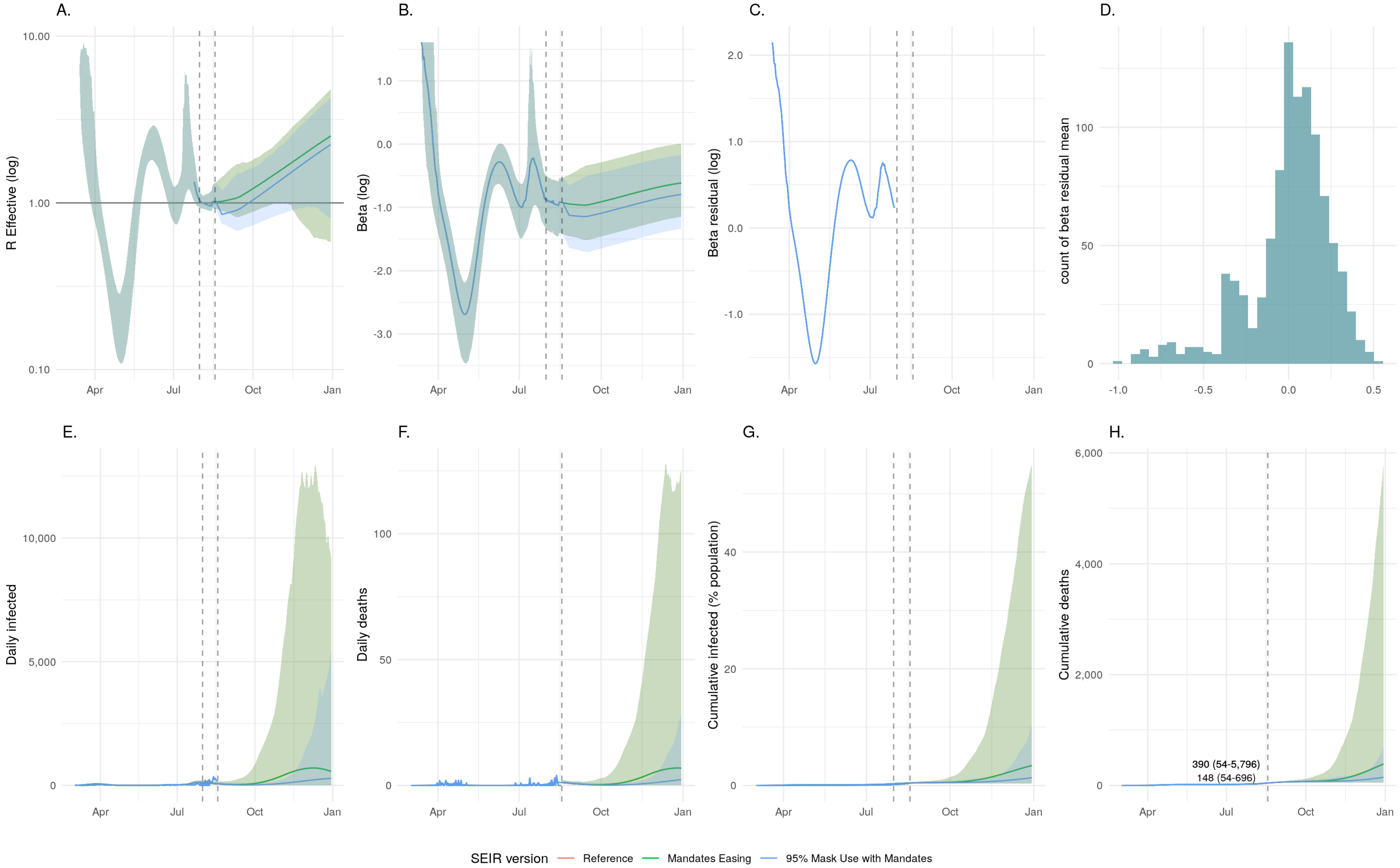

Hawaii: SEIR fit comparison. A: predicted R effective for each model through December 31. **B**: predicted SEIR *beta* parameter. **C**: residual of predicted *beta* and the observed value calculated directly from infection data over time. **D**: histogram of residual values for *beta*. Panels A, B, C, and D are all displayed in log space, reflecting the space in which the SEIR model is fit. **E**: predicted daily infections from each model through December 31. **F**: predicted daily deaths from each model through December 31. **G**: predicted cumulative infections through December 31, as a proportion of the total population. **H**: predicted cumulative deaths through December 31. In panels E, F, G, and H, reported death and infections are plotted alongside model predictions in light blue.

26 Hawaii: Covariate fits and regression coefficients

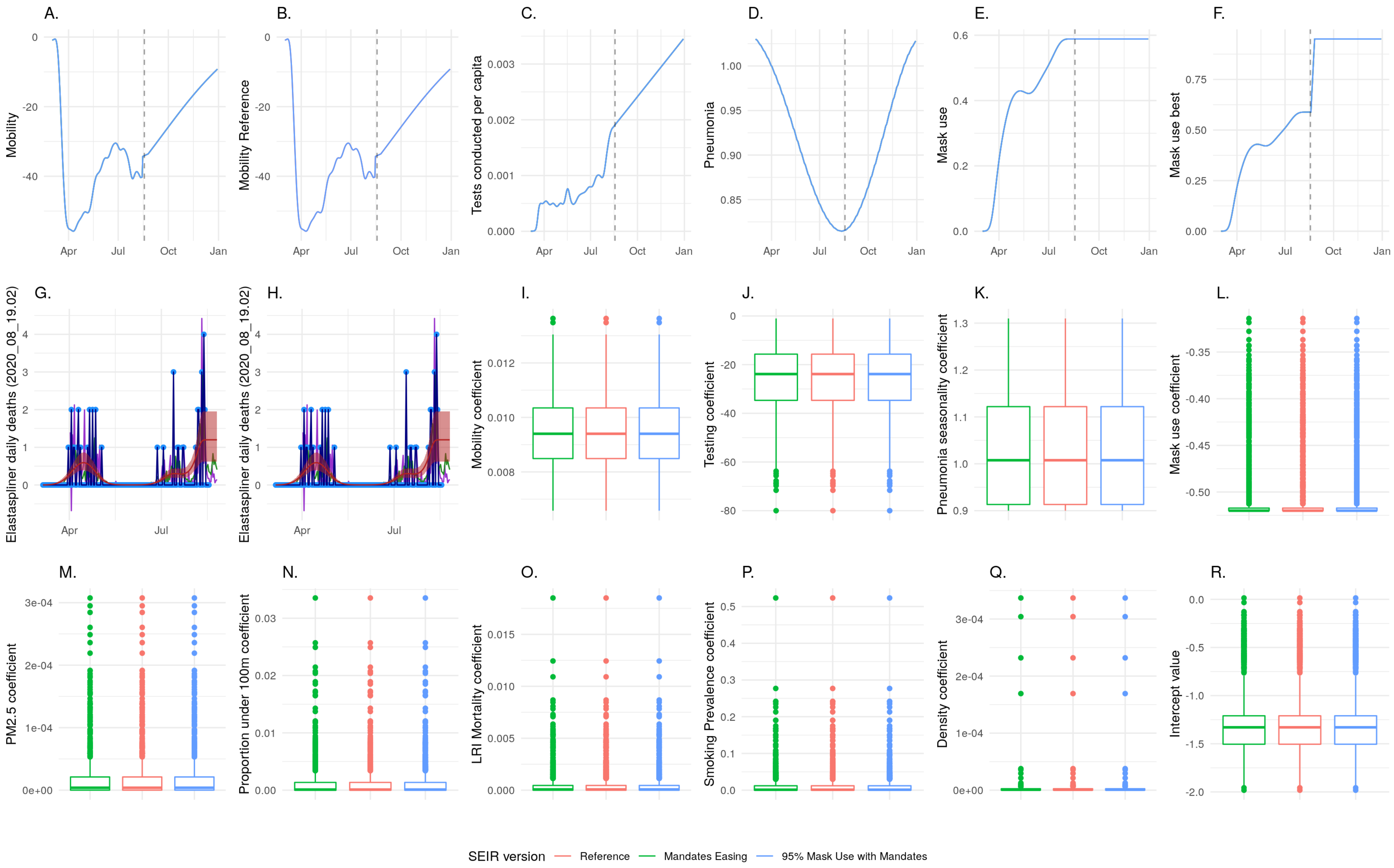

Hawaii: Covariate fits and regression coefficients. **A-F**: Line plots showing predicted covariate time trends for **A)** mobility in the absence of additional mandates; **B)** mobility with additional mandates applied; **C)** diagnostic testing per capita; **D)** pneumonia seasonality; **E)** mask use per capita, and; **F)** mask use in a scenario where adherence increases to 95% of the population. **G-H**: COVID mortality data generated from reported daily deaths (blue); estimated based on reported hospitalizations (purple); estimated from reported cases (green); and via a spline fit through all available data types (red, 95% UI in pink). **I-R**: Box plots showing 1,000 draws of fixed effect coefficients in a multivariate regression fit to $log(beta)$.

27 Idaho: SEIR fit comparison

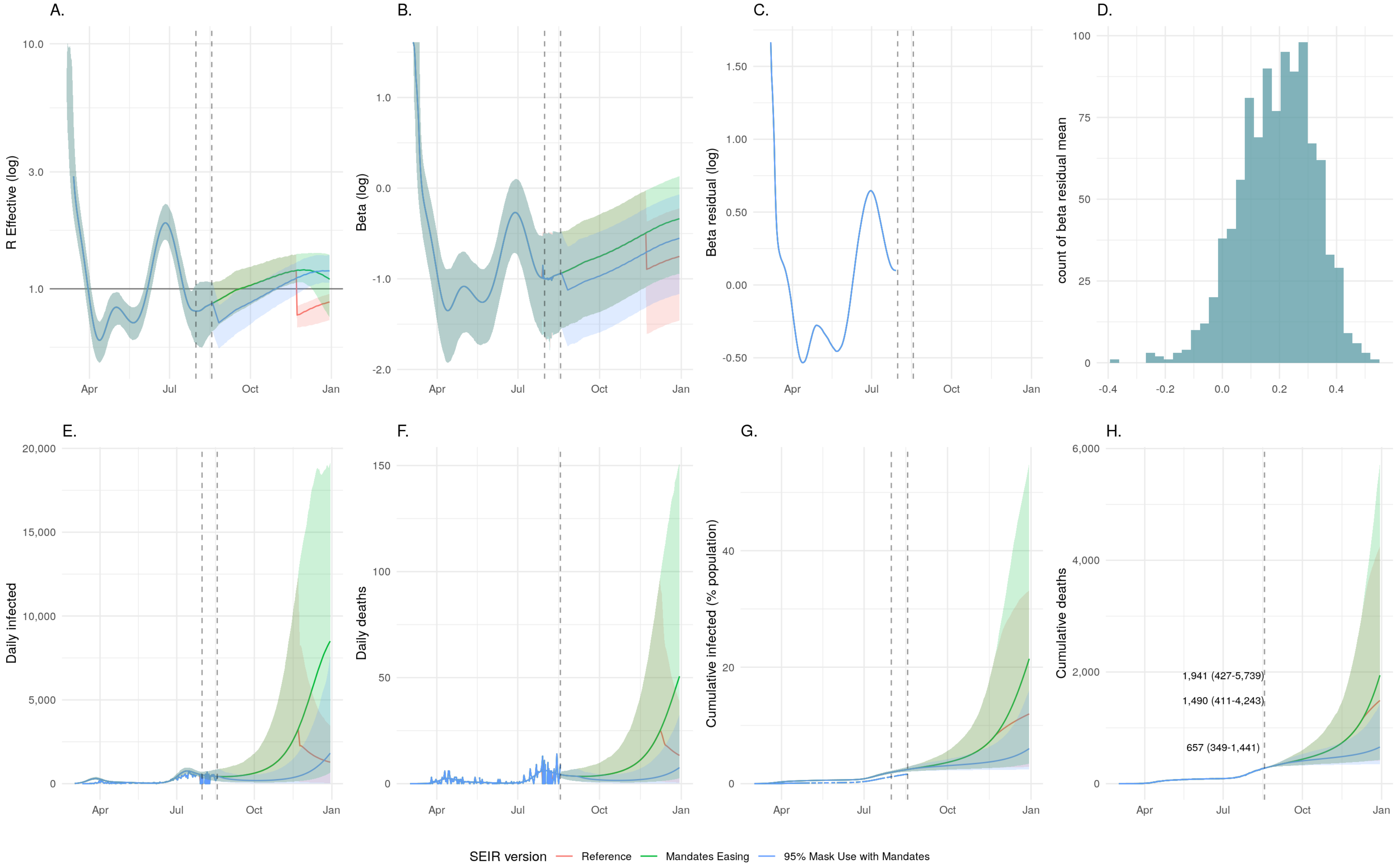

Idaho: SEIR fit comparison. **A**: predicted R effective for each model through December 31. **B**: predicted SEIR *beta* parameter. **C**: residual of predicted *beta* and the observed value calculated directly from infection data over time. **D**: histogram of residual values for *beta*. Panels A, B, C, and D are all displayed in log space, reflecting the space in which the SEIR model is fit. **E**: predicted daily infections from each model through December 31. **F**: predicted daily deaths from each model through December 31. **G**: predicted cumulative infections through December 31, as a proportion of the total population. **H**: predicted cumulative deaths through December 31. In panels E, F, G, and H, reported death and infections are plotted alongside model predictions in light blue.

28 Idaho: Covariate fits and regression coefficients

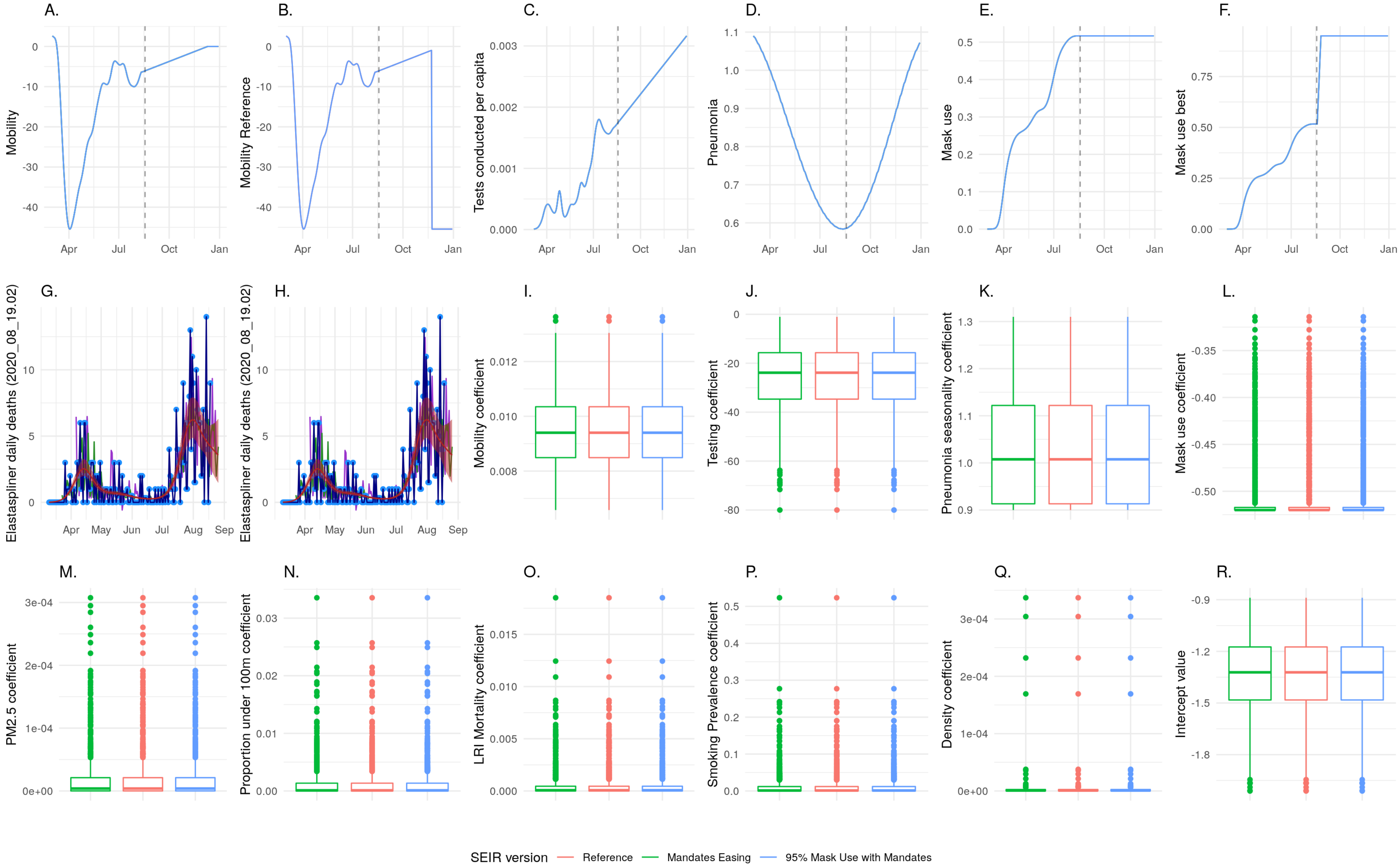

Idaho: Covariate fits and regression coefficients. **A-F**: Line plots showing predicted covariate time trends for **A)** mobility in the absence of additional mandates; **B)** mobility with additional mandates applied; **C)** diagnostic testing per capita; **D)** pneumonia seasonality; **E)** mask use per capita, and; **F)** mask use in a scenario where adherence increases to 95% of the population. **G-H**: COVID mortality data generated from reported daily deaths (blue); estimated based on reported hospitalizations (purple); estimated from reported cases (green); and via a spline fit through all available data types (red, 95% UI in pink). **I-R**: Box plots showing 1,000 draws of fixed effect coefficients in a multivariate regression fit to $log(beta)$.

29 Illinois: SEIR fit comparison

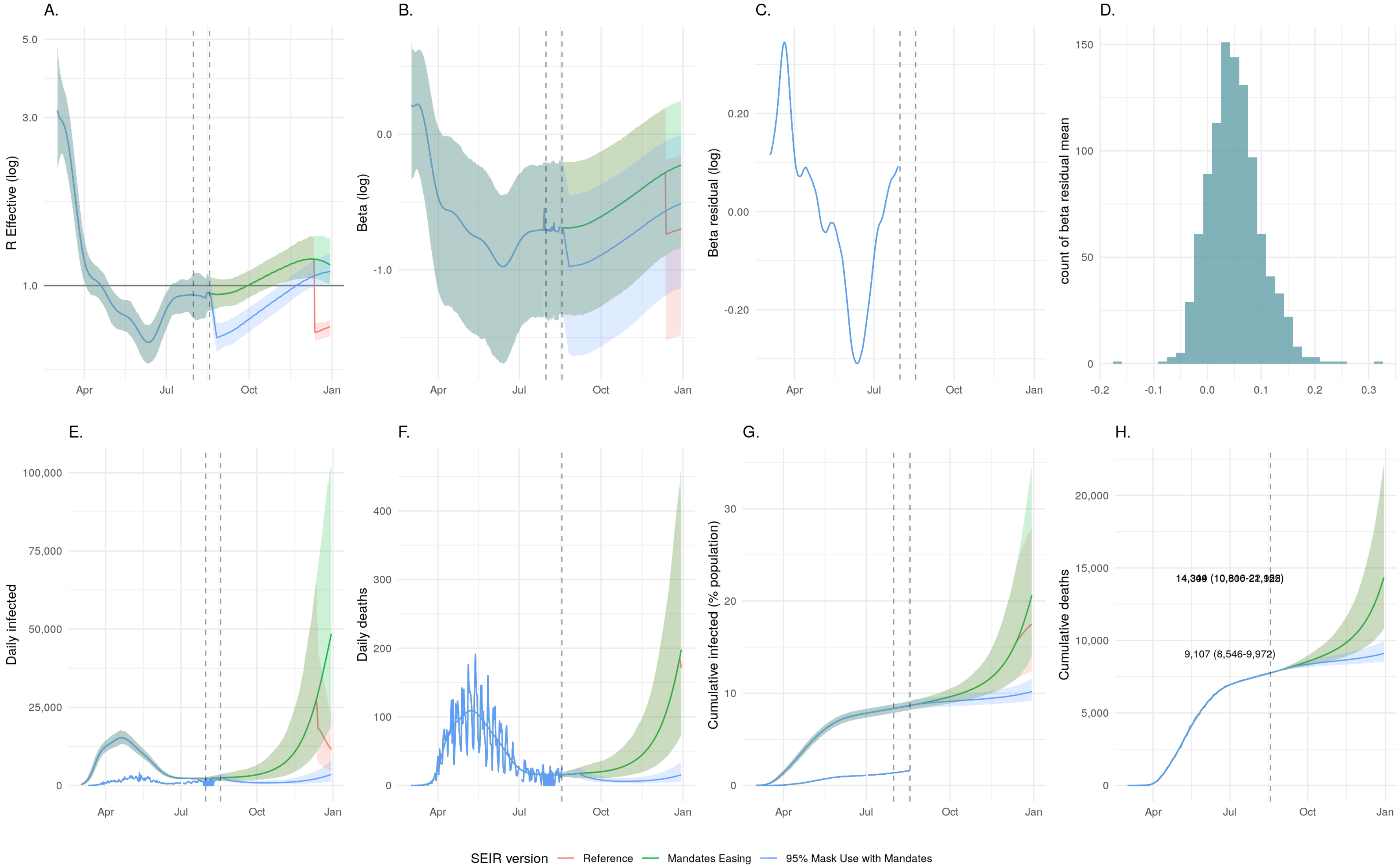

Illinois: SEIR fit comparison. A: predicted R effective for each model through December 31. **B**: predicted SEIR *beta* parameter. **C**: residual of predicted *beta* and the observed value calculated directly from infection data over time. **D**: histogram of residual values for *beta*. Panels A, B, C, and D are all displayed in log space, reflecting the space in which the SEIR model is fit. **E**: predicted daily infections from each model through December 31. **F**: predicted daily deaths from each model through December 31. **G**: predicted cumulative infections through December 31, as a proportion of the total population. **H**: predicted cumulative deaths through December 31. In panels E, F, G, and H, reported death and infections are plotted alongside model predictions in light blue.

30 Illinois: Covariate fits and regression coefficients

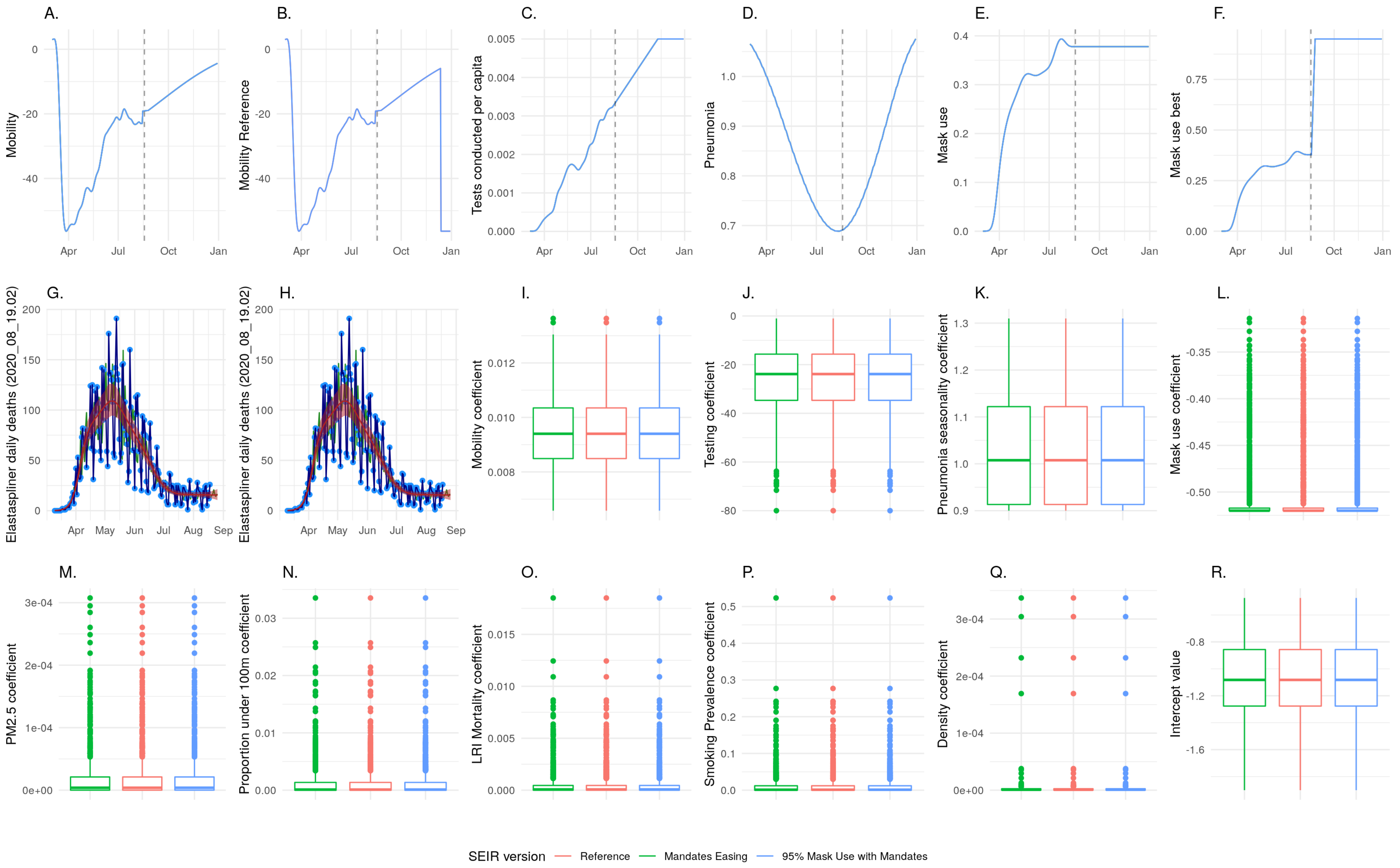

Illinois: Covariate fits and regression coefficients. **A-F**: Line plots showing predicted covariate time trends for **A)** mobility in the absence of additional mandates; **B)** mobility with additional mandates applied; **C)** diagnostic testing per capita; **D)** pneumonia seasonality; **E)** mask use per capita, and; **F)** mask use in a scenario where adherence increases to 95% of the population. **G-H**: COVID mortality data generated from reported daily deaths (blue); estimated based on reported hospitalizations (purple); estimated from reported cases (green); and via a spline fit through all available data types (red, 95% UI in pink). **I-R**: Box plots showing 1,000 draws of fixed effect coefficients in a multivariate regression fit to $log(beta)$.

31 Indiana: SEIR fit comparison

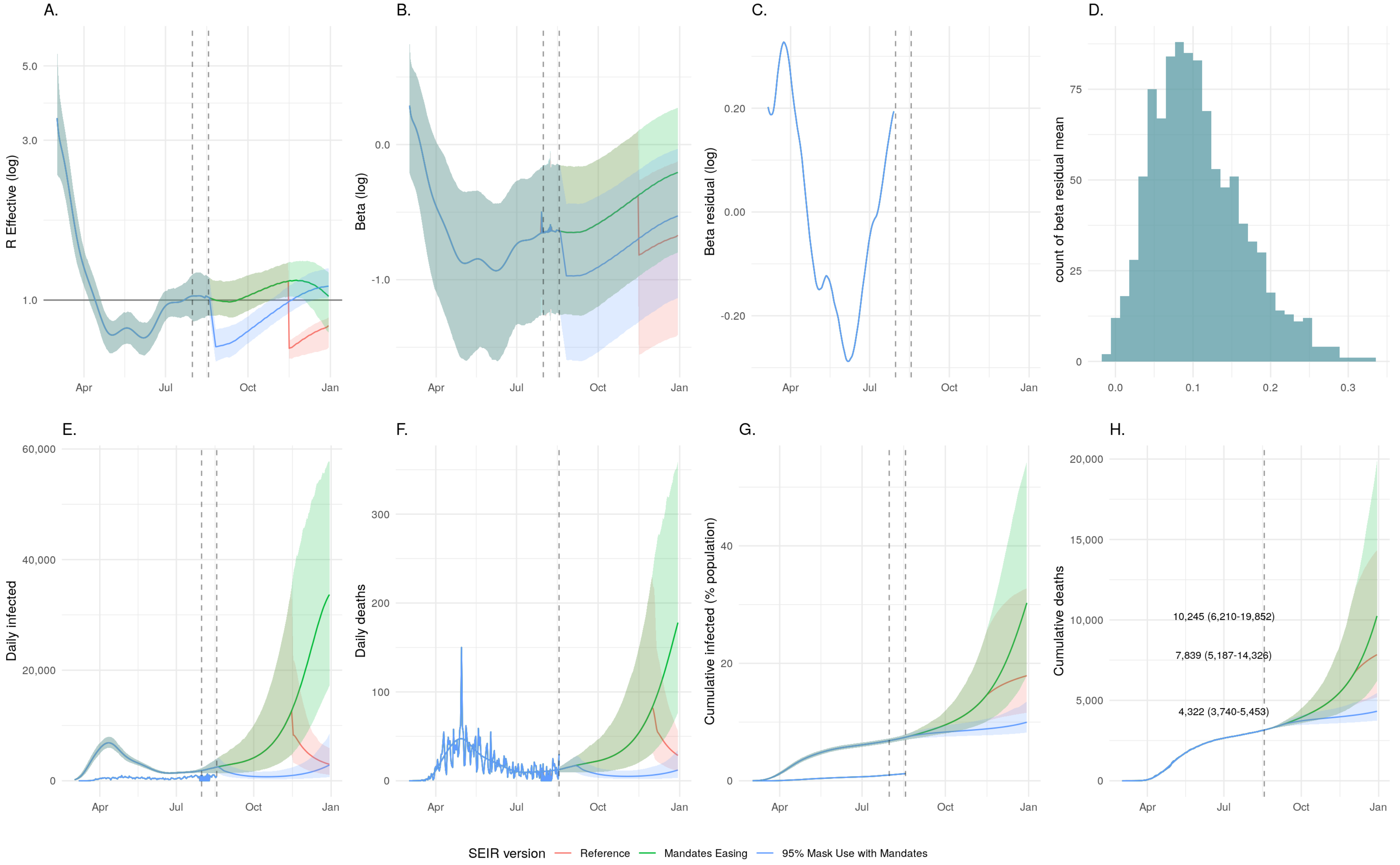

Indiana: SEIR fit comparison. A: predicted R effective for each model through December 31. **B**: predicted SEIR *beta* parameter. **C**: residual of predicted *beta* and the observed value calculated directly from infection data over time. **D**: histogram of residual values for *beta*. Panels A, B, C, and D are all displayed in log space, reflecting the space in which the SEIR model is fit. **E**: predicted daily infections from each model through December 31. **F**: predicted daily deaths from each model through December 31. **G**: predicted cumulative infections through December 31, as a proportion of the total population. **H**: predicted cumulative deaths through December 31. In panels E, F, G, and H, reported death and infections are plotted alongside model predictions in light blue.

32 Indiana: Covariate fits and regression coefficients

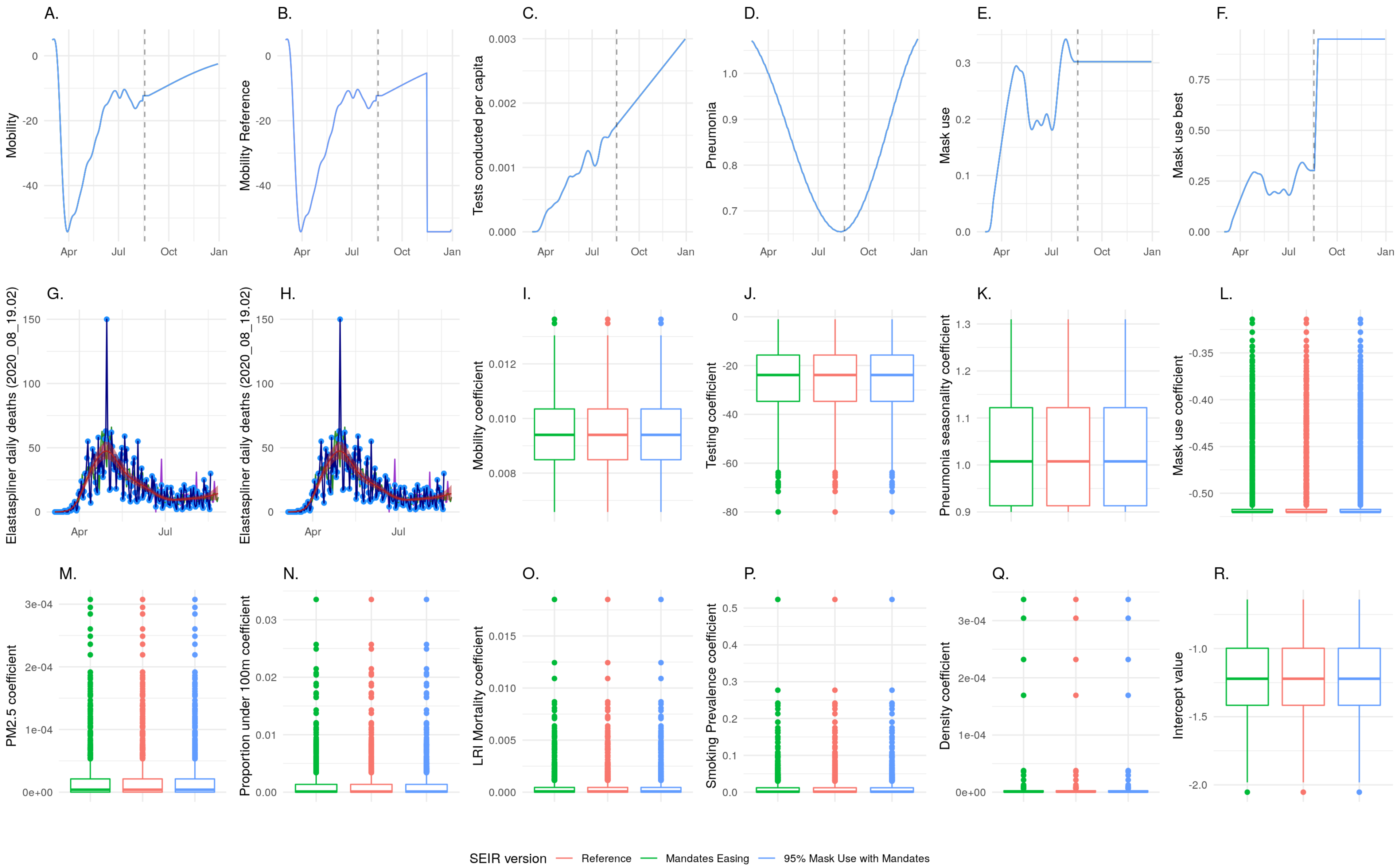

Indiana: Covariate fits and regression coefficients. A-F: Line plots showing predicted covariate time trends for **A)** mobility in the absence of additional mandates; **B)** mobility with additional mandates applied; **C)** diagnostic testing per capita; **D)** pneumonia seasonality; **E)** mask use per capita, and; **F)** mask use in a scenario where adherence increases to 95% of the population. **G-H**: COVID mortality data generated from reported daily deaths (blue); estimated based on reported hospitalizations (purple); estimated from reported cases (green); and via a spline fit through all available data types (red, 95% UI in pink). **I-R**: Box plots showing 1,000 draws of fixed effect coefficients in a multivariate regression fit to $log(beta)$.

33 Iowa: SEIR fit comparison

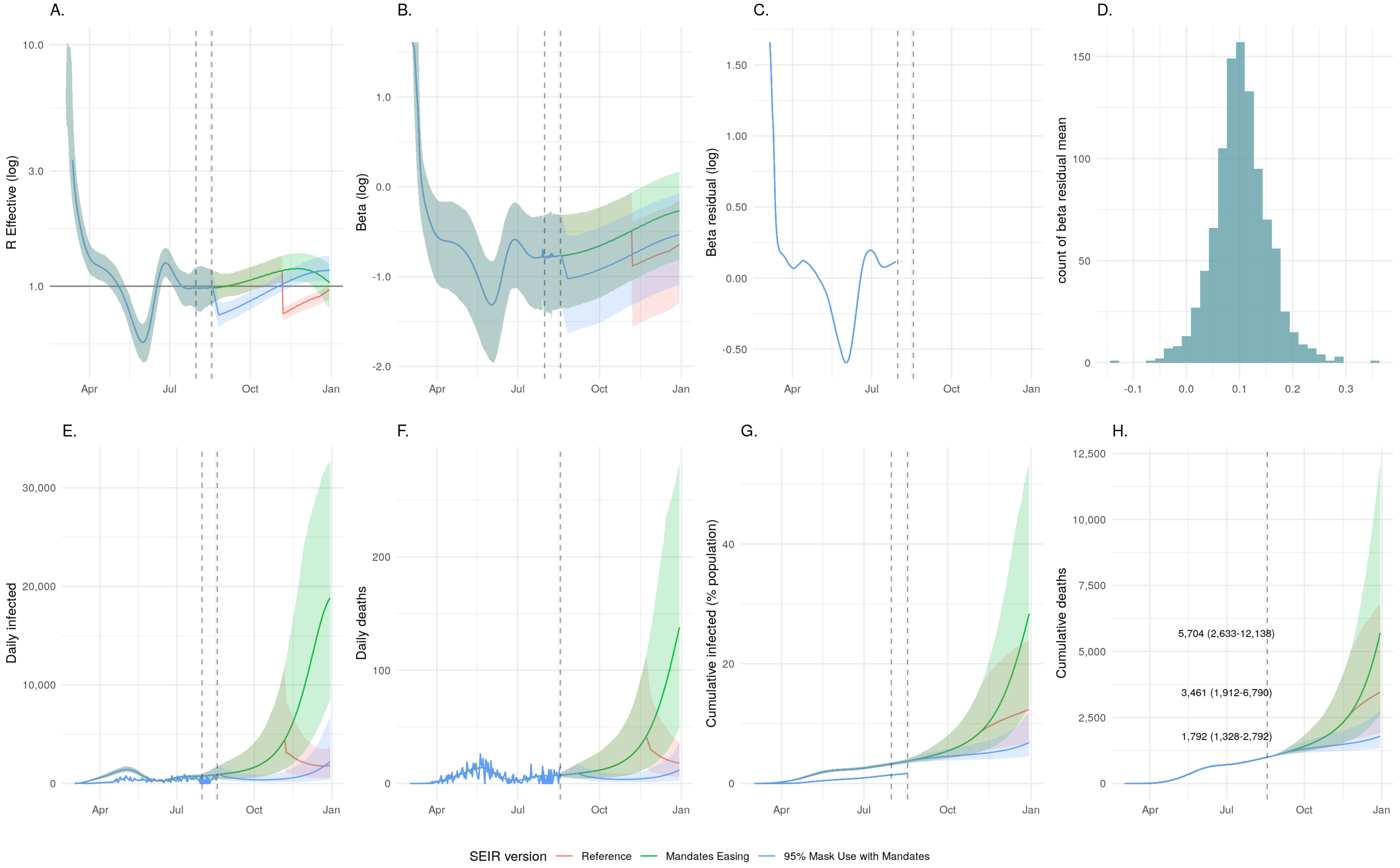

Iowa: SEIR fit comparison. A: predicted R effective for each model through December 31. **B**: predicted SEIR *beta* parameter. **C**: residual of predicted *beta* and the observed value calculated directly from infection data over time. **D**: histogram of residual values for *beta*. Panels A, B, C, and D are all displayed in log space, reflecting the space in which the SEIR model is fit. **E**: predicted daily infections from each model through December 31. **F**: predicted daily deaths from each model through December 31. **G**: predicted cumulative infections through December 31, as a proportion of the total population. **H**: predicted cumulative deaths through December 31. In panels E, F, G, and H, reported death and infections are plotted alongside model predictions in light blue.

34 Iowa: Covariate fits and regression coefficients

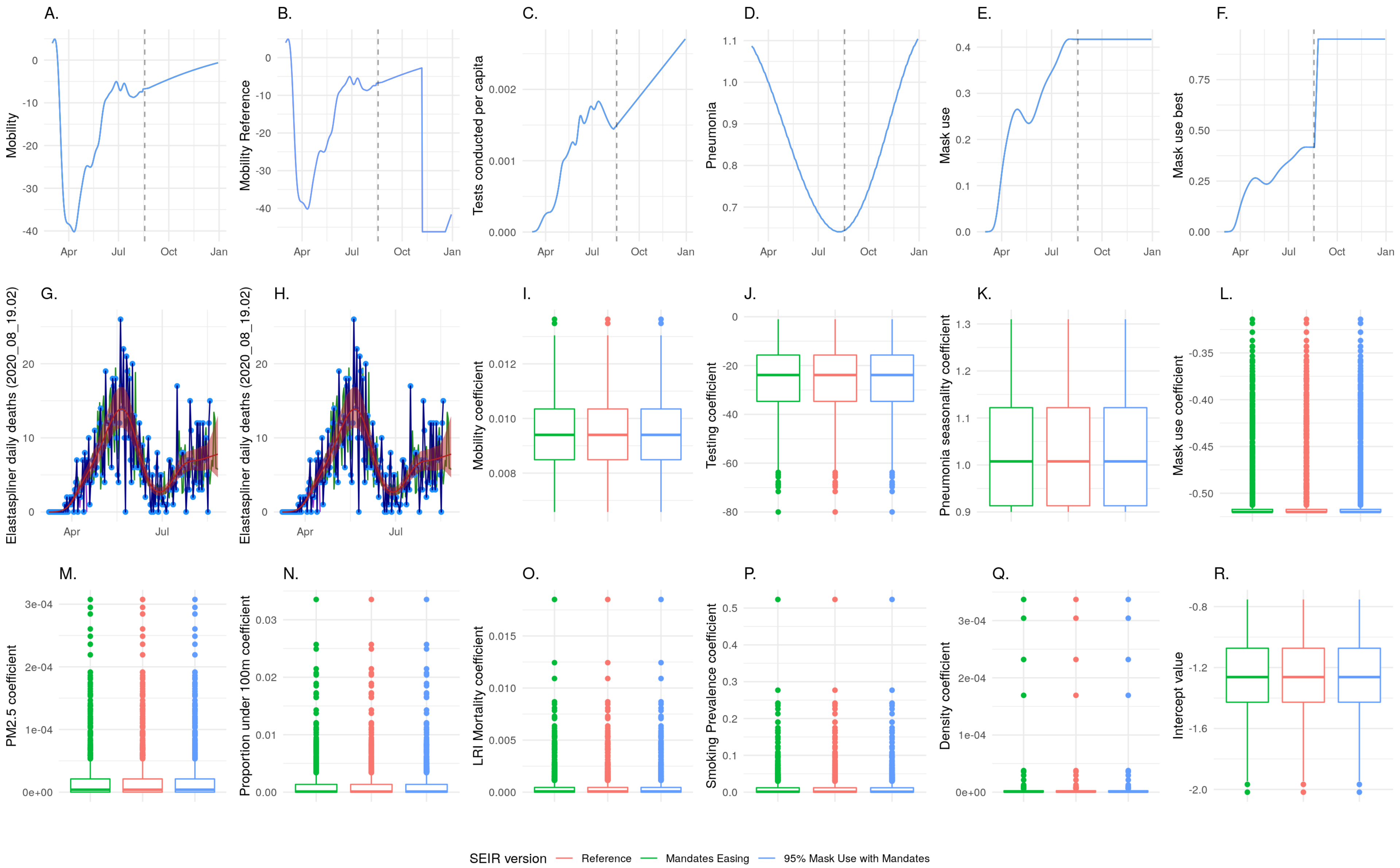

Iowa: Covariate fits and regression coefficients. A-F: Line plots showing predicted covariate time trends for **A)** mobility in the absence of additional mandates; **B)** mobility with additional mandates applied; **C)** diagnostic testing per capita; **D)** pneumonia seasonality; **E)** mask use per capita, and; **F)** mask use in a scenario where adherence increases to 95% of the population. **G-H**: COVID mortality data generated from reported daily deaths (blue); estimated based on reported hospitalizations (purple); estimated from reported cases (green); and via a spline fit through all available data types (red, 95% UI in pink). **I-R**: Box plots showing 1,000 draws of fixed effect coefficients in a multivariate regression fit to $log(beta)$.

35 Kansas: SEIR fit comparison

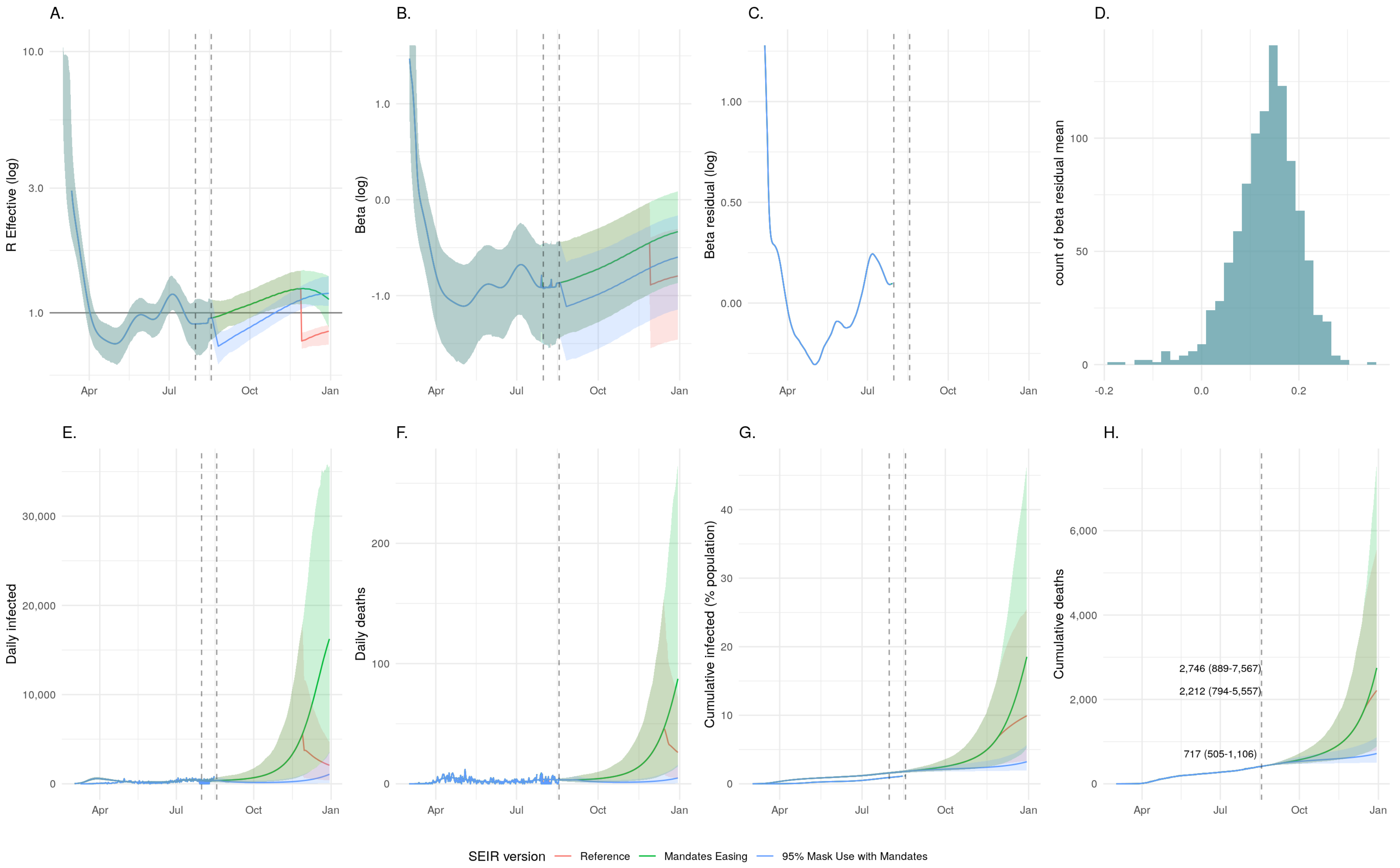

Kansas: SEIR fit comparison. A: predicted R effective for each model through December 31. **B**: predicted SEIR *beta* parameter. **C**: residual of predicted *beta* and the observed value calculated directly from infection data over time. **D**: histogram of residual values for *beta*. Panels A, B, C, and D are all displayed in log space, reflecting the space in which the SEIR model is fit. **E**: predicted daily infections from each model through December 31. **F**: predicted daily deaths from each model through December 31. **G**: predicted cumulative infections through December 31, as a proportion of the total population. **H**: predicted cumulative deaths through December 31. In panels E, F, G, and H, reported death and infections are plotted alongside model predictions in light blue.

36 Kansas: Covariate fits and regression coefficients

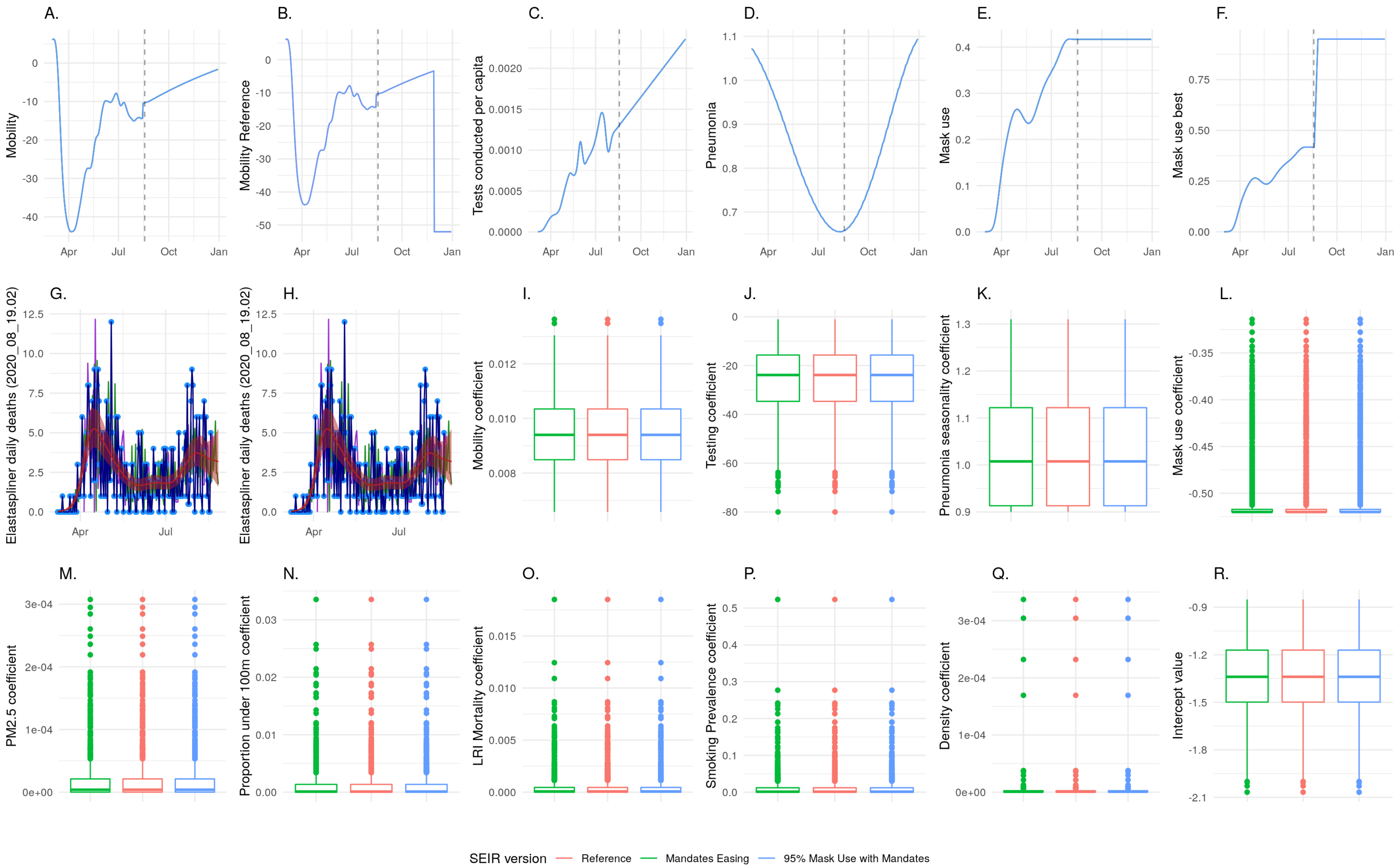

Kansas: Covariate fits and regression coefficients. **A-F**: Line plots showing predicted covariate time trends for **A)** mobility in the absence of additional mandates; **B)** mobility with additional mandates applied; **C)** diagnostic testing per capita; **D)** pneumonia seasonality; **E)** mask use per capita, and; **F)** mask use in a scenario where adherence increases to 95% of the population. **G-H**: COVID mortality data generated from reported daily deaths (blue); estimated based on reported hospitalizations (purple); estimated from reported cases (green); and via a spline fit through all available data types (red, 95% UI in pink). **I-R**: Box plots showing 1,000 draws of fixed effect coefficients in a multivariate regression fit to $log(beta)$.

37 Kentucky: SEIR fit comparison

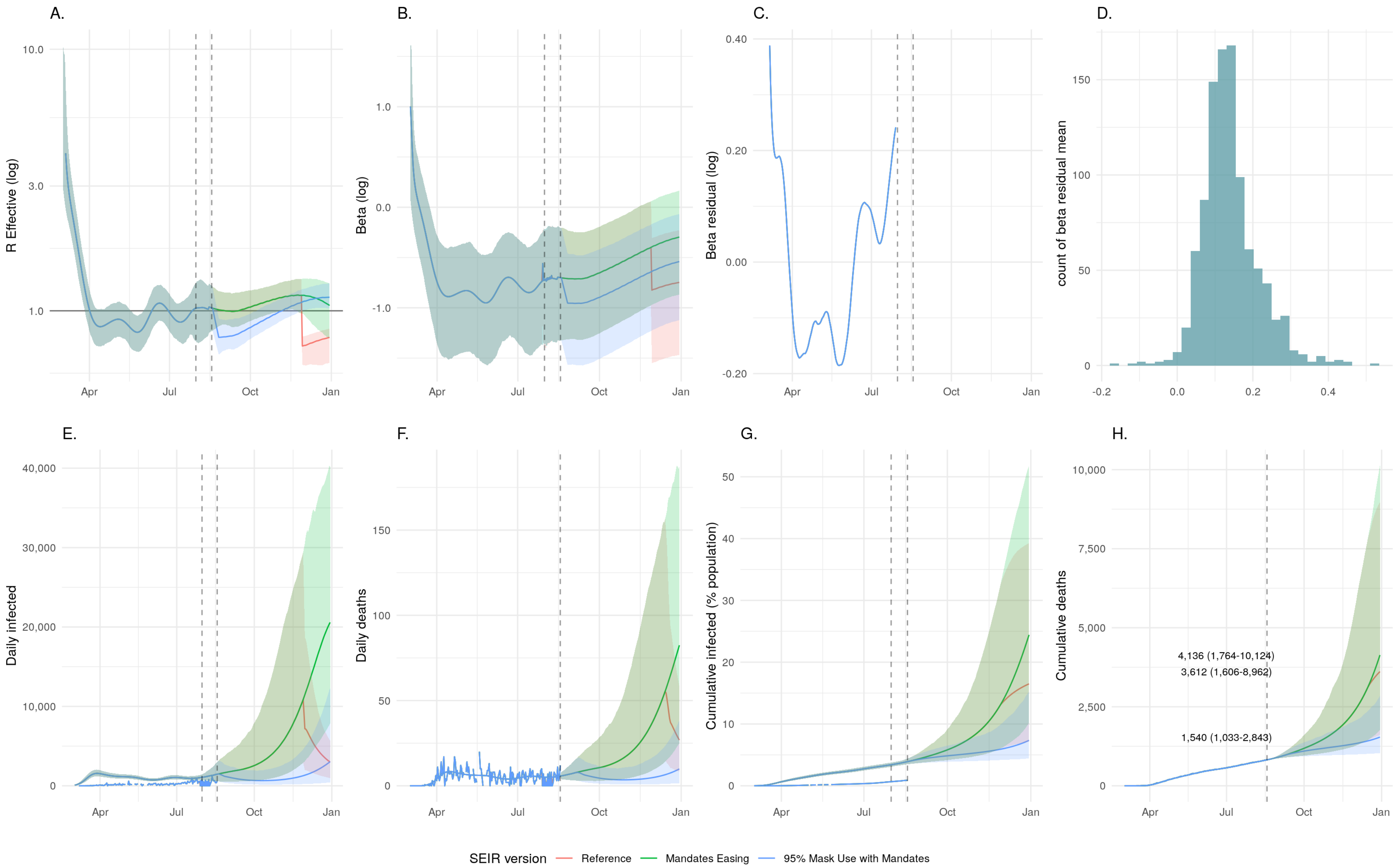

Kentucky: SEIR fit comparison. A: predicted R effective for each model through December 31. **B**: predicted SEIR *beta* parameter. **C**: residual of predicted *beta* and the observed value calculated directly from infection data over time. **D**: histogram of residual values for *beta*. Panels A, B, C, and D are all displayed in log space, reflecting the space in which the SEIR model is fit. **E**: predicted daily infections from each model through December 31. **F**: predicted daily deaths from each model through December 31. **G**: predicted cumulative infections through December 31, as a proportion of the total population. **H**: predicted cumulative deaths through December 31. In panels E, F, G, and H, reported death and infections are plotted alongside model predictions in light blue.

38 Kentucky: Covariate fits and regression coefficients

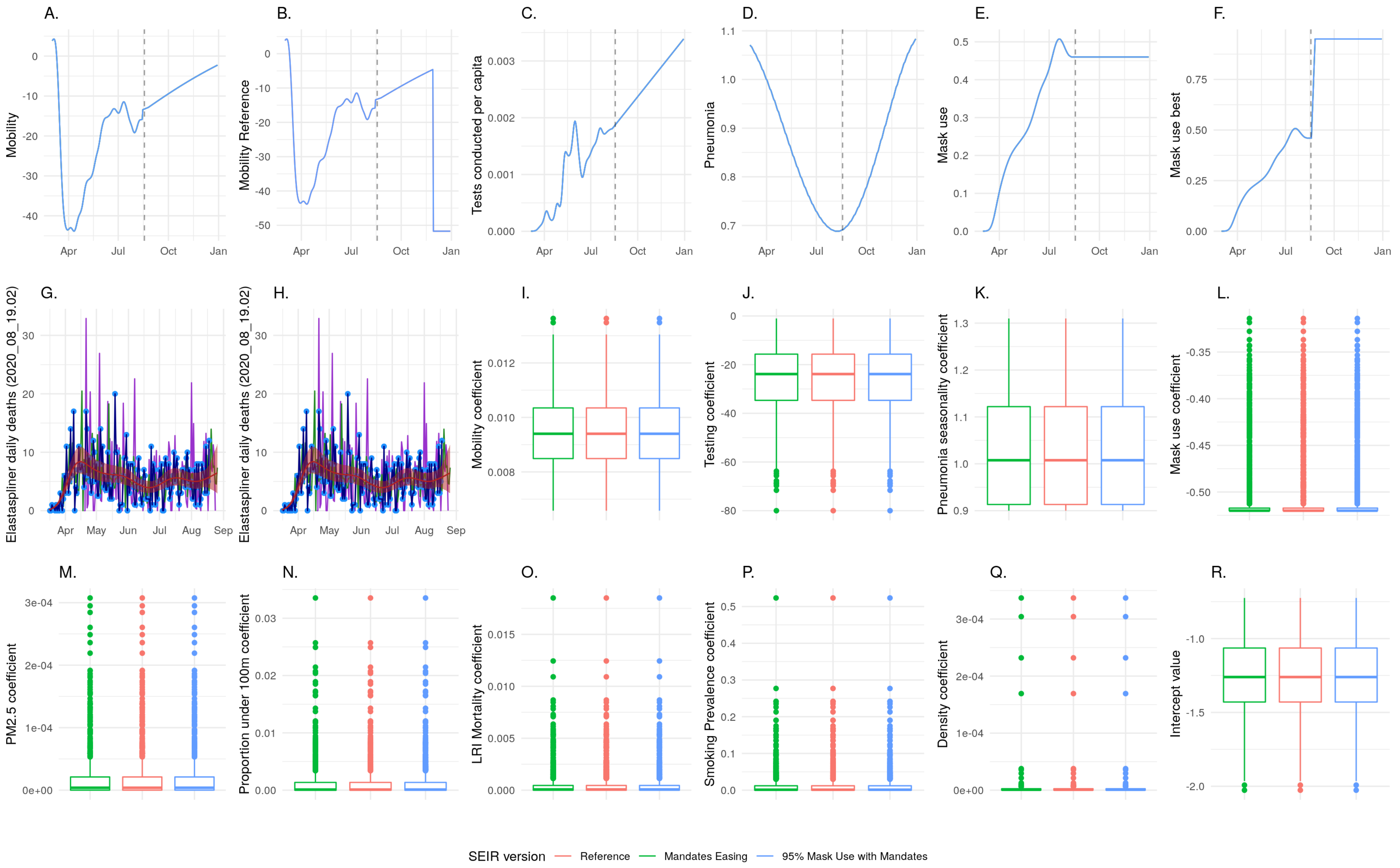

Kentucky: Covariate fits and regression coefficients. A-F: Line plots showing predicted covariate time trends for **A)** mobility in the absence of additional mandates; **B)** mobility with additional mandates applied; **C)** diagnostic testing per capita; **D)** pneumonia seasonality; **E)** mask use per capita, and; **F)** mask use in a scenario where adherence increases to 95% of the population. **G-H**: COVID mortality data generated from reported daily deaths (blue); estimated based on reported hospitalizations (purple); estimated from reported cases (green); and via a spline fit through all available data types (red, 95% UI in pink). **I-R**: Box plots showing 1,000 draws of fixed effect coefficients in a multivariate regression fit to $log(beta)$.

39 Louisiana: SEIR fit comparison

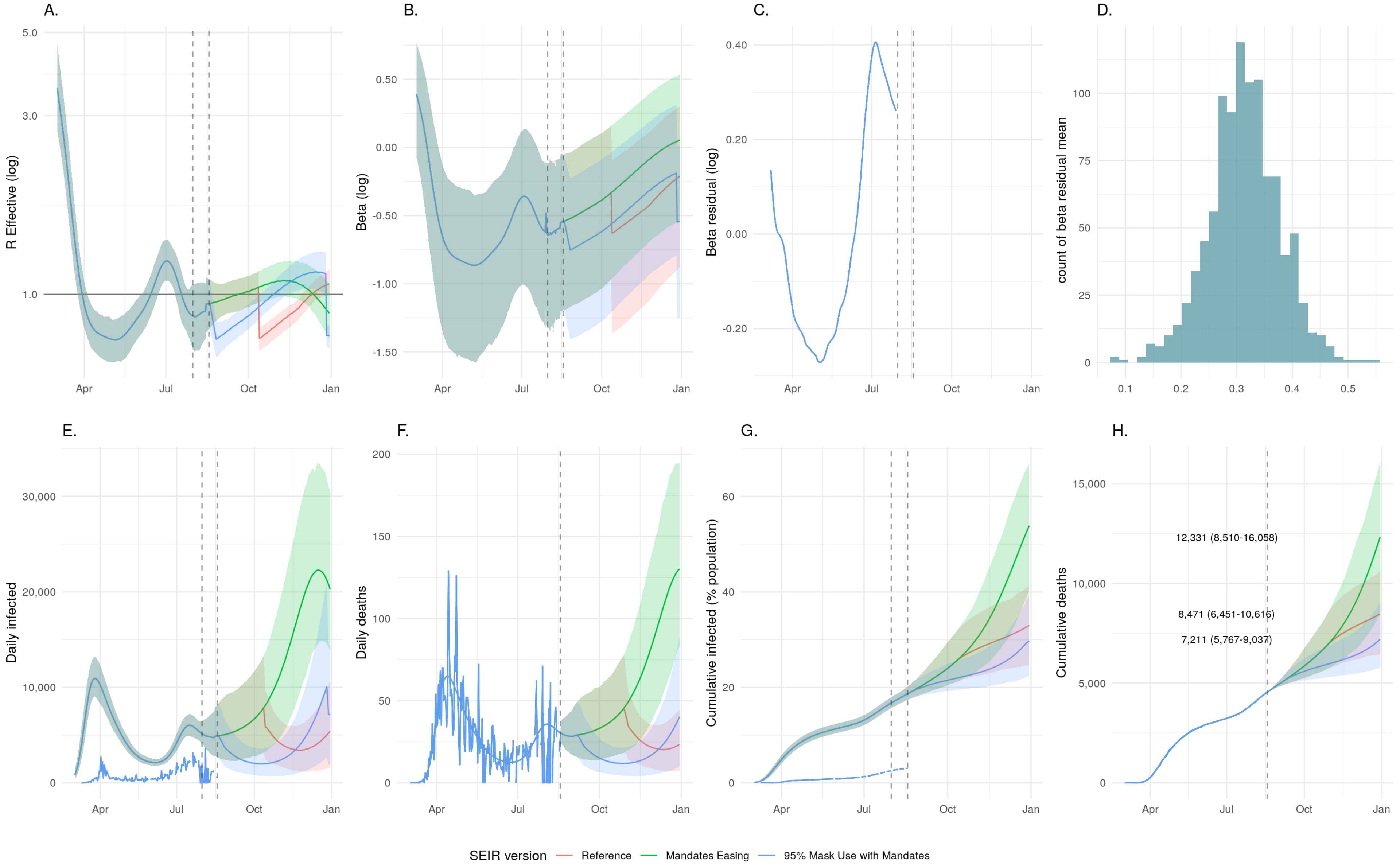

Louisiana: SEIR fit comparison. A: predicted R effective for each model through December 31. **B**: predicted SEIR *beta* parameter. **C**: residual of predicted *beta* and the observed value calculated directly from infection data over time. **D**: histogram of residual values for *beta*. Panels A, B, C, and D are all displayed in log space, reflecting the space in which the SEIR model is fit. **E**: predicted daily infections from each model through December 31. **F**: predicted daily deaths from each model through December 31. **G**: predicted cumulative infections through December 31, as a proportion of the total population. **H**: predicted cumulative deaths through December 31. In panels E, F, G, and H, reported death and infections are plotted alongside model predictions in light blue.

40 Louisiana: Covariate fits and regression coefficients

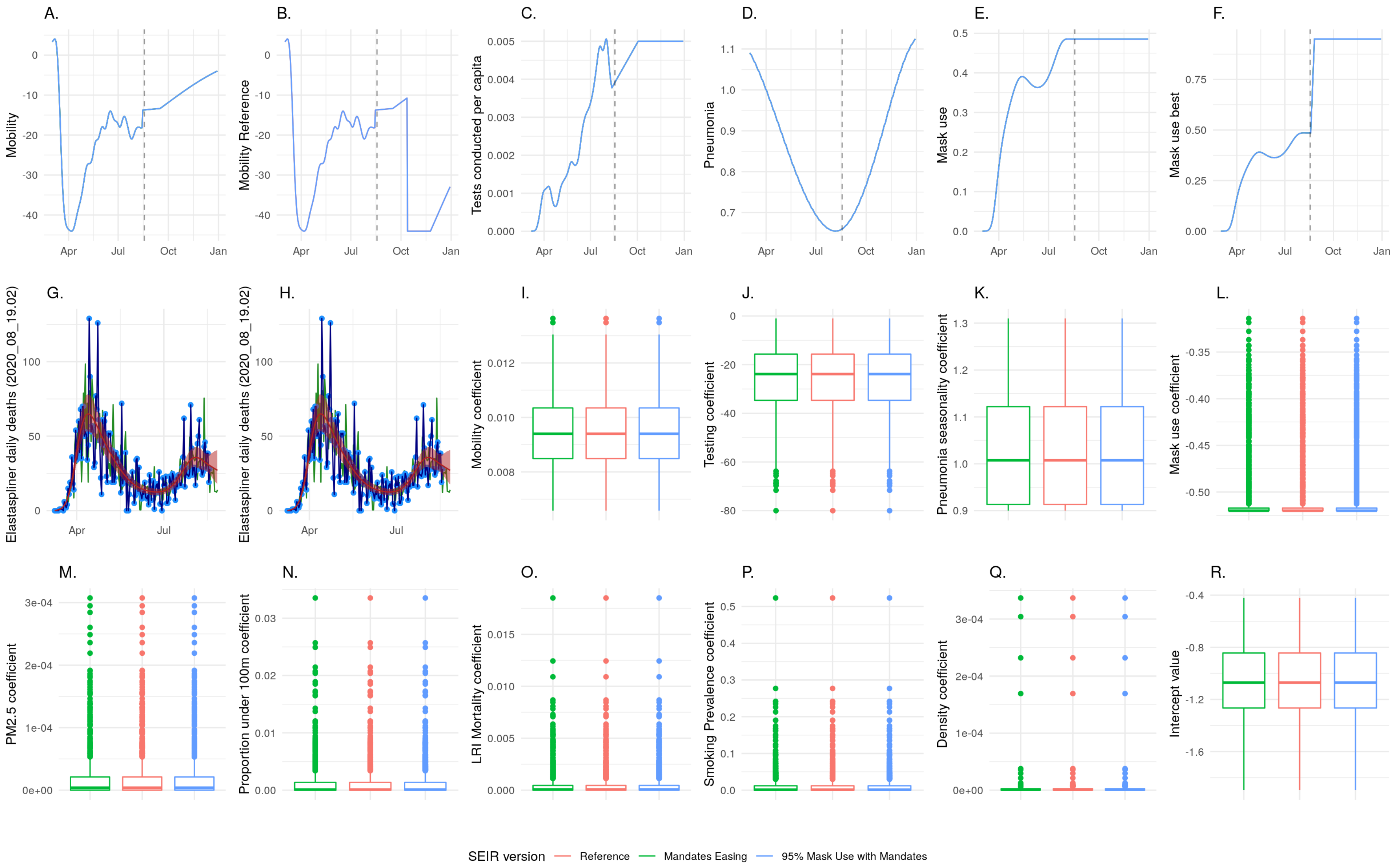

Louisiana: Covariate fits and regression coefficients. A-F: Line plots showing predicted covariate time trends for **A)** mobility in the absence of additional mandates; **B)** mobility with additional mandates applied; **C)** diagnostic testing per capita; **D)** pneumonia seasonality; **E)** mask use per capita, and; **F)** mask use in a scenario where adherence increases to 95% of the population. **G-H**: COVID mortality data generated from reported daily deaths (blue); estimated based on reported hospitalizations (purple); estimated from reported cases (green); and via a spline fit through all available data types (red, 95% UI in pink). **I-R**: Box plots showing 1,000 draws of fixed effect coefficients in a multivariate regression fit to $log(beta)$.

41 Maine: SEIR fit comparison

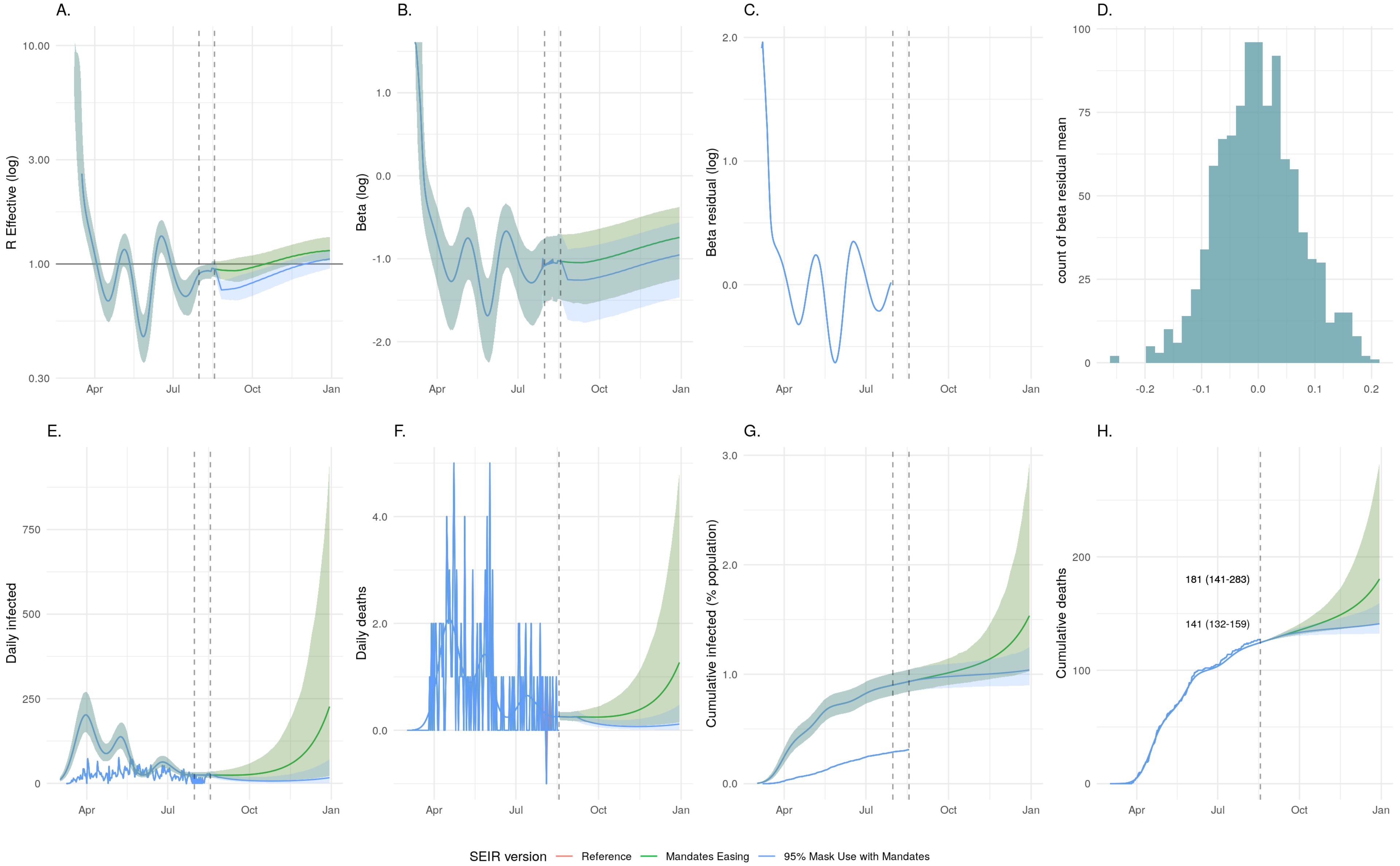

Maine: SEIR fit comparison. **A**: predicted R effective for each model through December 31. **B**: predicted SEIR *beta* parameter. **C**: residual of predicted *beta* and the observed value calculated directly from infection data over time. **D**: histogram of residual values for *beta*. Panels A, B, C, and D are all displayed in log space, reflecting the space in which the SEIR model is fit. **E**: predicted daily infections from each model through December 31. **F**: predicted daily deaths from each model through December 31. **G**: predicted cumulative infections through December 31, as a proportion of the total population. **H**: predicted cumulative deaths through December 31. In panels E, F, G, and H, reported death and infections are plotted alongside model predictions in light blue.

42 Maine: Covariate fits and regression coefficients

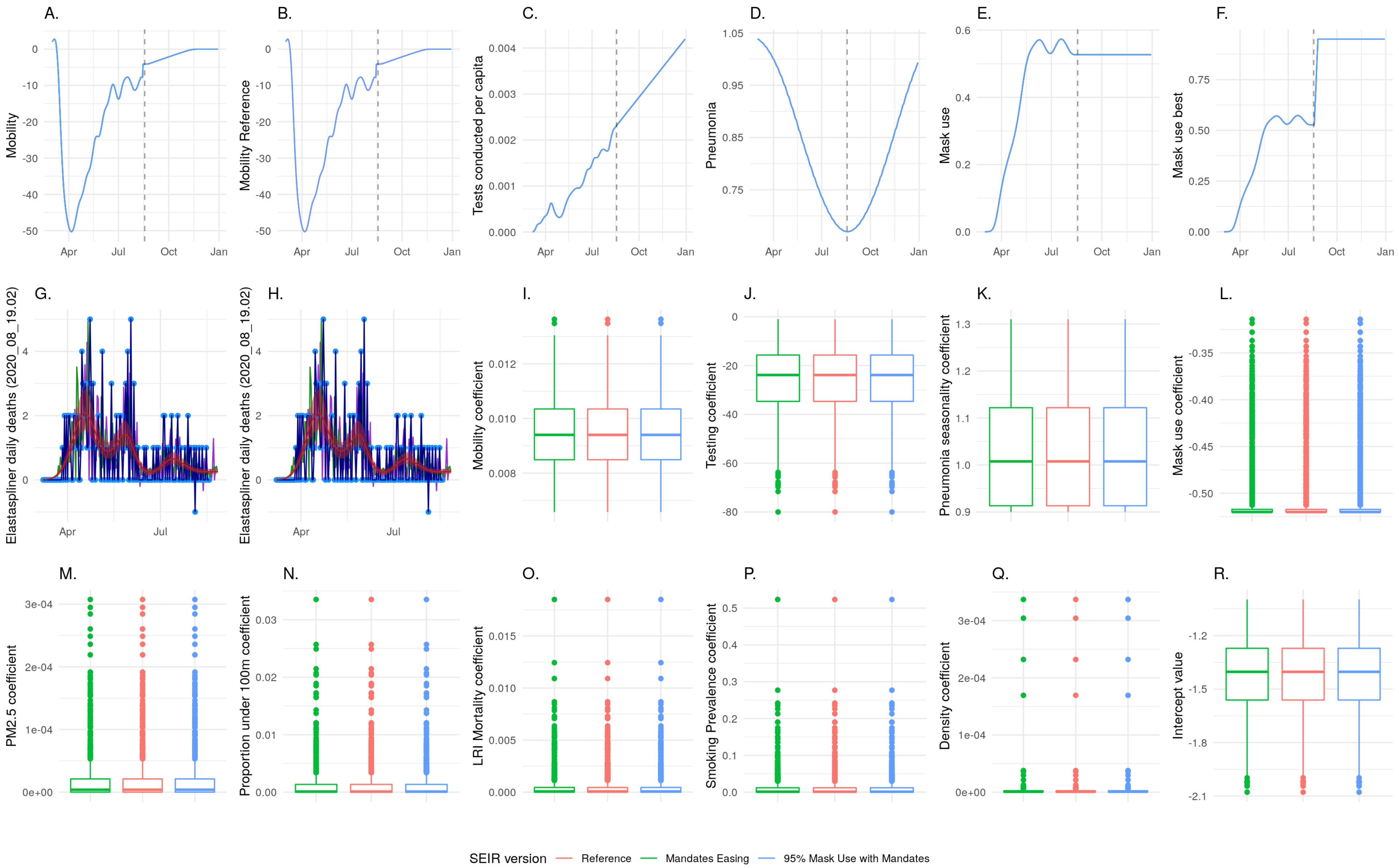

Maine: Covariate fits and regression coefficients. **A-F**: Line plots showing predicted covariate time trends for **A)** mobility in the absence of additional mandates; **B)** mobility with additional mandates applied; **C)** diagnostic testing per capita; **D)** pneumonia seasonality; **E)** mask use per capita, and; **F)** mask use in a scenario where adherence increases to 95% of the population. **G-H**: COVID mortality data generated from reported daily deaths (blue); estimated based on reported hospitalizations (purple); estimated from reported cases (green); and via a spline fit through all available data types (red, 95% UI in pink). **I-R**: Box plots showing 1,000 draws of fixed effect coefficients in a multivariate regression fit to $log(beta)$.

43 Maryland: SEIR fit comparison

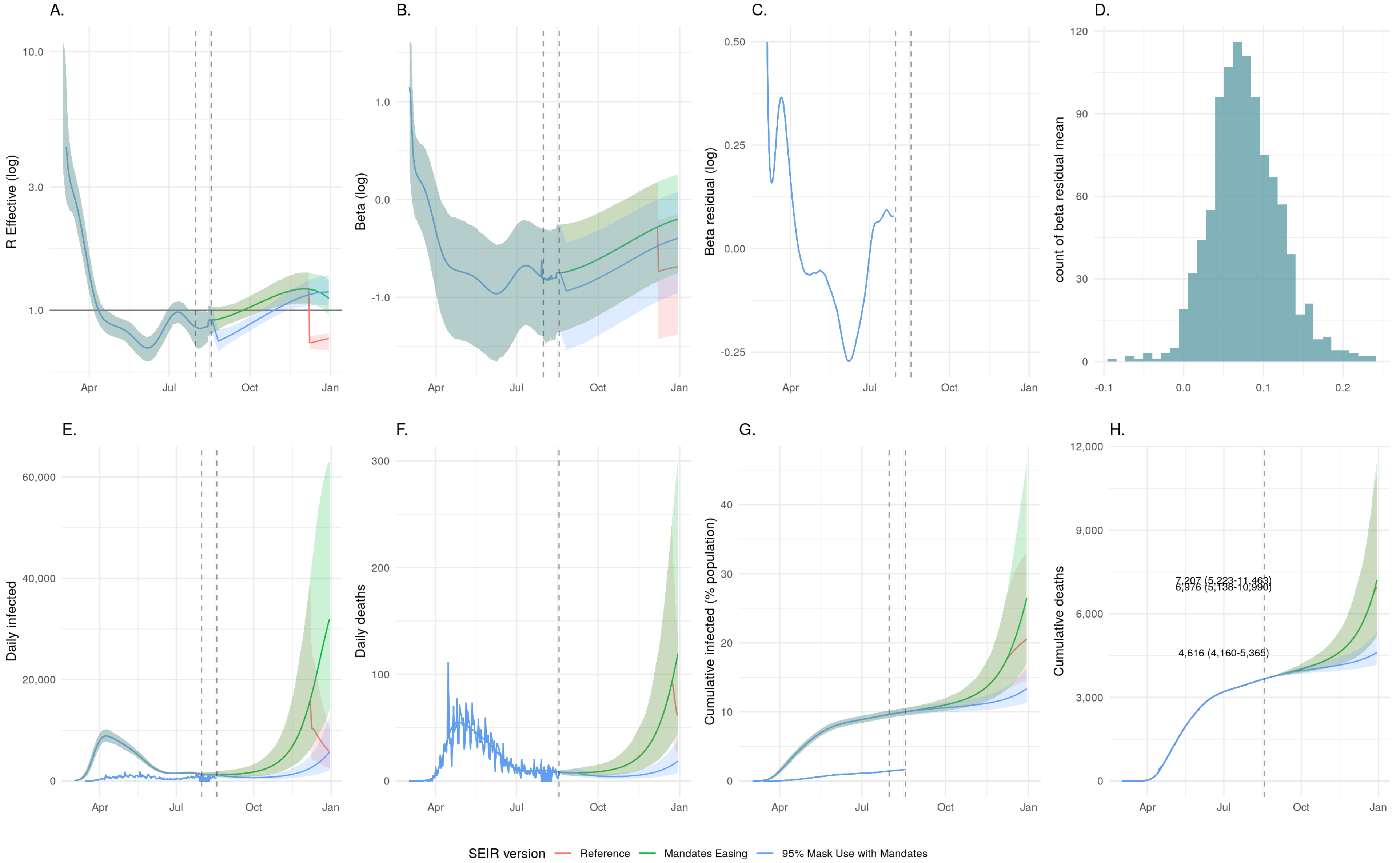

Maryland: SEIR fit comparison. A: predicted R effective for each model through December 31. **B**: predicted SEIR *beta* parameter. **C**: residual of predicted *beta* and the observed value calculated directly from infection data over time. **D**: histogram of residual values for *beta*. Panels A, B, C, and D are all displayed in log space, reflecting the space in which the SEIR model is fit. **E**: predicted daily infections from each model through December 31. **F**: predicted daily deaths from each model through December 31. **G**: predicted cumulative infections through December 31, as a proportion of the total population. **H**: predicted cumulative deaths through December 31. In panels E, F, G, and H, reported death and infections are plotted alongside model predictions in light blue.

44 Maryland: Covariate fits and regression coefficients

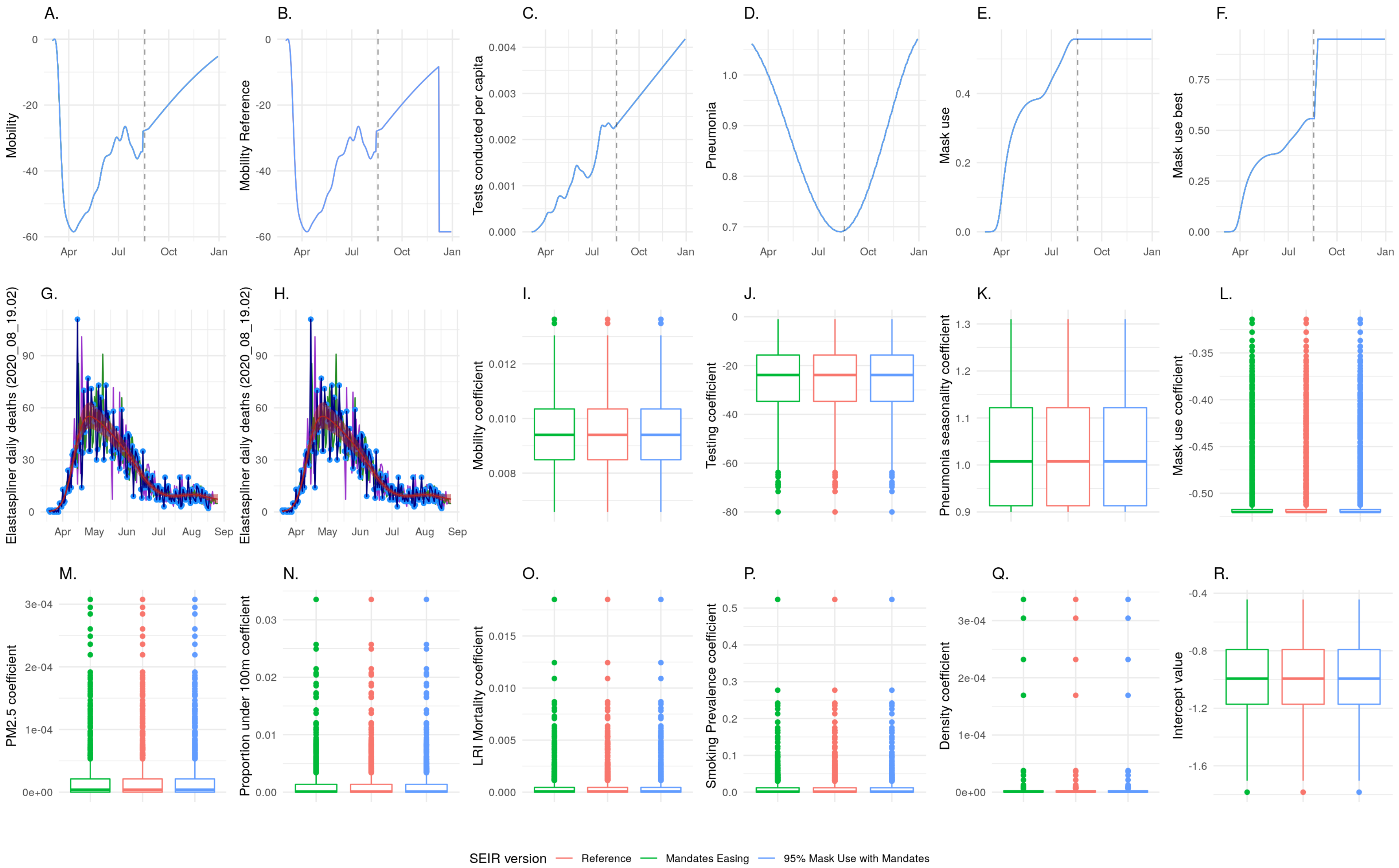

Maryland: Covariate fits and regression coefficients. **A-F**: Line plots showing predicted covariate time trends for **A)** mobility in the absence of additional mandates; **B)** mobility with additional mandates applied; **C)** diagnostic testing per capita; **D)** pneumonia seasonality; **E)** mask use per capita, and; **F)** mask use in a scenario where adherence increases to 95% of the population. **G-H**: COVID mortality data generated from reported daily deaths (blue); estimated based on reported hospitalizations (purple); estimated from reported cases (green); and via a spline fit through all available data types (red, 95% UI in pink). **I-R**: Box plots showing 1,000 draws of fixed effect coefficients in a multivariate regression fit to $log(beta)$.

45 Massachusetts: SEIR fit comparison

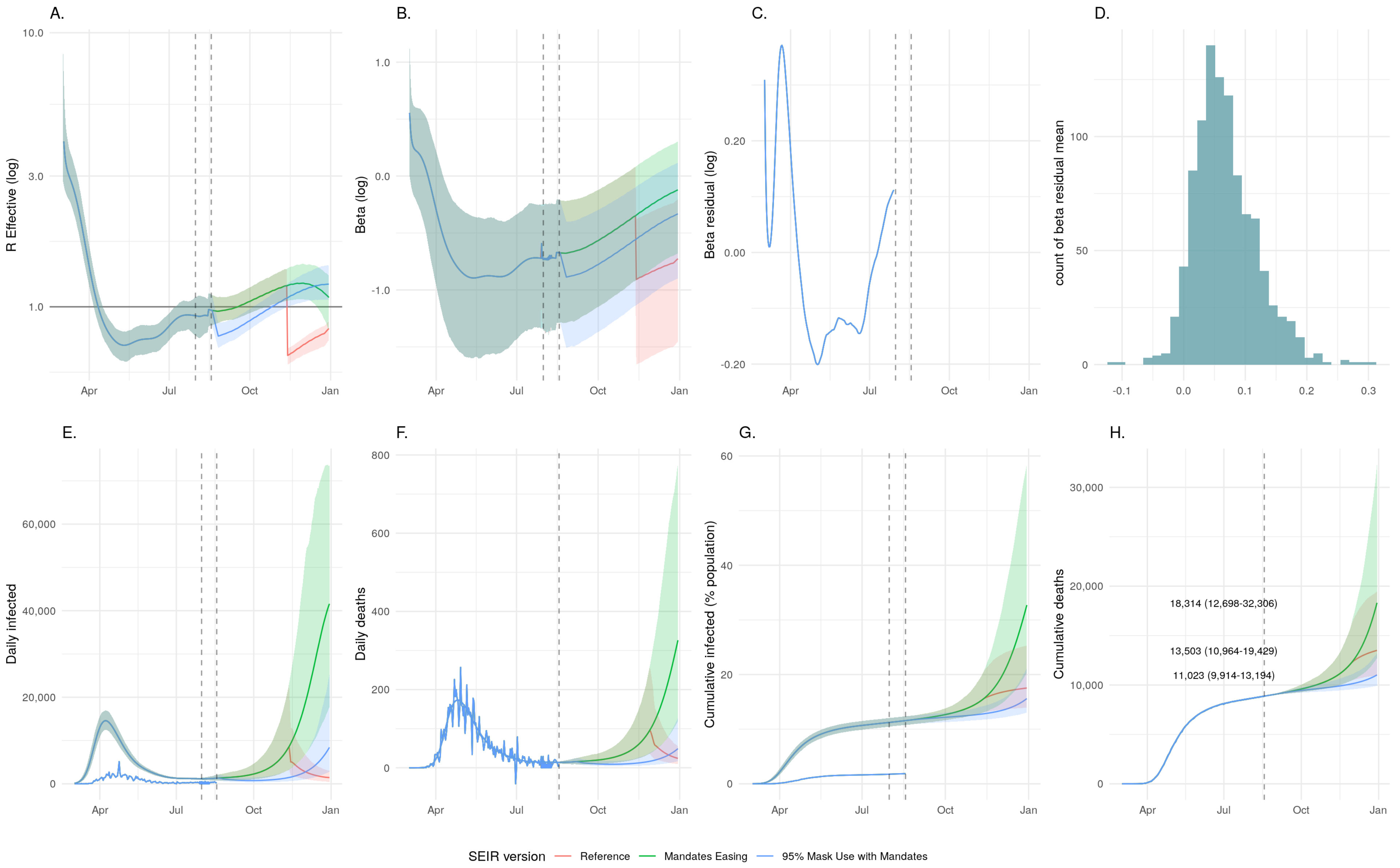

Massachusetts: SEIR fit comparison. **A**: predicted R effective for each model through December 31. **B**: predicted SEIR *beta* parameter. **C**: residual of predicted *beta* and the observed value calculated directly from infection data over time. **D**: histogram of residual values for *beta*. Panels A, B, C, and D are all displayed in log space, reflecting the space in which the SEIR model is fit. **E**: predicted daily infections from each model through December 31. **F**: predicted daily deaths from each model through December 31. **G**: predicted cumulative infections through December 31, as a proportion of the total population. **H**: predicted cumulative deaths through December 31. In panels E, F, G, and H, reported death and infections are plotted alongside model predictions in light blue.

46 Massachusetts: Covariate fits and regression coefficients

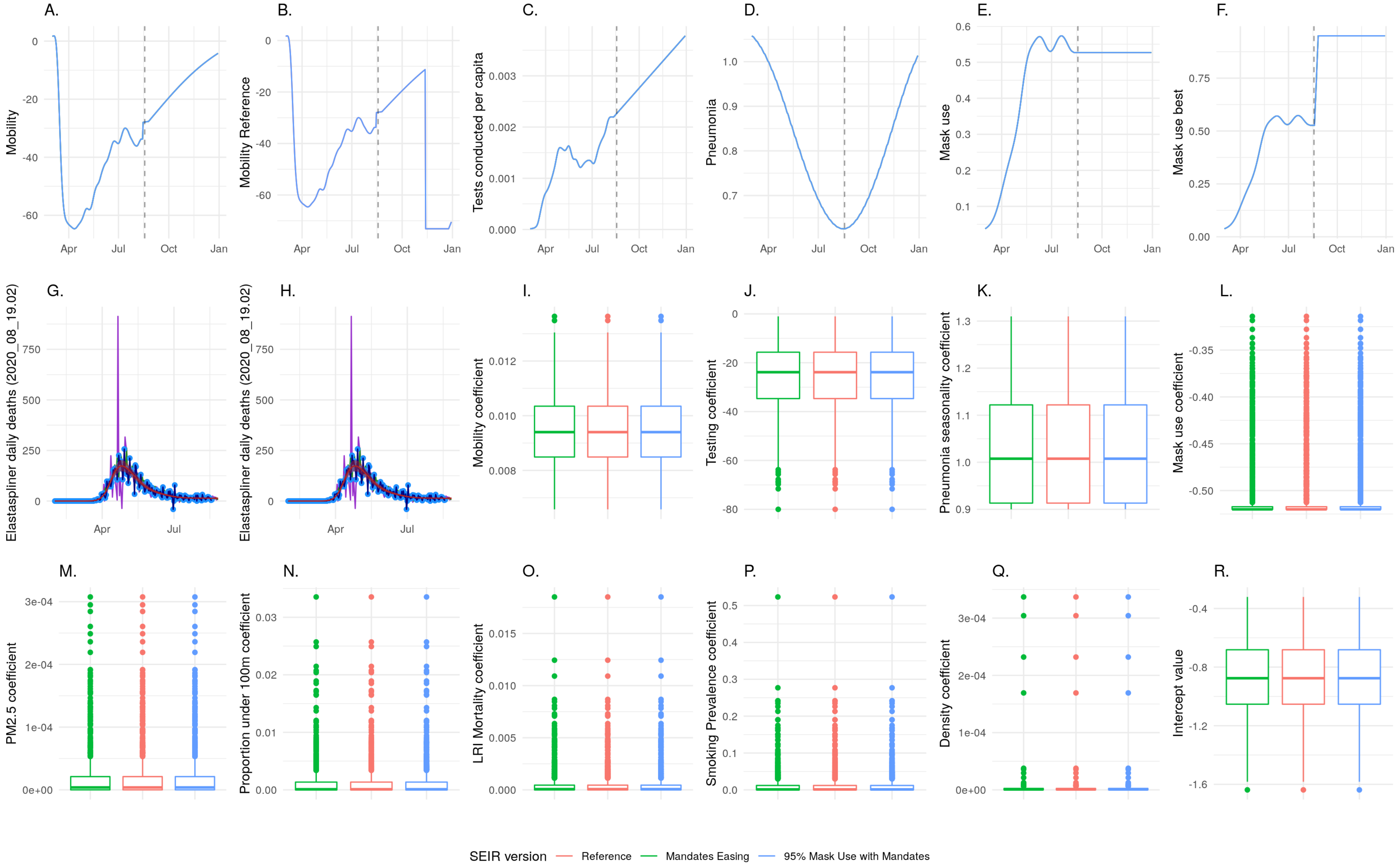

Massachusetts: Covariate fits and regression coefficients. A-F: Line plots showing predicted covariate time trends for **A)** mobility in the absence of additional mandates; **B)** mobility with additional mandates applied; **C)** diagnostic testing per capita; **D)** pneumonia seasonality; **E)** mask use per capita, and; **F)** mask use in a scenario where adherence increases to 95% of the population. **G-H**: COVID mortality data generated from reported daily deaths (blue); estimated based on reported hospitalizations (purple); estimated from reported cases (green); and via a spline fit through all available data types (red, 95% UI in pink). **I-R**: Box plots showing 1,000 draws of fixed effect coefficients in a multivariate regression fit to $log(beta)$.

47 Michigan: SEIR fit comparison

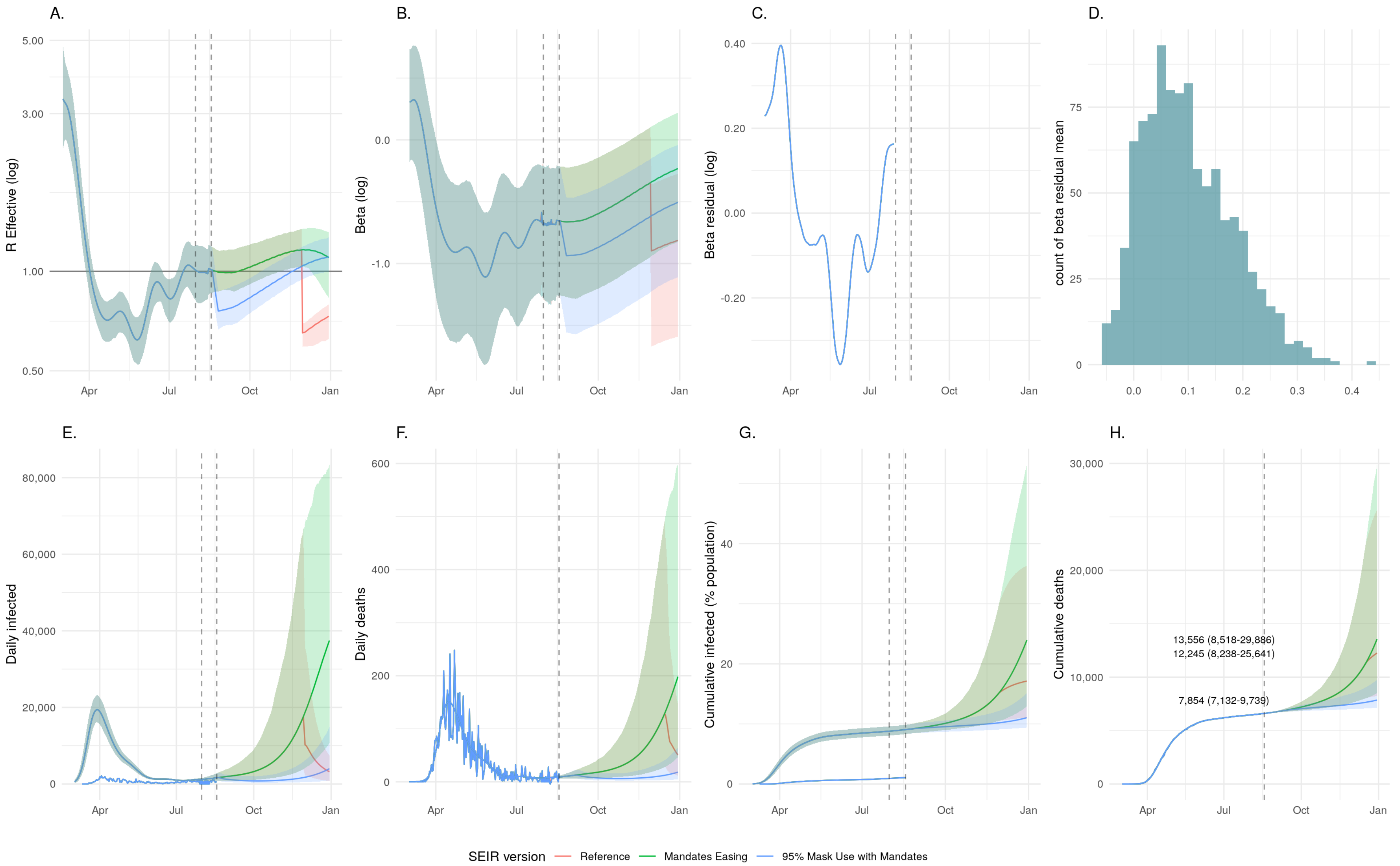

Michigan: SEIR fit comparison. **A**: predicted R effective for each model through December 31. **B**: predicted SEIR *beta* parameter. **C**: residual of predicted *beta* and the observed value calculated directly from infection data over time. **D**: histogram of residual values for *beta*. Panels A, B, C, and D are all displayed in log space, reflecting the space in which the SEIR model is fit. **E**: predicted daily infections from each model through December 31. **F**: predicted daily deaths from each model through December 31. **G**: predicted cumulative infections through December 31, as a proportion of the total population. **H**: predicted cumulative deaths through December 31. In panels E, F, G, and H, reported death and infections are plotted alongside model predictions in light blue.

48 Michigan: Covariate fits and regression coefficients

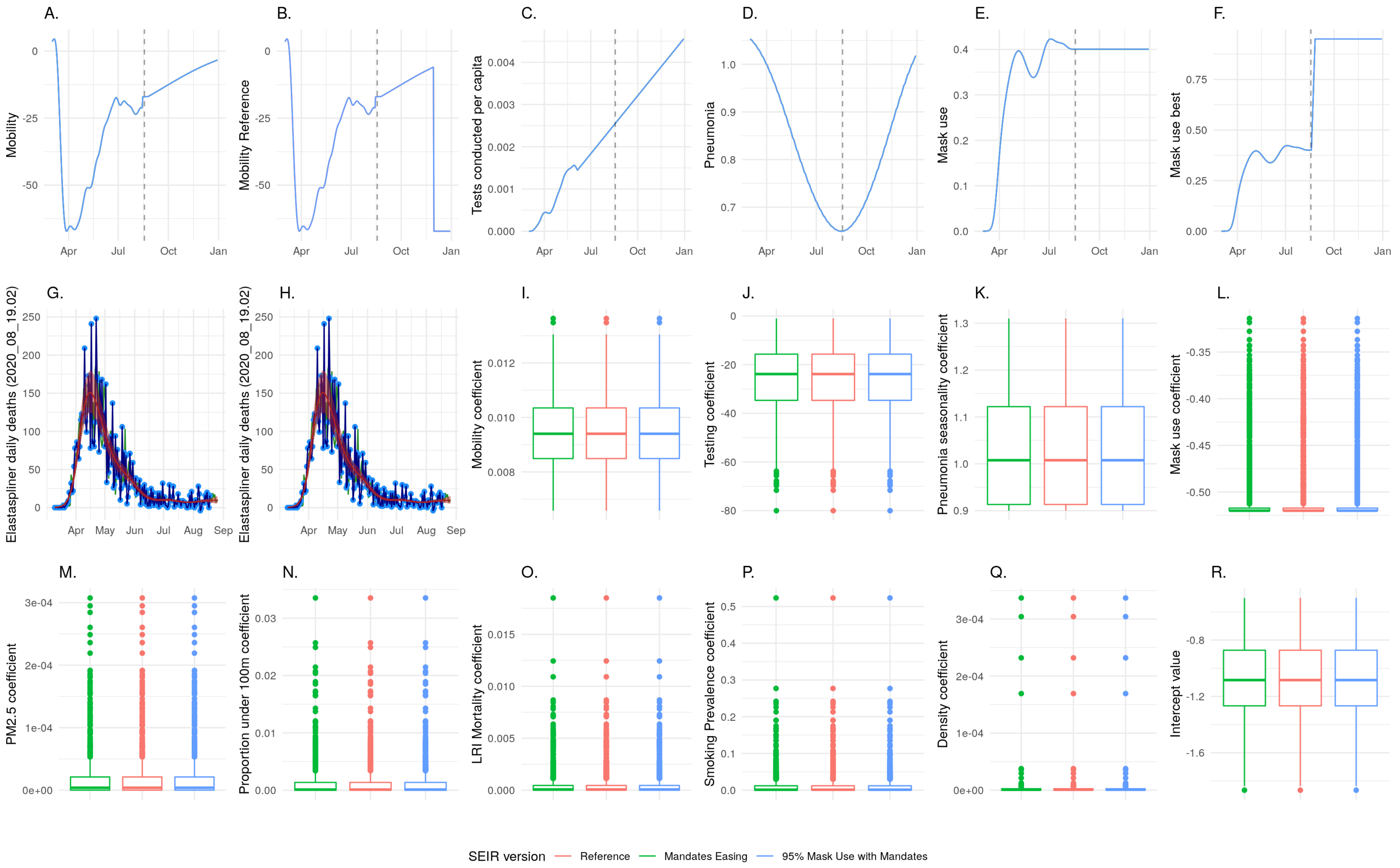

Michigan: Covariate fits and regression coefficients. A-F: Line plots showing predicted covariate time trends for **A)** mobility in the absence of additional mandates; **B)** mobility with additional mandates applied; **C)** diagnostic testing per capita; **D)** pneumonia seasonality; **E)** mask use per capita, and; **F)** mask use in a scenario where adherence increases to 95% of the population. **G-H**: COVID mortality data generated from reported daily deaths (blue); estimated based on reported hospitalizations (purple); estimated from reported cases (green); and via a spline fit through all available data types (red, 95% UI in pink). **I-R**: Box plots showing 1,000 draws of fixed effect coefficients in a multivariate regression fit to $log(beta)$.

49 Minnesota: SEIR fit comparison

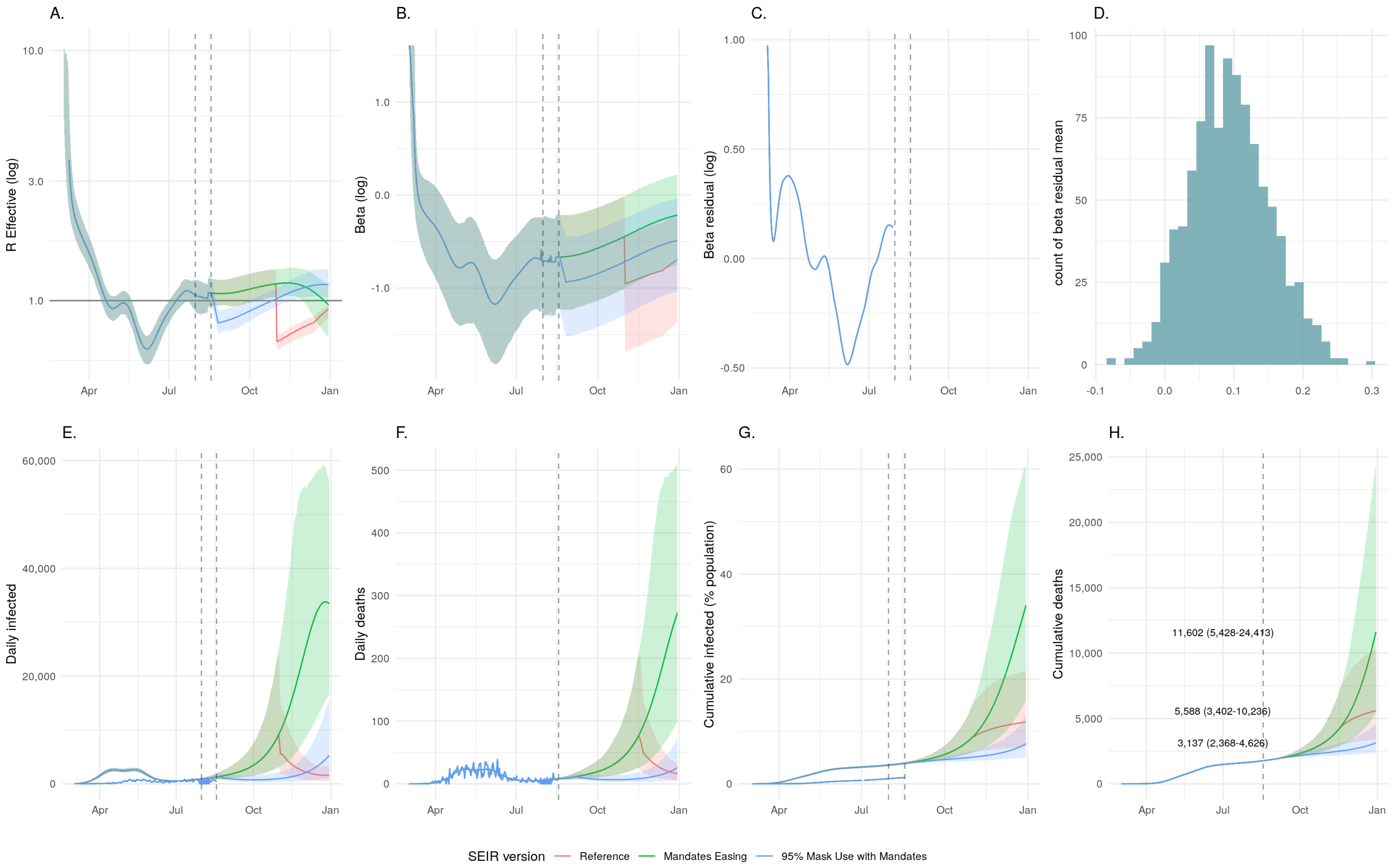

Minnesota: SEIR fit comparison. **A**: predicted R effective for each model through December 31. **B**: predicted SEIR *beta* parameter. **C**: residual of predicted *beta* and the observed value calculated directly from infection data over time. **D**: histogram of residual values for *beta*. Panels A, B, C, and D are all displayed in log space, reflecting the space in which the SEIR model is fit. **E**: predicted daily infections from each model through December 31. **F**: predicted daily deaths from each model through December 31. **G**: predicted cumulative infections through December 31, as a proportion of the total population. **H**: predicted cumulative deaths through December 31. In panels E, F, G, and H, reported death and infections are plotted alongside model predictions in light blue.

50 Minnesota: Covariate fits and regression coefficients

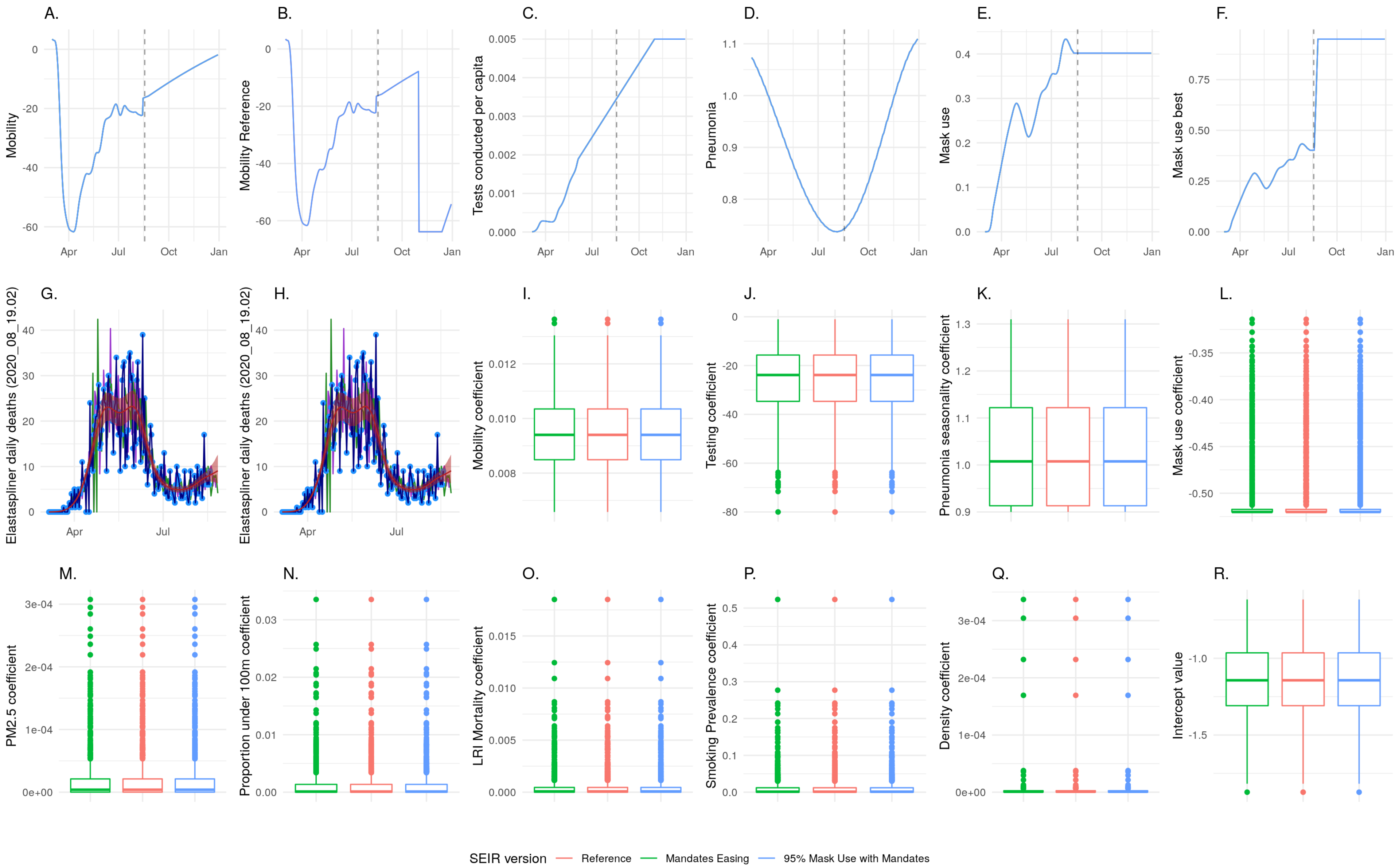

Minnesota: Covariate fits and regression coefficients. A-F: Line plots showing predicted covariate time trends for **A)** mobility in the absence of additional mandates; **B)** mobility with additional mandates applied; **C)** diagnostic testing per capita; **D)** pneumonia seasonality; **E)** mask use per capita, and; **F)** mask use in a scenario where adherence increases to 95% of the population. **G-H**: COVID mortality data generated from reported daily deaths (blue); estimated based on reported hospitalizations (purple); estimated from reported cases (green); and via a spline fit through all available data types (red, 95% UI in pink). **I-R**: Box plots showing 1,000 draws of fixed effect coefficients in a multivariate regression fit to $log(beta)$.

51 Mississippi: SEIR fit comparison

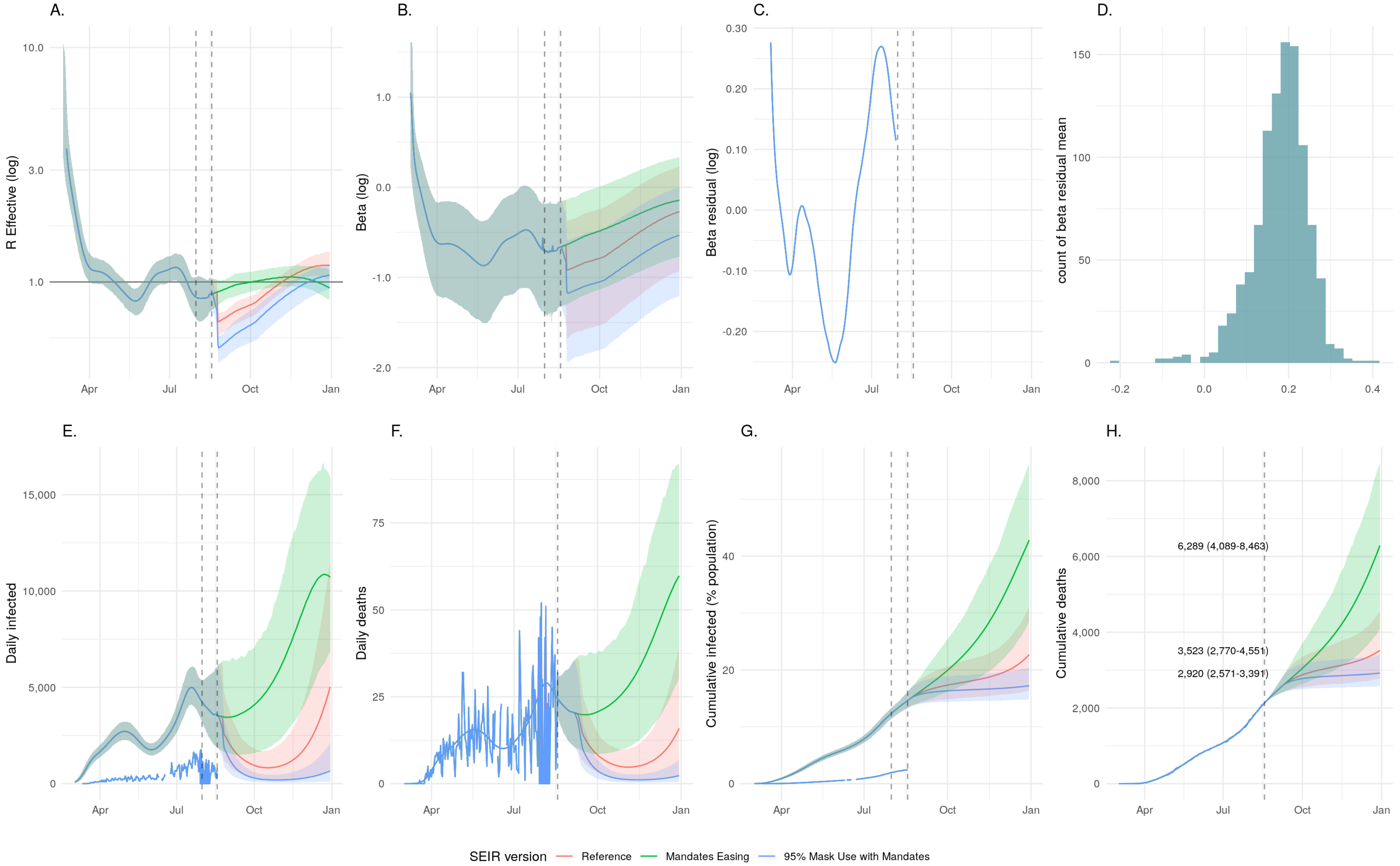

Mississippi: SEIR fit comparison. **A**: predicted R effective for each model through December 31. **B**: predicted SEIR *beta* parameter. **C**: residual of predicted *beta* and the observed value calculated directly from infection data over time. **D**: histogram of residual values for *beta*. Panels A, B, C, and D are all displayed in log space, reflecting the space in which the SEIR model is fit. **E**: predicted daily infections from each model through December 31. **F**: predicted daily deaths from each model through December 31. **G**: predicted cumulative infections through December 31, as a proportion of the total population. **H**: predicted cumulative deaths through December 31. In panels E, F, G, and H, reported death and infections are plotted alongside model predictions in light blue.

52 Mississippi: Covariate fits and regression coefficients

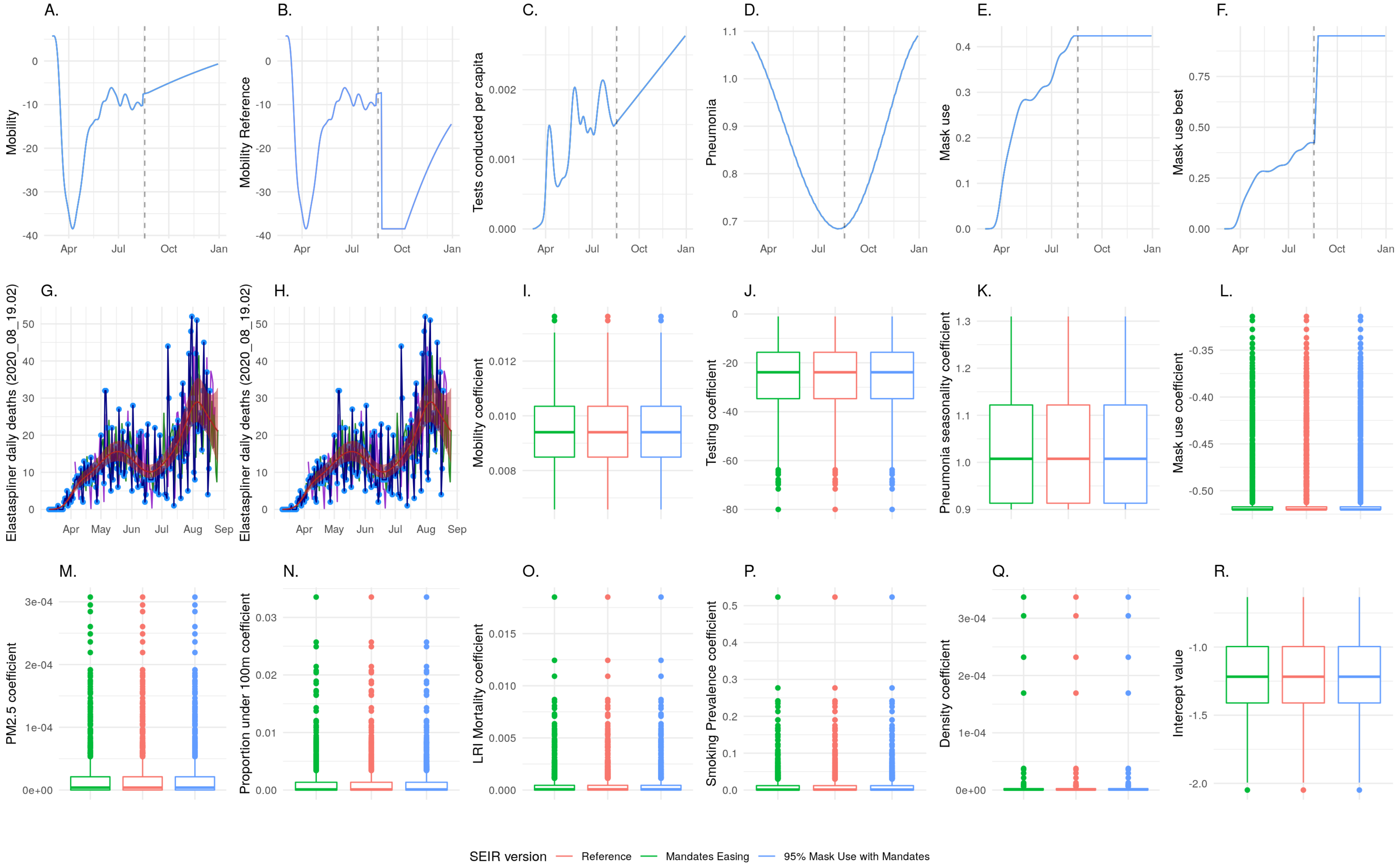

Mississippi: Covariate fits and regression coefficients. A-F: Line plots showing predicted covariate time trends for **A)** mobility in the absence of additional mandates; **B)** mobility with additional mandates applied; **C)** diagnostic testing per capita; **D)** pneumonia seasonality; **E)** mask use per capita, and; **F)** mask use in a scenario where adherence increases to 95% of the population. **G-H**: COVID mortality data generated from reported daily deaths (blue); estimated based on reported hospitalizations (purple); estimated from reported cases (green); and via a spline fit through all available data types (red, 95% UI in pink). **I-R**: Box plots showing 1,000 draws of fixed effect coefficients in a multivariate regression fit to $log(beta)$.

53 Missouri: SEIR fit comparison

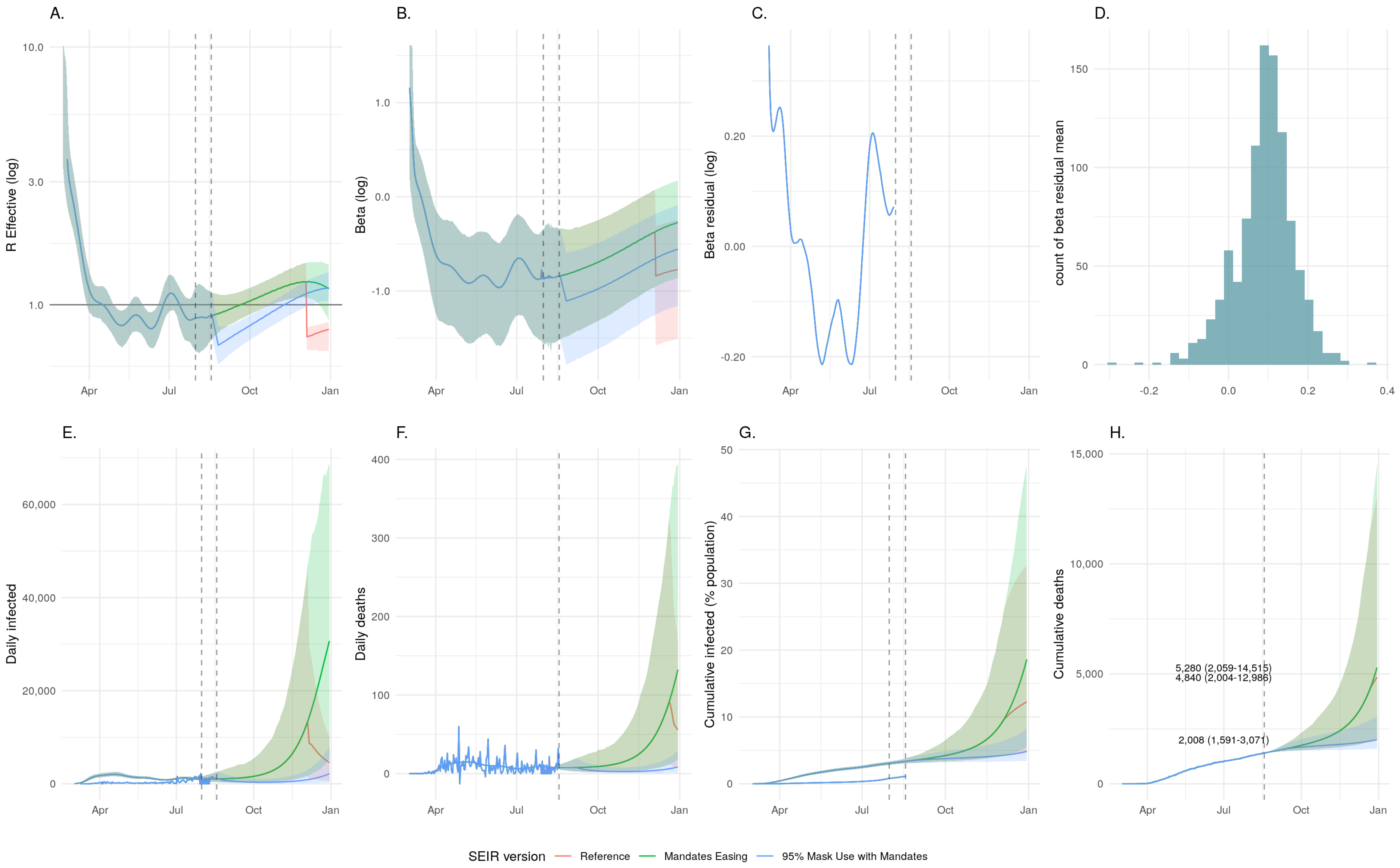

Missouri: SEIR fit comparison. A: predicted R effective for each model through December 31. **B**: predicted SEIR *beta* parameter. **C**: residual of predicted *beta* and the observed value calculated directly from infection data over time. **D**: histogram of residual values for *beta*. Panels A, B, C, and D are all displayed in log space, reflecting the space in which the SEIR model is fit. **E**: predicted daily infections from each model through December 31. **F**: predicted daily deaths from each model through December 31. **G**: predicted cumulative infections through December 31, as a proportion of the total population. **H**: predicted cumulative deaths through December 31. In panels E, F, G, and H, reported death and infections are plotted alongside model predictions in light blue.

54 Missouri: Covariate fits and regression coefficients

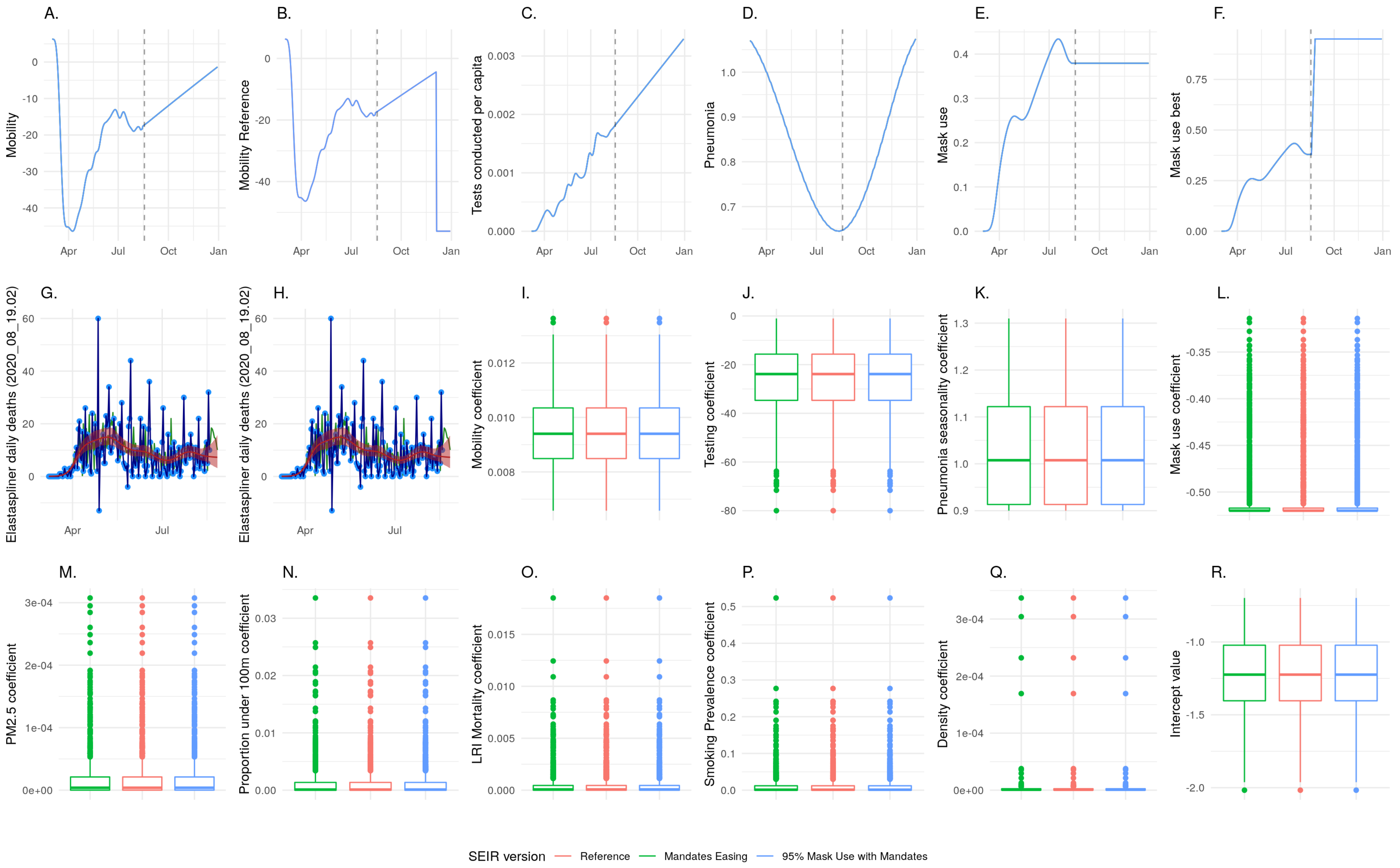

Missouri: Covariate fits and regression coefficients. A-F: Line plots showing predicted covariate time trends for **A)** mobility in the absence of additional mandates; **B)** mobility with additional mandates applied; **C)** diagnostic testing per capita; **D)** pneumonia seasonality; **E)** mask use per capita, and; **F)** mask use in a scenario where adherence increases to 95% of the population. **G-H**: COVID mortality data generated from reported daily deaths (blue); estimated based on reported hospitalizations (purple); estimated from reported cases (green); and via a spline fit through all available data types (red, 95% UI in pink). **I-R**: Box plots showing 1,000 draws of fixed effect coefficients in a multivariate regression fit to $log(beta)$.

55 Montana: SEIR fit comparison

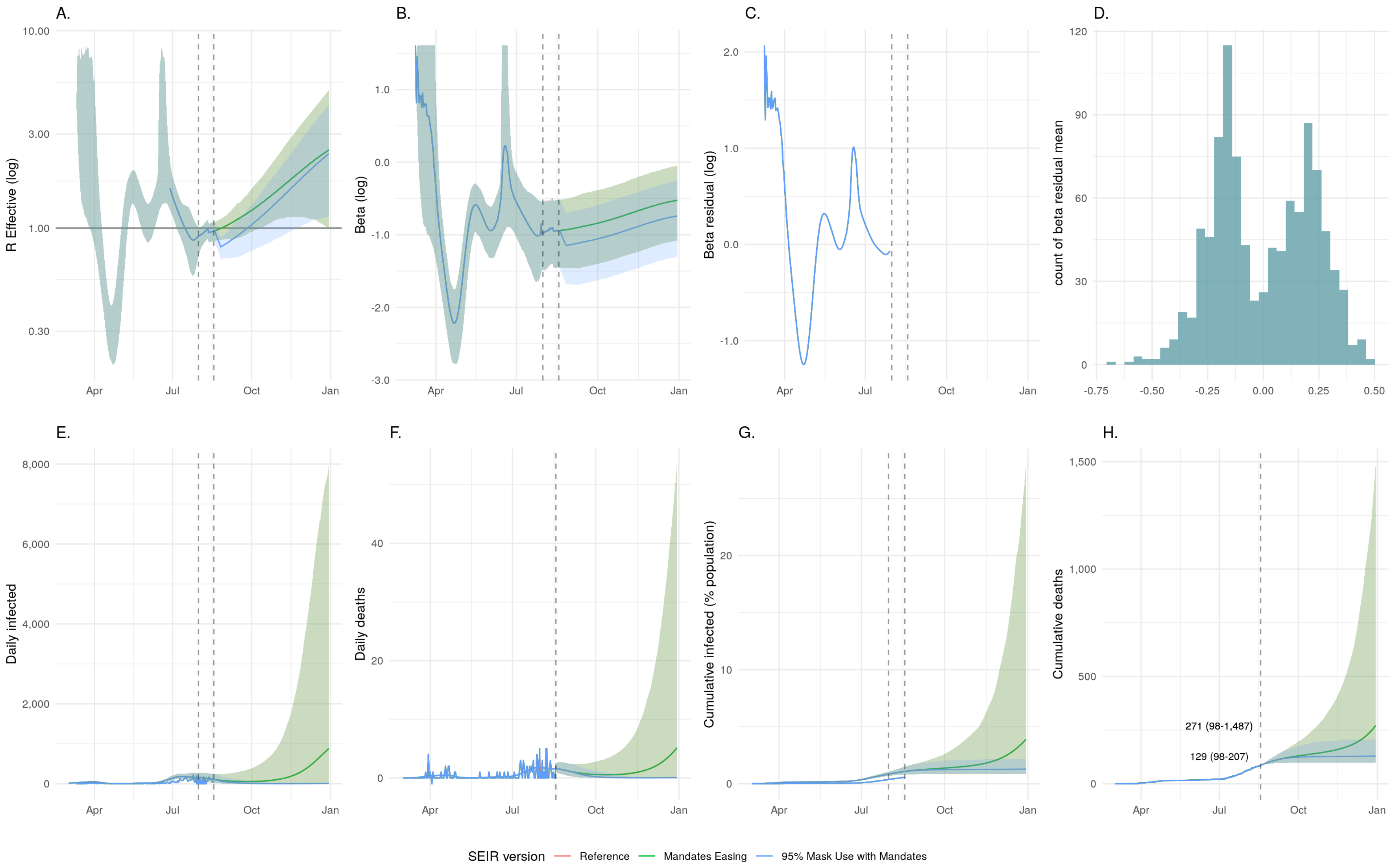

Montana: SEIR fit comparison. A: predicted R effective for each model through December 31. **B**: predicted SEIR *beta* parameter. **C**: residual of predicted *beta* and the observed value calculated directly from infection data over time. **D**: histogram of residual values for *beta*. Panels A, B, C, and D are all displayed in log space, reflecting the space in which the SEIR model is fit. **E**: predicted daily infections from each model through December 31. **F**: predicted daily deaths from each model through December 31. **G**: predicted cumulative infections through December 31, as a proportion of the total population. **H**: predicted cumulative deaths through December 31. In panels E, F, G, and H, reported death and infections are plotted alongside model predictions in light blue.

56 Montana: Covariate fits and regression coefficients

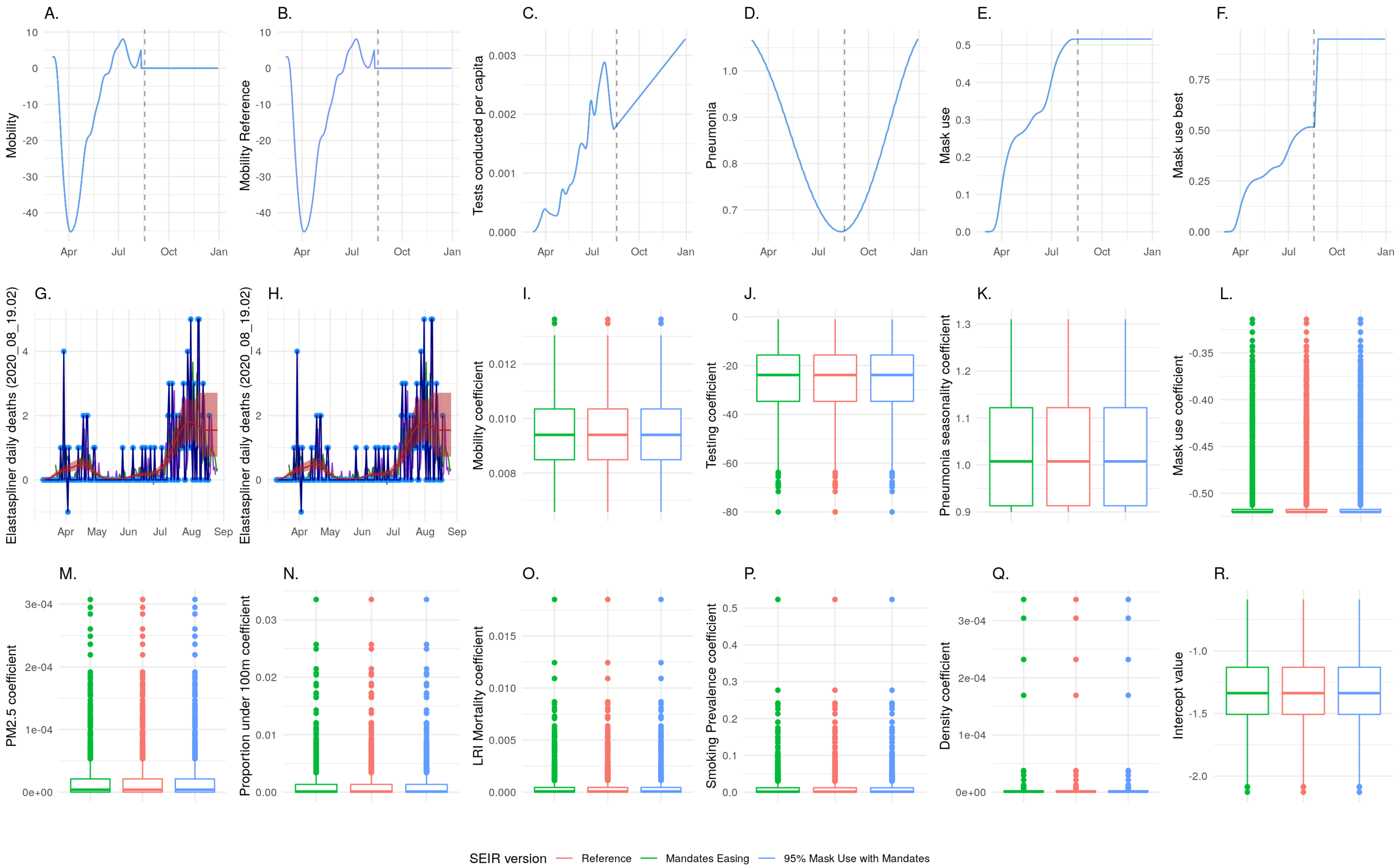

Montana: Covariate fits and regression coefficients. A-F: Line plots showing predicted covariate time trends for **A)** mobility in the absence of additional mandates; **B)** mobility with additional mandates applied; **C)** diagnostic testing per capita; **D)** pneumonia seasonality; **E)** mask use per capita, and; **F)** mask use in a scenario where adherence increases to 95% of the population. **G-H**: COVID mortality data generated from reported daily deaths (blue); estimated based on reported hospitalizations (purple); estimated from reported cases (green); and via a spline fit through all available data types (red, 95% UI in pink). **I-R**: Box plots showing 1,000 draws of fixed effect coefficients in a multivariate regression fit to $log(beta)$.

57 Nebraska: SEIR fit comparison

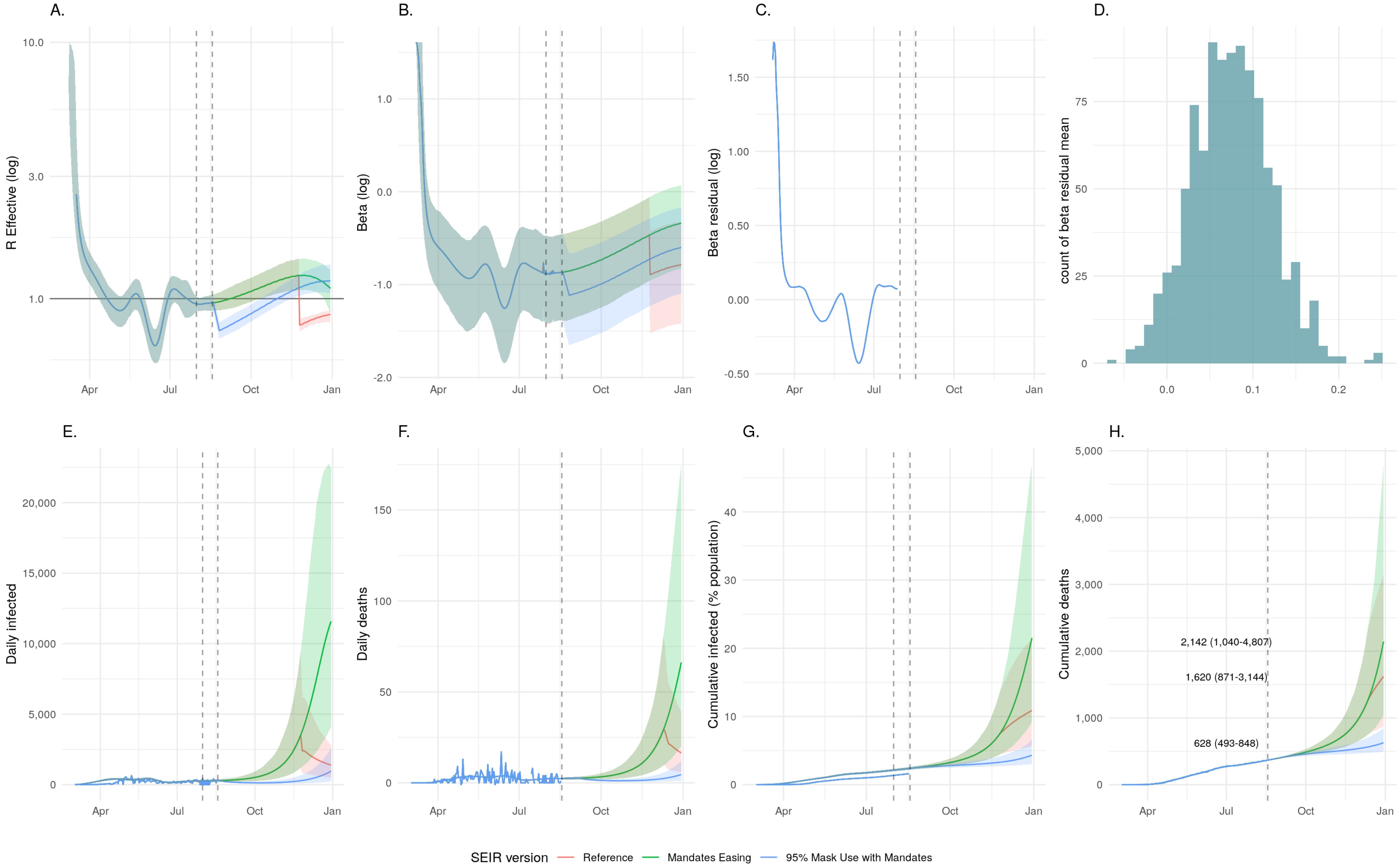

Nebraska: SEIR fit comparison. **A**: predicted R effective for each model through December 31. **B**: predicted SEIR *beta* parameter. **C**: residual of predicted *beta* and the observed value calculated directly from infection data over time. **D**: histogram of residual values for *beta*. Panels A, B, C, and D are all displayed in log space, reflecting the space in which the SEIR model is fit. **E**: predicted daily infections from each model through December 31. **F**: predicted daily deaths from each model through December 31. **G**: predicted cumulative infections through December 31, as a proportion of the total population. **H**: predicted cumulative deaths through December 31. In panels E, F, G, and H, reported death and infections are plotted alongside model predictions in light blue.

58 Nebraska: Covariate fits and regression coefficients

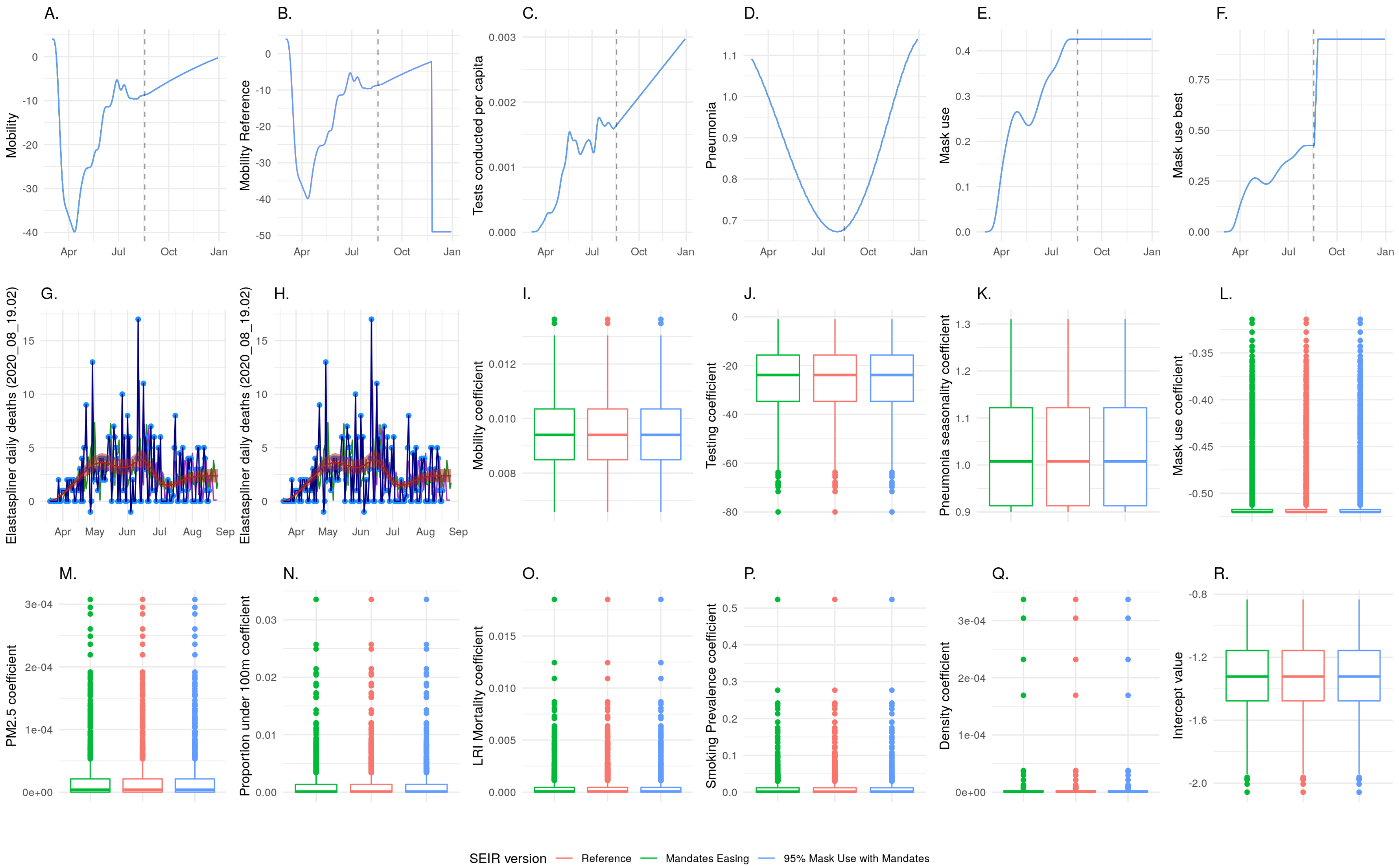

Nebraska: Covariate fits and regression coefficients. **A-F**: Line plots showing predicted covariate time trends for **A)** mobility in the absence of additional mandates; **B)** mobility with additional mandates applied; **C)** diagnostic testing per capita; **D)** pneumonia seasonality; **E)** mask use per capita, and; **F)** mask use in a scenario where adherence increases to 95% of the population. **G-H**: COVID mortality data generated from reported daily deaths (blue); estimated based on reported hospitalizations (purple); estimated from reported cases (green); and via a spline fit through all available data types (red, 95% UI in pink). **I-R**: Box plots showing 1,000 draws of fixed effect coefficients in a multivariate regression fit to $log(beta)$.

59 Nevada: SEIR fit comparison

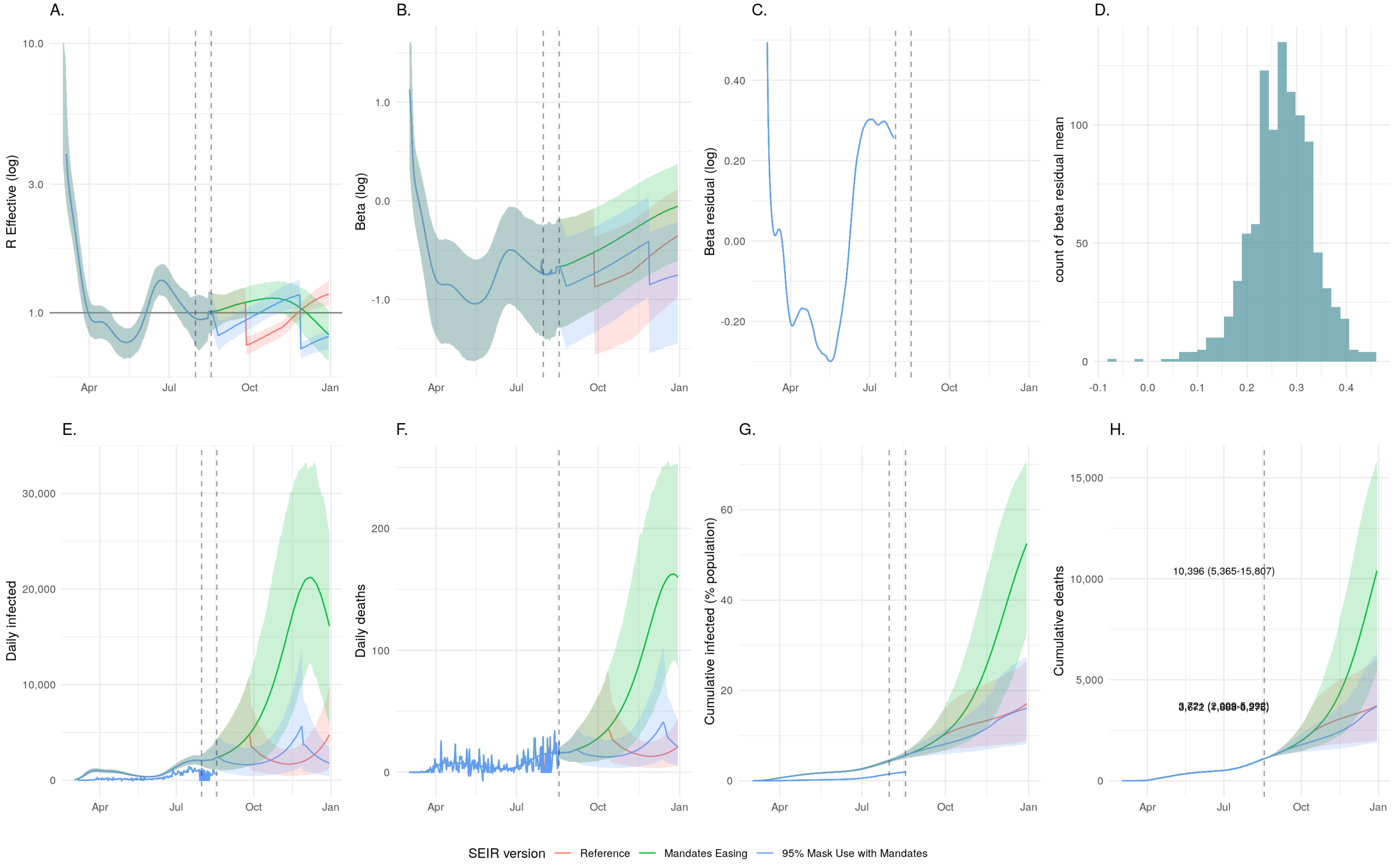

Nevada: SEIR fit comparison. **A**: predicted R effective for each model through December 31. **B**: predicted SEIR *beta* parameter. **C**: residual of predicted *beta* and the observed value calculated directly from infection data over time. **D**: histogram of residual values for *beta*. Panels A, B, C, and D are all displayed in log space, reflecting the space in which the SEIR model is fit. **E**: predicted daily infections from each model through December 31. **F**: predicted daily deaths from each model through December 31. **G**: predicted cumulative infections through December 31, as a proportion of the total population. **H**: predicted cumulative deaths through December 31. In panels E, F, G, and H, reported death and infections are plotted alongside model predictions in light blue.

60 Nevada: Covariate fits and regression coefficients

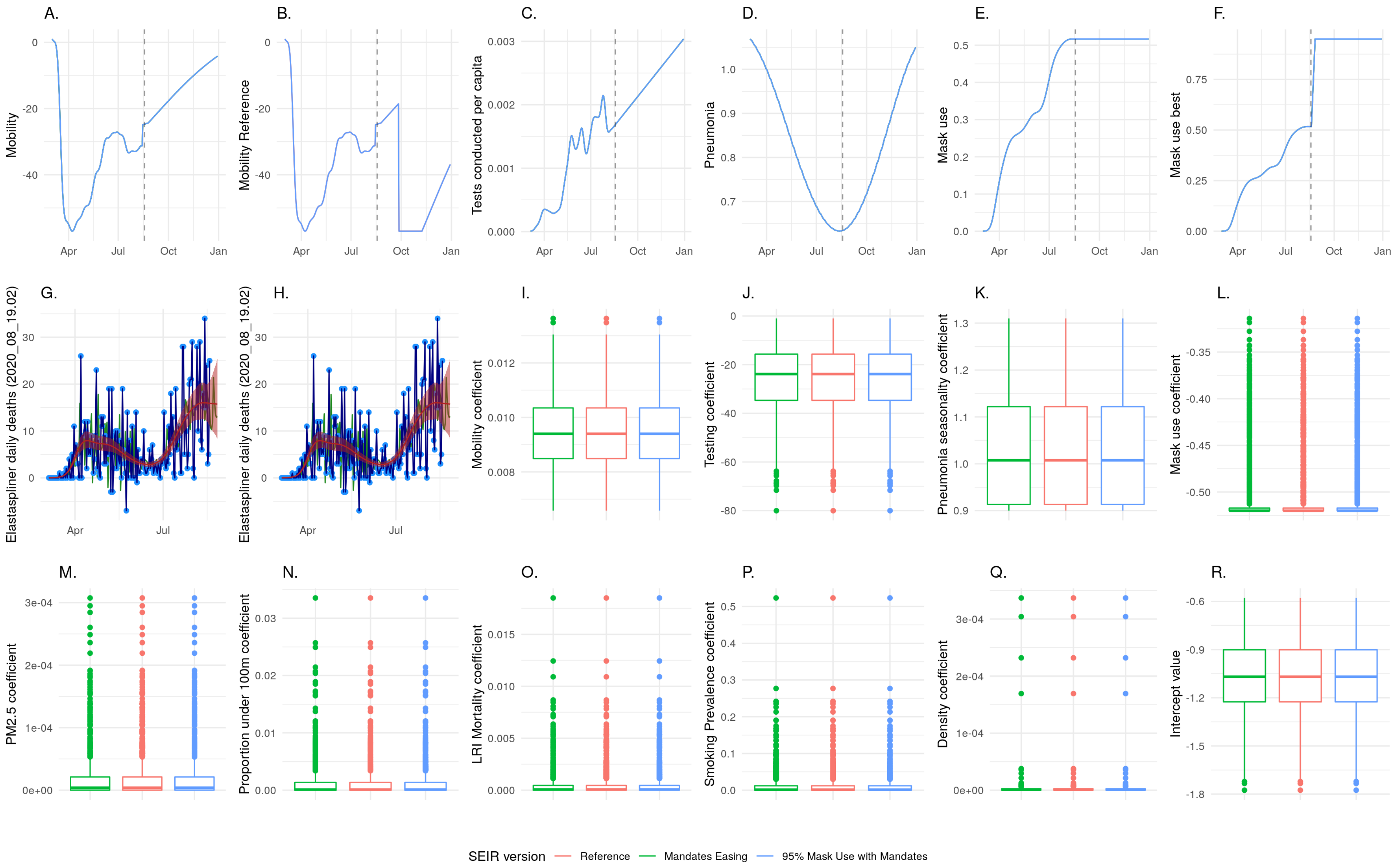

Nevada: Covariate fits and regression coefficients. A-F: Line plots showing predicted covariate time trends for **A)** mobility in the absence of additional mandates; **B)** mobility with additional mandates applied; **C)** diagnostic testing per capita; **D)** pneumonia seasonality; **E)** mask use per capita, and; **F)** mask use in a scenario where adherence increases to 95% of the population. **G-H**: COVID mortality data generated from reported daily deaths (blue); estimated based on reported hospitalizations (purple); estimated from reported cases (green); and via a spline fit through all available data types (red, 95% UI in pink). **I-R**: Box plots showing 1,000 draws of fixed effect coefficients in a multivariate regression fit to $log(beta)$.

61 New Hampshire: SEIR fit comparison

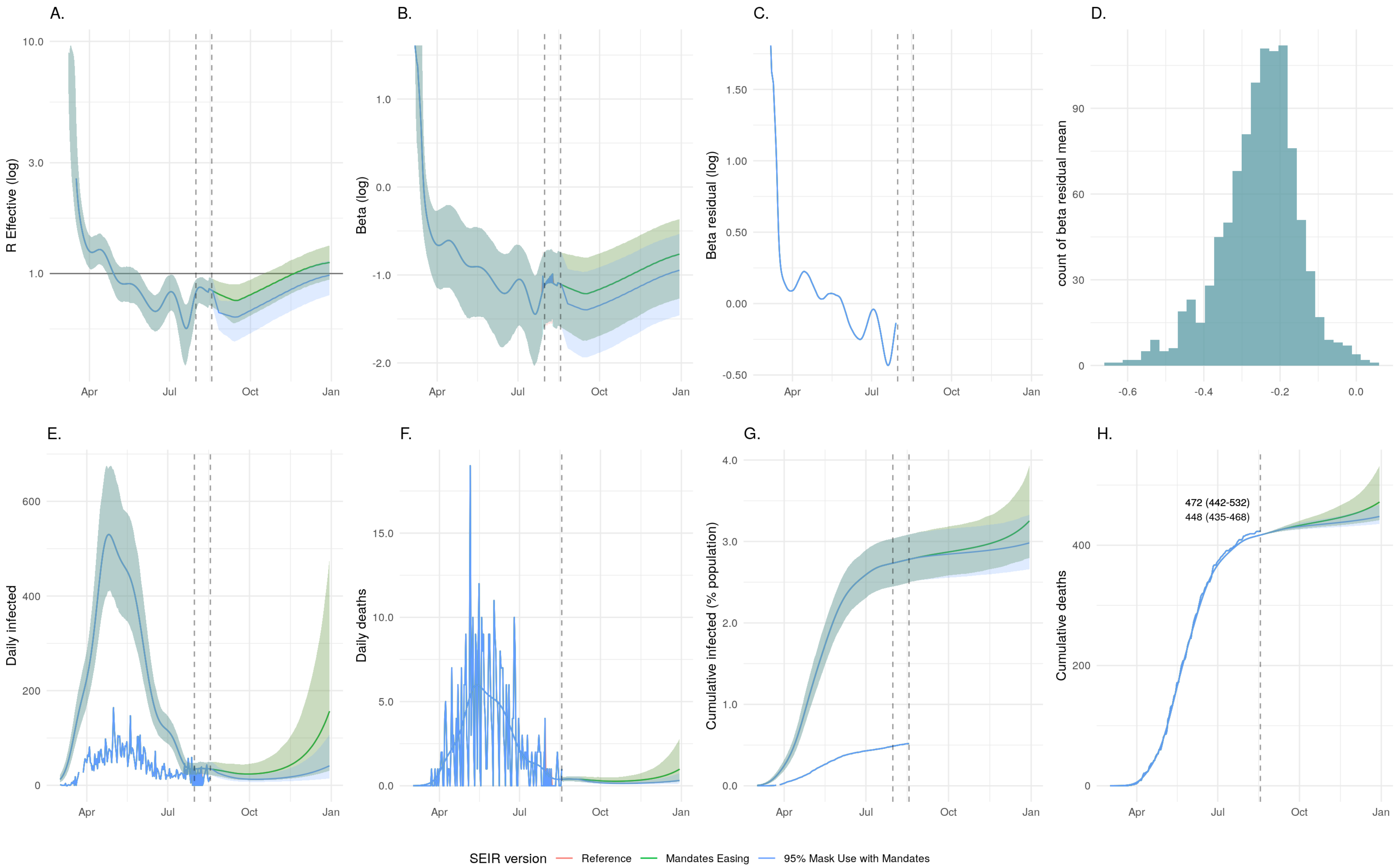

New Hampshire: SEIR fit comparison. A: predicted R effective for each model through December 31. **B**: predicted SEIR *beta* parameter. **C**: residual of predicted *beta* and the observed value calculated directly from infection data over time. **D**: histogram of residual values for *beta*. Panels A, B, C, and D are all displayed in log space, reflecting the space in which the SEIR model is fit. **E**: predicted daily infections from each model through December 31. **F**: predicted daily deaths from each model through December 31. **G**: predicted cumulative infections through December 31, as a proportion of the total population. **H**: predicted cumulative deaths through December 31. In panels E, F, G, and H, reported death and infections are plotted alongside model predictions in light blue.

62 New Hampshire: Covariate fits and regression coefficients

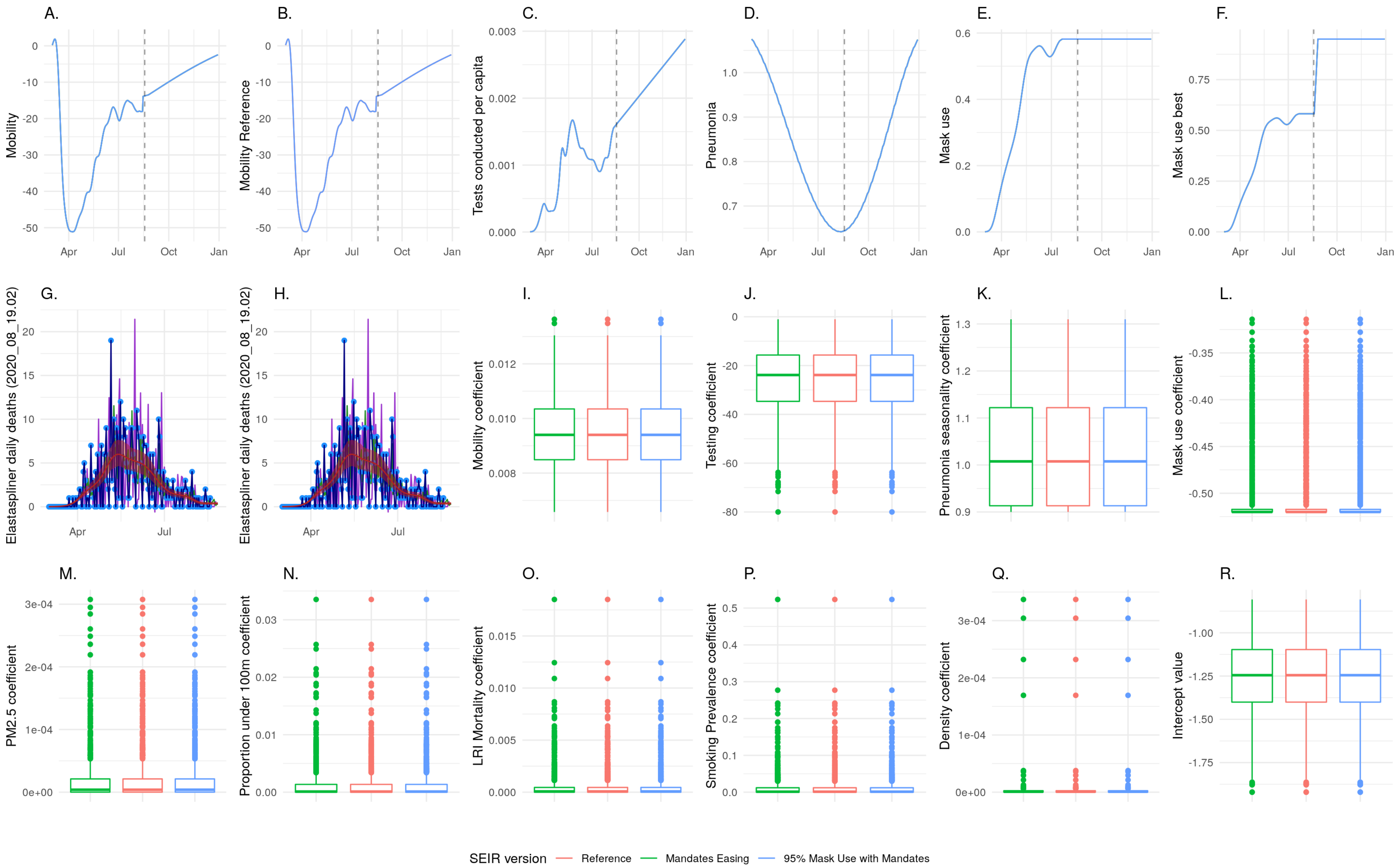

New Hampshire: Covariate fits and regression coefficients. A-F: Line plots showing predicted covariate time trends for **A)** mobility in the absence of additional mandates; **B)** mobility with additional mandates applied; **C)** diagnostic testing per capita; **D)** pneumonia seasonality; **E)** mask use per capita, and; **F)** mask use in a scenario where adherence increases to 95% of the population. **G-H**: COVID mortality data generated from reported daily deaths (blue); estimated based on reported hospitalizations (purple); estimated from reported cases (green); and via a spline fit through all available data types (red, 95% UI in pink). **I-R**: Box plots showing 1,000 draws of fixed effect coefficients in a multivariate regression fit to $log(beta)$.

63 New Jersey: SEIR fit comparison

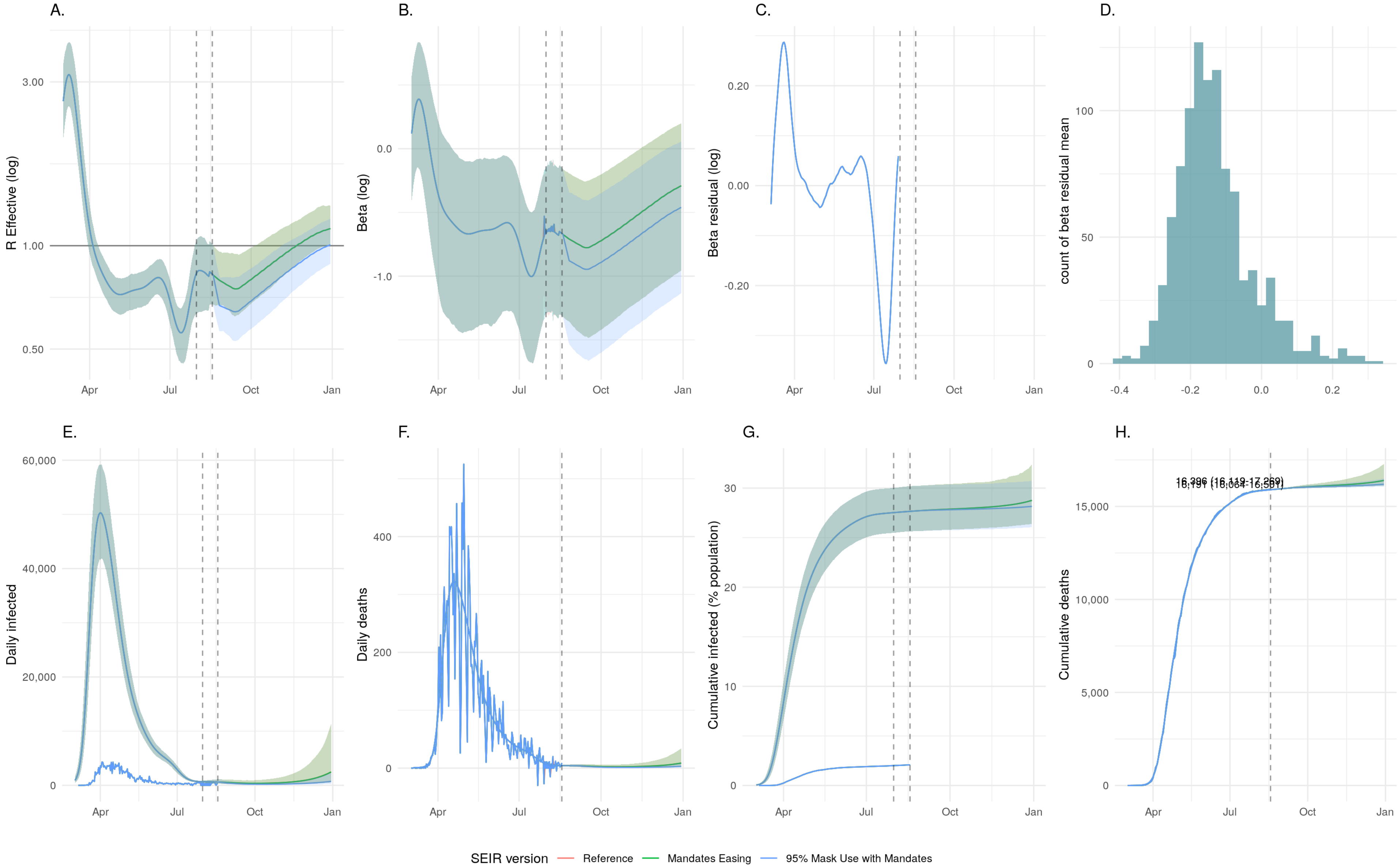

New Jersey: SEIR fit comparison. **A**: predicted R effective for each model through December 31. **B**: predicted SEIR *beta* parameter. **C**: residual of predicted *beta* and the observed value calculated directly from infection data over time. **D**: histogram of residual values for *beta*. Panels A, B, C, and D are all displayed in log space, reflecting the space in which the SEIR model is fit. **E**: predicted daily infections from each model through December 31. **F**: predicted daily deaths from each model through December 31. **G**: predicted cumulative infections through December 31, as a proportion of the total population. **H**: predicted cumulative deaths through December 31. In panels E, F, G, and H, reported death and infections are plotted alongside model predictions in light blue.

64 New Jersey: Covariate fits and regression coefficients

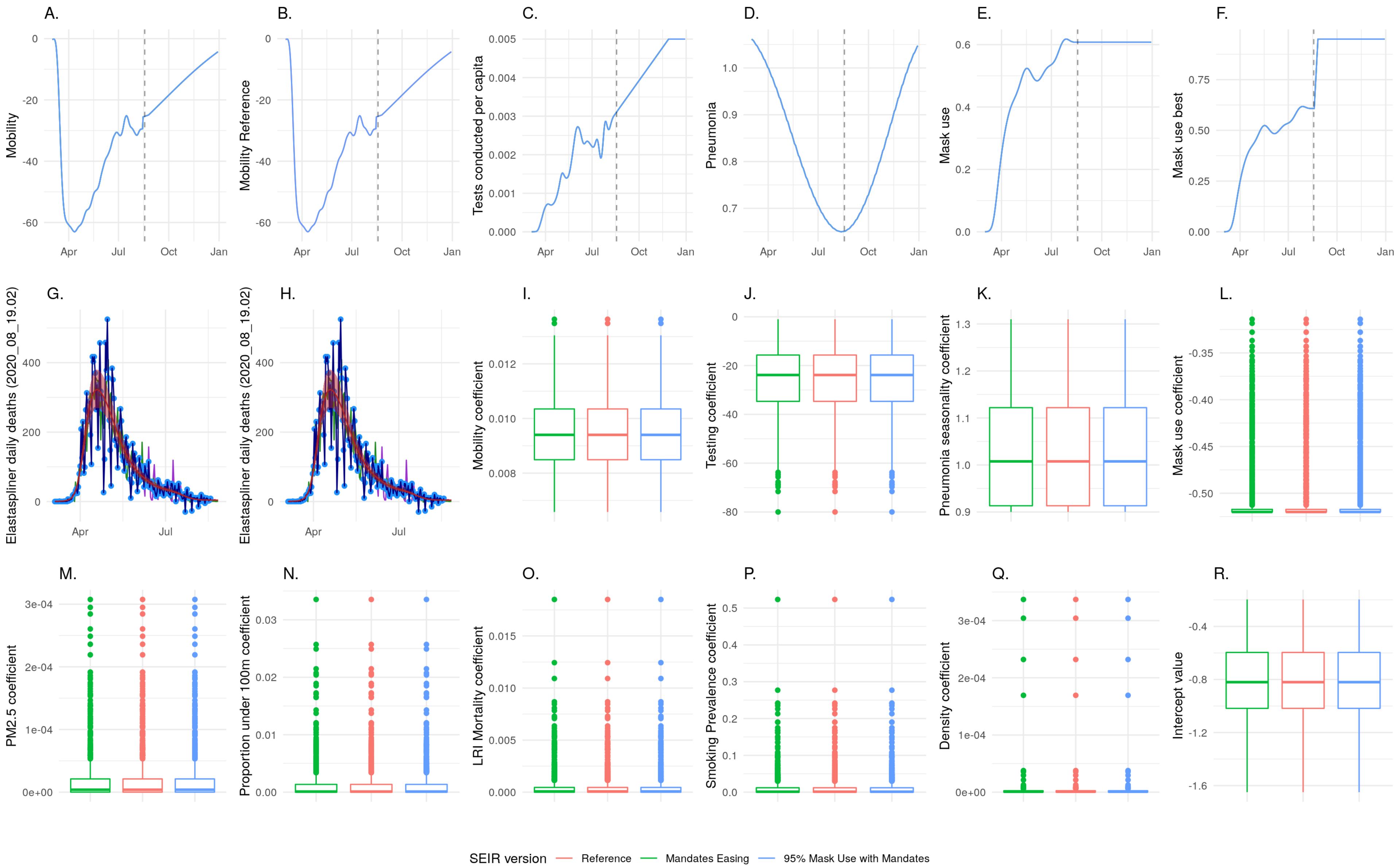

New Jersey: Covariate fits and regression coefficients. A-F: Line plots showing predicted covariate time trends for **A)** mobility in the absence of additional mandates; **B)** mobility with additional mandates applied; **C)** diagnostic testing per capita; **D)** pneumonia seasonality; **E)** mask use per capita, and; **F)** mask use in a scenario where adherence increases to 95% of the population. **G-H**: COVID mortality data generated from reported daily deaths (blue); estimated based on reported hospitalizations (purple); estimated from reported cases (green); and via a spline fit through all available data types (red, 95% UI in pink). **I-R**: Box plots showing 1,000 draws of fixed effect coefficients in a multivariate regression fit to $log(beta)$.

65 New Mexico: SEIR fit comparison

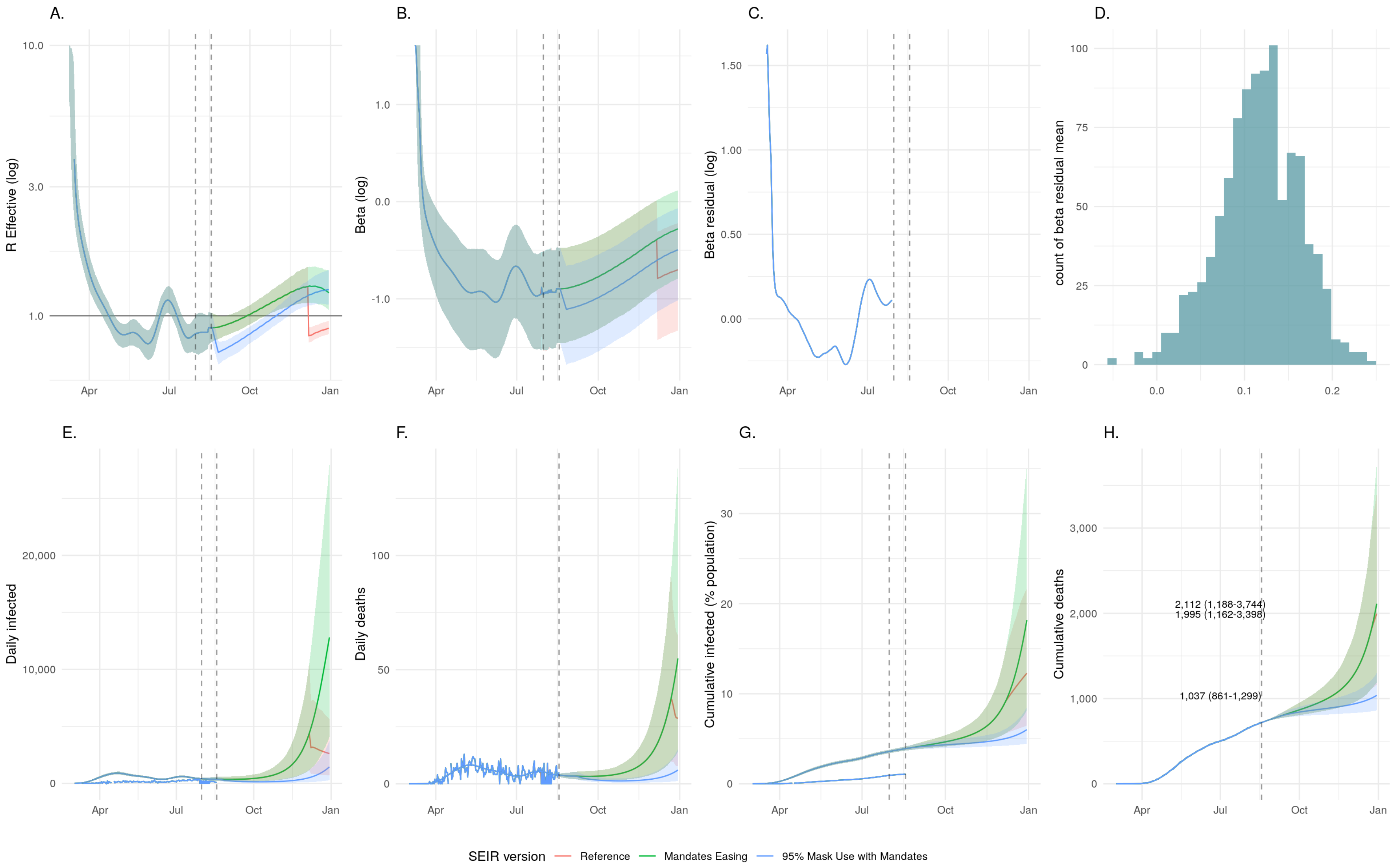

New Mexico: SEIR fit comparison. A: predicted R effective for each model through December 31. **B**: predicted SEIR *beta* parameter. **C**: residual of predicted *beta* and the observed value calculated directly from infection data over time. **D**: histogram of residual values for *beta*. Panels A, B, C, and D are all displayed in log space, reflecting the space in which the SEIR model is fit. **E**: predicted daily infections from each model through December 31. **F**: predicted daily deaths from each model through December 31. **G**: predicted cumulative infections through December 31, as a proportion of the total population. **H**: predicted cumulative deaths through December 31. In panels E, F, G, and H, reported death and infections are plotted alongside model predictions in light blue.

66 New Mexico: Covariate fits and regression coefficients

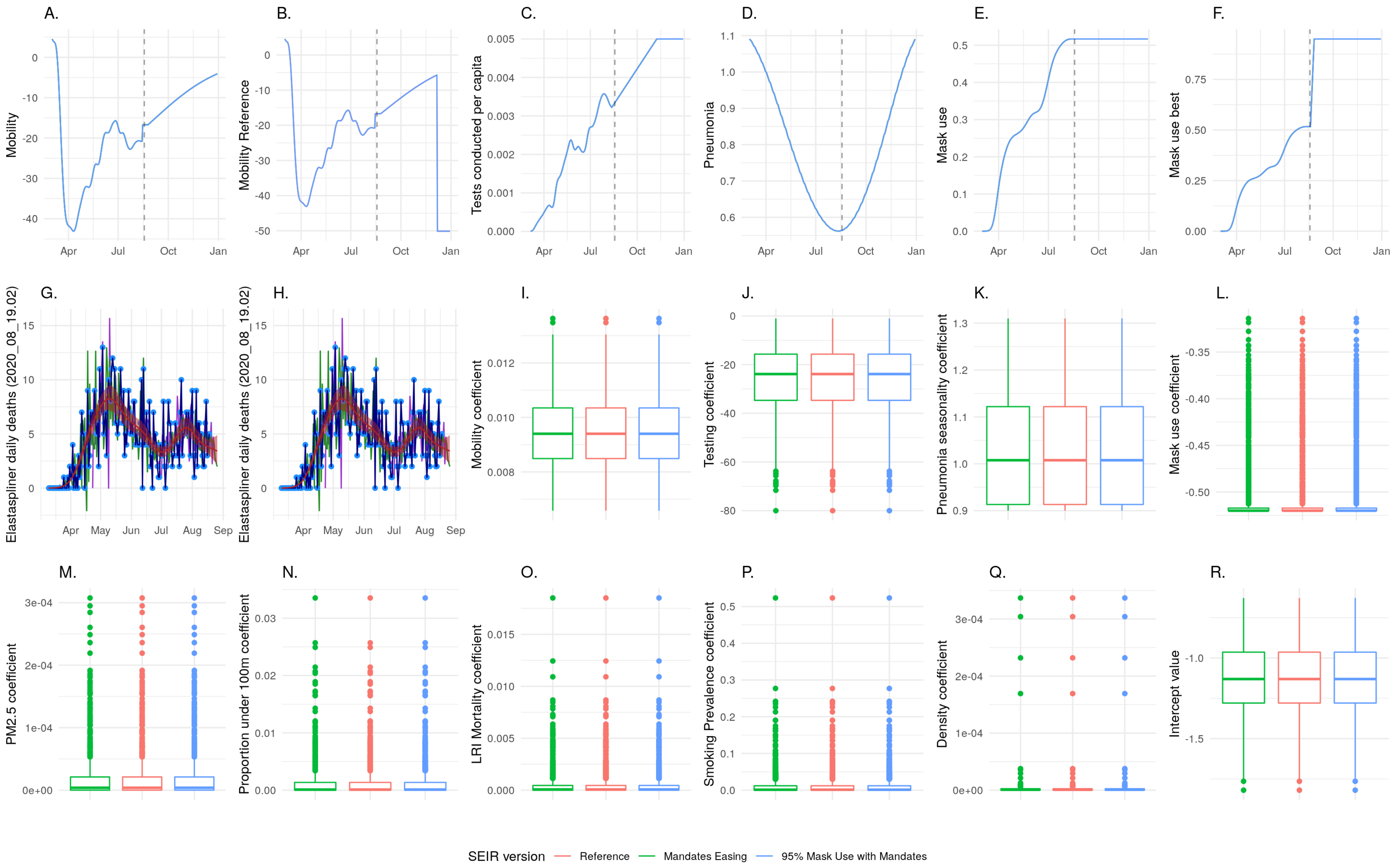

New Mexico: Covariate fits and regression coefficients. **A-F**: Line plots showing predicted covariate time trends for **A)** mobility in the absence of additional mandates; **B)** mobility with additional mandates applied; **C)** diagnostic testing per capita; **D)** pneumonia seasonality; **E)** mask use per capita, and; **F)** mask use in a scenario where adherence increases to 95% of the population. **G-H**: COVID mortality data generated from reported daily deaths (blue); estimated based on reported hospitalizations (purple); estimated from reported cases (green); and via a spline fit through all available data types (red, 95% UI in pink). **I-R**: Box plots showing 1,000 draws of fixed effect coefficients in a multivariate regression fit to $log(beta)$.

67 New York: SEIR fit comparison

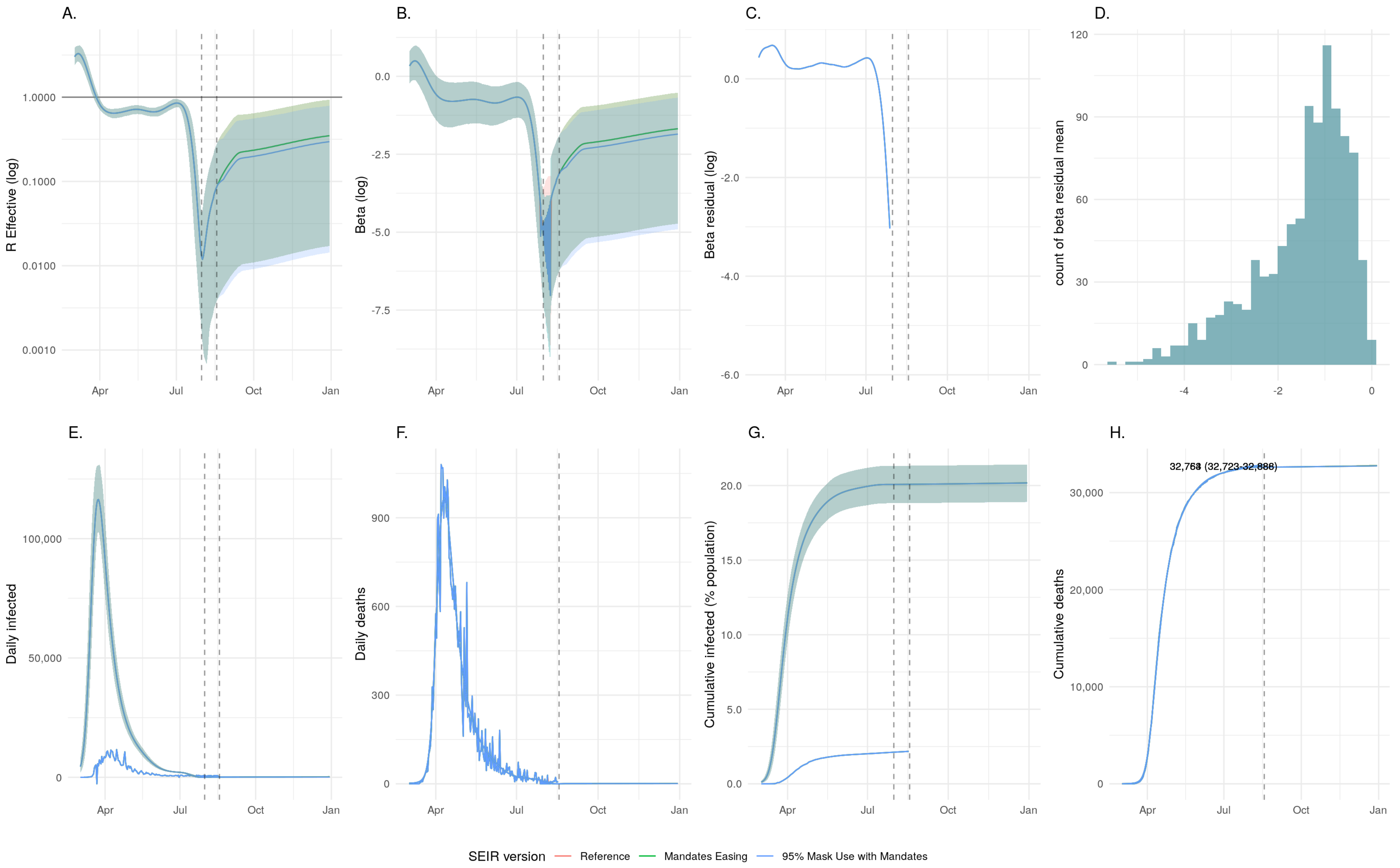

New York: SEIR fit comparison. A: predicted R effective for each model through December 31. **B**: predicted SEIR *beta* parameter. **C**: residual of predicted *beta* and the observed value calculated directly from infection data over time. **D**: histogram of residual values for *beta*. Panels A, B, C, and D are all displayed in log space, reflecting the space in which the SEIR model is fit. **E**: predicted daily infections from each model through December 31. **F**: predicted daily deaths from each model through December 31. **G**: predicted cumulative infections through December 31, as a proportion of the total population. **H**: predicted cumulative deaths through December 31. In panels E, F, G, and H, reported death and infections are plotted alongside model predictions in light blue.

68 New York: Covariate fits and regression coefficients

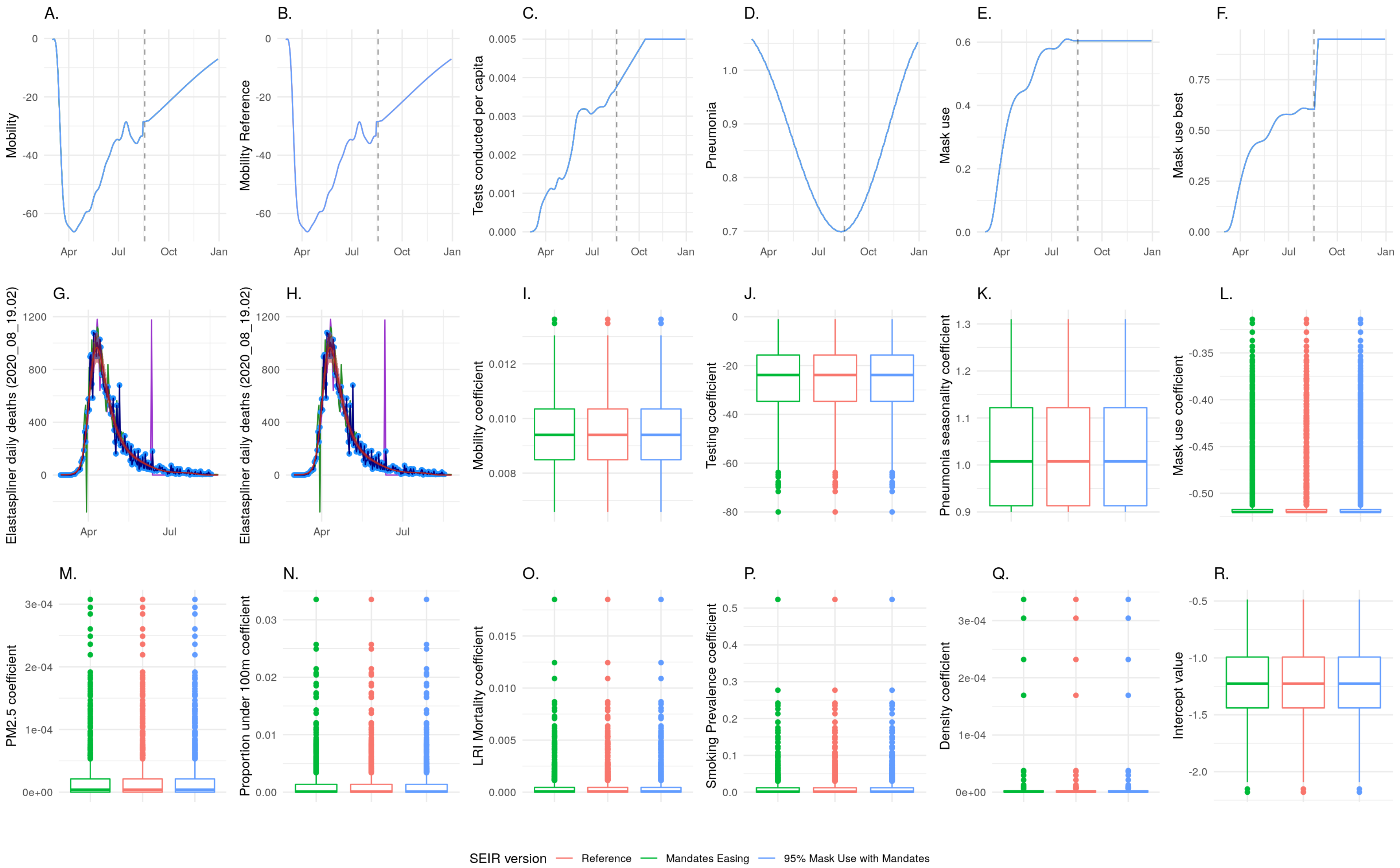

New York: Covariate fits and regression coefficients. **A-F**: Line plots showing predicted covariate time trends for **A)** mobility in the absence of additional mandates; **B)** mobility with additional mandates applied; **C)** diagnostic testing per capita; **D)** pneumonia seasonality; **E)** mask use per capita, and; **F)** mask use in a scenario where adherence increases to 95% of the population. **G-H**: COVID mortality data generated from reported daily deaths (blue); estimated based on reported hospitalizations (purple); estimated from reported cases (green); and via a spline fit through all available data types (red, 95% UI in pink). **I-R**: Box plots showing 1,000 draws of fixed effect coefficients in a multivariate regression fit to $log(beta)$.

69 North Carolina: SEIR fit comparison

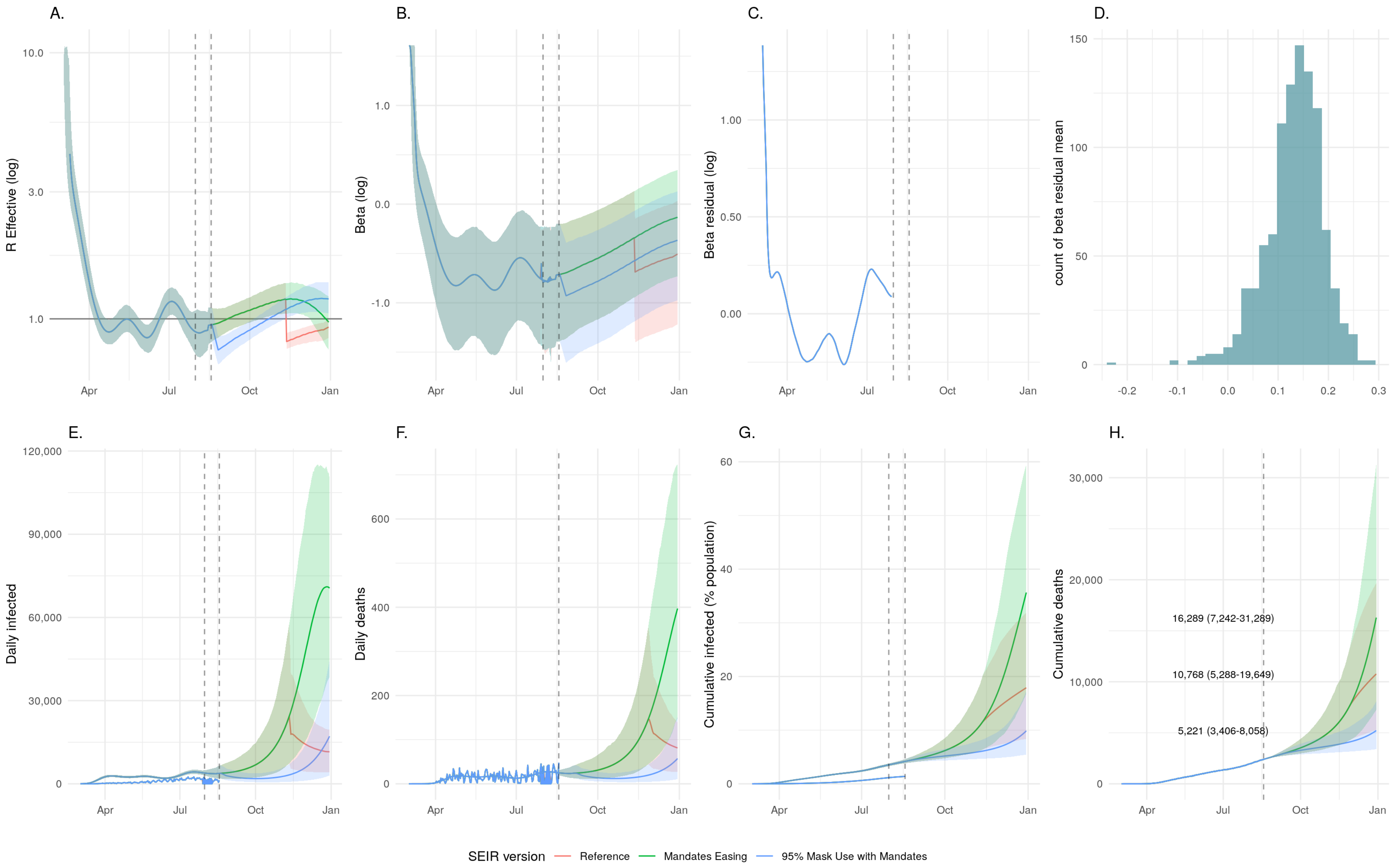

North Carolina: SEIR fit comparison. **A**: predicted R effective for each model through December 31. **B**: predicted SEIR *beta* parameter. **C**: residual of predicted *beta* and the observed value calculated directly from infection data over time. **D**: histogram of residual values for *beta*. Panels A, B, C, and D are all displayed in log space, reflecting the space in which the SEIR model is fit. **E**: predicted daily infections from each model through December 31. **F**: predicted daily deaths from each model through December 31. **G**: predicted cumulative infections through December 31, as a proportion of the total population. **H**: predicted cumulative deaths through December 31. In panels E, F, G, and H, reported death and infections are plotted alongside model predictions in light blue.

70 North Carolina: Covariate fits and regression coefficients

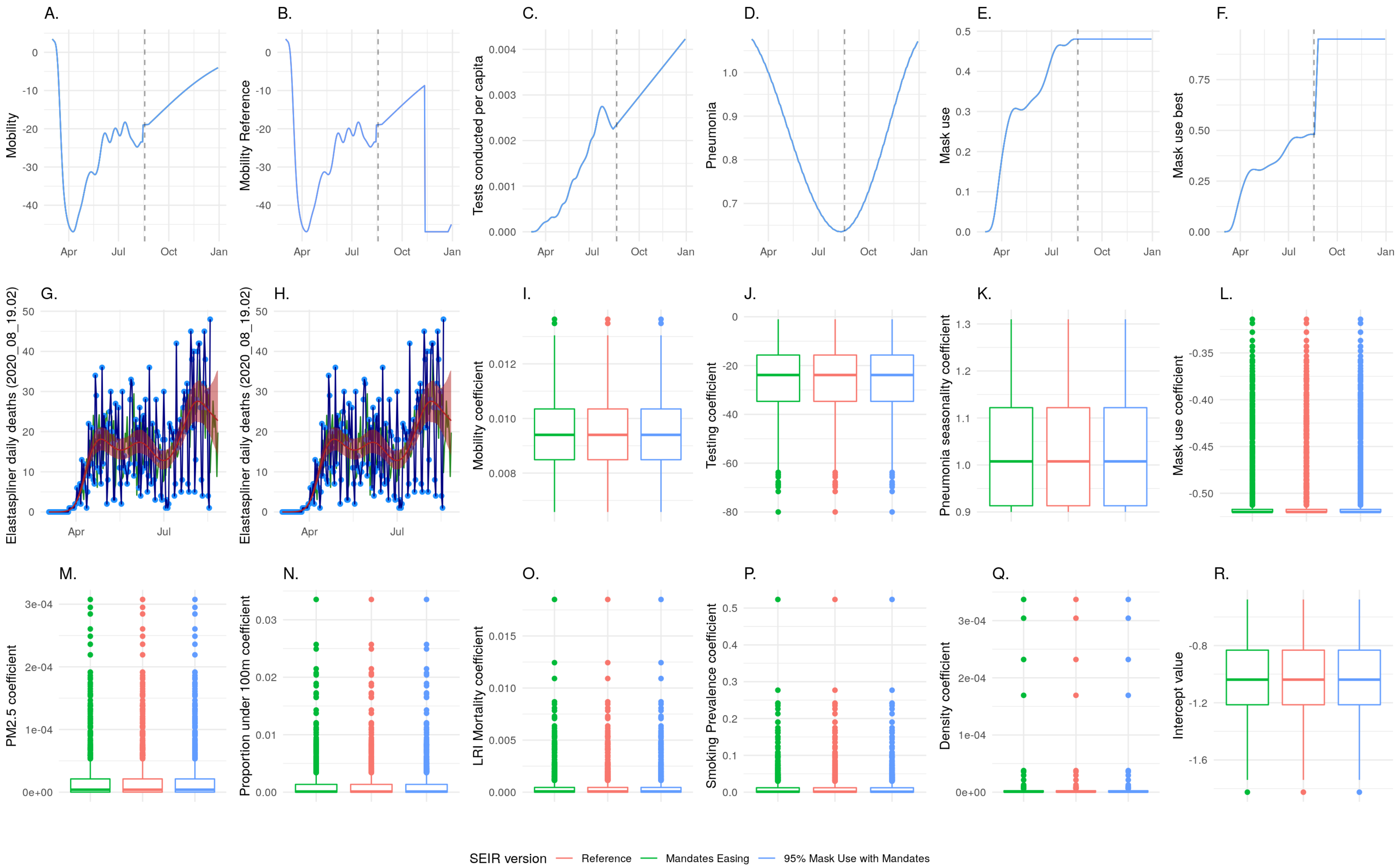

North Carolina: Covariate fits and regression coefficients. **A-F**: Line plots showing predicted covariate time trends for **A)** mobility in the absence of additional mandates; **B)** mobility with additional mandates applied; **C)** diagnostic testing per capita; **D)** pneumonia seasonality; **E)** mask use per capita, and; **F)** mask use in a scenario where adherence increases to 95% of the population. **G-H**: COVID mortality data generated from reported daily deaths (blue); estimated based on reported hospitalizations (purple); estimated from reported cases (green); and via a spline fit through all available data types (red, 95% UI in pink). **I-R**: Box plots showing 1,000 draws of fixed effect coefficients in a multivariate regression fit to $log(beta)$.

71 North Dakota: SEIR fit comparison

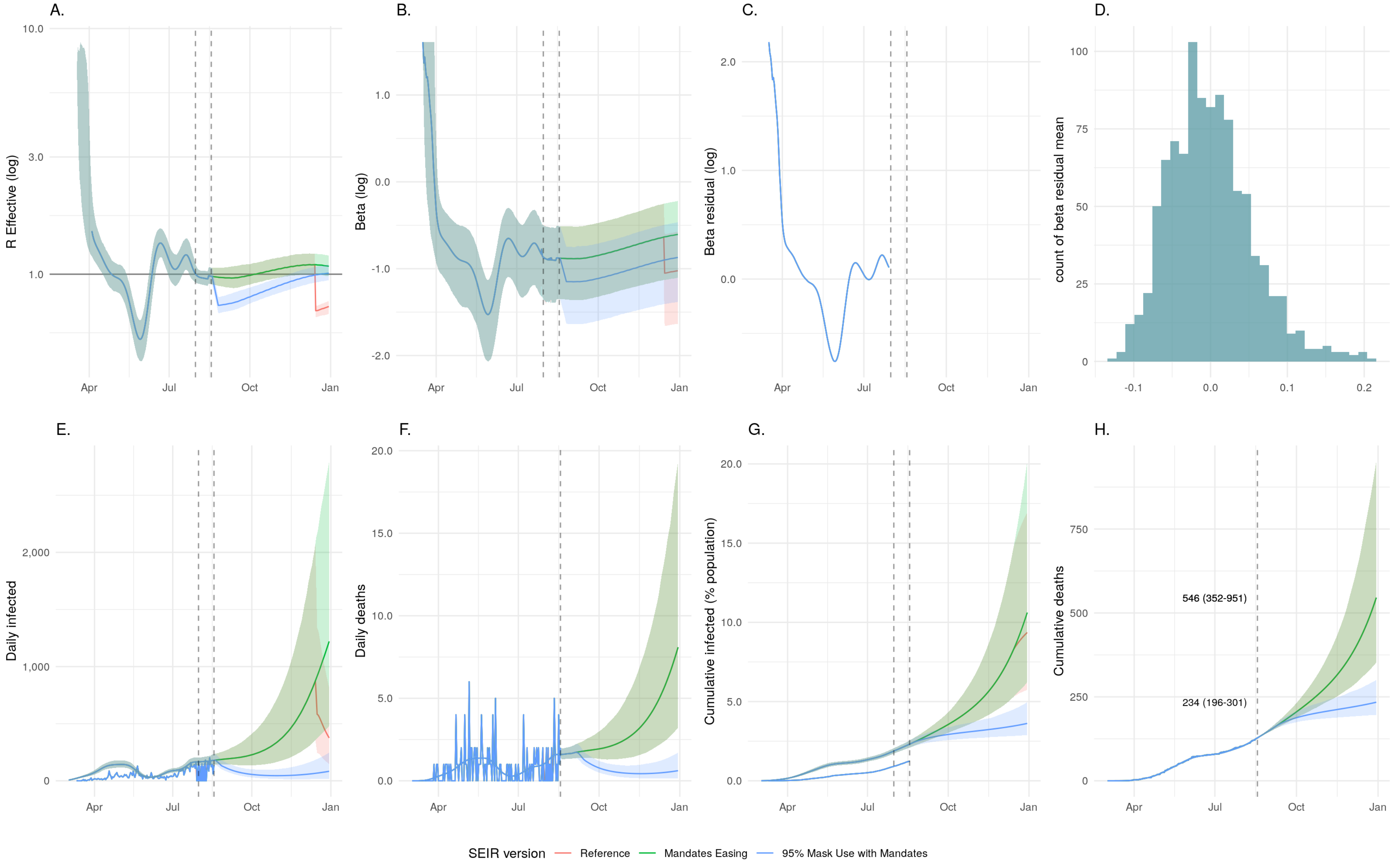

North Dakota: SEIR fit comparison. A: predicted R effective for each model through December 31. **B**: predicted SEIR *beta* parameter. **C**: residual of predicted *beta* and the observed value calculated directly from infection data over time. **D**: histogram of residual values for *beta*. Panels A, B, C, and D are all displayed in log space, reflecting the space in which the SEIR model is fit. **E**: predicted daily infections from each model through December 31. **F**: predicted daily deaths from each model through December 31. **G**: predicted cumulative infections through December 31, as a proportion of the total population. **H**: predicted cumulative deaths through December 31. In panels E, F, G, and H, reported death and infections are plotted alongside model predictions in light blue.

72 North Dakota: Covariate fits and regression coefficients

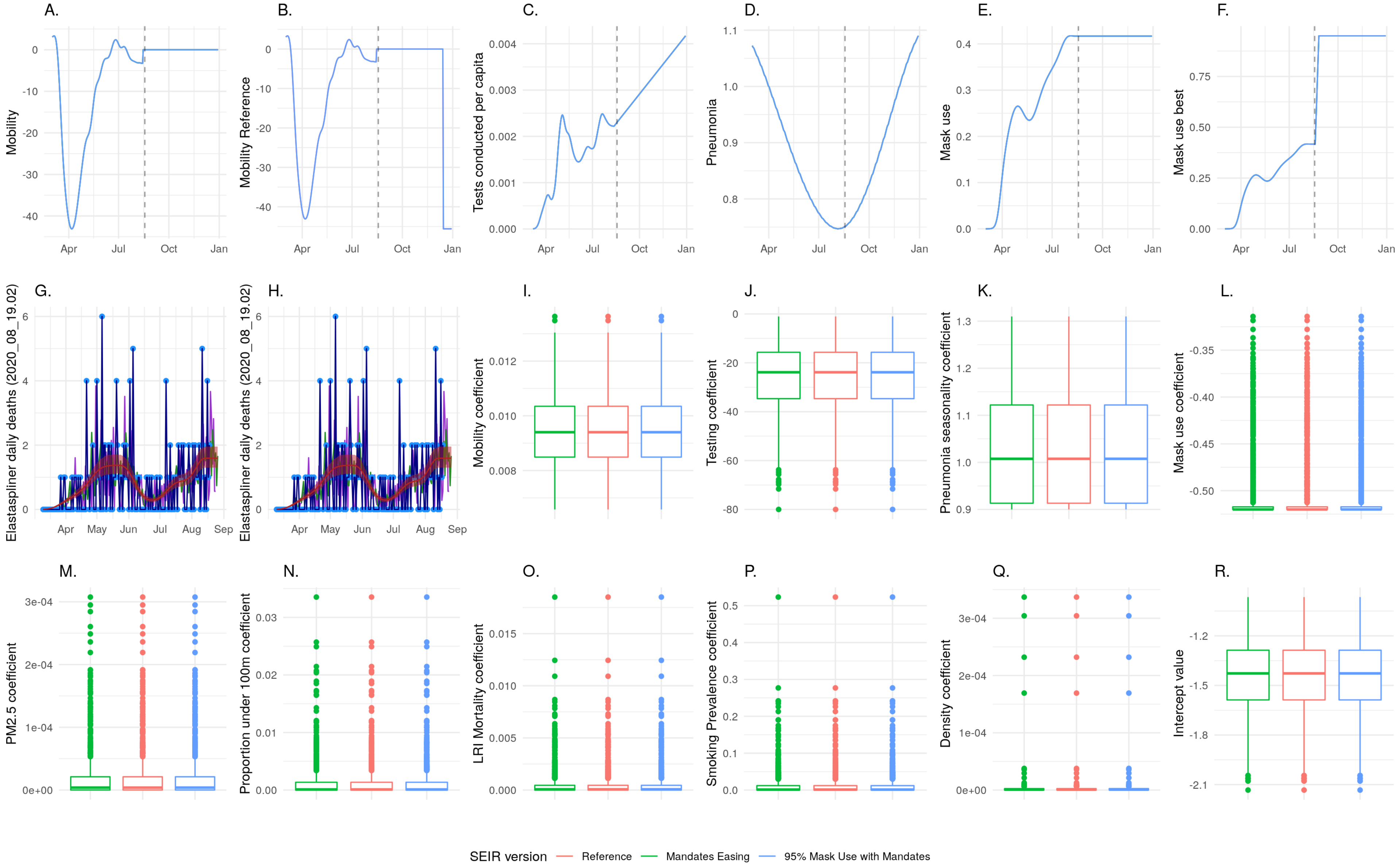

North Dakota: Covariate fits and regression coefficients. A-F: Line plots showing predicted covariate time trends for **A)** mobility in the absence of additional mandates; **B)** mobility with additional mandates applied; **C)** diagnostic testing per capita; **D)** pneumonia seasonality; **E)** mask use per capita, and; **F)** mask use in a scenario where adherence increases to 95% of the population. **G-H**: COVID mortality data generated from reported daily deaths (blue); estimated based on reported hospitalizations (purple); estimated from reported cases (green); and via a spline fit through all available data types (red, 95% UI in pink). **I-R**: Box plots showing 1,000 draws of fixed effect coefficients in a multivariate regression fit to $log(beta)$.

73 Ohio: SEIR fit comparison

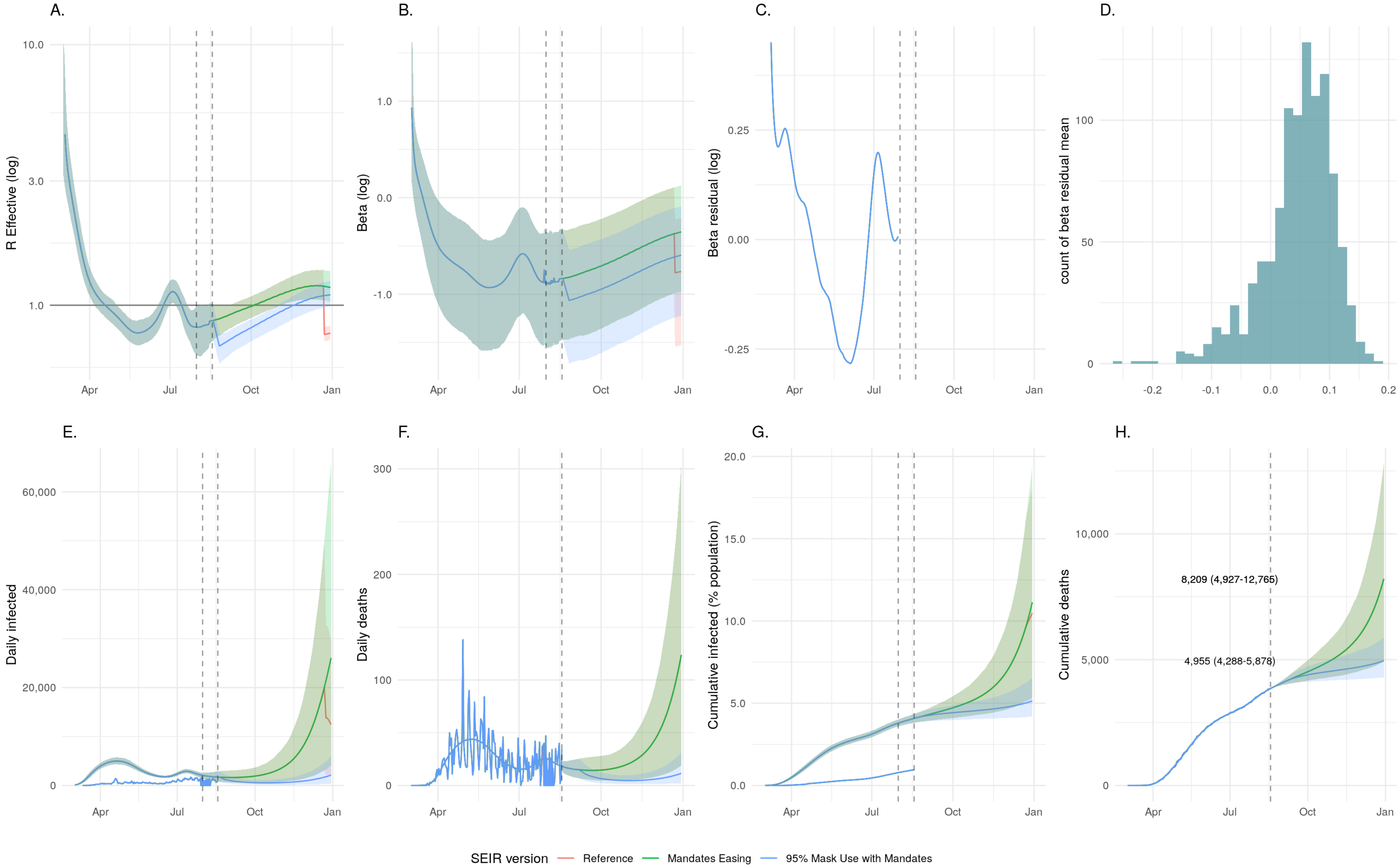

Ohio: SEIR fit comparison. **A**: predicted R effective for each model through December 31. **B**: predicted SEIR *beta* parameter. **C**: residual of predicted *beta* and the observed value calculated directly from infection data over time. **D**: histogram of residual values for *beta*. Panels A, B, C, and D are all displayed in log space, reflecting the space in which the SEIR model is fit. **E**: predicted daily infections from each model through December 31. **F**: predicted daily deaths from each model through December 31. **G**: predicted cumulative infections through December 31, as a proportion of the total population. **H**: predicted cumulative deaths through December 31. In panels E, F, G, and H, reported death and infections are plotted alongside model predictions in light blue.

74 Ohio: Covariate fits and regression coefficients

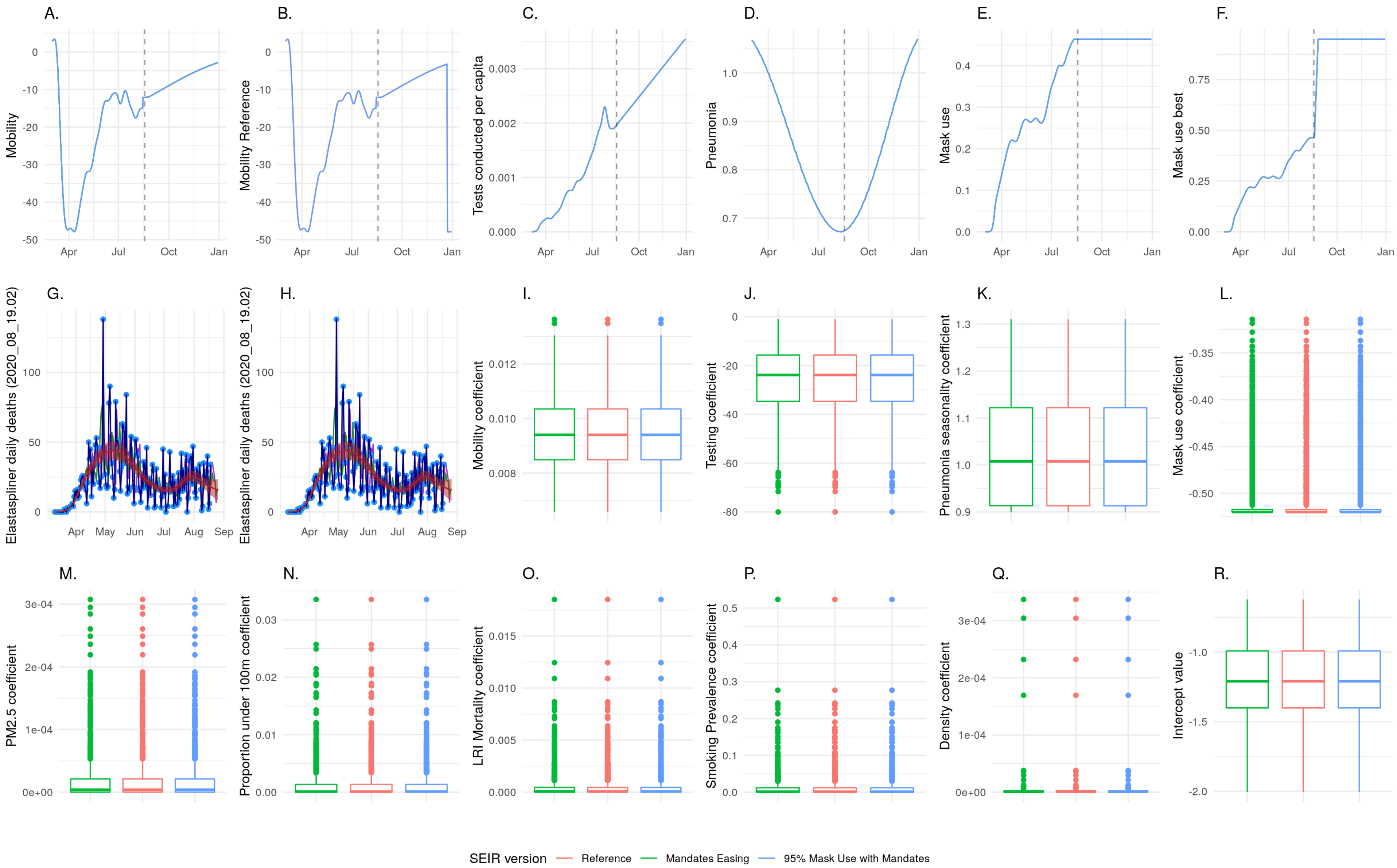

Ohio: Covariate fits and regression coefficients. **A-F**: Line plots showing predicted covariate time trends for **A)** mobility in the absence of additional mandates; **B)** mobility with additional mandates applied; **C)** diagnostic testing per capita; **D)** pneumonia seasonality; **E)** mask use per capita, and; **F)** mask use in a scenario where adherence increases to 95% of the population. **G-H**: COVID mortality data generated from reported daily deaths (blue); estimated based on reported hospitalizations (purple); estimated from reported cases (green); and via a spline fit through all available data types (red, 95% UI in pink). **I-R**: Box plots showing 1,000 draws of fixed effect coefficients in a multivariate regression fit to $log(beta)$.

75 Oklahoma: SEIR fit comparison

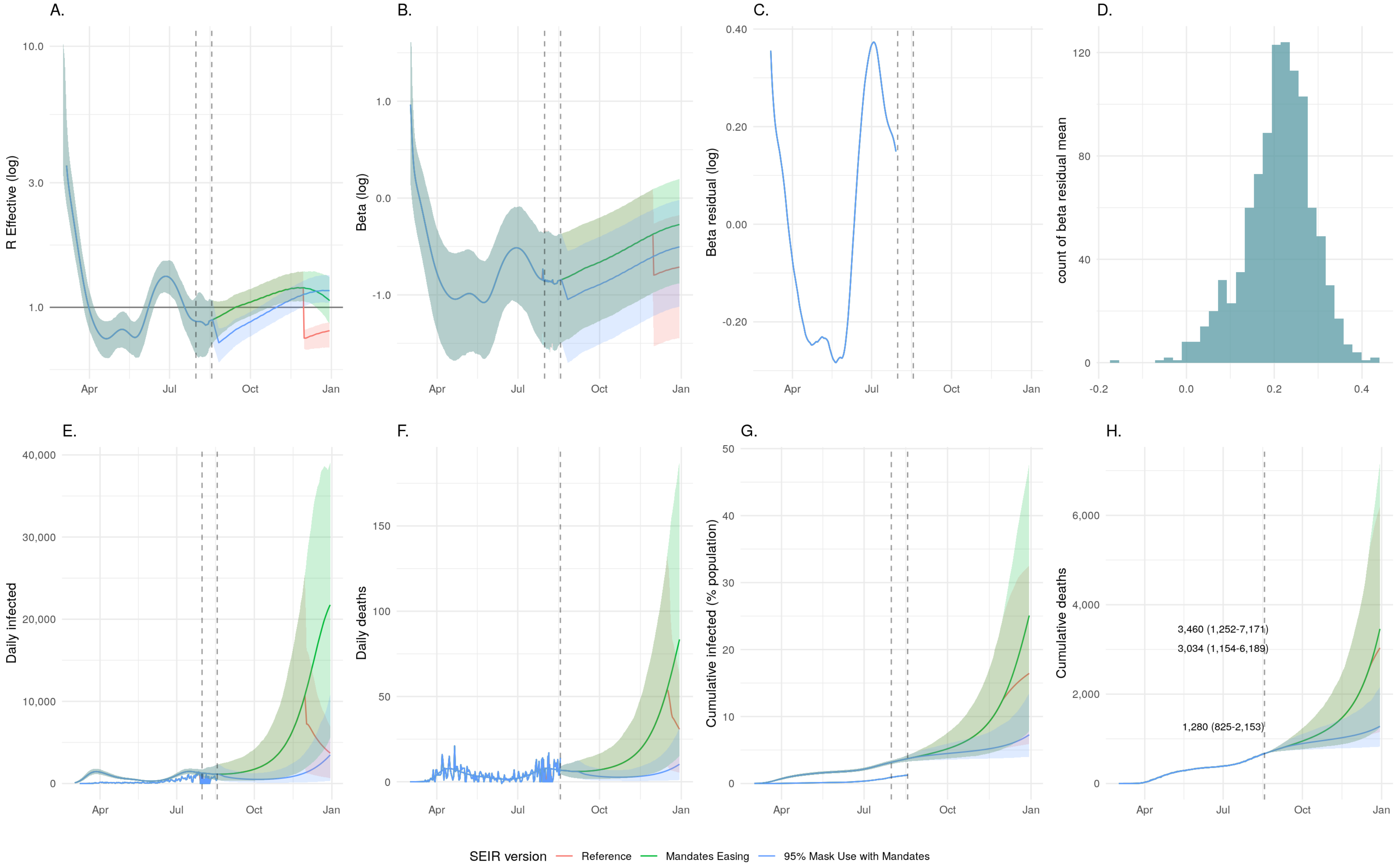

Oklahoma: SEIR fit comparison. **A**: predicted R effective for each model through December 31. **B**: predicted SEIR *beta* parameter. **C**: residual of predicted *beta* and the observed value calculated directly from infection data over time. **D**: histogram of residual values for *beta*. Panels A, B, C, and D are all displayed in log space, reflecting the space in which the SEIR model is fit. **E**: predicted daily infections from each model through December 31. **F**: predicted daily deaths from each model through December 31. **G**: predicted cumulative infections through December 31, as a proportion of the total population. **H**: predicted cumulative deaths through December 31. In panels E, F, G, and H, reported death and infections are plotted alongside model predictions in light blue.

76 Oklahoma: Covariate fits and regression coefficients

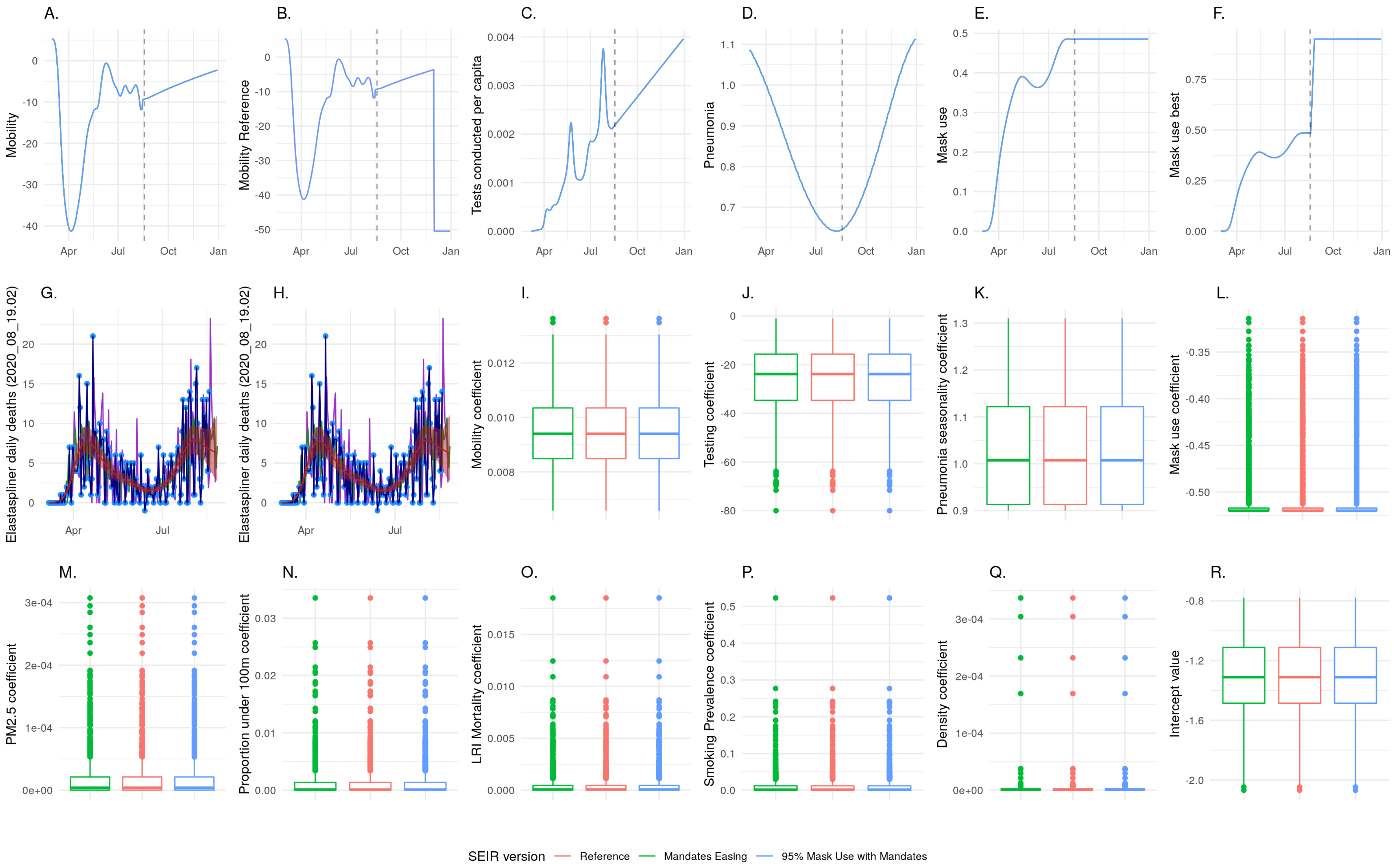

Oklahoma: Covariate fits and regression coefficients. A-F: Line plots showing predicted covariate time trends for **A)** mobility in the absence of additional mandates; **B)** mobility with additional mandates applied; **C)** diagnostic testing per capita; **D)** pneumonia seasonality; **E)** mask use per capita, and; **F)** mask use in a scenario where adherence increases to 95% of the population. **G-H**: COVID mortality data generated from reported daily deaths (blue); estimated based on reported hospitalizations (purple); estimated from reported cases (green); and via a spline fit through all available data types (red, 95% UI in pink). **I-R**: Box plots showing 1,000 draws of fixed effect coefficients in a multivariate regression fit to $log(beta)$.

77 Oregon: SEIR fit comparison

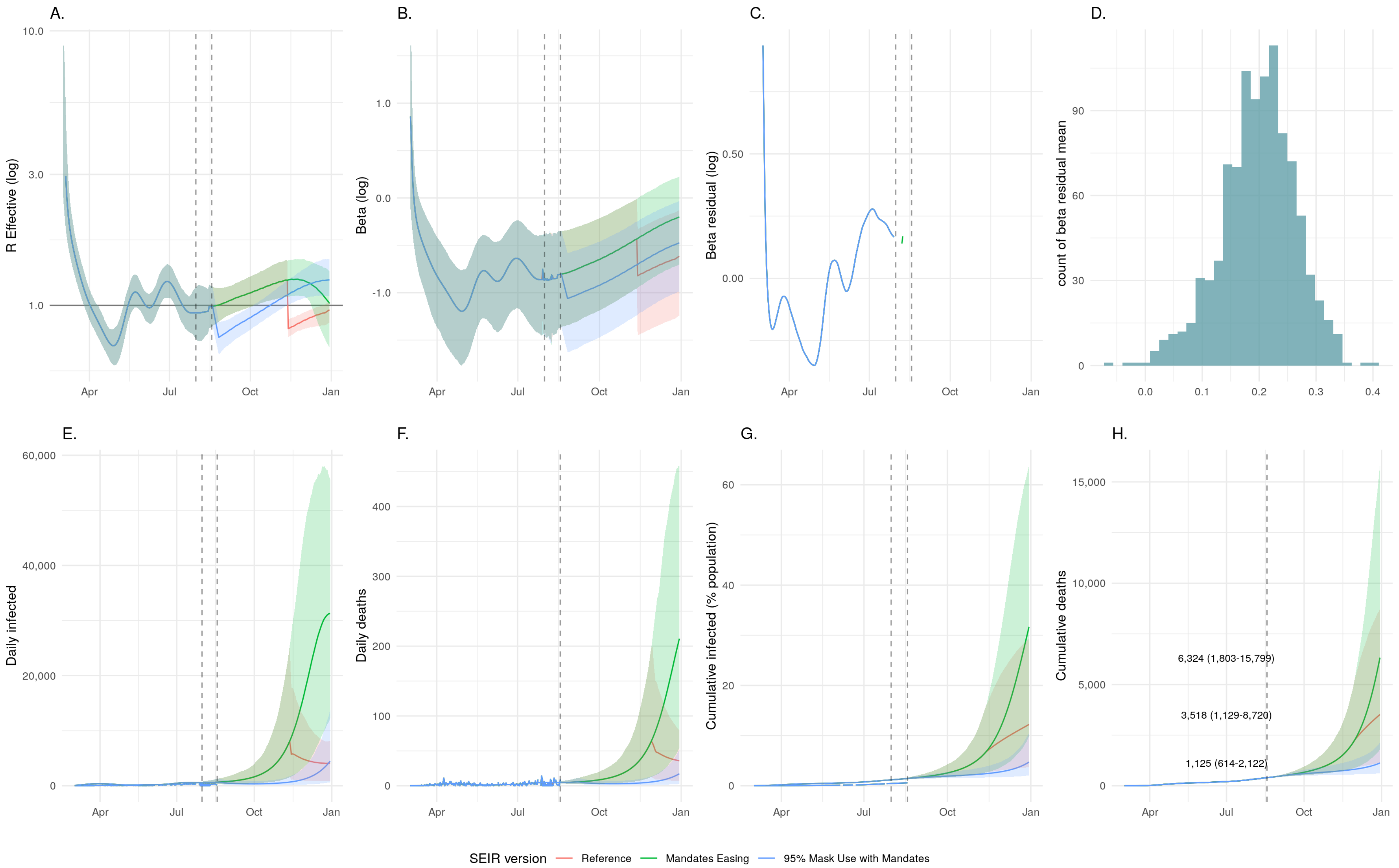

Oregon: SEIR fit comparison. **A**: predicted R effective for each model through December 31. **B**: predicted SEIR *beta* parameter. **C**: residual of predicted *beta* and the observed value calculated directly from infection data over time. **D**: histogram of residual values for *beta*. Panels A, B, C, and D are all displayed in log space, reflecting the space in which the SEIR model is fit. **E**: predicted daily infections from each model through December 31. **F**: predicted daily deaths from each model through December 31. **G**: predicted cumulative infections through December 31, as a proportion of the total population. **H**: predicted cumulative deaths through December 31. In panels E, F, G, and H, reported death and infections are plotted alongside model predictions in light blue.

78 Oregon: Covariate fits and regression coefficients

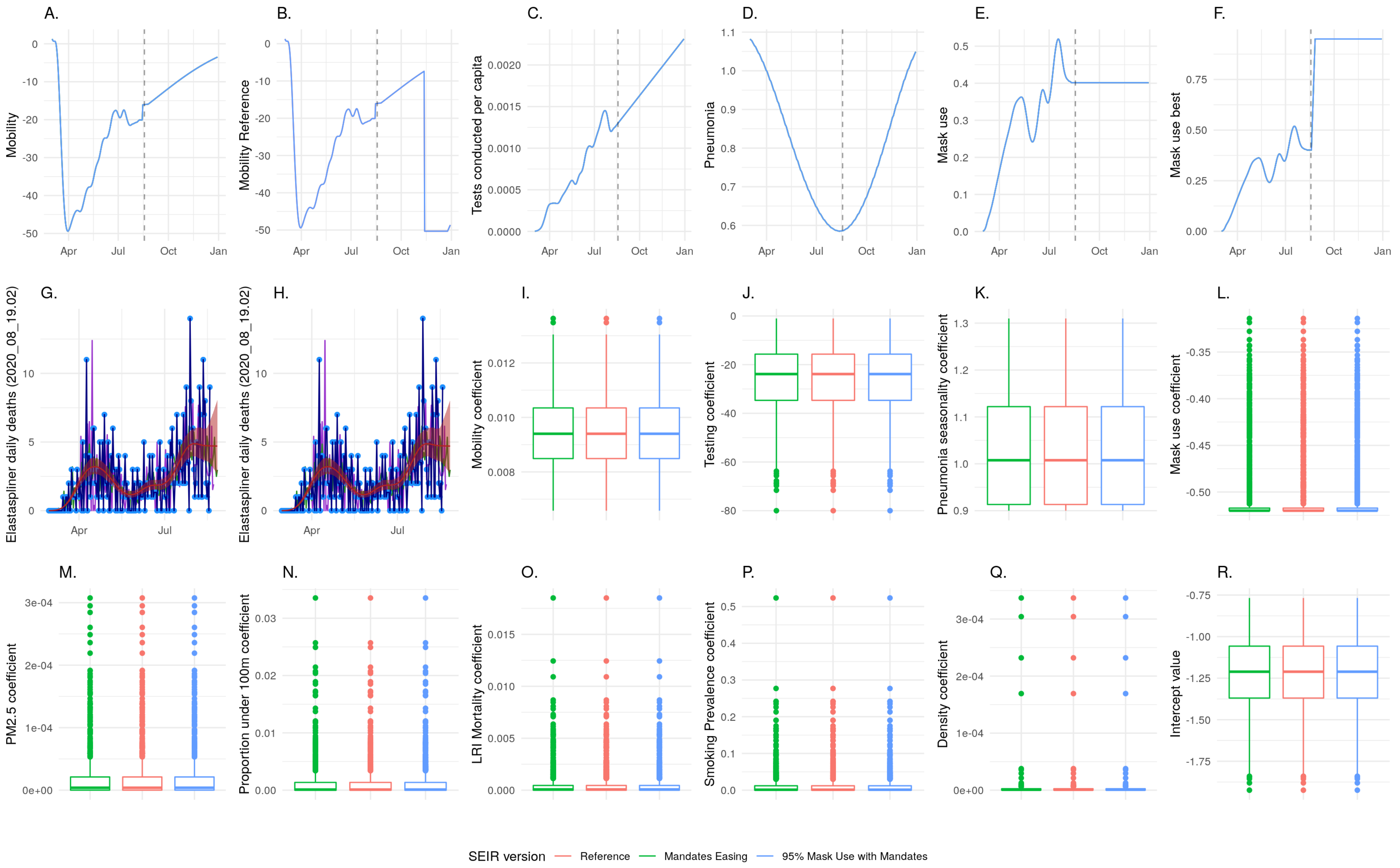

Oregon: Covariate fits and regression coefficients. **A-F**: Line plots showing predicted covariate time trends for **A)** mobility in the absence of additional mandates; **B)** mobility with additional mandates applied; **C)** diagnostic testing per capita; **D)** pneumonia seasonality; **E)** mask use per capita, and; **F)** mask use in a scenario where adherence increases to 95% of the population. **G-H**: COVID mortality data generated from reported daily deaths (blue); estimated based on reported hospitalizations (purple); estimated from reported cases (green); and via a spline fit through all available data types (red, 95% UI in pink). **I-R**: Box plots showing 1,000 draws of fixed effect coefficients in a multivariate regression fit to $log(beta)$.

79 Pennsylvania: SEIR fit comparison

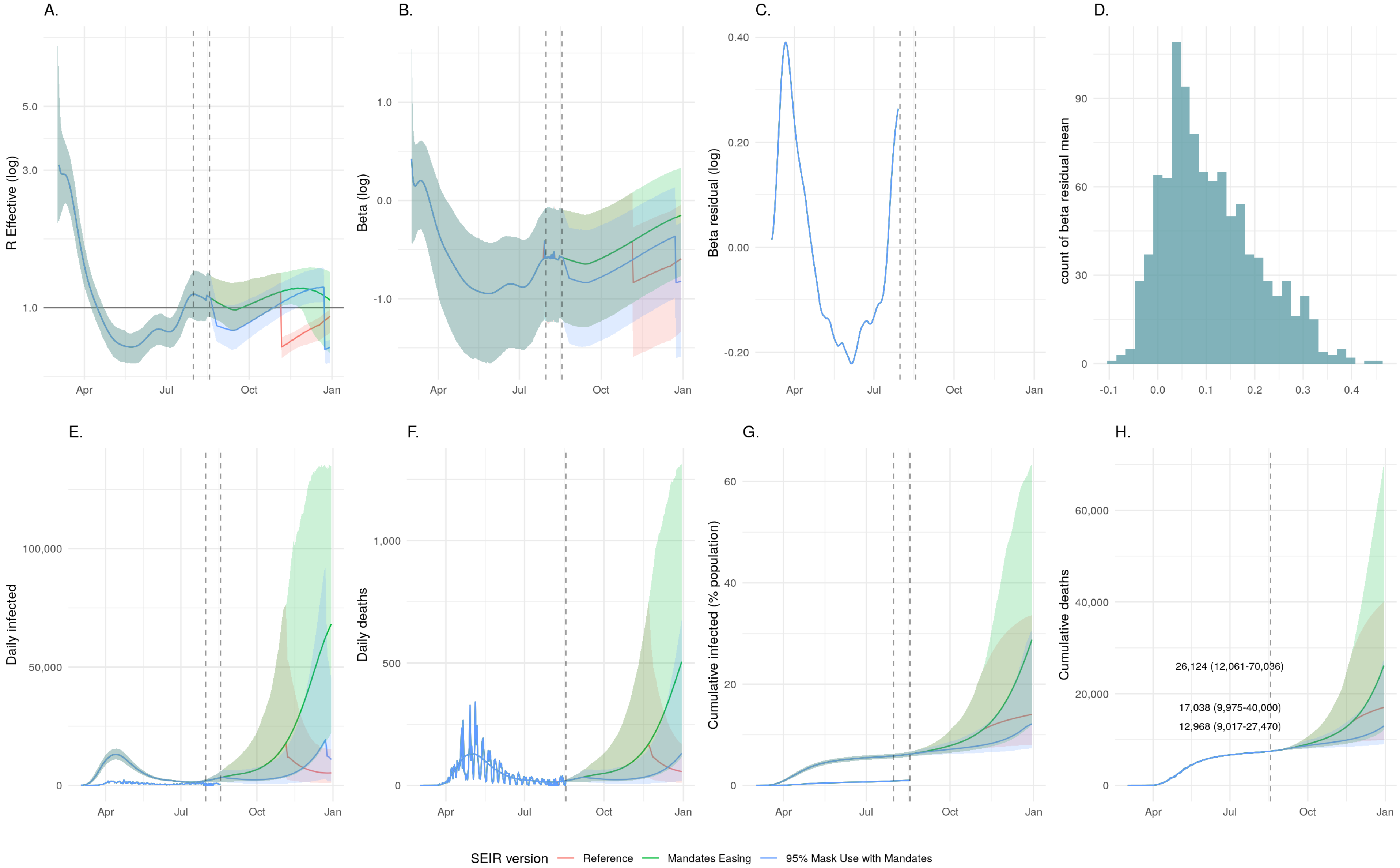

Pennsylvania: SEIR fit comparison. **A**: predicted R effective for each model through December 31. **B**: predicted SEIR *beta* parameter. **C**: residual of predicted *beta* and the observed value calculated directly from infection data over time. **D**: histogram of residual values for *beta*. Panels A, B, C, and D are all displayed in log space, reflecting the space in which the SEIR model is fit. **E**: predicted daily infections from each model through December 31. **F**: predicted daily deaths from each model through December 31. **G**: predicted cumulative infections through December 31, as a proportion of the total population. **H**: predicted cumulative deaths through December 31. In panels E, F, G, and H, reported death and infections are plotted alongside model predictions in light blue.

80 Pennsylvania: Covariate fits and regression coefficients

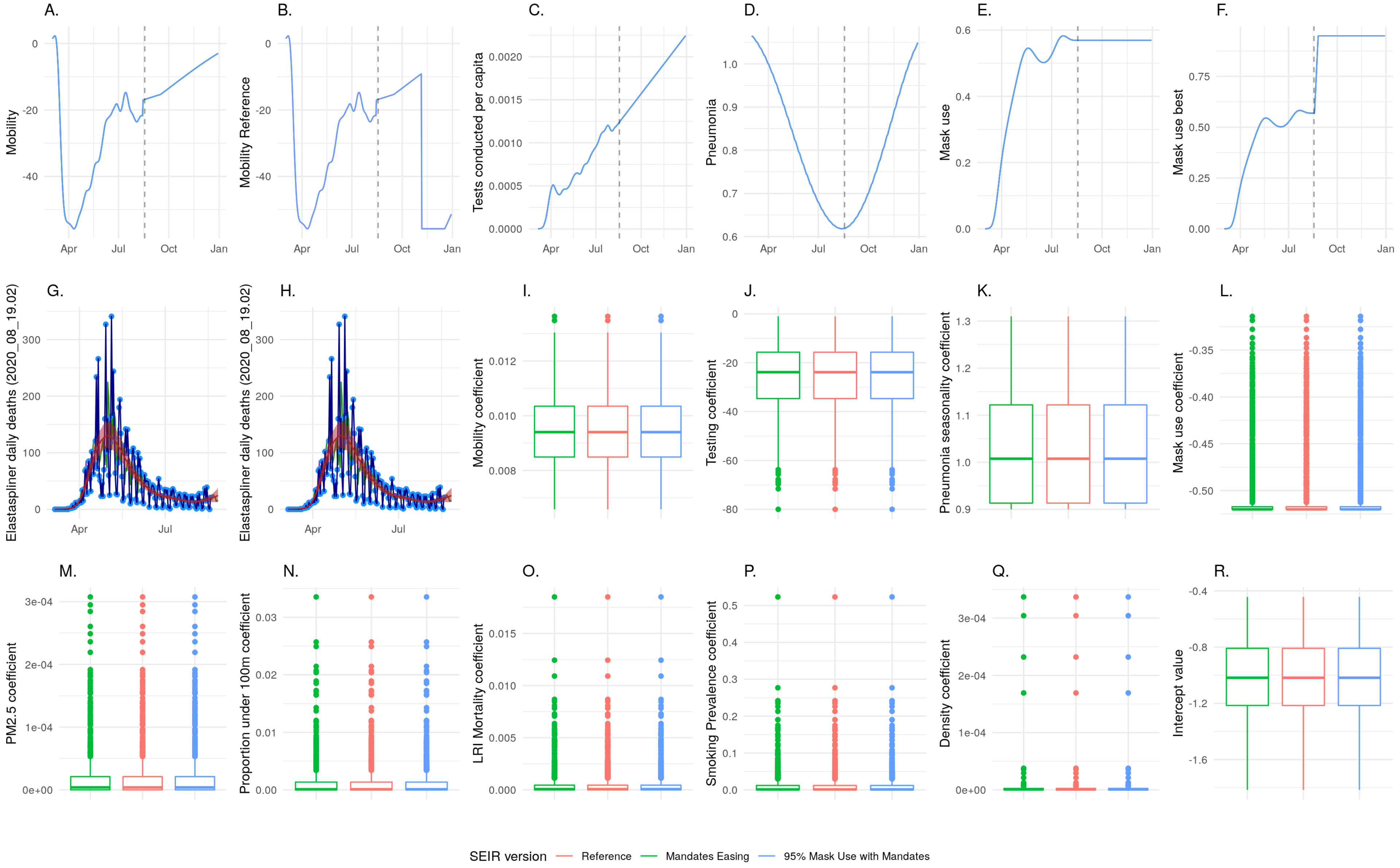

Pennsylvania: Covariate fits and regression coefficients. A-F: Line plots showing predicted covariate time trends for **A)** mobility in the absence of additional mandates; **B)** mobility with additional mandates applied; **C)** diagnostic testing per capita; **D)** pneumonia seasonality; **E)** mask use per capita, and; **F)** mask use in a scenario where adherence increases to 95% of the population. **G-H**: COVID mortality data generated from reported daily deaths (blue); estimated based on reported hospitalizations (purple); estimated from reported cases (green); and via a spline fit through all available data types (red, 95% UI in pink). **I-R**: Box plots showing 1,000 draws of fixed effect coefficients in a multivariate regression fit to $log(beta)$.

81 Rhode Island: SEIR fit comparison

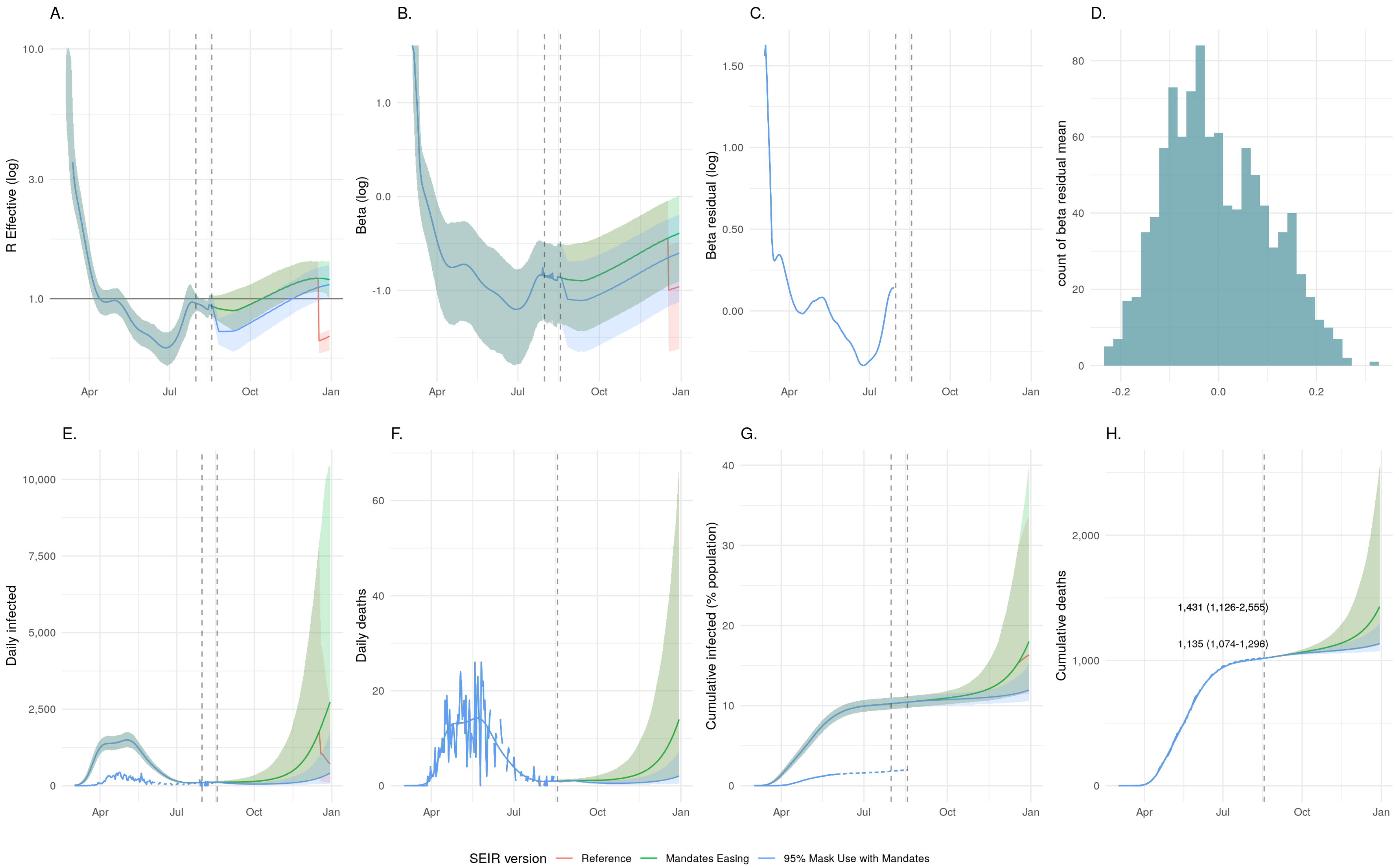

Rhode Island: SEIR fit comparison. A: predicted R effective for each model through December 31. **B**: predicted SEIR *beta* parameter. **C**: residual of predicted *beta* and the observed value calculated directly from infection data over time. **D**: histogram of residual values for *beta*. Panels A, B, C, and D are all displayed in log space, reflecting the space in which the SEIR model is fit. **E**: predicted daily infections from each model through December 31. **F**: predicted daily deaths from each model through December 31. **G**: predicted cumulative infections through December 31, as a proportion of the total population. **H**: predicted cumulative deaths through December 31. In panels E, F, G, and H, reported death and infections are plotted alongside model predictions in light blue.

82 Rhode Island: Covariate fits and regression coefficients

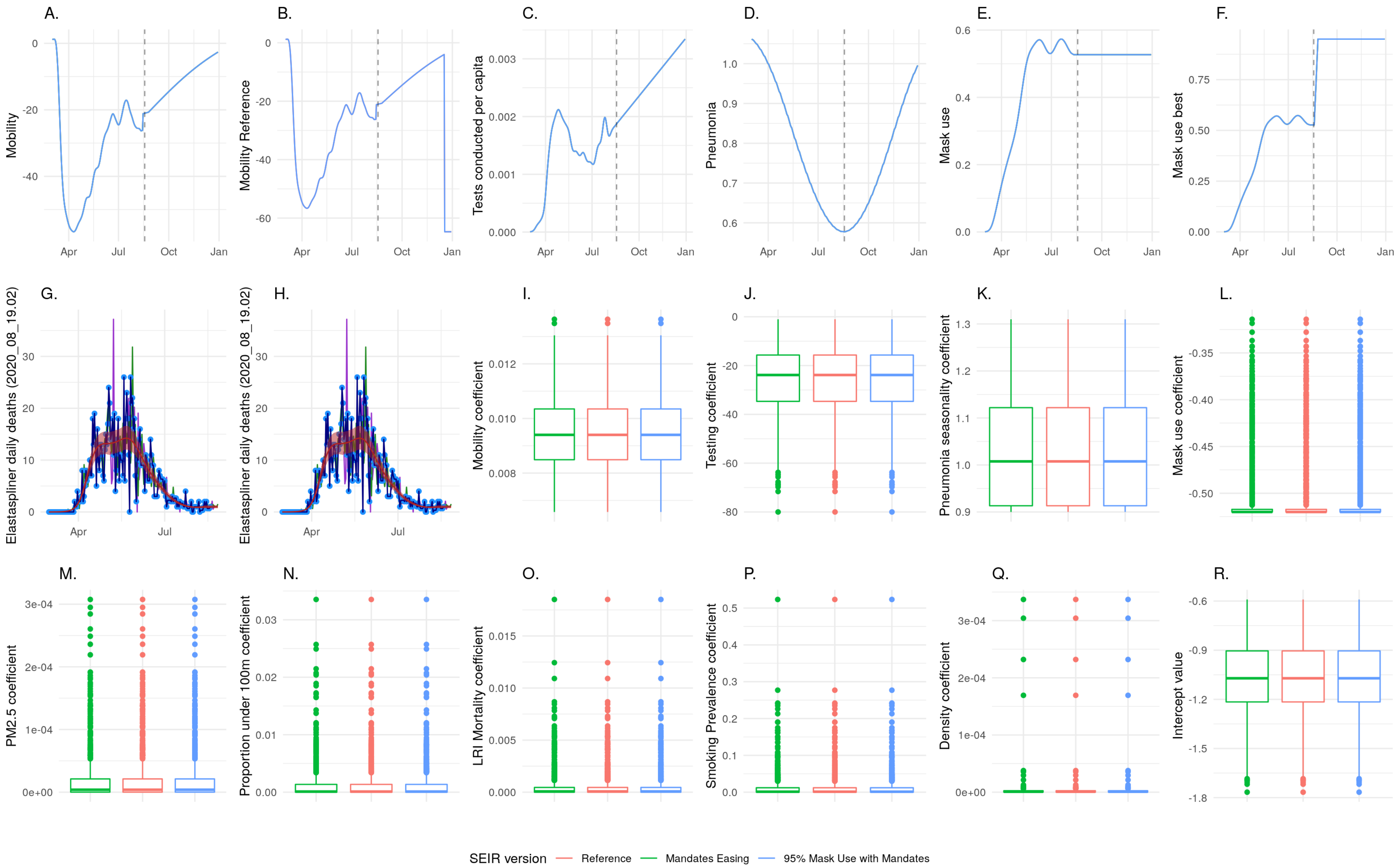

Rhode Island: Covariate fits and regression coefficients. A-F: Line plots showing predicted covariate time trends for **A)** mobility in the absence of additional mandates; **B)** mobility with additional mandates applied; **C)** diagnostic testing per capita; **D)** pneumonia seasonality; **E)** mask use per capita, and; **F)** mask use in a scenario where adherence increases to 95% of the population. **G-H**: COVID mortality data generated from reported daily deaths (blue); estimated based on reported hospitalizations (purple); estimated from reported cases (green); and via a spline fit through all available data types (red, 95% UI in pink). **I-R**: Box plots showing 1,000 draws of fixed effect coefficients in a multivariate regression fit to $log(beta)$.

83 South Carolina: SEIR fit comparison

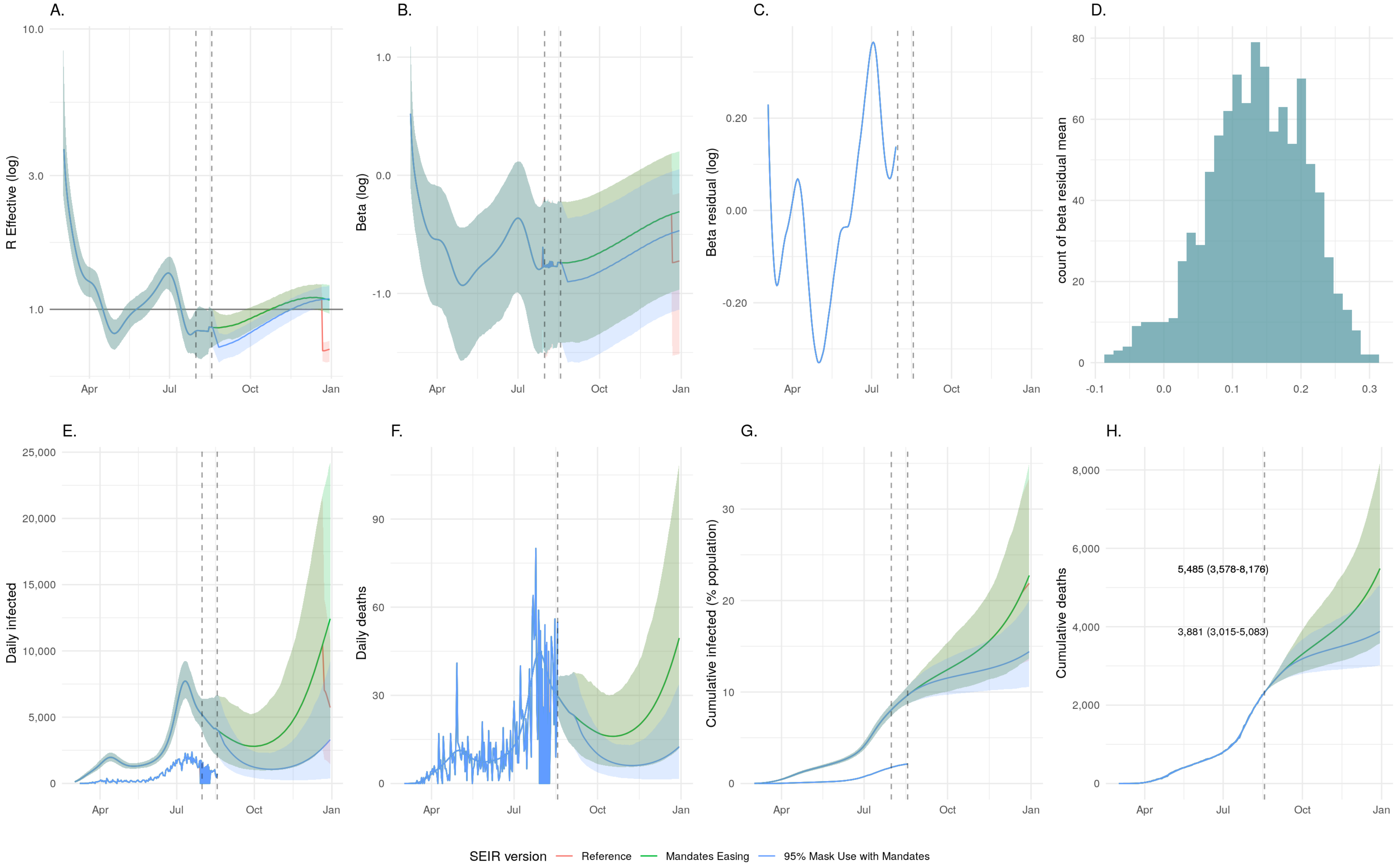

South Carolina: SEIR fit comparison. A: predicted R effective for each model through December 31. **B**: predicted SEIR *beta* parameter. **C**: residual of predicted *beta* and the observed value calculated directly from infection data over time. **D**: histogram of residual values for *beta*. Panels A, B, C, and D are all displayed in log space, reflecting the space in which the SEIR model is fit. **E**: predicted daily infections from each model through December 31. **F**: predicted daily deaths from each model through December 31. **G**: predicted cumulative infections through December 31, as a proportion of the total population. **H**: predicted cumulative deaths through December 31. In panels E, F, G, and H, reported death and infections are plotted alongside model predictions in light blue.

84 South Carolina: Covariate fits and regression coefficients

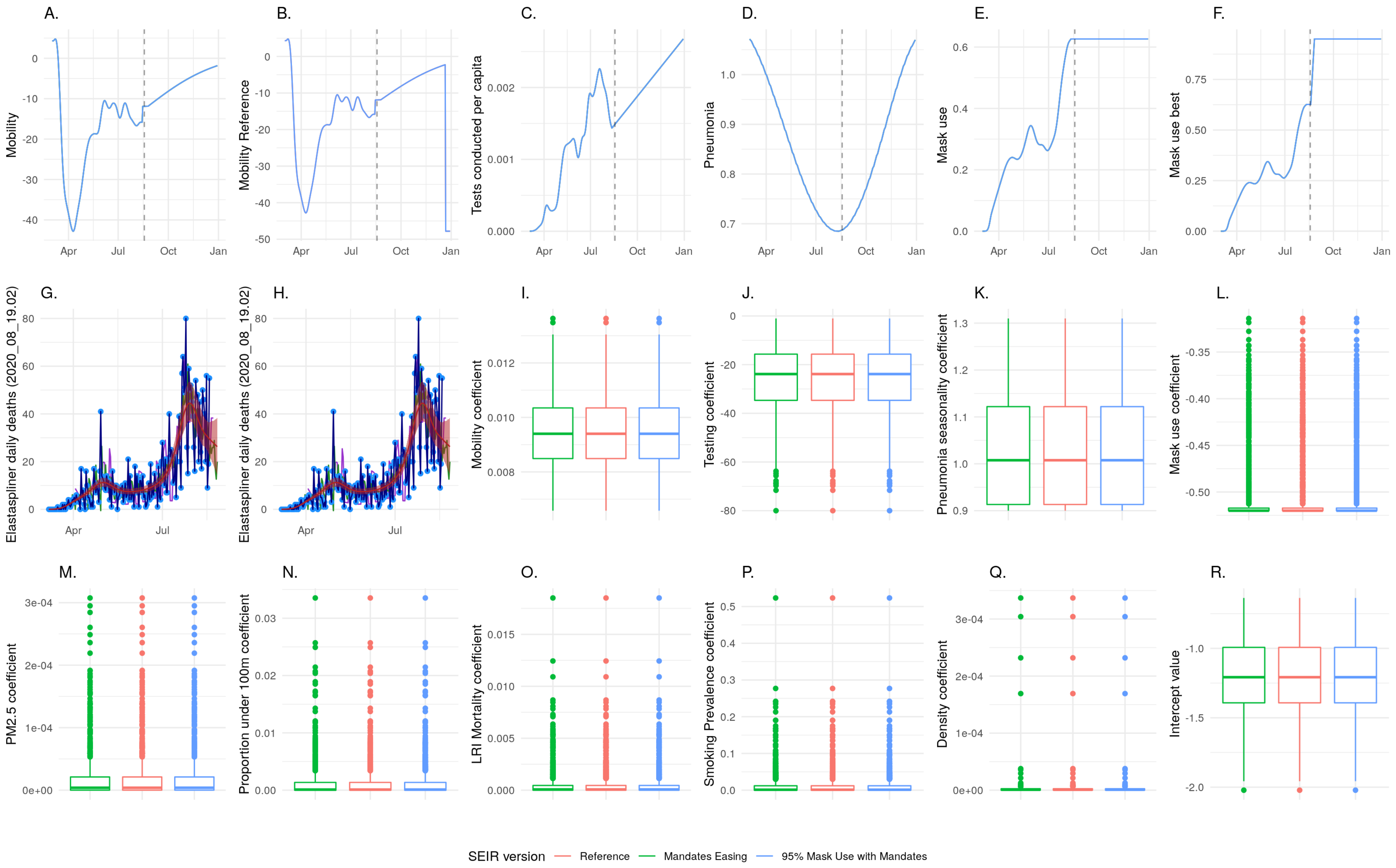

South Carolina: Covariate fits and regression coefficients. A-F: Line plots showing predicted covariate time trends for **A)** mobility in the absence of additional mandates; **B)** mobility with additional mandates applied; **C)** diagnostic testing per capita; **D)** pneumonia seasonality; **E)** mask use per capita, and; **F)** mask use in a scenario where adherence increases to 95% of the population. **G-H**: COVID mortality data generated from reported daily deaths (blue); estimated based on reported hospitalizations (purple); estimated from reported cases (green); and via a spline fit through all available data types (red, 95% UI in pink). **I-R**: Box plots showing 1,000 draws of fixed effect coefficients in a multivariate regression fit to $log(beta)$.

85 South Dakota: SEIR fit comparison

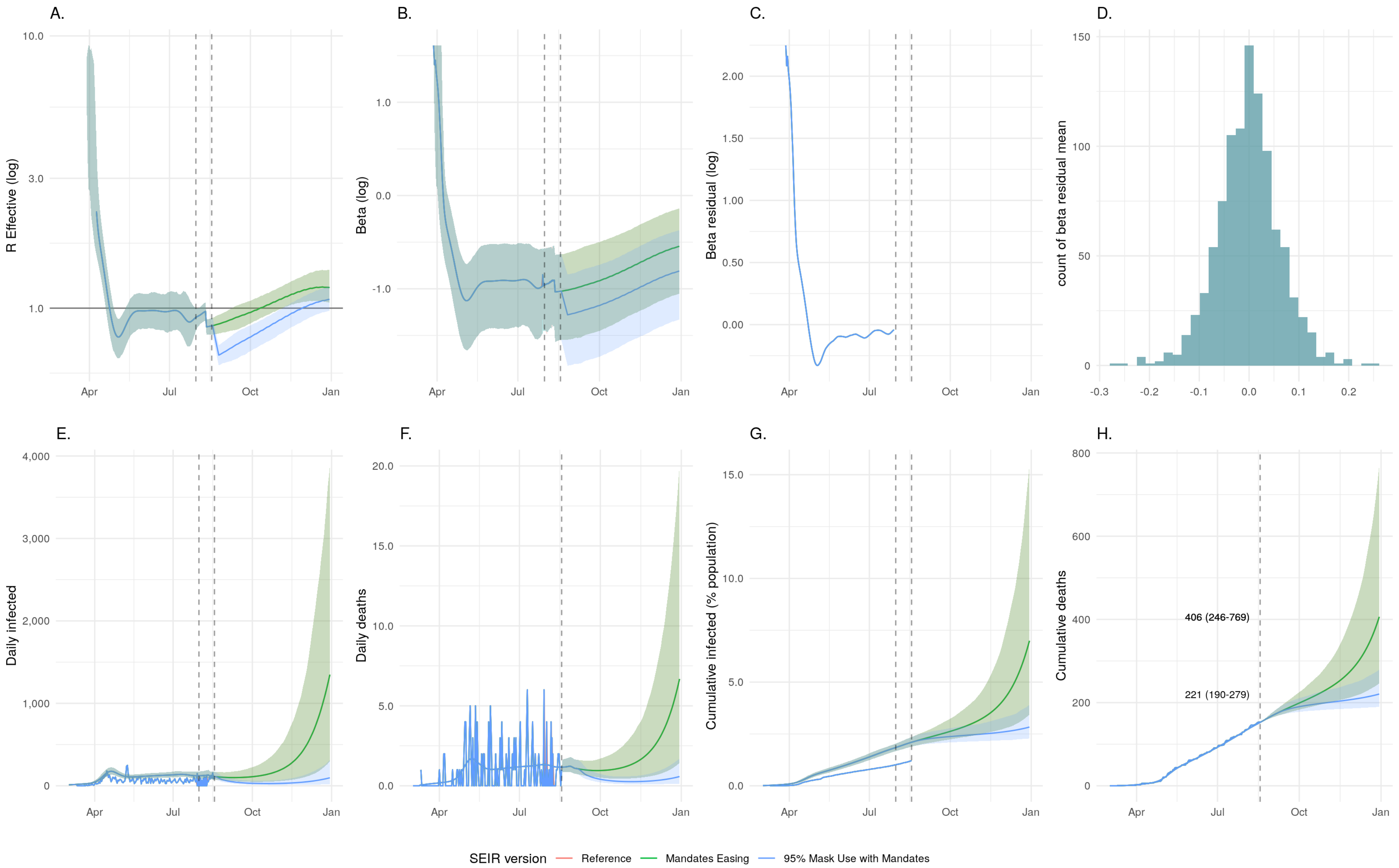

South Dakota: SEIR fit comparison. A: predicted R effective for each model through December 31. **B**: predicted SEIR *beta* parameter. **C**: residual of predicted *beta* and the observed value calculated directly from infection data over time. **D**: histogram of residual values for *beta*. Panels A, B, C, and D are all displayed in log space, reflecting the space in which the SEIR model is fit. **E**: predicted daily infections from each model through December 31. **F**: predicted daily deaths from each model through December 31. **G**: predicted cumulative infections through December 31, as a proportion of the total population. **H**: predicted cumulative deaths through December 31. In panels E, F, G, and H, reported death and infections are plotted alongside model predictions in light blue.

86 South Dakota: Covariate fits and regression coefficients

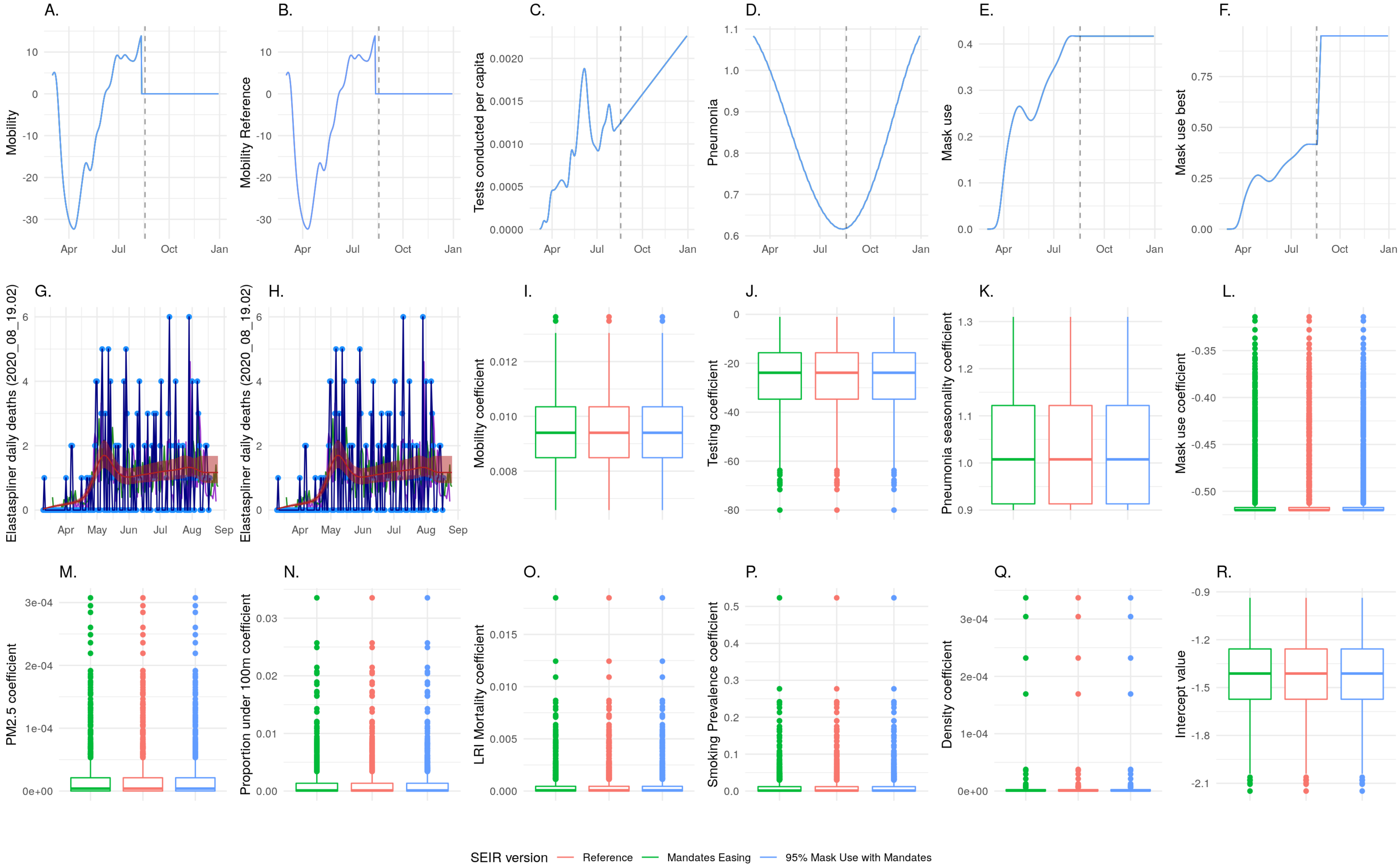

South Dakota: Covariate fits and regression coefficients. A-F: Line plots showing predicted covariate time trends for **A)** mobility in the absence of additional mandates; **B)** mobility with additional mandates applied; **C)** diagnostic testing per capita; **D)** pneumonia seasonality; **E)** mask use per capita, and; **F)** mask use in a scenario where adherence increases to 95% of the population. **G-H**: COVID mortality data generated from reported daily deaths (blue); estimated based on reported hospitalizations (purple); estimated from reported cases (green); and via a spline fit through all available data types (red, 95% UI in pink). **I-R**: Box plots showing 1,000 draws of fixed effect coefficients in a multivariate regression fit to $log(beta)$.

87 Tennessee: SEIR fit comparison

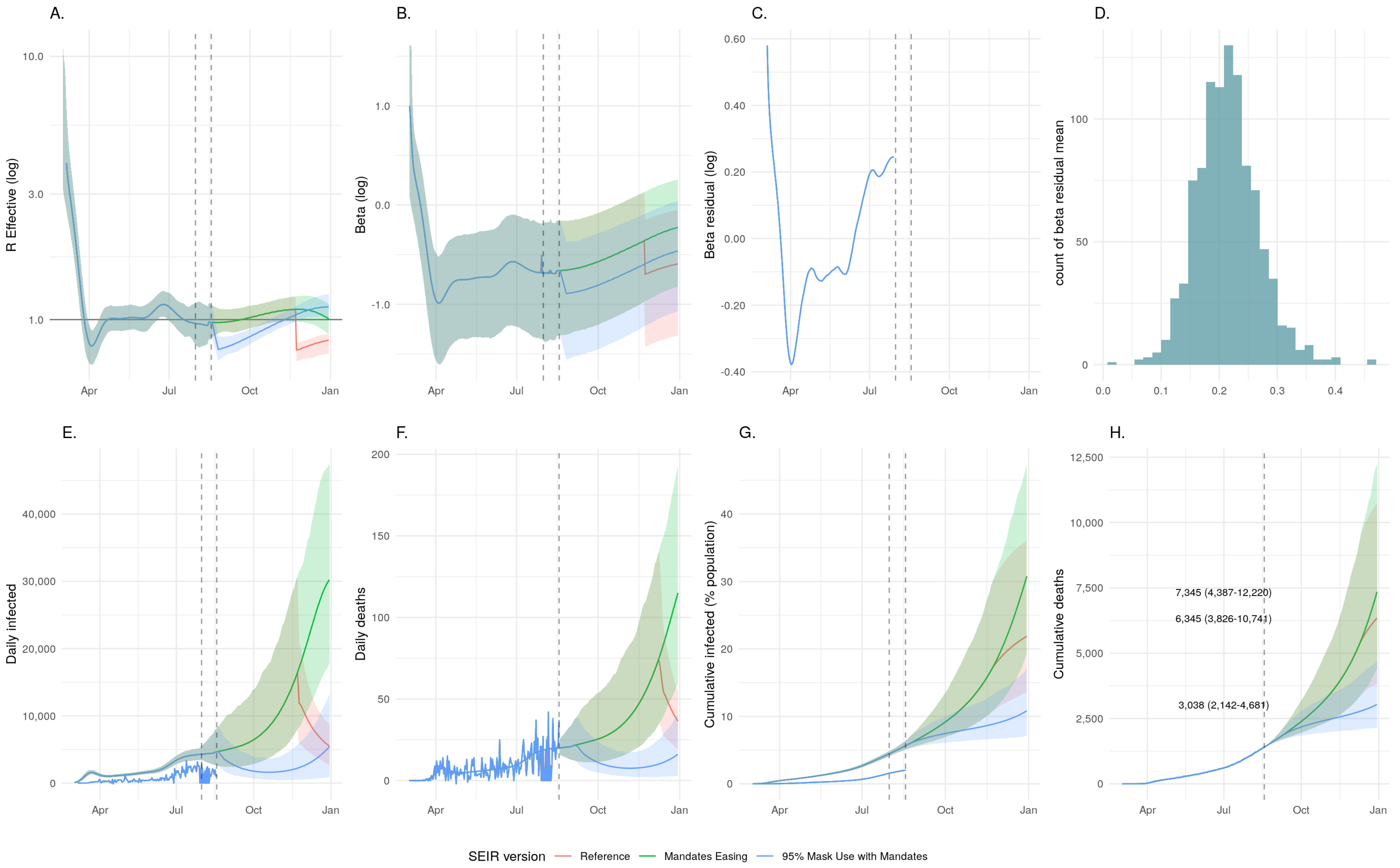

Tennessee: SEIR fit comparison. A: predicted R effective for each model through December 31. **B**: predicted SEIR *beta* parameter. **C**: residual of predicted *beta* and the observed value calculated directly from infection data over time. **D**: histogram of residual values for *beta*. Panels A, B, C, and D are all displayed in log space, reflecting the space in which the SEIR model is fit. **E**: predicted daily infections from each model through December 31. **F**: predicted daily deaths from each model through December 31. **G**: predicted cumulative infections through December 31, as a proportion of the total population. **H**: predicted cumulative deaths through December 31. In panels E, F, G, and H, reported death and infections are plotted alongside model predictions in light blue.

88 Tennessee: Covariate fits and regression coefficients

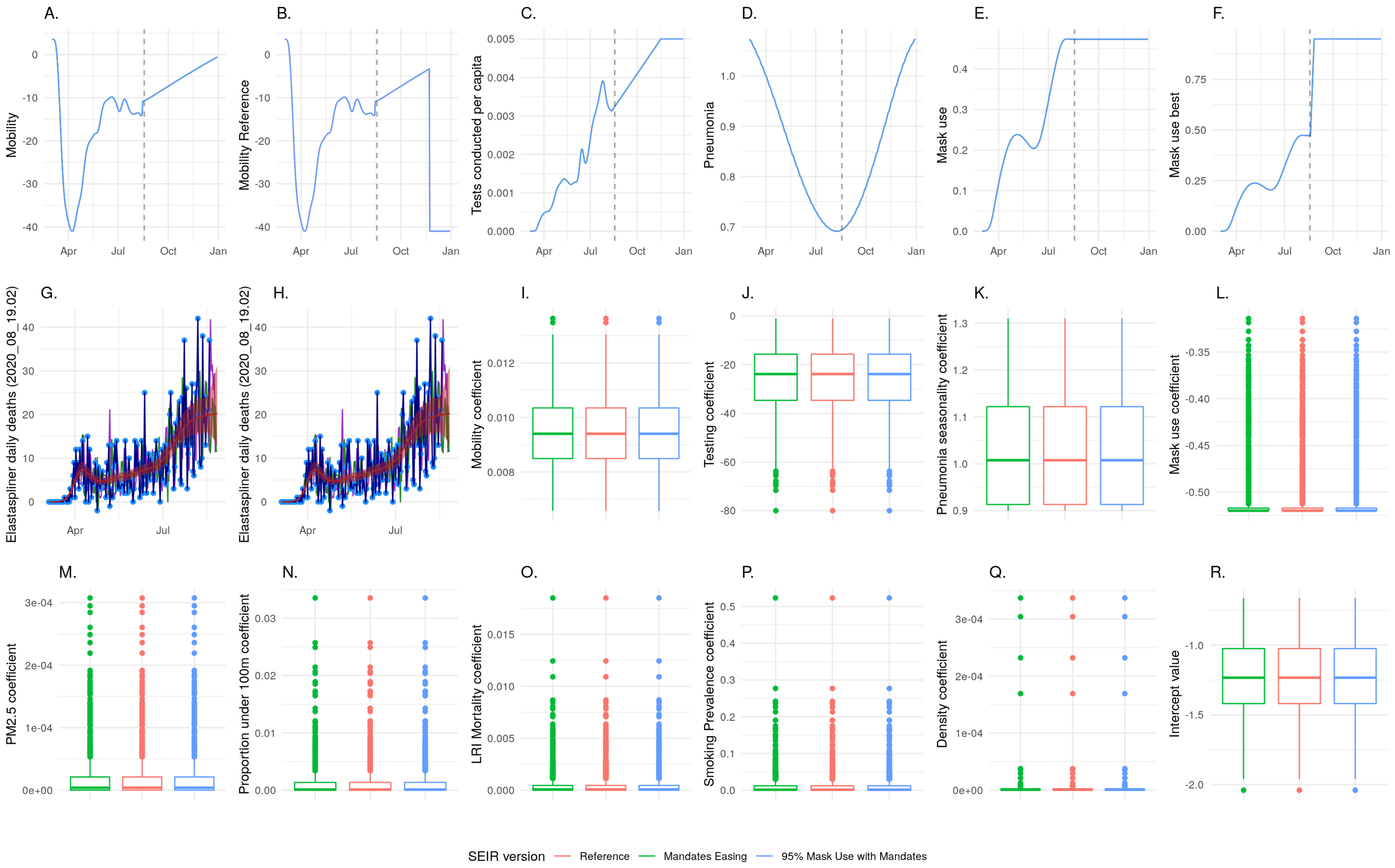

Tennessee: Covariate fits and regression coefficients. **A-F**: Line plots showing predicted covariate time trends for **A)** mobility in the absence of additional mandates; **B)** mobility with additional mandates applied; **C)** diagnostic testing per capita; **D)** pneumonia seasonality; **E)** mask use per capita, and; **F)** mask use in a scenario where adherence increases to 95% of the population. **G-H**: COVID mortality data generated from reported daily deaths (blue); estimated based on reported hospitalizations (purple); estimated from reported cases (green); and via a spline fit through all available data types (red, 95% UI in pink). **I-R**: Box plots showing 1,000 draws of fixed effect coefficients in a multivariate regression fit to $log(beta)$.

89 Texas: SEIR fit comparison

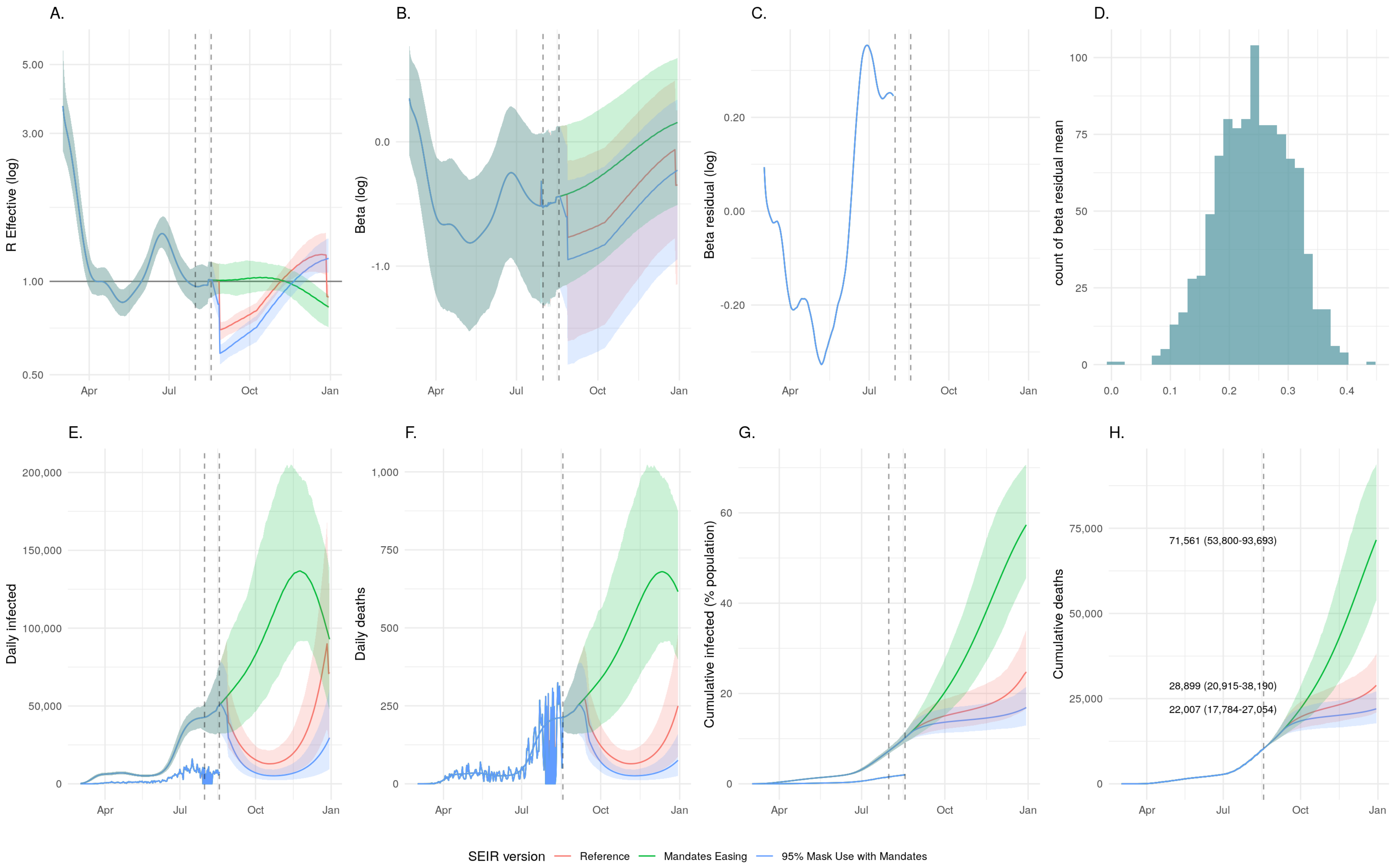

Texas: SEIR fit comparison. **A**: predicted R effective for each model through December 31. **B**: predicted SEIR *beta* parameter. **C**: residual of predicted *beta* and the observed value calculated directly from infection data over time. **D**: histogram of residual values for *beta*. Panels A, B, C, and D are all displayed in log space, reflecting the space in which the SEIR model is fit. **E**: predicted daily infections from each model through December 31. **F**: predicted daily deaths from each model through December 31. **G**: predicted cumulative infections through December 31, as a proportion of the total population. **H**: predicted cumulative deaths through December 31. In panels E, F, G, and H, reported death and infections are plotted alongside model predictions in light blue.

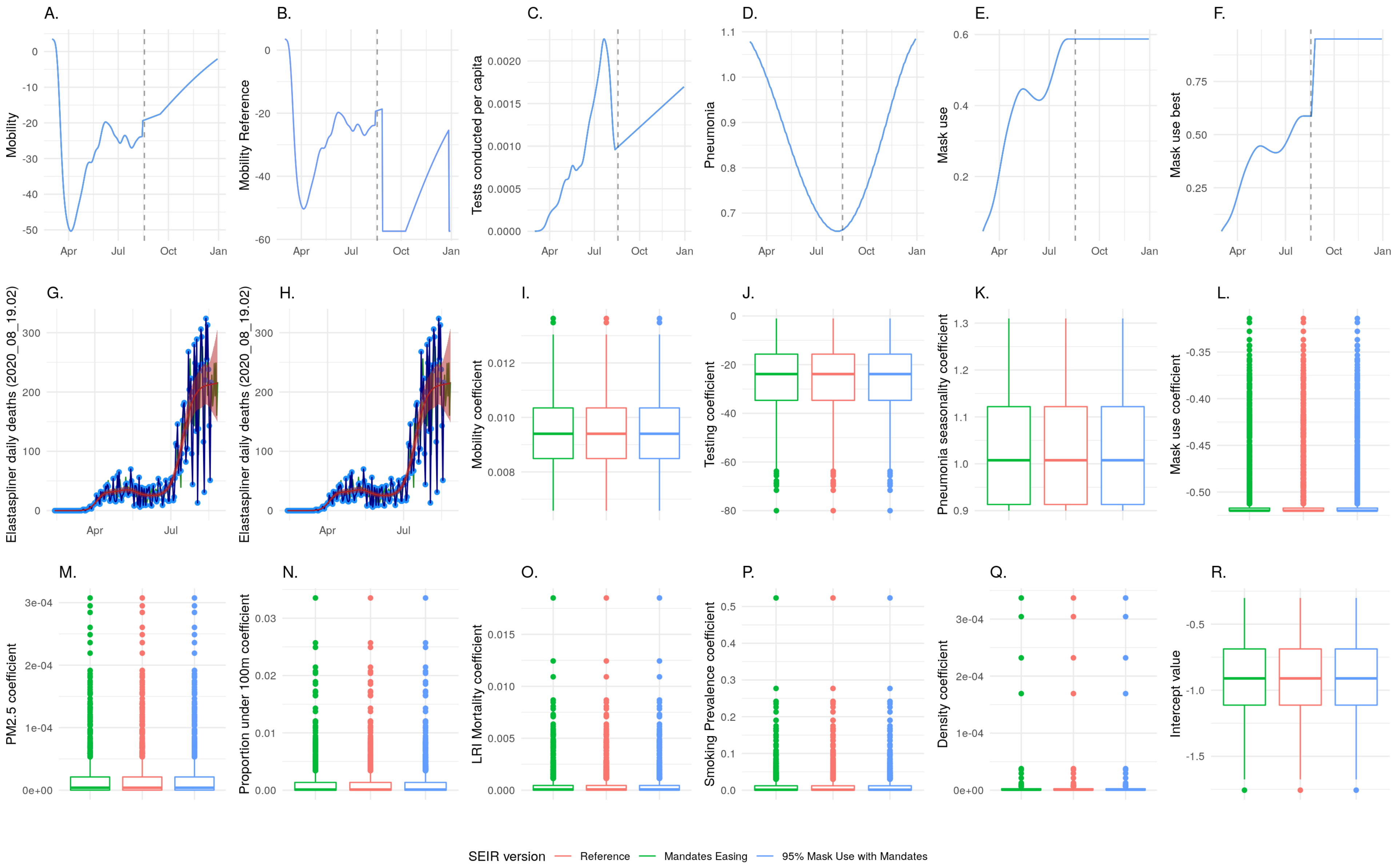

Texas: Covariate fits and regression coefficients. A-F: Line plots showing predicted covariate time trends for **A)** mobility in the absence of additional mandates; **B)** mobility with additional mandates applied; **C)** diagnostic testing per capita; **D)** pneumonia seasonality; **E)** mask use per capita, and; **F)** mask use in a scenario where adherence increases to 95% of the population. **G-H**: COVID mortality data generated from reported daily deaths (blue); estimated based on reported hospitalizations (purple); estimated from reported cases (green); and via a spline fit through all available data types (red, 95% UI in pink). **I-R**: Box plots showing 1,000 draws of fixed effect coefficients in a multivariate regression fit to $log(beta)$.

91 Utah: SEIR fit comparison

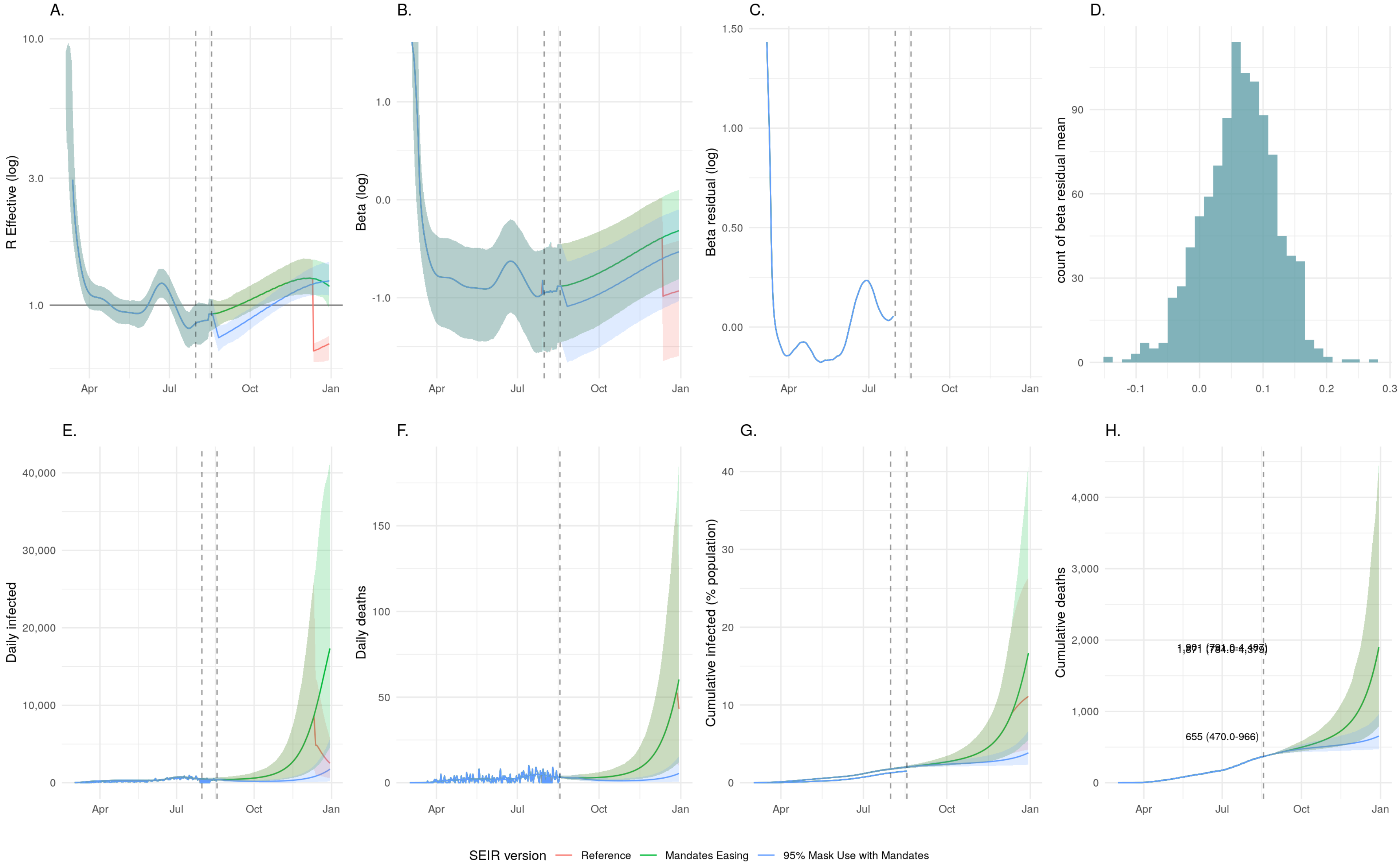

Utah: SEIR fit comparison. **A**: predicted R effective for each model through December 31. **B**: predicted SEIR *beta* parameter. **C**: residual of predicted *beta* and the observed value calculated directly from infection data over time. **D**: histogram of residual values for *beta*. Panels A, B, C, and D are all displayed in log space, reflecting the space in which the SEIR model is fit. **E**: predicted daily infections from each model through December 31. **F**: predicted daily deaths from each model through December 31. **G**: predicted cumulative infections through December 31, as a proportion of the total population. **H**: predicted cumulative deaths through December 31. In panels E, F, G, and H, reported death and infections are plotted alongside model predictions in light blue.

92 Utah: Covariate fits and regression coefficients

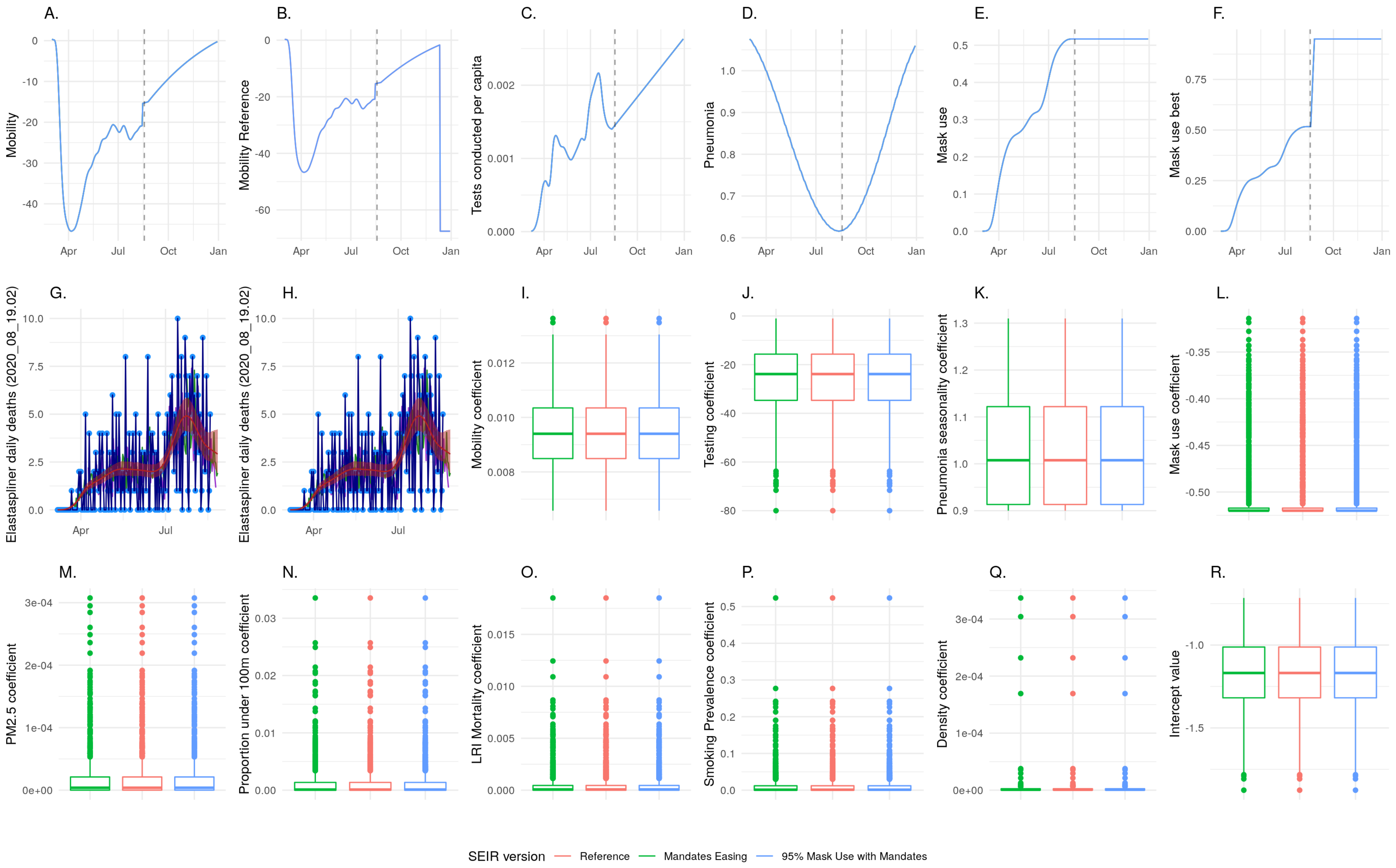

Utah: Covariate fits and regression coefficients. A-F: Line plots showing predicted covariate time trends for **A)** mobility in the absence of additional mandates; **B)** mobility with additional mandates applied; **C)** diagnostic testing per capita; **D)** pneumonia seasonality; **E)** mask use per capita, and; **F)** mask use in a scenario where adherence increases to 95% of the population. **G-H**: COVID mortality data generated from reported daily deaths (blue); estimated based on reported hospitalizations (purple); estimated from reported cases (green); and via a spline fit through all available data types (red, 95% UI in pink). **I-R**: Box plots showing 1,000 draws of fixed effect coefficients in a multivariate regression fit to $log(beta)$.

93 Vermont: SEIR fit comparison

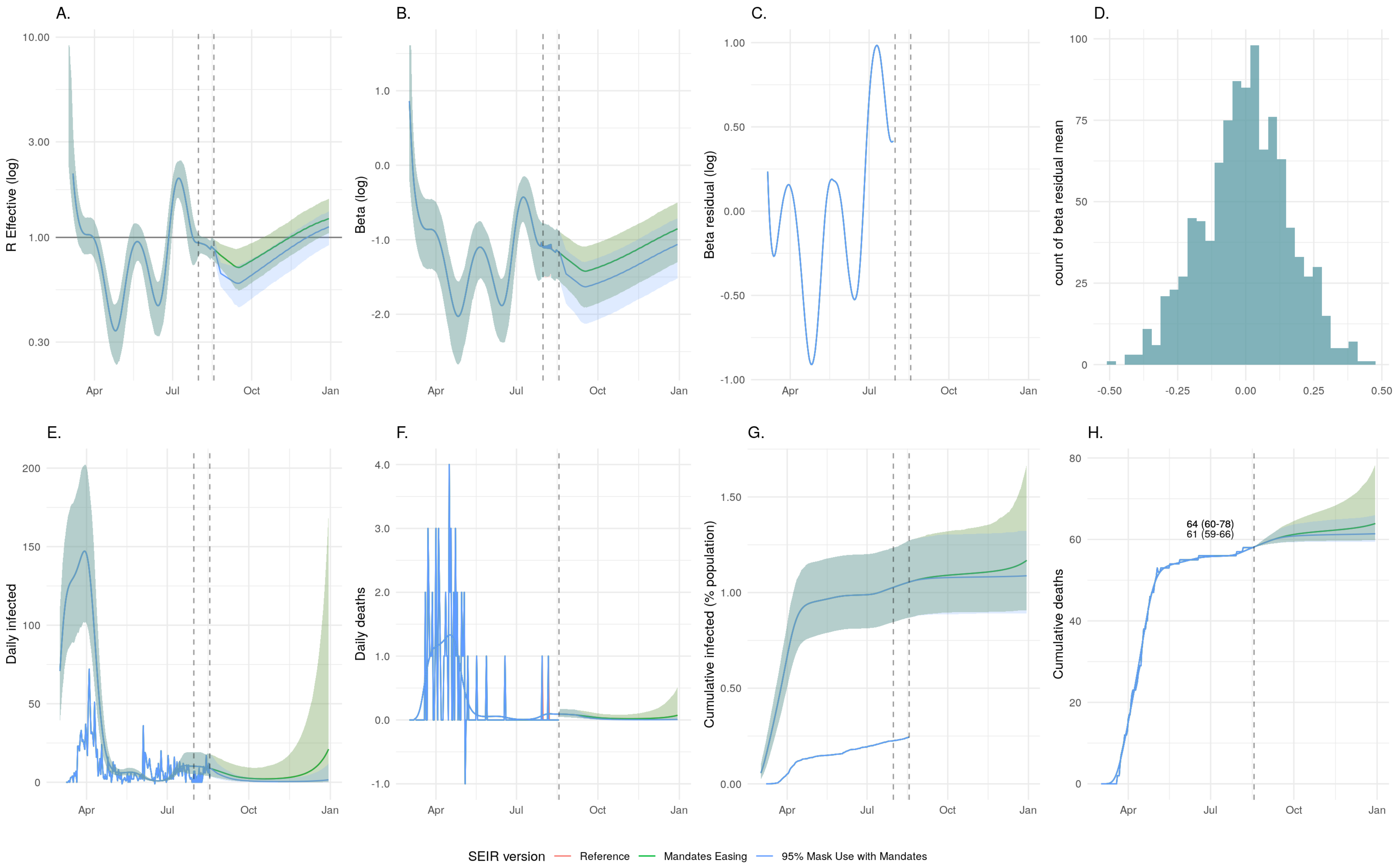

Vermont: SEIR fit comparison. **A**: predicted R effective for each model through December 31. **B**: predicted SEIR *beta* parameter. **C**: residual of predicted *beta* and the observed value calculated directly from infection data over time. **D**: histogram of residual values for *beta*. Panels A, B, C, and D are all displayed in log space, reflecting the space in which the SEIR model is fit. **E**: predicted daily infections from each model through December 31. **F**: predicted daily deaths from each model through December 31. **G**: predicted cumulative infections through December 31, as a proportion of the total population. **H**: predicted cumulative deaths through December 31. In panels E, F, G, and H, reported death and infections are plotted alongside model predictions in light blue.

94 Vermont: Covariate fits and regression coefficients

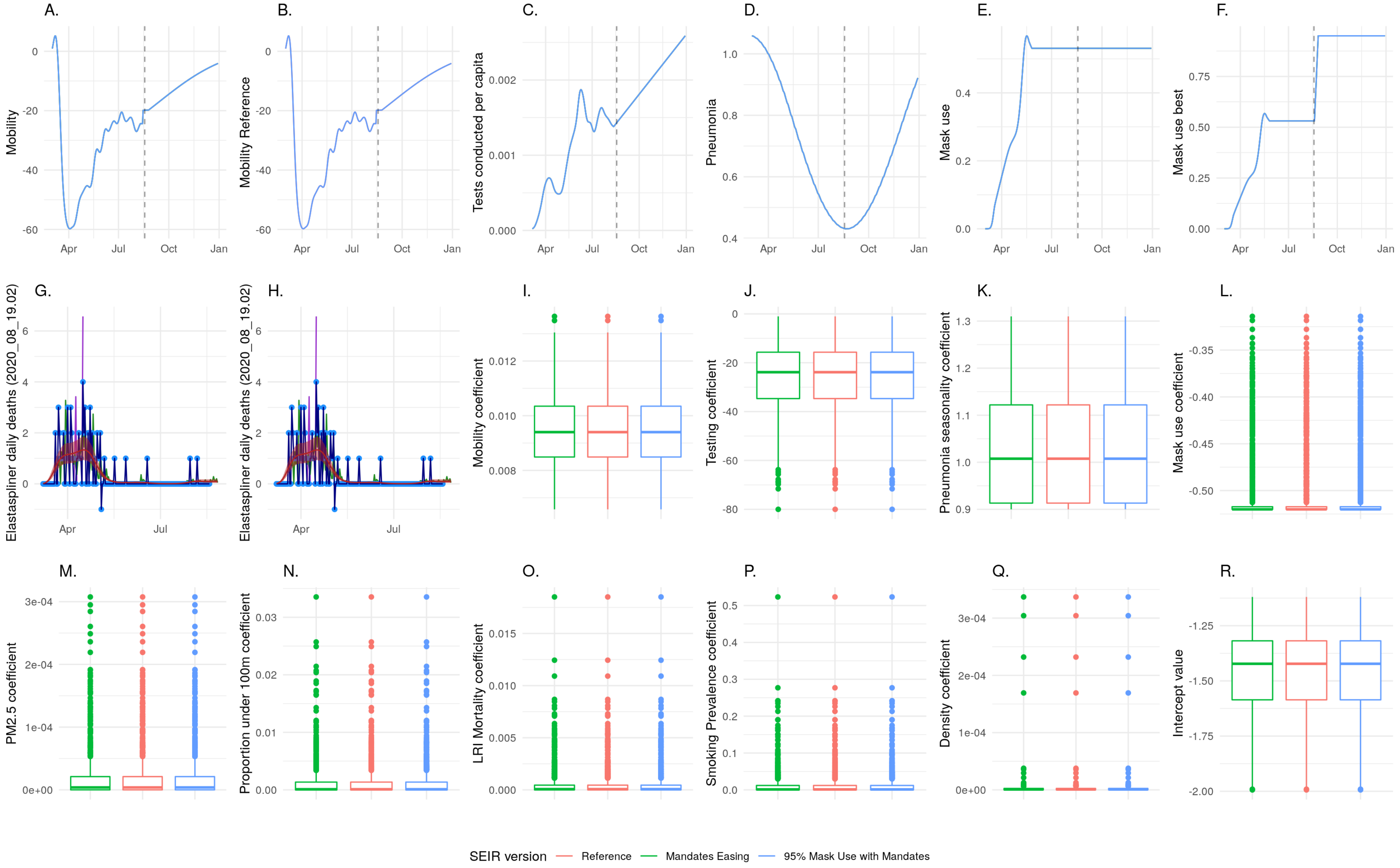

Vermont: Covariate fits and regression coefficients. **A-F**: Line plots showing predicted covariate time trends for **A)** mobility in the absence of additional mandates; **B)** mobility with additional mandates applied; **C)** diagnostic testing per capita; **D)** pneumonia seasonality; **E)** mask use per capita, and; **F)** mask use in a scenario where adherence increases to 95% of the population. **G-H**: COVID mortality data generated from reported daily deaths (blue); estimated based on reported hospitalizations (purple); estimated from reported cases (green); and via a spline fit through all available data types (red, 95% UI in pink). **I-R**: Box plots showing 1,000 draws of fixed effect coefficients in a multivariate regression fit to $log(beta)$.

95 Virginia: SEIR fit comparison

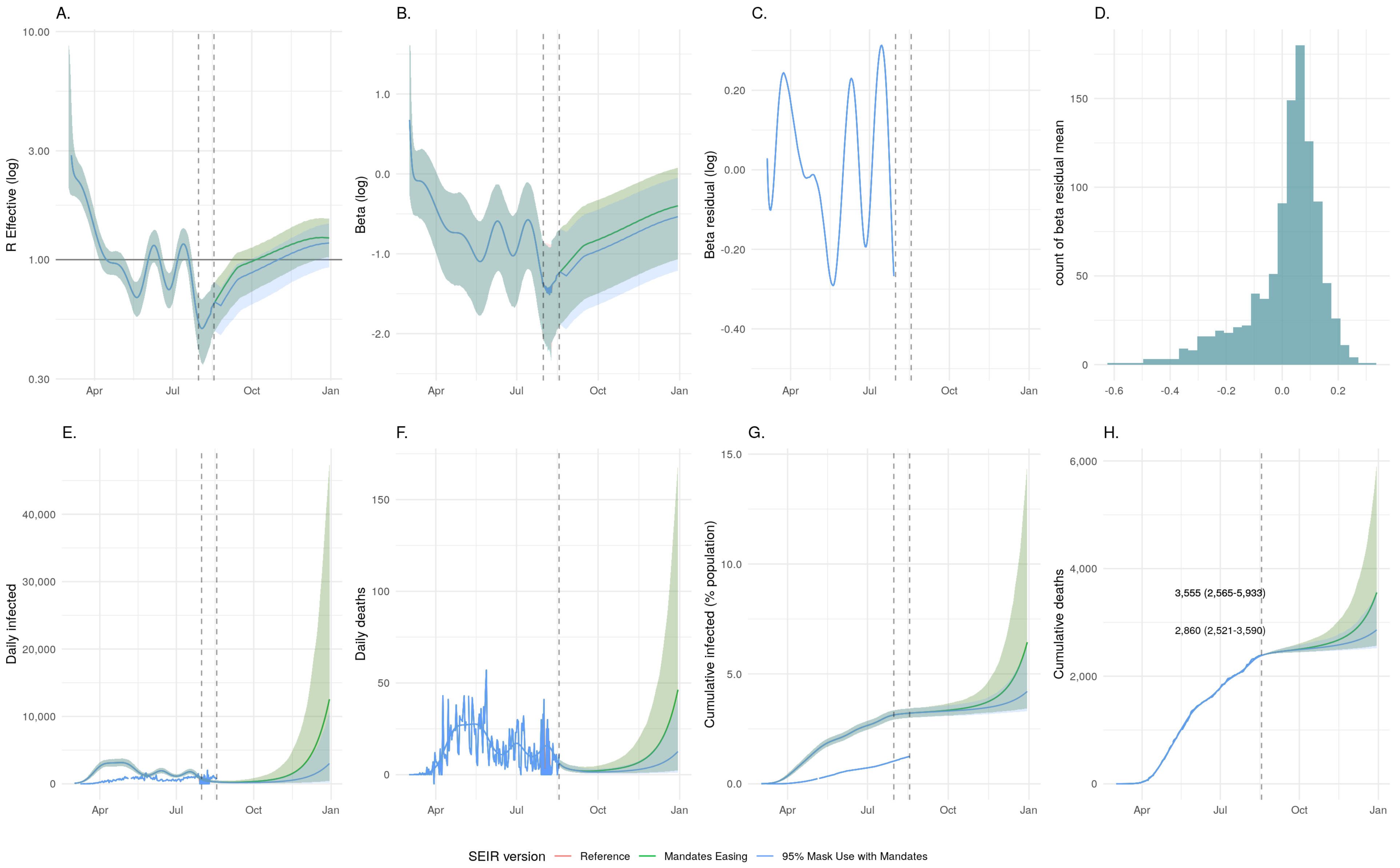

Virginia: SEIR fit comparison. A: predicted R effective for each model through December 31. **B**: predicted SEIR *beta* parameter. **C**: residual of predicted *beta* and the observed value calculated directly from infection data over time. **D**: histogram of residual values for *beta*. Panels A, B, C, and D are all displayed in log space, reflecting the space in which the SEIR model is fit. **E**: predicted daily infections from each model through December 31. **F**: predicted daily deaths from each model through December 31. **G**: predicted cumulative infections through December 31, as a proportion of the total population. **H**: predicted cumulative deaths through December 31. In panels E, F, G, and H, reported death and infections are plotted alongside model predictions in light blue.

96 Virginia: Covariate fits and regression coefficients

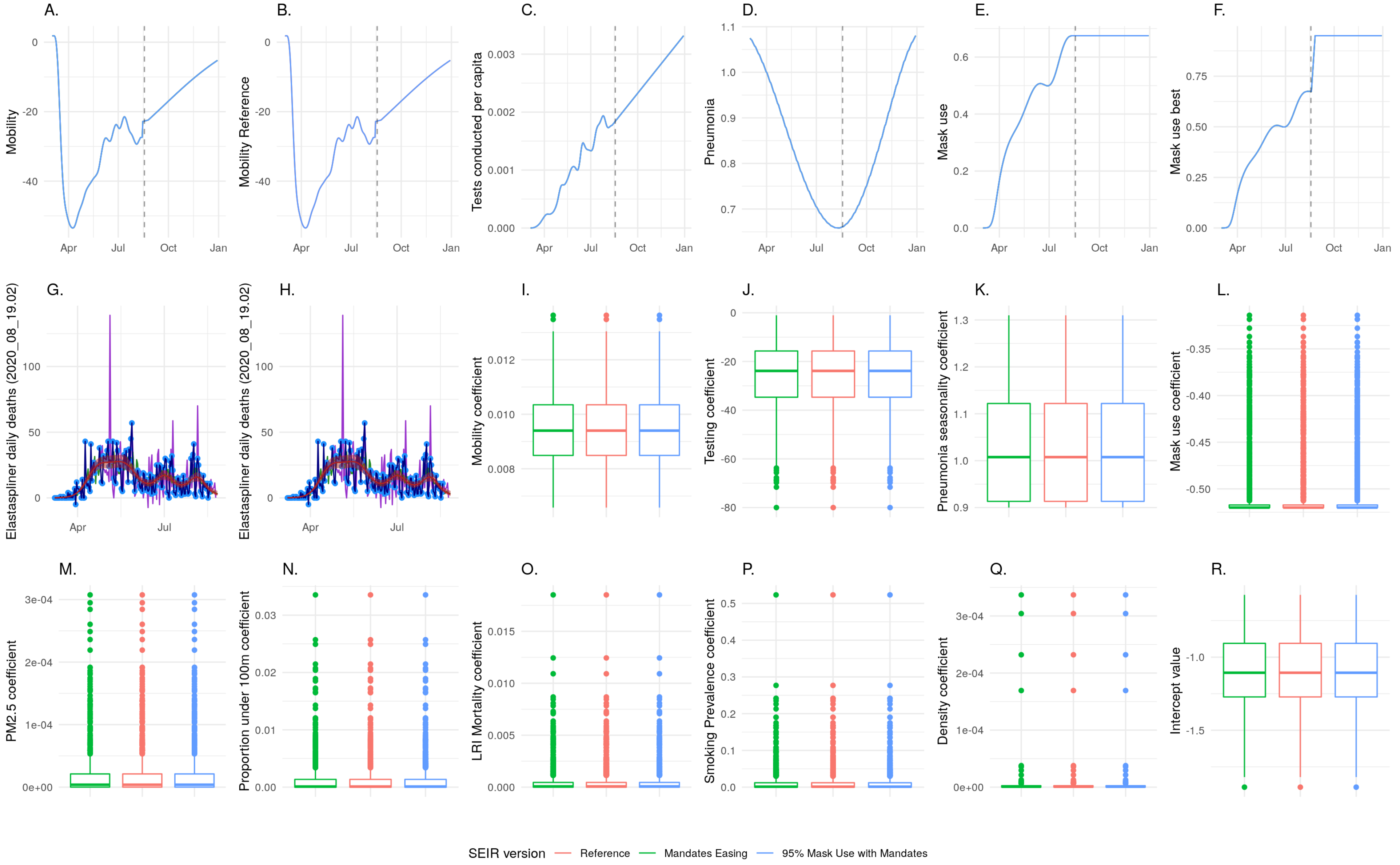

Virginia: Covariate fits and regression coefficients. A-F: Line plots showing predicted covariate time trends for **A)** mobility in the absence of additional mandates; **B)** mobility with additional mandates applied; **C)** diagnostic testing per capita; **D)** pneumonia seasonality; **E)** mask use per capita, and; **F)** mask use in a scenario where adherence increases to 95% of the population. **G-H**: COVID mortality data generated from reported daily deaths (blue); estimated based on reported hospitalizations (purple); estimated from reported cases (green); and via a spline fit through all available data types (red, 95% UI in pink). **I-R**: Box plots showing 1,000 draws of fixed effect coefficients in a multivariate regression fit to $log(beta)$.

97 Washington: SEIR fit comparison

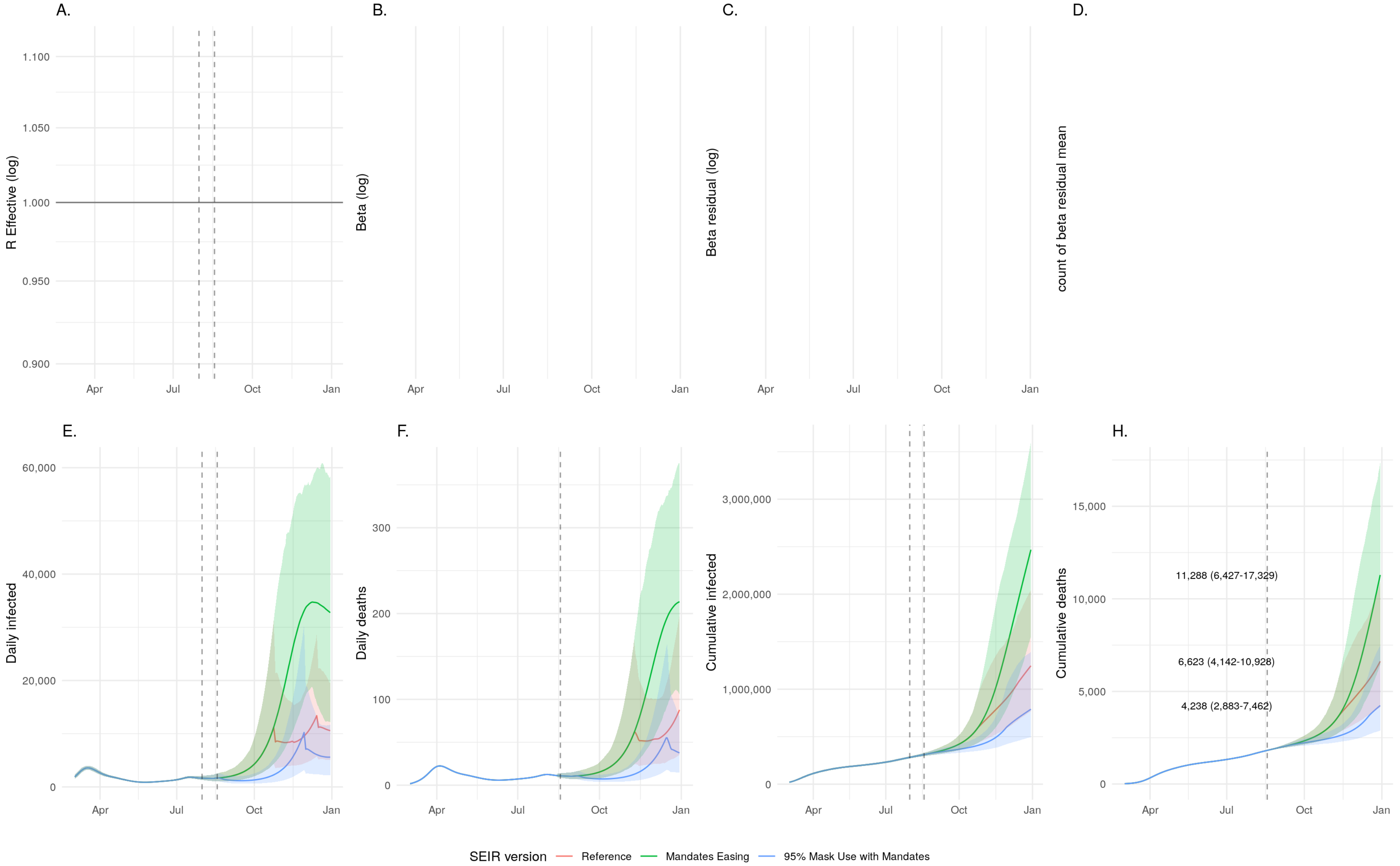

Washington: SEIR fit comparison. Panels **A-D** display values that are not directly calculated for aggregate locations. **E**: predicted daily infections from each model through December 31. **F**: predicted daily deaths from each model through December 31. **G**: predicted cumulative infections through December 31, as a proportion of the total population. **H**: predicted cumulative deaths through December 31. In panels E, F, G, and H, reported death and infections are plotted alongside model predictions in light blue.

98 Washington: Covariate fits and regression coefficients

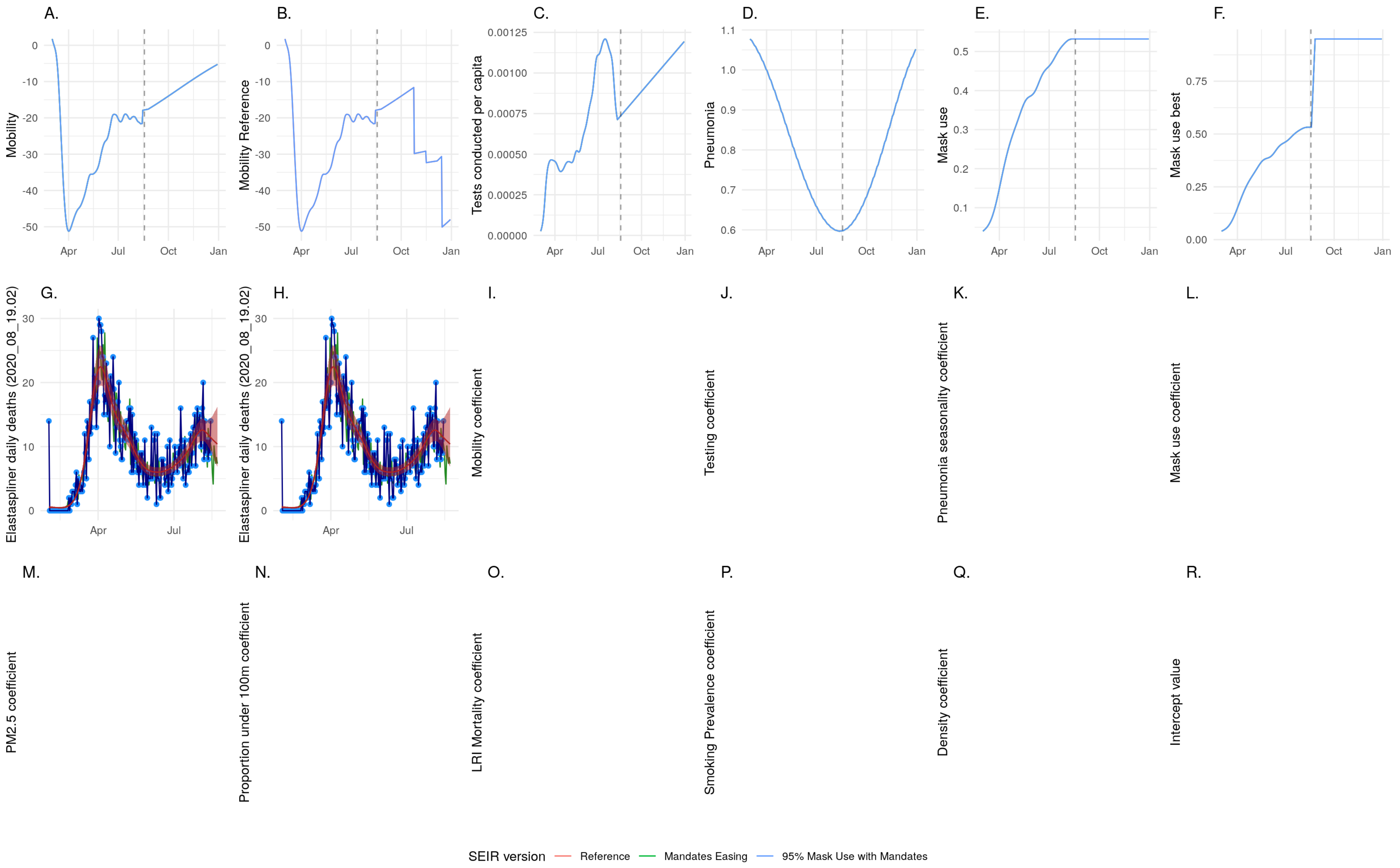

Washington: Covariate fits and regression coefficients. A-F: Line plots showing predicted covariate time trends for **A)** mobility in the absence of additional mandates; **B)** mobility with additional mandates applied; **C)** diagnostic testing per capita; **D)** pneumonia seasonality; **E)** mask use per capita, and; **F)** mask use in a scenario where adherence increases to 95% of the population. **G-H**: COVID mortality data generated from reported daily deaths (blue); estimated based on reported hospitalizations (purple); estimated from reported cases (green); and via a spline fit through all available data types (red, 95% UI in pink). Panels **I-R** display coefficients for a regression fit to *log*(*beta*), which is not applicable to aggregate locations.

99 West Virginia: SEIR fit comparison

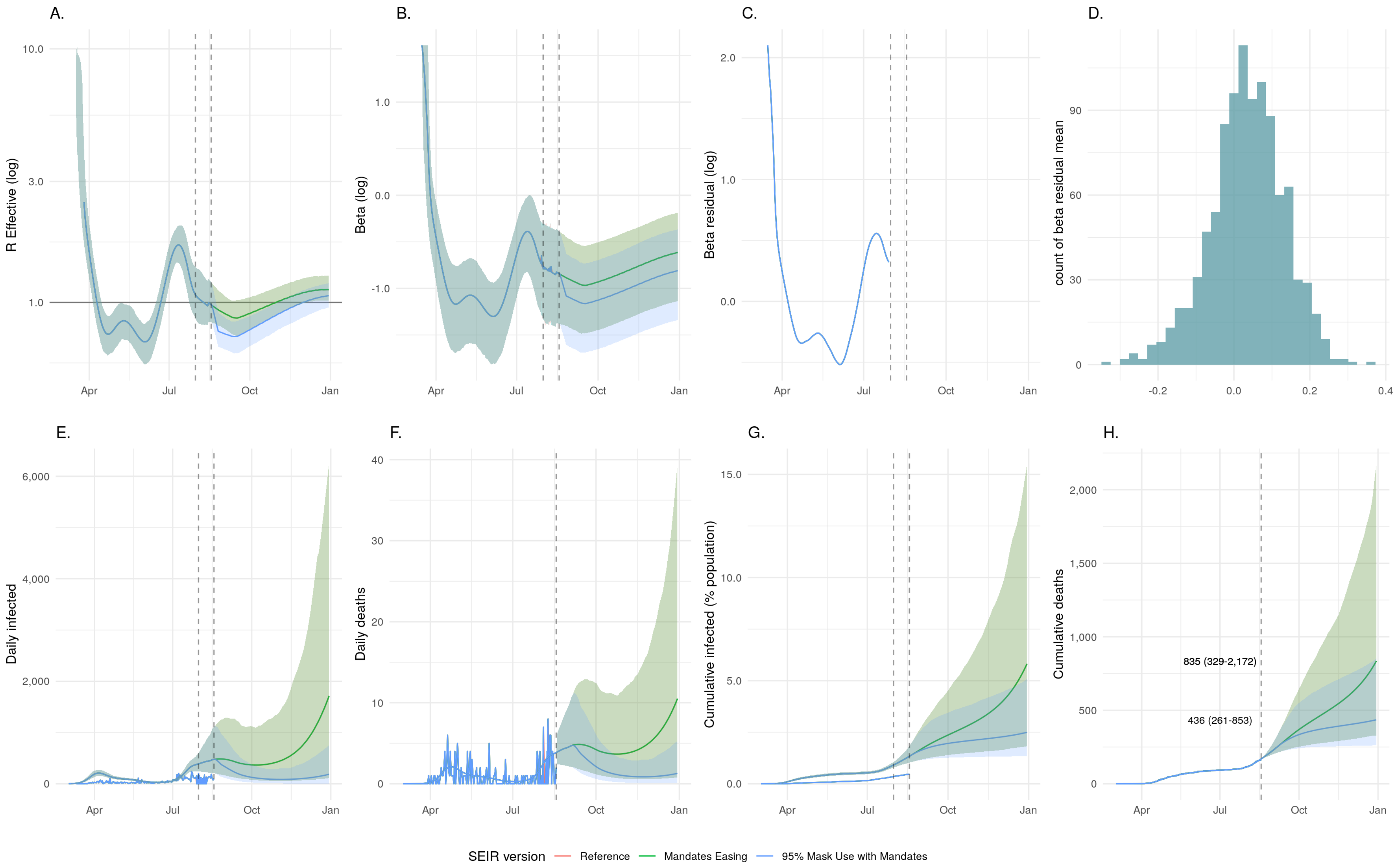

West Virginia: SEIR fit comparison. A: predicted R effective for each model through December 31. **B**: predicted SEIR *beta* parameter. **C**: residual of predicted *beta* and the observed value calculated directly from infection data over time. **D**: histogram of residual values for *beta*. Panels A, B, C, and D are all displayed in log space, reflecting the space in which the SEIR model is fit. **E**: predicted daily infections from each model through December 31. **F**: predicted daily deaths from each model through December 31. **G**: predicted cumulative infections through December 31, as a proportion of the total population. **H**: predicted cumulative deaths through December 31. In panels E, F, G, and H, reported death and infections are plotted alongside model predictions in light blue.

100 West Virginia: Covariate fits and regression coefficients

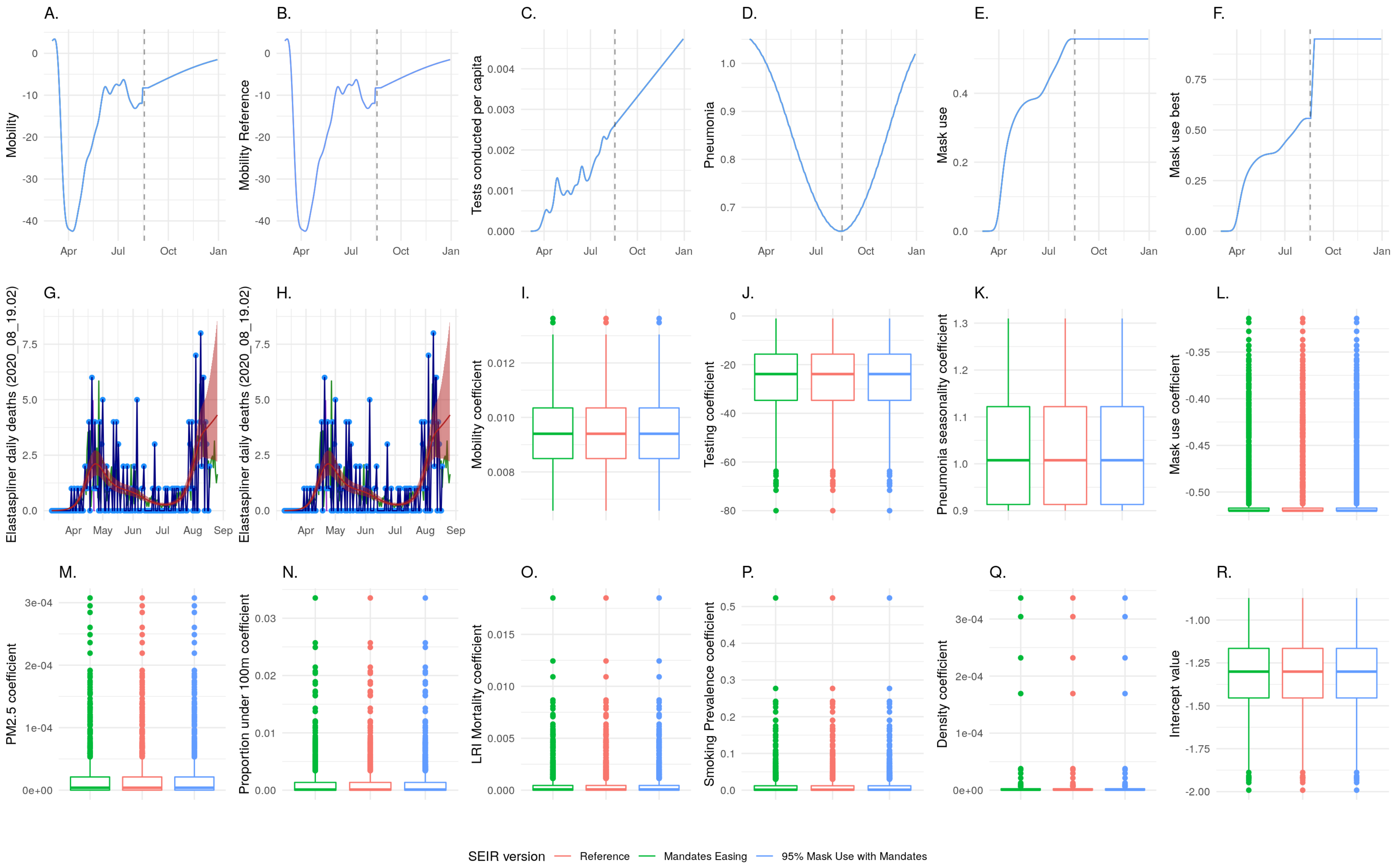

West Virginia: Covariate fits and regression coefficients. A-F: Line plots showing predicted covariate time trends for **A)** mobility in the absence of additional mandates; **B)** mobility with additional mandates applied; **C)** diagnostic testing per capita; **D)** pneumonia seasonality; **E)** mask use per capita, and; **F)** mask use in a scenario where adherence increases to 95% of the population. **G-H**: COVID mortality data generated from reported daily deaths (blue); estimated based on reported hospitalizations (purple); estimated from reported cases (green); and via a spline fit through all available data types (red, 95% UI in pink). **I-R**: Box plots showing 1,000 draws of fixed effect coefficients in a multivariate regression fit to $log(beta)$.

101 Wisconsin: SEIR fit comparison

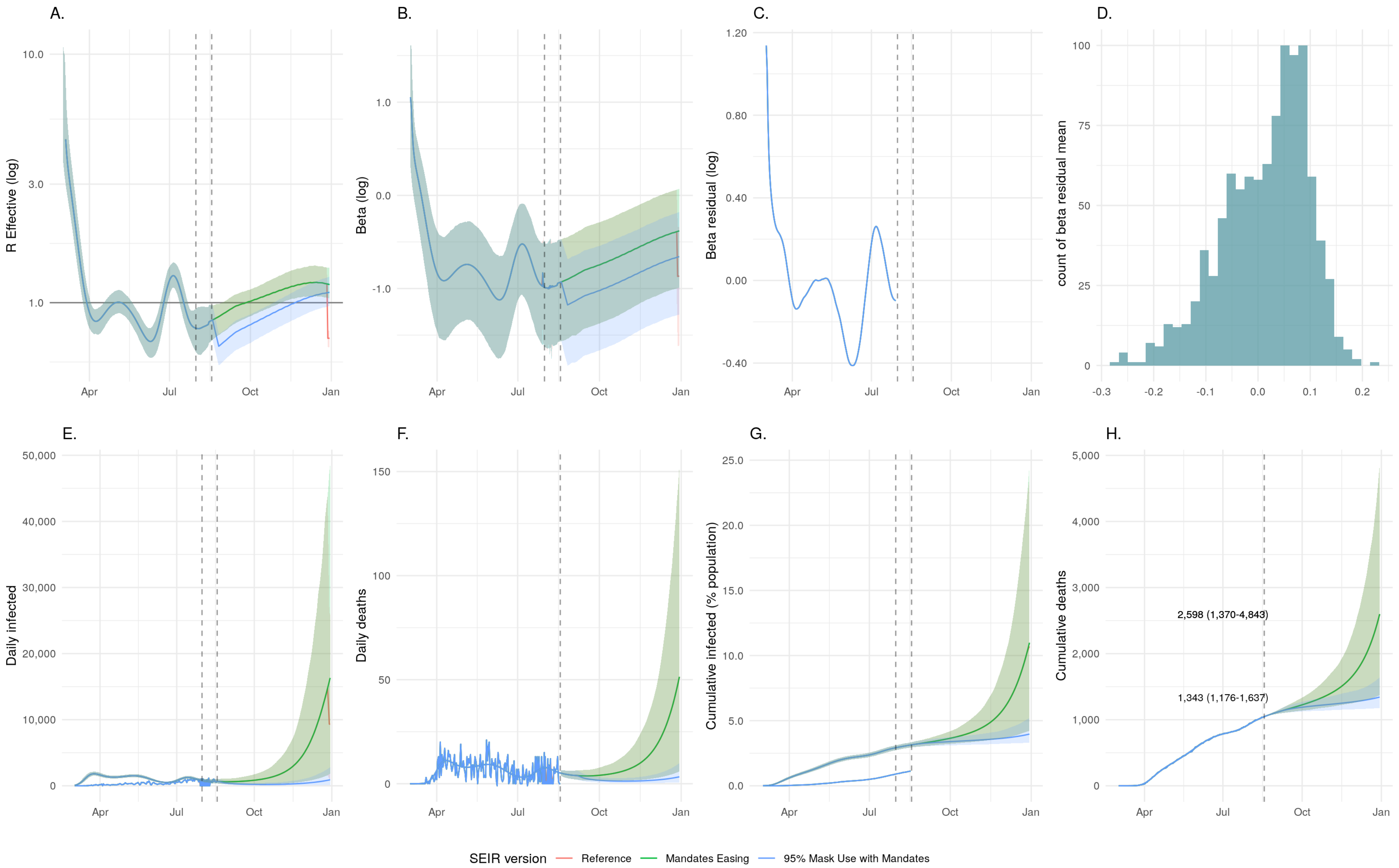

Wisconsin: SEIR fit comparison. A: predicted R effective for each model through December 31. **B**: predicted SEIR *beta* parameter. **C**: residual of predicted *beta* and the observed value calculated directly from infection data over time. **D**: histogram of residual values for *beta*. Panels A, B, C, and D are all displayed in log space, reflecting the space in which the SEIR model is fit. **E**: predicted daily infections from each model through December 31. **F**: predicted daily deaths from each model through December 31. **G**: predicted cumulative infections through December 31, as a proportion of the total population. **H**: predicted cumulative deaths through December 31. In panels E, F, G, and H, reported death and infections are plotted alongside model predictions in light blue.

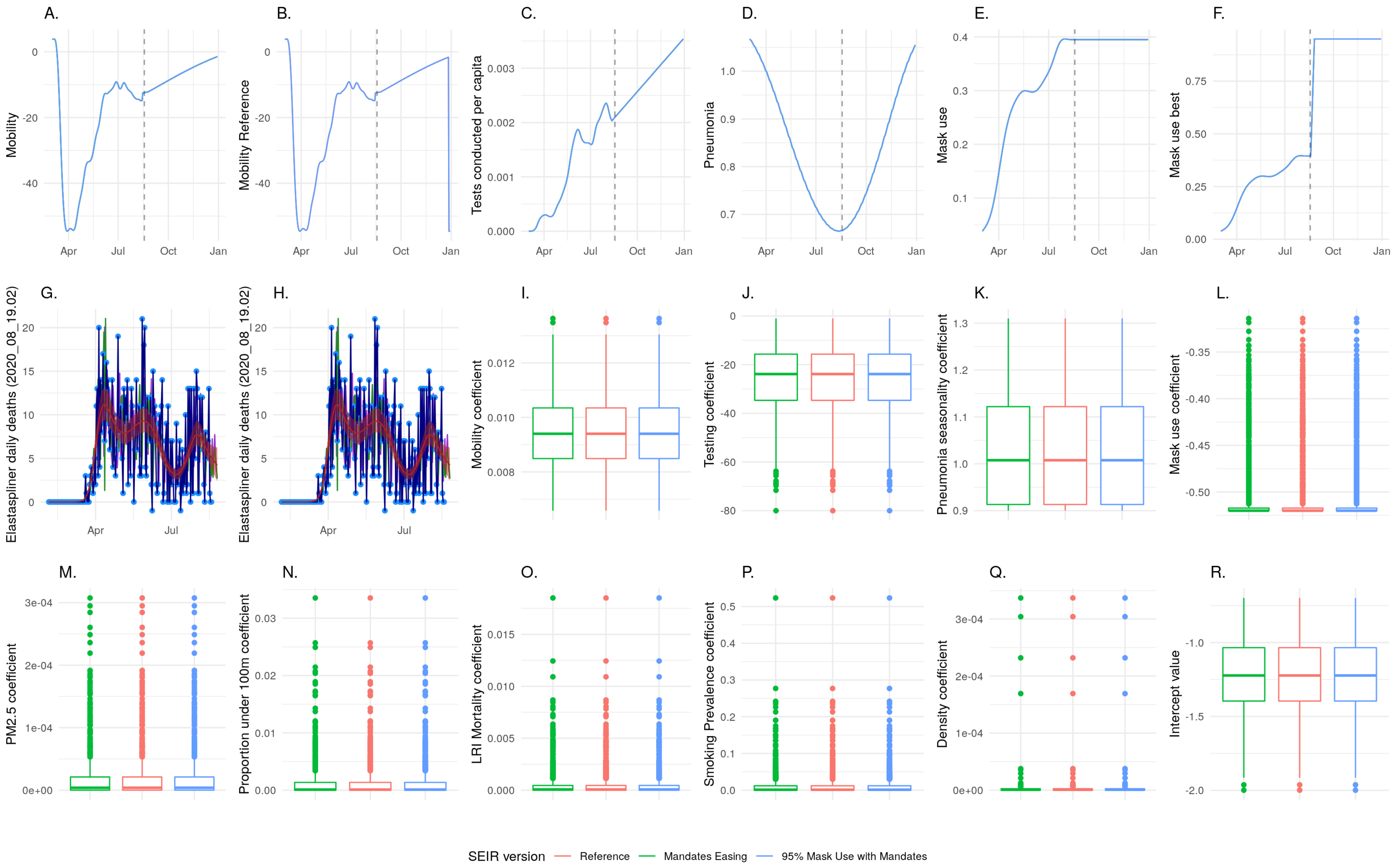

Wisconsin: Covariate fits and regression coefficients. A-F: Line plots showing predicted covariate time trends for **A)** mobility in the absence of additional mandates; **B)** mobility with additional mandates applied; **C)** diagnostic testing per capita; **D)** pneumonia seasonality; **E)** mask use per capita, and; **F)** mask use in a scenario where adherence increases to 95% of the population. **G-H**: COVID mortality data generated from reported daily deaths (blue); estimated based on reported hospitalizations (purple); estimated from reported cases (green); and via a spline fit through all available data types (red, 95% UI in pink). **I-R**: Box plots showing 1,000 draws of fixed effect coefficients in a multivariate regression fit to $log(beta)$.

103 Wyoming: SEIR fit comparison

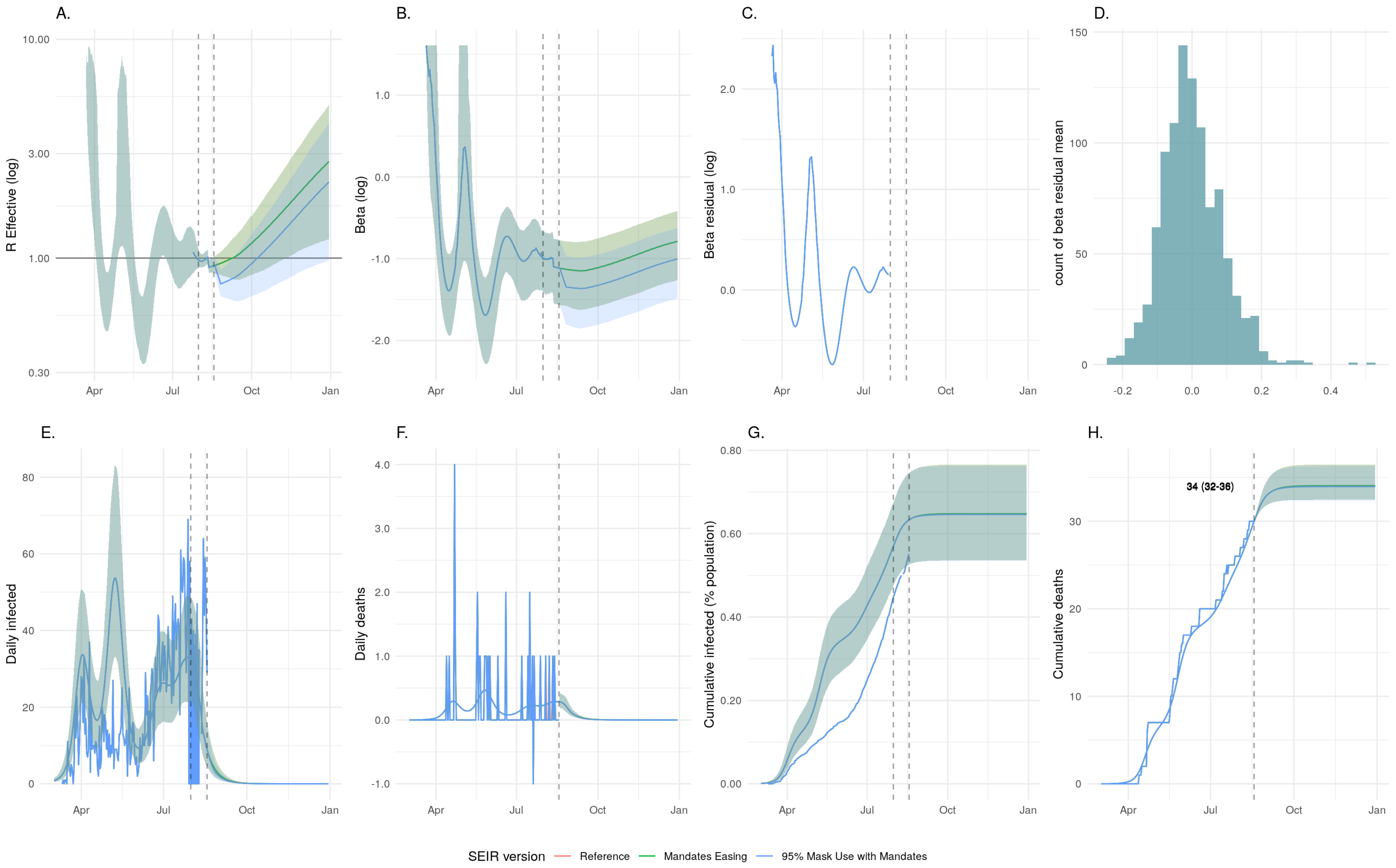

Wyoming: SEIR fit comparison. A: predicted R effective for each model through December 31. **B**: predicted SEIR *beta* parameter. **C**: residual of predicted *beta* and the observed value calculated directly from infection data over time. **D**: histogram of residual values for *beta*. Panels A, B, C, and D are all displayed in log space, reflecting the space in which the SEIR model is fit. **E**: predicted daily infections from each model through December 31. **F**: predicted daily deaths from each model through December 31. **G**: predicted cumulative infections through December 31, as a proportion of the total population. **H**: predicted cumulative deaths through December 31. In panels E, F, G, and H, reported death and infections are plotted alongside model predictions in light blue.

104 Wyoming: Covariate fits and regression coefficients

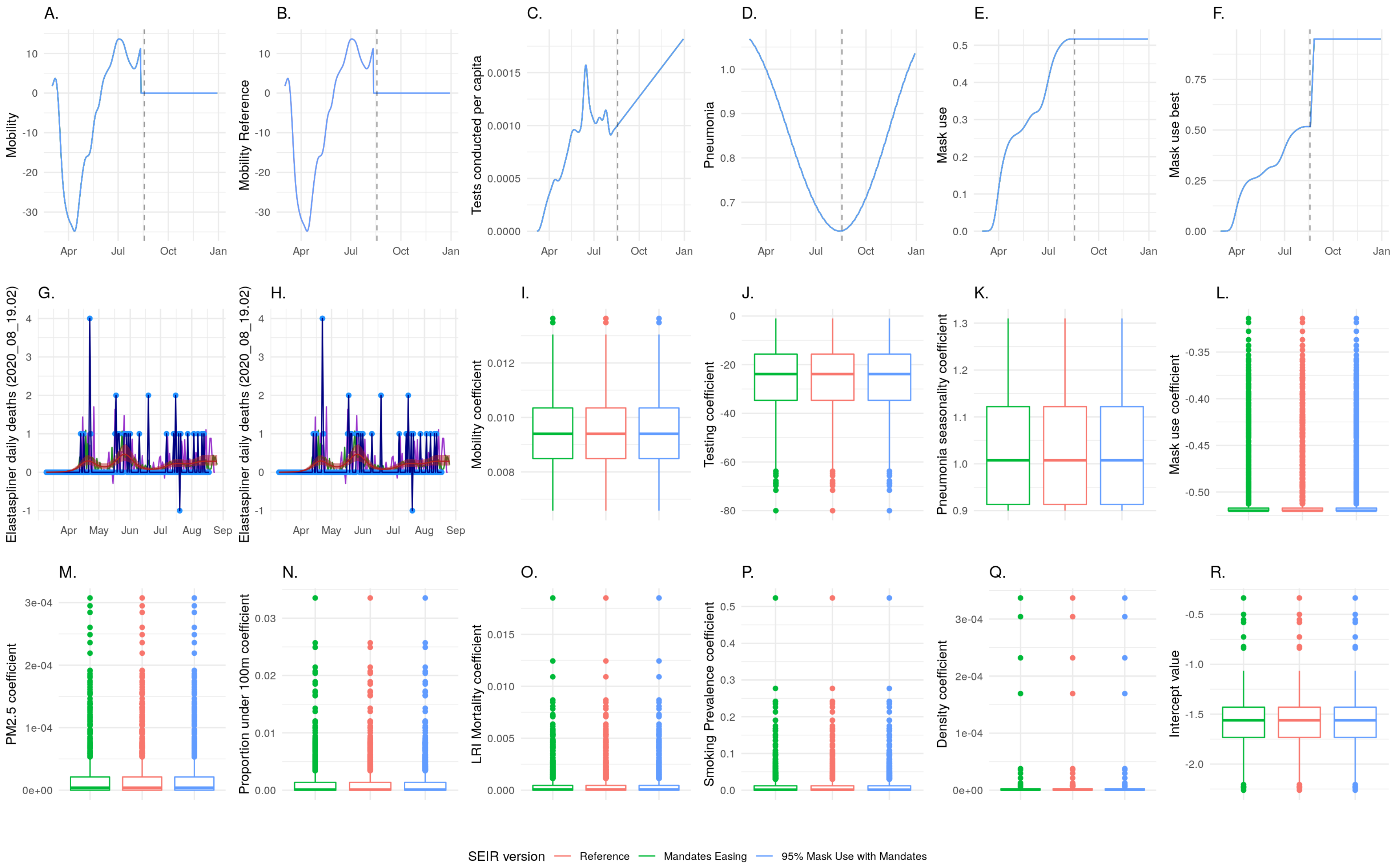

Wyoming: Covariate fits and regression coefficients. A-F: Line plots showing predicted covariate time trends for **A)** mobility in the absence of additional mandates; **B)** mobility with additional mandates applied; **C)** diagnostic testing per capita; **D)** pneumonia seasonality; **E)** mask use per capita, and; **F)** mask use in a scenario where adherence increases to 95% of the population. **G-H**: COVID mortality data generated from reported daily deaths (blue); estimated based on reported hospitalizations (purple); estimated from reported cases (green); and via a spline fit through all available data types (red, 95% UI in pink). **I-R**: Box plots showing 1,000 draws of fixed effect coefficients in a multivariate regression fit to $log(beta)$.

105 Spokane County: SEIR fit comparison

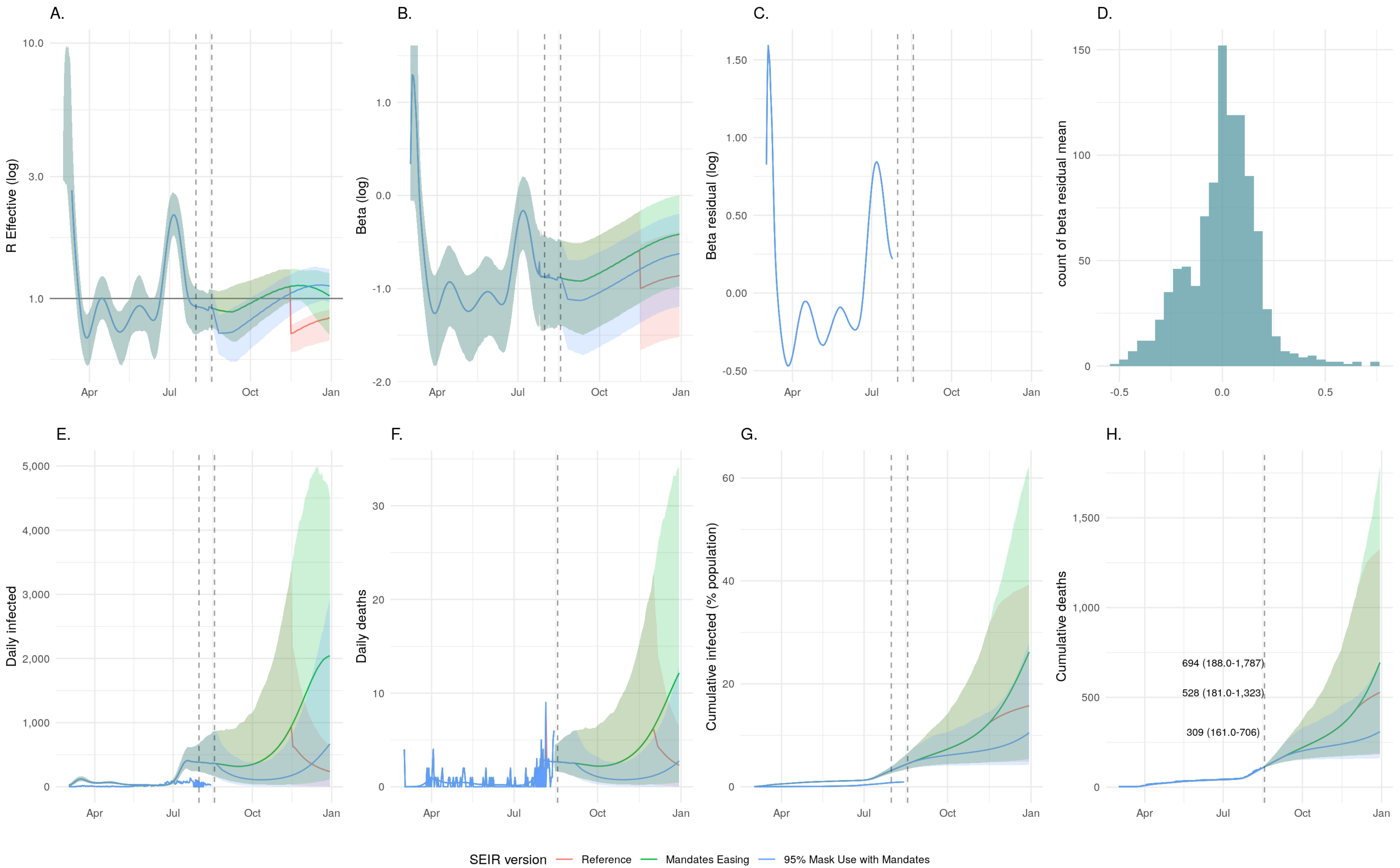

Spokane County: SEIR fit comparison. **A**: predicted R effective for each model through December 31. **B**: predicted SEIR *beta* parameter. **C**: residual of predicted *beta* and the observed value calculated directly from infection data over time. **D**: histogram of residual values for *beta*. Panels A, B, C, and D are all displayed in log space, reflecting the space in which the SEIR model is fit. **E**: predicted daily infections from each model through December 31. **F**: predicted daily deaths from each model through December 31. **G**: predicted cumulative infections through December 31, as a proportion of the total population. **H**: predicted cumulative deaths through December 31. In panels E, F, G, and H, reported death and infections are plotted alongside model predictions in light blue.

106 Spokane County: Covariate fits and regression coefficients

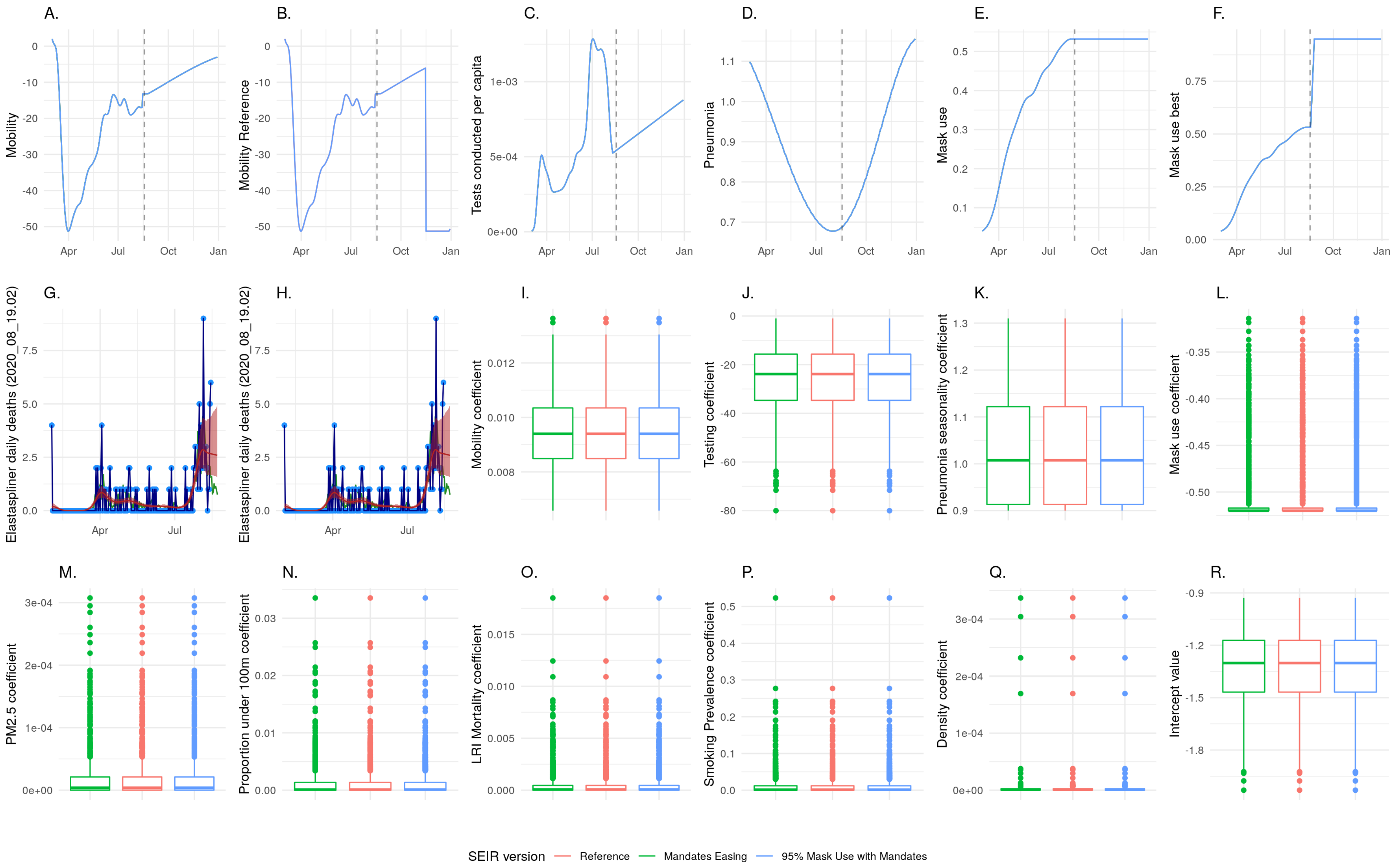

Spokane County: Covariate fits and regression coefficients. A-F: Line plots showing predicted covariate time trends for **A)** mobility in the absence of additional mandates; **B)** mobility with additional mandates applied; **C)** diagnostic testing per capita; **D)** pneumonia seasonality; **E)** mask use per capita, and; **F)** mask use in a scenario where adherence increases to 95% of the population. **G-H**: COVID mortality data generated from reported daily deaths (blue); estimated based on reported hospitalizations (purple); estimated from reported cases (green); and via a spline fit through all available data types (red, 95% UI in pink). **I-R**: Box plots showing 1,000 draws of fixed effect coefficients in a multivariate regression fit to $log(beta)$.

107 King and Snohomish Counties: SEIR fit comparison

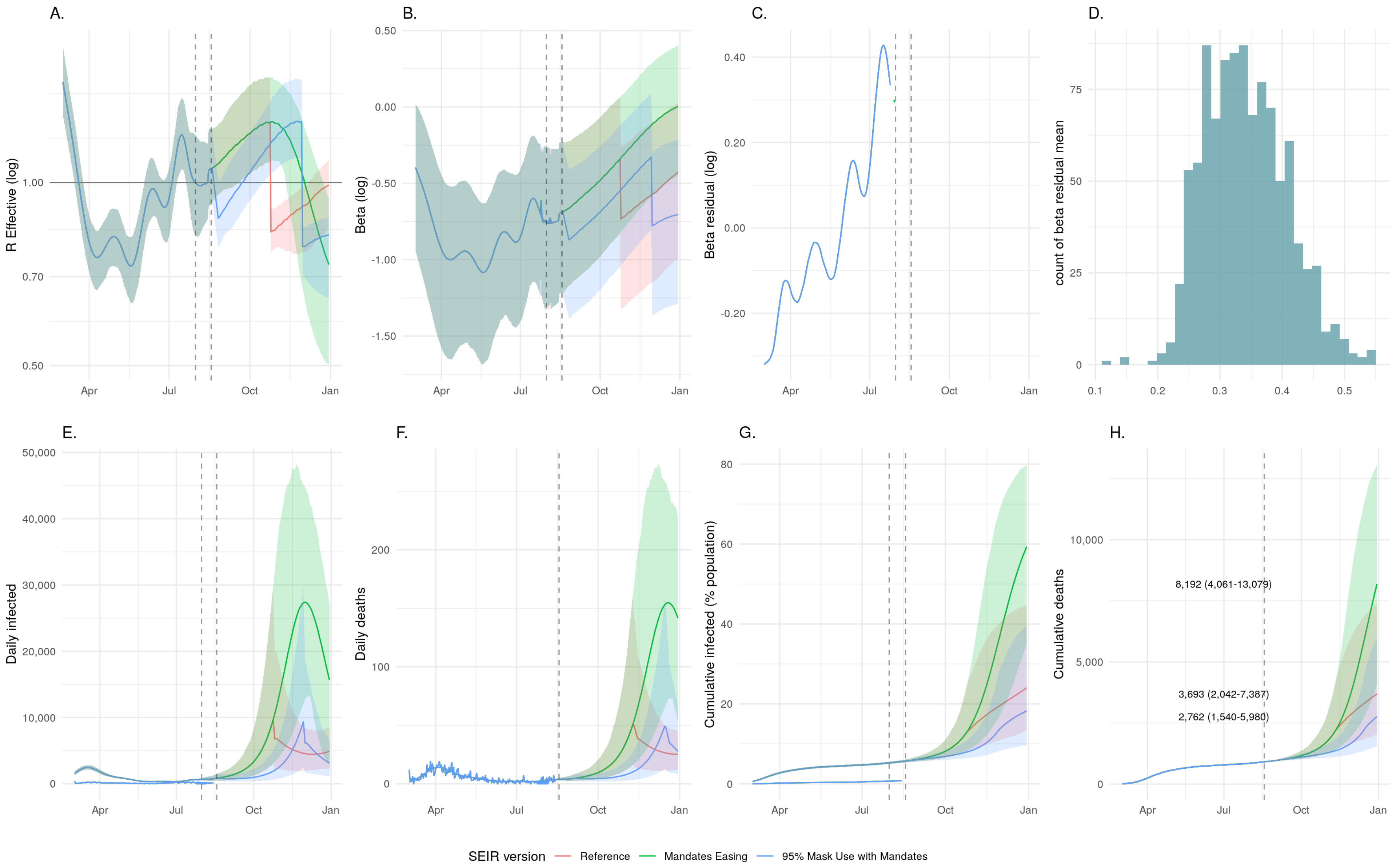

King and Snohomish Counties: SEIR fit comparison. **A**: predicted R effective for each model through December 31. **B**: predicted SEIR *beta* parameter. **C**: residual of predicted *beta* and the observed value calculated directly from infection data over time. **D**: histogram of residual values for *beta*. Panels A, B, C, and D are all displayed in log space, reflecting the space in which the SEIR model is fit. **E**: predicted daily infections from each model through December 31. **F**: predicted daily deaths from each model through December 31. **G**: predicted cumulative infections through December 31, as a proportion of the total population. **H**: predicted cumulative deaths through December 31. In panels E, F, G, and H, reported death and infections are plotted alongside model predictions in light blue.

108 King and Snohomish Counties: Covariate fits and regression coefficients

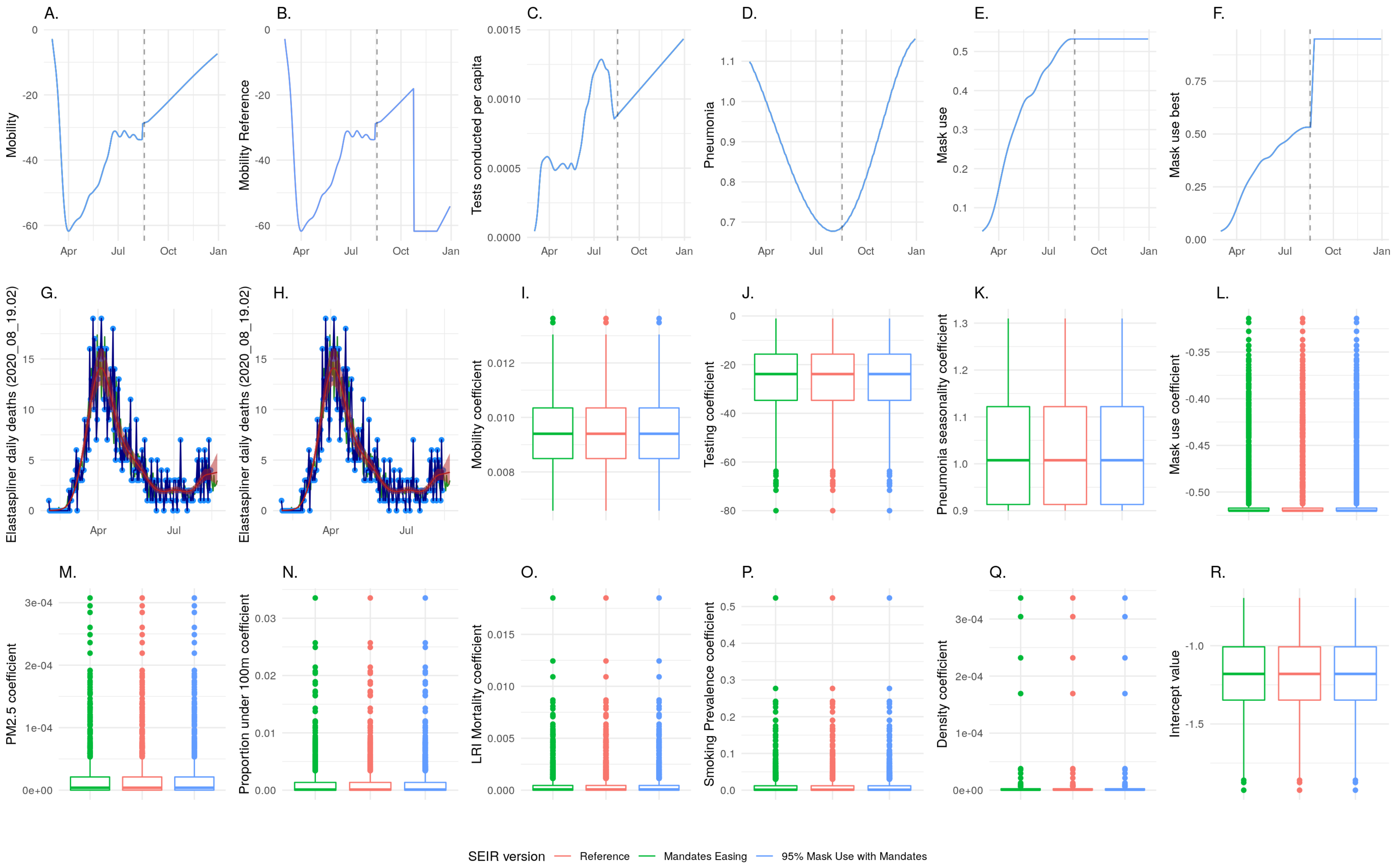

King and Snohomish Counties: Covariate fits and regression coefficients. A-F: Line plots showing predicted covariate time trends for **A)** mobility in the absence of additional mandates; **B)** mobility with additional mandates applied; **C)** diagnostic testing per capita; **D)** pneumonia seasonality; **E)** mask use per capita, and; **F)** mask use in a scenario where adherence increases to 95% of the population. **G-H**: COVID mortality data generated from reported daily deaths (blue); estimated based on reported hospitalizations (purple); estimated from reported cases (green); and via a spline fit through all available data types (red, 95% UI in pink). **I-R**: Box plots showing 1,000 draws of fixed effect coefficients in a multivariate regression fit to $log(beta)$.

109 Washington except for King, Snohomish, and Spokane Counties: SEIR fit comparison

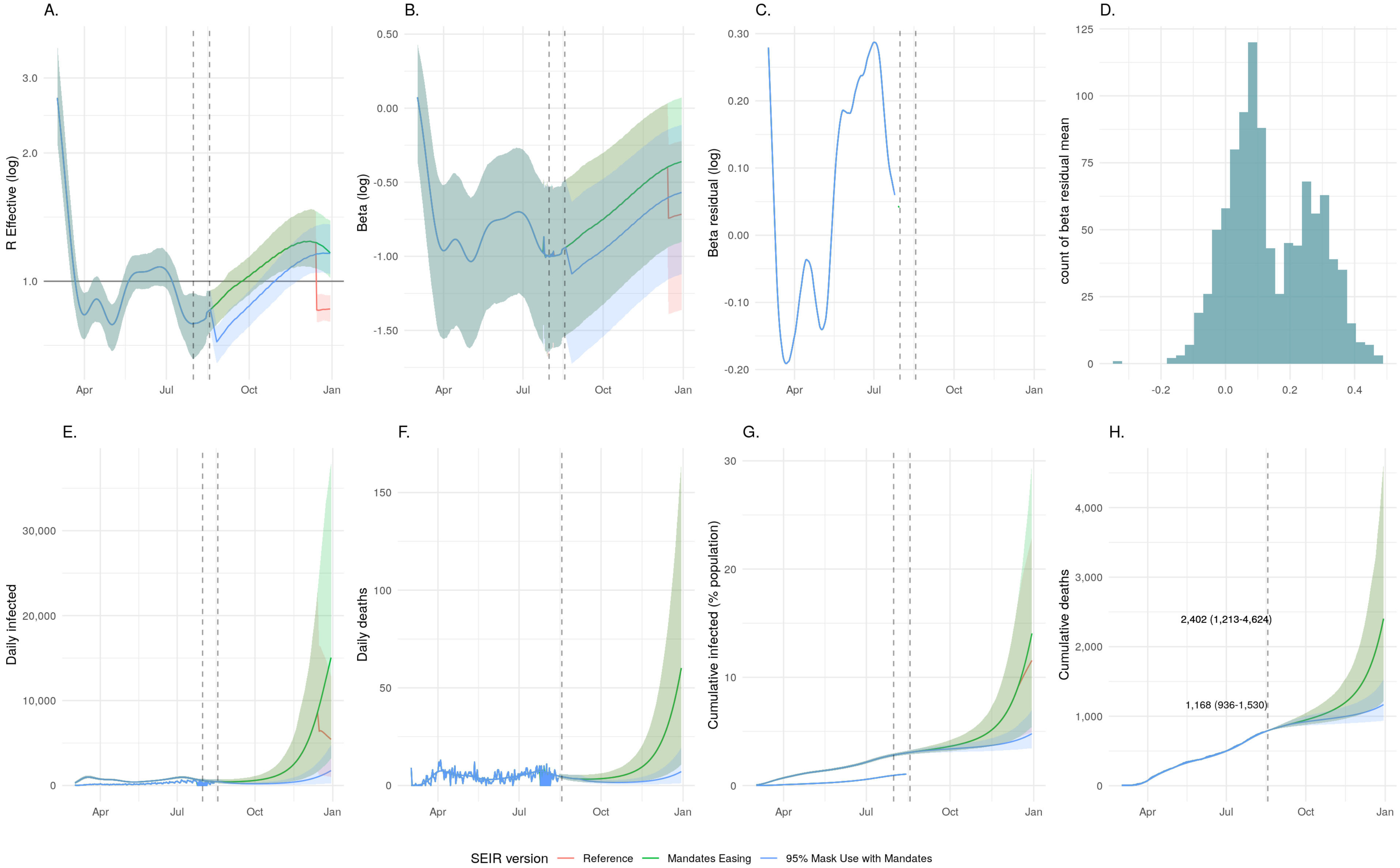

Washington except for King, Snohomish, and Spokane Counties: SEIR fit comparison. **A**: predicted R effective for each model through December 31. **B**: predicted SEIR *beta* parameter. **C**: residual of predicted *beta* and the observed value calculated directly from infection data over time. **D**: histogram of residual values for *beta*. Panels A, B, C, and D are all displayed in log space, reflecting the space in which the SEIR model is fit. **E**: predicted daily infections from each model through December 31. **F**: predicted daily deaths from each model through December 31. **G**: predicted cumulative infections through December 31, as a proportion of the total population. **H**: predicted cumulative deaths through December 31. In panels E, F, G, and H, reported death and infections are plotted alongside model predictions in

light blue.

110 Washington except for King, Snohomish, and Spokane Counties: Covariate fits and regression coefficients

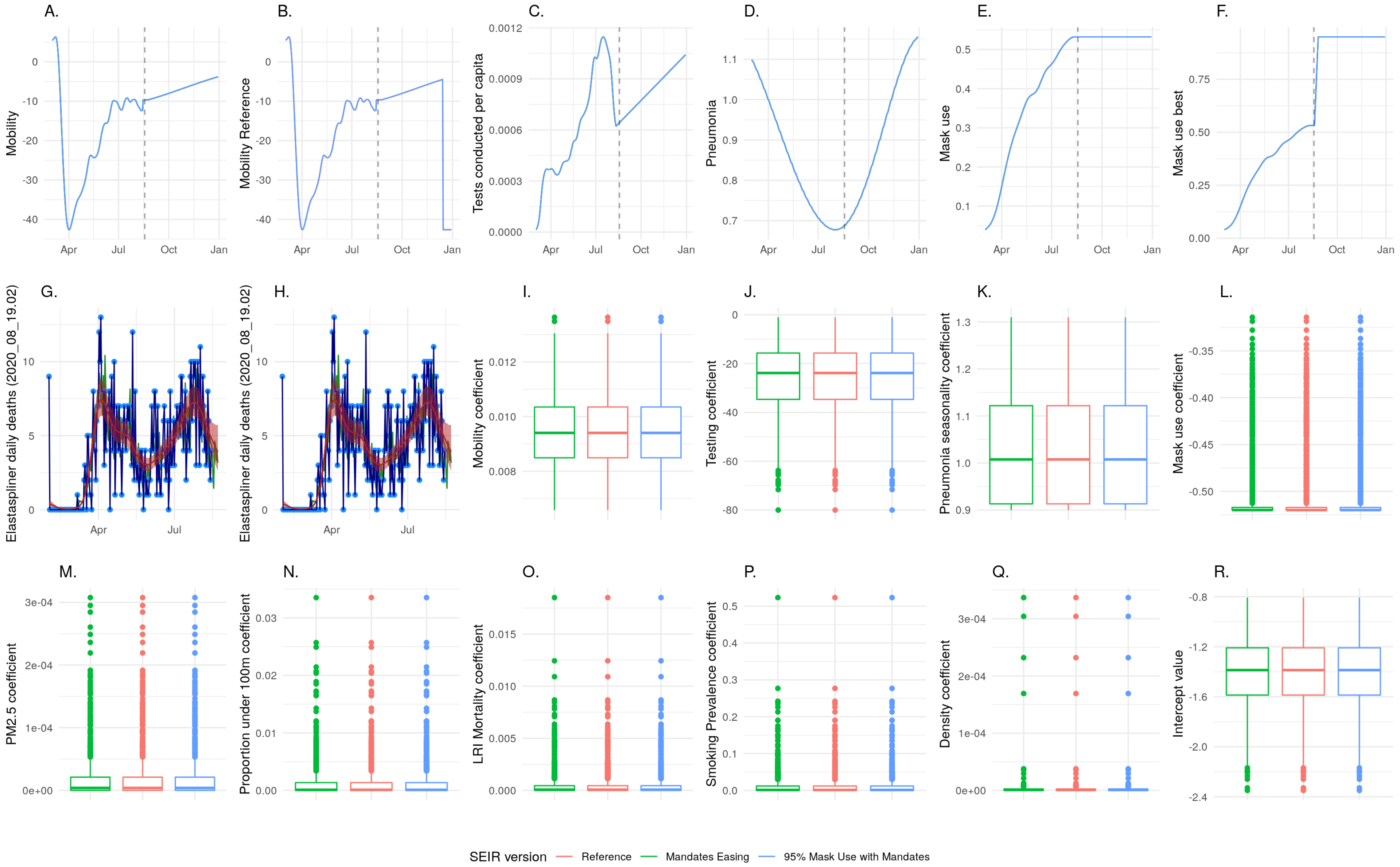

Washington except for King, Snohomish, and Spokane Counties: Covariate fits and regression coefficients. A-F: Line plots showing predicted covariate time trends for **A)** mobility in the absence of additional mandates; **B)** mobility with additional mandates applied; **C)** diagnostic testing per capita; **D)** pneumonia seasonality; **E)** mask use per capita, and; **F)** mask use in a scenario where adherence increases to 95% of the population. **G-H**: COVID mortality data generated from

reported daily deaths (blue); estimated based on reported hospitalizations (purple); estimated from reported cases (green); and via a spline fit through all available data types (red, 95% UI in pink). **I-R**: Box plots showing 1,000 draws of fixed effect coefficients in a multivariate regression fit to $log(beta)$.
