# Supplementary material for: Modeling COVID-19 scenarios for the United States
Source: Nat Med. 2020 Oct 23;27(1):94–105. doi: 10.1038/s41591-020-1132-9 (PMC7806509; doi:10.1038/s41591-020-1132-9)
Supplement: Supplementary file 5 — Appendix 3: SEIR model vetting plots for the scenario of 85% mask use with mandates. [file 41591_2020_1132_MOESM5_ESM.pdf]

Appendix 3:
SEIR Model Vetting Plots
Scenario: 85% Mask Use With Mandates

Contents

1 United States of America: SEIR fit comparison **7**

2 United States of America: Covariate fits and regression coefficients **8**

3 Alabama: SEIR fit comparison **9**

4 Alabama: Covariate fits and regression coefficients **10**

5 Alaska: SEIR fit comparison **11**

6 Alaska: Covariate fits and regression coefficients **12**

7 Arizona: SEIR fit comparison **13**

8 Arizona: Covariate fits and regression coefficients **14**

9 Arkansas: SEIR fit comparison **15**

10 Arkansas: Covariate fits and regression coefficients **16**

11 California: SEIR fit comparison **17**

12 California: Covariate fits and regression coefficients **18**

13 Colorado: SEIR fit comparison **19**

14 Colorado: Covariate fits and regression coefficients **20**

15 Connecticut: SEIR fit comparison **21**

16 Connecticut: Covariate fits and regression coefficients **22**

17 Delaware: SEIR fit comparison **23**

18 Delaware: Covariate fits and regression coefficients **24**

19 District of Columbia: SEIR fit comparison **25**

20 District of Columbia: Covariate fits and regression coefficients **26**

21 Florida: SEIR fit comparison **27**

22 Florida: Covariate fits and regression coefficients **28**

23 Georgia: SEIR fit comparison **29**

24 Georgia: Covariate fits and regression coefficients 30
25 Hawaii: SEIR fit comparison 31
26 Hawaii: Covariate fits and regression coefficients 32
27 Idaho: SEIR fit comparison 33
28 Idaho: Covariate fits and regression coefficients 34
29 Illinois: SEIR fit comparison 35
30 Illinois: Covariate fits and regression coefficients 36
31 Indiana: SEIR fit comparison 37
32 Indiana: Covariate fits and regression coefficients 38
33 Iowa: SEIR fit comparison 39
34 Iowa: Covariate fits and regression coefficients 40
35 Kansas: SEIR fit comparison 41
36 Kansas: Covariate fits and regression coefficients 42
37 Kentucky: SEIR fit comparison 43
38 Kentucky: Covariate fits and regression coefficients 44
39 Louisiana: SEIR fit comparison 45
40 Louisiana: Covariate fits and regression coefficients 46
41 Maine: SEIR fit comparison 47
42 Maine: Covariate fits and regression coefficients 48
43 Maryland: SEIR fit comparison 49
44 Maryland: Covariate fits and regression coefficients 50
45 Massachusetts: SEIR fit comparison 51
46 Massachusetts: Covariate fits and regression coefficients 52
47 Michigan: SEIR fit comparison 53
48 Michigan: Covariate fits and regression coefficients 54

49 Minnesota: SEIR fit comparison 55

50 Minnesota: Covariate fits and regression coefficients 56

51 Mississippi: SEIR fit comparison 57

52 Mississippi: Covariate fits and regression coefficients 58

53 Missouri: SEIR fit comparison 59

54 Missouri: Covariate fits and regression coefficients 60

55 Montana: SEIR fit comparison 61

56 Montana: Covariate fits and regression coefficients 62

57 Nebraska: SEIR fit comparison 63

58 Nebraska: Covariate fits and regression coefficients 64

59 Nevada: SEIR fit comparison 65

60 Nevada: Covariate fits and regression coefficients 66

61 New Hampshire: SEIR fit comparison 67

62 New Hampshire: Covariate fits and regression coefficients 68

63 New Jersey: SEIR fit comparison 69

64 New Jersey: Covariate fits and regression coefficients 70

65 New Mexico: SEIR fit comparison 71

66 New Mexico: Covariate fits and regression coefficients 72

67 New York: SEIR fit comparison 73

68 New York: Covariate fits and regression coefficients 74

69 North Carolina: SEIR fit comparison 75

70 North Carolina: Covariate fits and regression coefficients 76

71 North Dakota: SEIR fit comparison 77

72 North Dakota: Covariate fits and regression coefficients 78

73 Ohio: SEIR fit comparison 79

74 Ohio: Covariate fits and regression coefficients **80**

75 Oklahoma: SEIR fit comparison **81**

76 Oklahoma: Covariate fits and regression coefficients **82**

77 Oregon: SEIR fit comparison **83**

78 Oregon: Covariate fits and regression coefficients **84**

79 Pennsylvania: SEIR fit comparison **85**

80 Pennsylvania: Covariate fits and regression coefficients **86**

81 Rhode Island: SEIR fit comparison **87**

82 Rhode Island: Covariate fits and regression coefficients **88**

83 South Carolina: SEIR fit comparison **89**

84 South Carolina: Covariate fits and regression coefficients **90**

85 South Dakota: SEIR fit comparison **91**

86 South Dakota: Covariate fits and regression coefficients **92**

87 Tennessee: SEIR fit comparison **93**

88 Tennessee: Covariate fits and regression coefficients **94**

89 Texas: SEIR fit comparison **95**

90 Texas: Covariate fits and regression coefficients **96**

91 Utah: SEIR fit comparison **97**

92 Utah: Covariate fits and regression coefficients **98**

93 Vermont: SEIR fit comparison **99**

94 Vermont: Covariate fits and regression coefficients **100**

95 Virginia: SEIR fit comparison **101**

96 Virginia: Covariate fits and regression coefficients **102**

97 Washington: SEIR fit comparison **103**

98 Washington: Covariate fits and regression coefficients **104**

99 West Virginia: SEIR fit comparison 105

100 West Virginia: Covariate fits and regression coefficients 106

101 Wisconsin: SEIR fit comparison 107

102 Wisconsin: Covariate fits and regression coefficients 108

103 Wyoming: SEIR fit comparison 109

104 Wyoming: Covariate fits and regression coefficients 110

105 Spokane County: SEIR fit comparison 111

106 Spokane County: Covariate fits and regression coefficients 112

107 King and Snohomish Counties: SEIR fit comparison 113

108 King and Snohomish Counties: Covariate fits and regression coefficients 114

109 Washington except for King, Snohomish, and Spokane Counties: SEIR fit comparison 115

110 Washington except for King, Snohomish, and Spokane Counties: Covariate fits and regression coefficients 117

1 United States of America: SEIR fit comparison

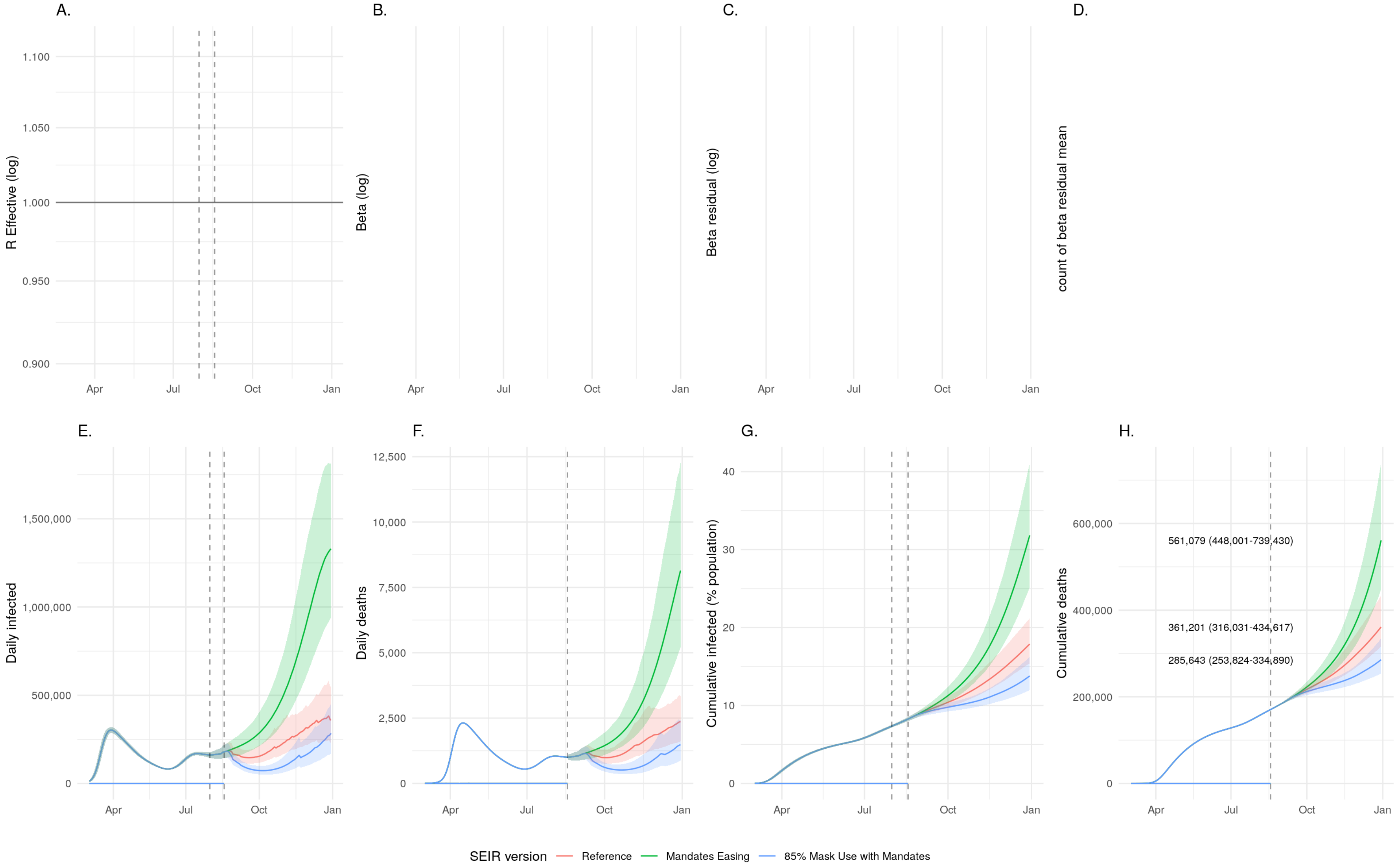

United States of America: SEIR fit comparison. Panels **A-D** display values that are not directly calculated for aggregate locations. **E**: predicted daily infections from each model through December 31. **F**: predicted daily deaths from each model through December 31. **G**: predicted cumulative infections through December 31, as a proportion of the total population. **H**: predicted cumulative deaths through December 31. In panels E, F, G, and H, reported death and infections are plotted alongside model predictions in light blue.

2 United States of America: Covariate fits and regression coefficients

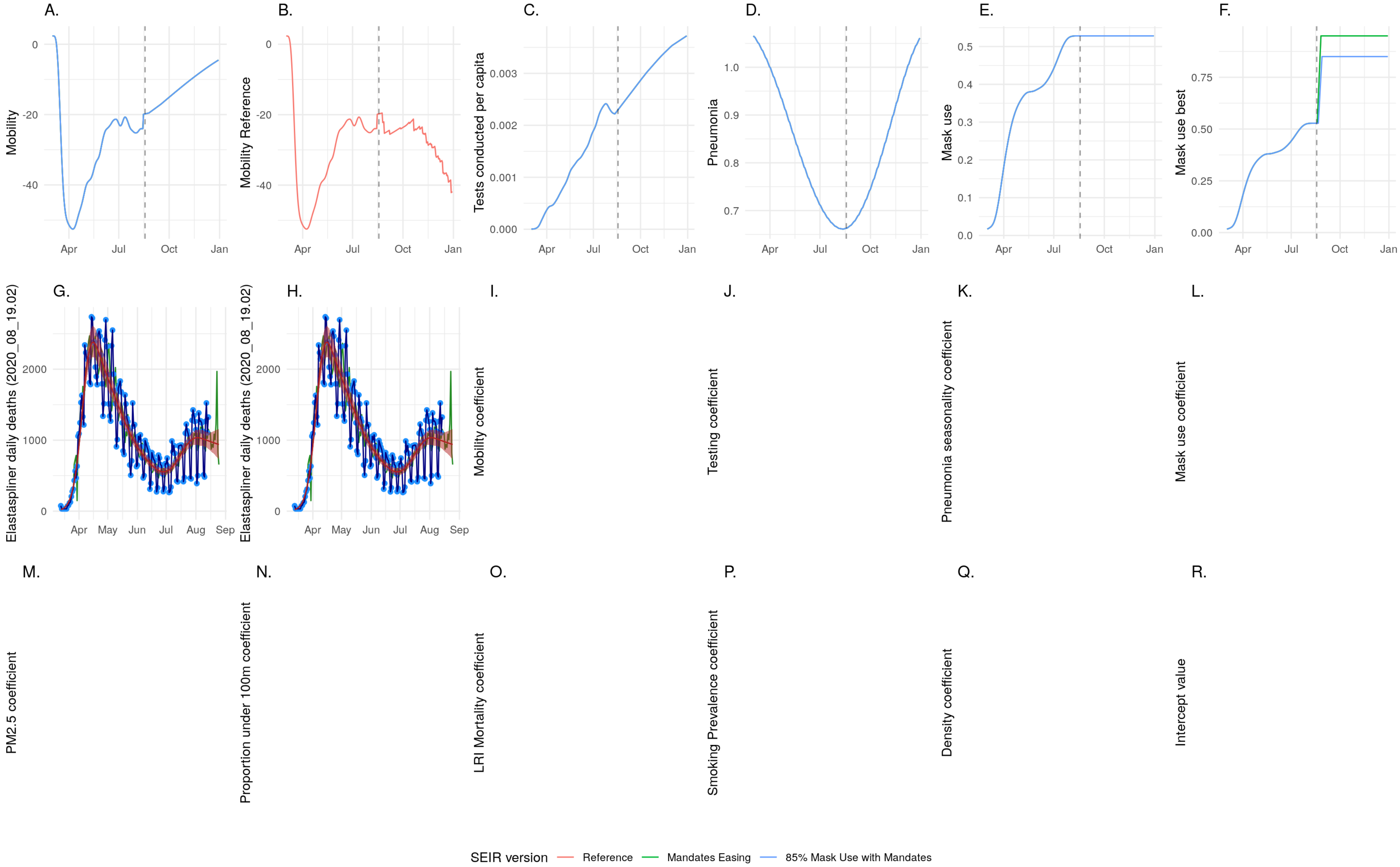

United States of America: Covariate fits and regression coefficients. A-F: Line plots showing predicted covariate time trends for **A)** mobility in the absence of additional mandates; **B)** mobility with additional mandates applied; **C)** diagnostic testing per capita; **D)** pneumonia seasonality; **E)** mask use per capita, and; **F)** mask use in a scenario where adherence increases to 85% of the population. **G-H**: COVID mortality data generated from reported daily deaths (blue); estimated based on reported hospitalizations (purple); estimated from reported cases (green); and via a spline fit through all available data types (red, 95% UI in pink). Panels **I-R** display coefficients for a regression fit to $log(beta)$, which is not applicable to aggregate locations.

3 Alabama: SEIR fit comparison

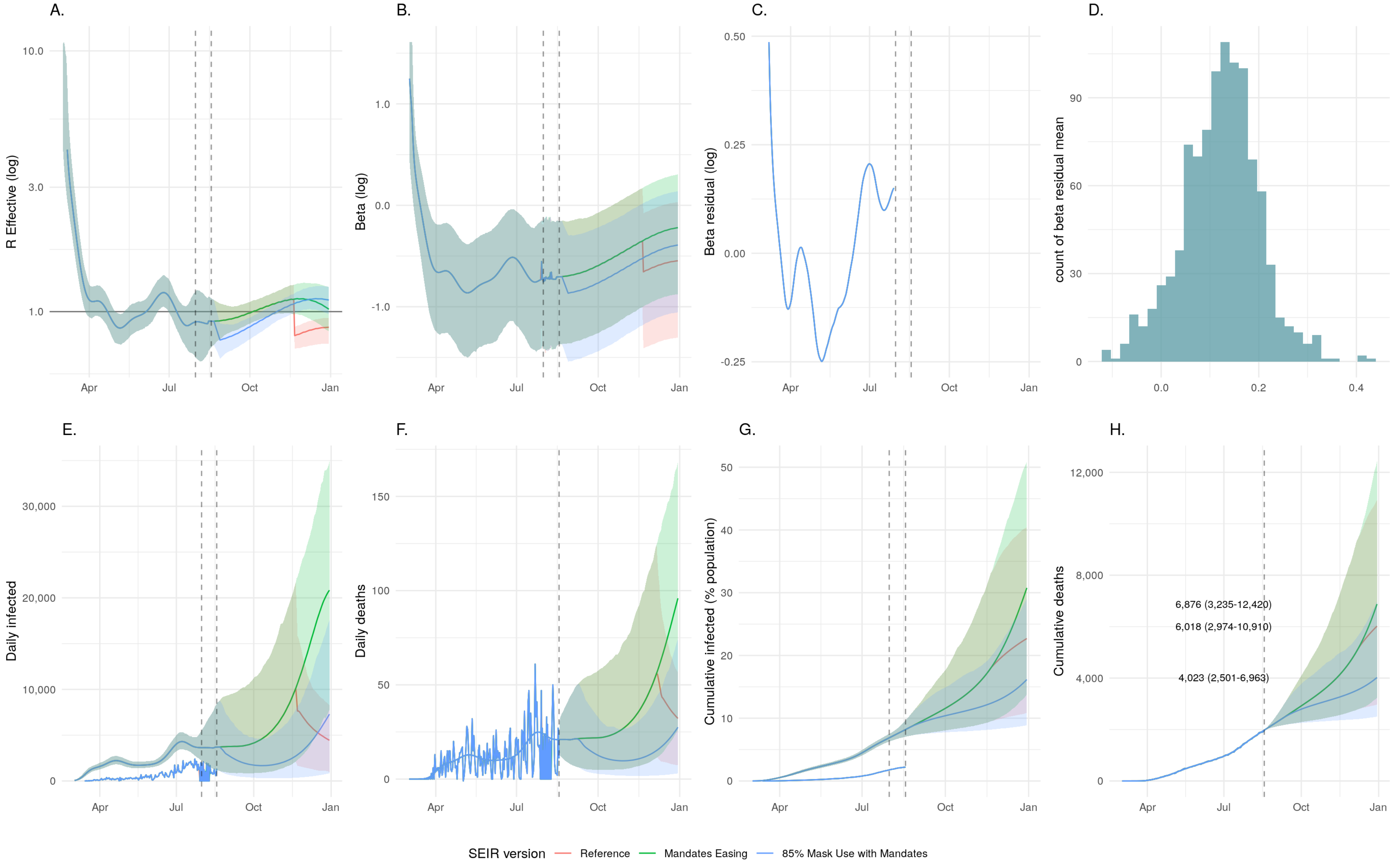

Alabama: SEIR fit comparison. **A**: predicted R effective for each model through December 31. **B**: predicted SEIR *beta* parameter. **C**: residual of predicted *beta* and the observed value calculated directly from infection data over time. **D**: histogram of residual values for *beta*. Panels A, B, C, and D are all displayed in log space, reflecting the space in which the SEIR model is fit. **E**: predicted daily infections from each model through December 31. **F**: predicted daily deaths from each model through December 31. **G**: predicted cumulative infections through December 31, as a proportion of the total population. **H**: predicted cumulative deaths through December 31. In panels E, F, G, and H, reported death and infections are plotted alongside model predictions in light blue.

4 Alabama: Covariate fits and regression coefficients

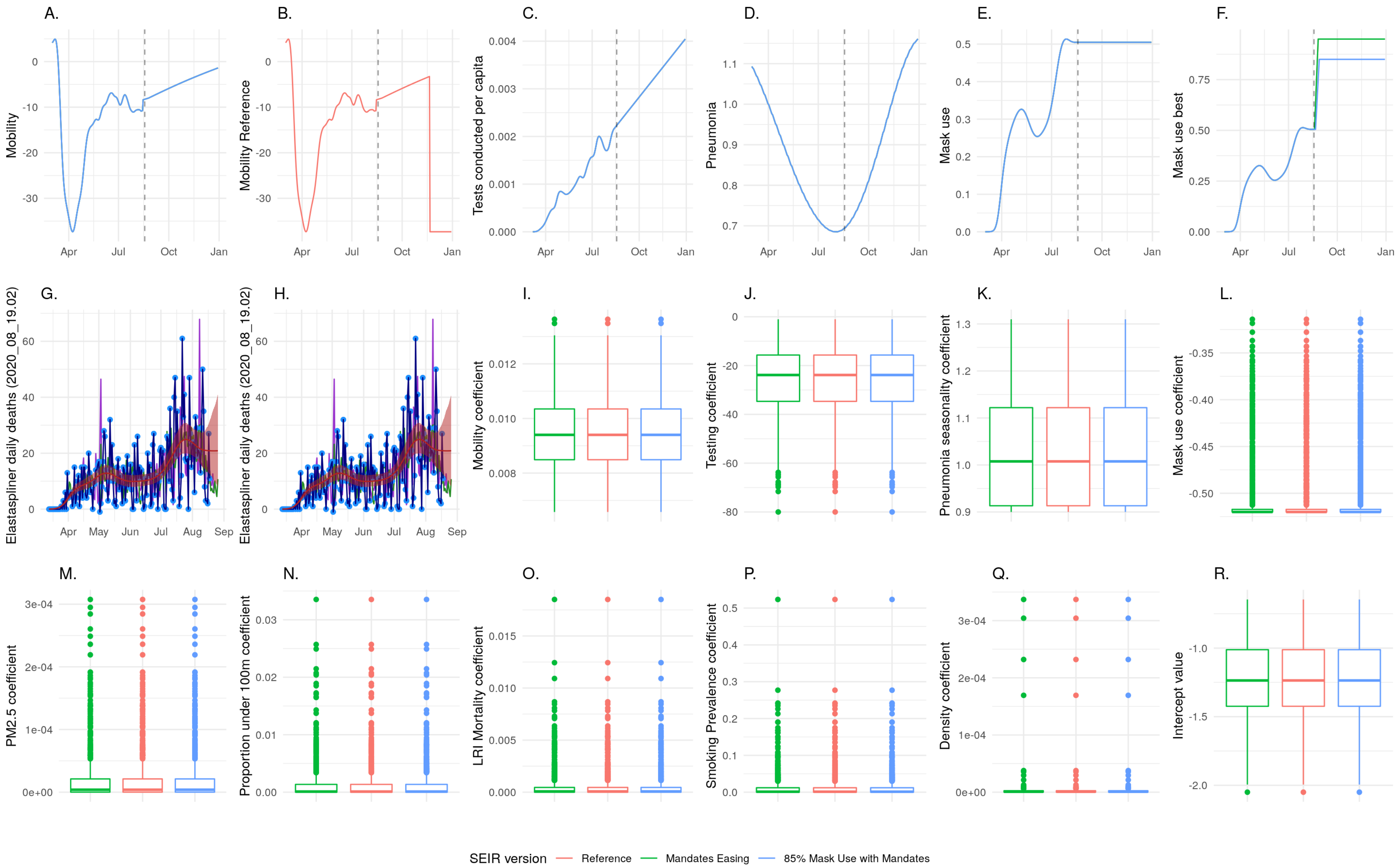

Alabama: Covariate fits and regression coefficients. A-F: Line plots showing predicted covariate time trends for **A)** mobility in the absence of additional mandates; **B)** mobility with additional mandates applied; **C)** diagnostic testing per capita; **D)** pneumonia seasonality; **E)** mask use per capita, and; **F)** mask use in a scenario where adherence increases to 85% of the population. **G-H**: COVID mortality data generated from reported daily deaths (blue); estimated based on reported hospitalizations (purple); estimated from reported cases (green); and via a spline fit through all available data types (red, 95% UI in pink). **I-R**: Box plots showing 1,000 draws of fixed effect coefficients in a multivariate regression fit to $log(beta)$.

5 Alaska: SEIR fit comparison

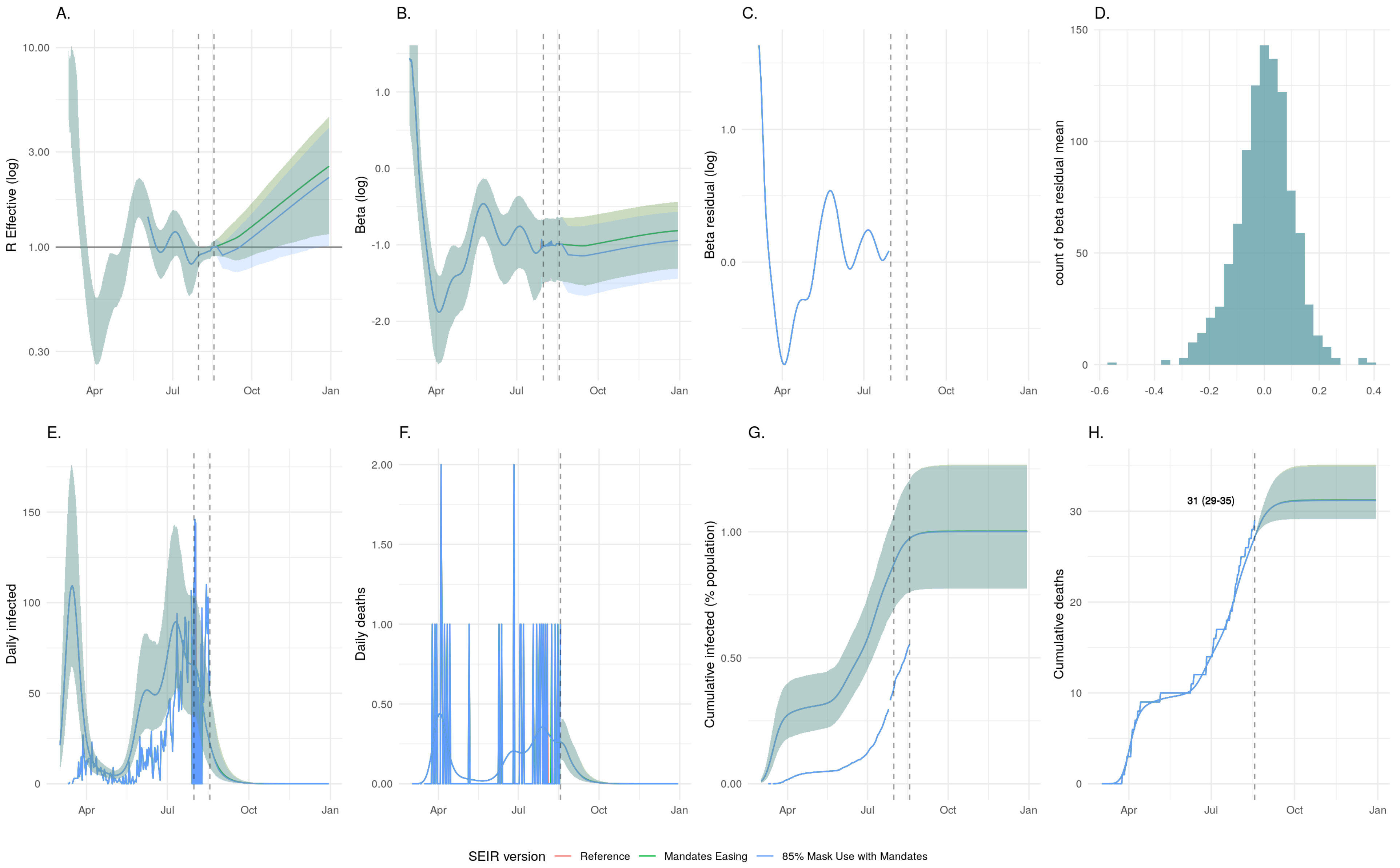

Alaska: SEIR fit comparison. **A**: predicted R effective for each model through December 31. **B**: predicted SEIR *beta* parameter. **C**: residual of predicted *beta* and the observed value calculated directly from infection data over time. **D**: histogram of residual values for *beta*. Panels A, B, C, and D are all displayed in log space, reflecting the space in which the SEIR model is fit. **E**: predicted daily infections from each model through December 31. **F**: predicted daily deaths from each model through December 31. **G**: predicted cumulative infections through December 31, as a proportion of the total population. **H**: predicted cumulative deaths through December 31. In panels E, F, G, and H, reported death and infections are plotted alongside model predictions in light blue.

6 Alaska: Covariate fits and regression coefficients

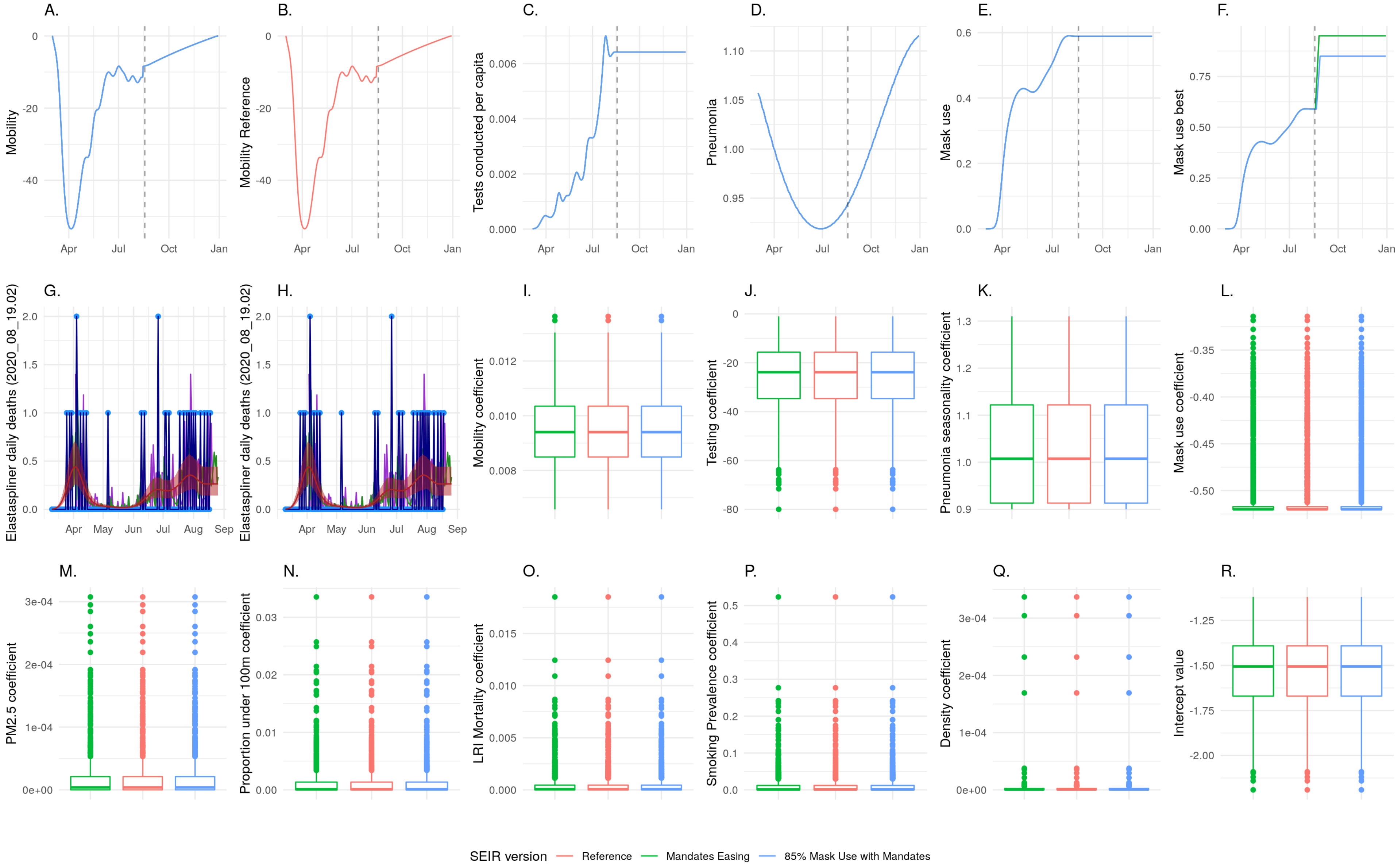

Alaska: Covariate fits and regression coefficients. A-F: Line plots showing predicted covariate time trends for **A)** mobility in the absence of additional mandates; **B)** mobility with additional mandates applied; **C)** diagnostic testing per capita; **D)** pneumonia seasonality; **E)** mask use per capita, and; **F)** mask use in a scenario where adherence increases to 85% of the population. **G-H**: COVID mortality data generated from reported daily deaths (blue); estimated based on reported hospitalizations (purple); estimated from reported cases (green); and via a spline fit through all available data types (red, 95% UI in pink). **I-R**: Box plots showing 1,000 draws of fixed effect coefficients in a multivariate regression fit to $log(beta)$.

7 Arizona: SEIR fit comparison

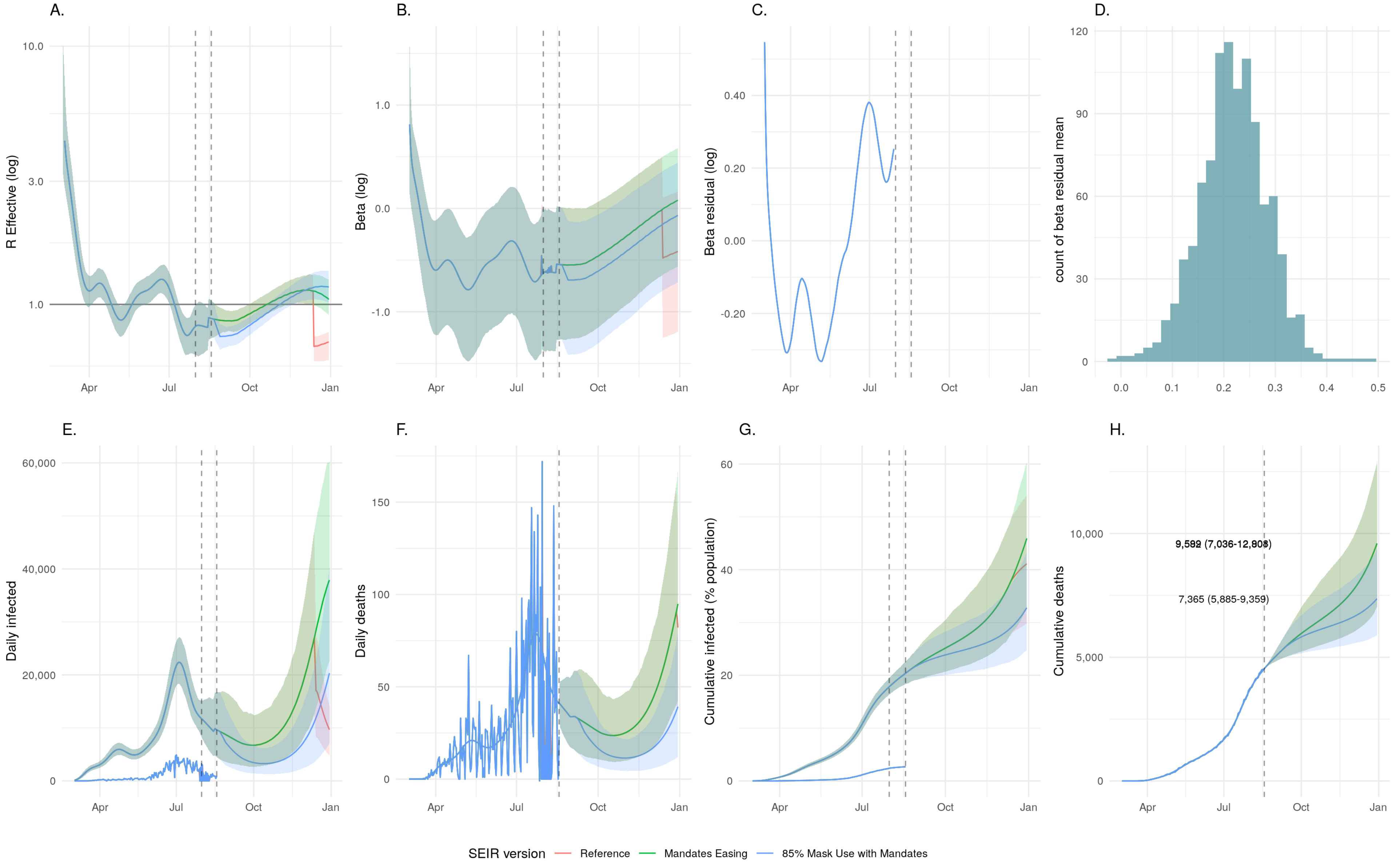

Arizona: SEIR fit comparison. A: predicted R effective for each model through December 31. **B**: predicted SEIR *beta* parameter. **C**: residual of predicted *beta* and the observed value calculated directly from infection data over time. **D**: histogram of residual values for *beta*. Panels A, B, C, and D are all displayed in log space, reflecting the space in which the SEIR model is fit. **E**: predicted daily infections from each model through December 31. **F**: predicted daily deaths from each model through December 31. **G**: predicted cumulative infections through December 31, as a proportion of the total population. **H**: predicted cumulative deaths through December 31. In panels E, F, G, and H, reported death and infections are plotted alongside model predictions in light blue.

8 Arizona: Covariate fits and regression coefficients

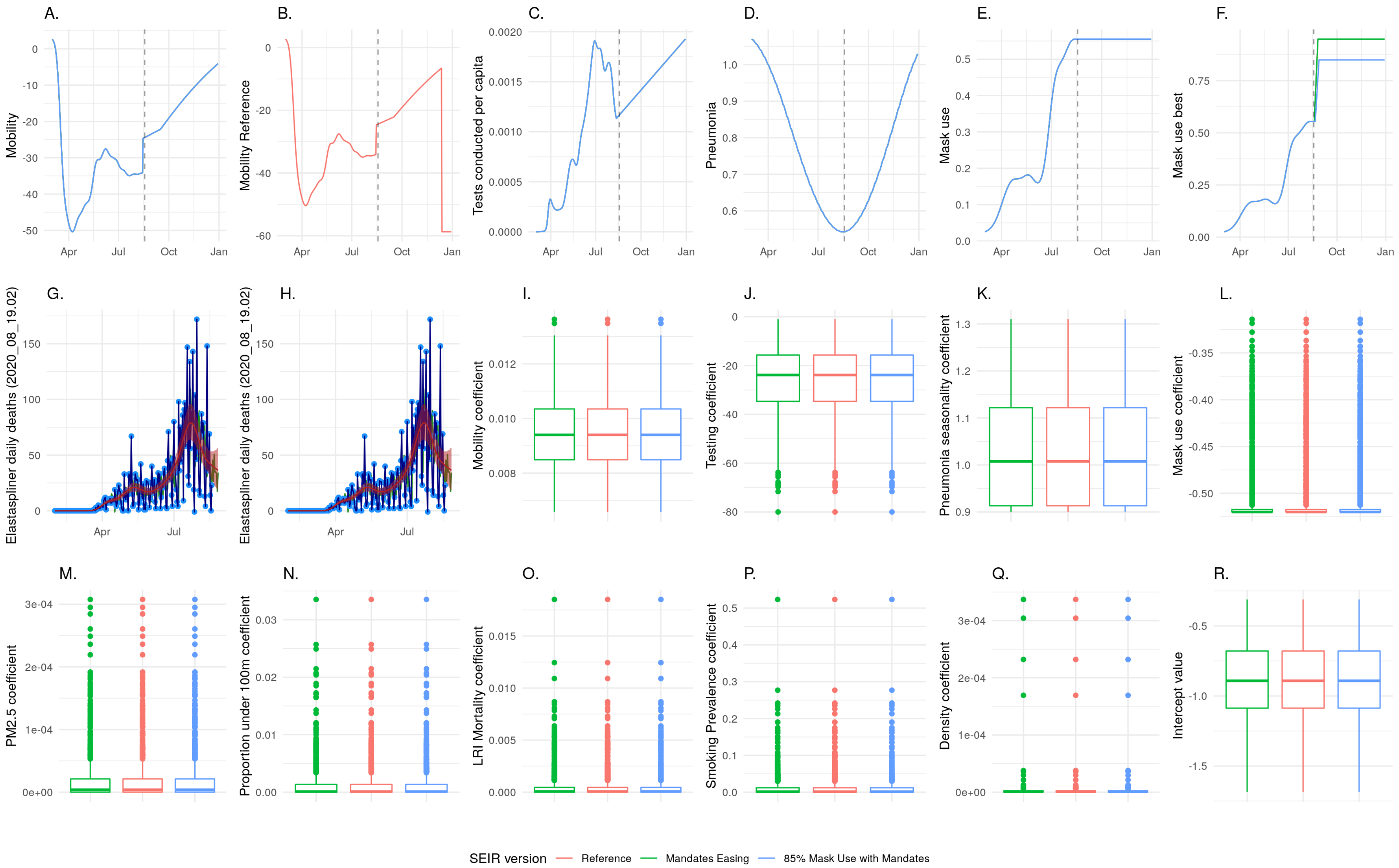

Arizona: Covariate fits and regression coefficients. A-F: Line plots showing predicted covariate time trends for **A)** mobility in the absence of additional mandates; **B)** mobility with additional mandates applied; **C)** diagnostic testing per capita; **D)** pneumonia seasonality; **E)** mask use per capita, and; **F)** mask use in a scenario where adherence increases to 85% of the population. **G-H**: COVID mortality data generated from reported daily deaths (blue); estimated based on reported hospitalizations (purple); estimated from reported cases (green); and via a spline fit through all available data types (red, 95% UI in pink). **I-R**: Box plots showing 1,000 draws of fixed effect coefficients in a multivariate regression fit to $log(beta)$.

9 Arkansas: SEIR fit comparison

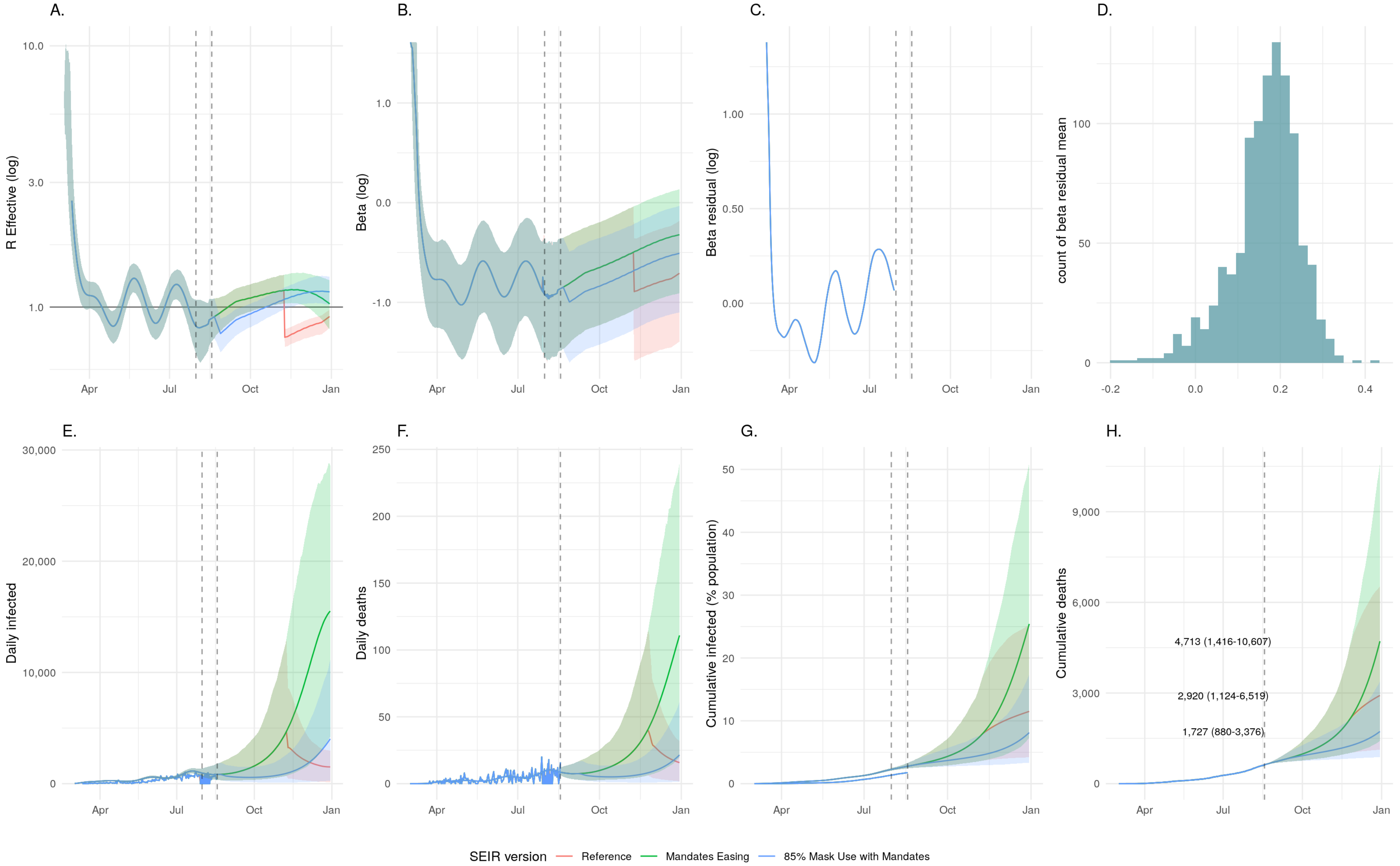

Arkansas: SEIR fit comparison. A: predicted R effective for each model through December 31. **B**: predicted SEIR *beta* parameter. **C**: residual of predicted *beta* and the observed value calculated directly from infection data over time. **D**: histogram of residual values for *beta*. Panels A, B, C, and D are all displayed in log space, reflecting the space in which the SEIR model is fit. **E**: predicted daily infections from each model through December 31. **F**: predicted daily deaths from each model through December 31. **G**: predicted cumulative infections through December 31, as a proportion of the total population. **H**: predicted cumulative deaths through December 31. In panels E, F, G, and H, reported death and infections are plotted alongside model predictions in light blue.

10 Arkansas: Covariate fits and regression coefficients

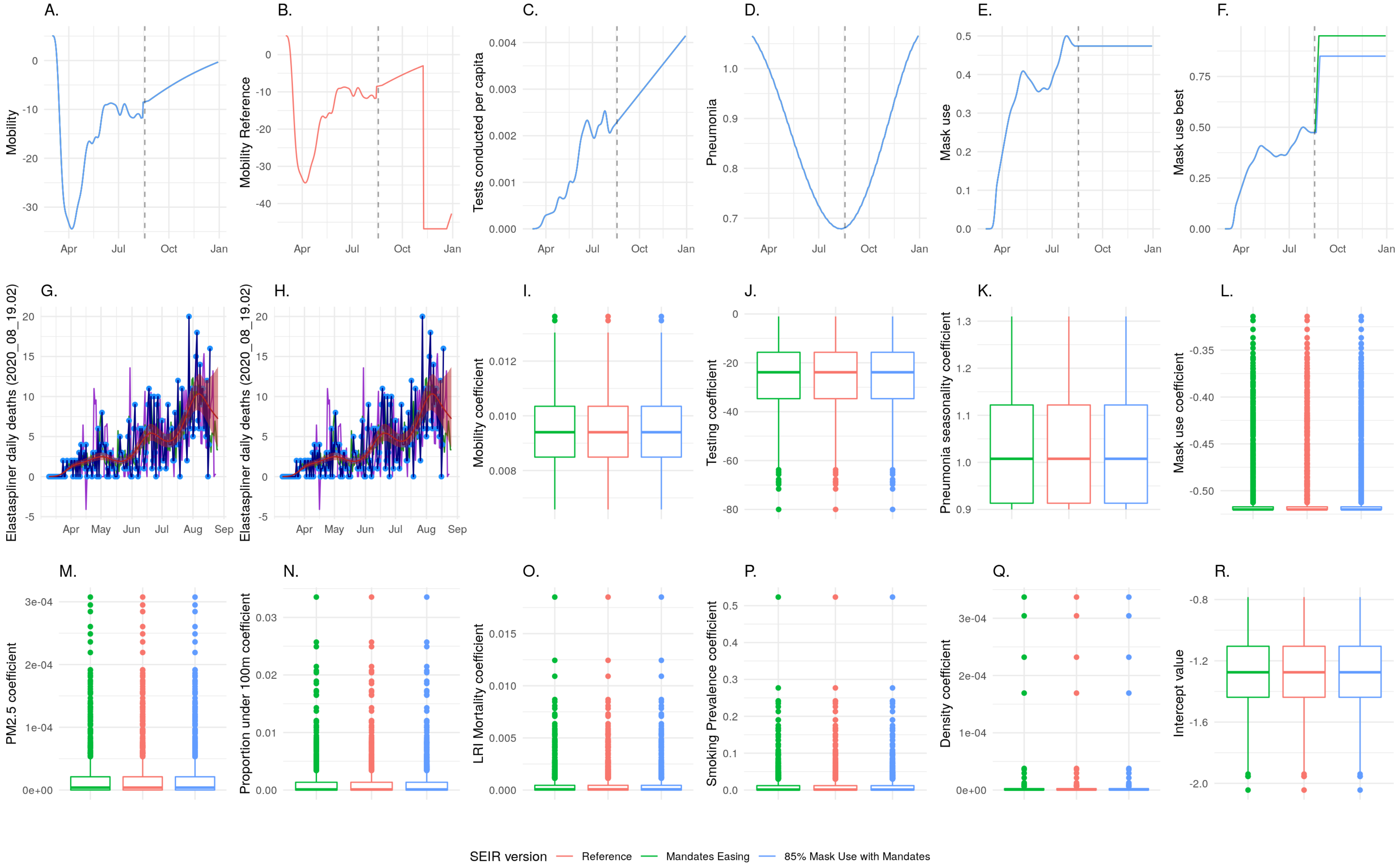

Arkansas: Covariate fits and regression coefficients. **A-F**: Line plots showing predicted covariate time trends for **A)** mobility in the absence of additional mandates; **B)** mobility with additional mandates applied; **C)** diagnostic testing per capita; **D)** pneumonia seasonality; **E)** mask use per capita, and; **F)** mask use in a scenario where adherence increases to 85% of the population. **G-H**: COVID mortality data generated from reported daily deaths (blue); estimated based on reported hospitalizations (purple); estimated from reported cases (green); and via a spline fit through all available data types (red, 95% UI in pink). **I-R**: Box plots showing 1,000 draws of fixed effect coefficients in a multivariate regression fit to $log(beta)$.

11 California: SEIR fit comparison

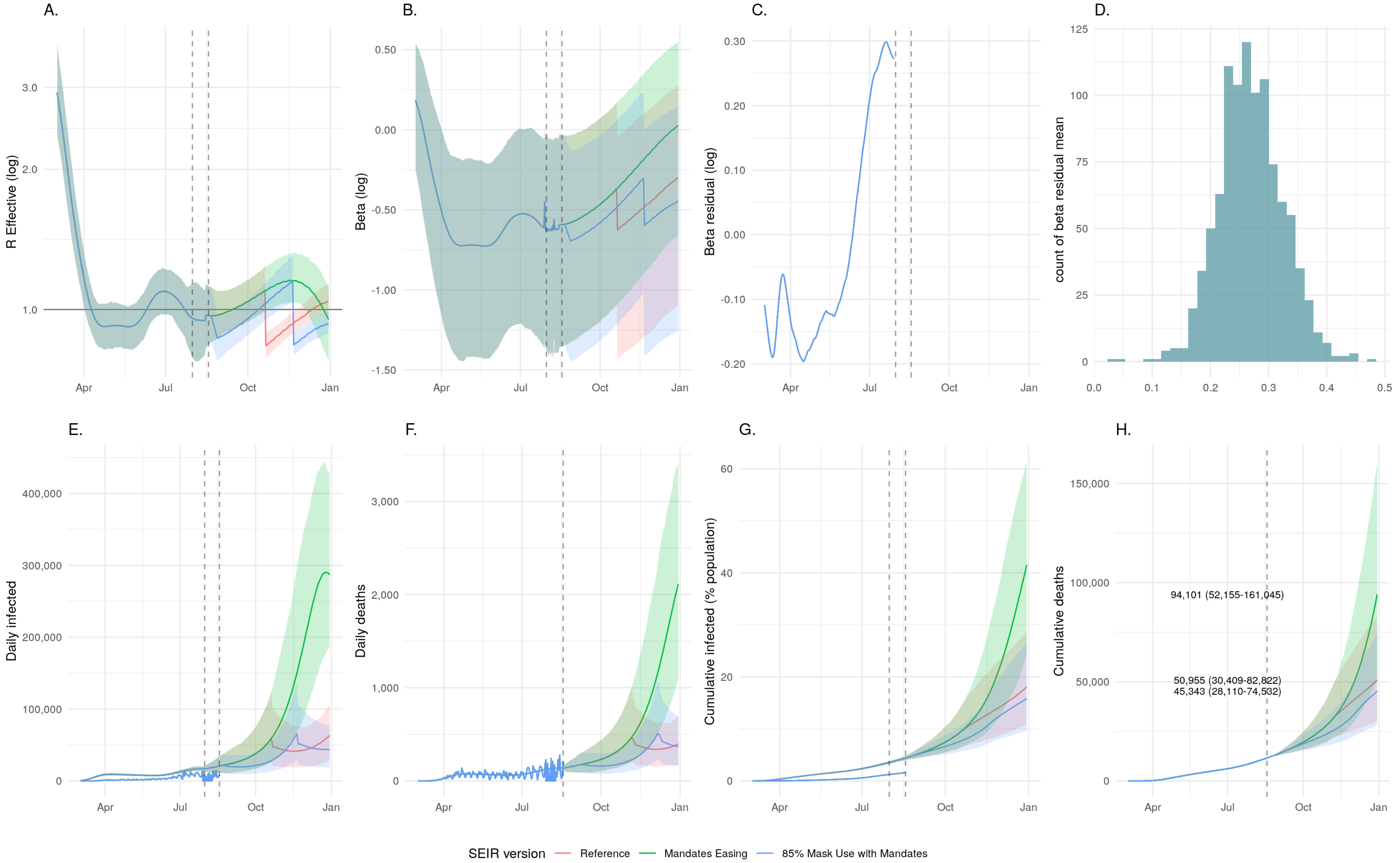

California: SEIR fit comparison. **A**: predicted R effective for each model through December 31. **B**: predicted SEIR *beta* parameter. **C**: residual of predicted *beta* and the observed value calculated directly from infection data over time. **D**: histogram of residual values for *beta*. Panels A, B, C, and D are all displayed in log space, reflecting the space in which the SEIR model is fit. **E**: predicted daily infections from each model through December 31. **F**: predicted daily deaths from each model through December 31. **G**: predicted cumulative infections through December 31, as a proportion of the total population. **H**: predicted cumulative deaths through December 31. In panels E, F, G, and H, reported death and infections are plotted alongside model predictions in light blue.

12 California: Covariate fits and regression coefficients

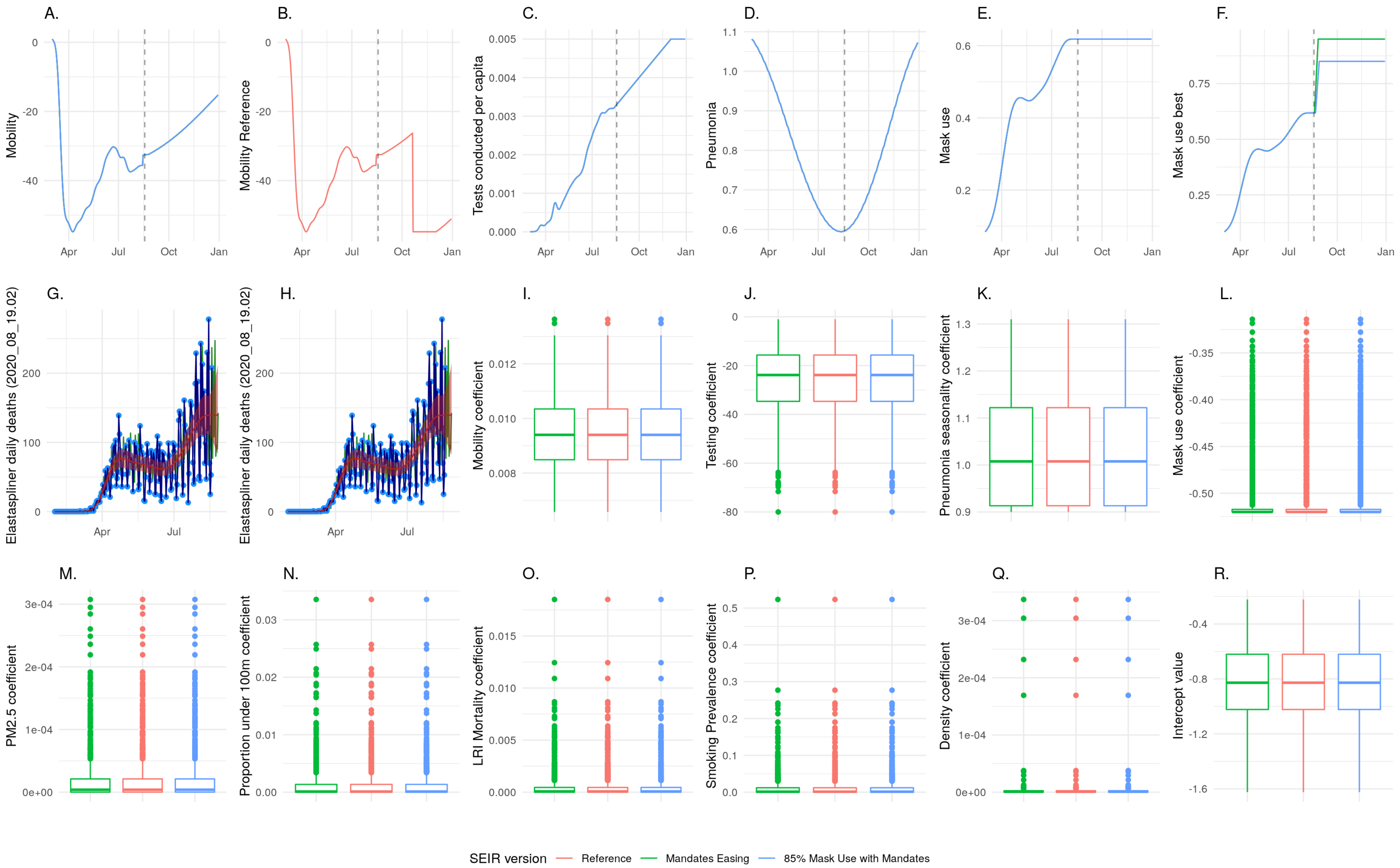

California: Covariate fits and regression coefficients. **A-F**: Line plots showing predicted covariate time trends for **A)** mobility in the absence of additional mandates; **B)** mobility with additional mandates applied; **C)** diagnostic testing per capita; **D)** pneumonia seasonality; **E)** mask use per capita, and; **F)** mask use in a scenario where adherence increases to 85% of the population. **G-H**: COVID mortality data generated from reported daily deaths (blue); estimated based on reported hospitalizations (purple); estimated from reported cases (green); and via a spline fit through all available data types (red, 95% UI in pink). **I-R**: Box plots showing 1,000 draws of fixed effect coefficients in a multivariate regression fit to $log(beta)$.

13 Colorado: SEIR fit comparison

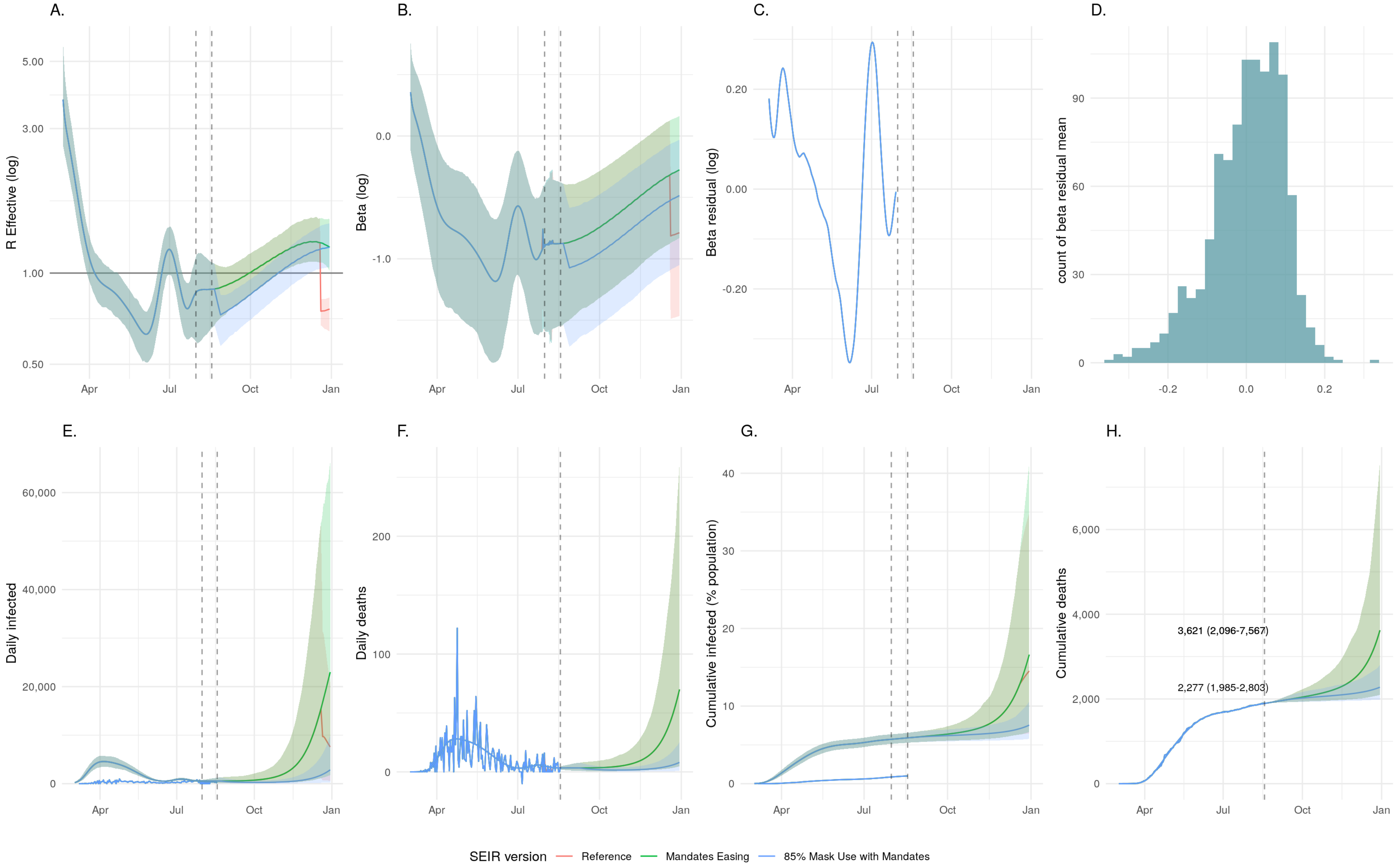

Colorado: SEIR fit comparison. A: predicted R effective for each model through December 31. **B**: predicted SEIR *beta* parameter. **C**: residual of predicted *beta* and the observed value calculated directly from infection data over time. **D**: histogram of residual values for *beta*. Panels A, B, C, and D are all displayed in log space, reflecting the space in which the SEIR model is fit. **E**: predicted daily infections from each model through December 31. **F**: predicted daily deaths from each model through December 31. **G**: predicted cumulative infections through December 31, as a proportion of the total population. **H**: predicted cumulative deaths through December 31. In panels E, F, G, and H, reported death and infections are plotted alongside model predictions in light blue.

14 Colorado: Covariate fits and regression coefficients

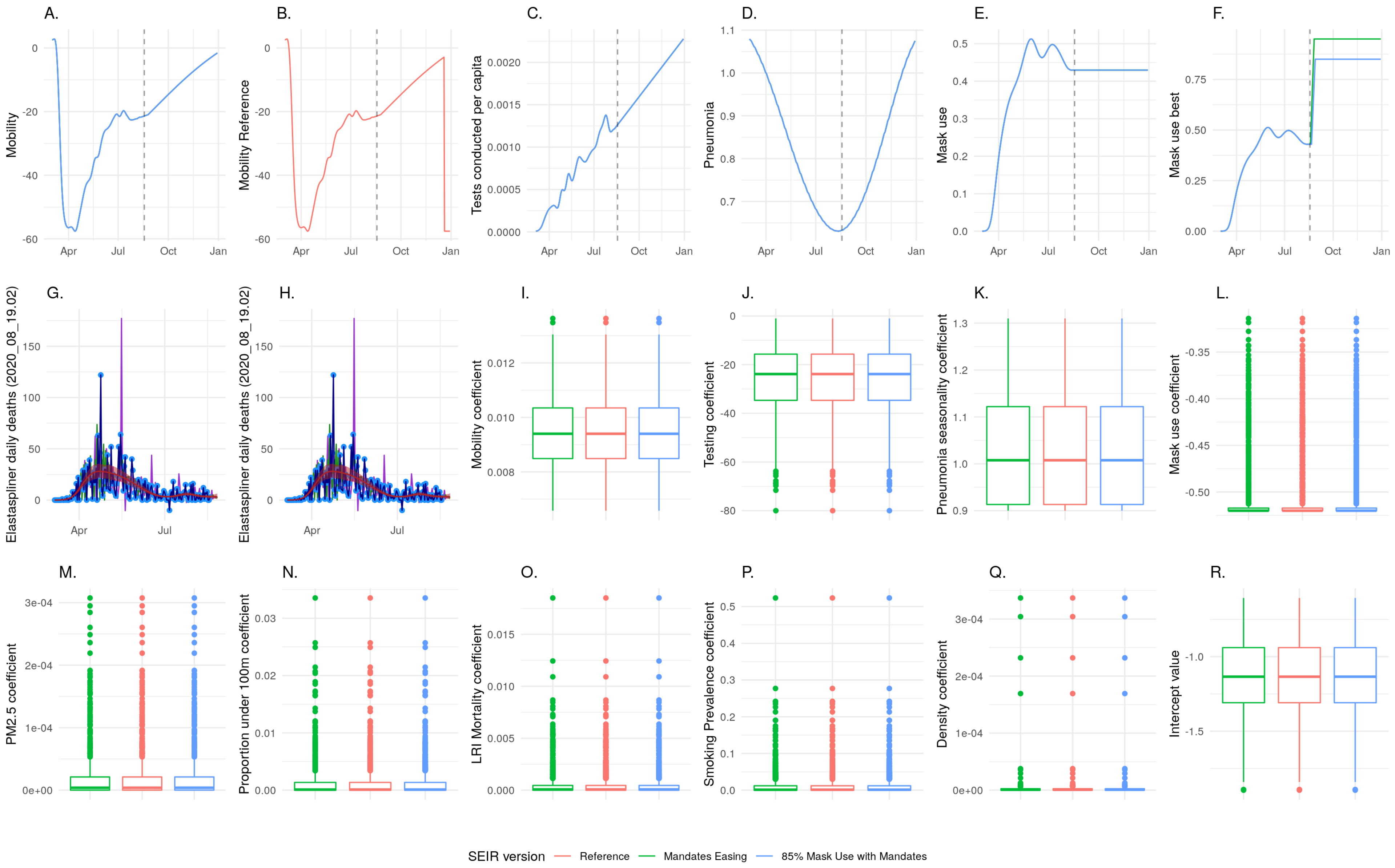

Colorado: Covariate fits and regression coefficients. A-F: Line plots showing predicted covariate time trends for **A)** mobility in the absence of additional mandates; **B)** mobility with additional mandates applied; **C)** diagnostic testing per capita; **D)** pneumonia seasonality; **E)** mask use per capita, and; **F)** mask use in a scenario where adherence increases to 85% of the population. **G-H**: COVID mortality data generated from reported daily deaths (blue); estimated based on reported hospitalizations (purple); estimated from reported cases (green); and via a spline fit through all available data types (red, 95% UI in pink). **I-R**: Box plots showing 1,000 draws of fixed effect coefficients in a multivariate regression fit to $log(beta)$.

15 Connecticut: SEIR fit comparison

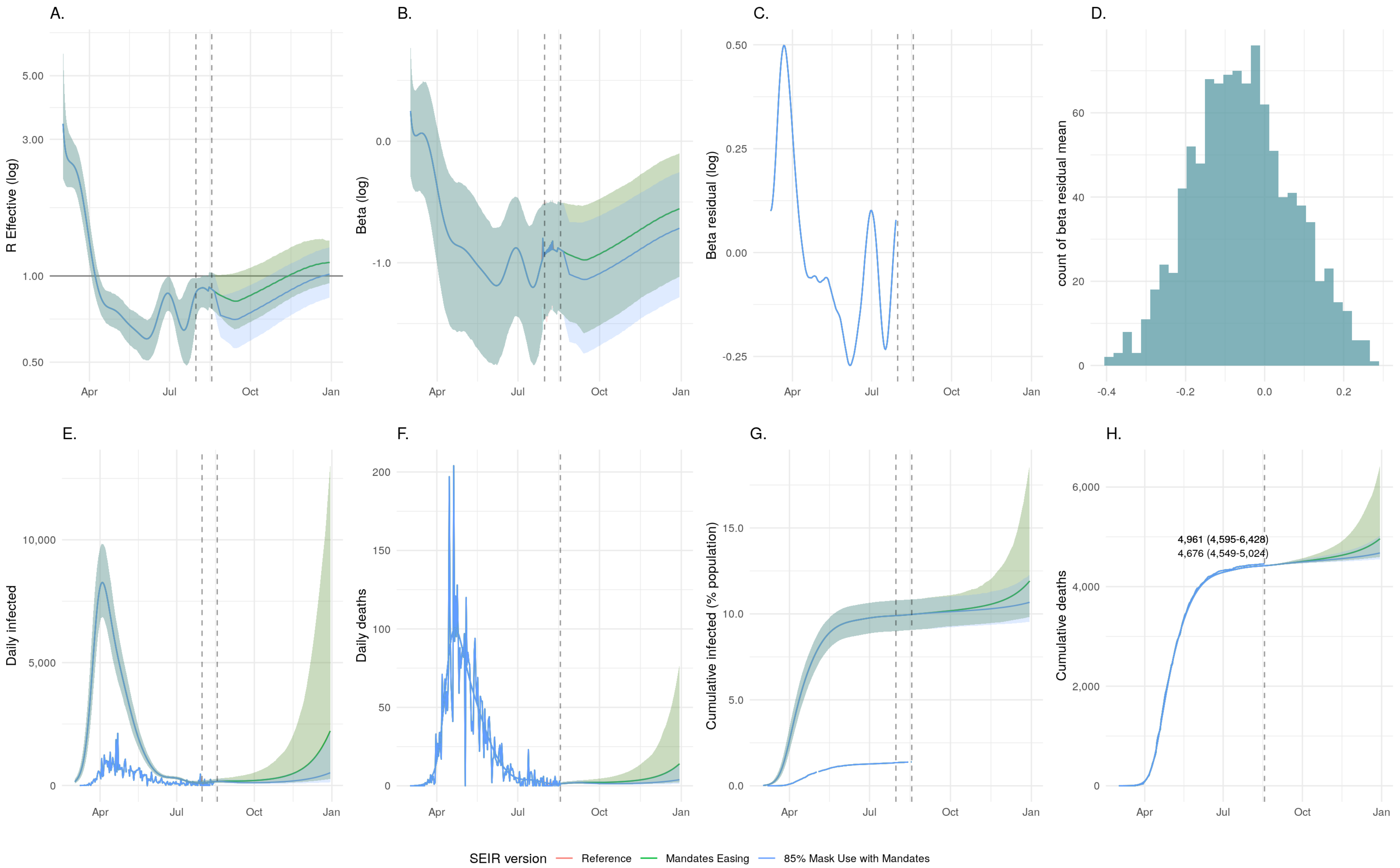

Connecticut: SEIR fit comparison. **A**: predicted R effective for each model through December 31. **B**: predicted SEIR *beta* parameter. **C**: residual of predicted *beta* and the observed value calculated directly from infection data over time. **D**: histogram of residual values for *beta*. Panels A, B, C, and D are all displayed in log space, reflecting the space in which the SEIR model is fit. **E**: predicted daily infections from each model through December 31. **F**: predicted daily deaths from each model through December 31. **G**: predicted cumulative infections through December 31, as a proportion of the total population. **H**: predicted cumulative deaths through December 31. In panels E, F, G, and H, reported death and infections are plotted alongside model predictions in light blue.

16 Connecticut: Covariate fits and regression coefficients

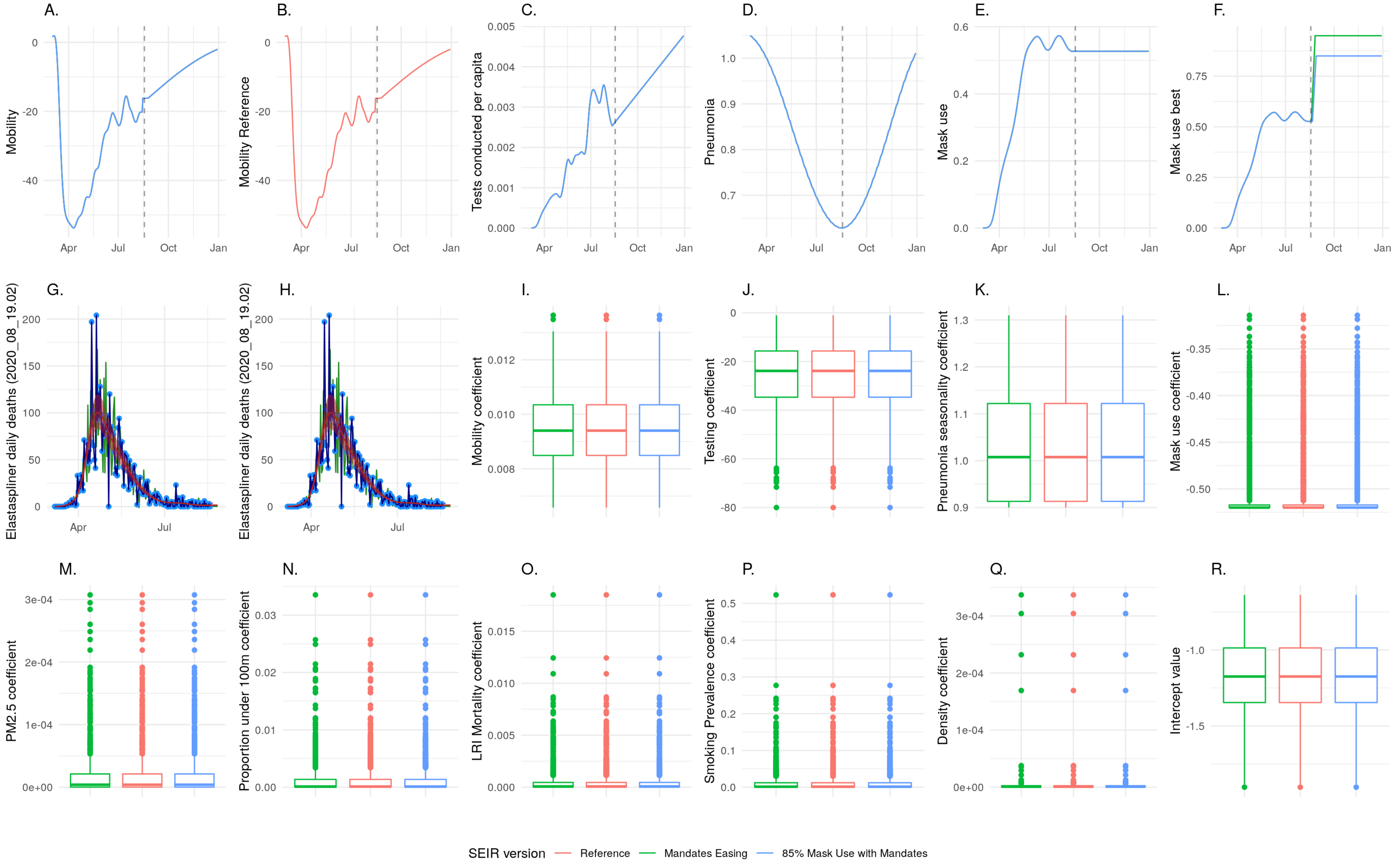

Connecticut: Covariate fits and regression coefficients. **A-F**: Line plots showing predicted covariate time trends for **A)** mobility in the absence of additional mandates; **B)** mobility with additional mandates applied; **C)** diagnostic testing per capita; **D)** pneumonia seasonality; **E)** mask use per capita, and; **F)** mask use in a scenario where adherence increases to 85% of the population. **G-H**: COVID mortality data generated from reported daily deaths (blue); estimated based on reported hospitalizations (purple); estimated from reported cases (green); and via a spline fit through all available data types (red, 95% UI in pink). **I-R**: Box plots showing 1,000 draws of fixed effect coefficients in a multivariate regression fit to $log(beta)$.

17 Delaware: SEIR fit comparison

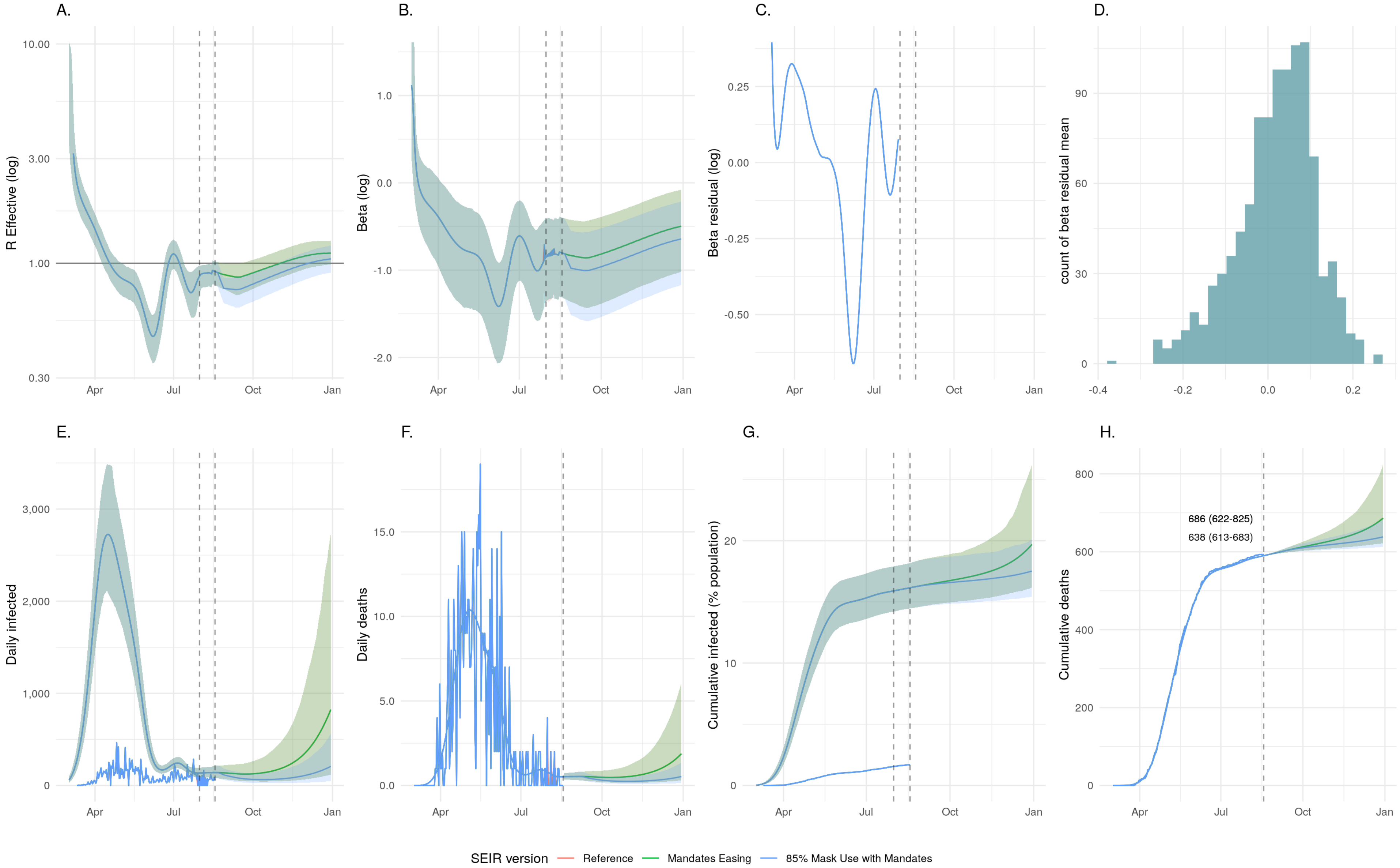

Delaware: SEIR fit comparison. A: predicted R effective for each model through December 31. **B**: predicted SEIR *beta* parameter. **C**: residual of predicted *beta* and the observed value calculated directly from infection data over time. **D**: histogram of residual values for *beta*. Panels A, B, C, and D are all displayed in log space, reflecting the space in which the SEIR model is fit. **E**: predicted daily infections from each model through December 31. **F**: predicted daily deaths from each model through December 31. **G**: predicted cumulative infections through December 31, as a proportion of the total population. **H**: predicted cumulative deaths through December 31. In panels E, F, G, and H, reported death and infections are plotted alongside model predictions in light blue.

18 Delaware: Covariate fits and regression coefficients

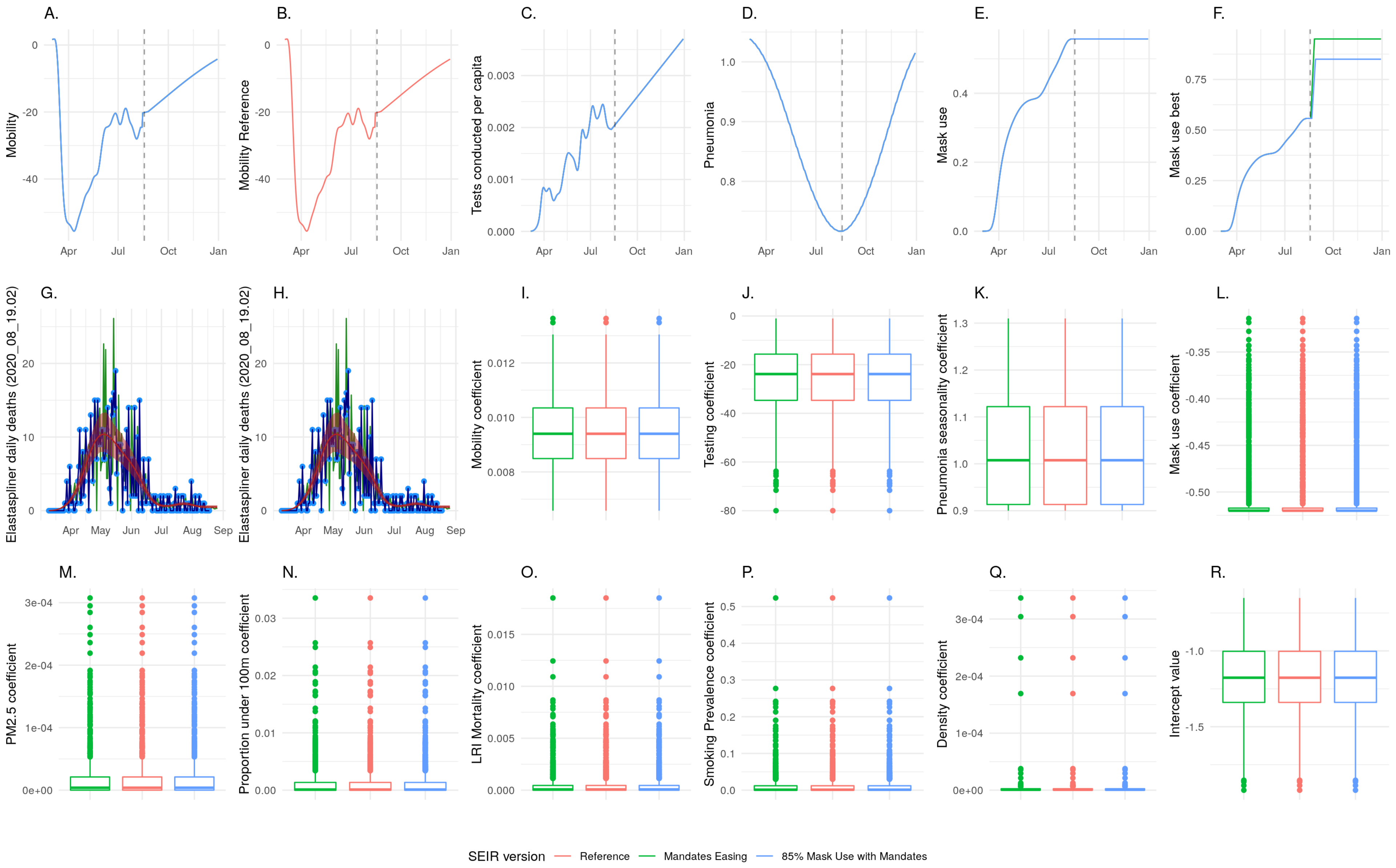

Delaware: Covariate fits and regression coefficients. **A-F**: Line plots showing predicted covariate time trends for **A)** mobility in the absence of additional mandates; **B)** mobility with additional mandates applied; **C)** diagnostic testing per capita; **D)** pneumonia seasonality; **E)** mask use per capita, and; **F)** mask use in a scenario where adherence increases to 85% of the population. **G-H**: COVID mortality data generated from reported daily deaths (blue); estimated based on reported hospitalizations (purple); estimated from reported cases (green); and via a spline fit through all available data types (red, 95% UI in pink). **I-R**: Box plots showing 1,000 draws of fixed effect coefficients in a multivariate regression fit to $log(beta)$.

19 District of Columbia: SEIR fit comparison

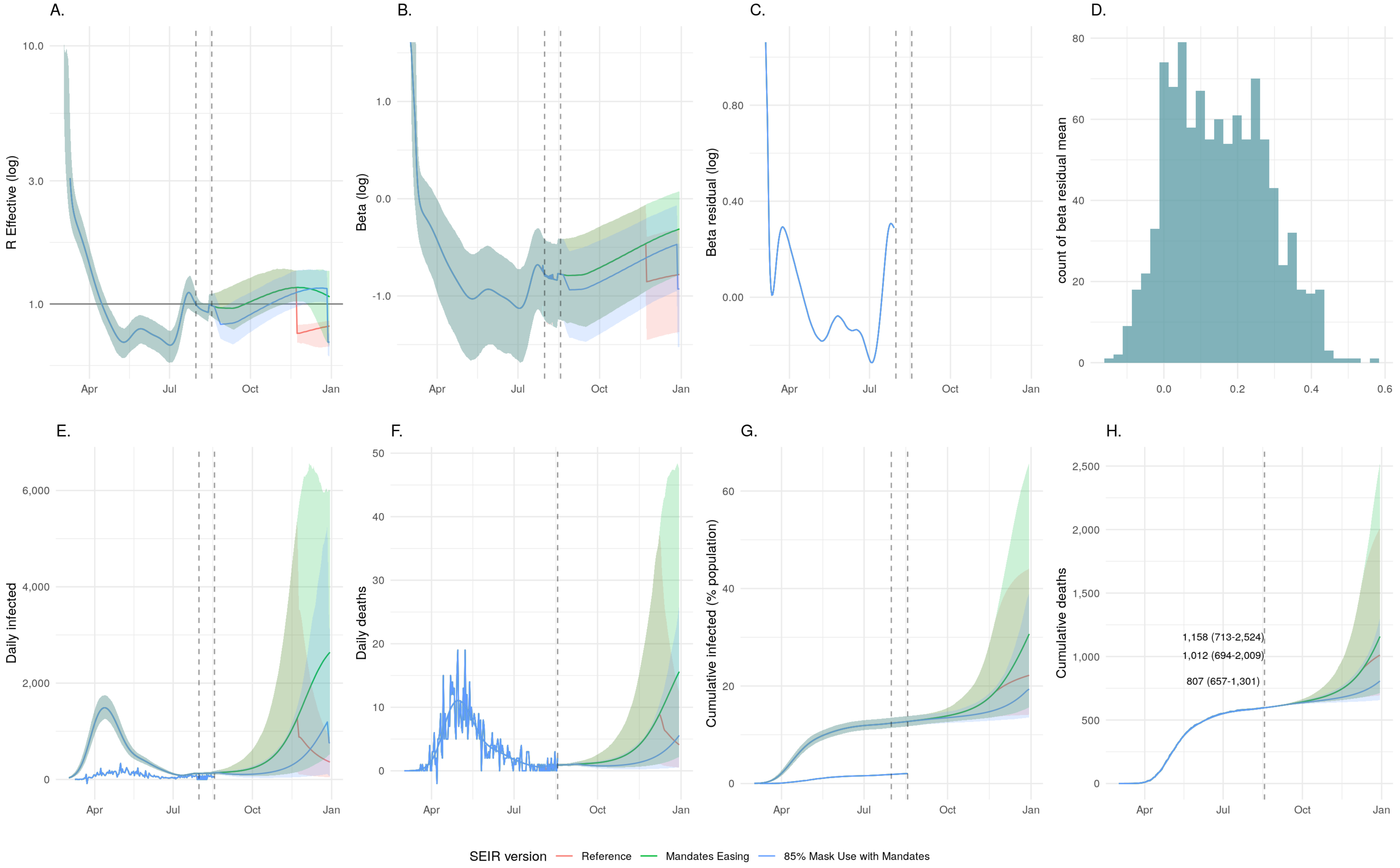

District of Columbia: SEIR fit comparison. **A**: predicted R effective for each model through December 31. **B**: predicted SEIR *beta* parameter. **C**: residual of predicted *beta* and the observed value calculated directly from infection data over time. **D**: histogram of residual values for *beta*. Panels A, B, C, and D are all displayed in log space, reflecting the space in which the SEIR model is fit. **E**: predicted daily infections from each model through December 31. **F**: predicted daily deaths from each model through December 31. **G**: predicted cumulative infections through December 31, as a proportion of the total population. **H**: predicted cumulative deaths through December 31. In panels E, F, G, and H, reported death and infections are plotted alongside model predictions in light blue.

20 District of Columbia: Covariate fits and regression coefficients

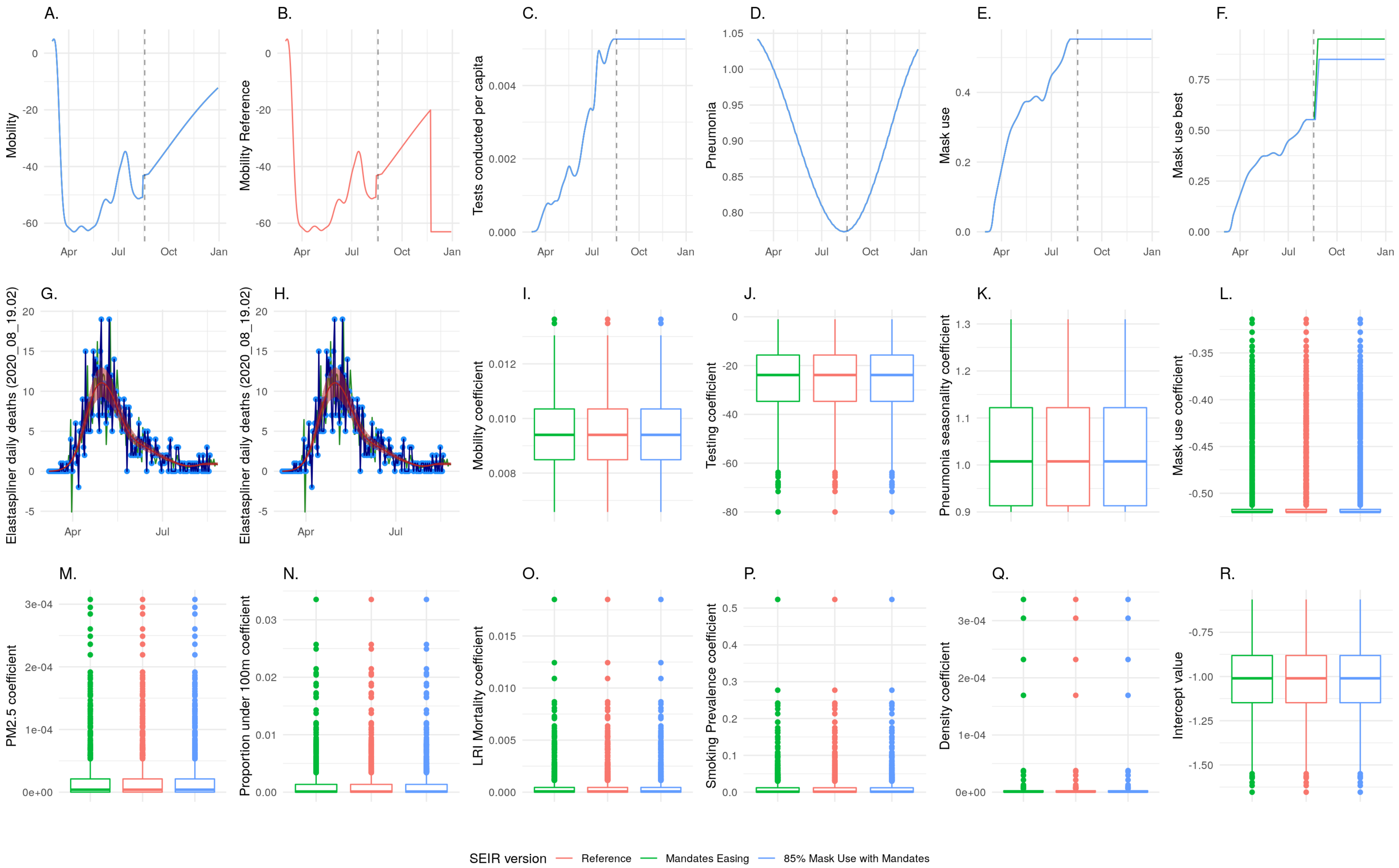

District of Columbia: Covariate fits and regression coefficients. A-F: Line plots showing predicted covariate time trends for **A)** mobility in the absence of additional mandates; **B)** mobility with additional mandates applied; **C)** diagnostic testing per capita; **D)** pneumonia seasonality; **E)** mask use per capita, and; **F)** mask use in a scenario where adherence increases to 85% of the population. **G-H**: COVID mortality data generated from reported daily deaths (blue); estimated based on reported hospitalizations (purple); estimated from reported cases (green); and via a spline fit through all available data types (red, 95% UI in pink). **I-R**: Box plots showing 1,000 draws of fixed effect coefficients in a multivariate regression fit to $log(beta)$.

21 Florida: SEIR fit comparison

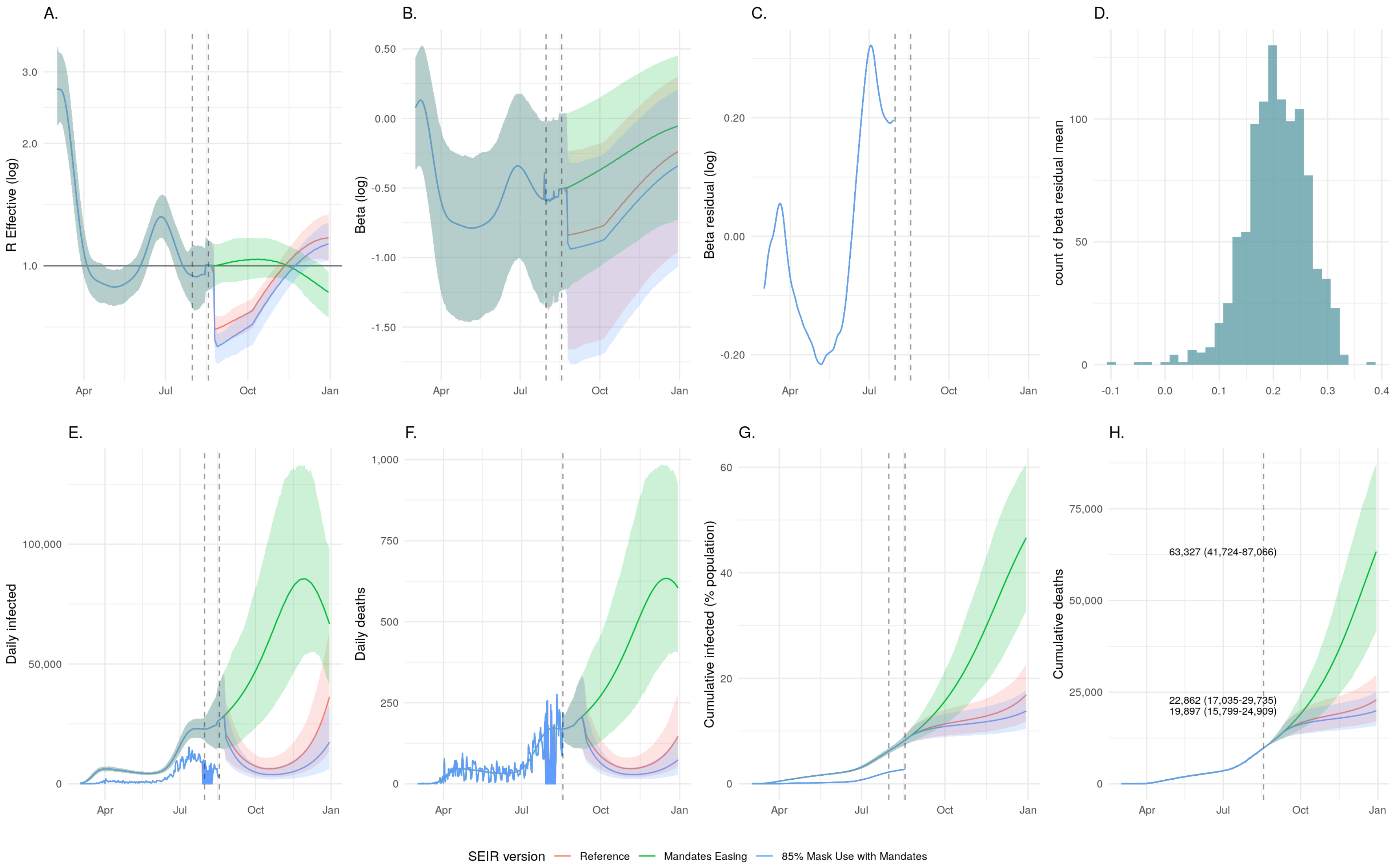

Florida: SEIR fit comparison. A: predicted R effective for each model through December 31. **B**: predicted SEIR *beta* parameter. **C**: residual of predicted *beta* and the observed value calculated directly from infection data over time. **D**: histogram of residual values for *beta*. Panels A, B, C, and D are all displayed in log space, reflecting the space in which the SEIR model is fit. **E**: predicted daily infections from each model through December 31. **F**: predicted daily deaths from each model through December 31. **G**: predicted cumulative infections through December 31, as a proportion of the total population. **H**: predicted cumulative deaths through December 31. In panels E, F, G, and H, reported death and infections are plotted alongside model predictions in light blue.

22 Florida: Covariate fits and regression coefficients

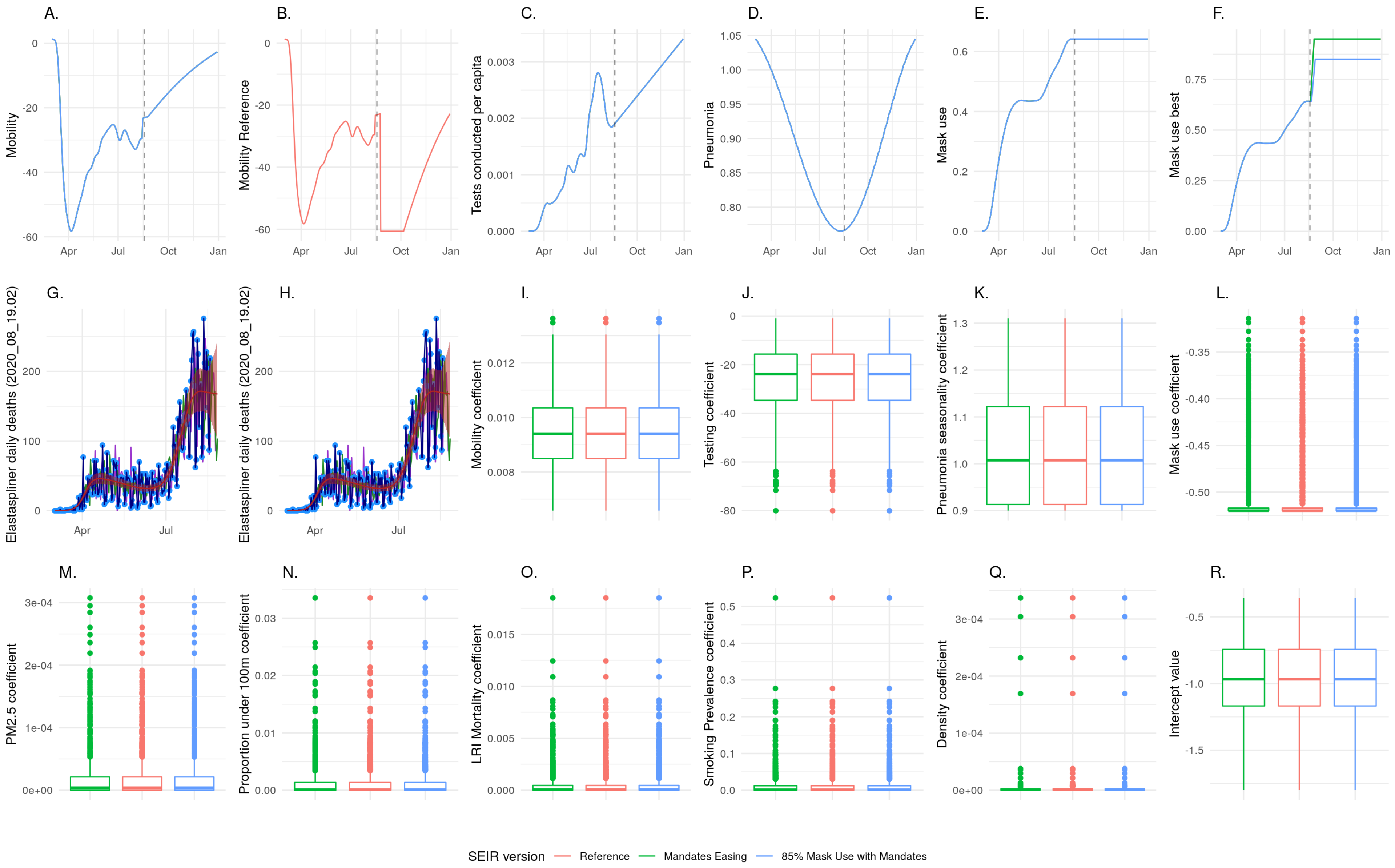

Florida: Covariate fits and regression coefficients. A-F: Line plots showing predicted covariate time trends for **A)** mobility in the absence of additional mandates; **B)** mobility with additional mandates applied; **C)** diagnostic testing per capita; **D)** pneumonia seasonality; **E)** mask use per capita, and; **F)** mask use in a scenario where adherence increases to 85% of the population. **G-H**: COVID mortality data generated from reported daily deaths (blue); estimated based on reported hospitalizations (purple); estimated from reported cases (green); and via a spline fit through all available data types (red, 95% UI in pink). **I-R**: Box plots showing 1,000 draws of fixed effect coefficients in a multivariate regression fit to $log(beta)$.

23 Georgia: SEIR fit comparison

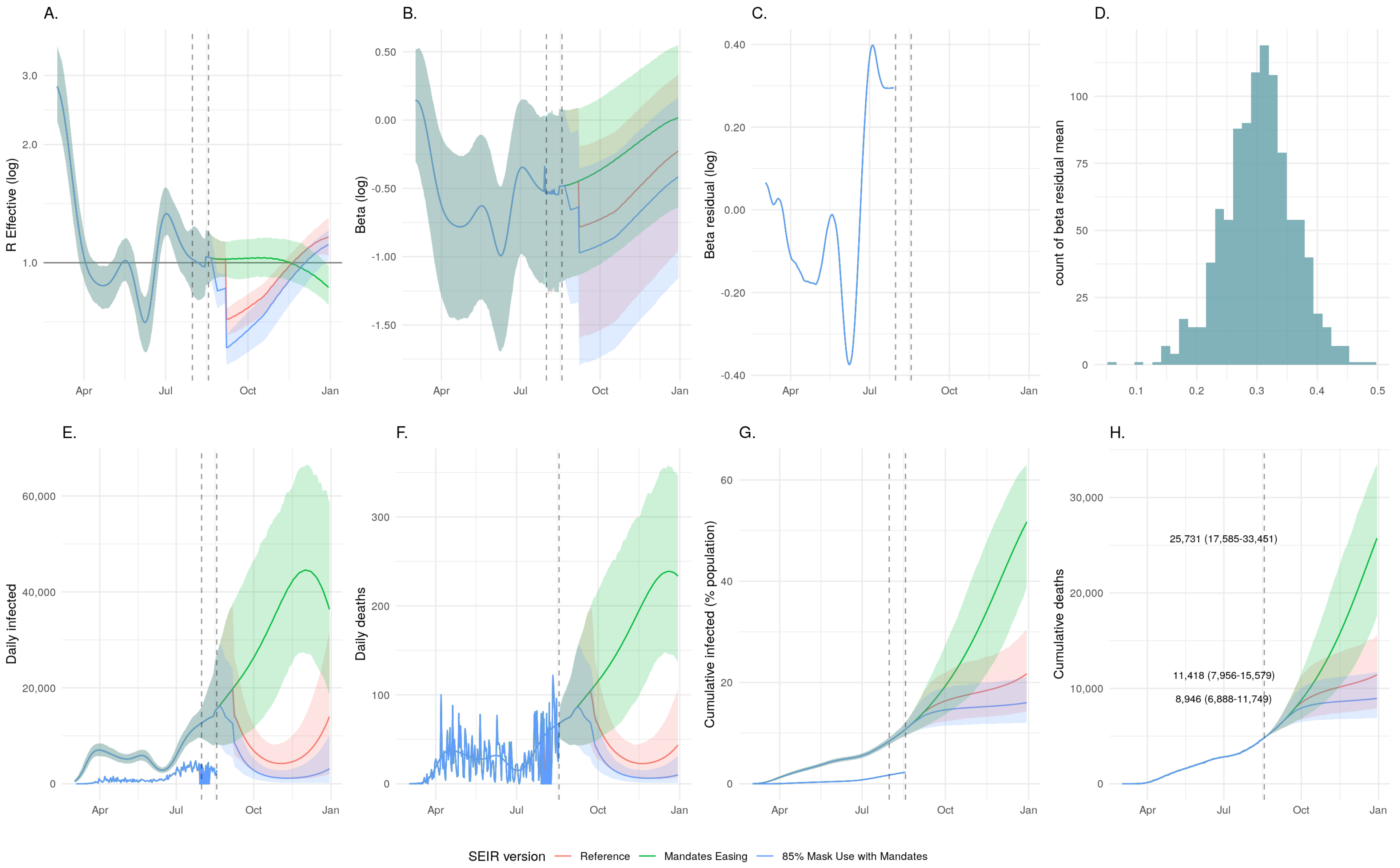

Georgia: SEIR fit comparison. **A**: predicted R effective for each model through December 31. **B**: predicted SEIR *beta* parameter. **C**: residual of predicted *beta* and the observed value calculated directly from infection data over time. **D**: histogram of residual values for *beta*. Panels A, B, C, and D are all displayed in log space, reflecting the space in which the SEIR model is fit. **E**: predicted daily infections from each model through December 31. **F**: predicted daily deaths from each model through December 31. **G**: predicted cumulative infections through December 31, as a proportion of the total population. **H**: predicted cumulative deaths through December 31. In panels E, F, G, and H, reported death and infections are plotted alongside model predictions in light blue.

24 Georgia: Covariate fits and regression coefficients

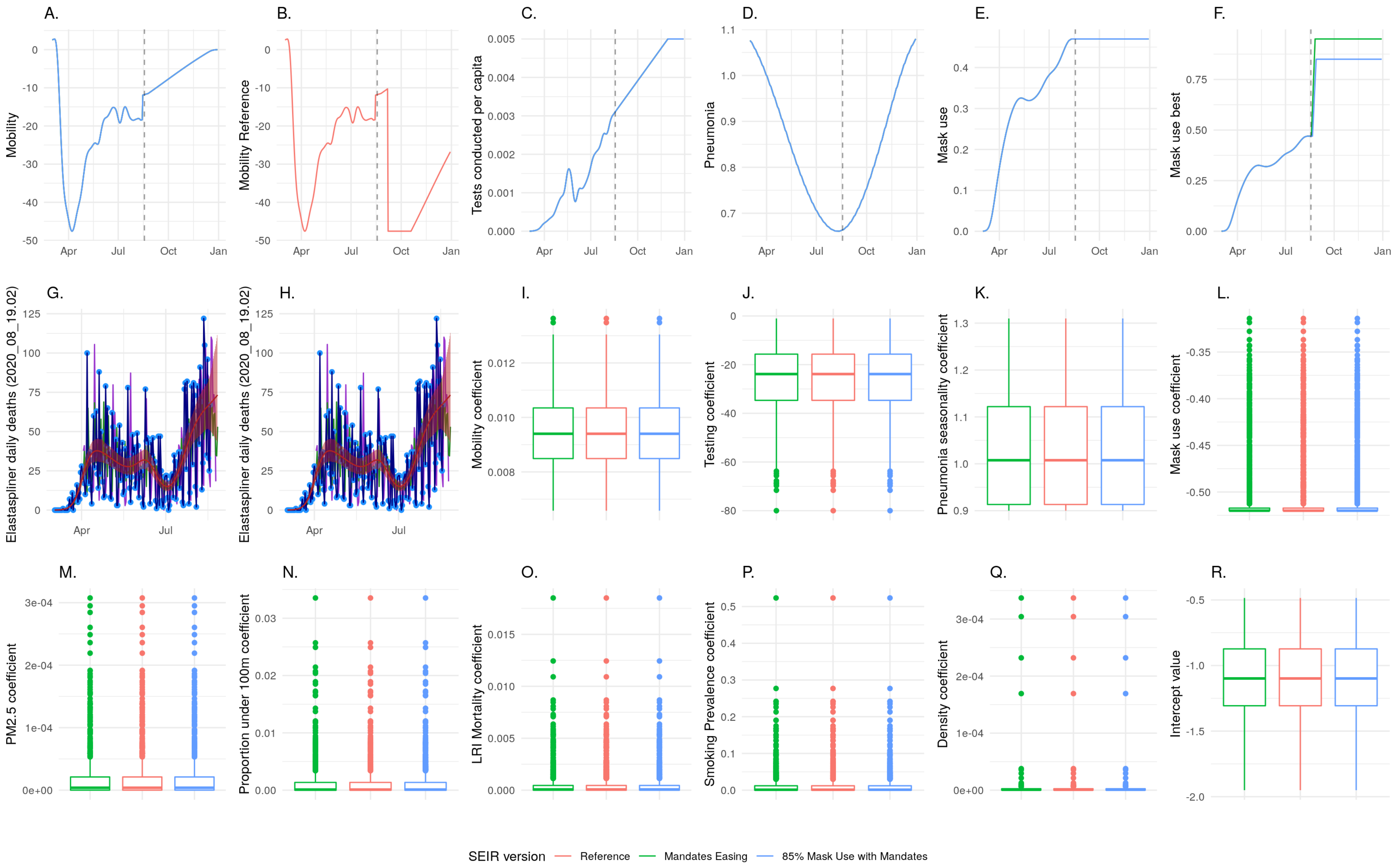

Georgia: Covariate fits and regression coefficients. A-F: Line plots showing predicted covariate time trends for **A)** mobility in the absence of additional mandates; **B)** mobility with additional mandates applied; **C)** diagnostic testing per capita; **D)** pneumonia seasonality; **E)** mask use per capita, and; **F)** mask use in a scenario where adherence increases to 85% of the population. **G-H**: COVID mortality data generated from reported daily deaths (blue); estimated based on reported hospitalizations (purple); estimated from reported cases (green); and via a spline fit through all available data types (red, 95% UI in pink). **I-R**: Box plots showing 1,000 draws of fixed effect coefficients in a multivariate regression fit to $log(beta)$.

25 Hawaii: SEIR fit comparison

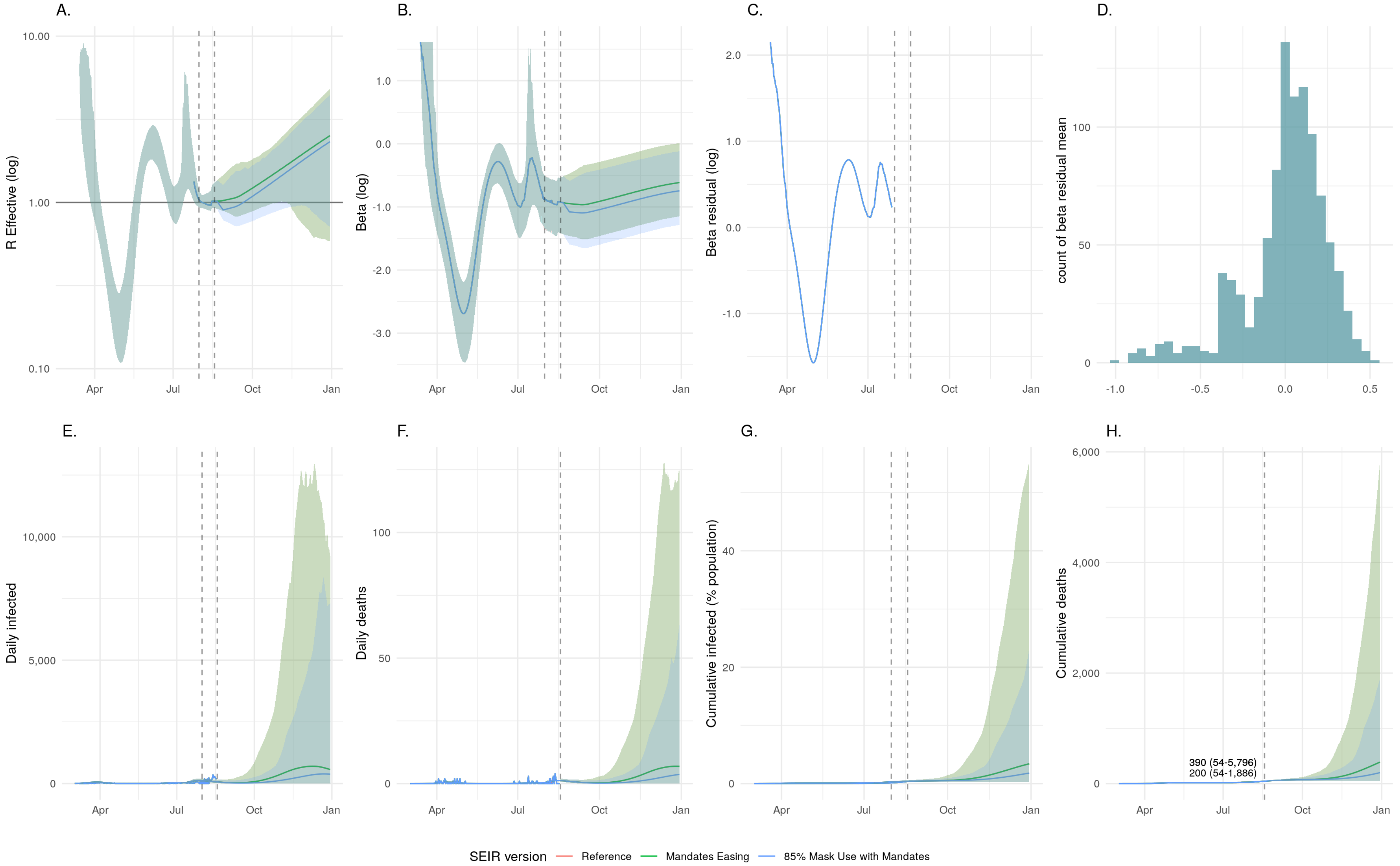

Hawaii: SEIR fit comparison. A: predicted R effective for each model through December 31. **B**: predicted SEIR *beta* parameter. **C**: residual of predicted *beta* and the observed value calculated directly from infection data over time. **D**: histogram of residual values for *beta*. Panels A, B, C, and D are all displayed in log space, reflecting the space in which the SEIR model is fit. **E**: predicted daily infections from each model through December 31. **F**: predicted daily deaths from each model through December 31. **G**: predicted cumulative infections through December 31, as a proportion of the total population. **H**: predicted cumulative deaths through December 31. In panels E, F, G, and H, reported death and infections are plotted alongside model predictions in light blue.

26 Hawaii: Covariate fits and regression coefficients

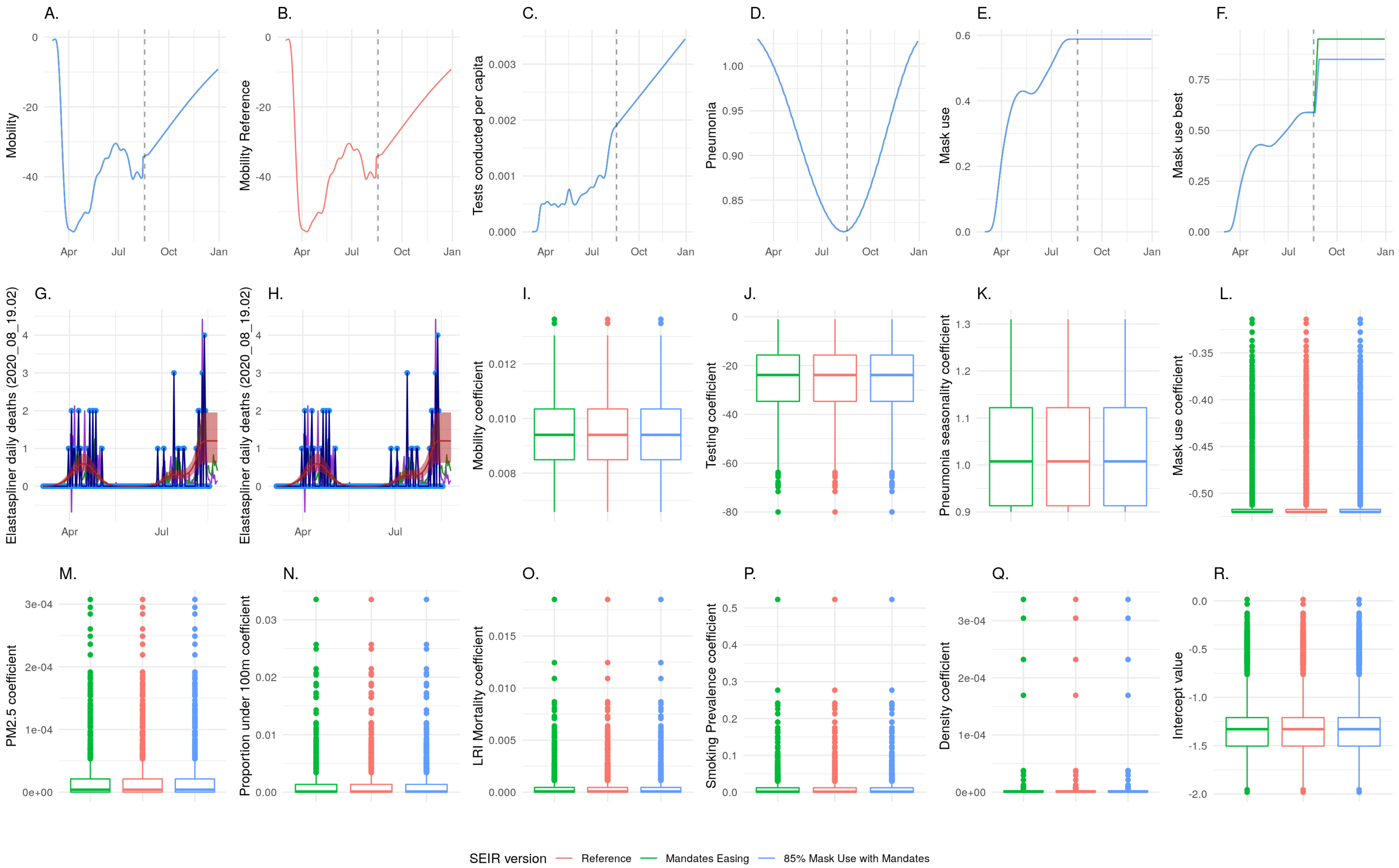

Hawaii: Covariate fits and regression coefficients. **A-F**: Line plots showing predicted covariate time trends for **A)** mobility in the absence of additional mandates; **B)** mobility with additional mandates applied; **C)** diagnostic testing per capita; **D)** pneumonia seasonality; **E)** mask use per capita, and; **F)** mask use in a scenario where adherence increases to 85% of the population. **G-H**: COVID mortality data generated from reported daily deaths (blue); estimated based on reported hospitalizations (purple); estimated from reported cases (green); and via a spline fit through all available data types (red, 95% UI in pink). **I-R**: Box plots showing 1,000 draws of fixed effect coefficients in a multivariate regression fit to $log(beta)$.

27 Idaho: SEIR fit comparison

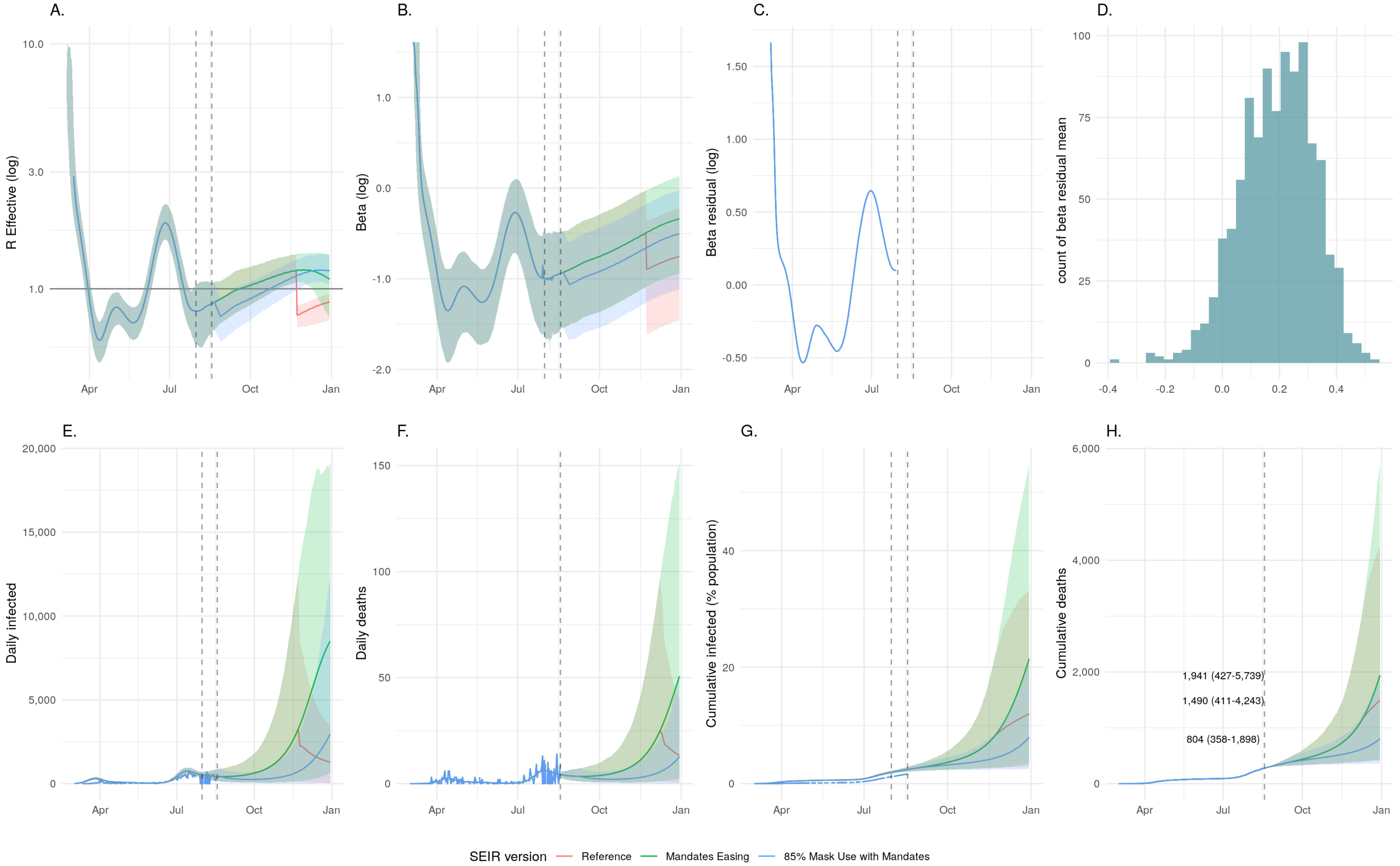

Idaho: SEIR fit comparison. **A**: predicted R effective for each model through December 31. **B**: predicted SEIR *beta* parameter. **C**: residual of predicted *beta* and the observed value calculated directly from infection data over time. **D**: histogram of residual values for *beta*. Panels A, B, C, and D are all displayed in log space, reflecting the space in which the SEIR model is fit. **E**: predicted daily infections from each model through December 31. **F**: predicted daily deaths from each model through December 31. **G**: predicted cumulative infections through December 31, as a proportion of the total population. **H**: predicted cumulative deaths through December 31. In panels E, F, G, and H, reported death and infections are plotted alongside model predictions in light blue.

28 Idaho: Covariate fits and regression coefficients

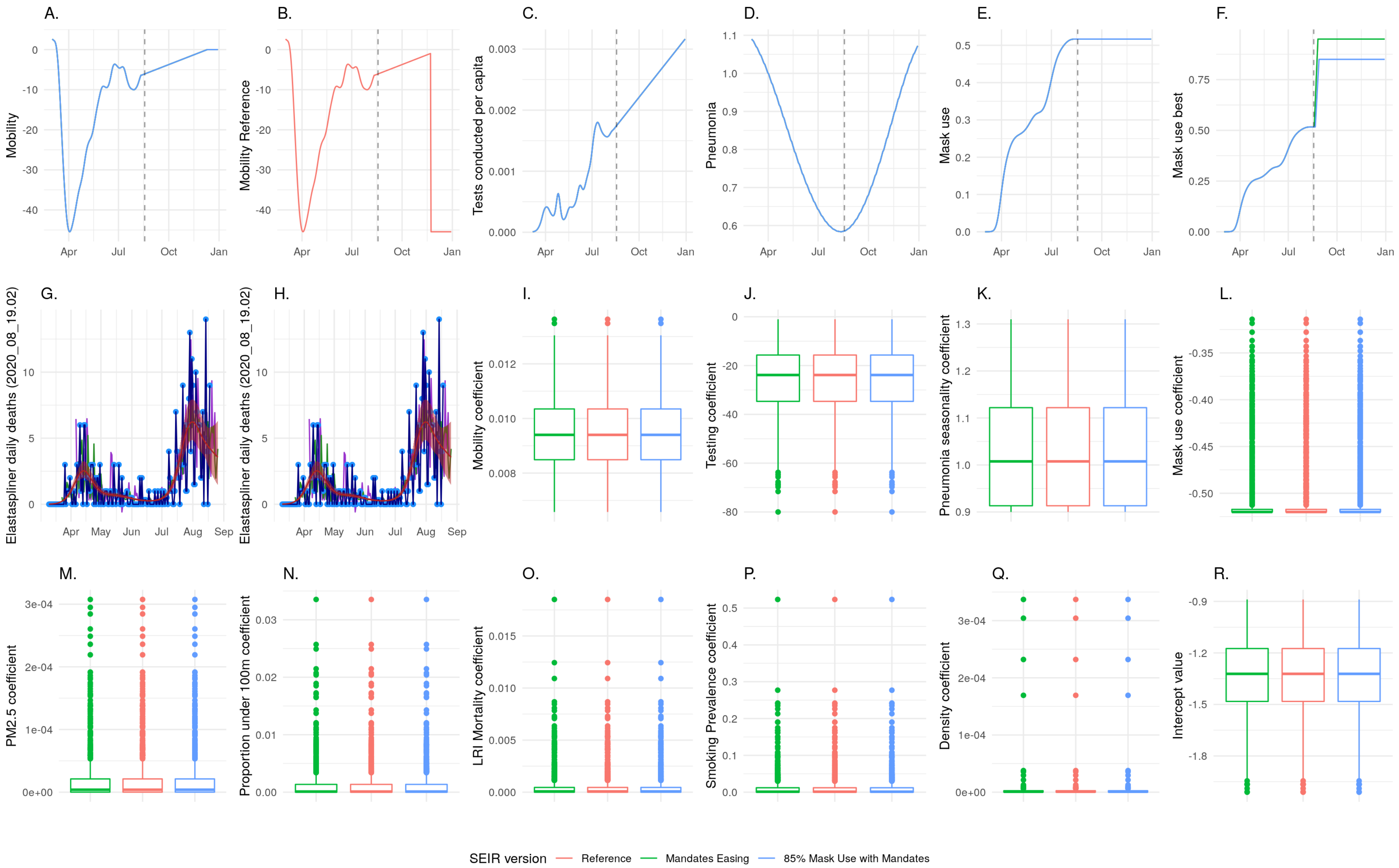

Idaho: Covariate fits and regression coefficients. **A-F**: Line plots showing predicted covariate time trends for **A)** mobility in the absence of additional mandates; **B)** mobility with additional mandates applied; **C)** diagnostic testing per capita; **D)** pneumonia seasonality; **E)** mask use per capita, and; **F)** mask use in a scenario where adherence increases to 85% of the population. **G-H**: COVID mortality data generated from reported daily deaths (blue); estimated based on reported hospitalizations (purple); estimated from reported cases (green); and via a spline fit through all available data types (red, 95% UI in pink). **I-R**: Box plots showing 1,000 draws of fixed effect coefficients in a multivariate regression fit to $log(beta)$.

29 Illinois: SEIR fit comparison

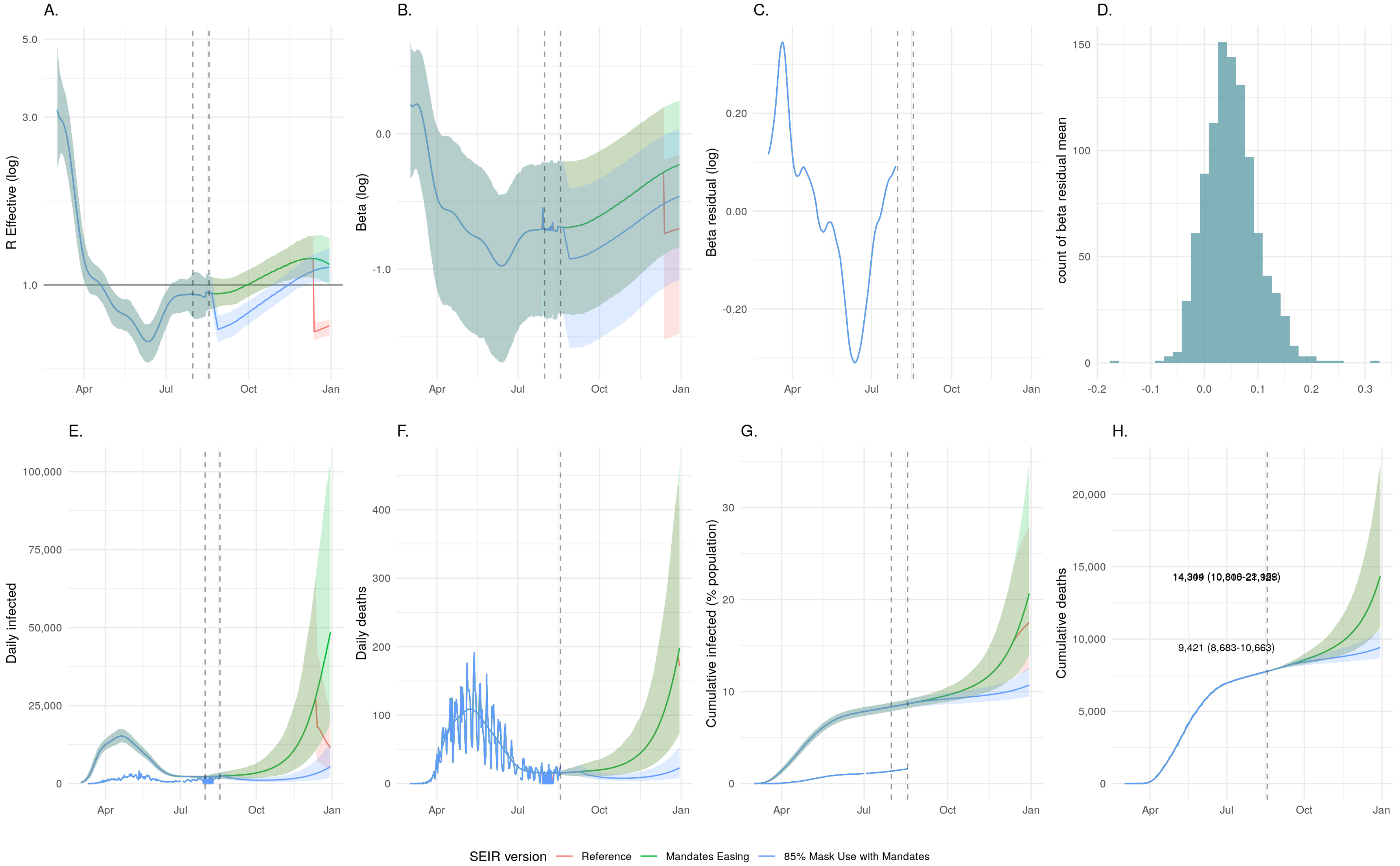

Illinois: SEIR fit comparison. A: predicted R effective for each model through December 31. **B**: predicted SEIR *beta* parameter. **C**: residual of predicted *beta* and the observed value calculated directly from infection data over time. **D**: histogram of residual values for *beta*. Panels A, B, C, and D are all displayed in log space, reflecting the space in which the SEIR model is fit. **E**: predicted daily infections from each model through December 31. **F**: predicted daily deaths from each model through December 31. **G**: predicted cumulative infections through December 31, as a proportion of the total population. **H**: predicted cumulative deaths through December 31. In panels E, F, G, and H, reported death and infections are plotted alongside model predictions in light blue.

30 Illinois: Covariate fits and regression coefficients

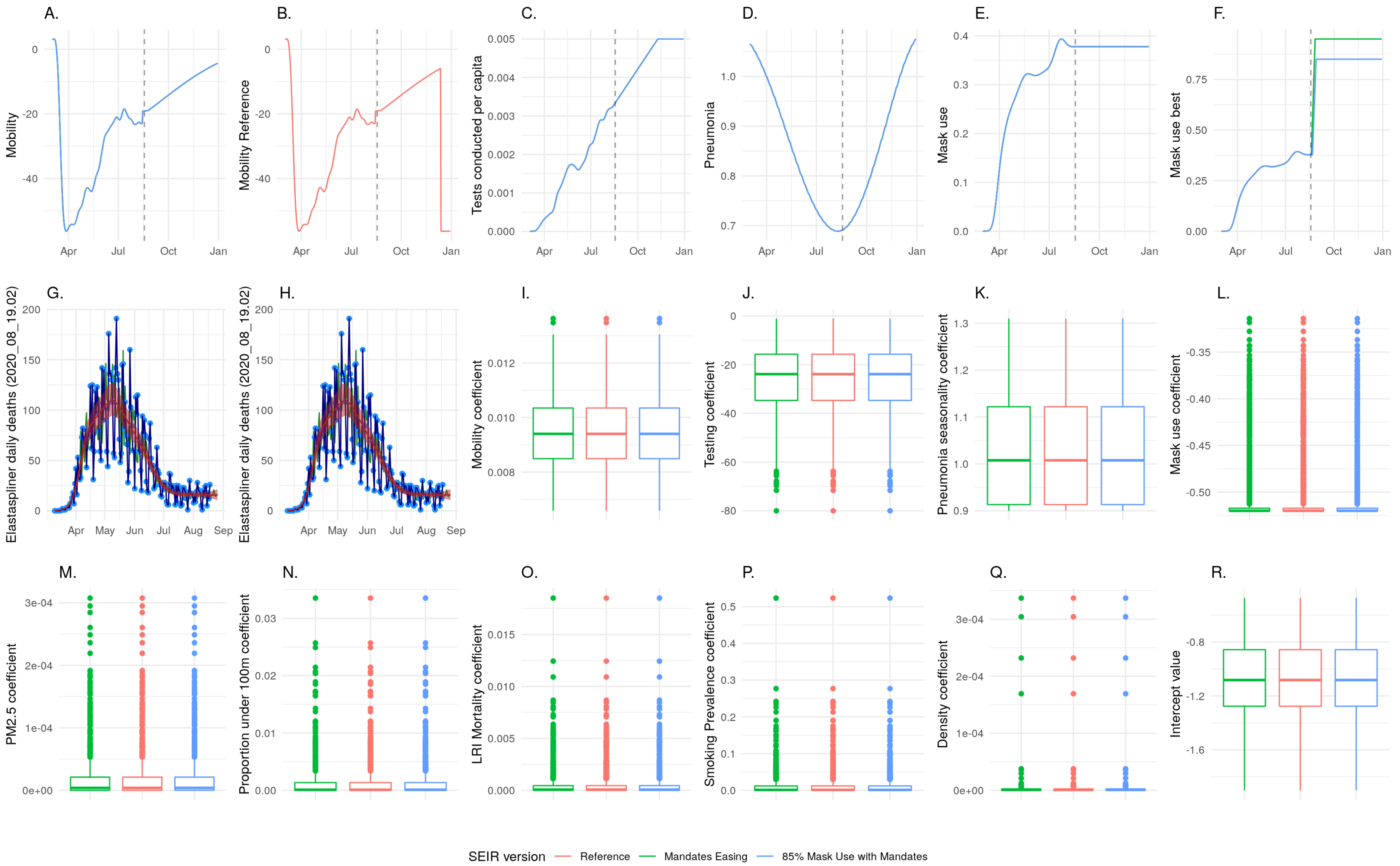

Illinois: Covariate fits and regression coefficients. **A-F**: Line plots showing predicted covariate time trends for **A)** mobility in the absence of additional mandates; **B)** mobility with additional mandates applied; **C)** diagnostic testing per capita; **D)** pneumonia seasonality; **E)** mask use per capita, and; **F)** mask use in a scenario where adherence increases to 85% of the population. **G-H**: COVID mortality data generated from reported daily deaths (blue); estimated based on reported hospitalizations (purple); estimated from reported cases (green); and via a spline fit through all available data types (red, 95% UI in pink). **I-R**: Box plots showing 1,000 draws of fixed effect coefficients in a multivariate regression fit to $log(beta)$.

31 Indiana: SEIR fit comparison

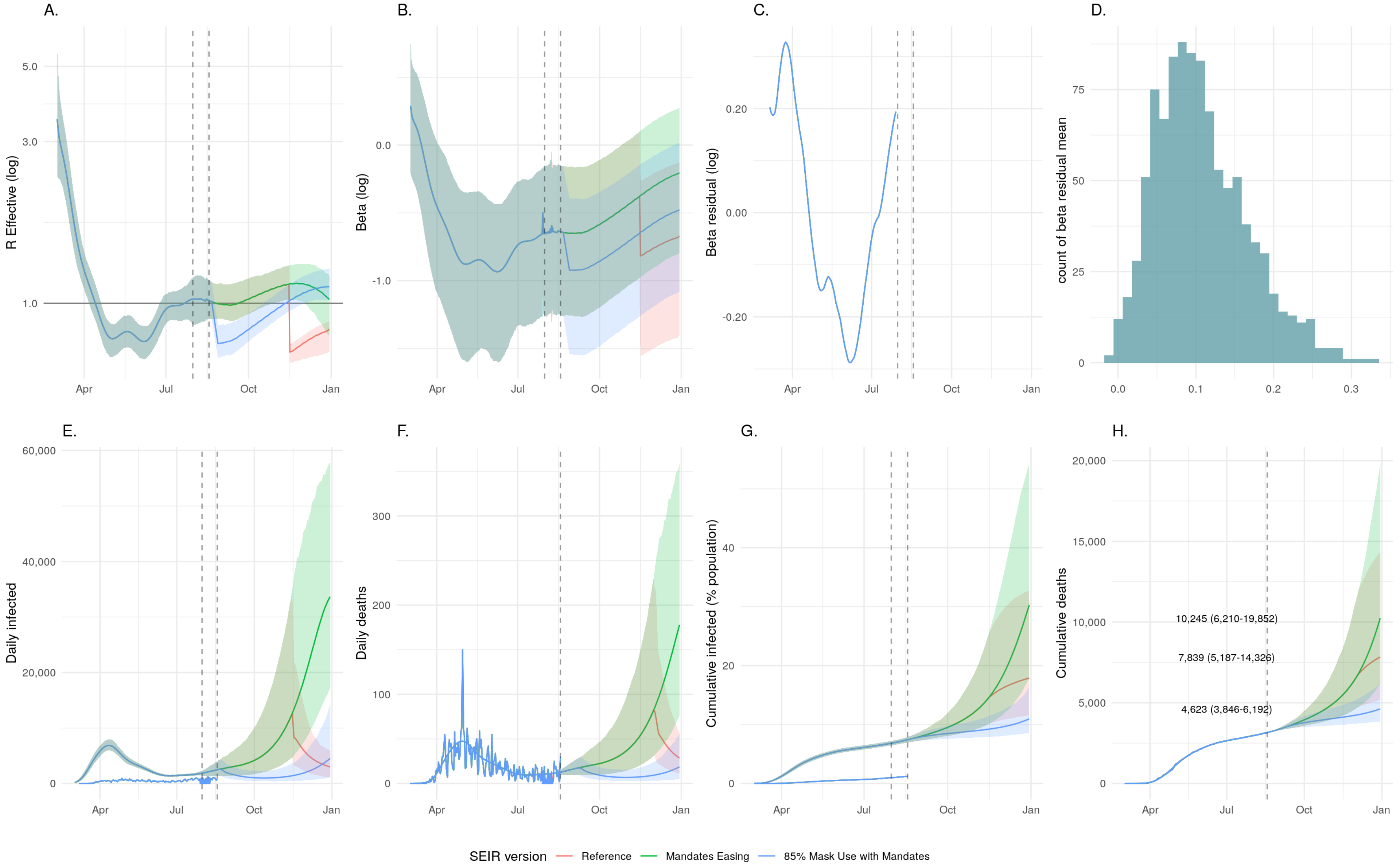

Indiana: SEIR fit comparison. A: predicted R effective for each model through December 31. **B**: predicted SEIR *beta* parameter. **C**: residual of predicted *beta* and the observed value calculated directly from infection data over time. **D**: histogram of residual values for *beta*. Panels A, B, C, and D are all displayed in log space, reflecting the space in which the SEIR model is fit. **E**: predicted daily infections from each model through December 31. **F**: predicted daily deaths from each model through December 31. **G**: predicted cumulative infections through December 31, as a proportion of the total population. **H**: predicted cumulative deaths through December 31. In panels E, F, G, and H, reported death and infections are plotted alongside model predictions in light blue.

32 Indiana: Covariate fits and regression coefficients

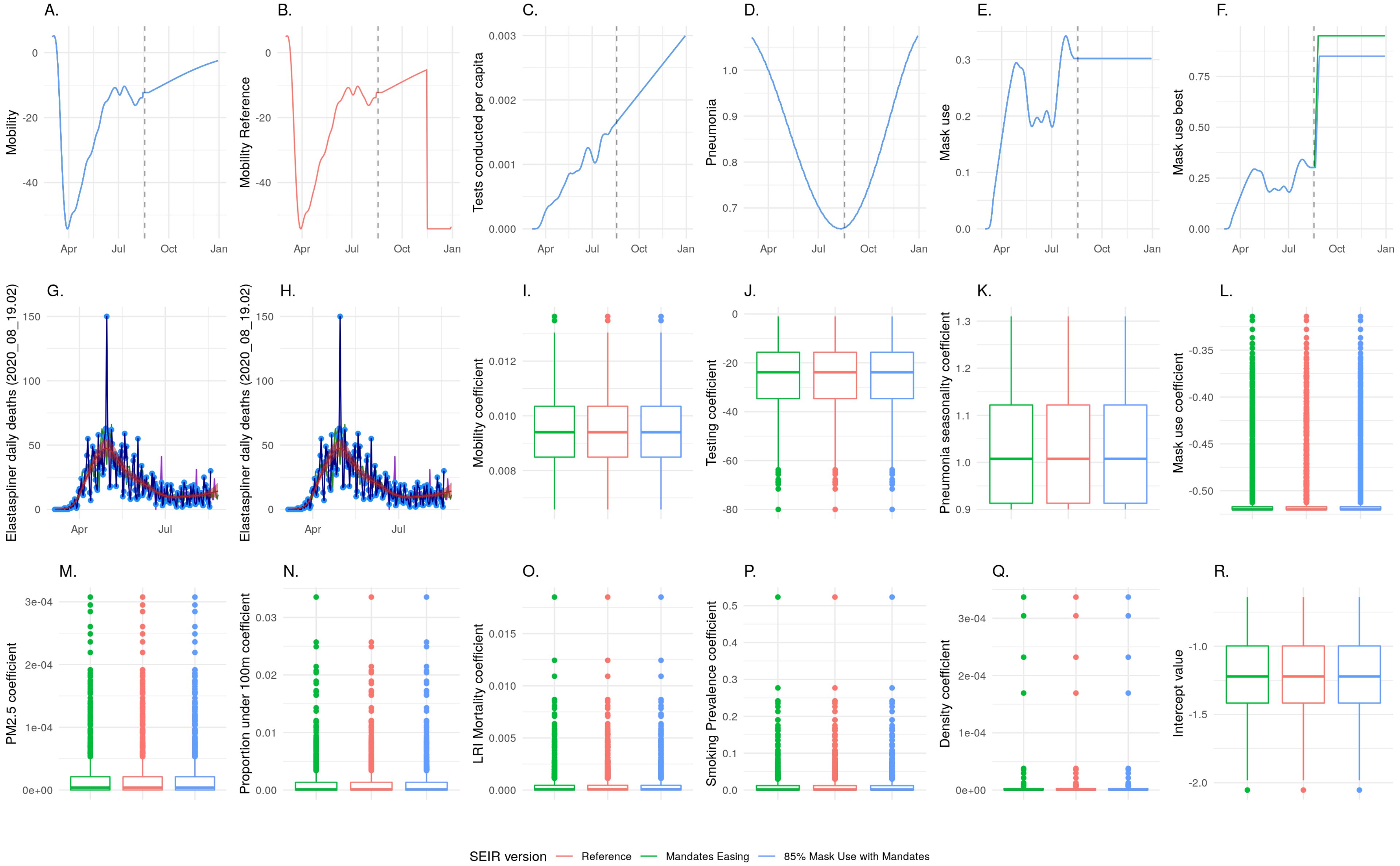

Indiana: Covariate fits and regression coefficients. A-F: Line plots showing predicted covariate time trends for **A)** mobility in the absence of additional mandates; **B)** mobility with additional mandates applied; **C)** diagnostic testing per capita; **D)** pneumonia seasonality; **E)** mask use per capita, and; **F)** mask use in a scenario where adherence increases to 85% of the population. **G-H**: COVID mortality data generated from reported daily deaths (blue); estimated based on reported hospitalizations (purple); estimated from reported cases (green); and via a spline fit through all available data types (red, 95% UI in pink). **I-R**: Box plots showing 1,000 draws of fixed effect coefficients in a multivariate regression fit to $log(beta)$.

33 Iowa: SEIR fit comparison

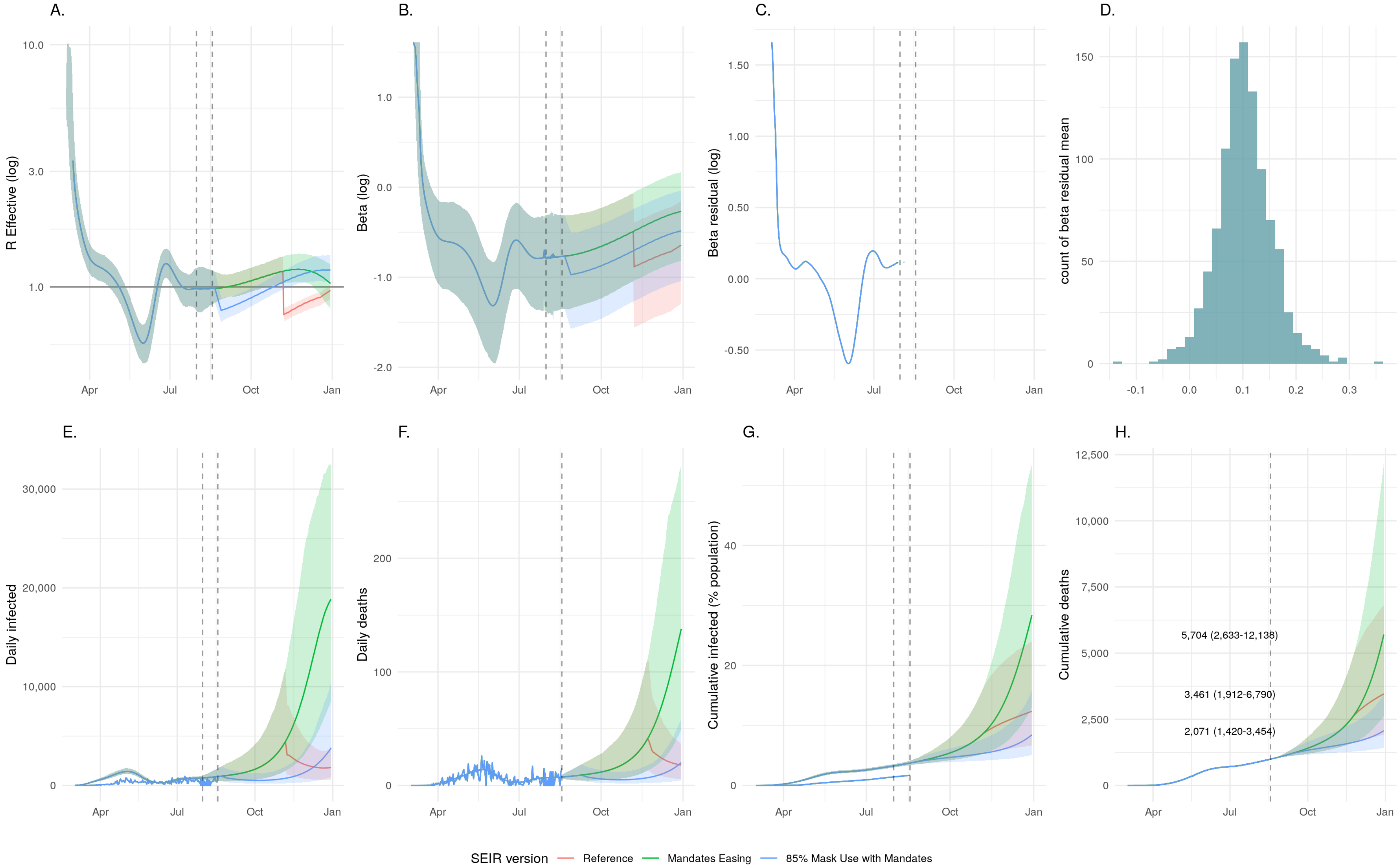

Iowa: SEIR fit comparison. A: predicted R effective for each model through December 31. **B**: predicted SEIR *beta* parameter. **C**: residual of predicted *beta* and the observed value calculated directly from infection data over time. **D**: histogram of residual values for *beta*. Panels A, B, C, and D are all displayed in log space, reflecting the space in which the SEIR model is fit. **E**: predicted daily infections from each model through December 31. **F**: predicted daily deaths from each model through December 31. **G**: predicted cumulative infections through December 31, as a proportion of the total population. **H**: predicted cumulative deaths through December 31. In panels E, F, G, and H, reported death and infections are plotted alongside model predictions in light blue.

34 Iowa: Covariate fits and regression coefficients

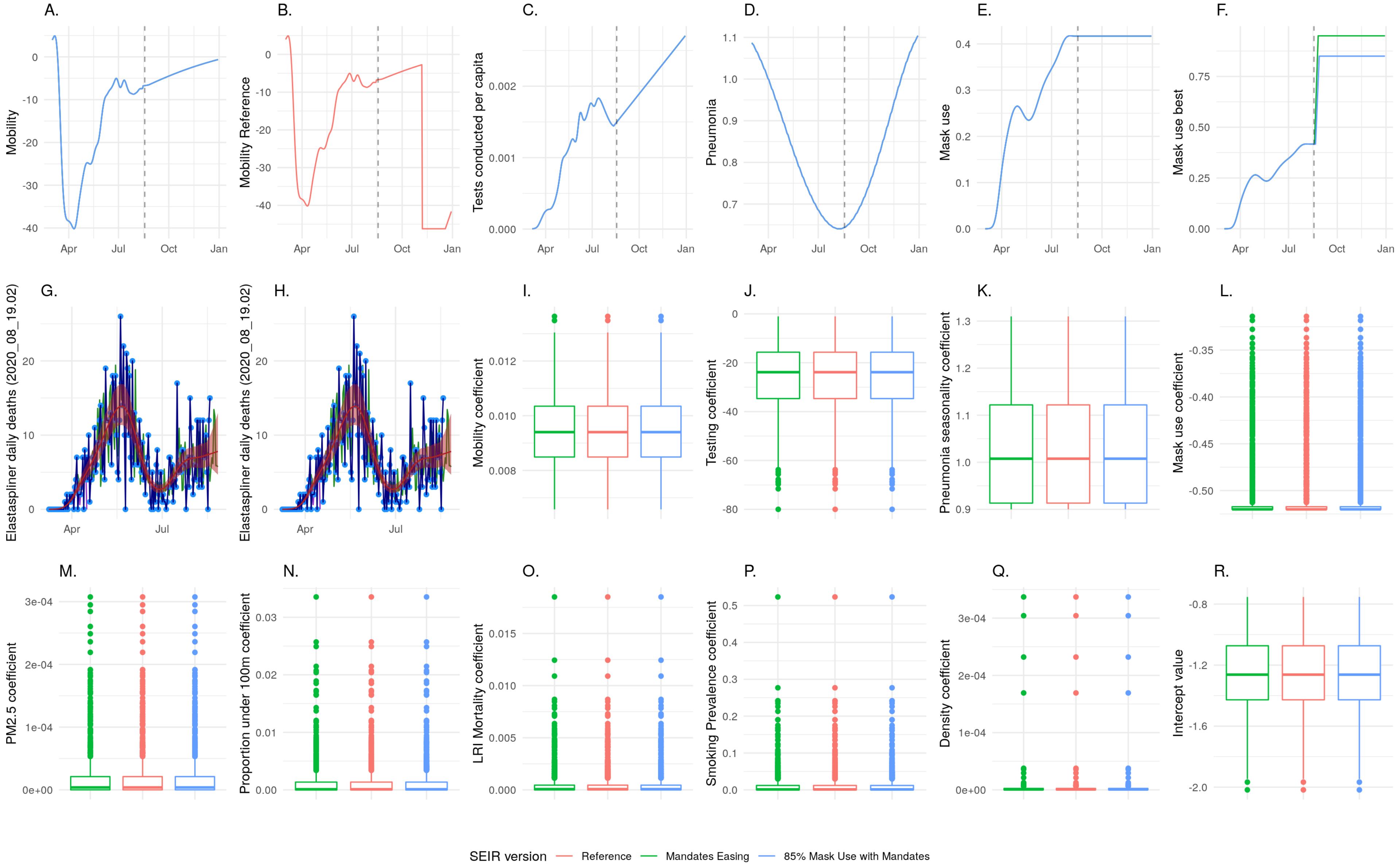

Iowa: Covariate fits and regression coefficients. A-F: Line plots showing predicted covariate time trends for **A)** mobility in the absence of additional mandates; **B)** mobility with additional mandates applied; **C)** diagnostic testing per capita; **D)** pneumonia seasonality; **E)** mask use per capita, and; **F)** mask use in a scenario where adherence increases to 85% of the population. **G-H**: COVID mortality data generated from reported daily deaths (blue); estimated based on reported hospitalizations (purple); estimated from reported cases (green); and via a spline fit through all available data types (red, 95% UI in pink). **I-R**: Box plots showing 1,000 draws of fixed effect coefficients in a multivariate regression fit to $log(beta)$.

35 Kansas: SEIR fit comparison

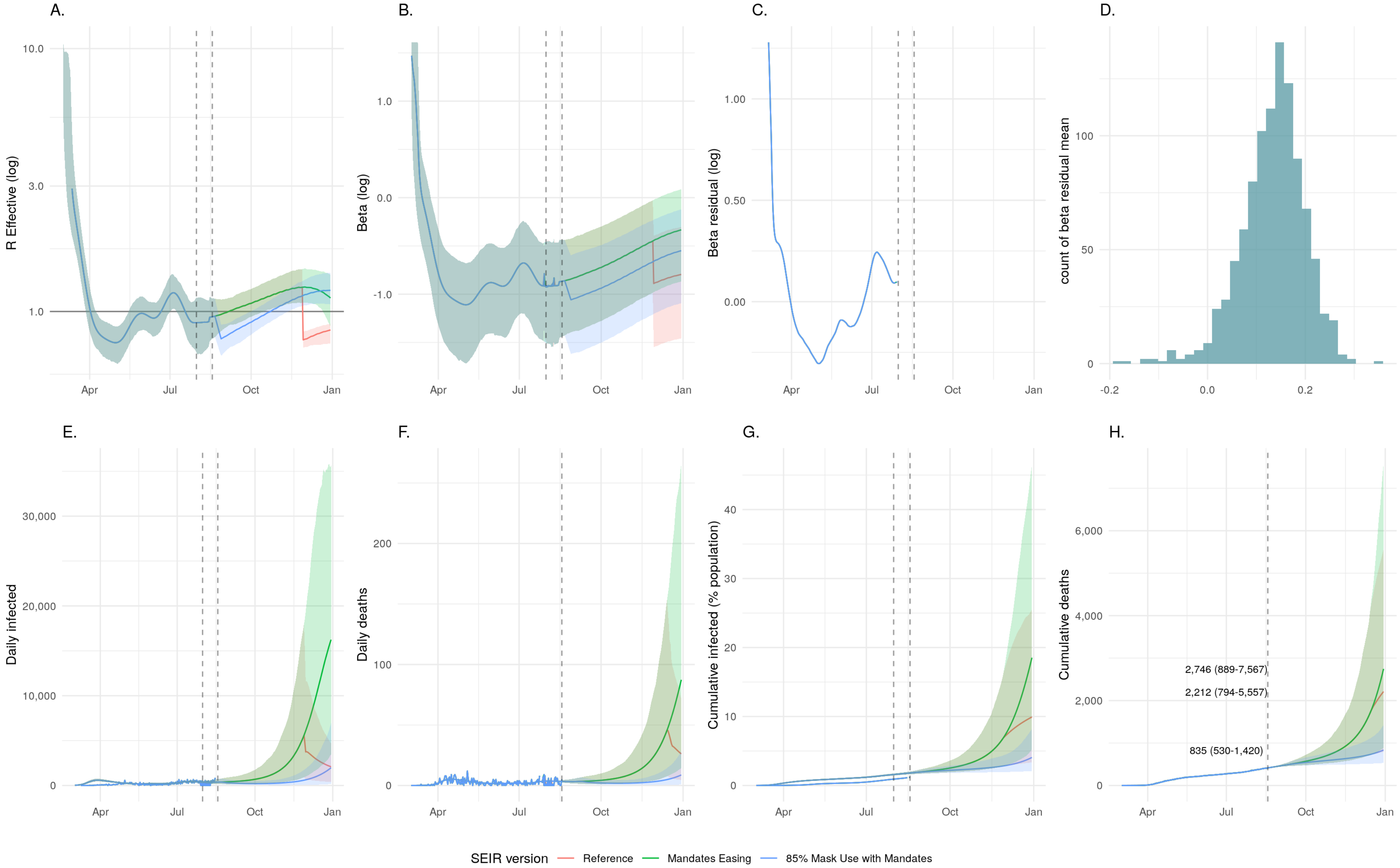

Kansas: SEIR fit comparison. A: predicted R effective for each model through December 31. **B**: predicted SEIR *beta* parameter. **C**: residual of predicted *beta* and the observed value calculated directly from infection data over time. **D**: histogram of residual values for *beta*. Panels A, B, C, and D are all displayed in log space, reflecting the space in which the SEIR model is fit. **E**: predicted daily infections from each model through December 31. **F**: predicted daily deaths from each model through December 31. **G**: predicted cumulative infections through December 31, as a proportion of the total population. **H**: predicted cumulative deaths through December 31. In panels E, F, G, and H, reported death and infections are plotted alongside model predictions in light blue.

36 Kansas: Covariate fits and regression coefficients

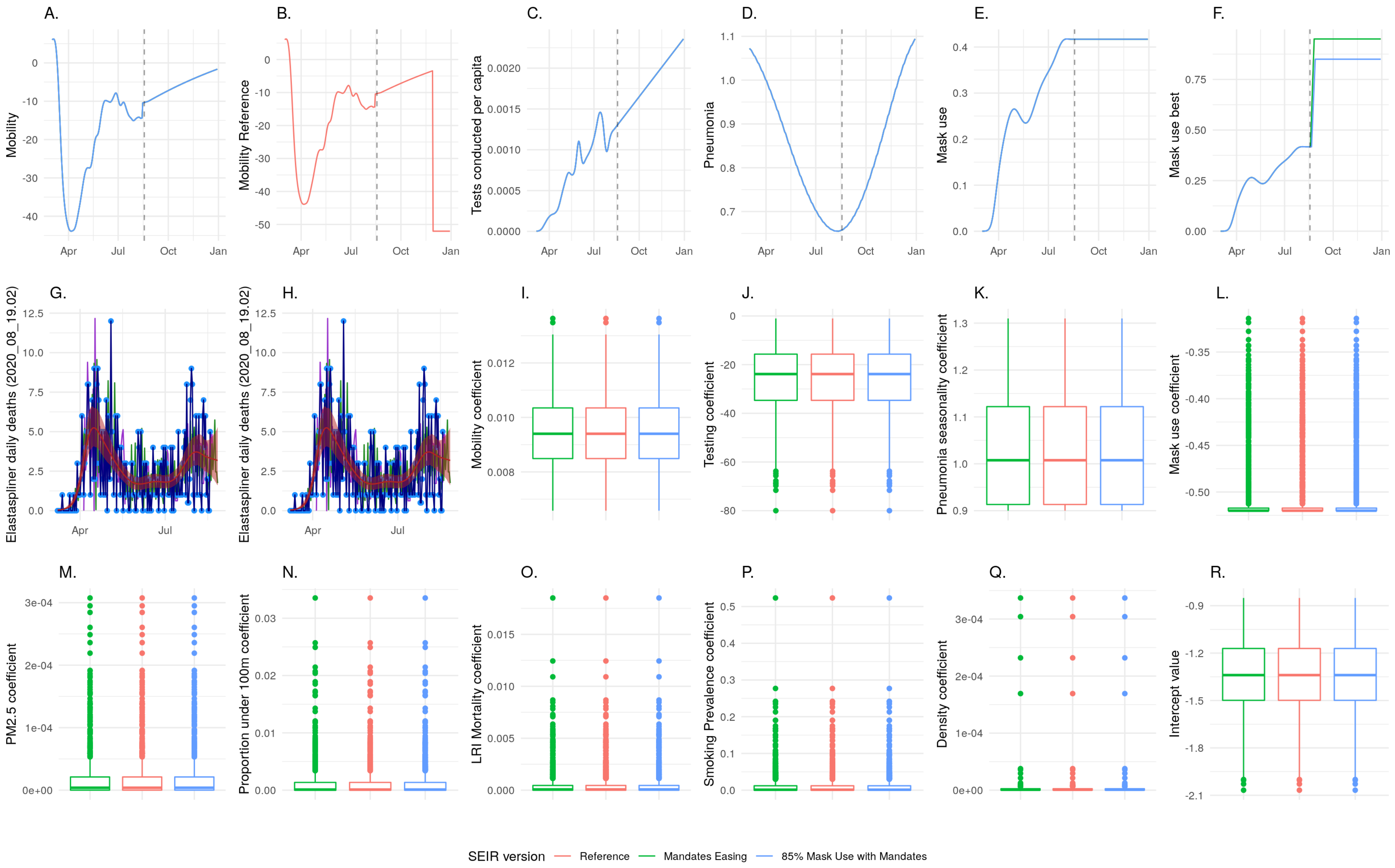

Kansas: Covariate fits and regression coefficients. **A-F**: Line plots showing predicted covariate time trends for **A)** mobility in the absence of additional mandates; **B)** mobility with additional mandates applied; **C)** diagnostic testing per capita; **D)** pneumonia seasonality; **E)** mask use per capita, and; **F)** mask use in a scenario where adherence increases to 85% of the population. **G-H**: COVID mortality data generated from reported daily deaths (blue); estimated based on reported hospitalizations (purple); estimated from reported cases (green); and via a spline fit through all available data types (red, 95% UI in pink). **I-R**: Box plots showing 1,000 draws of fixed effect coefficients in a multivariate regression fit to $log(beta)$.

37 Kentucky: SEIR fit comparison

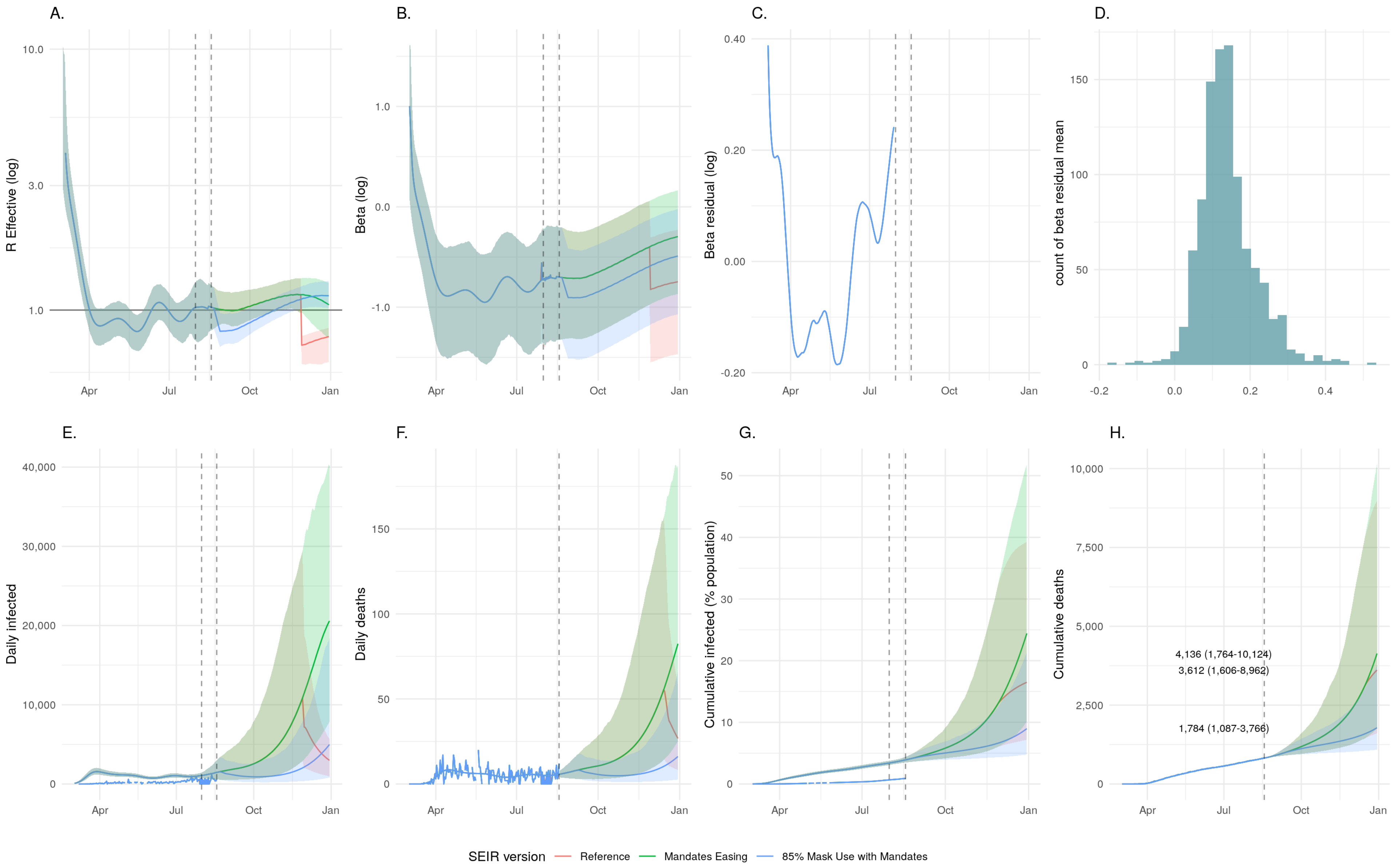

Kentucky: SEIR fit comparison. A: predicted R effective for each model through December 31. **B**: predicted SEIR *beta* parameter. **C**: residual of predicted *beta* and the observed value calculated directly from infection data over time. **D**: histogram of residual values for *beta*. Panels A, B, C, and D are all displayed in log space, reflecting the space in which the SEIR model is fit. **E**: predicted daily infections from each model through December 31. **F**: predicted daily deaths from each model through December 31. **G**: predicted cumulative infections through December 31, as a proportion of the total population. **H**: predicted cumulative deaths through December 31. In panels E, F, G, and H, reported death and infections are plotted alongside model predictions in light blue.

38 Kentucky: Covariate fits and regression coefficients

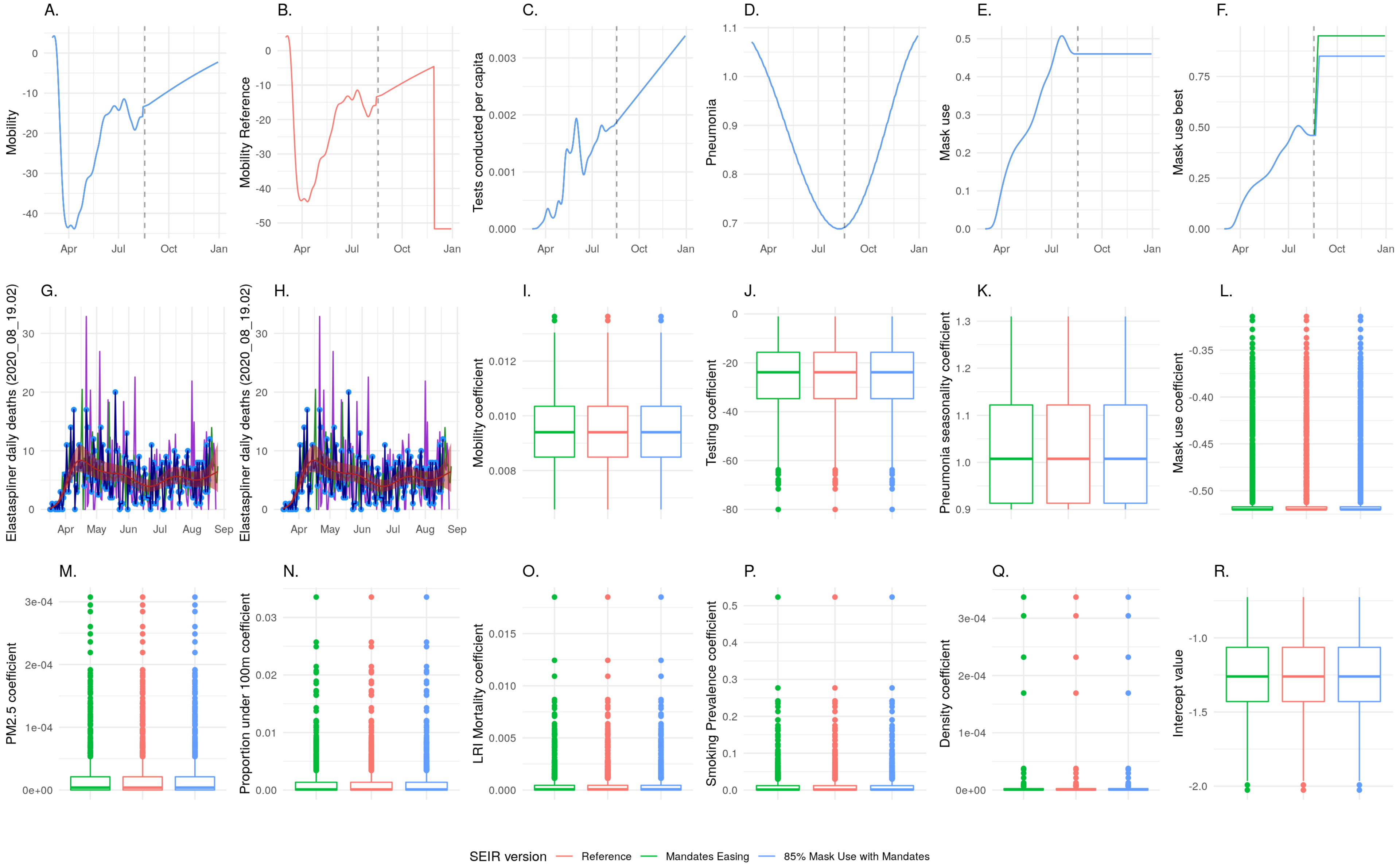

Kentucky: Covariate fits and regression coefficients. **A-F**: Line plots showing predicted covariate time trends for **A)** mobility in the absence of additional mandates; **B)** mobility with additional mandates applied; **C)** diagnostic testing per capita; **D)** pneumonia seasonality; **E)** mask use per capita, and; **F)** mask use in a scenario where adherence increases to 85% of the population. **G-H**: COVID mortality data generated from reported daily deaths (blue); estimated based on reported hospitalizations (purple); estimated from reported cases (green); and via a spline fit through all available data types (red, 95% UI in pink). **I-R**: Box plots showing 1,000 draws of fixed effect coefficients in a multivariate regression fit to $log(beta)$.

39 Louisiana: SEIR fit comparison

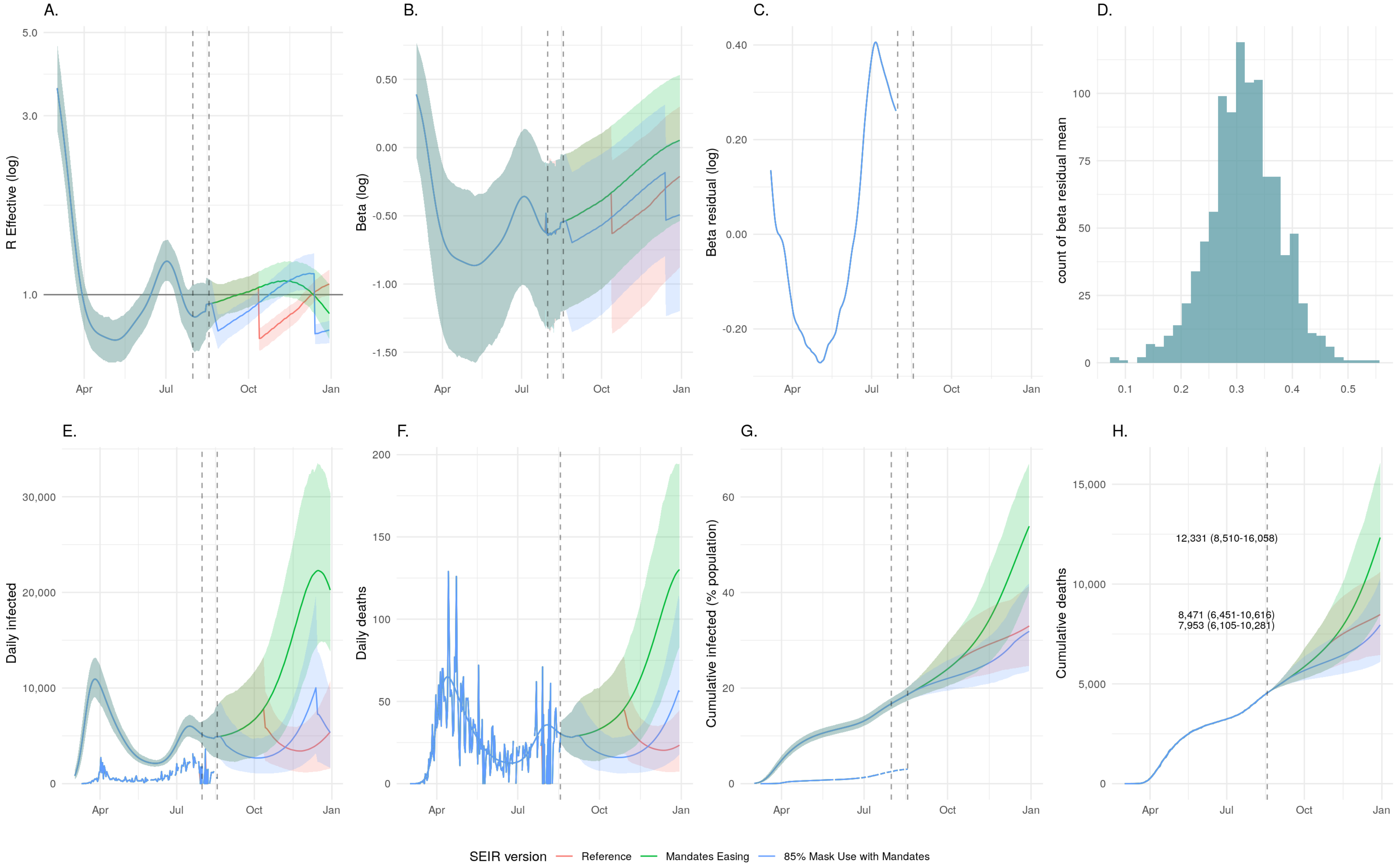

Louisiana: SEIR fit comparison. A: predicted R effective for each model through December 31. **B**: predicted SEIR *beta* parameter. **C**: residual of predicted *beta* and the observed value calculated directly from infection data over time. **D**: histogram of residual values for *beta*. Panels A, B, C, and D are all displayed in log space, reflecting the space in which the SEIR model is fit. **E**: predicted daily infections from each model through December 31. **F**: predicted daily deaths from each model through December 31. **G**: predicted cumulative infections through December 31, as a proportion of the total population. **H**: predicted cumulative deaths through December 31. In panels E, F, G, and H, reported death and infections are plotted alongside model predictions in light blue.

40 Louisiana: Covariate fits and regression coefficients

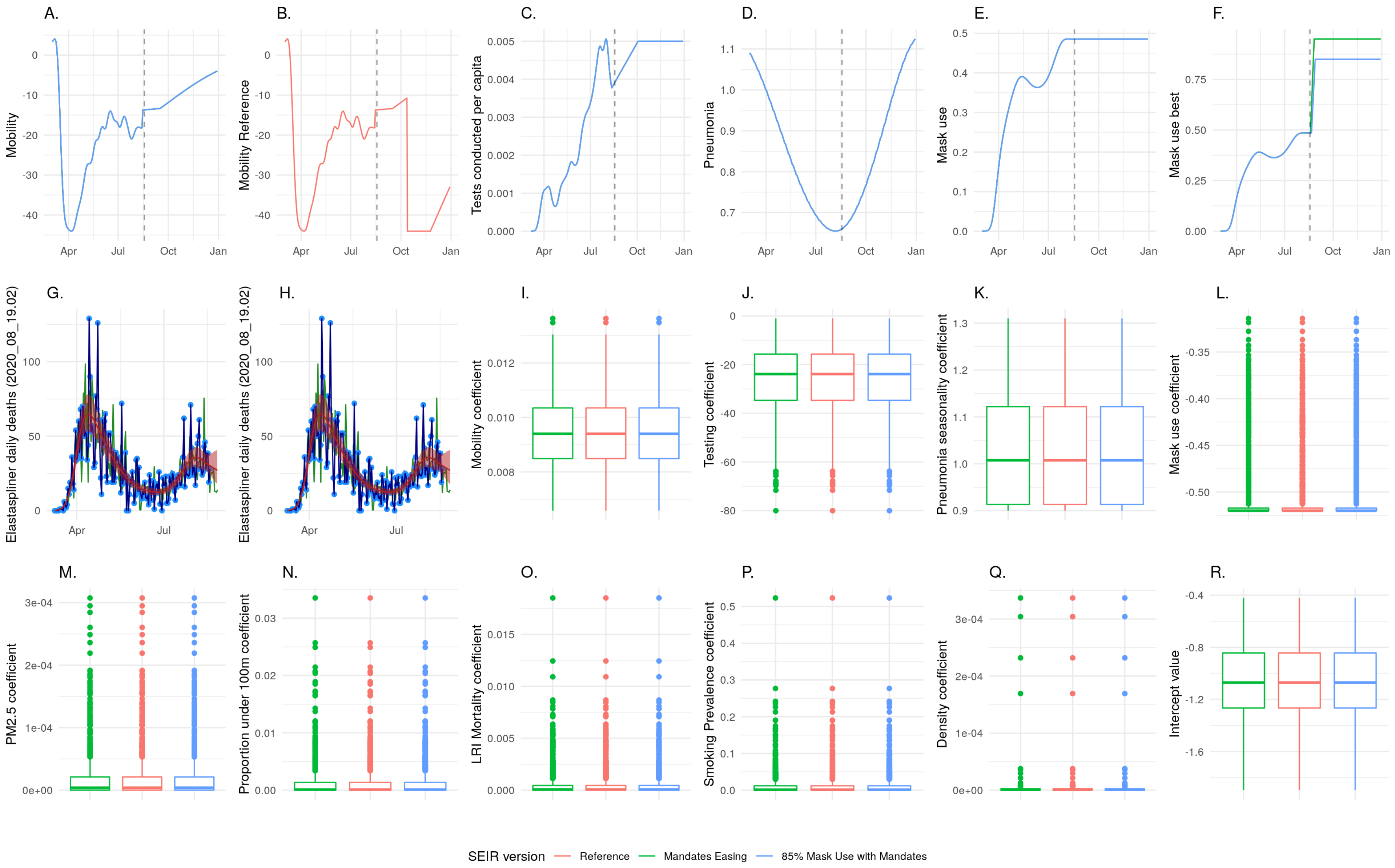

Louisiana: Covariate fits and regression coefficients. A-F: Line plots showing predicted covariate time trends for **A)** mobility in the absence of additional mandates; **B)** mobility with additional mandates applied; **C)** diagnostic testing per capita; **D)** pneumonia seasonality; **E)** mask use per capita, and; **F)** mask use in a scenario where adherence increases to 85% of the population. **G-H**: COVID mortality data generated from reported daily deaths (blue); estimated based on reported hospitalizations (purple); estimated from reported cases (green); and via a spline fit through all available data types (red, 95% UI in pink). **I-R**: Box plots showing 1,000 draws of fixed effect coefficients in a multivariate regression fit to $log(beta)$.

41 Maine: SEIR fit comparison

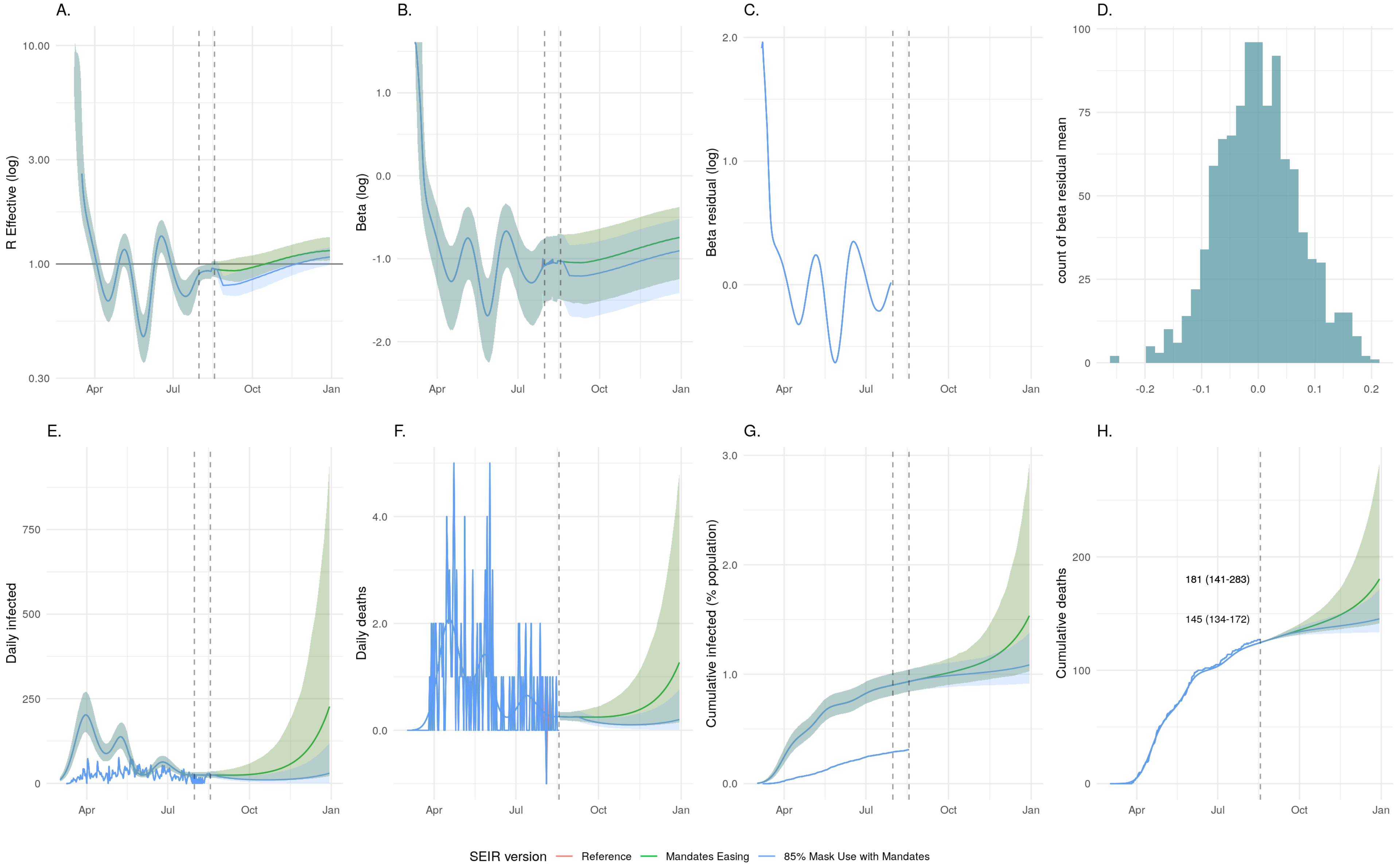

Maine: SEIR fit comparison. **A**: predicted R effective for each model through December 31. **B**: predicted SEIR *beta* parameter. **C**: residual of predicted *beta* and the observed value calculated directly from infection data over time. **D**: histogram of residual values for *beta*. Panels A, B, C, and D are all displayed in log space, reflecting the space in which the SEIR model is fit. **E**: predicted daily infections from each model through December 31. **F**: predicted daily deaths from each model through December 31. **G**: predicted cumulative infections through December 31, as a proportion of the total population. **H**: predicted cumulative deaths through December 31. In panels E, F, G, and H, reported death and infections are plotted alongside model predictions in light blue.

42 Maine: Covariate fits and regression coefficients

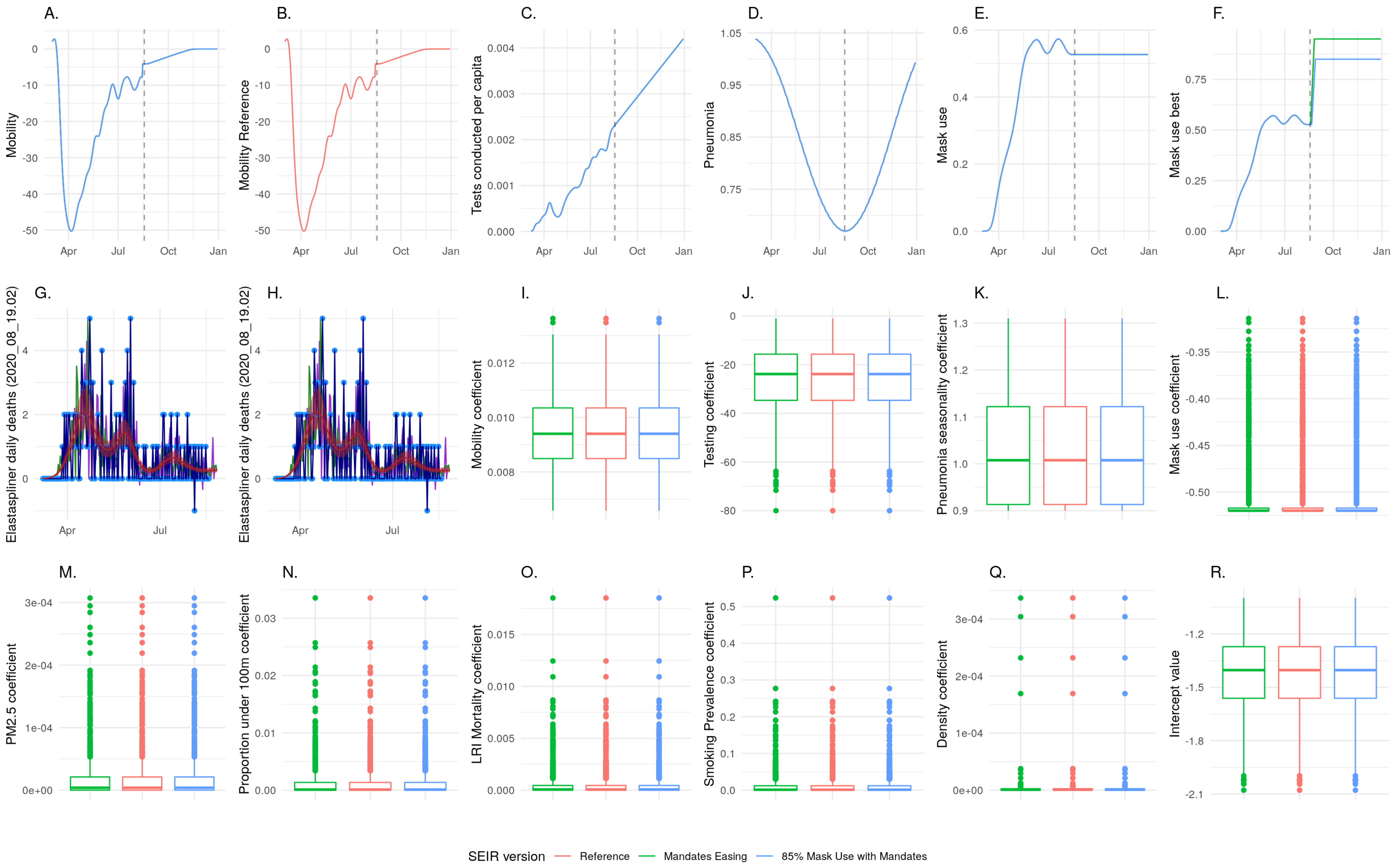

Maine: Covariate fits and regression coefficients. **A-F**: Line plots showing predicted covariate time trends for **A)** mobility in the absence of additional mandates; **B)** mobility with additional mandates applied; **C)** diagnostic testing per capita; **D)** pneumonia seasonality; **E)** mask use per capita, and; **F)** mask use in a scenario where adherence increases to 85% of the population. **G-H**: COVID mortality data generated from reported daily deaths (blue); estimated based on reported hospitalizations (purple); estimated from reported cases (green); and via a spline fit through all available data types (red, 95% UI in pink). **I-R**: Box plots showing 1,000 draws of fixed effect coefficients in a multivariate regression fit to $log(beta)$.

43 Maryland: SEIR fit comparison

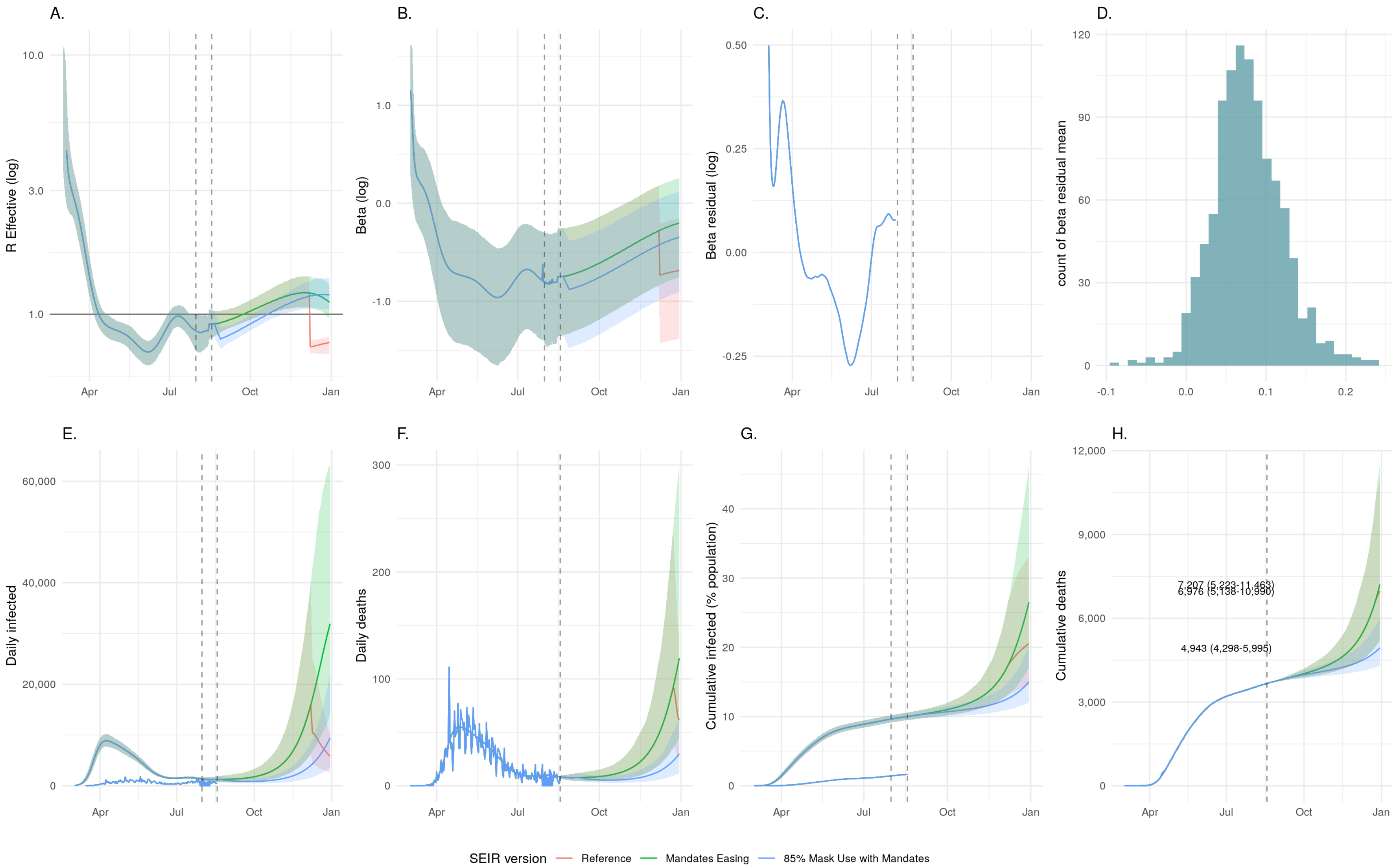

Maryland: SEIR fit comparison. **A**: predicted R effective for each model through December 31. **B**: predicted SEIR *beta* parameter. **C**: residual of predicted *beta* and the observed value calculated directly from infection data over time. **D**: histogram of residual values for *beta*. Panels A, B, C, and D are all displayed in log space, reflecting the space in which the SEIR model is fit. **E**: predicted daily infections from each model through December 31. **F**: predicted daily deaths from each model through December 31. **G**: predicted cumulative infections through December 31, as a proportion of the total population. **H**: predicted cumulative deaths through December 31. In panels E, F, G, and H, reported death and infections are plotted alongside model predictions in light blue.

44 Maryland: Covariate fits and regression coefficients

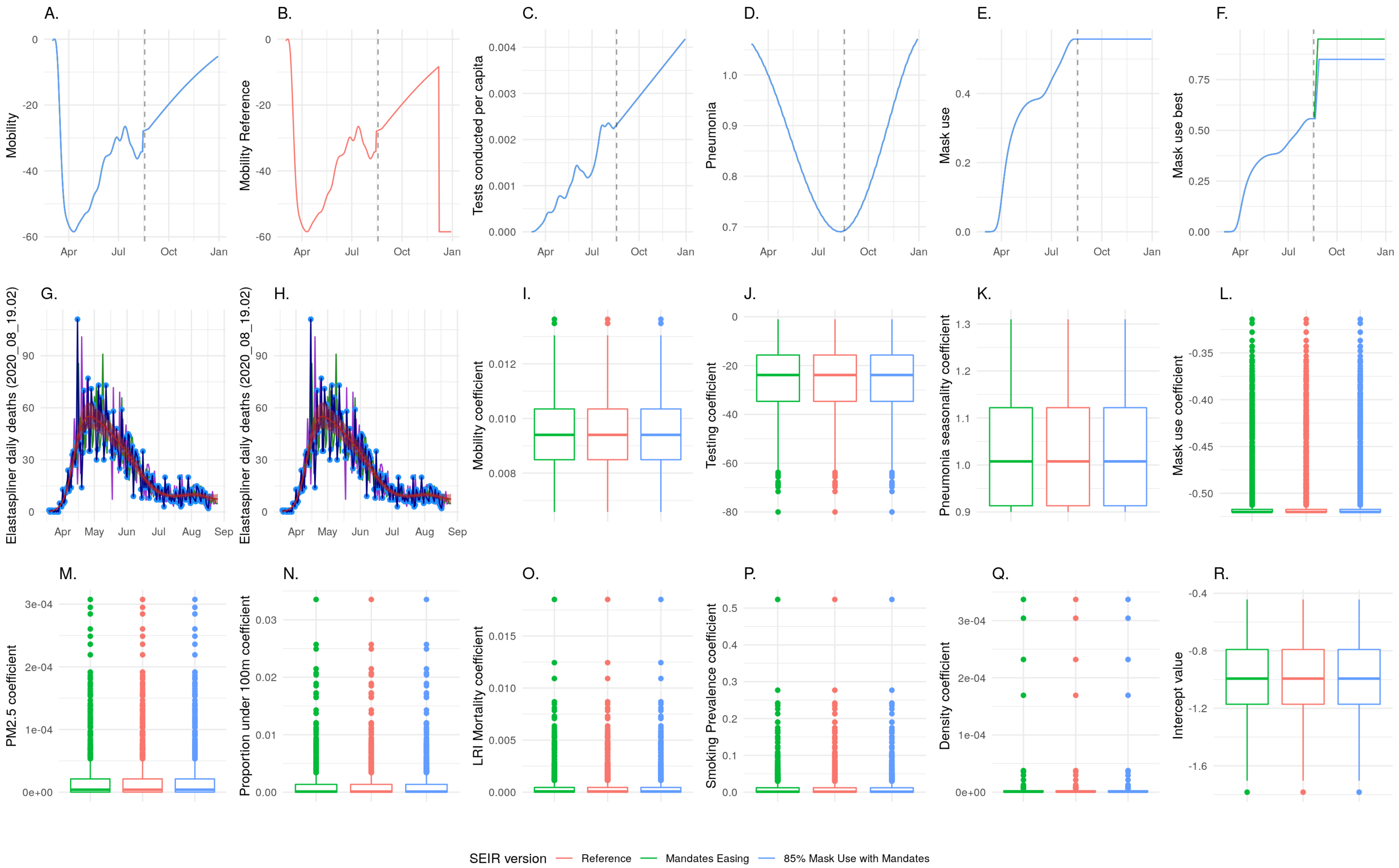

Maryland: Covariate fits and regression coefficients. **A-F**: Line plots showing predicted covariate time trends for **A)** mobility in the absence of additional mandates; **B)** mobility with additional mandates applied; **C)** diagnostic testing per capita; **D)** pneumonia seasonality; **E)** mask use per capita, and; **F)** mask use in a scenario where adherence increases to 85% of the population. **G-H**: COVID mortality data generated from reported daily deaths (blue); estimated based on reported hospitalizations (purple); estimated from reported cases (green); and via a spline fit through all available data types (red, 95% UI in pink). **I-R**: Box plots showing 1,000 draws of fixed effect coefficients in a multivariate regression fit to $log(beta)$.

45 Massachusetts: SEIR fit comparison

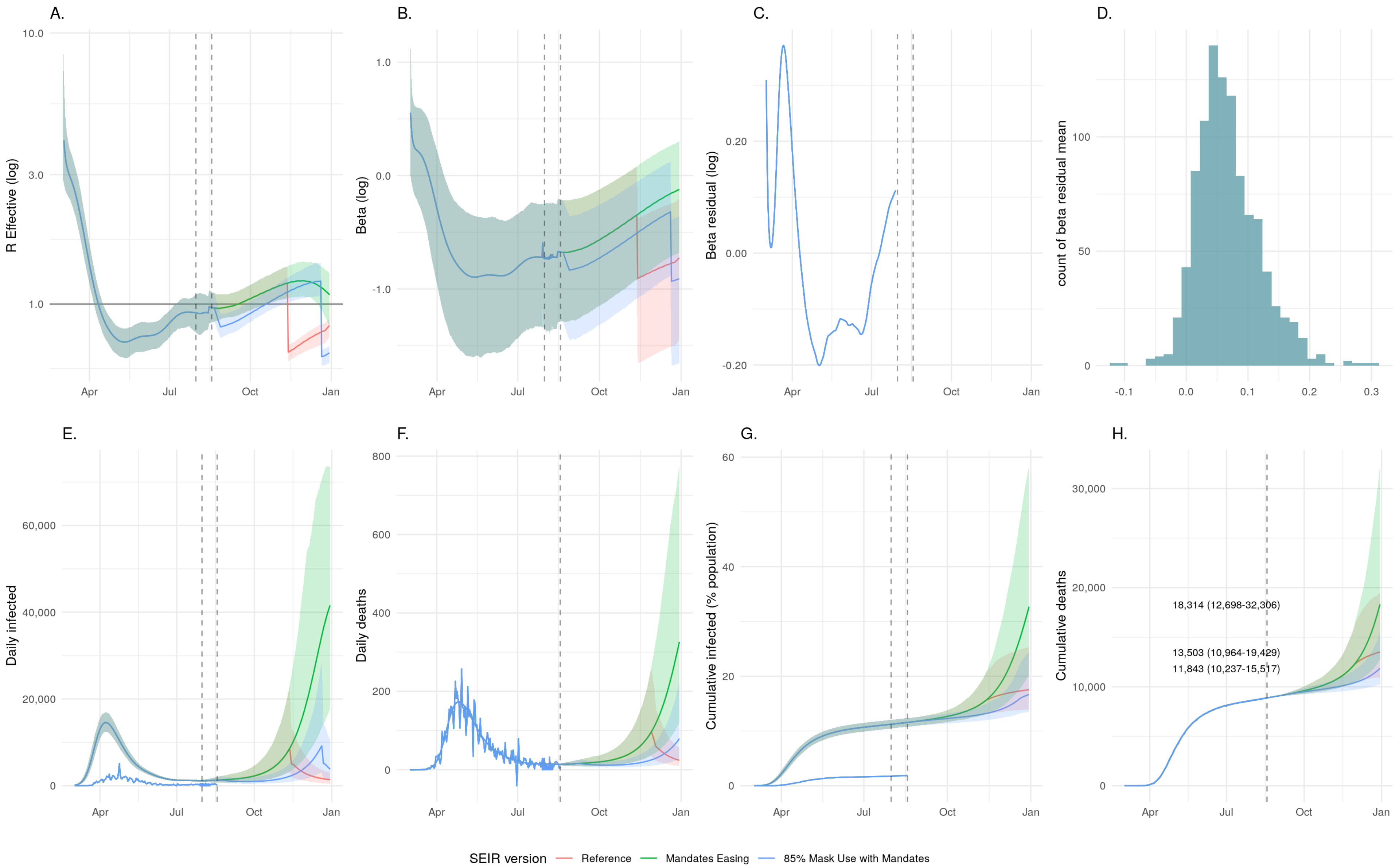

Massachusetts: SEIR fit comparison. **A**: predicted R effective for each model through December 31. **B**: predicted SEIR *beta* parameter. **C**: residual of predicted *beta* and the observed value calculated directly from infection data over time. **D**: histogram of residual values for *beta*. Panels A, B, C, and D are all displayed in log space, reflecting the space in which the SEIR model is fit. **E**: predicted daily infections from each model through December 31. **F**: predicted daily deaths from each model through December 31. **G**: predicted cumulative infections through December 31, as a proportion of the total population. **H**: predicted cumulative deaths through December 31. In panels E, F, G, and H, reported death and infections are plotted alongside model predictions in light blue.

46 Massachusetts: Covariate fits and regression coefficients

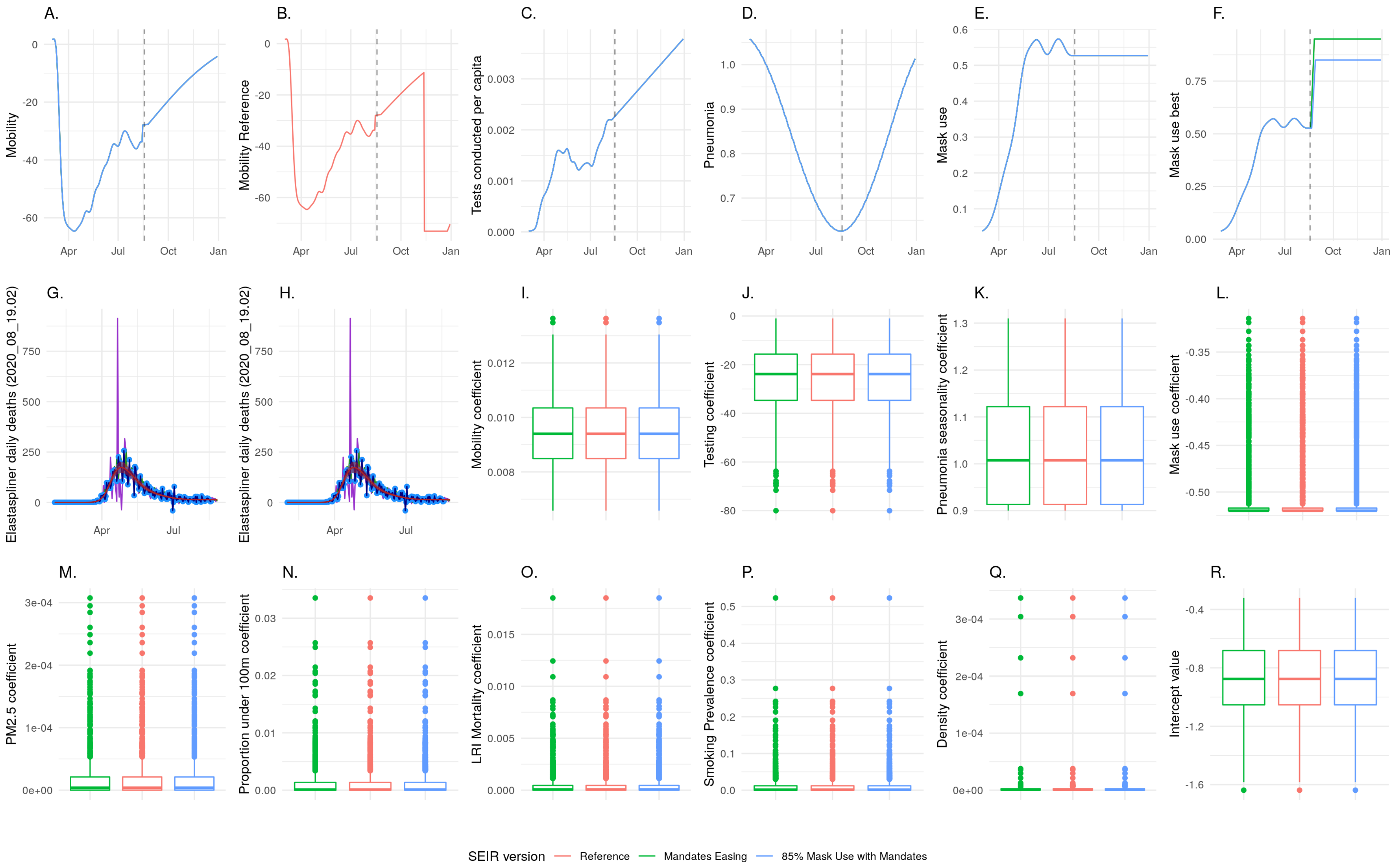

Massachusetts: Covariate fits and regression coefficients. A-F: Line plots showing predicted covariate time trends for **A)** mobility in the absence of additional mandates; **B)** mobility with additional mandates applied; **C)** diagnostic testing per capita; **D)** pneumonia seasonality; **E)** mask use per capita, and; **F)** mask use in a scenario where adherence increases to 85% of the population. **G-H**: COVID mortality data generated from reported daily deaths (blue); estimated based on reported hospitalizations (purple); estimated from reported cases (green); and via a spline fit through all available data types (red, 95% UI in pink). **I-R**: Box plots showing 1,000 draws of fixed effect coefficients in a multivariate regression fit to $log(beta)$.

47 Michigan: SEIR fit comparison

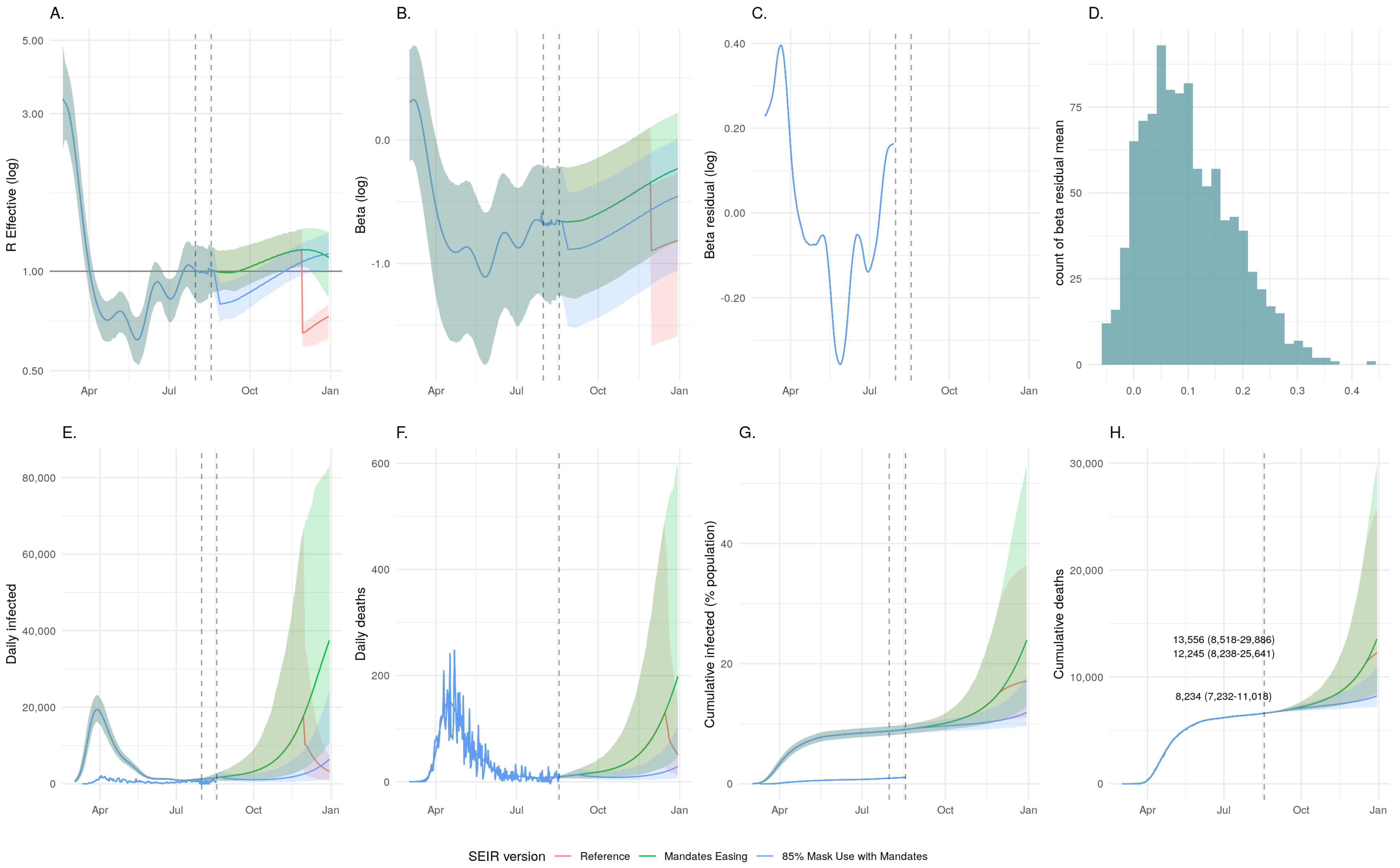

Michigan: SEIR fit comparison. A: predicted R effective for each model through December 31. **B**: predicted SEIR *beta* parameter. **C**: residual of predicted *beta* and the observed value calculated directly from infection data over time. **D**: histogram of residual values for *beta*. Panels A, B, C, and D are all displayed in log space, reflecting the space in which the SEIR model is fit. **E**: predicted daily infections from each model through December 31. **F**: predicted daily deaths from each model through December 31. **G**: predicted cumulative infections through December 31, as a proportion of the total population. **H**: predicted cumulative deaths through December 31. In panels E, F, G, and H, reported death and infections are plotted alongside model predictions in light blue.

48 Michigan: Covariate fits and regression coefficients

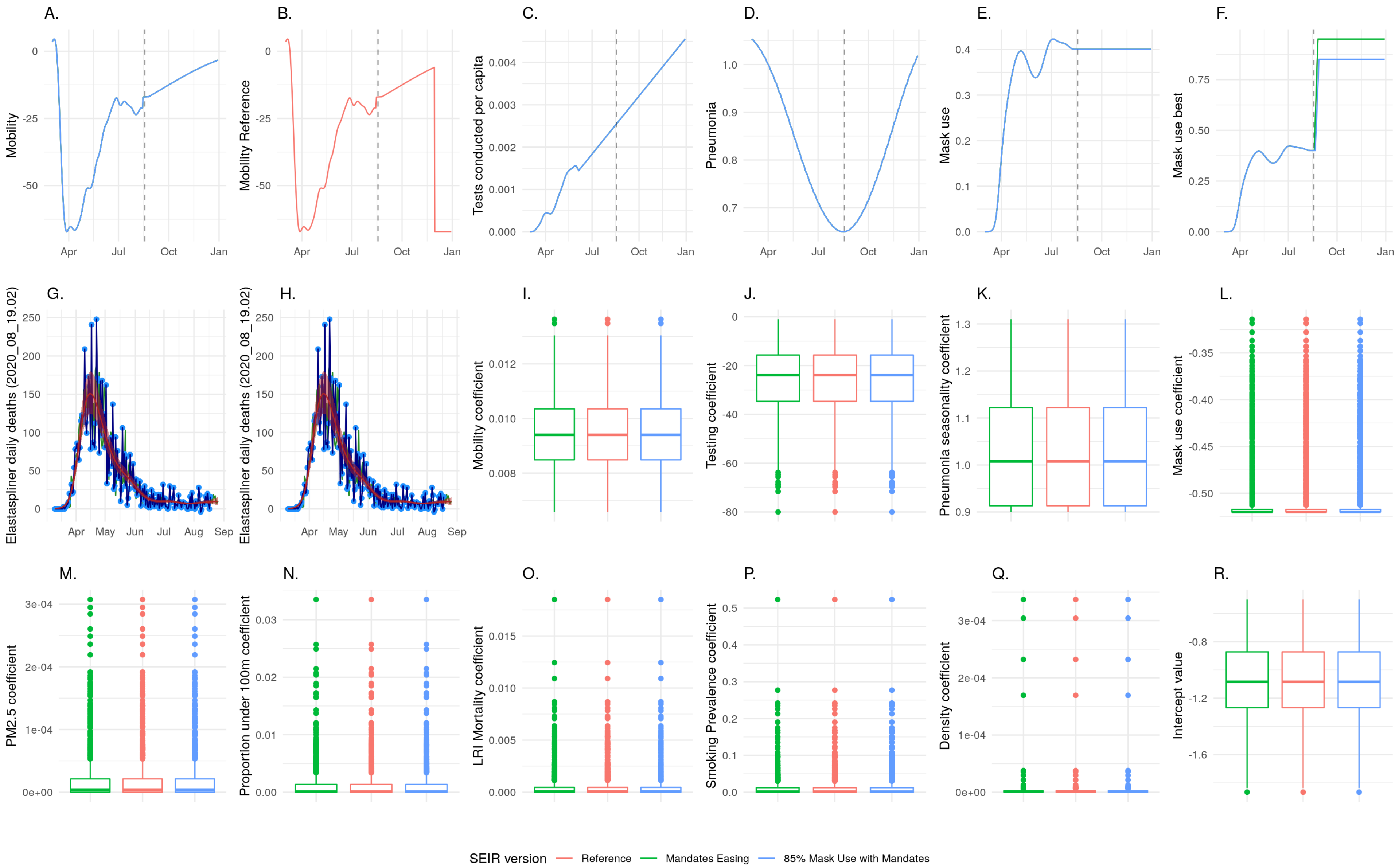

Michigan: Covariate fits and regression coefficients. A-F: Line plots showing predicted covariate time trends for **A)** mobility in the absence of additional mandates; **B)** mobility with additional mandates applied; **C)** diagnostic testing per capita; **D)** pneumonia seasonality; **E)** mask use per capita, and; **F)** mask use in a scenario where adherence increases to 85% of the population. **G-H**: COVID mortality data generated from reported daily deaths (blue); estimated based on reported hospitalizations (purple); estimated from reported cases (green); and via a spline fit through all available data types (red, 95% UI in pink). **I-R**: Box plots showing 1,000 draws of fixed effect coefficients in a multivariate regression fit to $log(beta)$.

49 Minnesota: SEIR fit comparison

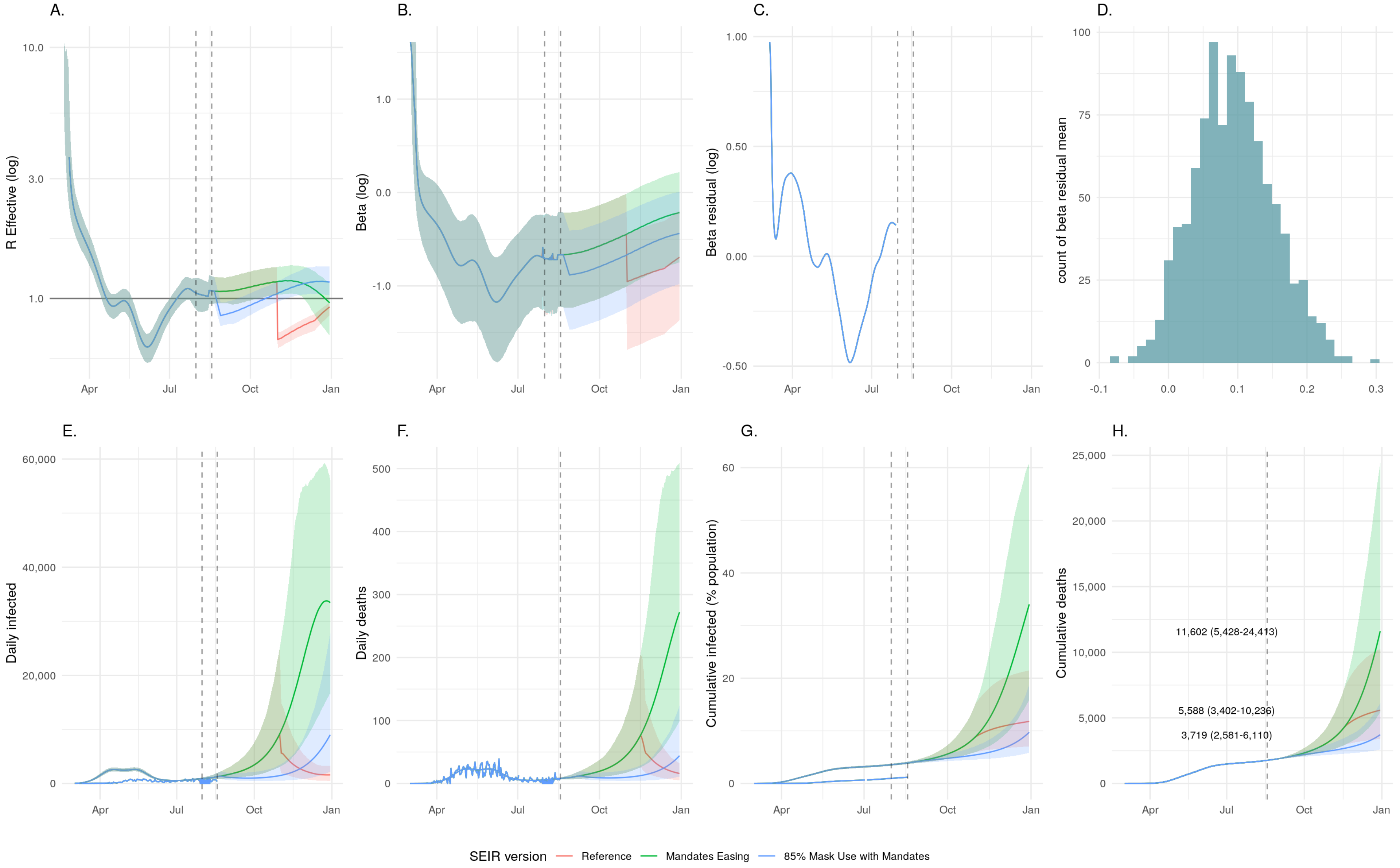

Minnesota: SEIR fit comparison. A: predicted R effective for each model through December 31. **B**: predicted SEIR *beta* parameter. **C**: residual of predicted *beta* and the observed value calculated directly from infection data over time. **D**: histogram of residual values for *beta*. Panels A, B, C, and D are all displayed in log space, reflecting the space in which the SEIR model is fit. **E**: predicted daily infections from each model through December 31. **F**: predicted daily deaths from each model through December 31. **G**: predicted cumulative infections through December 31, as a proportion of the total population. **H**: predicted cumulative deaths through December 31. In panels E, F, G, and H, reported death and infections are plotted alongside model predictions in light blue.

50 Minnesota: Covariate fits and regression coefficients

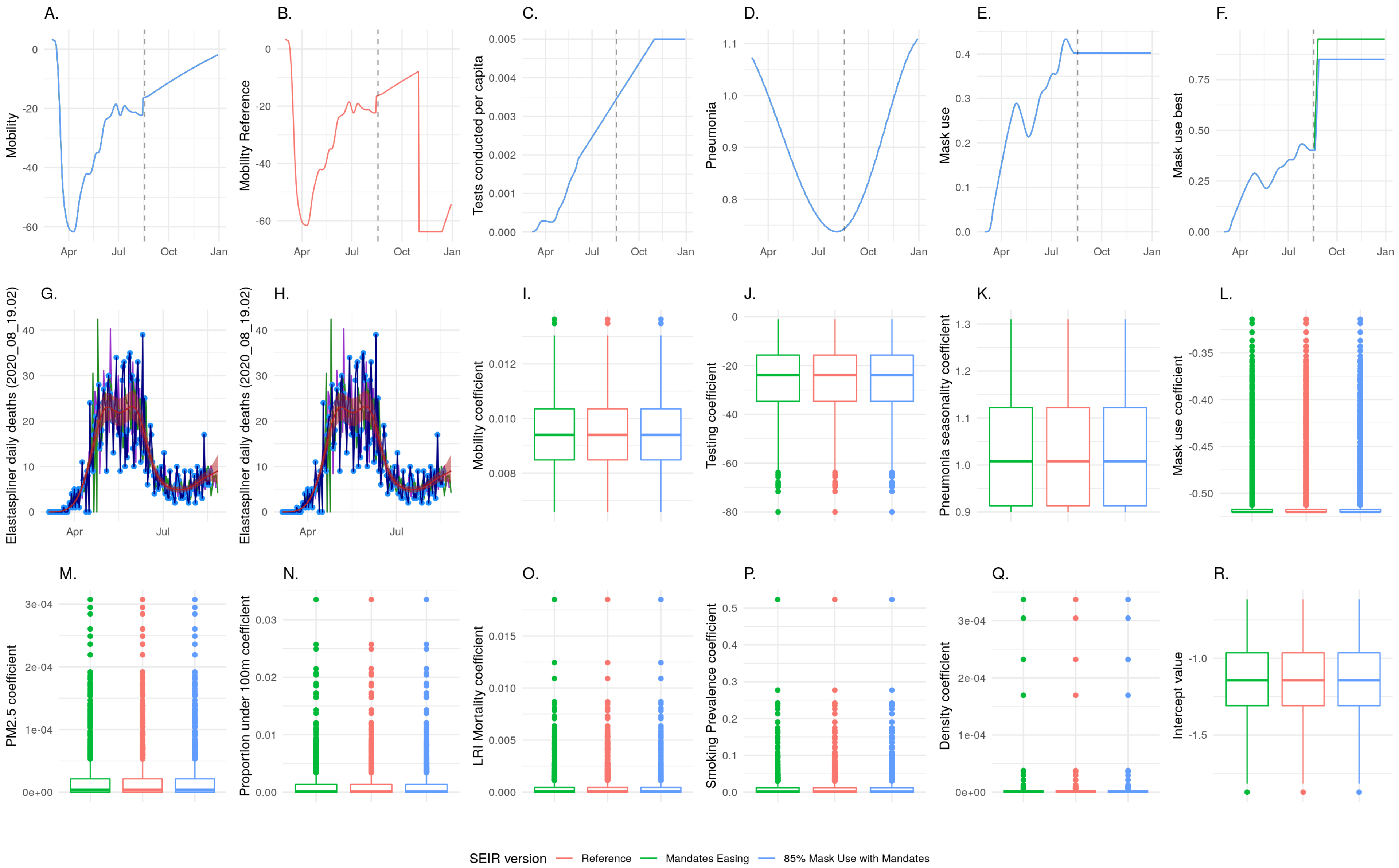

Minnesota: Covariate fits and regression coefficients. **A-F**: Line plots showing predicted covariate time trends for **A)** mobility in the absence of additional mandates; **B)** mobility with additional mandates applied; **C)** diagnostic testing per capita; **D)** pneumonia seasonality; **E)** mask use per capita, and; **F)** mask use in a scenario where adherence increases to 85% of the population. **G-H**: COVID mortality data generated from reported daily deaths (blue); estimated based on reported hospitalizations (purple); estimated from reported cases (green); and via a spline fit through all available data types (red, 95% UI in pink). **I-R**: Box plots showing 1,000 draws of fixed effect coefficients in a multivariate regression fit to $log(beta)$.

51 Mississippi: SEIR fit comparison

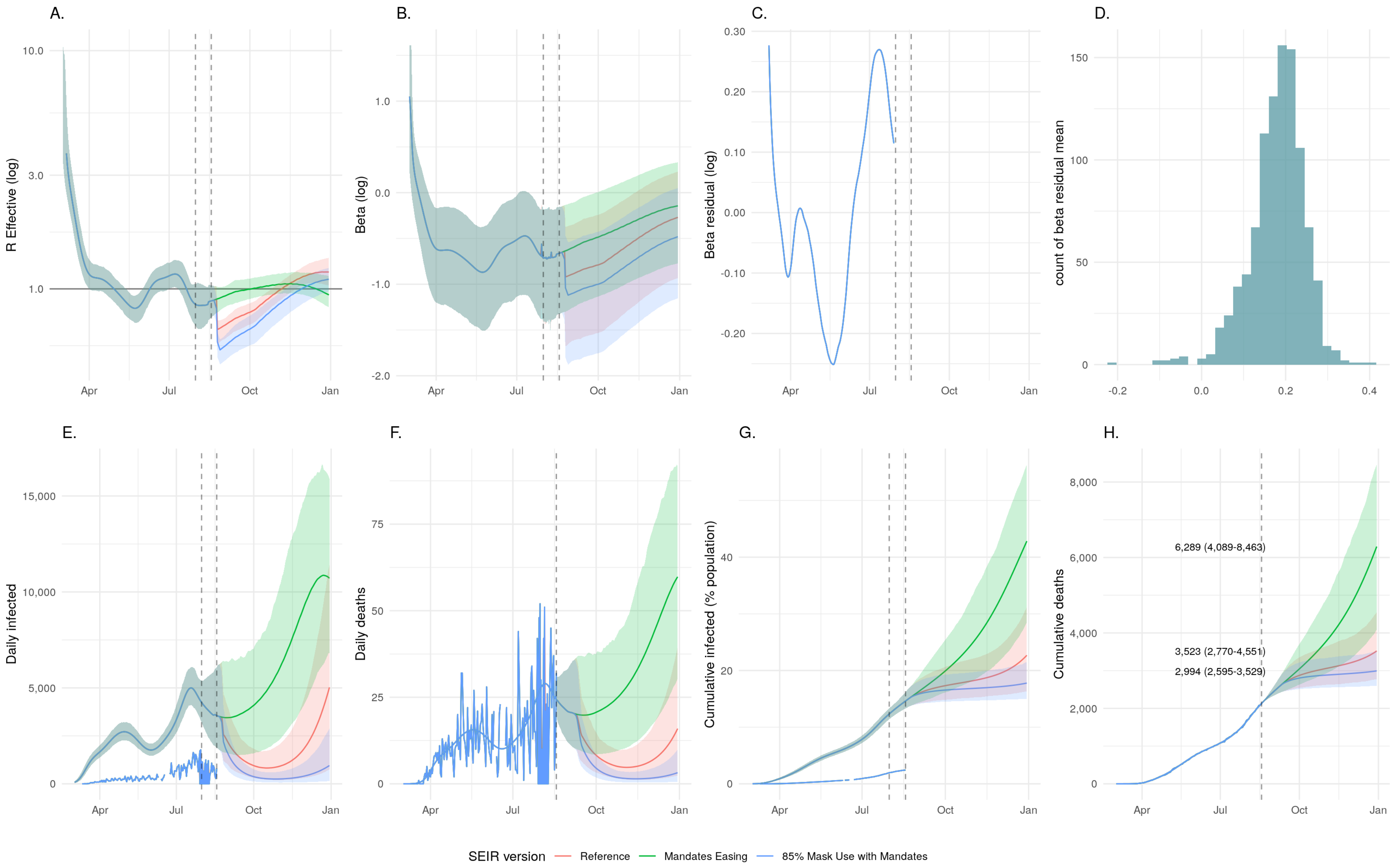

Mississippi: SEIR fit comparison. A: predicted R effective for each model through December 31. **B**: predicted SEIR *beta* parameter. **C**: residual of predicted *beta* and the observed value calculated directly from infection data over time. **D**: histogram of residual values for *beta*. Panels A, B, C, and D are all displayed in log space, reflecting the space in which the SEIR model is fit. **E**: predicted daily infections from each model through December 31. **F**: predicted daily deaths from each model through December 31. **G**: predicted cumulative infections through December 31, as a proportion of the total population. **H**: predicted cumulative deaths through December 31. In panels E, F, G, and H, reported death and infections are plotted alongside model predictions in light blue.

52 Mississippi: Covariate fits and regression coefficients

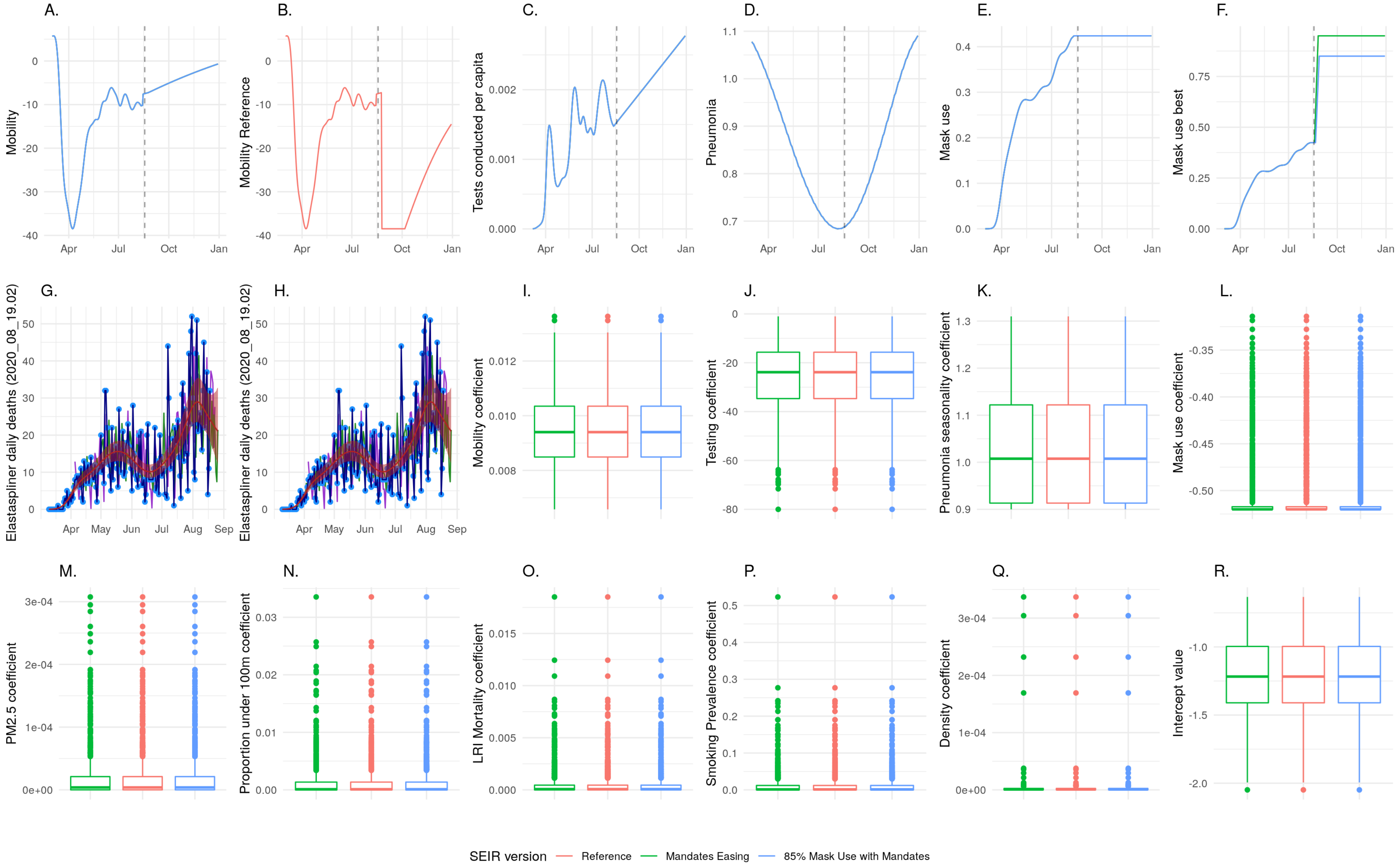

Mississippi: Covariate fits and regression coefficients. A-F: Line plots showing predicted covariate time trends for **A)** mobility in the absence of additional mandates; **B)** mobility with additional mandates applied; **C)** diagnostic testing per capita; **D)** pneumonia seasonality; **E)** mask use per capita, and; **F)** mask use in a scenario where adherence increases to 85% of the population. **G-H**: COVID mortality data generated from reported daily deaths (blue); estimated based on reported hospitalizations (purple); estimated from reported cases (green); and via a spline fit through all available data types (red, 95% UI in pink). **I-R**: Box plots showing 1,000 draws of fixed effect coefficients in a multivariate regression fit to $log(beta)$.

53 Missouri: SEIR fit comparison

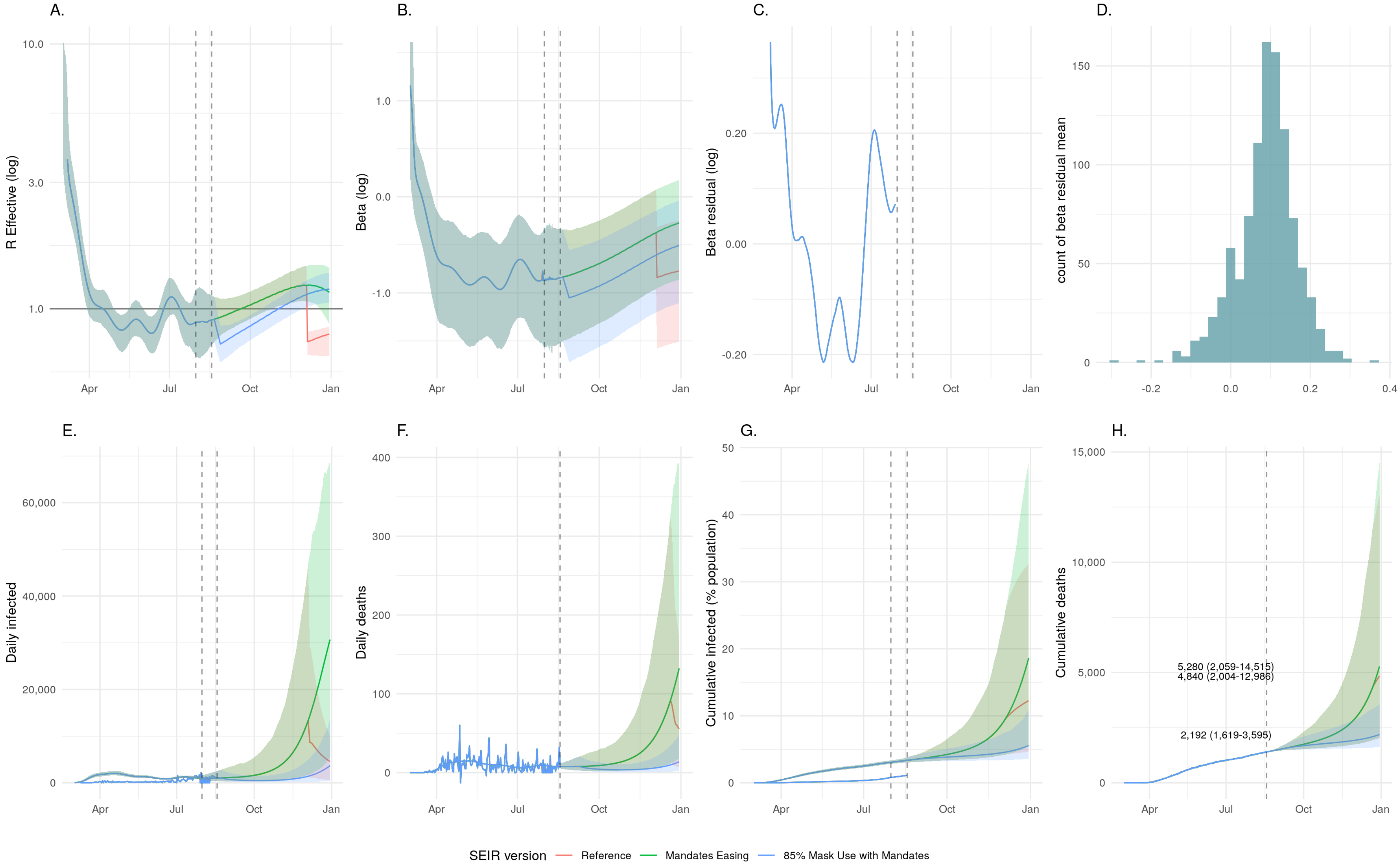

Missouri: SEIR fit comparison. A: predicted R effective for each model through December 31. **B**: predicted SEIR *beta* parameter. **C**: residual of predicted *beta* and the observed value calculated directly from infection data over time. **D**: histogram of residual values for *beta*. Panels A, B, C, and D are all displayed in log space, reflecting the space in which the SEIR model is fit. **E**: predicted daily infections from each model through December 31. **F**: predicted daily deaths from each model through December 31. **G**: predicted cumulative infections through December 31, as a proportion of the total population. **H**: predicted cumulative deaths through December 31. In panels E, F, G, and H, reported death and infections are plotted alongside model predictions in light blue.

54 Missouri: Covariate fits and regression coefficients

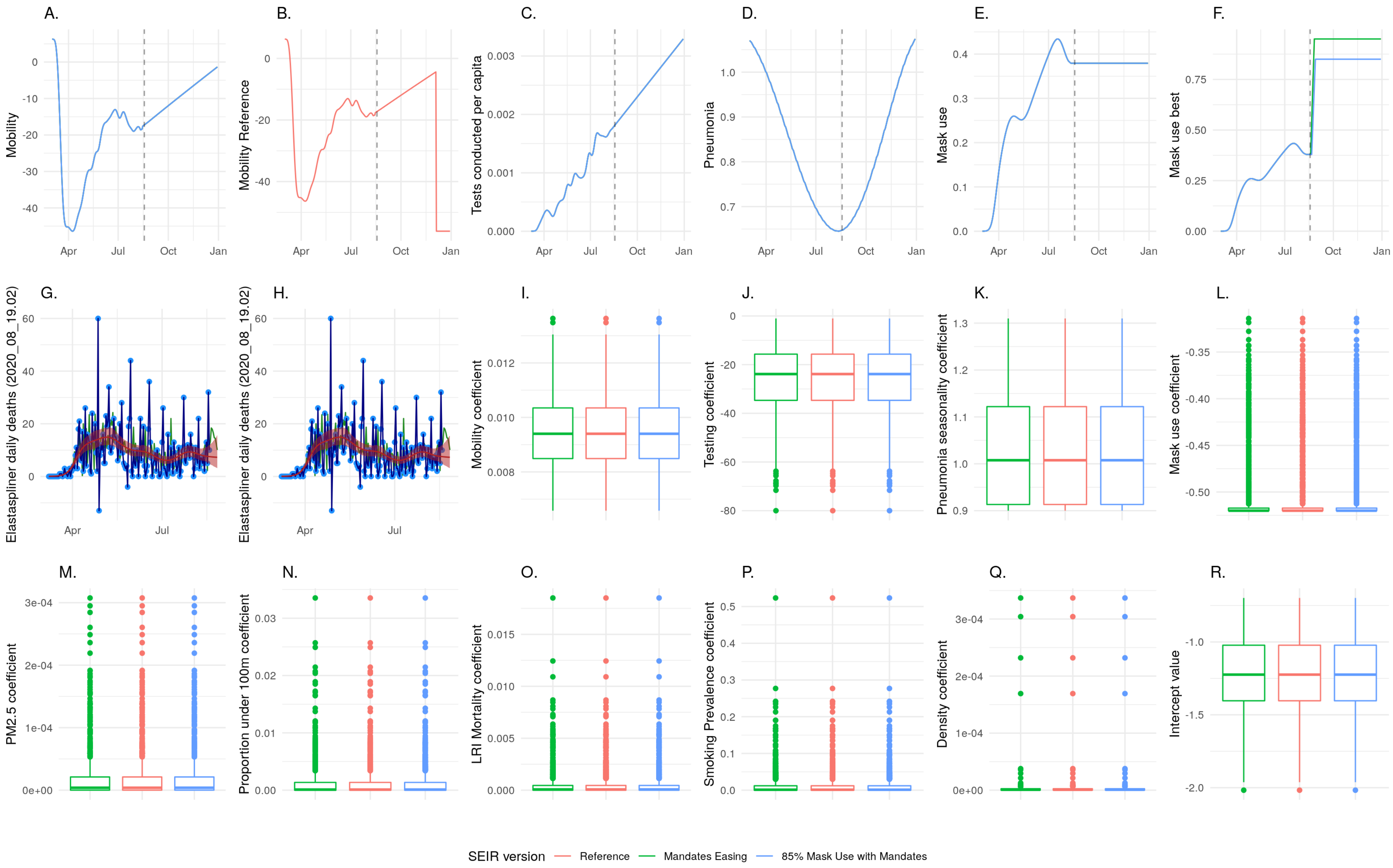

Missouri: Covariate fits and regression coefficients. A-F: Line plots showing predicted covariate time trends for **A)** mobility in the absence of additional mandates; **B)** mobility with additional mandates applied; **C)** diagnostic testing per capita; **D)** pneumonia seasonality; **E)** mask use per capita, and; **F)** mask use in a scenario where adherence increases to 85% of the population. **G-H**: COVID mortality data generated from reported daily deaths (blue); estimated based on reported hospitalizations (purple); estimated from reported cases (green); and via a spline fit through all available data types (red, 95% UI in pink). **I-R**: Box plots showing 1,000 draws of fixed effect coefficients in a multivariate regression fit to $log(beta)$.

55 Montana: SEIR fit comparison

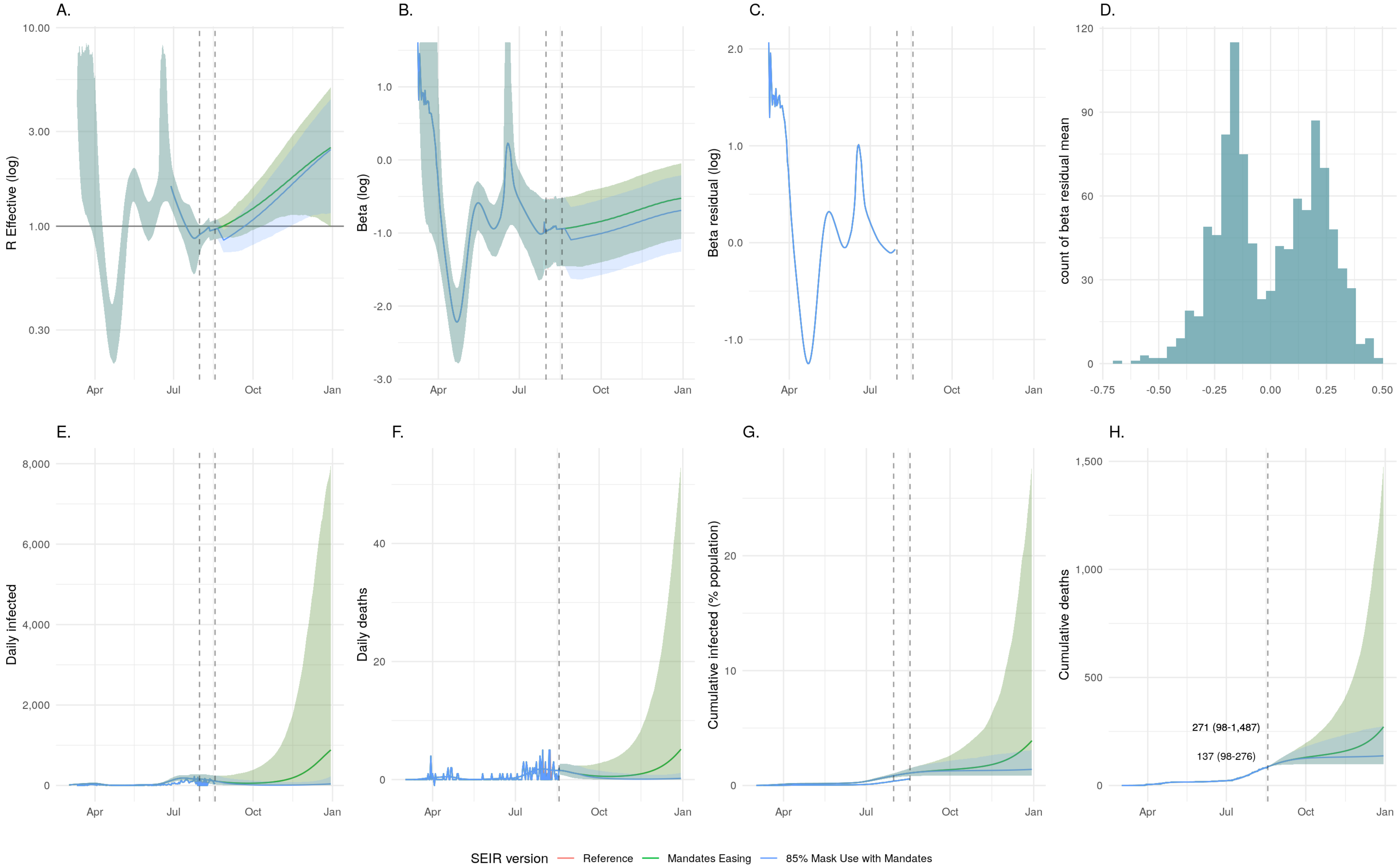

Montana: SEIR fit comparison. **A**: predicted R effective for each model through December 31. **B**: predicted SEIR *beta* parameter. **C**: residual of predicted *beta* and the observed value calculated directly from infection data over time. **D**: histogram of residual values for *beta*. Panels A, B, C, and D are all displayed in log space, reflecting the space in which the SEIR model is fit. **E**: predicted daily infections from each model through December 31. **F**: predicted daily deaths from each model through December 31. **G**: predicted cumulative infections through December 31, as a proportion of the total population. **H**: predicted cumulative deaths through December 31. In panels E, F, G, and H, reported death and infections are plotted alongside model predictions in light blue.

56 Montana: Covariate fits and regression coefficients

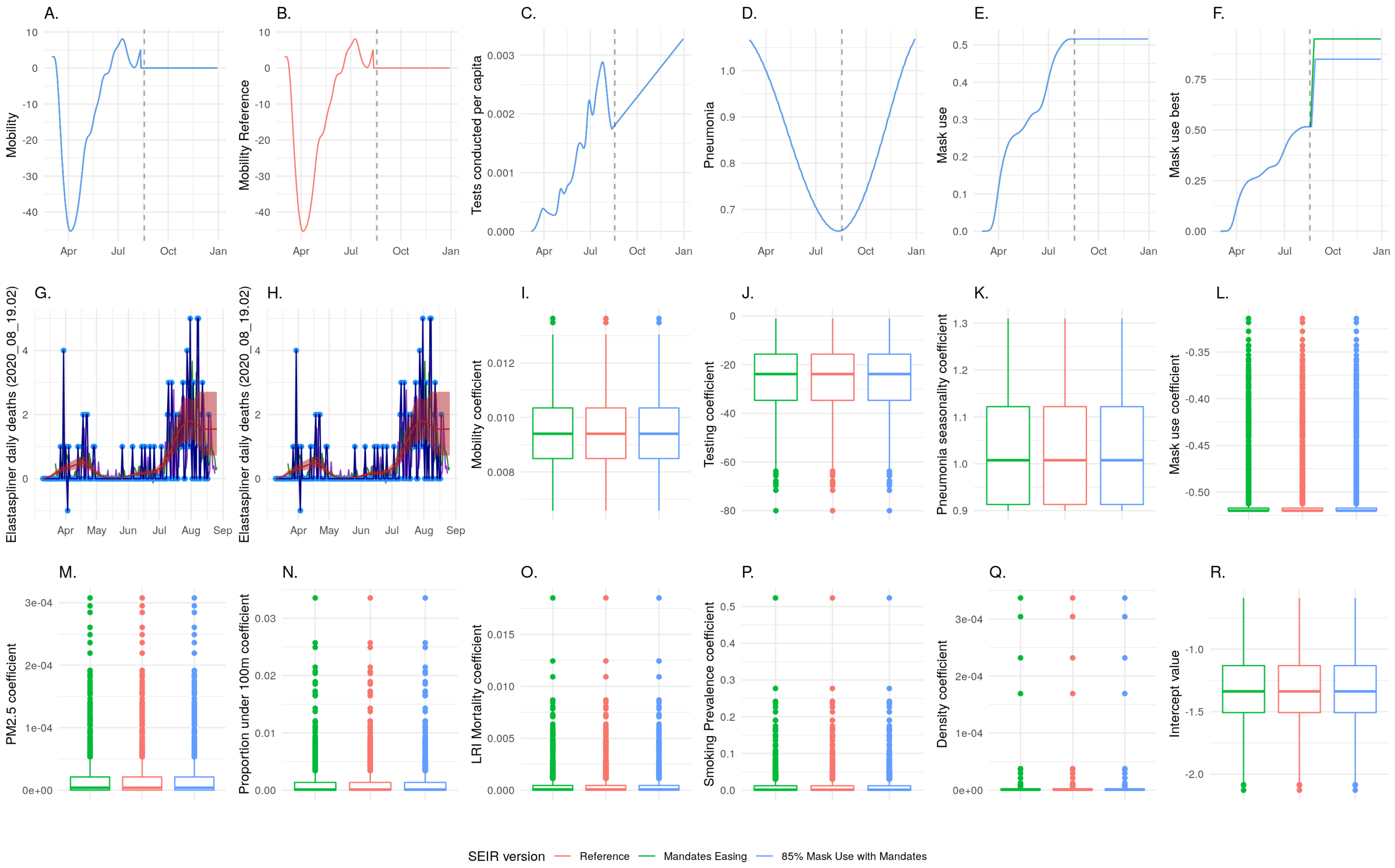

Montana: Covariate fits and regression coefficients. A-F: Line plots showing predicted covariate time trends for **A)** mobility in the absence of additional mandates; **B)** mobility with additional mandates applied; **C)** diagnostic testing per capita; **D)** pneumonia seasonality; **E)** mask use per capita, and; **F)** mask use in a scenario where adherence increases to 85% of the population. **G-H**: COVID mortality data generated from reported daily deaths (blue); estimated based on reported hospitalizations (purple); estimated from reported cases (green); and via a spline fit through all available data types (red, 95% UI in pink). **I-R**: Box plots showing 1,000 draws of fixed effect coefficients in a multivariate regression fit to $log(beta)$.

57 Nebraska: SEIR fit comparison

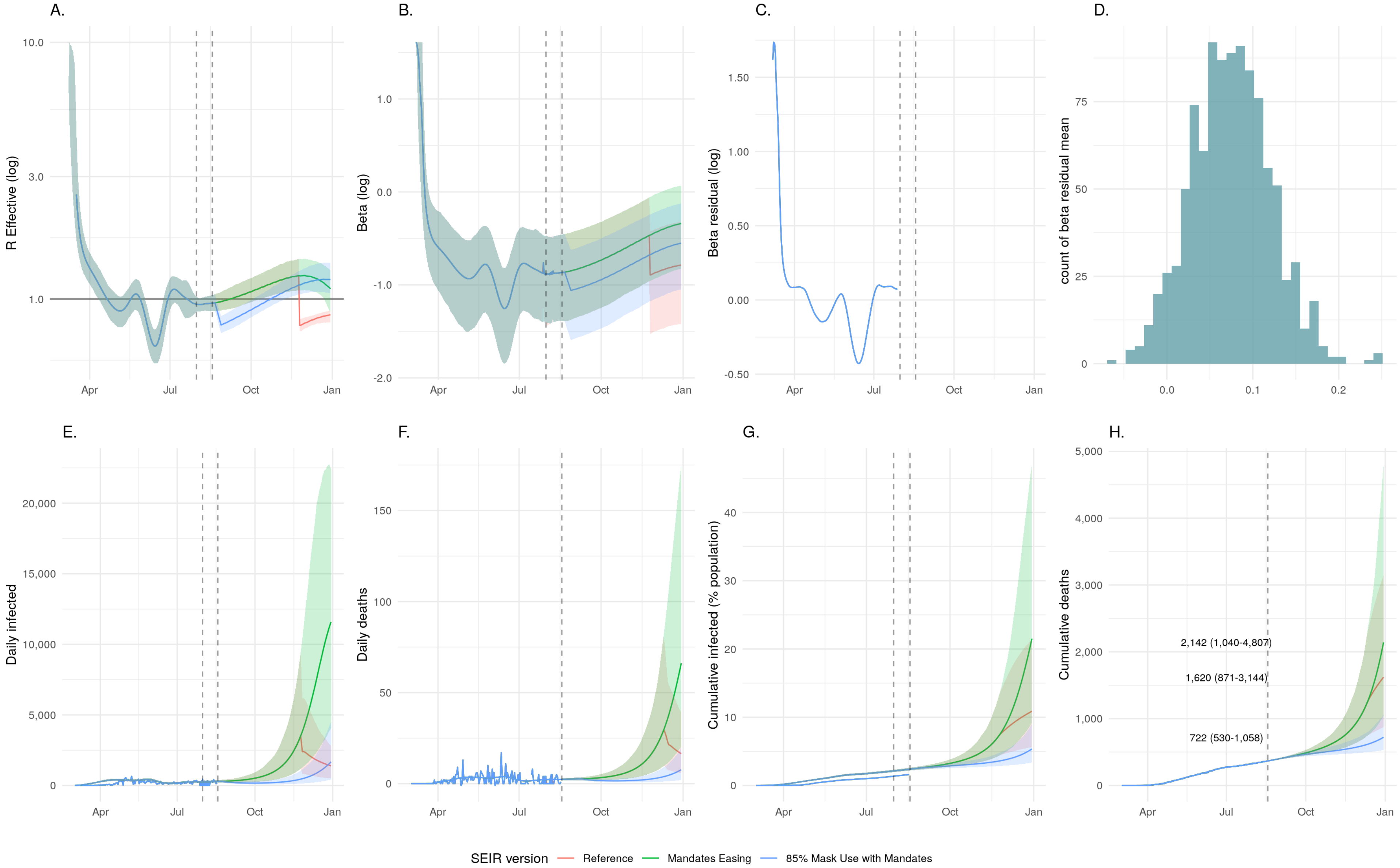

Nebraska: SEIR fit comparison. **A**: predicted R effective for each model through December 31. **B**: predicted SEIR *beta* parameter. **C**: residual of predicted *beta* and the observed value calculated directly from infection data over time. **D**: histogram of residual values for *beta*. Panels A, B, C, and D are all displayed in log space, reflecting the space in which the SEIR model is fit. **E**: predicted daily infections from each model through December 31. **F**: predicted daily deaths from each model through December 31. **G**: predicted cumulative infections through December 31, as a proportion of the total population. **H**: predicted cumulative deaths through December 31. In panels E, F, G, and H, reported death and infections are plotted alongside model predictions in light blue.

58 Nebraska: Covariate fits and regression coefficients

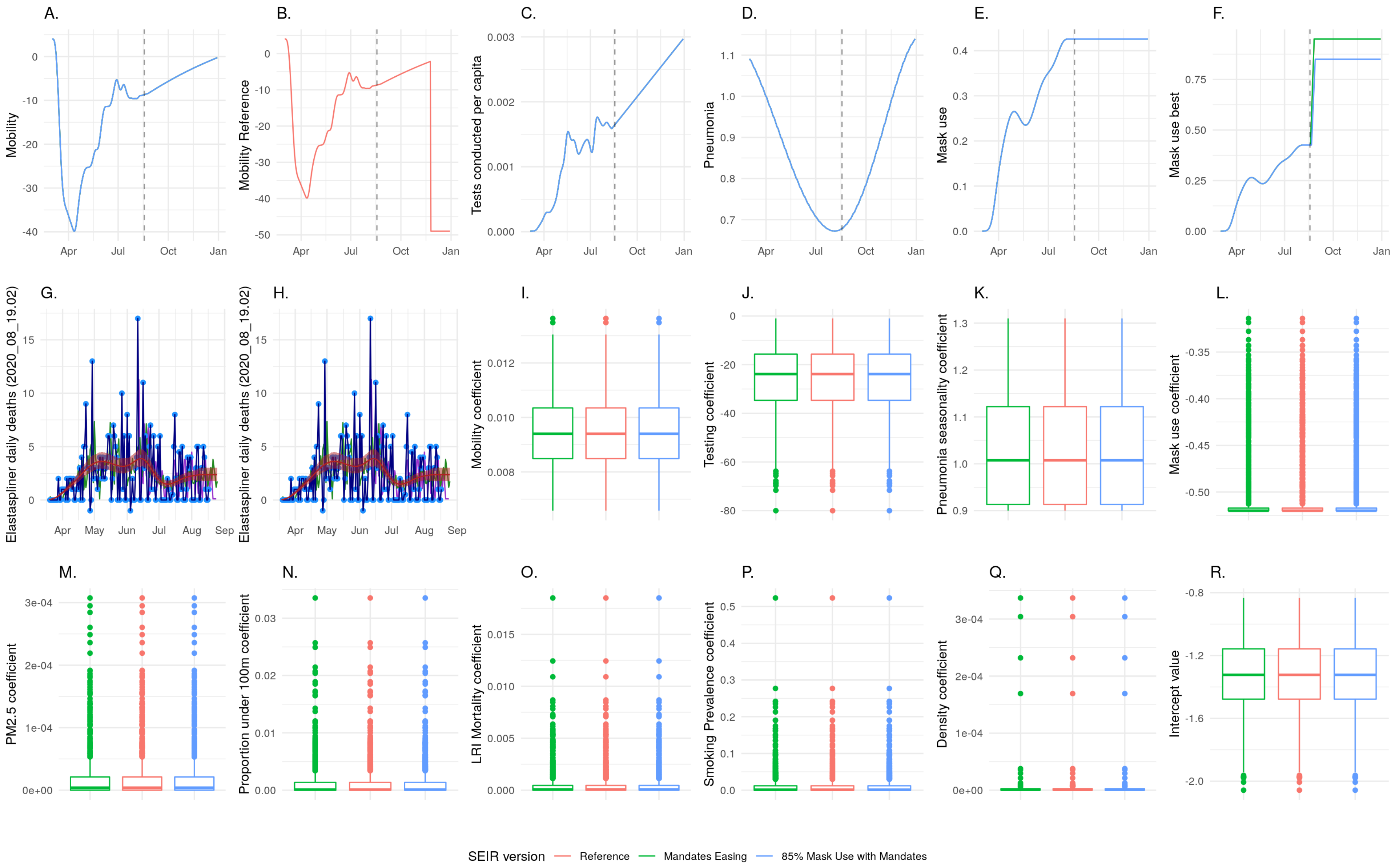

Nebraska: Covariate fits and regression coefficients. **A-F**: Line plots showing predicted covariate time trends for **A)** mobility in the absence of additional mandates; **B)** mobility with additional mandates applied; **C)** diagnostic testing per capita; **D)** pneumonia seasonality; **E)** mask use per capita, and; **F)** mask use in a scenario where adherence increases to 85% of the population. **G-H**: COVID mortality data generated from reported daily deaths (blue); estimated based on reported hospitalizations (purple); estimated from reported cases (green); and via a spline fit through all available data types (red, 95% UI in pink). **I-R**: Box plots showing 1,000 draws of fixed effect coefficients in a multivariate regression fit to $log(beta)$.

59 Nevada: SEIR fit comparison

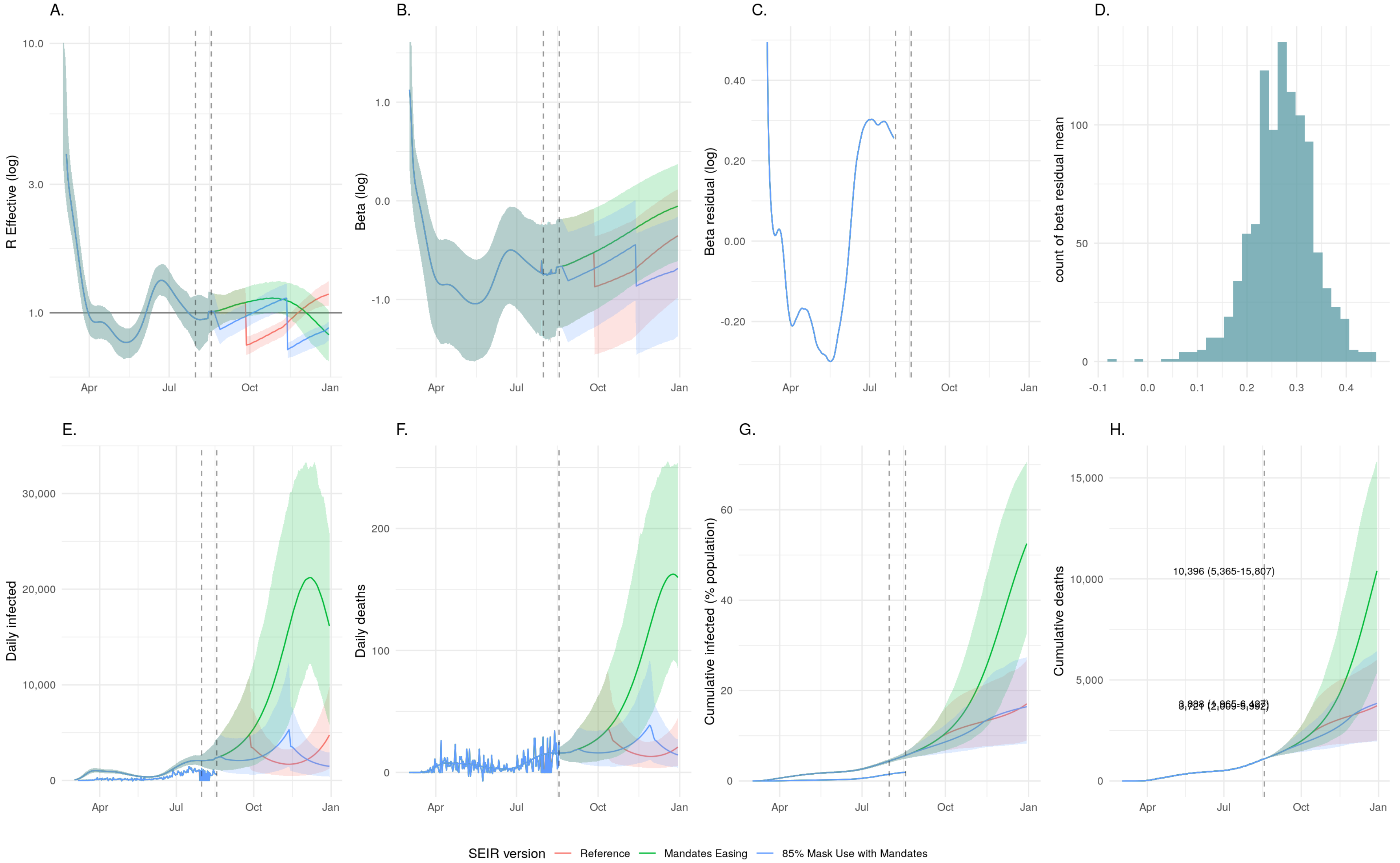

Nevada: SEIR fit comparison. **A**: predicted R effective for each model through December 31. **B**: predicted SEIR *beta* parameter. **C**: residual of predicted *beta* and the observed value calculated directly from infection data over time. **D**: histogram of residual values for *beta*. Panels A, B, C, and D are all displayed in log space, reflecting the space in which the SEIR model is fit. **E**: predicted daily infections from each model through December 31. **F**: predicted daily deaths from each model through December 31. **G**: predicted cumulative infections through December 31, as a proportion of the total population. **H**: predicted cumulative deaths through December 31. In panels E, F, G, and H, reported death and infections are plotted alongside model predictions in light blue.

60 Nevada: Covariate fits and regression coefficients

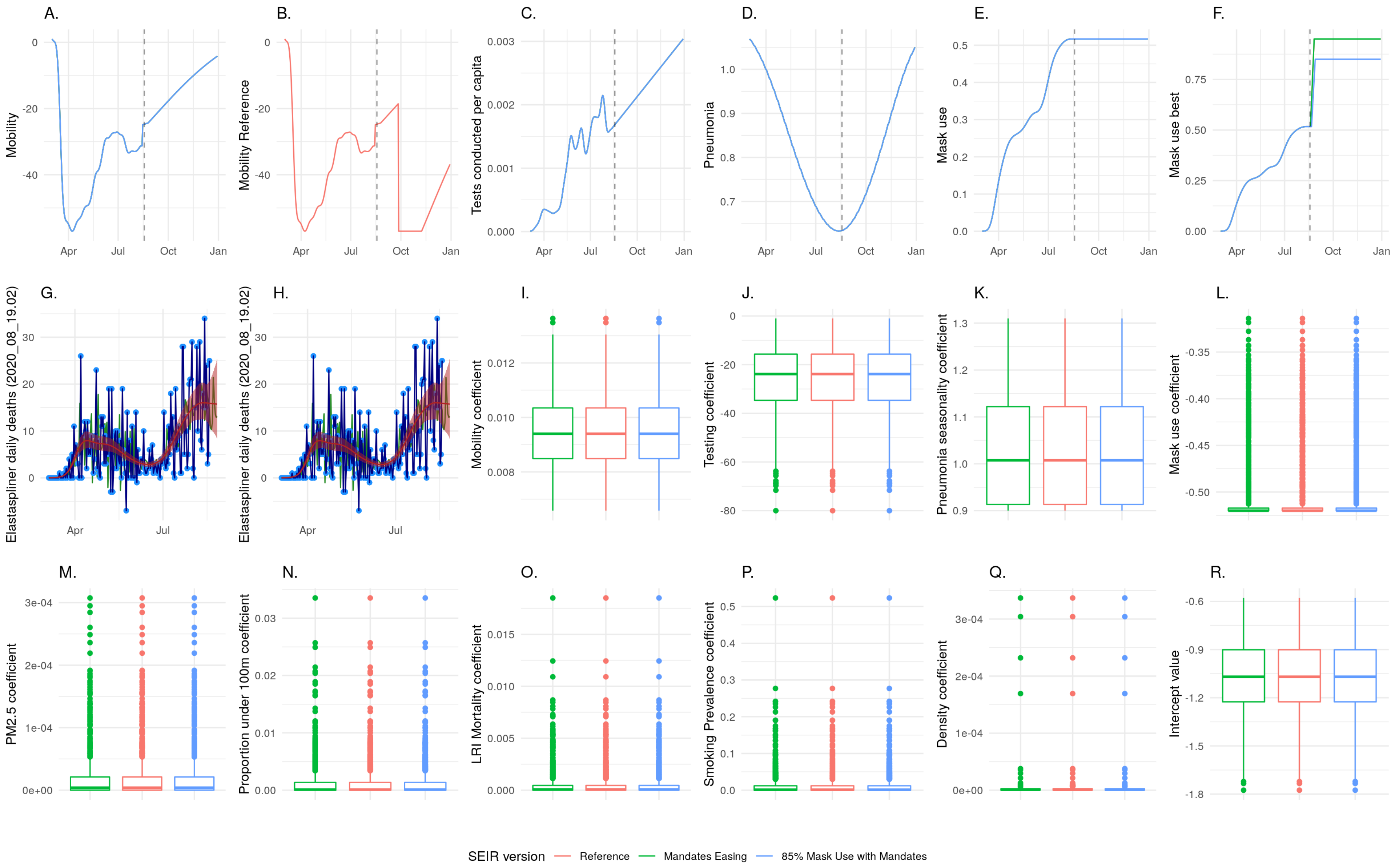

Nevada: Covariate fits and regression coefficients. A-F: Line plots showing predicted covariate time trends for **A)** mobility in the absence of additional mandates; **B)** mobility with additional mandates applied; **C)** diagnostic testing per capita; **D)** pneumonia seasonality; **E)** mask use per capita, and; **F)** mask use in a scenario where adherence increases to 85% of the population. **G-H**: COVID mortality data generated from reported daily deaths (blue); estimated based on reported hospitalizations (purple); estimated from reported cases (green); and via a spline fit through all available data types (red, 95% UI in pink). **I-R**: Box plots showing 1,000 draws of fixed effect coefficients in a multivariate regression fit to $log(beta)$.

61 New Hampshire: SEIR fit comparison

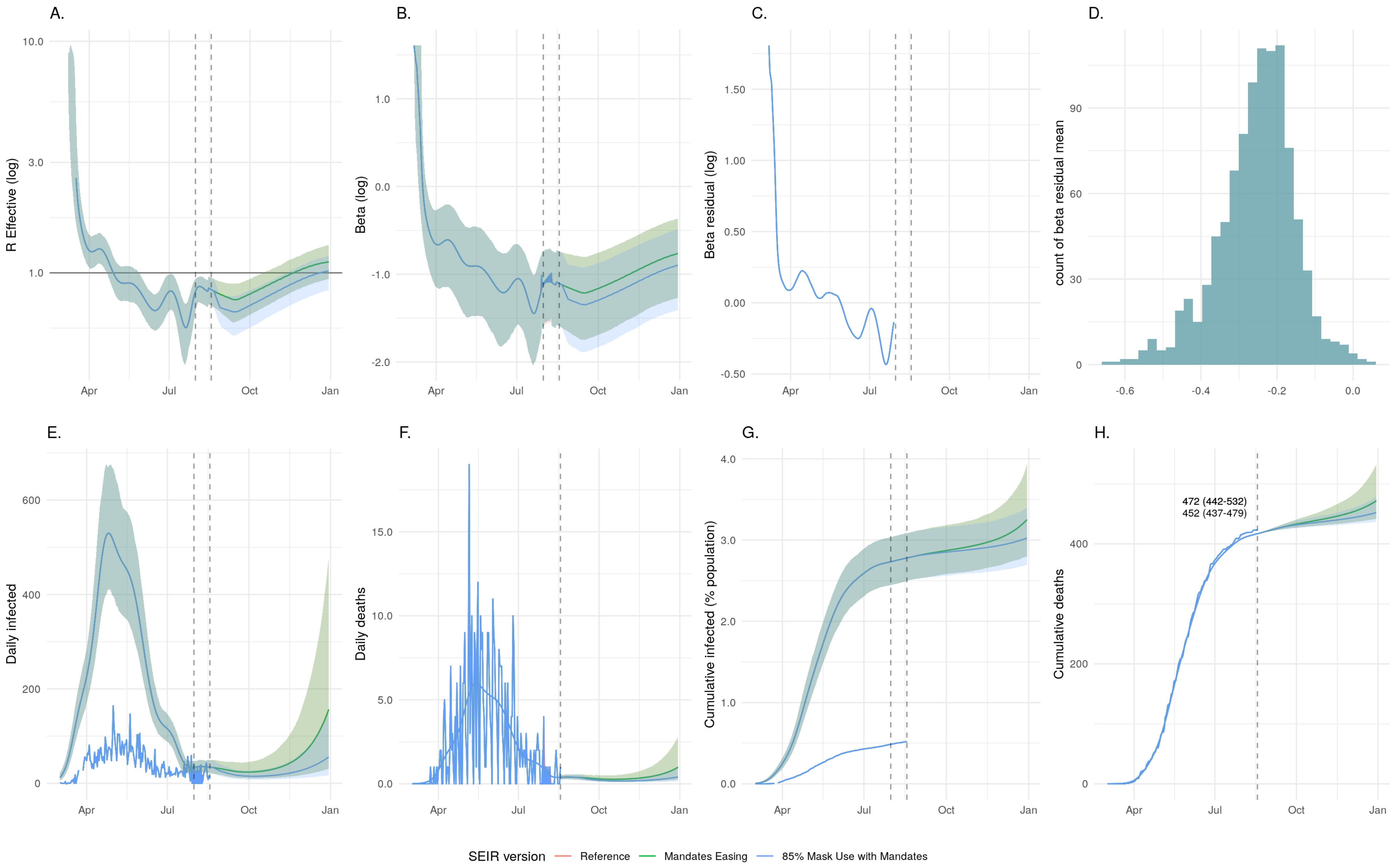

New Hampshire: SEIR fit comparison. **A**: predicted R effective for each model through December 31. **B**: predicted SEIR *beta* parameter. **C**: residual of predicted *beta* and the observed value calculated directly from infection data over time. **D**: histogram of residual values for *beta*. Panels A, B, C, and D are all displayed in log space, reflecting the space in which the SEIR model is fit. **E**: predicted daily infections from each model through December 31. **F**: predicted daily deaths from each model through December 31. **G**: predicted cumulative infections through December 31, as a proportion of the total population. **H**: predicted cumulative deaths through December 31. In panels E, F, G, and H, reported death and infections are plotted alongside model predictions in light blue.

62 New Hampshire: Covariate fits and regression coefficients

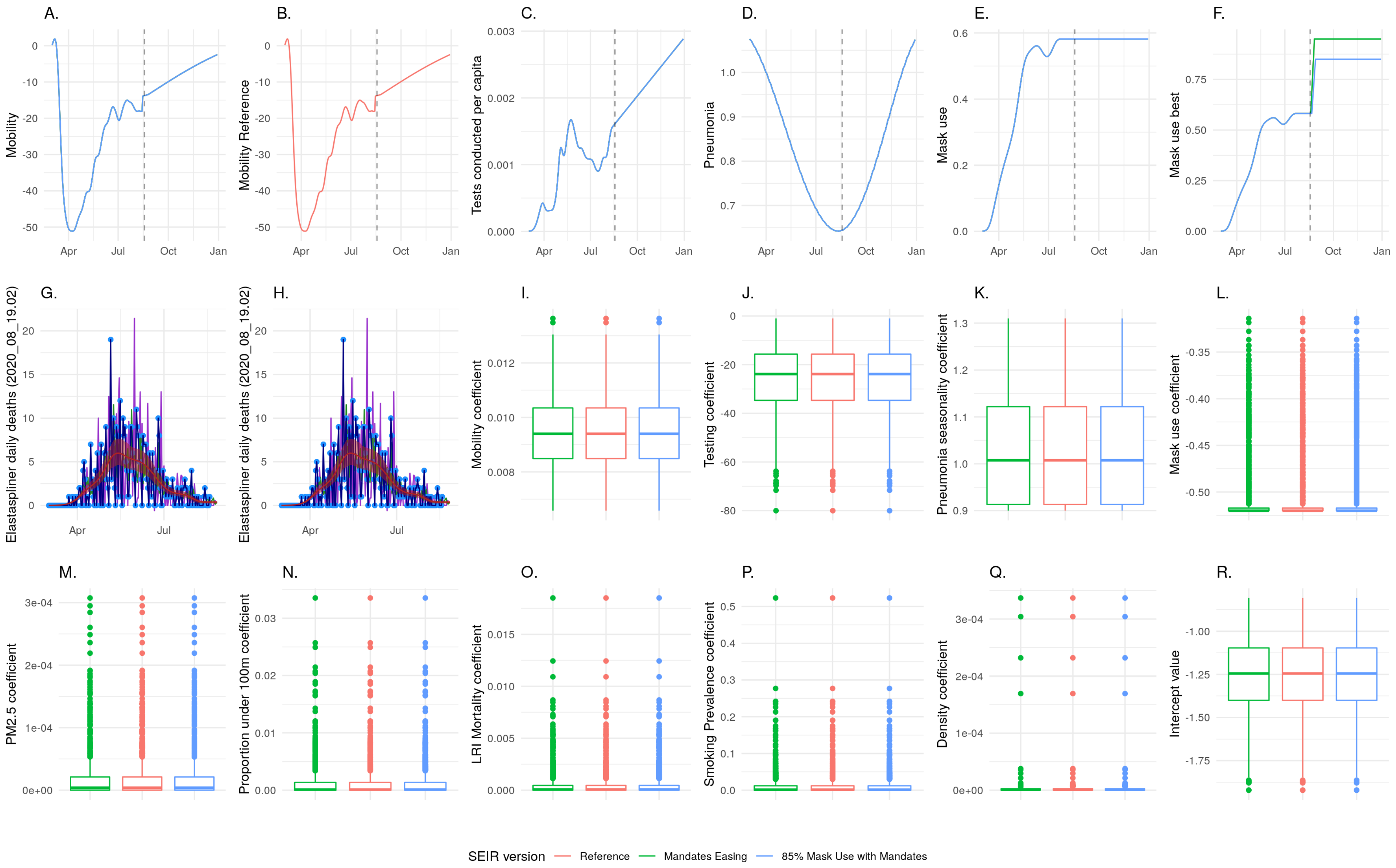

New Hampshire: Covariate fits and regression coefficients. **A-F**: Line plots showing predicted covariate time trends for **A)** mobility in the absence of additional mandates; **B)** mobility with additional mandates applied; **C)** diagnostic testing per capita; **D)** pneumonia seasonality; **E)** mask use per capita, and; **F)** mask use in a scenario where adherence increases to 85% of the population. **G-H**: COVID mortality data generated from reported daily deaths (blue); estimated based on reported hospitalizations (purple); estimated from reported cases (green); and via a spline fit through all available data types (red, 95% UI in pink). **I-R**: Box plots showing 1,000 draws of fixed effect coefficients in a multivariate regression fit to *log*(*beta*).

63 New Jersey: SEIR fit comparison

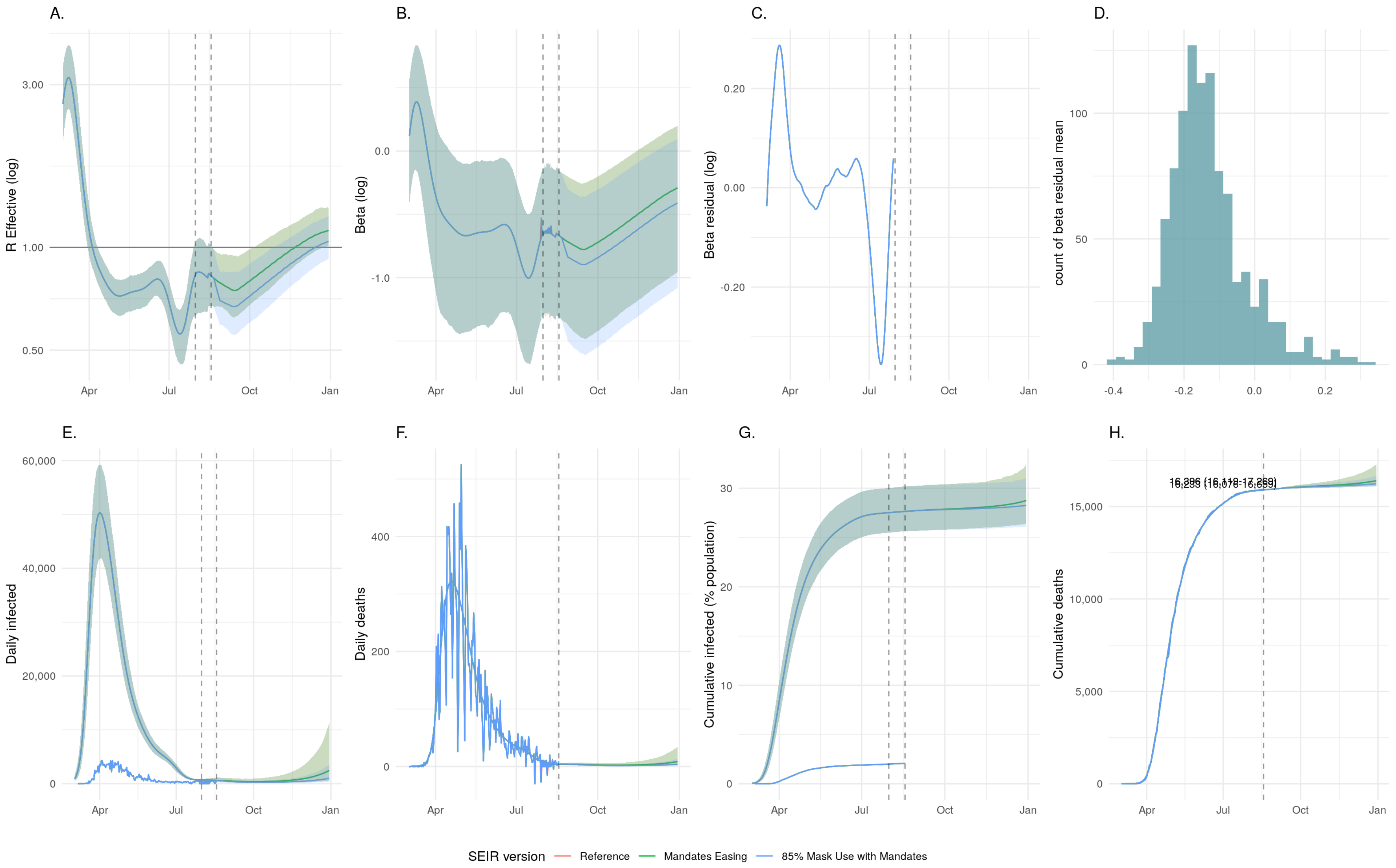

New Jersey: SEIR fit comparison. A: predicted R effective for each model through December 31. **B**: predicted SEIR *beta* parameter. **C**: residual of predicted *beta* and the observed value calculated directly from infection data over time. **D**: histogram of residual values for *beta*. Panels A, B, C, and D are all displayed in log space, reflecting the space in which the SEIR model is fit. **E**: predicted daily infections from each model through December 31. **F**: predicted daily deaths from each model through December 31. **G**: predicted cumulative infections through December 31, as a proportion of the total population. **H**: predicted cumulative deaths through December 31. In panels E, F, G, and H, reported death and infections are plotted alongside model predictions in light blue.

64 New Jersey: Covariate fits and regression coefficients

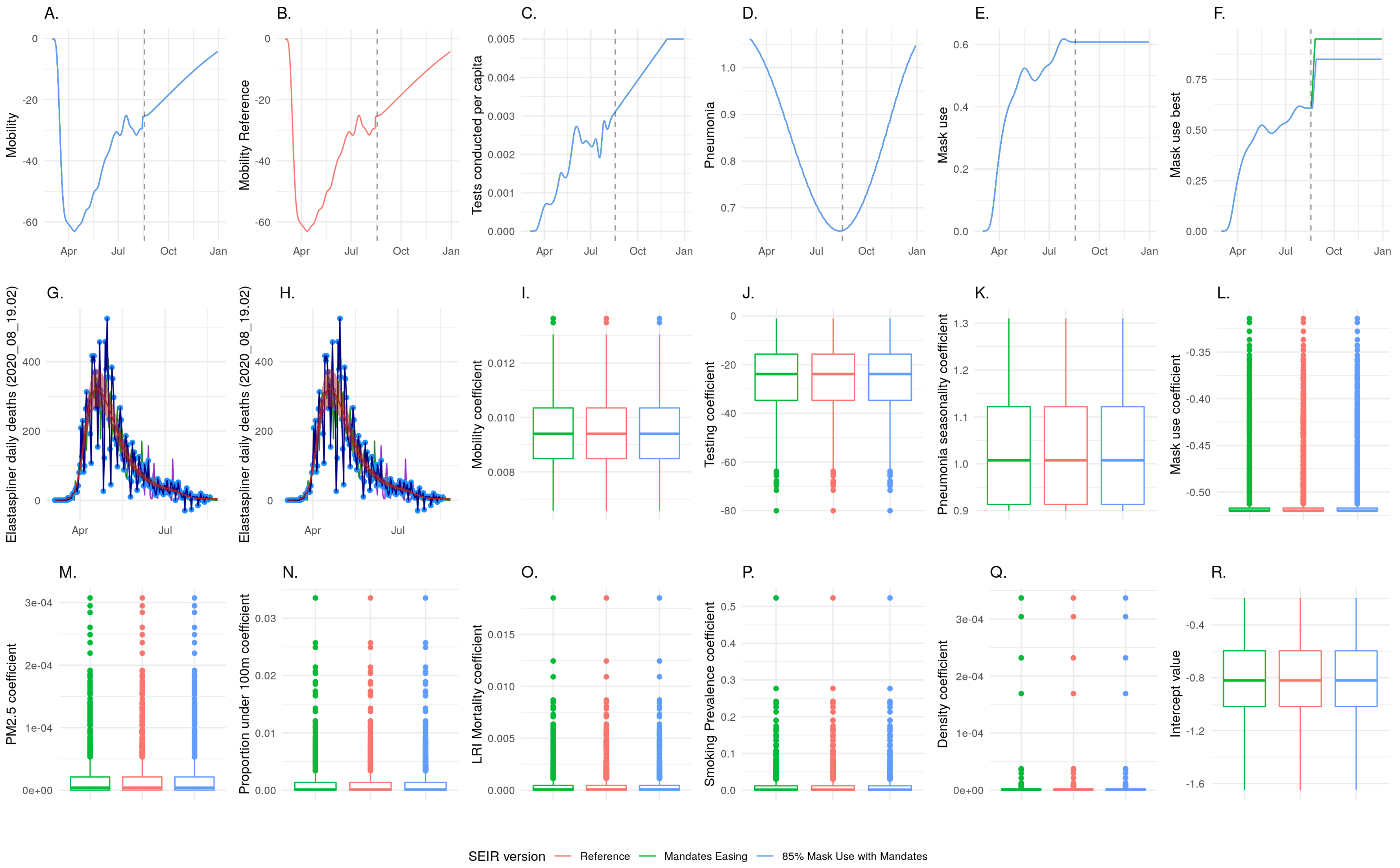

New Jersey: Covariate fits and regression coefficients. A-F: Line plots showing predicted covariate time trends for **A)** mobility in the absence of additional mandates; **B)** mobility with additional mandates applied; **C)** diagnostic testing per capita; **D)** pneumonia seasonality; **E)** mask use per capita, and; **F)** mask use in a scenario where adherence increases to 85% of the population. **G-H**: COVID mortality data generated from reported daily deaths (blue); estimated based on reported hospitalizations (purple); estimated from reported cases (green); and via a spline fit through all available data types (red, 95% UI in pink). **I-R**: Box plots showing 1,000 draws of fixed effect coefficients in a multivariate regression fit to $log(beta)$.

65 New Mexico: SEIR fit comparison

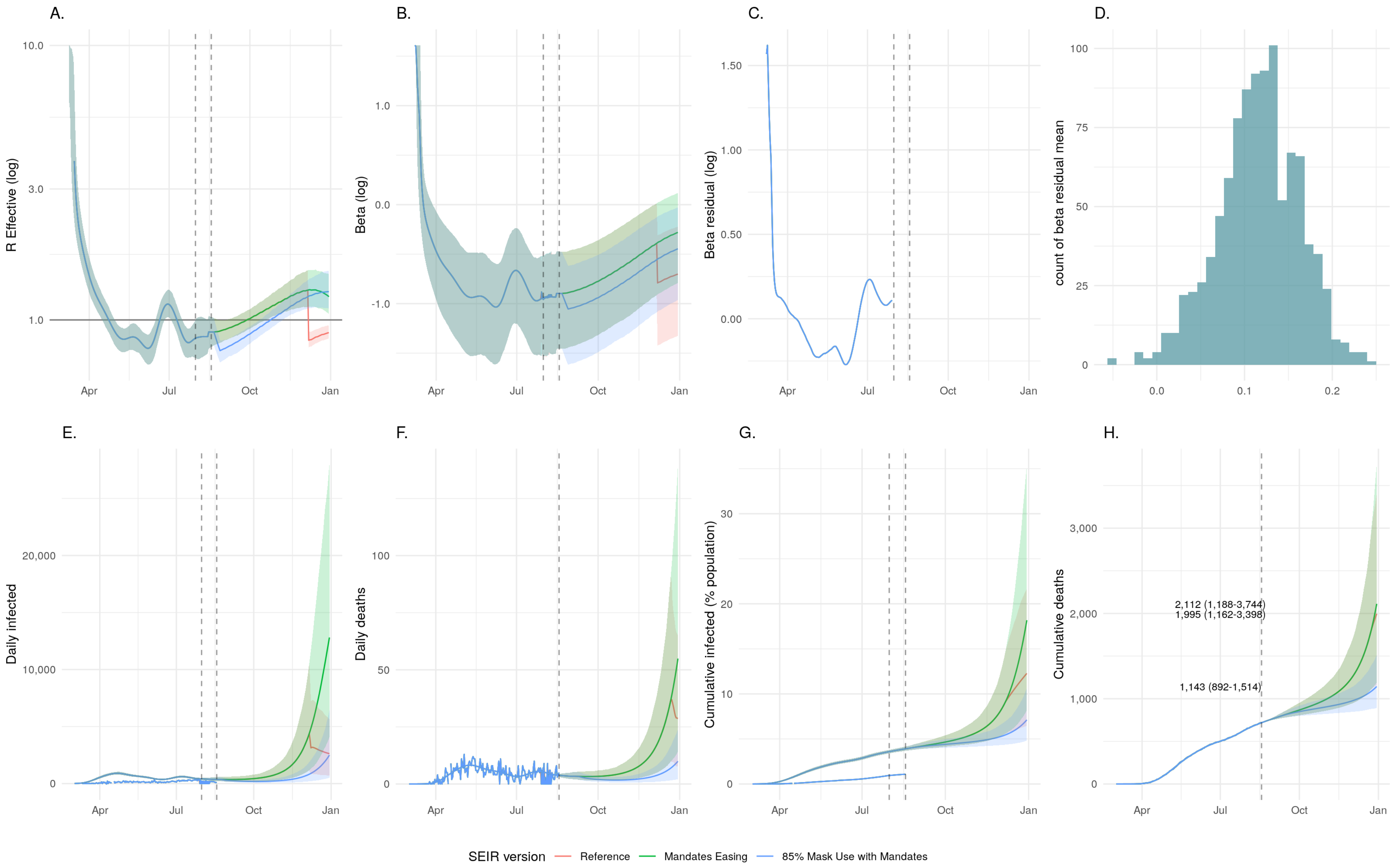

New Mexico: SEIR fit comparison. A: predicted R effective for each model through December 31. **B**: predicted SEIR *beta* parameter. **C**: residual of predicted *beta* and the observed value calculated directly from infection data over time. **D**: histogram of residual values for *beta*. Panels A, B, C, and D are all displayed in log space, reflecting the space in which the SEIR model is fit. **E**: predicted daily infections from each model through December 31. **F**: predicted daily deaths from each model through December 31. **G**: predicted cumulative infections through December 31, as a proportion of the total population. **H**: predicted cumulative deaths through December 31. In panels E, F, G, and H, reported death and infections are plotted alongside model predictions in light blue.

66 New Mexico: Covariate fits and regression coefficients

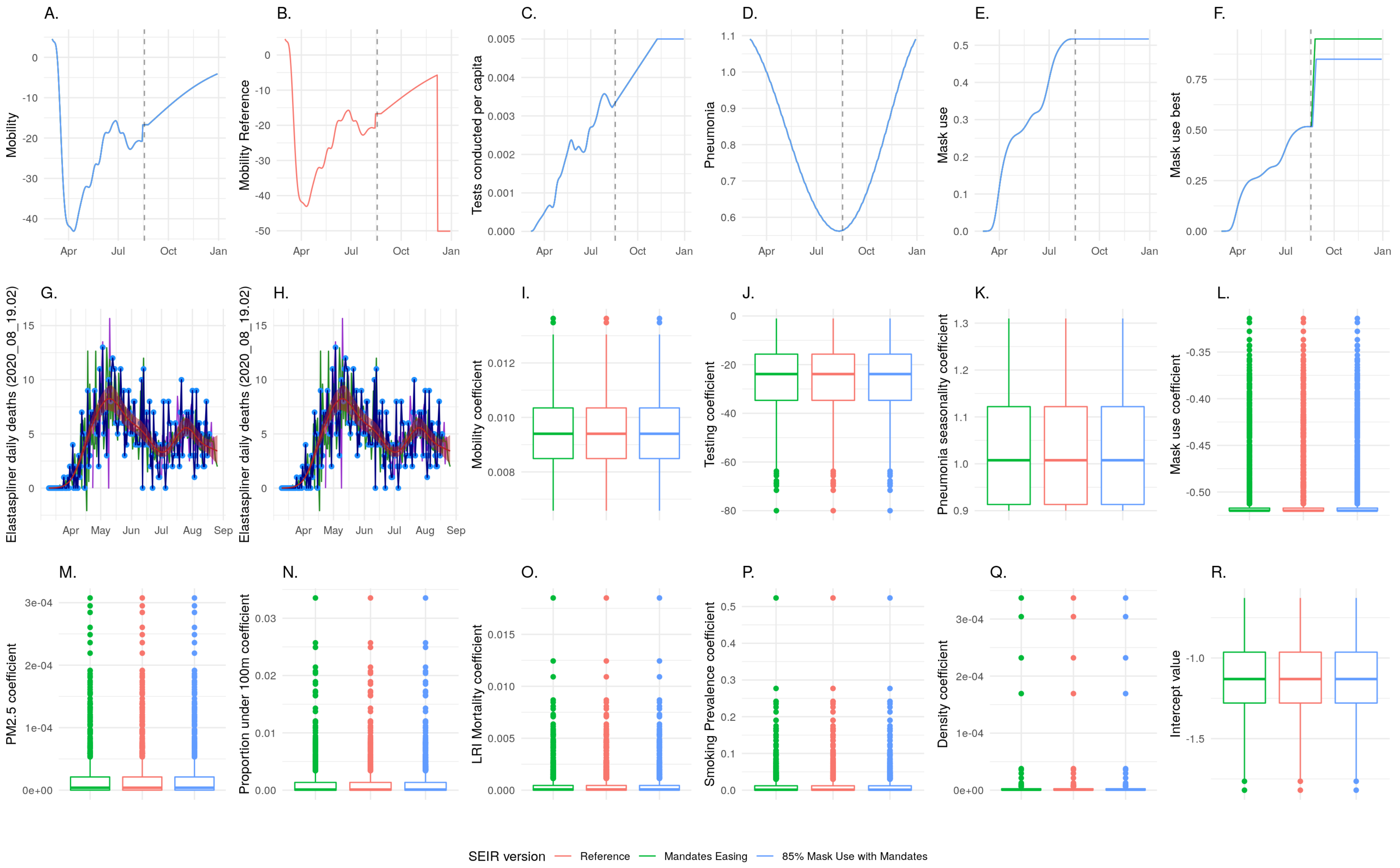

New Mexico: Covariate fits and regression coefficients. A-F: Line plots showing predicted covariate time trends for **A)** mobility in the absence of additional mandates; **B)** mobility with additional mandates applied; **C)** diagnostic testing per capita; **D)** pneumonia seasonality; **E)** mask use per capita, and; **F)** mask use in a scenario where adherence increases to 85% of the population. **G-H**: COVID mortality data generated from reported daily deaths (blue); estimated based on reported hospitalizations (purple); estimated from reported cases (green); and via a spline fit through all available data types (red, 95% UI in pink). **I-R**: Box plots showing 1,000 draws of fixed effect coefficients in a multivariate regression fit to $log(beta)$.

67 New York: SEIR fit comparison

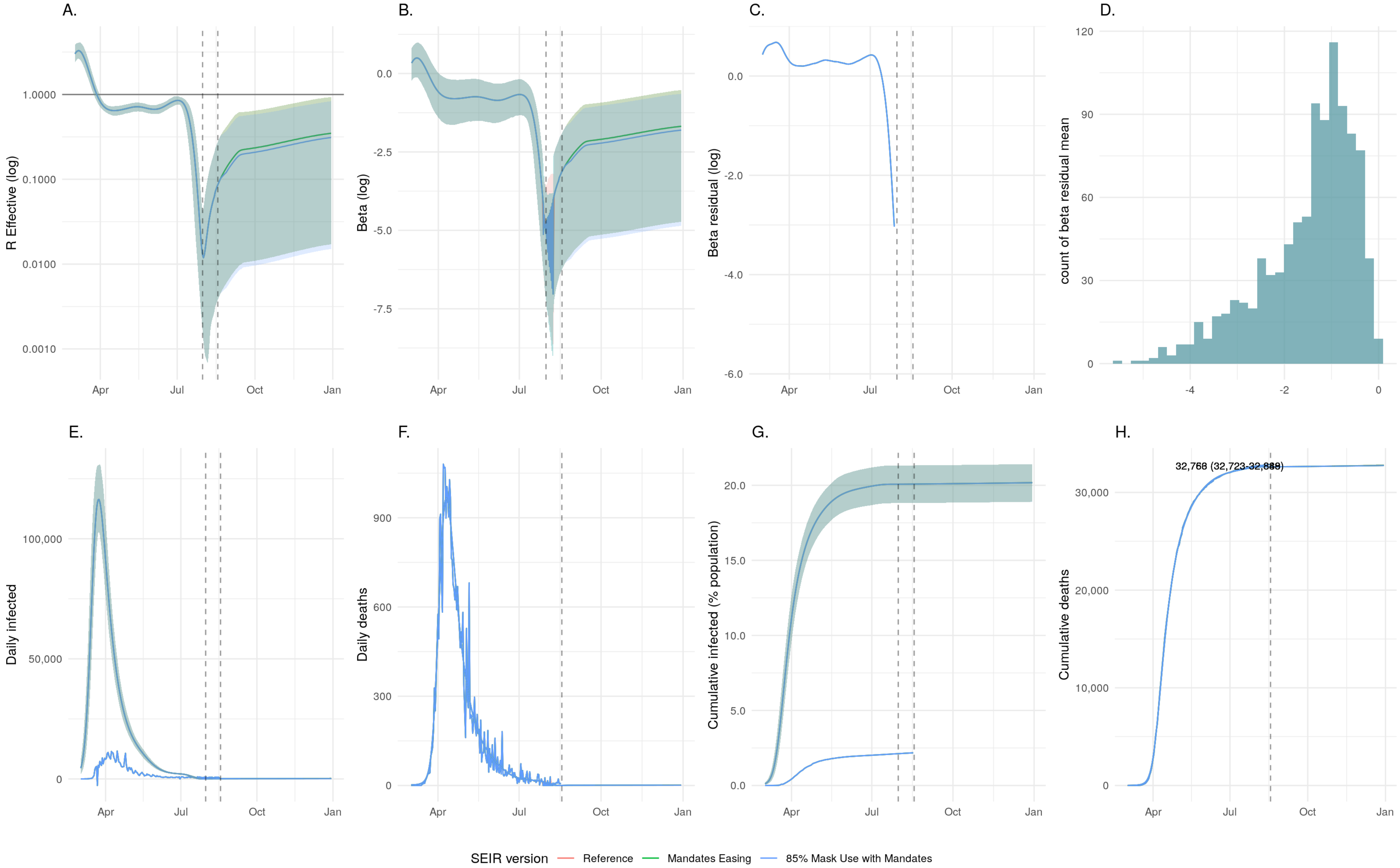

New York: SEIR fit comparison. A: predicted R effective for each model through December 31. **B**: predicted SEIR *beta* parameter. **C**: residual of predicted *beta* and the observed value calculated directly from infection data over time. **D**: histogram of residual values for *beta*. Panels A, B, C, and D are all displayed in log space, reflecting the space in which the SEIR model is fit. **E**: predicted daily infections from each model through December 31. **F**: predicted daily deaths from each model through December 31. **G**: predicted cumulative infections through December 31, as a proportion of the total population. **H**: predicted cumulative deaths through December 31. In panels E, F, G, and H, reported death and infections are plotted alongside model predictions in light blue.

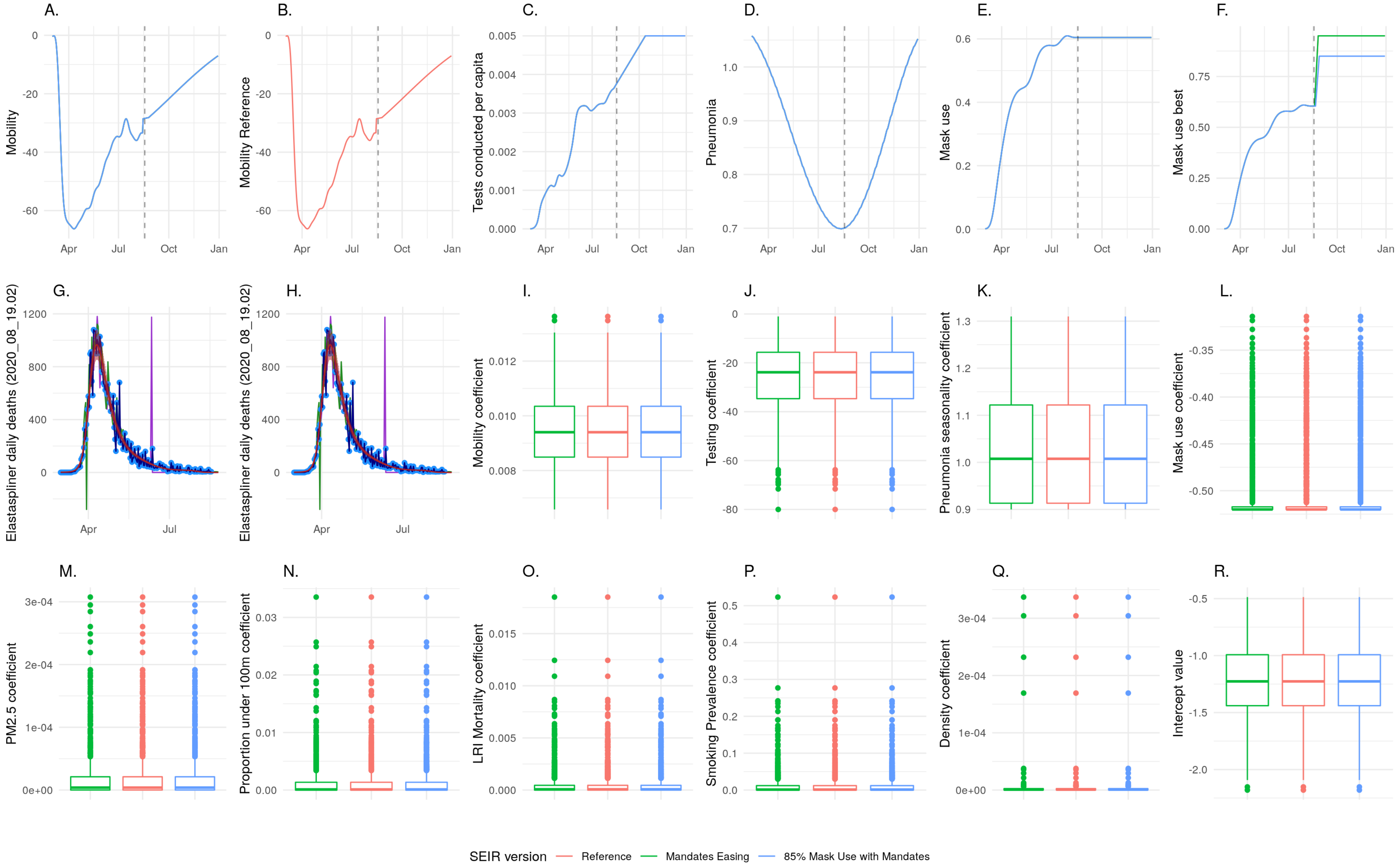

New York: Covariate fits and regression coefficients. **A-F**: Line plots showing predicted covariate time trends for **A)** mobility in the absence of additional mandates; **B)** mobility with additional mandates applied; **C)** diagnostic testing per capita; **D)** pneumonia seasonality; **E)** mask use per capita, and; **F)** mask use in a scenario where adherence increases to 85% of the population. **G-H**: COVID mortality data generated from reported daily deaths (blue); estimated based on reported hospitalizations (purple); estimated from reported cases (green); and via a spline fit through all available data types (red, 95% UI in pink). **I-R**: Box plots showing 1,000 draws of fixed effect coefficients in a multivariate regression fit to $log(beta)$.

69 North Carolina: SEIR fit comparison

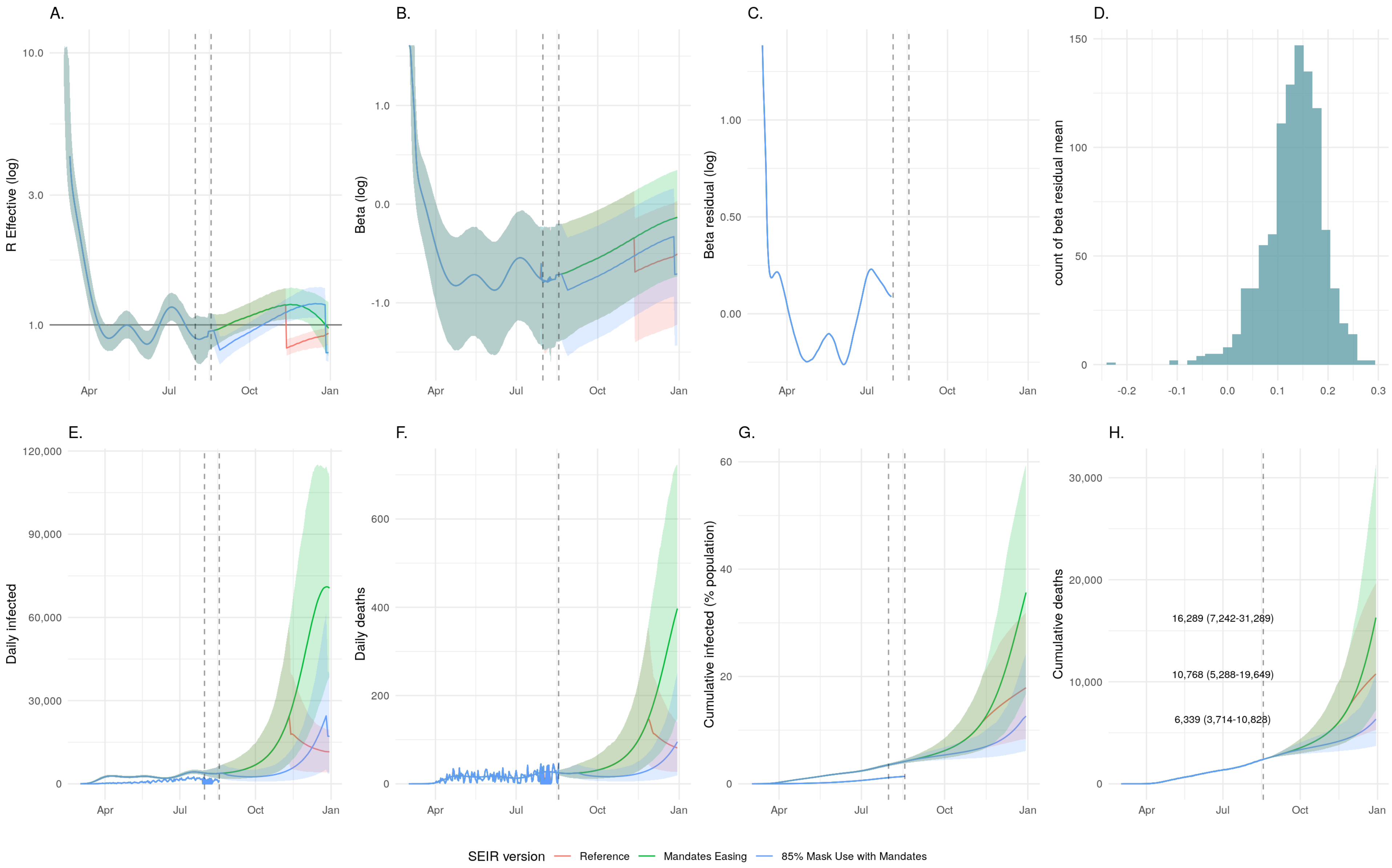

North Carolina: SEIR fit comparison. **A**: predicted R effective for each model through December 31. **B**: predicted SEIR *beta* parameter. **C**: residual of predicted *beta* and the observed value calculated directly from infection data over time. **D**: histogram of residual values for *beta*. Panels A, B, C, and D are all displayed in log space, reflecting the space in which the SEIR model is fit. **E**: predicted daily infections from each model through December 31. **F**: predicted daily deaths from each model through December 31. **G**: predicted cumulative infections through December 31, as a proportion of the total population. **H**: predicted cumulative deaths through December 31. In panels E, F, G, and H, reported death and infections are plotted alongside model predictions in light blue.

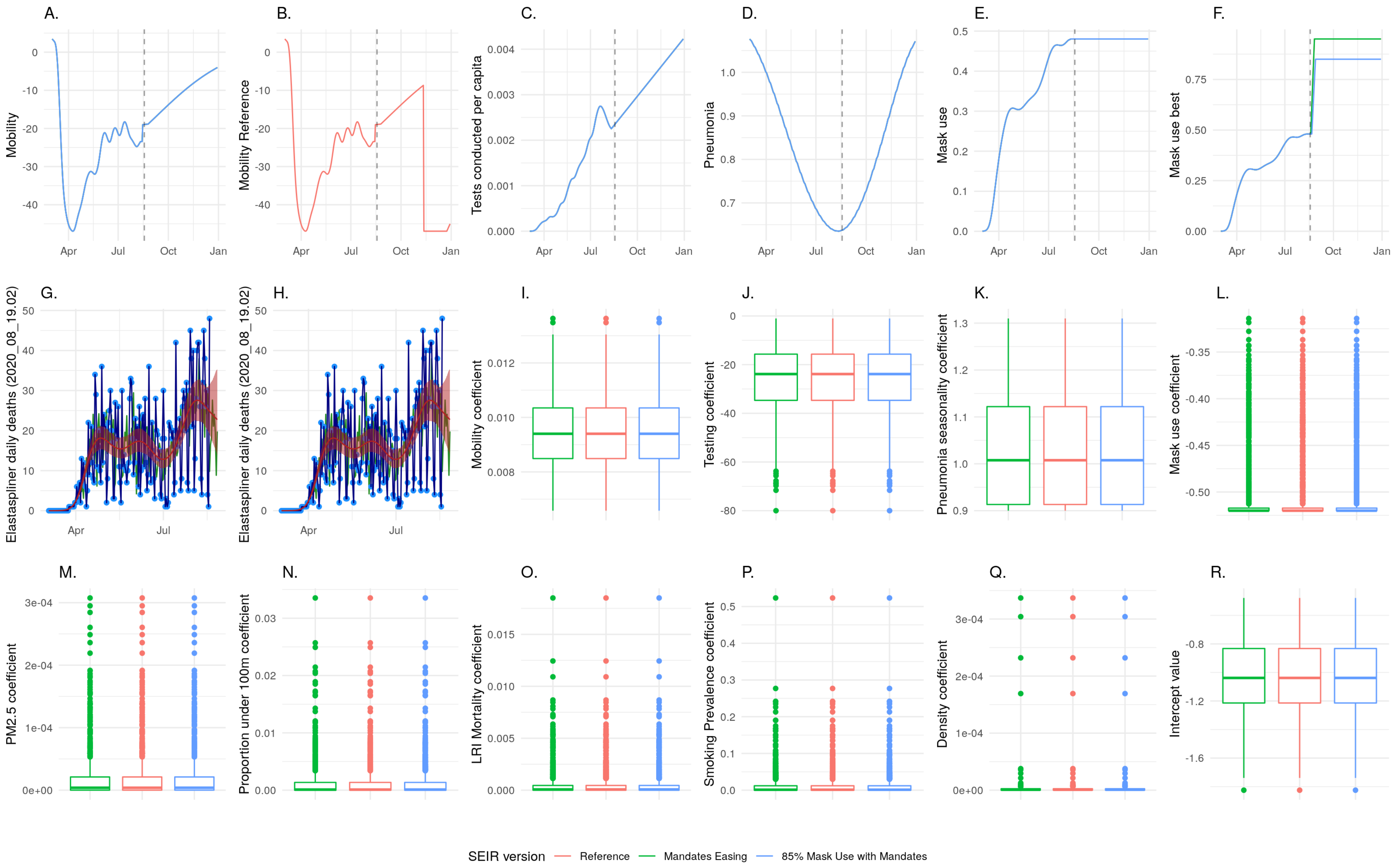

North Carolina: Covariate fits and regression coefficients. A-F: Line plots showing predicted covariate time trends for **A)** mobility in the absence of additional mandates; **B)** mobility with additional mandates applied; **C)** diagnostic testing per capita; **D)** pneumonia seasonality; **E)** mask use per capita, and; **F)** mask use in a scenario where adherence increases to 85% of the population. **G-H**: COVID mortality data generated from reported daily deaths (blue); estimated based on reported hospitalizations (purple); estimated from reported cases (green); and via a spline fit through all available data types (red, 95% UI in pink). **I-R**: Box plots showing 1,000 draws of fixed effect coefficients in a multivariate regression fit to $log(beta)$.

71 North Dakota: SEIR fit comparison

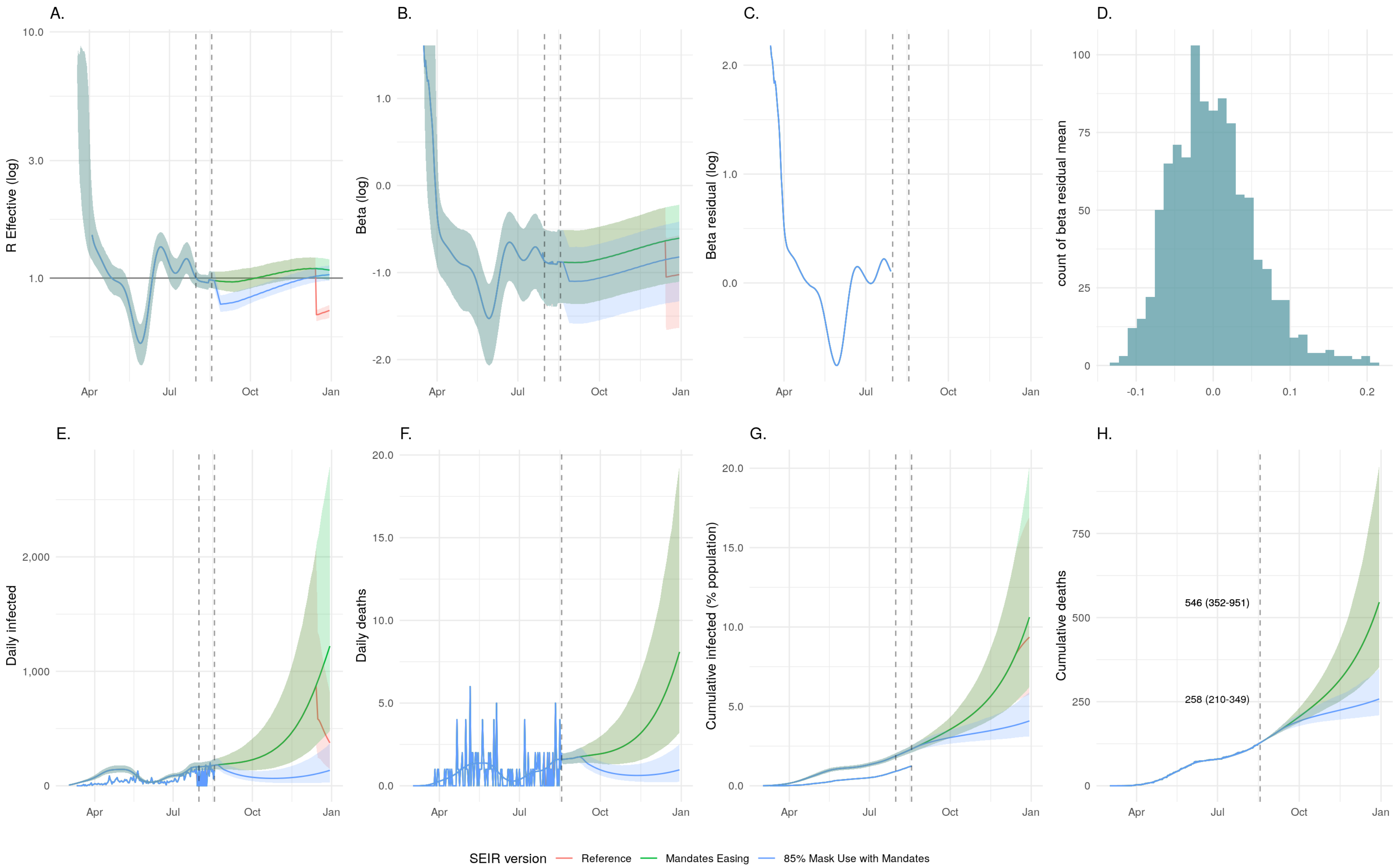

North Dakota: SEIR fit comparison. A: predicted R effective for each model through December 31. **B**: predicted SEIR *beta* parameter. **C**: residual of predicted *beta* and the observed value calculated directly from infection data over time. **D**: histogram of residual values for *beta*. Panels A, B, C, and D are all displayed in log space, reflecting the space in which the SEIR model is fit. **E**: predicted daily infections from each model through December 31. **F**: predicted daily deaths from each model through December 31. **G**: predicted cumulative infections through December 31, as a proportion of the total population. **H**: predicted cumulative deaths through December 31. In panels E, F, G, and H, reported death and infections are plotted alongside model predictions in light blue.

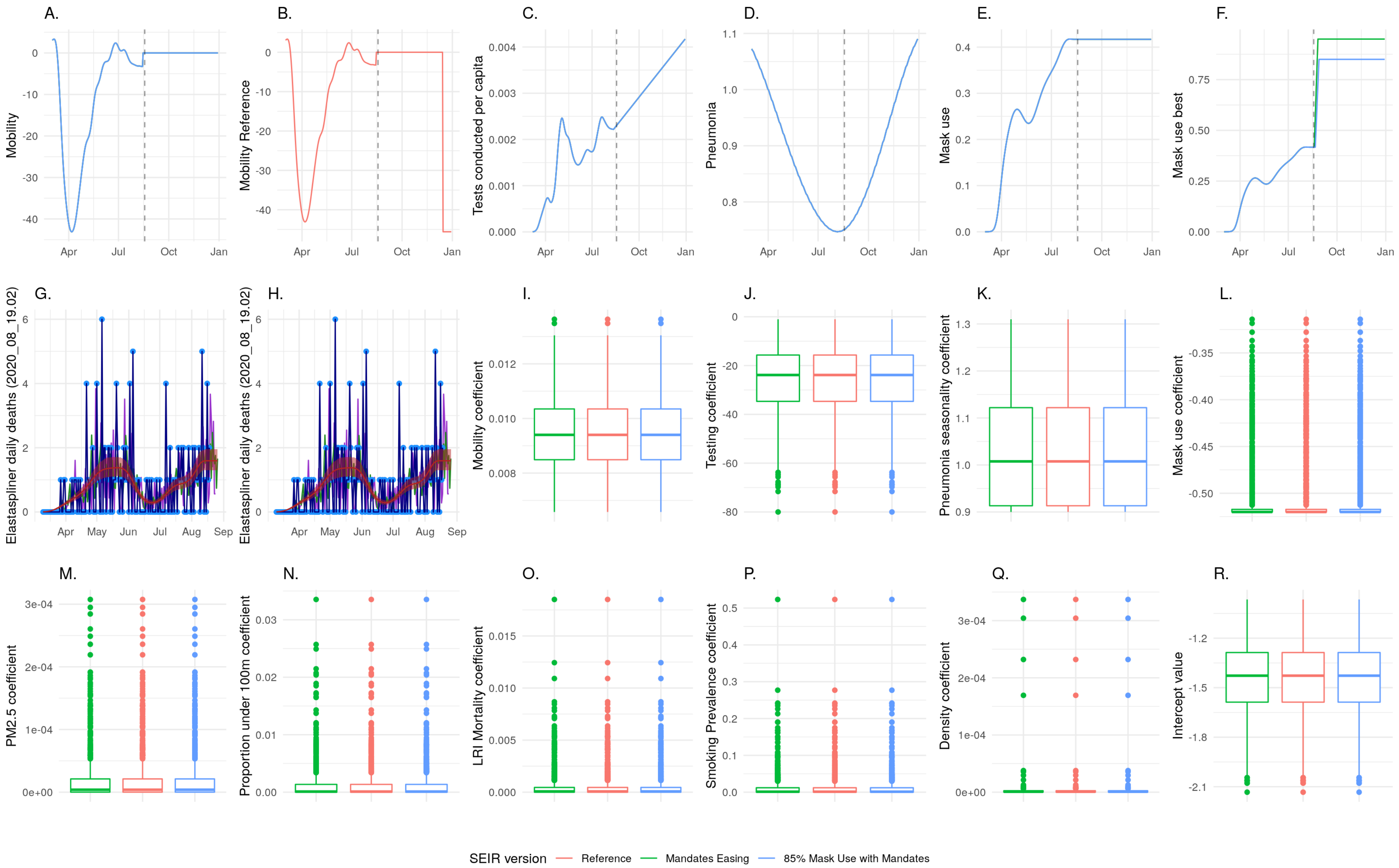

North Dakota: Covariate fits and regression coefficients. **A-F**: Line plots showing predicted covariate time trends for **A)** mobility in the absence of additional mandates; **B)** mobility with additional mandates applied; **C)** diagnostic testing per capita; **D)** pneumonia seasonality; **E)** mask use per capita, and; **F)** mask use in a scenario where adherence increases to 85% of the population. **G-H**: COVID mortality data generated from reported daily deaths (blue); estimated based on reported hospitalizations (purple); estimated from reported cases (green); and via a spline fit through all available data types (red, 95% UI in pink). **I-R**: Box plots showing 1,000 draws of fixed effect coefficients in a multivariate regression fit to $log(beta)$.

73 Ohio: SEIR fit comparison

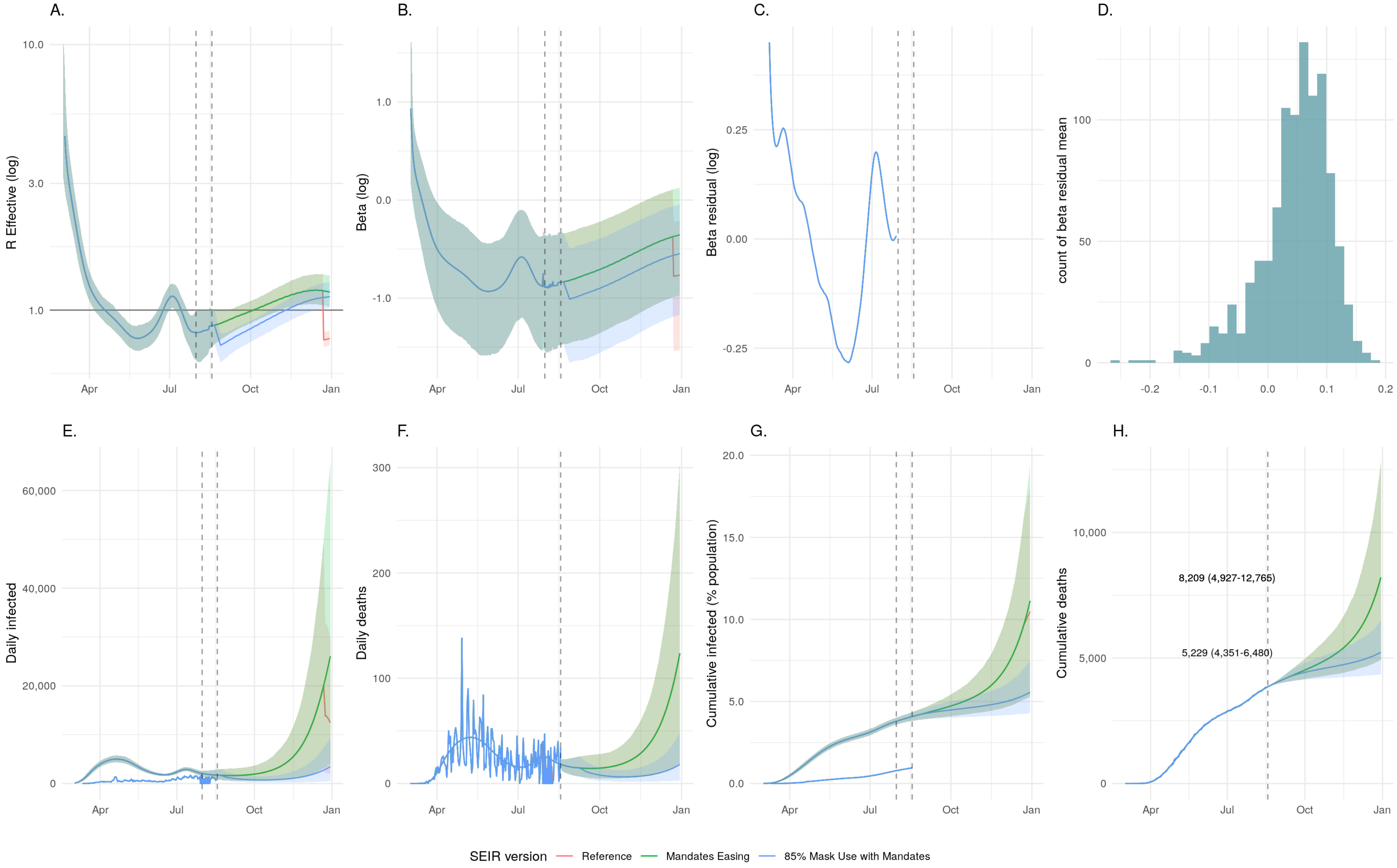

Ohio: SEIR fit comparison. A: predicted R effective for each model through December 31. **B**: predicted SEIR *beta* parameter. **C**: residual of predicted *beta* and the observed value calculated directly from infection data over time. **D**: histogram of residual values for *beta*. Panels A, B, C, and D are all displayed in log space, reflecting the space in which the SEIR model is fit. **E**: predicted daily infections from each model through December 31. **F**: predicted daily deaths from each model through December 31. **G**: predicted cumulative infections through December 31, as a proportion of the total population. **H**: predicted cumulative deaths through December 31. In panels E, F, G, and H, reported death and infections are plotted alongside model predictions in light blue.

74 Ohio: Covariate fits and regression coefficients

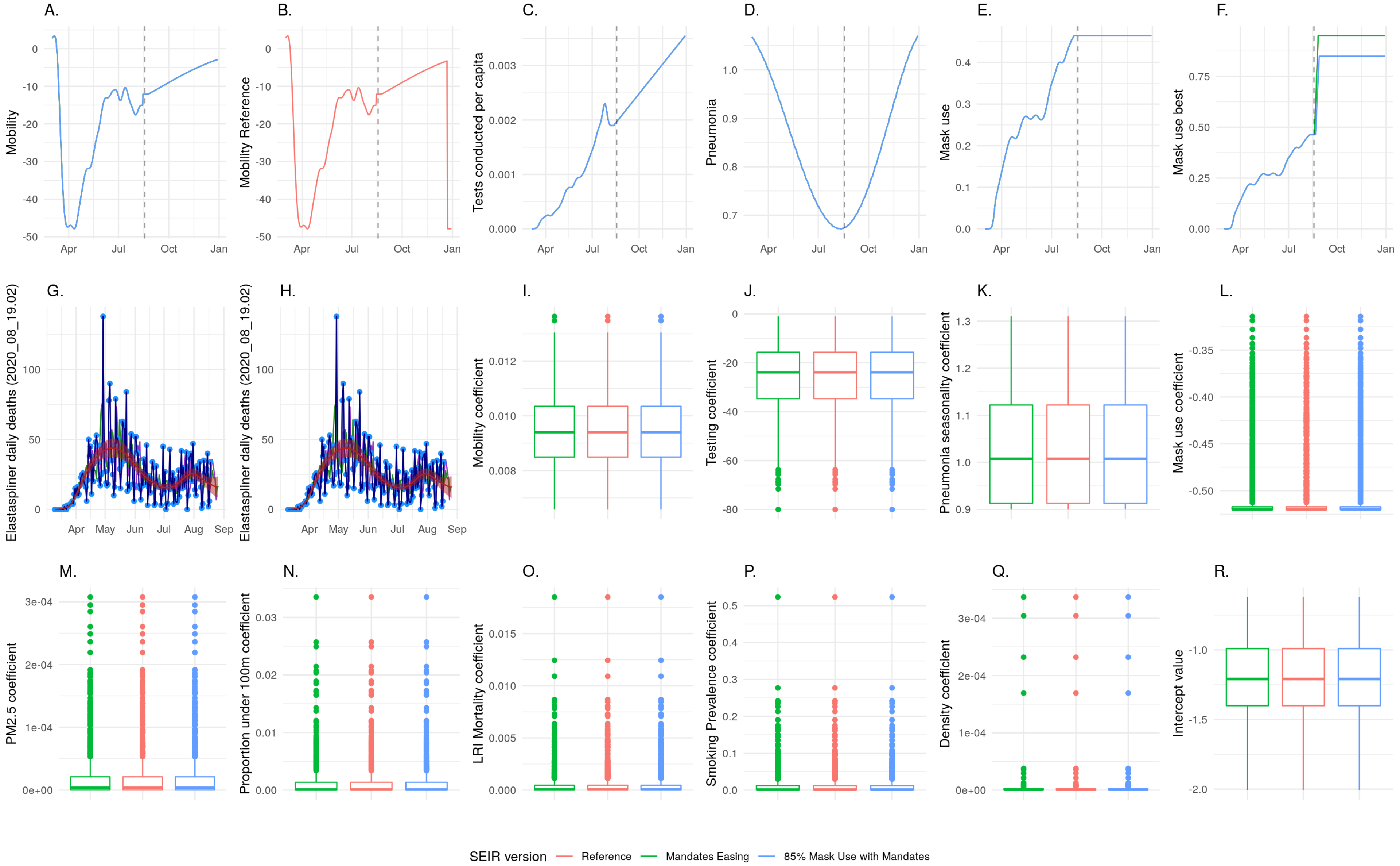

Ohio: Covariate fits and regression coefficients. A-F: Line plots showing predicted covariate time trends for **A)** mobility in the absence of additional mandates; **B)** mobility with additional mandates applied; **C)** diagnostic testing per capita; **D)** pneumonia seasonality; **E)** mask use per capita, and; **F)** mask use in a scenario where adherence increases to 85% of the population. **G-H**: COVID mortality data generated from reported daily deaths (blue); estimated based on reported hospitalizations (purple); estimated from reported cases (green); and via a spline fit through all available data types (red, 95% UI in pink). **I-R**: Box plots showing 1,000 draws of fixed effect coefficients in a multivariate regression fit to $log(beta)$.

75 Oklahoma: SEIR fit comparison

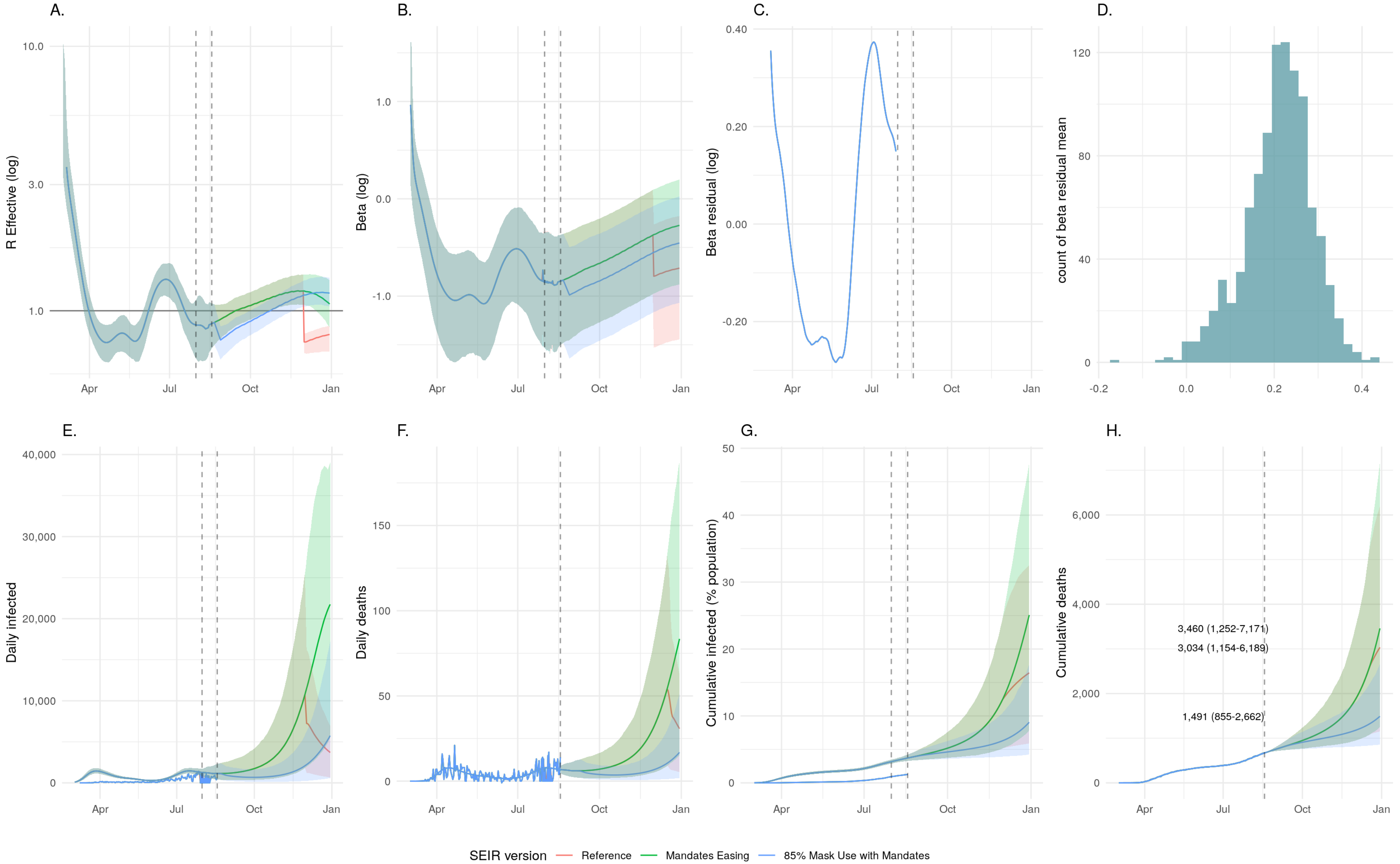

Oklahoma: SEIR fit comparison. A: predicted R effective for each model through December 31. **B**: predicted SEIR *beta* parameter. **C**: residual of predicted *beta* and the observed value calculated directly from infection data over time. **D**: histogram of residual values for *beta*. Panels A, B, C, and D are all displayed in log space, reflecting the space in which the SEIR model is fit. **E**: predicted daily infections from each model through December 31. **F**: predicted daily deaths from each model through December 31. **G**: predicted cumulative infections through December 31, as a proportion of the total population. **H**: predicted cumulative deaths through December 31. In panels E, F, G, and H, reported death and infections are plotted alongside model predictions in light blue.

76 Oklahoma: Covariate fits and regression coefficients

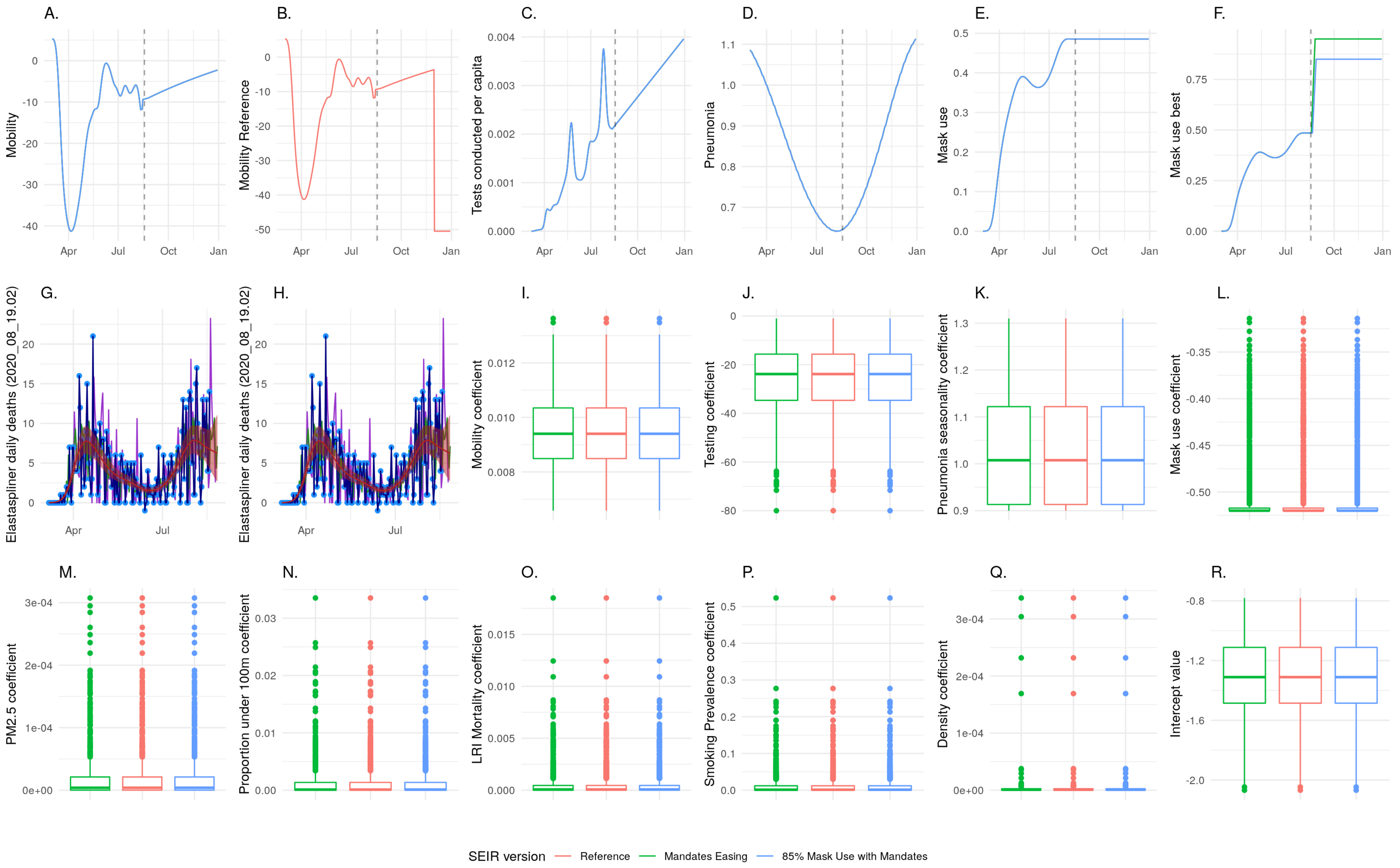

Oklahoma: Covariate fits and regression coefficients. **A-F**: Line plots showing predicted covariate time trends for **A)** mobility in the absence of additional mandates; **B)** mobility with additional mandates applied; **C)** diagnostic testing per capita; **D)** pneumonia seasonality; **E)** mask use per capita, and; **F)** mask use in a scenario where adherence increases to 85% of the population. **G-H**: COVID mortality data generated from reported daily deaths (blue); estimated based on reported hospitalizations (purple); estimated from reported cases (green); and via a spline fit through all available data types (red, 95% UI in pink). **I-R**: Box plots showing 1,000 draws of fixed effect coefficients in a multivariate regression fit to $log(beta)$.

77 Oregon: SEIR fit comparison

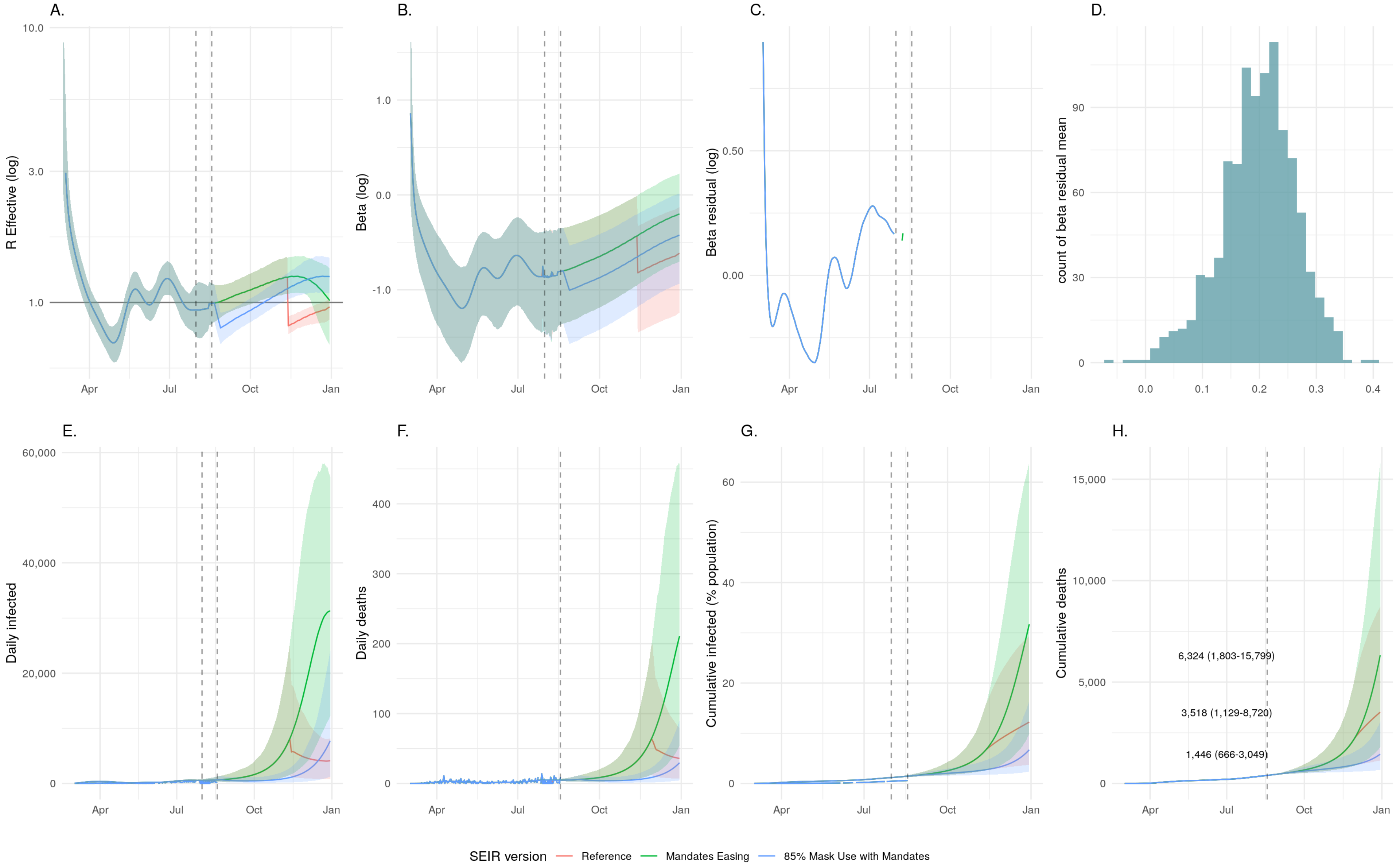

Oregon: SEIR fit comparison. **A**: predicted R effective for each model through December 31. **B**: predicted SEIR *beta* parameter. **C**: residual of predicted *beta* and the observed value calculated directly from infection data over time. **D**: histogram of residual values for *beta*. Panels A, B, C, and D are all displayed in log space, reflecting the space in which the SEIR model is fit. **E**: predicted daily infections from each model through December 31. **F**: predicted daily deaths from each model through December 31. **G**: predicted cumulative infections through December 31, as a proportion of the total population. **H**: predicted cumulative deaths through December 31. In panels E, F, G, and H, reported death and infections are plotted alongside model predictions in light blue.

78 Oregon: Covariate fits and regression coefficients

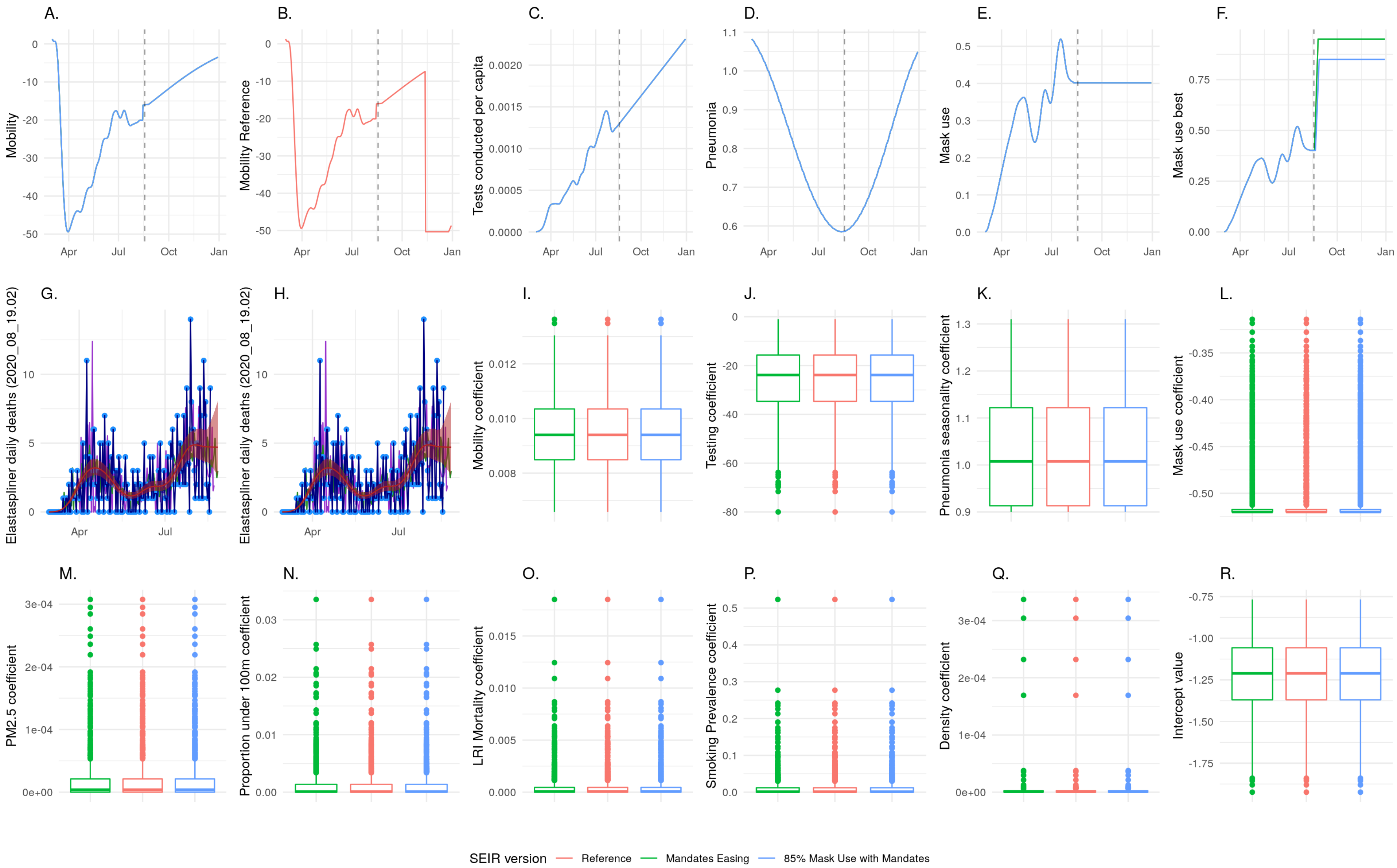

Oregon: Covariate fits and regression coefficients. **A-F**: Line plots showing predicted covariate time trends for **A)** mobility in the absence of additional mandates; **B)** mobility with additional mandates applied; **C)** diagnostic testing per capita; **D)** pneumonia seasonality; **E)** mask use per capita, and; **F)** mask use in a scenario where adherence increases to 85% of the population. **G-H**: COVID mortality data generated from reported daily deaths (blue); estimated based on reported hospitalizations (purple); estimated from reported cases (green); and via a spline fit through all available data types (red, 95% UI in pink). **I-R**: Box plots showing 1,000 draws of fixed effect coefficients in a multivariate regression fit to $log(beta)$.

79 Pennsylvania: SEIR fit comparison

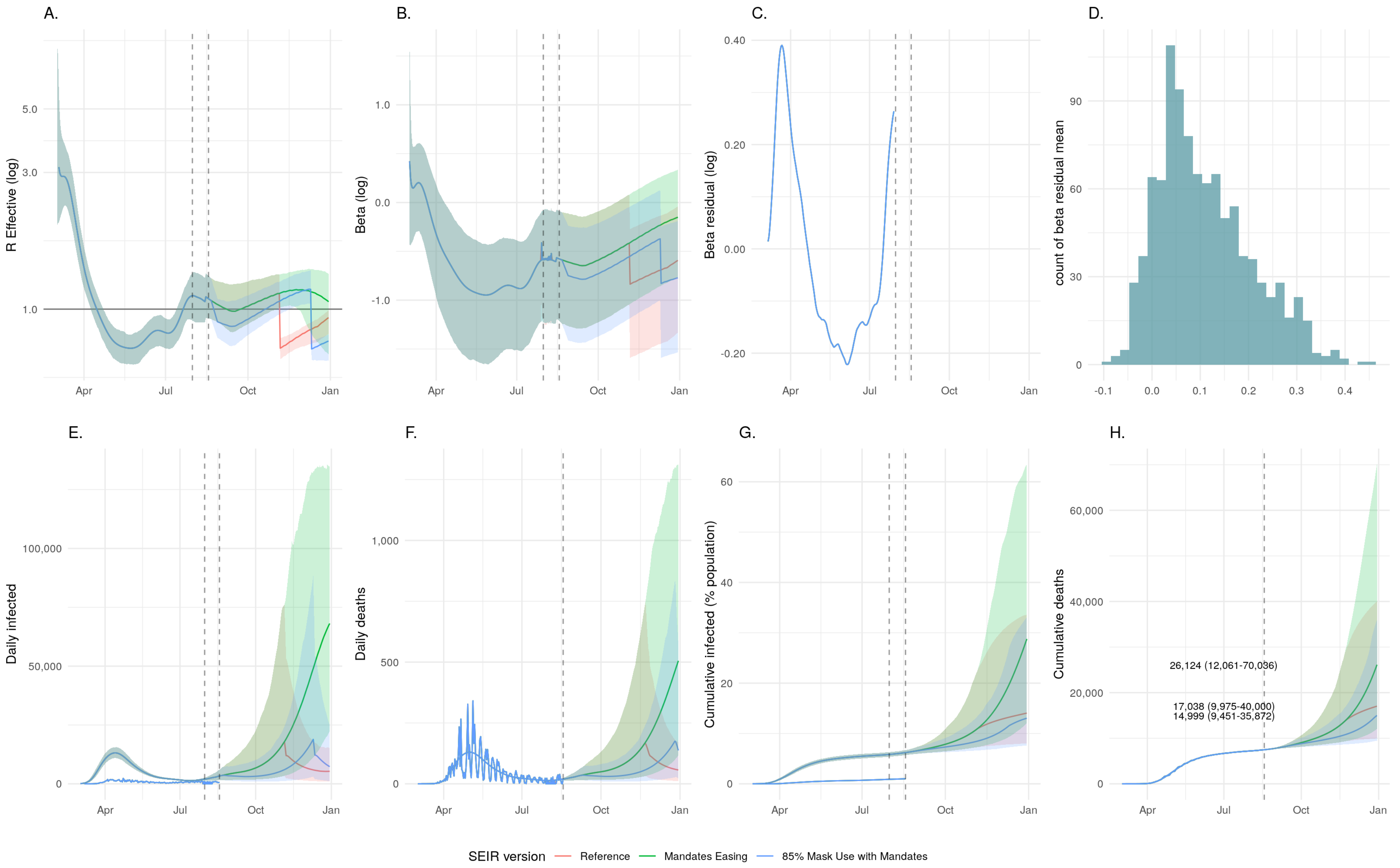

Pennsylvania: SEIR fit comparison. A: predicted R effective for each model through December 31. **B**: predicted SEIR *beta* parameter. **C**: residual of predicted *beta* and the observed value calculated directly from infection data over time. **D**: histogram of residual values for *beta*. Panels A, B, C, and D are all displayed in log space, reflecting the space in which the SEIR model is fit. **E**: predicted daily infections from each model through December 31. **F**: predicted daily deaths from each model through December 31. **G**: predicted cumulative infections through December 31, as a proportion of the total population. **H**: predicted cumulative deaths through December 31. In panels E, F, G, and H, reported death and infections are plotted alongside model predictions in light blue.

80 Pennsylvania: Covariate fits and regression coefficients

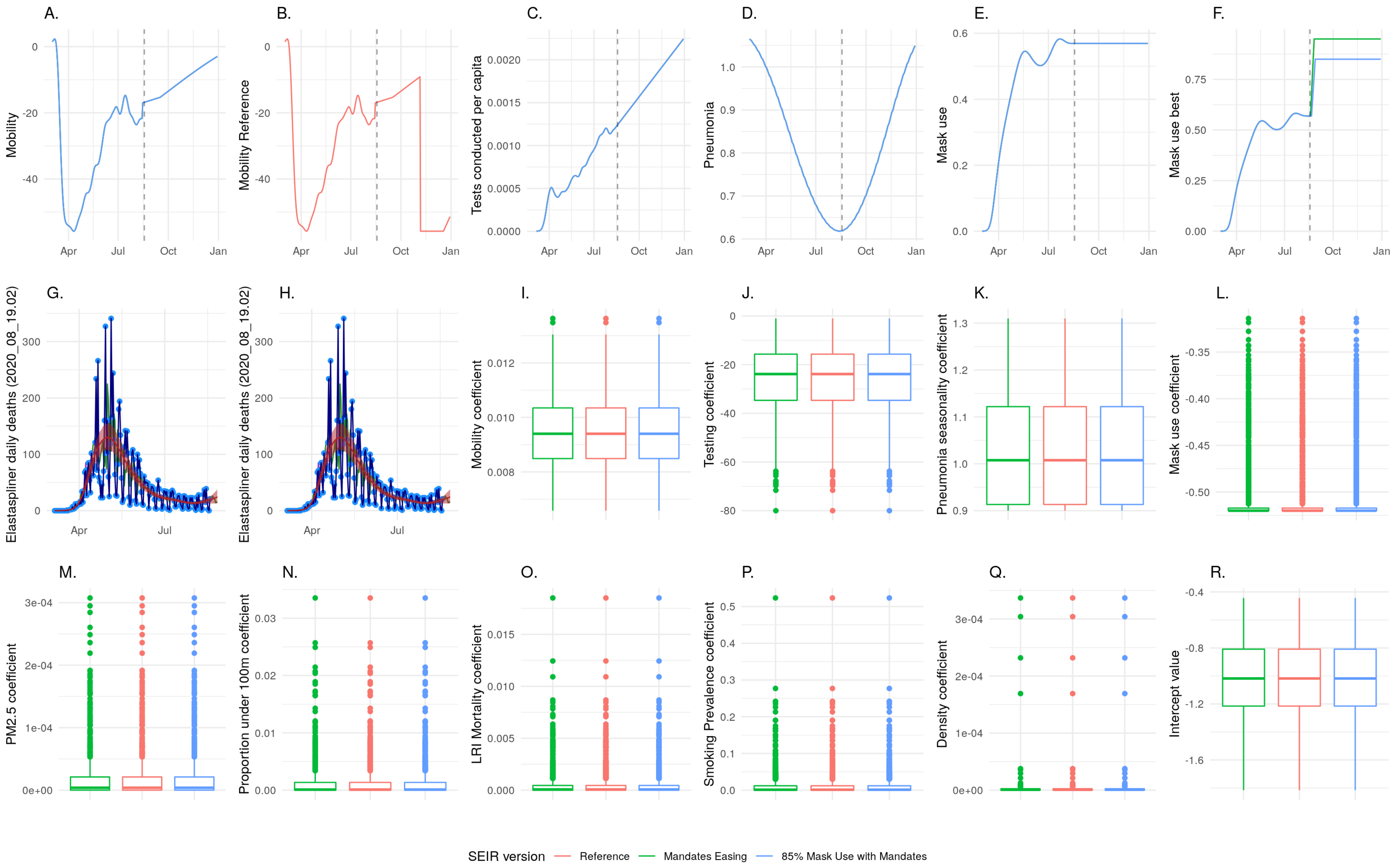

Pennsylvania: Covariate fits and regression coefficients. A-F: Line plots showing predicted covariate time trends for **A)** mobility in the absence of additional mandates; **B)** mobility with additional mandates applied; **C)** diagnostic testing per capita; **D)** pneumonia seasonality; **E)** mask use per capita, and; **F)** mask use in a scenario where adherence increases to 85% of the population. **G-H**: COVID mortality data generated from reported daily deaths (blue); estimated based on reported hospitalizations (purple); estimated from reported cases (green); and via a spline fit through all available data types (red, 95% UI in pink). **I-R**: Box plots showing 1,000 draws of fixed effect coefficients in a multivariate regression fit to $log(beta)$.

81 Rhode Island: SEIR fit comparison

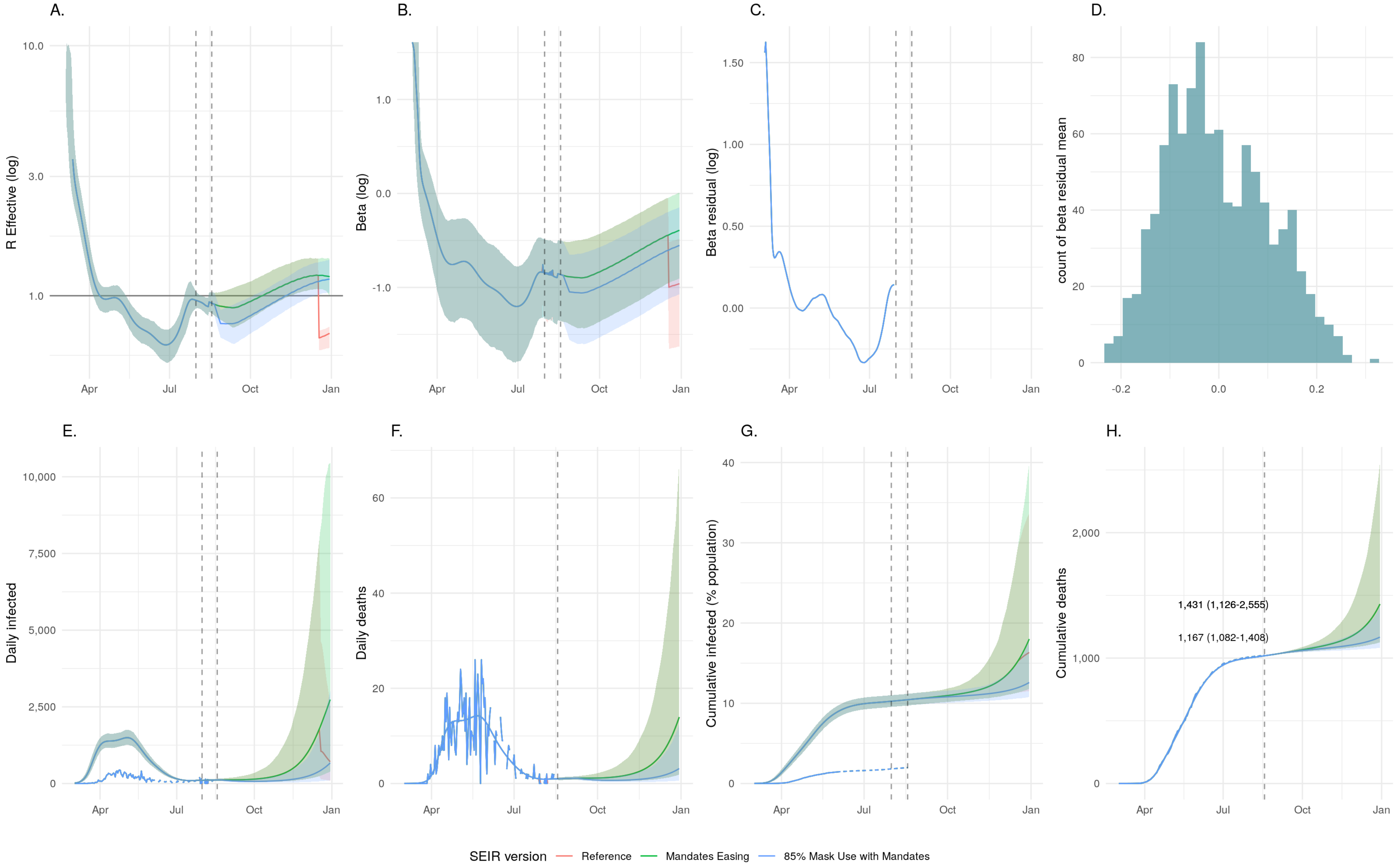

Rhode Island: SEIR fit comparison. **A**: predicted R effective for each model through December 31. **B**: predicted SEIR *beta* parameter. **C**: residual of predicted *beta* and the observed value calculated directly from infection data over time. **D**: histogram of residual values for *beta*. Panels A, B, C, and D are all displayed in log space, reflecting the space in which the SEIR model is fit. **E**: predicted daily infections from each model through December 31. **F**: predicted daily deaths from each model through December 31. **G**: predicted cumulative infections through December 31, as a proportion of the total population. **H**: predicted cumulative deaths through December 31. In panels E, F, G, and H, reported death and infections are plotted alongside model predictions in light blue.

82 Rhode Island: Covariate fits and regression coefficients

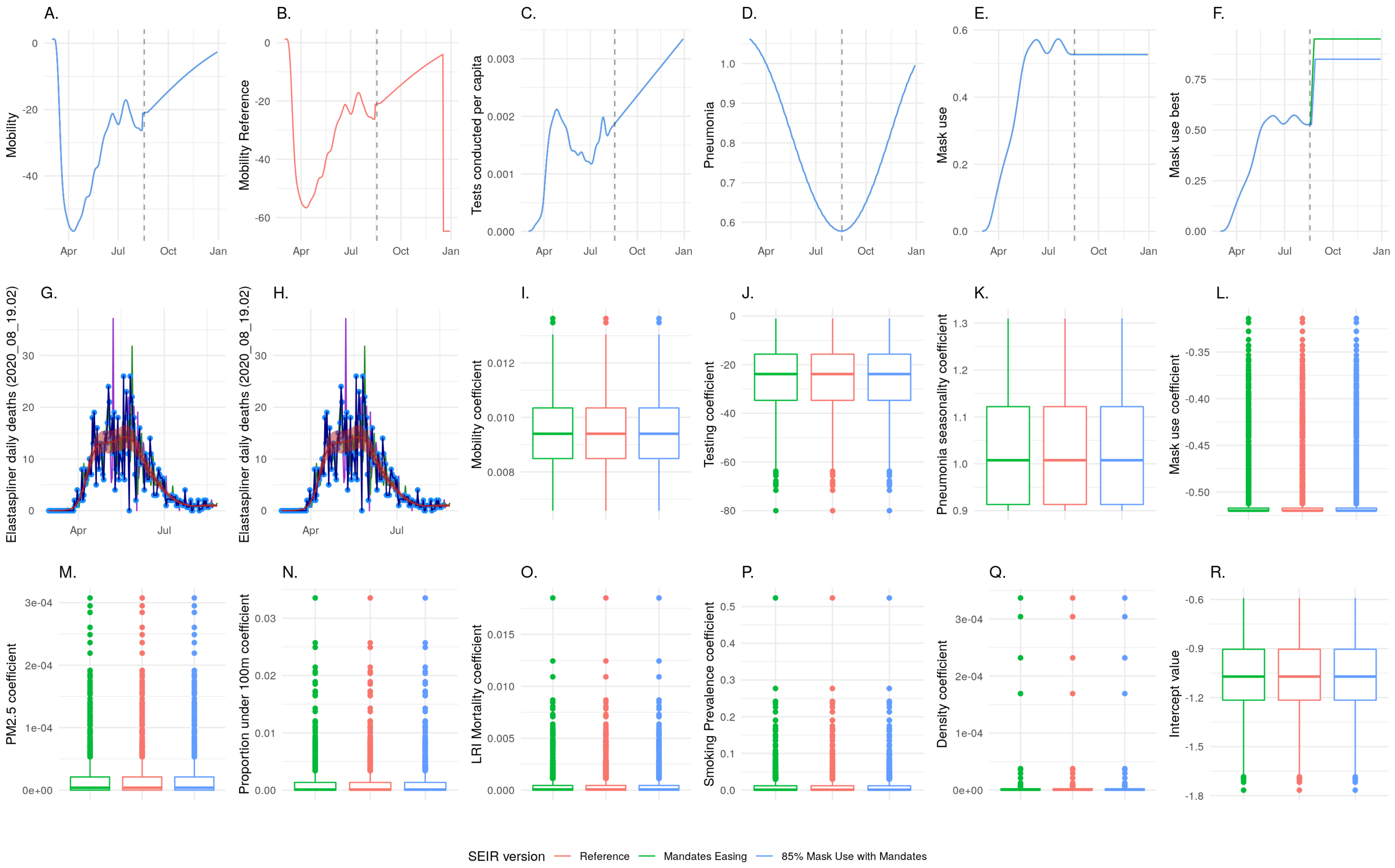

Rhode Island: Covariate fits and regression coefficients. A-F: Line plots showing predicted covariate time trends for **A)** mobility in the absence of additional mandates; **B)** mobility with additional mandates applied; **C)** diagnostic testing per capita; **D)** pneumonia seasonality; **E)** mask use per capita, and; **F)** mask use in a scenario where adherence increases to 85% of the population. **G-H**: COVID mortality data generated from reported daily deaths (blue); estimated based on reported hospitalizations (purple); estimated from reported cases (green); and via a spline fit through all available data types (red, 95% UI in pink). **I-R**: Box plots showing 1,000 draws of fixed effect coefficients in a multivariate regression fit to $log(beta)$.

83 South Carolina: SEIR fit comparison

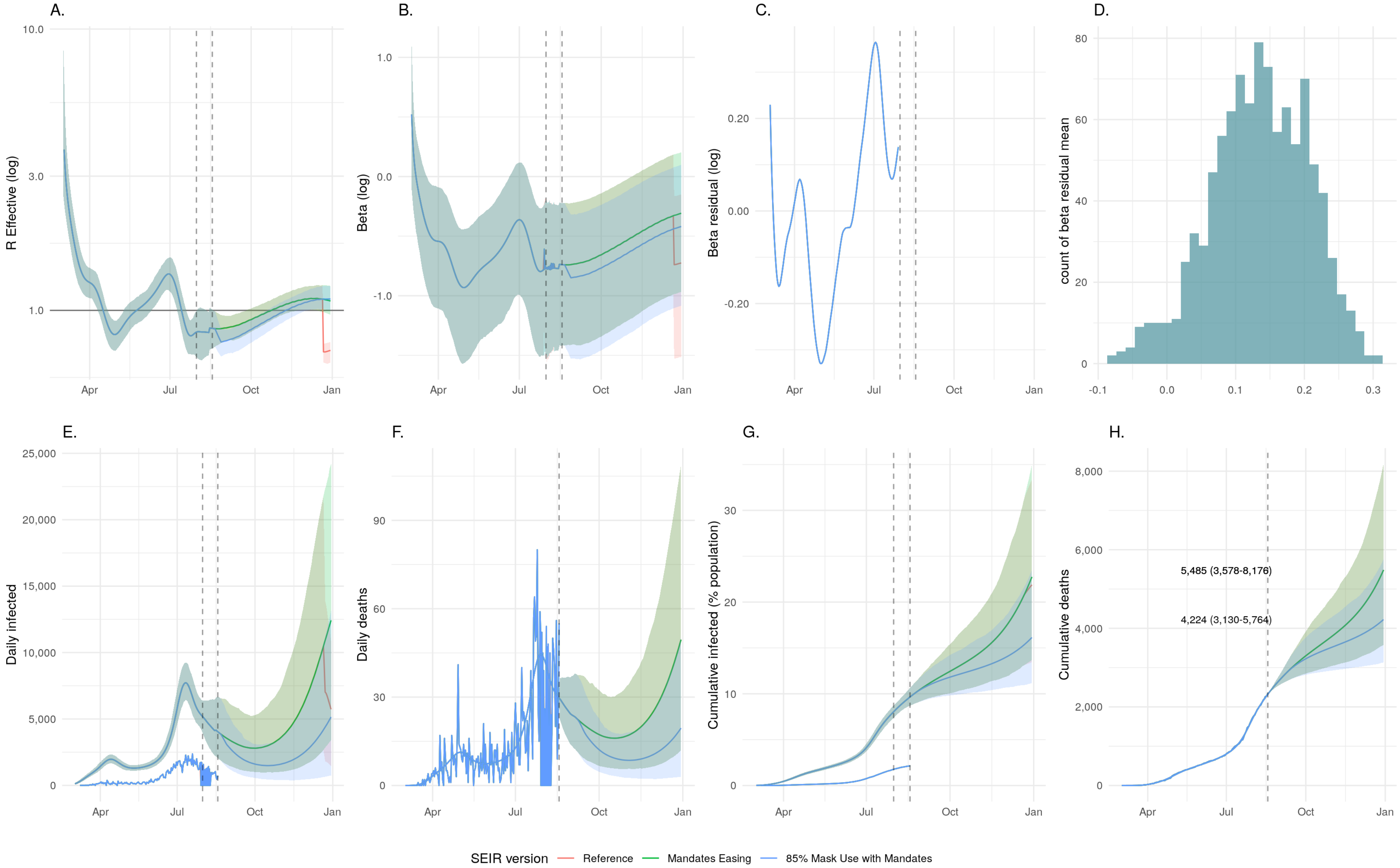

South Carolina: SEIR fit comparison. **A**: predicted R effective for each model through December 31. **B**: predicted SEIR *beta* parameter. **C**: residual of predicted *beta* and the observed value calculated directly from infection data over time. **D**: histogram of residual values for *beta*. Panels A, B, C, and D are all displayed in log space, reflecting the space in which the SEIR model is fit. **E**: predicted daily infections from each model through December 31. **F**: predicted daily deaths from each model through December 31. **G**: predicted cumulative infections through December 31, as a proportion of the total population. **H**: predicted cumulative deaths through December 31. In panels E, F, G, and H, reported death and infections are plotted alongside model predictions in light blue.

84 South Carolina: Covariate fits and regression coefficients

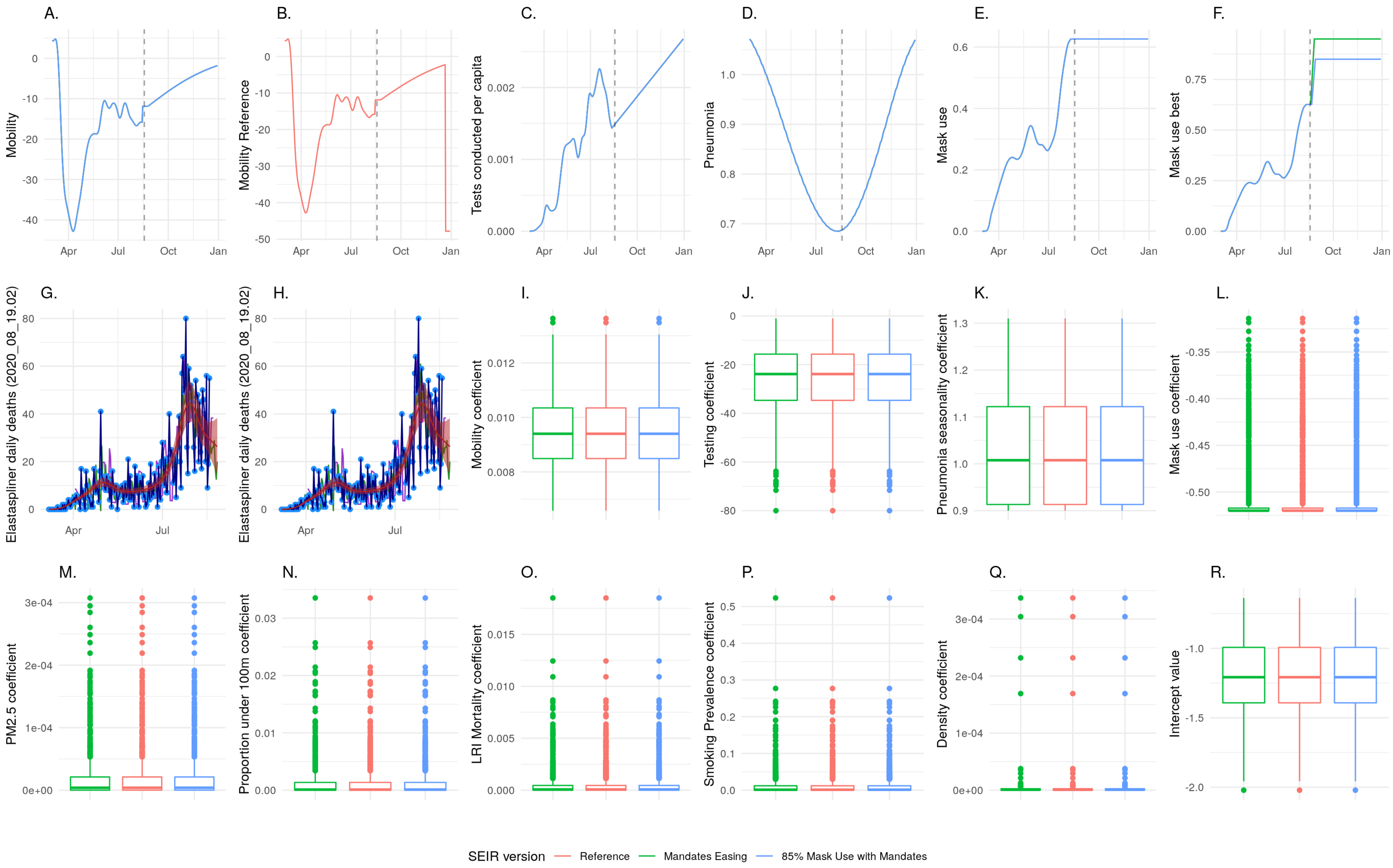

South Carolina: Covariate fits and regression coefficients. A-F: Line plots showing predicted covariate time trends for **A)** mobility in the absence of additional mandates; **B)** mobility with additional mandates applied; **C)** diagnostic testing per capita; **D)** pneumonia seasonality; **E)** mask use per capita, and; **F)** mask use in a scenario where adherence increases to 85% of the population. **G-H**: COVID mortality data generated from reported daily deaths (blue); estimated based on reported hospitalizations (purple); estimated from reported cases (green); and via a spline fit through all available data types (red, 95% UI in pink). **I-R**: Box plots showing 1,000 draws of fixed effect coefficients in a multivariate regression fit to $log(beta)$.

85 South Dakota: SEIR fit comparison

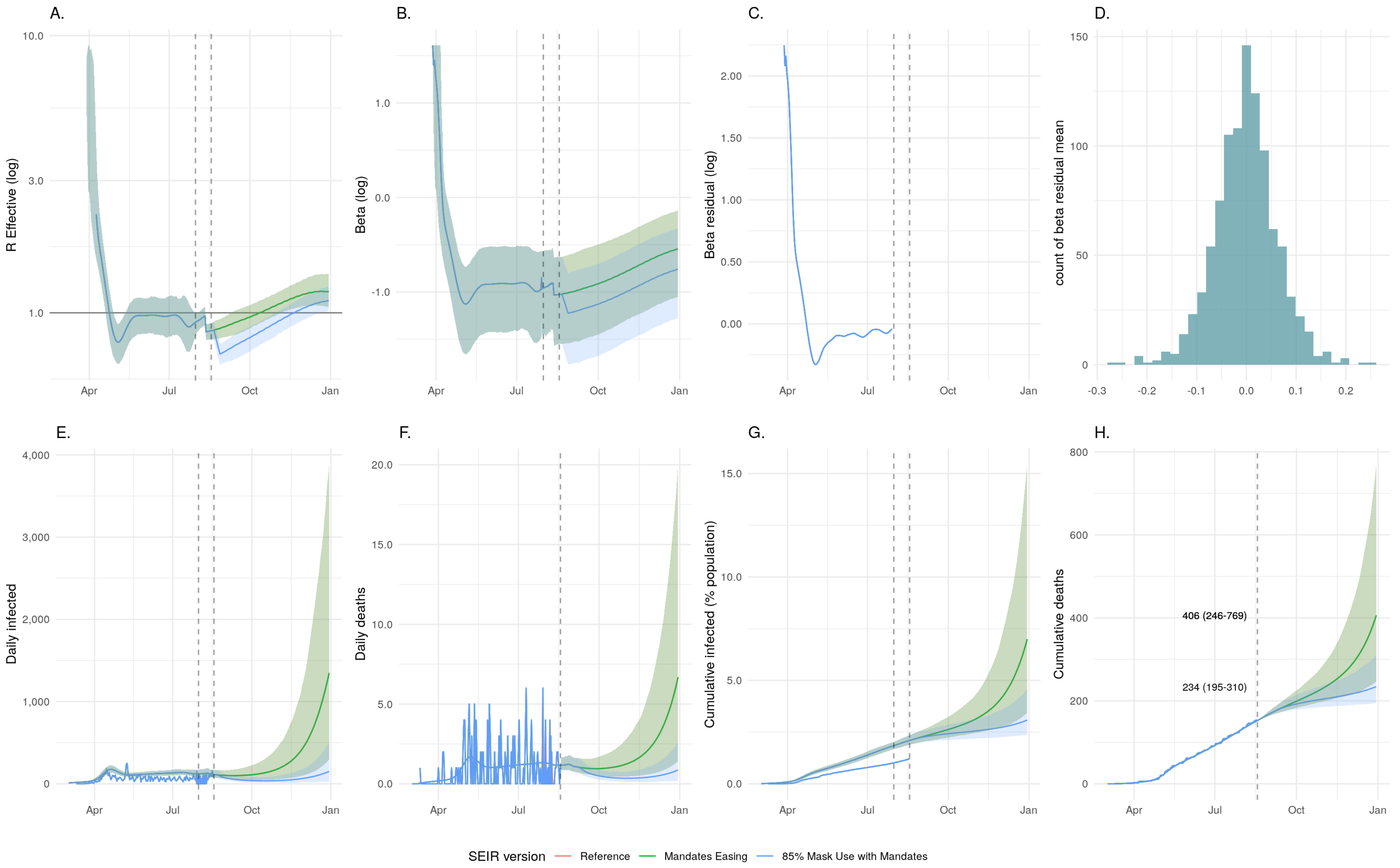

South Dakota: SEIR fit comparison. A: predicted R effective for each model through December 31. **B**: predicted SEIR *beta* parameter. **C**: residual of predicted *beta* and the observed value calculated directly from infection data over time. **D**: histogram of residual values for *beta*. Panels A, B, C, and D are all displayed in log space, reflecting the space in which the SEIR model is fit. **E**: predicted daily infections from each model through December 31. **F**: predicted daily deaths from each model through December 31. **G**: predicted cumulative infections through December 31, as a proportion of the total population. **H**: predicted cumulative deaths through December 31. In panels E, F, G, and H, reported death and infections are plotted alongside model predictions in light blue.

86 South Dakota: Covariate fits and regression coefficients

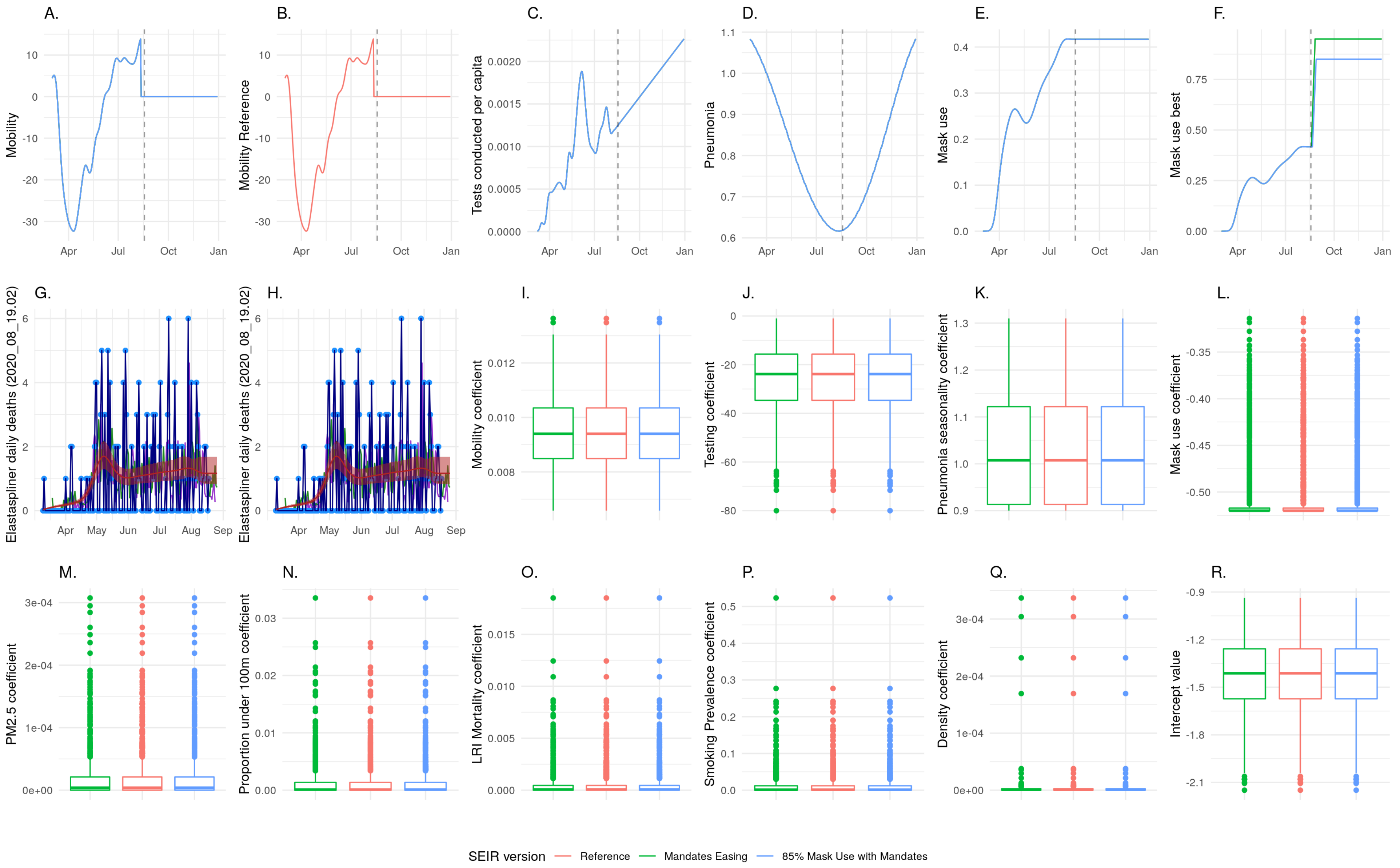

South Dakota: Covariate fits and regression coefficients. A-F: Line plots showing predicted covariate time trends for **A)** mobility in the absence of additional mandates; **B)** mobility with additional mandates applied; **C)** diagnostic testing per capita; **D)** pneumonia seasonality; **E)** mask use per capita, and; **F)** mask use in a scenario where adherence increases to 85% of the population. **G-H**: COVID mortality data generated from reported daily deaths (blue); estimated based on reported hospitalizations (purple); estimated from reported cases (green); and via a spline fit through all available data types (red, 95% UI in pink). **I-R**: Box plots showing 1,000 draws of fixed effect coefficients in a multivariate regression fit to $log(beta)$.

87 Tennessee: SEIR fit comparison

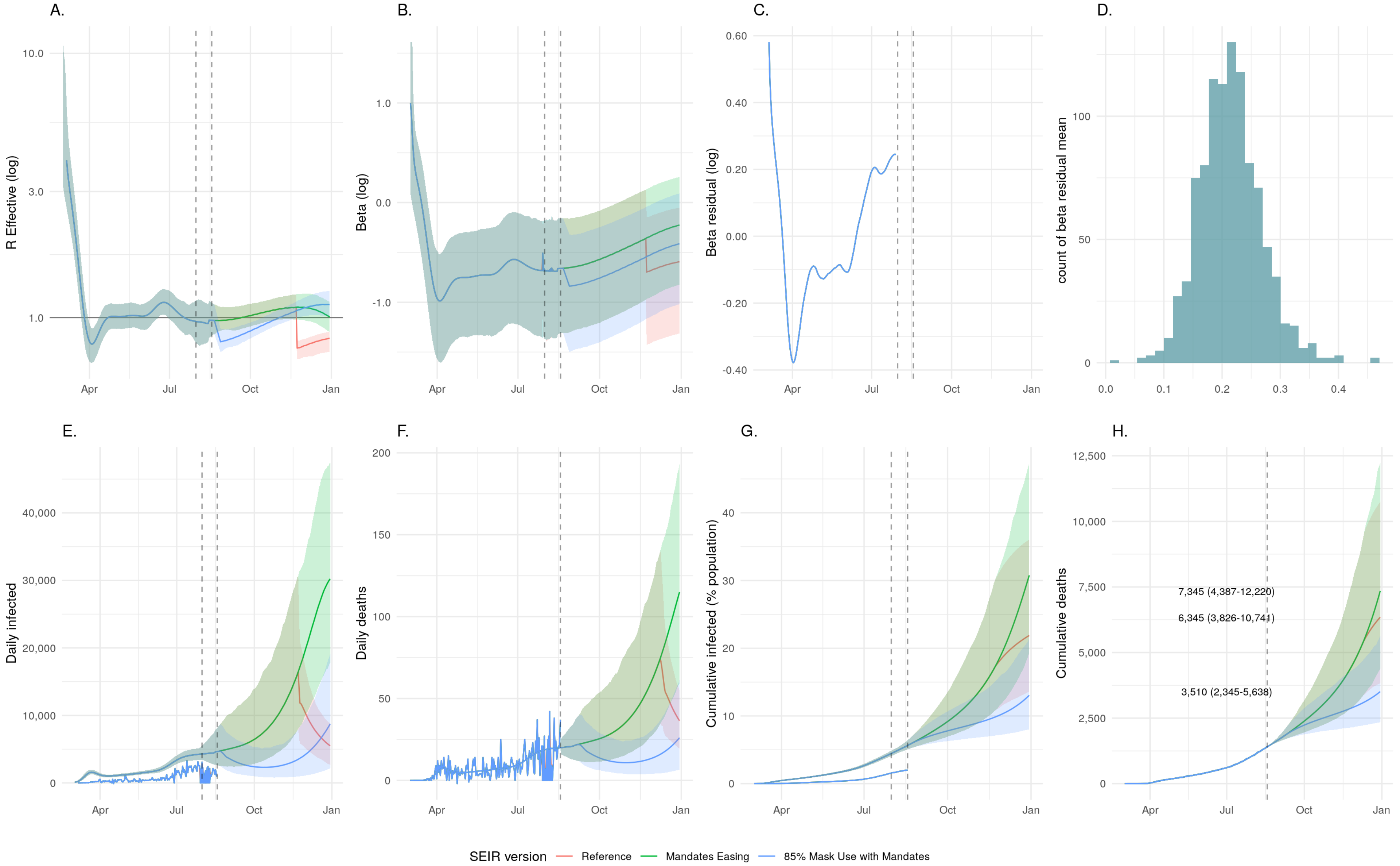

Tennessee: SEIR fit comparison. A: predicted R effective for each model through December 31. **B**: predicted SEIR *beta* parameter. **C**: residual of predicted *beta* and the observed value calculated directly from infection data over time. **D**: histogram of residual values for *beta*. Panels A, B, C, and D are all displayed in log space, reflecting the space in which the SEIR model is fit. **E**: predicted daily infections from each model through December 31. **F**: predicted daily deaths from each model through December 31. **G**: predicted cumulative infections through December 31, as a proportion of the total population. **H**: predicted cumulative deaths through December 31. In panels E, F, G, and H, reported death and infections are plotted alongside model predictions in light blue.

88 Tennessee: Covariate fits and regression coefficients

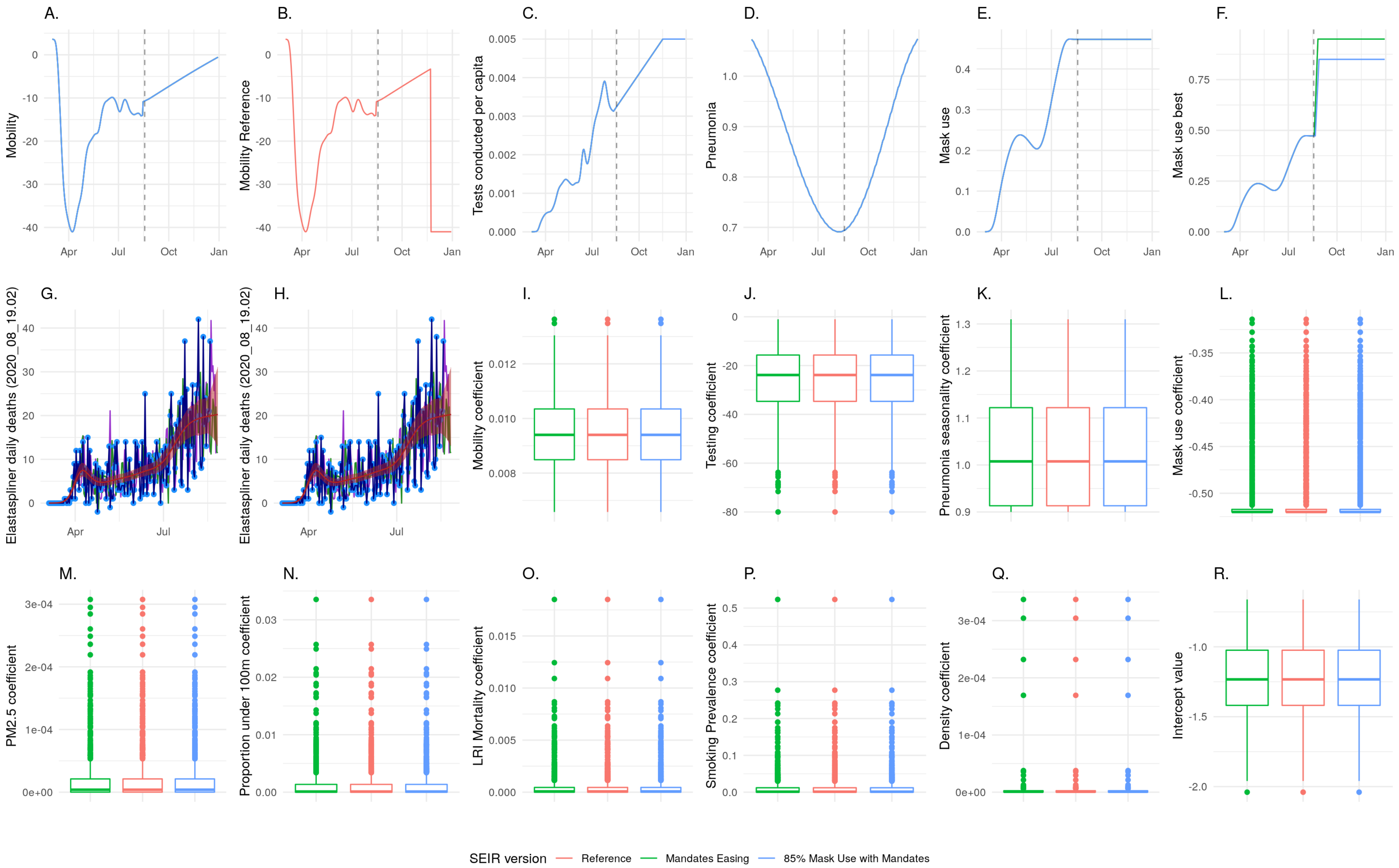

Tennessee: Covariate fits and regression coefficients. **A-F**: Line plots showing predicted covariate time trends for **A)** mobility in the absence of additional mandates; **B)** mobility with additional mandates applied; **C)** diagnostic testing per capita; **D)** pneumonia seasonality; **E)** mask use per capita, and; **F)** mask use in a scenario where adherence increases to 85% of the population. **G-H**: COVID mortality data generated from reported daily deaths (blue); estimated based on reported hospitalizations (purple); estimated from reported cases (green); and via a spline fit through all available data types (red, 95% UI in pink). **I-R**: Box plots showing 1,000 draws of fixed effect coefficients in a multivariate regression fit to $log(beta)$.

89 Texas: SEIR fit comparison

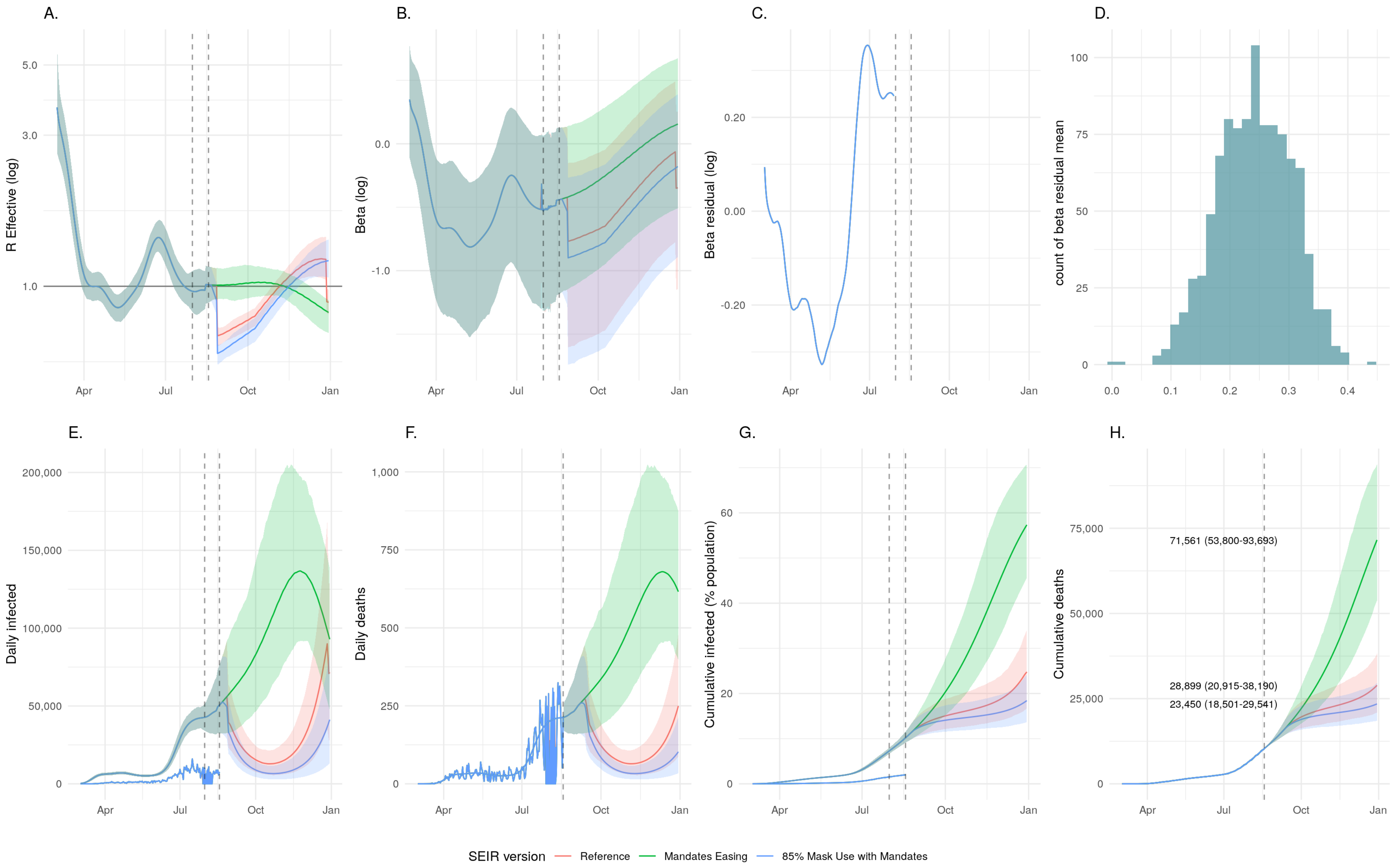

Texas: SEIR fit comparison. **A**: predicted R effective for each model through December 31. **B**: predicted SEIR *beta* parameter. **C**: residual of predicted *beta* and the observed value calculated directly from infection data over time. **D**: histogram of residual values for *beta*. Panels A, B, C, and D are all displayed in log space, reflecting the space in which the SEIR model is fit. **E**: predicted daily infections from each model through December 31. **F**: predicted daily deaths from each model through December 31. **G**: predicted cumulative infections through December 31, as a proportion of the total population. **H**: predicted cumulative deaths through December 31. In panels E, F, G, and H, reported death and infections are plotted alongside model predictions in light blue.

90 Texas: Covariate fits and regression coefficients

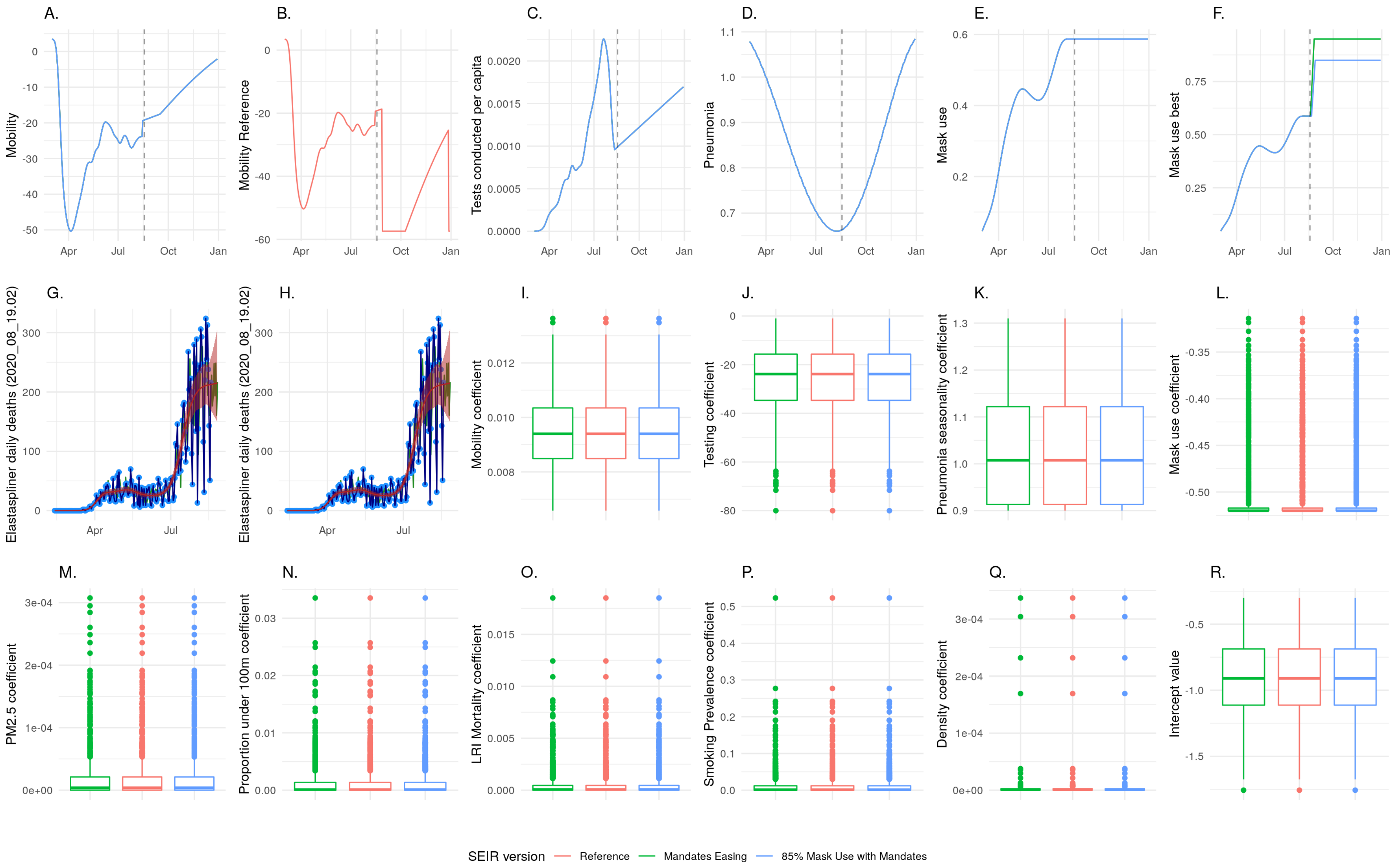

Texas: Covariate fits and regression coefficients. A-F: Line plots showing predicted covariate time trends for **A)** mobility in the absence of additional mandates; **B)** mobility with additional mandates applied; **C)** diagnostic testing per capita; **D)** pneumonia seasonality; **E)** mask use per capita, and; **F)** mask use in a scenario where adherence increases to 85% of the population. **G-H**: COVID mortality data generated from reported daily deaths (blue); estimated based on reported hospitalizations (purple); estimated from reported cases (green); and via a spline fit through all available data types (red, 95% UI in pink). **I-R**: Box plots showing 1,000 draws of fixed effect coefficients in a multivariate regression fit to $log(beta)$.

91 Utah: SEIR fit comparison

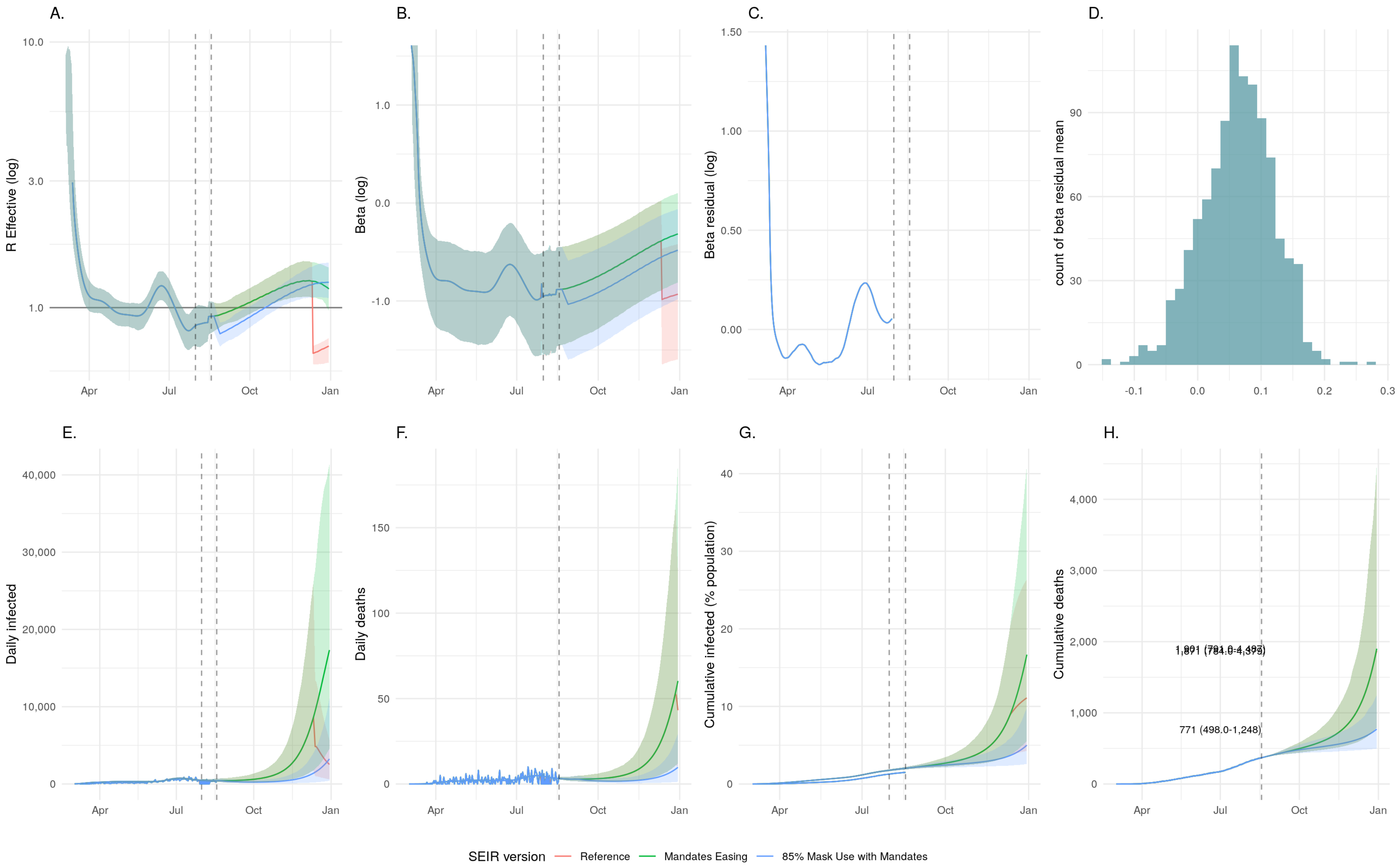

Utah: SEIR fit comparison. A: predicted R effective for each model through December 31. **B**: predicted SEIR *beta* parameter. **C**: residual of predicted *beta* and the observed value calculated directly from infection data over time. **D**: histogram of residual values for *beta*. Panels A, B, C, and D are all displayed in log space, reflecting the space in which the SEIR model is fit. **E**: predicted daily infections from each model through December 31. **F**: predicted daily deaths from each model through December 31. **G**: predicted cumulative infections through December 31, as a proportion of the total population. **H**: predicted cumulative deaths through December 31. In panels E, F, G, and H, reported death and infections are plotted alongside model predictions in light blue.

92 Utah: Covariate fits and regression coefficients

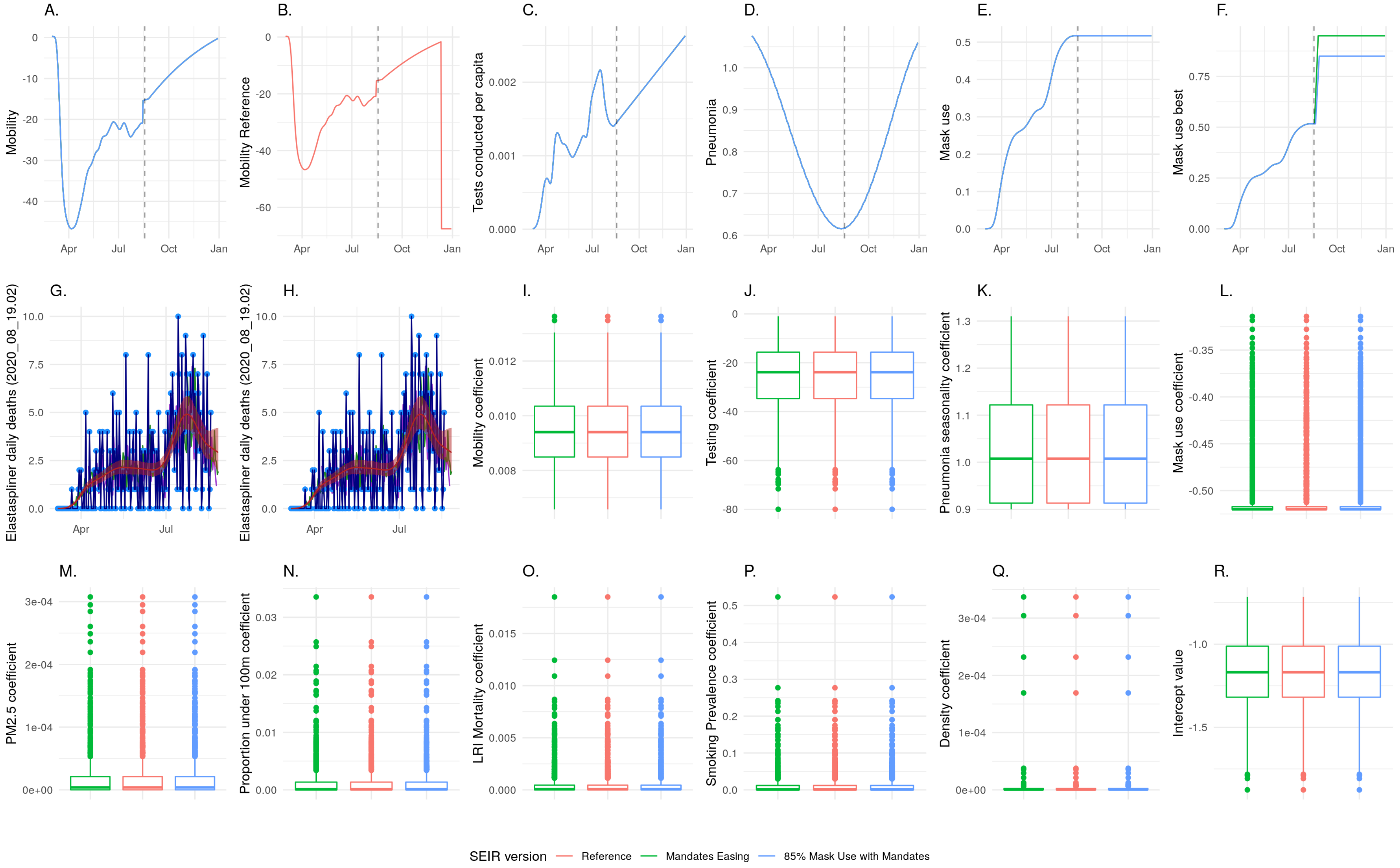

Utah: Covariate fits and regression coefficients. **A-F**: Line plots showing predicted covariate time trends for **A)** mobility in the absence of additional mandates; **B)** mobility with additional mandates applied; **C)** diagnostic testing per capita; **D)** pneumonia seasonality; **E)** mask use per capita, and; **F)** mask use in a scenario where adherence increases to 85% of the population. **G-H**: COVID mortality data generated from reported daily deaths (blue); estimated based on reported hospitalizations (purple); estimated from reported cases (green); and via a spline fit through all available data types (red, 95% UI in pink). **I-R**: Box plots showing 1,000 draws of fixed effect coefficients in a multivariate regression fit to $log(beta)$.

93 Vermont: SEIR fit comparison

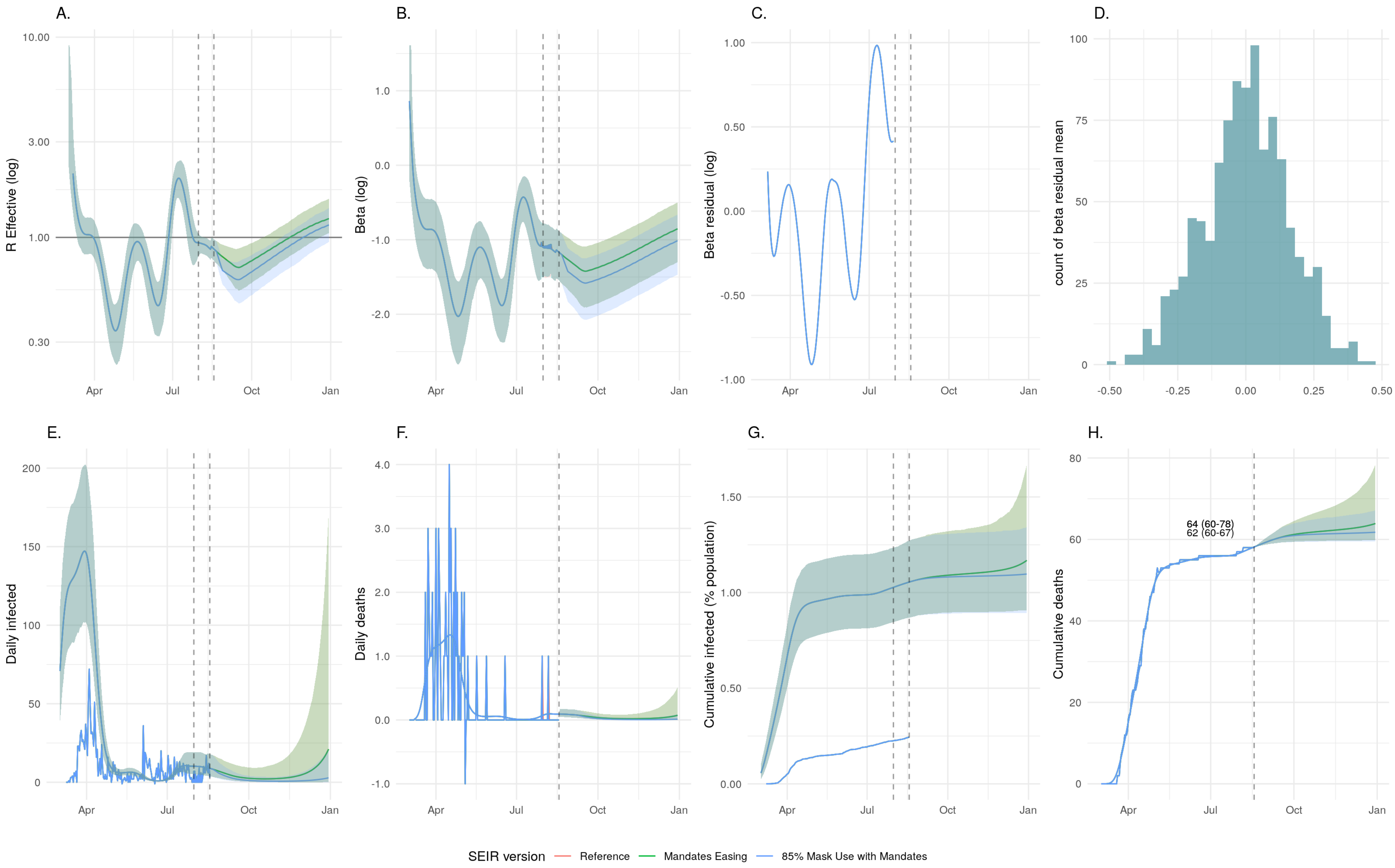

Vermont: SEIR fit comparison. A: predicted R effective for each model through December 31. **B**: predicted SEIR *beta* parameter. **C**: residual of predicted *beta* and the observed value calculated directly from infection data over time. **D**: histogram of residual values for *beta*. Panels A, B, C, and D are all displayed in log space, reflecting the space in which the SEIR model is fit. **E**: predicted daily infections from each model through December 31. **F**: predicted daily deaths from each model through December 31. **G**: predicted cumulative infections through December 31, as a proportion of the total population. **H**: predicted cumulative deaths through December 31. In panels E, F, G, and H, reported death and infections are plotted alongside model predictions in light blue.

94 Vermont: Covariate fits and regression coefficients

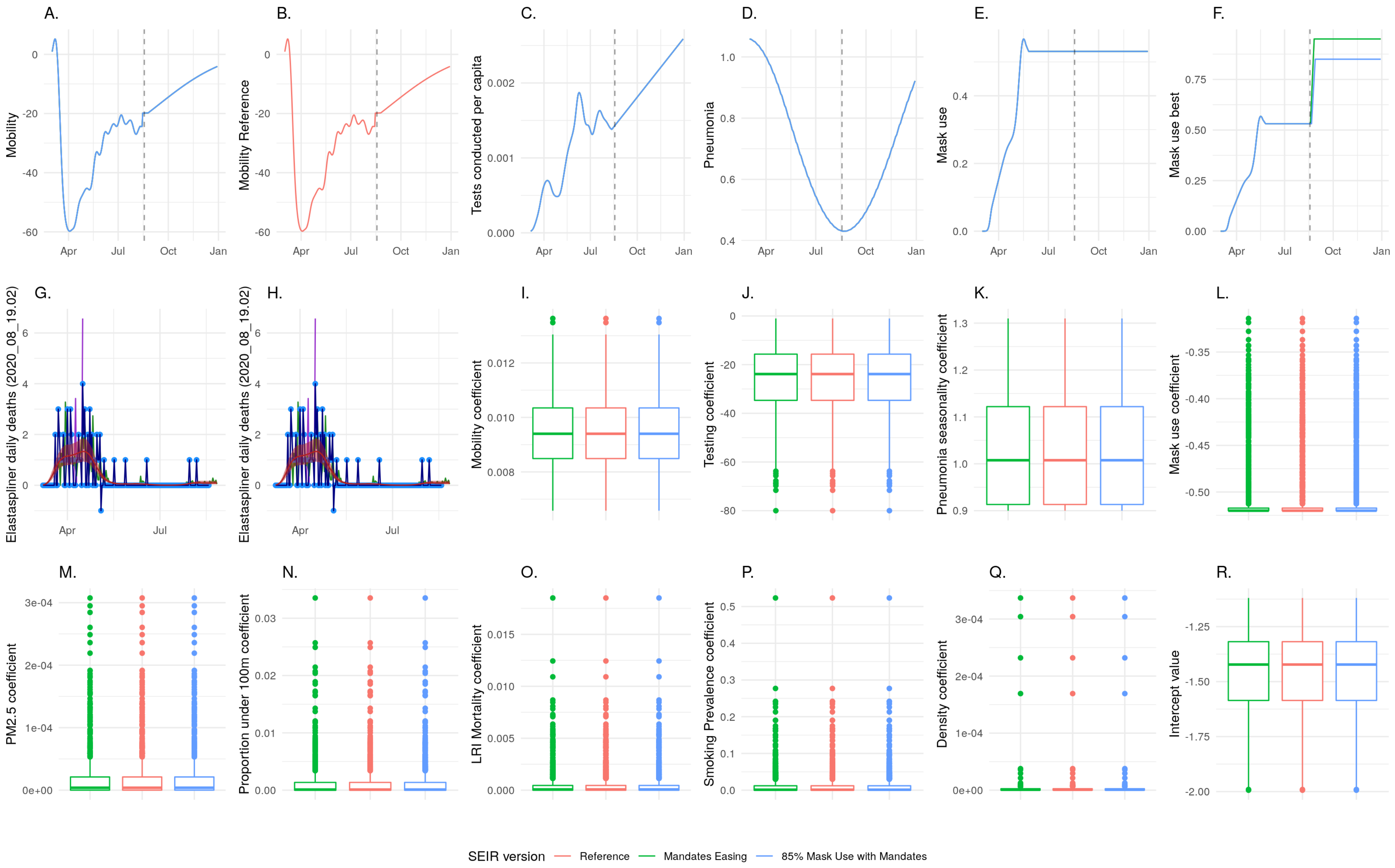

Vermont: Covariate fits and regression coefficients. **A-F**: Line plots showing predicted covariate time trends for **A)** mobility in the absence of additional mandates; **B)** mobility with additional mandates applied; **C)** diagnostic testing per capita; **D)** pneumonia seasonality; **E)** mask use per capita, and; **F)** mask use in a scenario where adherence increases to 85% of the population. **G-H**: COVID mortality data generated from reported daily deaths (blue); estimated based on reported hospitalizations (purple); estimated from reported cases (green); and via a spline fit through all available data types (red, 95% UI in pink). **I-R**: Box plots showing 1,000 draws of fixed effect coefficients in a multivariate regression fit to $log(beta)$.

95 Virginia: SEIR fit comparison

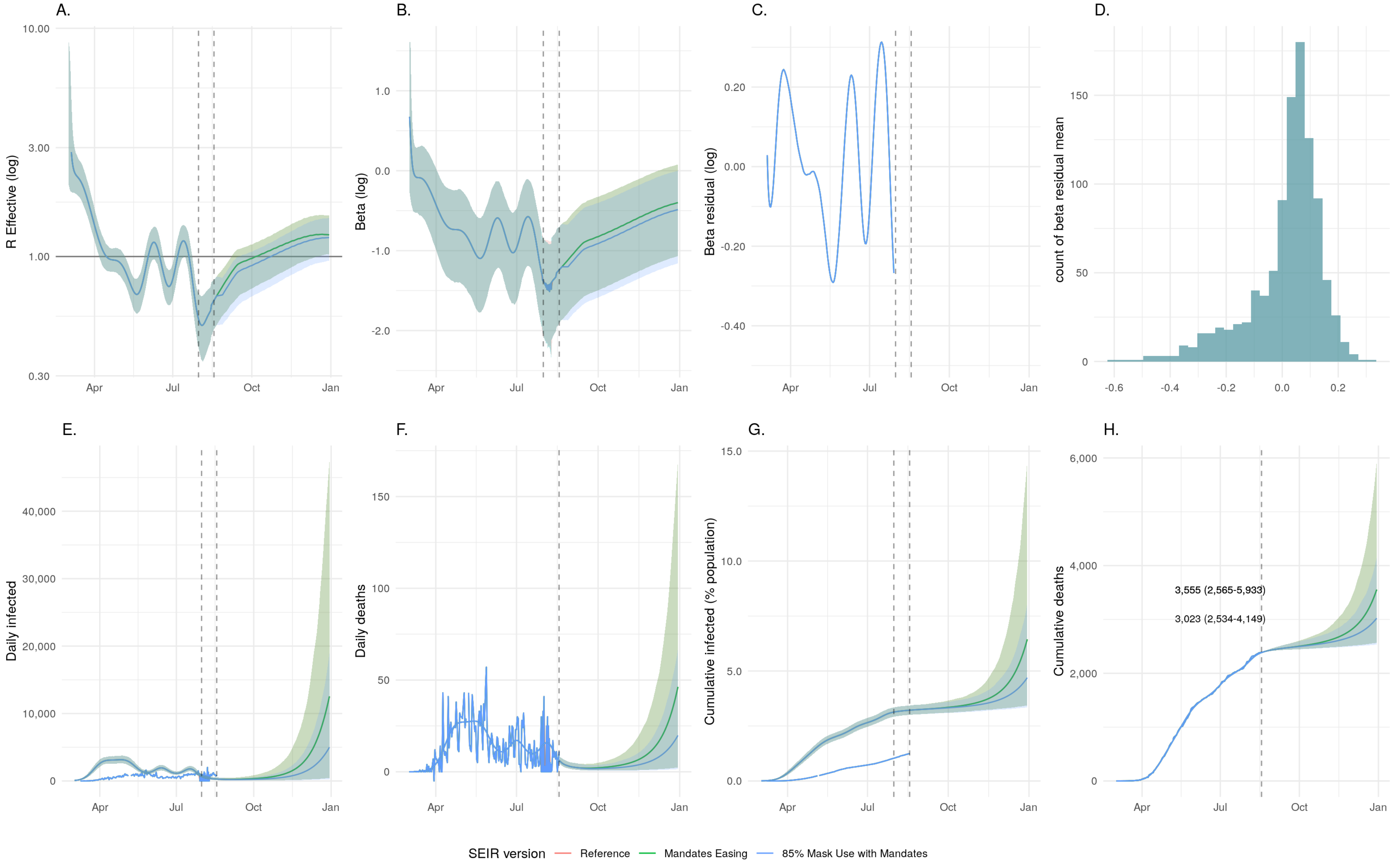

Virginia: SEIR fit comparison. A: predicted R effective for each model through December 31. **B**: predicted SEIR *beta* parameter. **C**: residual of predicted *beta* and the observed value calculated directly from infection data over time. **D**: histogram of residual values for *beta*. Panels A, B, C, and D are all displayed in log space, reflecting the space in which the SEIR model is fit. **E**: predicted daily infections from each model through December 31. **F**: predicted daily deaths from each model through December 31. **G**: predicted cumulative infections through December 31, as a proportion of the total population. **H**: predicted cumulative deaths through December 31. In panels E, F, G, and H, reported death and infections are plotted alongside model predictions in light blue.

96 Virginia: Covariate fits and regression coefficients

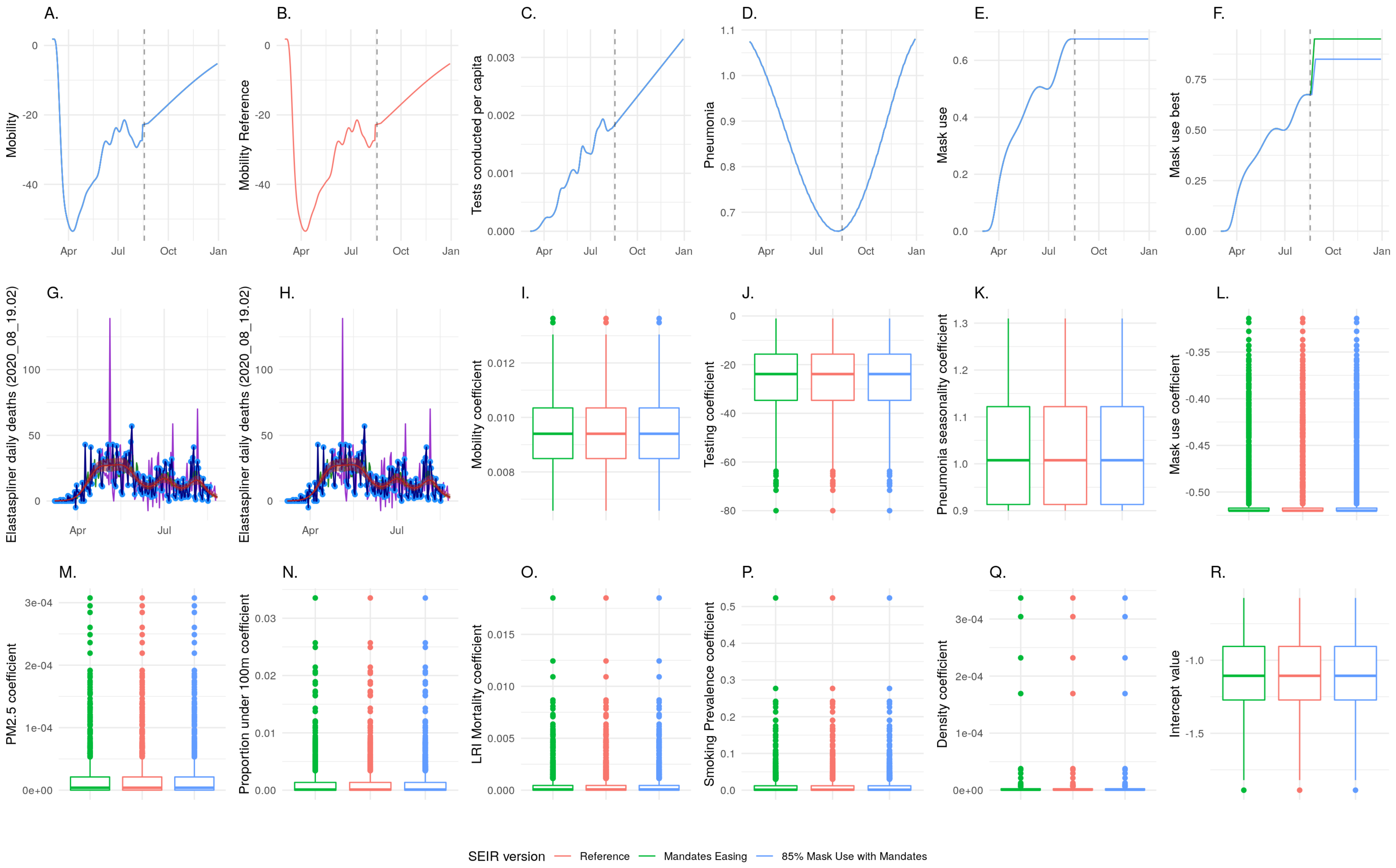

Virginia: Covariate fits and regression coefficients. A-F: Line plots showing predicted covariate time trends for **A)** mobility in the absence of additional mandates; **B)** mobility with additional mandates applied; **C)** diagnostic testing per capita; **D)** pneumonia seasonality; **E)** mask use per capita, and; **F)** mask use in a scenario where adherence increases to 85% of the population. **G-H**: COVID mortality data generated from reported daily deaths (blue); estimated based on reported hospitalizations (purple); estimated from reported cases (green); and via a spline fit through all available data types (red, 95% UI in pink). **I-R**: Box plots showing 1,000 draws of fixed effect coefficients in a multivariate regression fit to $log(beta)$.

97 Washington: SEIR fit comparison

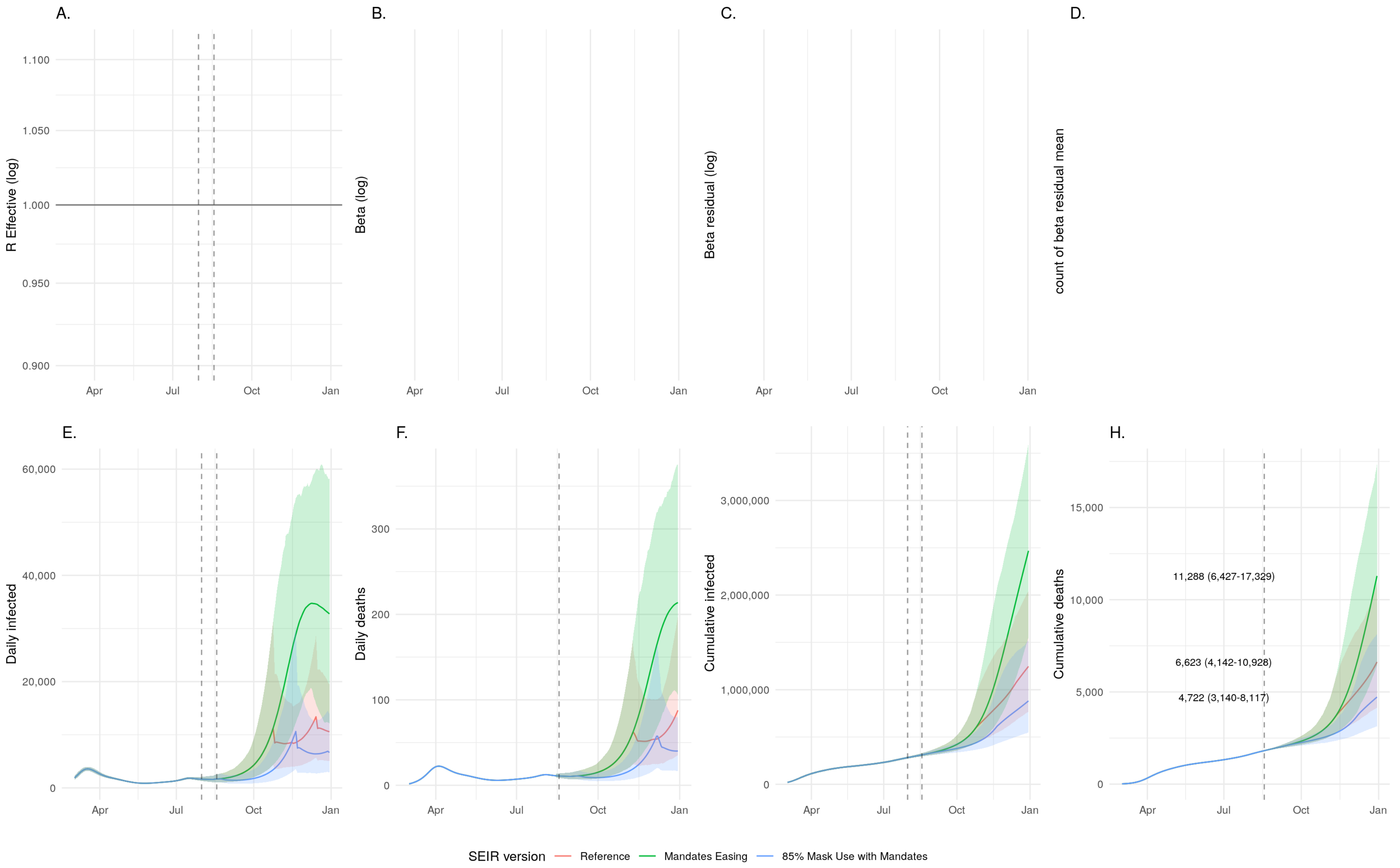

Washington: SEIR fit comparison. Panels **A-D** display values that are not directly calculated for aggregate locations. **E**: predicted daily infections from each model through December 31. **F**: predicted daily deaths from each model through December 31. **G**: predicted cumulative infections through December 31, as a proportion of the total population. **H**: predicted cumulative deaths through December 31. In panels E, F, G, and H, reported death and infections are plotted alongside model predictions in light blue.

98 Washington: Covariate fits and regression coefficients

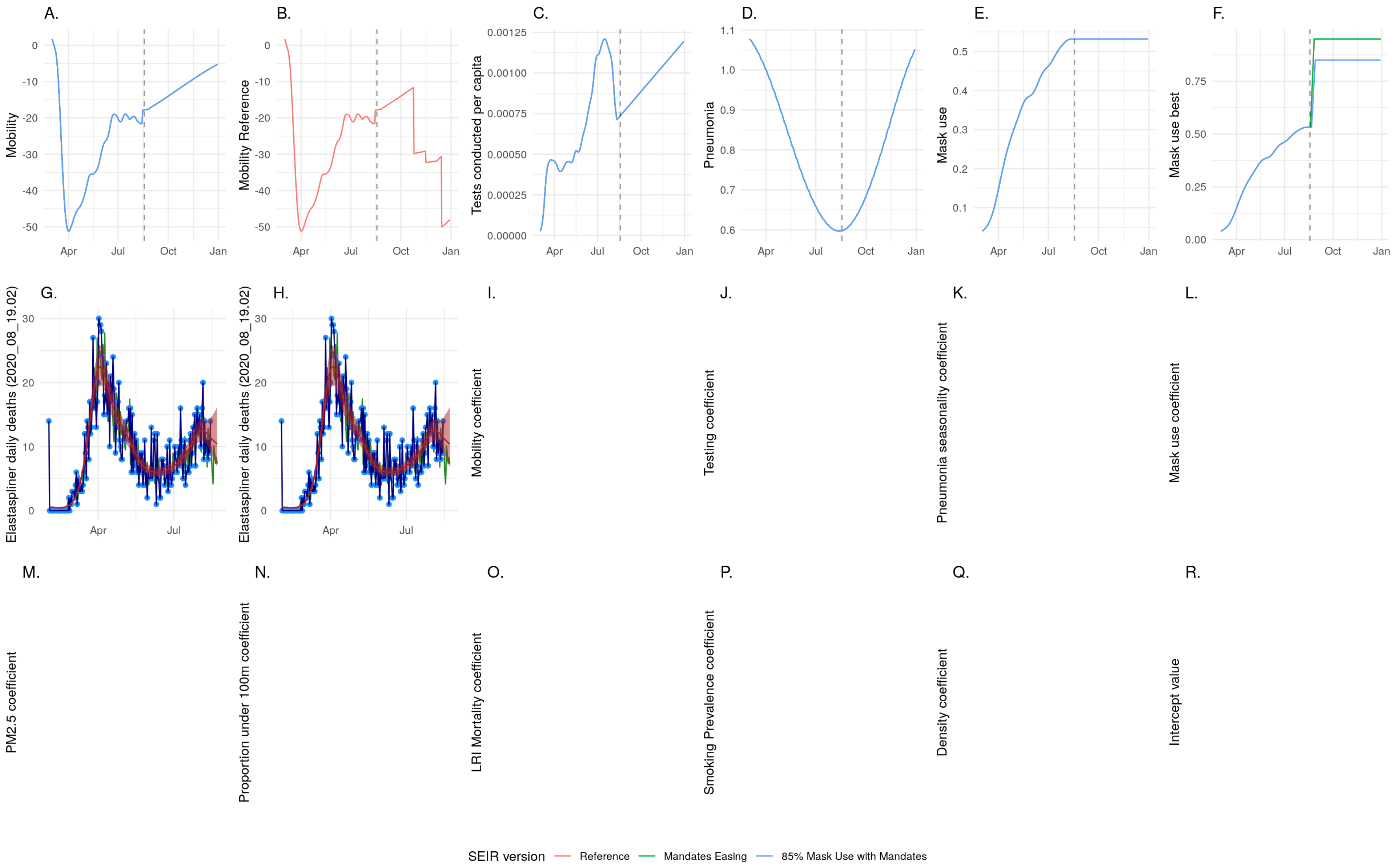

Washington: Covariate fits and regression coefficients. **A-F**: Line plots showing predicted covariate time trends for **A)** mobility in the absence of additional mandates; **B)** mobility with additional mandates applied; **C)** diagnostic testing per capita; **D)** pneumonia seasonality; **E)** mask use per capita, and; **F)** mask use in a scenario where adherence increases to 85% of the population. **G-H**: COVID mortality data generated from reported daily deaths (blue); estimated based on reported hospitalizations (purple); estimated from reported cases (green); and via a spline fit through all available data types (red, 95% UI in pink). Panels **I-R** display coefficients for a regression fit to $log(beta)$, which is not applicable to aggregate locations.

99 West Virginia: SEIR fit comparison

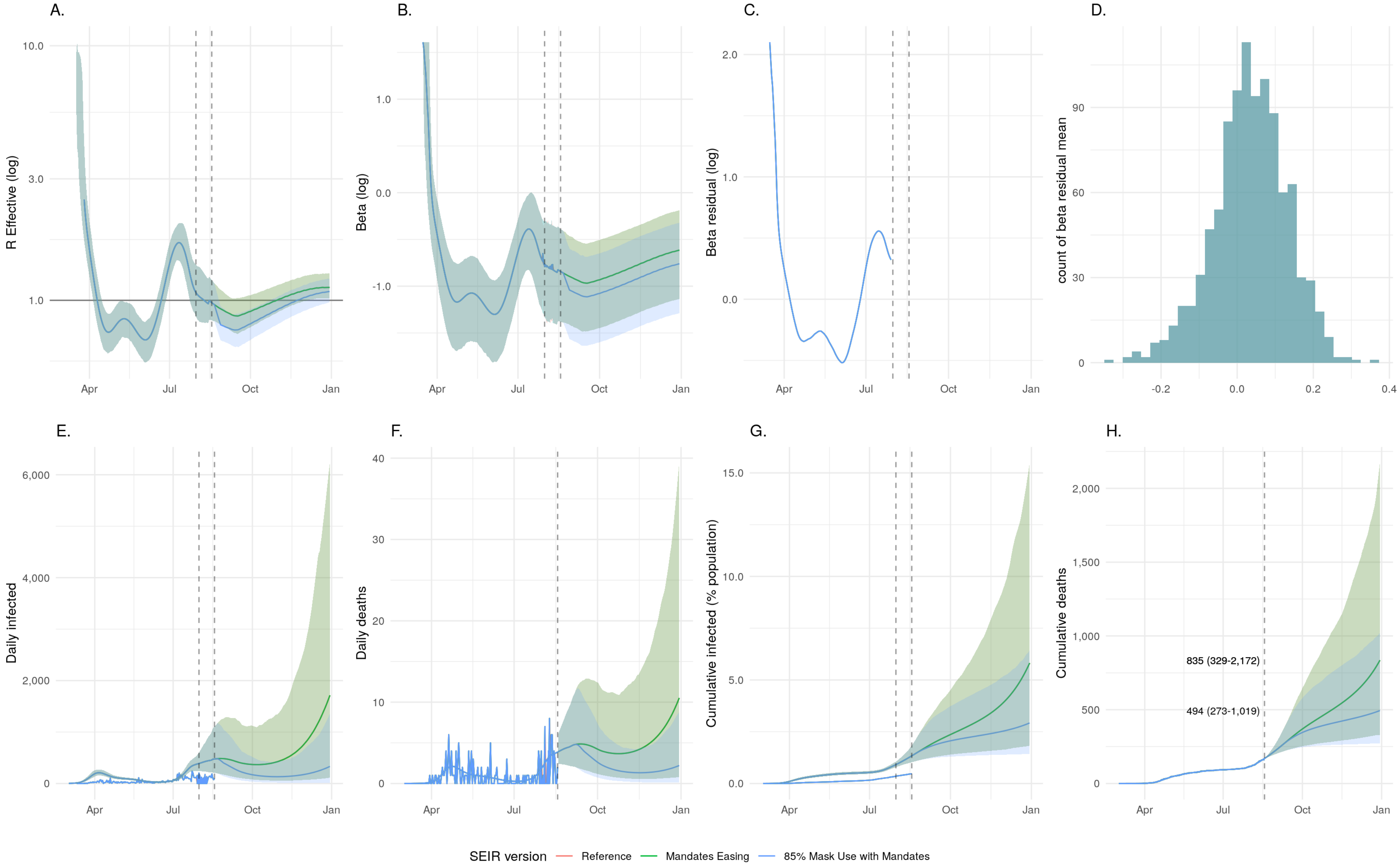

West Virginia: SEIR fit comparison. **A**: predicted R effective for each model through December 31. **B**: predicted SEIR *beta* parameter. **C**: residual of predicted *beta* and the observed value calculated directly from infection data over time. **D**: histogram of residual values for *beta*. Panels A, B, C, and D are all displayed in log space, reflecting the space in which the SEIR model is fit. **E**: predicted daily infections from each model through December 31. **F**: predicted daily deaths from each model through December 31. **G**: predicted cumulative infections through December 31, as a proportion of the total population. **H**: predicted cumulative deaths through December 31. In panels E, F, G, and H, reported death and infections are plotted alongside model predictions in light blue.

100 West Virginia: Covariate fits and regression coefficients

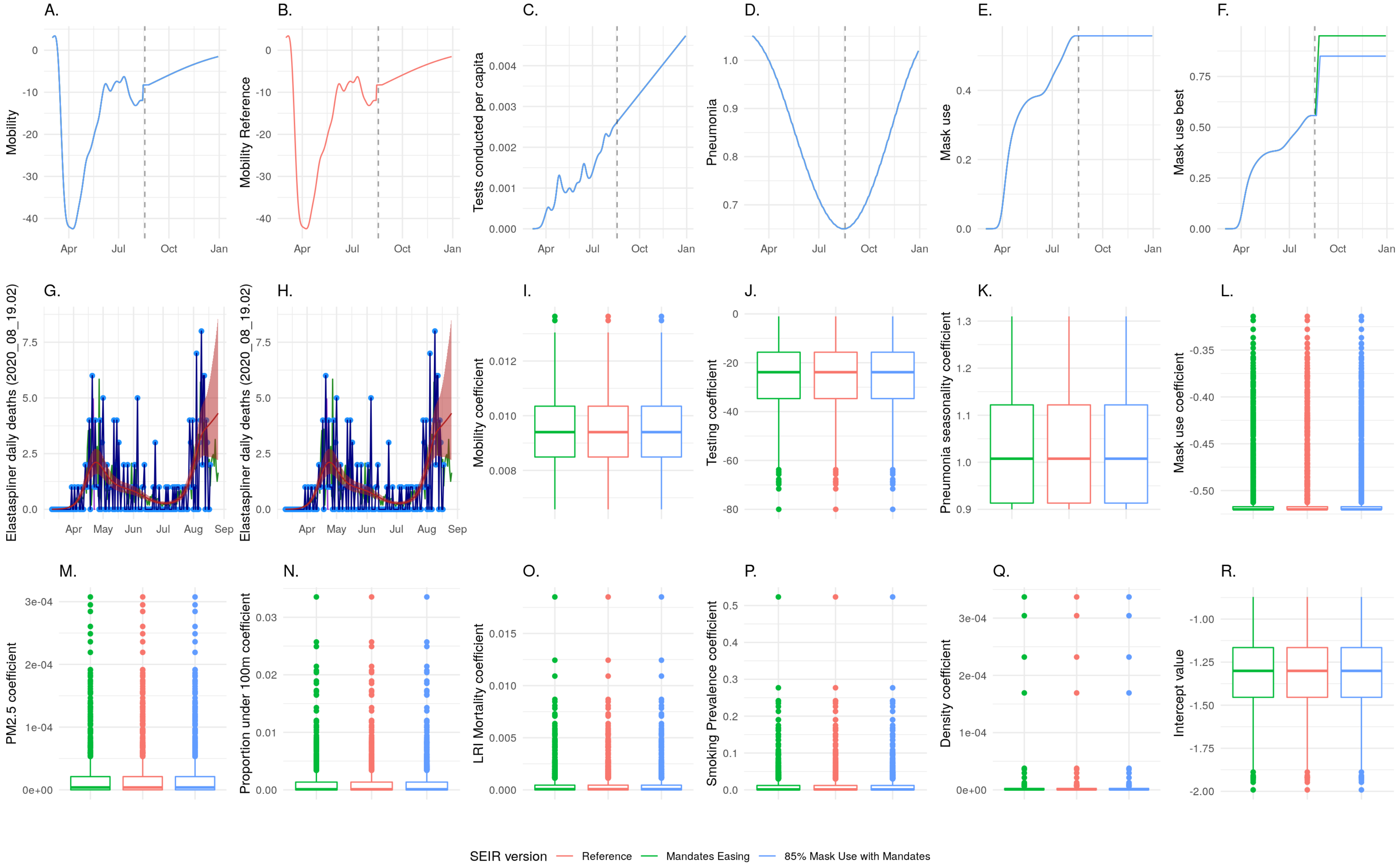

West Virginia: Covariate fits and regression coefficients. A-F: Line plots showing predicted covariate time trends for **A)** mobility in the absence of additional mandates; **B)** mobility with additional mandates applied; **C)** diagnostic testing per capita; **D)** pneumonia seasonality; **E)** mask use per capita, and; **F)** mask use in a scenario where adherence increases to 85% of the population. **G-H**: COVID mortality data generated from reported daily deaths (blue); estimated based on reported hospitalizations (purple); estimated from reported cases (green); and via a spline fit through all available data types (red, 95% UI in pink). **I-R**: Box plots showing 1,000 draws of fixed effect coefficients in a multivariate regression fit to $log(beta)$.

101 Wisconsin: SEIR fit comparison

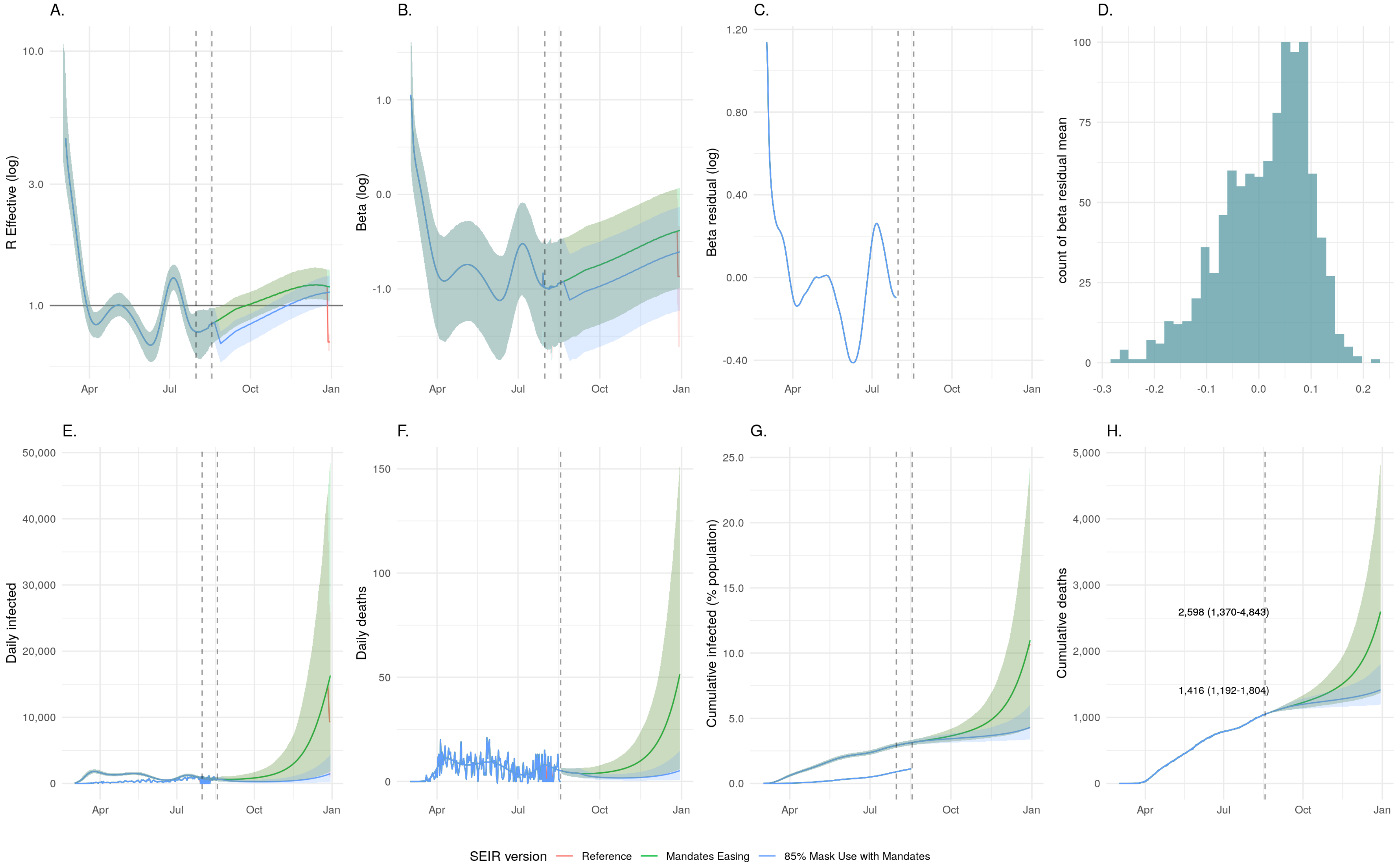

Wisconsin: SEIR fit comparison. A: predicted R effective for each model through December 31. **B**: predicted SEIR *beta* parameter. **C**: residual of predicted *beta* and the observed value calculated directly from infection data over time. **D**: histogram of residual values for *beta*. Panels A, B, C, and D are all displayed in log space, reflecting the space in which the SEIR model is fit. **E**: predicted daily infections from each model through December 31. **F**: predicted daily deaths from each model through December 31. **G**: predicted cumulative infections through December 31, as a proportion of the total population. **H**: predicted cumulative deaths through December 31. In panels E, F, G, and H, reported death and infections are plotted alongside model predictions in light blue.

102 Wisconsin: Covariate fits and regression coefficients

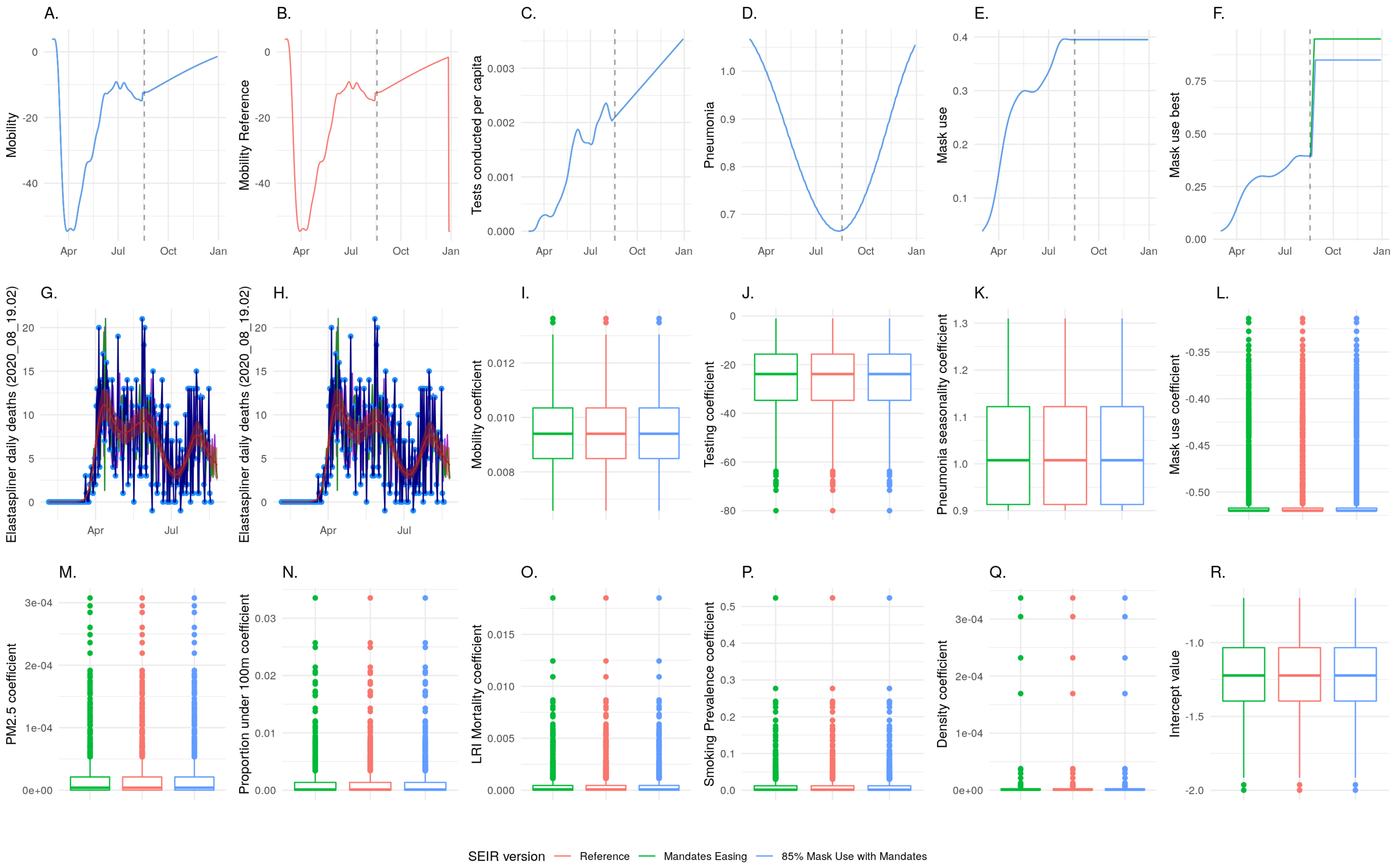

Wisconsin: Covariate fits and regression coefficients. A-F: Line plots showing predicted covariate time trends for **A)** mobility in the absence of additional mandates; **B)** mobility with additional mandates applied; **C)** diagnostic testing per capita; **D)** pneumonia seasonality; **E)** mask use per capita, and; **F)** mask use in a scenario where adherence increases to 85% of the population. **G-H**: COVID mortality data generated from reported daily deaths (blue); estimated based on reported hospitalizations (purple); estimated from reported cases (green); and via a spline fit through all available data types (red, 95% UI in pink). **I-R**: Box plots showing 1,000 draws of fixed effect coefficients in a multivariate regression fit to $log(beta)$.

103 Wyoming: SEIR fit comparison

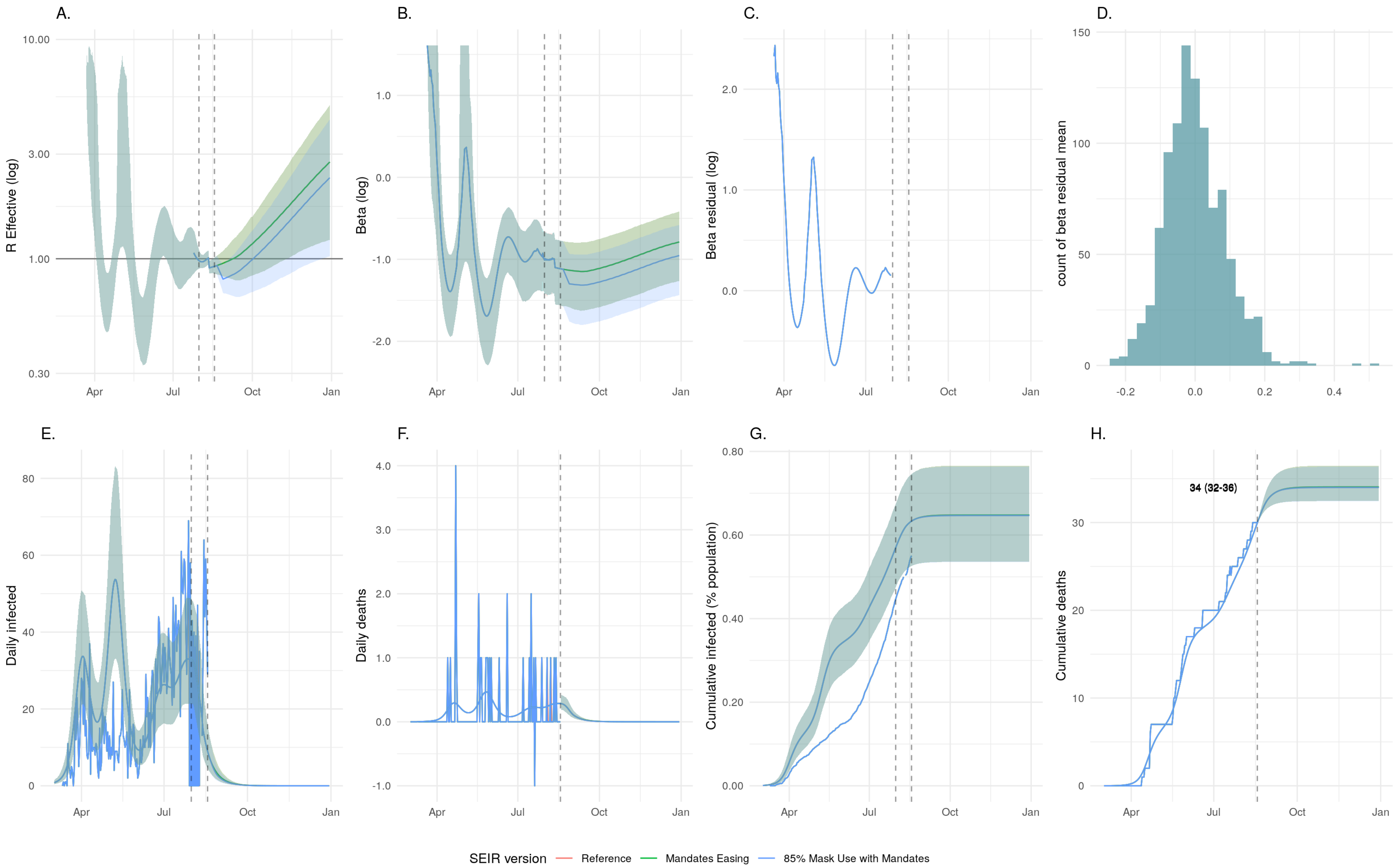

Wyoming: SEIR fit comparison. A: predicted R effective for each model through December 31. **B**: predicted SEIR *beta* parameter. **C**: residual of predicted *beta* and the observed value calculated directly from infection data over time. **D**: histogram of residual values for *beta*. Panels A, B, C, and D are all displayed in log space, reflecting the space in which the SEIR model is fit. **E**: predicted daily infections from each model through December 31. **F**: predicted daily deaths from each model through December 31. **G**: predicted cumulative infections through December 31, as a proportion of the total population. **H**: predicted cumulative deaths through December 31. In panels E, F, G, and H, reported death and infections are plotted alongside model predictions in light blue.

104 Wyoming: Covariate fits and regression coefficients

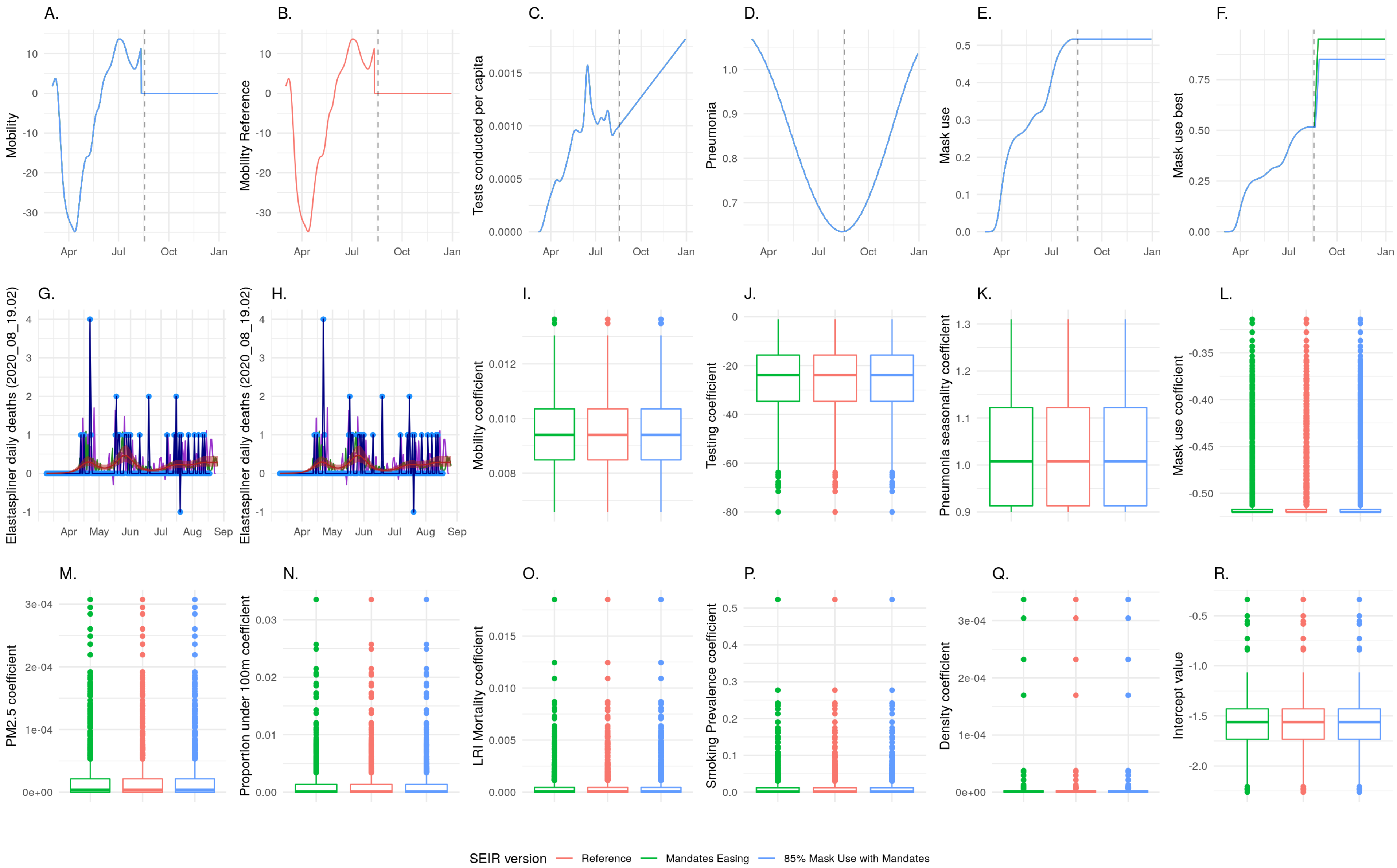

Wyoming: Covariate fits and regression coefficients. **A-F**: Line plots showing predicted covariate time trends for **A)** mobility in the absence of additional mandates; **B)** mobility with additional mandates applied; **C)** diagnostic testing per capita; **D)** pneumonia seasonality; **E)** mask use per capita, and; **F)** mask use in a scenario where adherence increases to 85% of the population. **G-H**: COVID mortality data generated from reported daily deaths (blue); estimated based on reported hospitalizations (purple); estimated from reported cases (green); and via a spline fit through all available data types (red, 95% UI in pink). **I-R**: Box plots showing 1,000 draws of fixed effect coefficients in a multivariate regression fit to $log(beta)$.

105 Spokane County: SEIR fit comparison

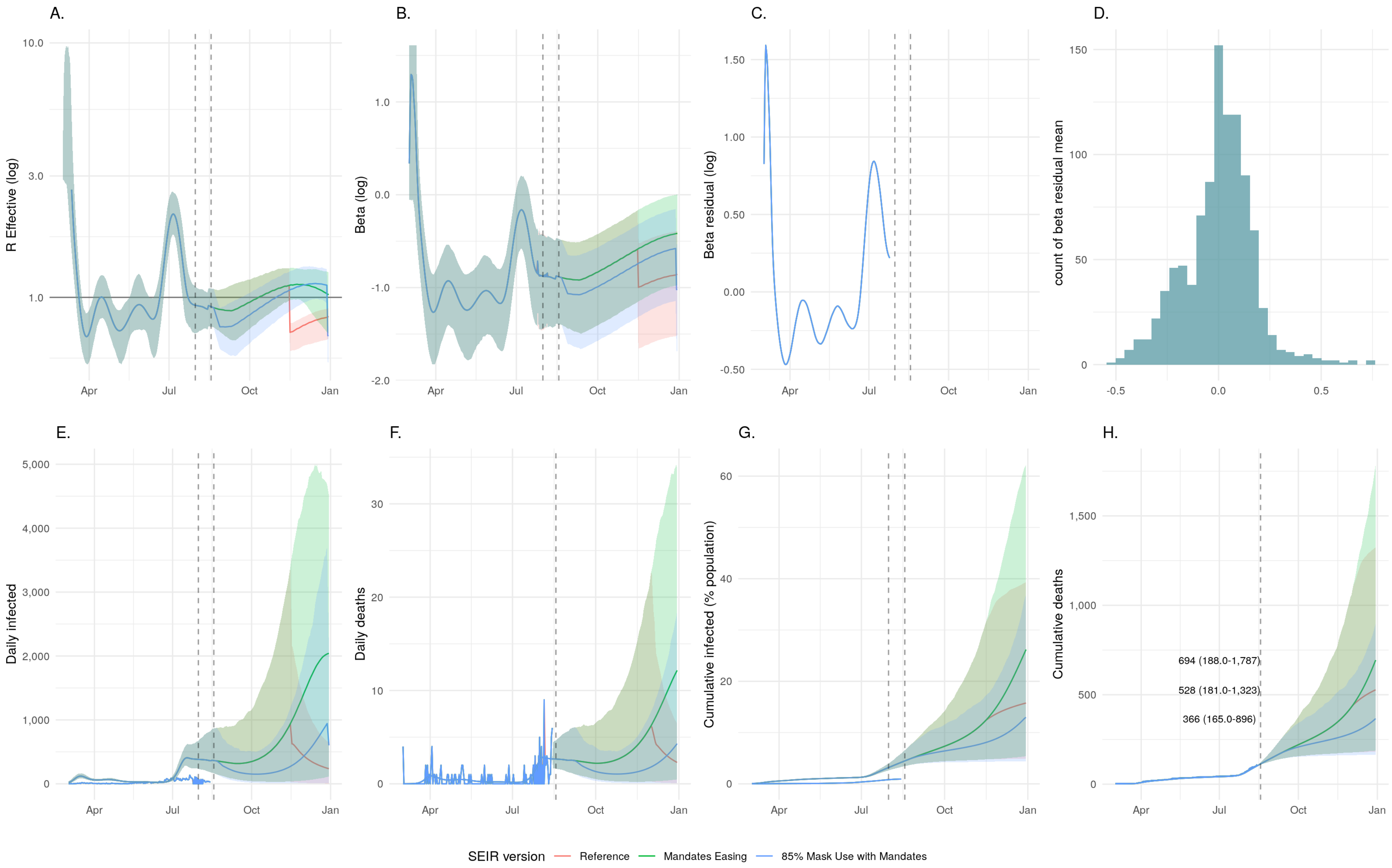

Spokane County: SEIR fit comparison. **A**: predicted R effective for each model through December 31. **B**: predicted SEIR *beta* parameter. **C**: residual of predicted *beta* and the observed value calculated directly from infection data over time. **D**: histogram of residual values for *beta*. Panels A, B, C, and D are all displayed in log space, reflecting the space in which the SEIR model is fit. **E**: predicted daily infections from each model through December 31. **F**: predicted daily deaths from each model through December 31. **G**: predicted cumulative infections through December 31, as a proportion of the total population. **H**: predicted cumulative deaths through December 31. In panels E, F, G, and H, reported death and infections are plotted alongside model predictions in light blue.

106 Spokane County: Covariate fits and regression coefficients

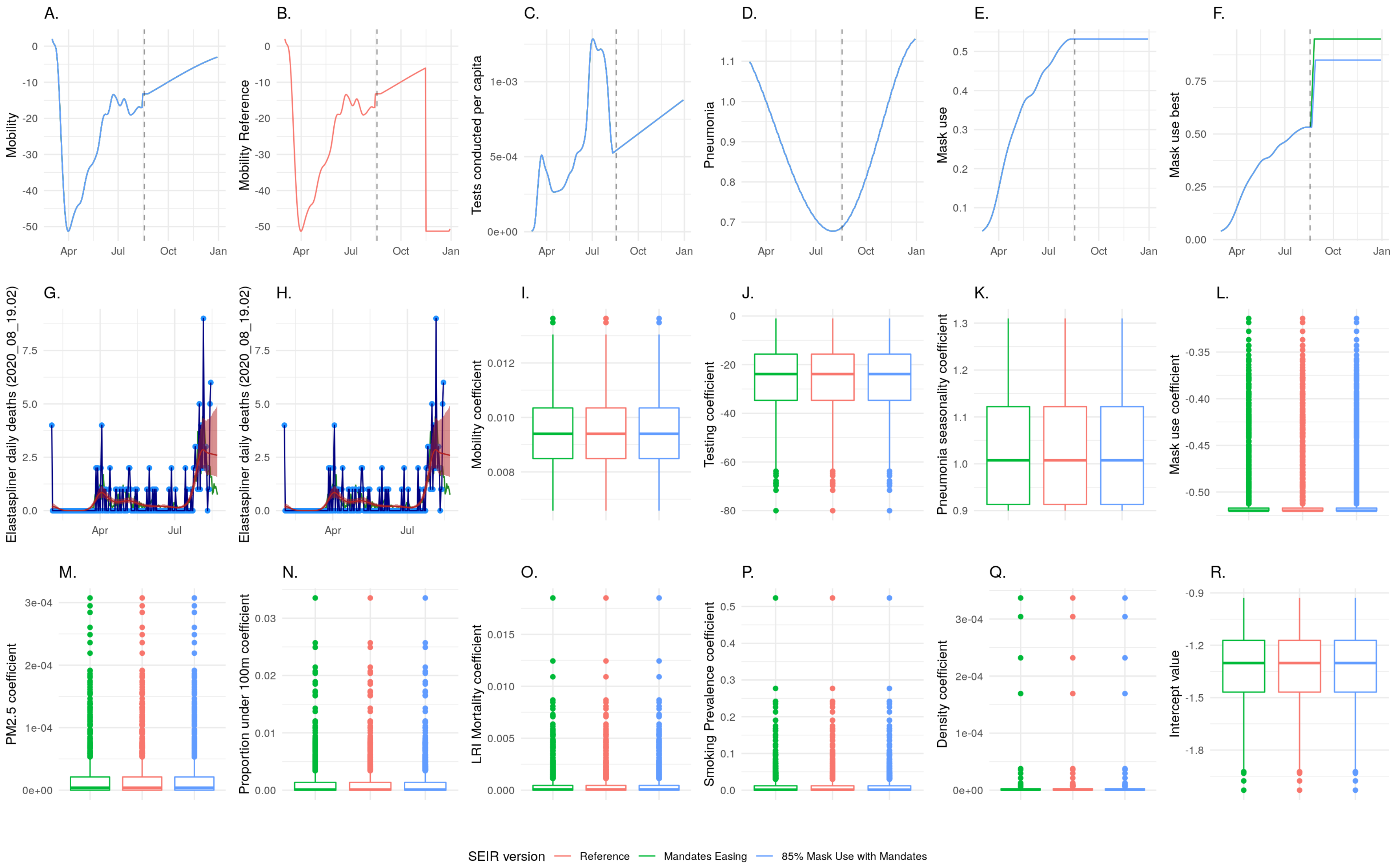

Spokane County: Covariate fits and regression coefficients. A-F: Line plots showing predicted covariate time trends for **A)** mobility in the absence of additional mandates; **B)** mobility with additional mandates applied; **C)** diagnostic testing per capita; **D)** pneumonia seasonality; **E)** mask use per capita, and; **F)** mask use in a scenario where adherence increases to 85% of the population. **G-H**: COVID mortality data generated from reported daily deaths (blue); estimated based on reported hospitalizations (purple); estimated from reported cases (green); and via a spline fit through all available data types (red, 95% UI in pink). **I-R**: Box plots showing 1,000 draws of fixed effect coefficients in a multivariate regression fit to $log(beta)$.

107 King and Snohomish Counties: SEIR fit comparison

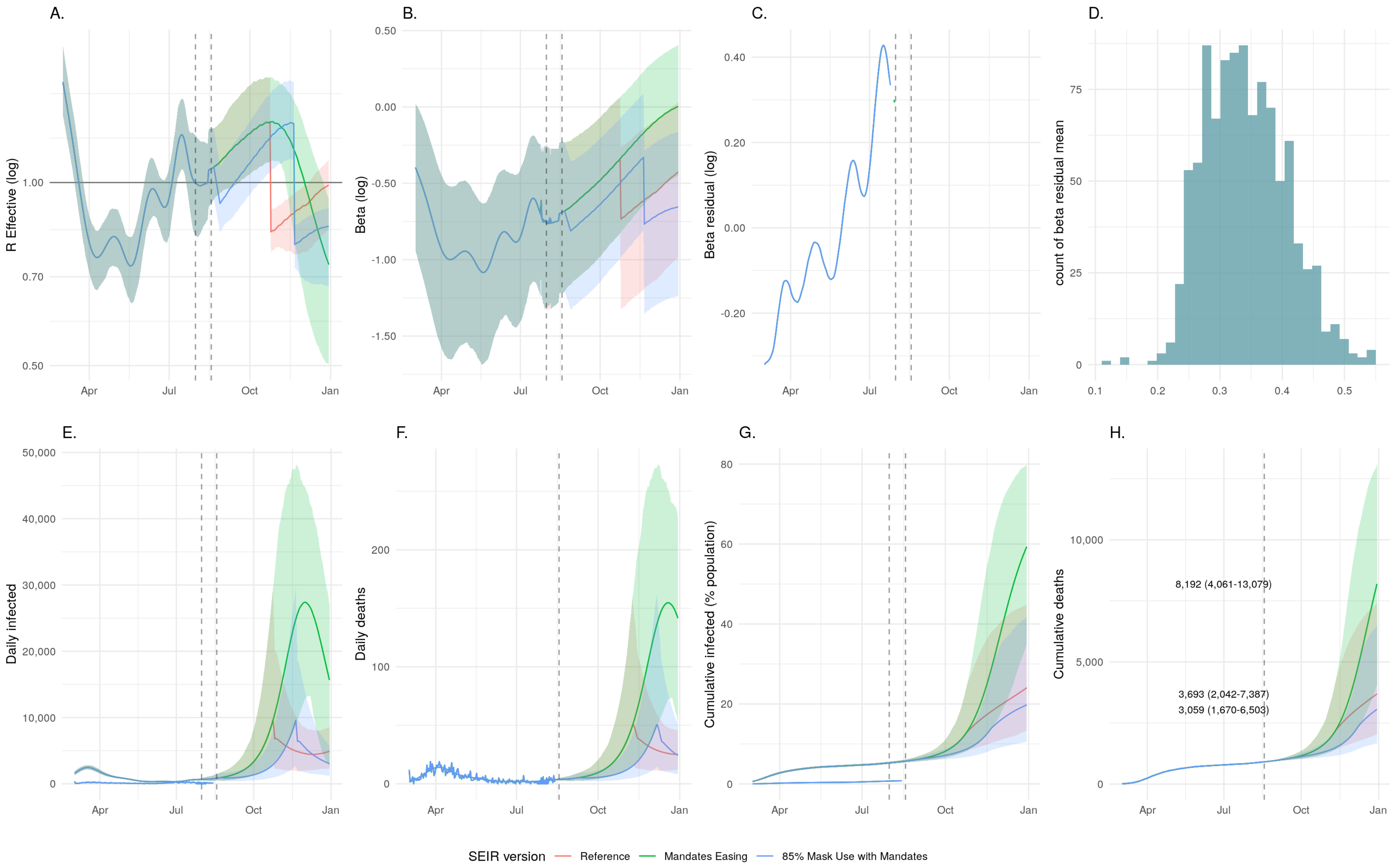

King and Snohomish Counties: SEIR fit comparison. **A**: predicted R effective for each model through December 31. **B**: predicted SEIR *beta* parameter. **C**: residual of predicted *beta* and the observed value calculated directly from infection data over time. **D**: histogram of residual values for *beta*. Panels A, B, C, and D are all displayed in log space, reflecting the space in which the SEIR model is fit. **E**: predicted daily infections from each model through December 31. **F**: predicted daily deaths from each model through December 31. **G**: predicted cumulative infections through December 31, as a proportion of the total population. **H**: predicted cumulative deaths through December 31. In panels E, F, G, and H, reported death and infections are plotted alongside model predictions in light blue.

108 King and Snohomish Counties: Covariate fits and regression coefficients

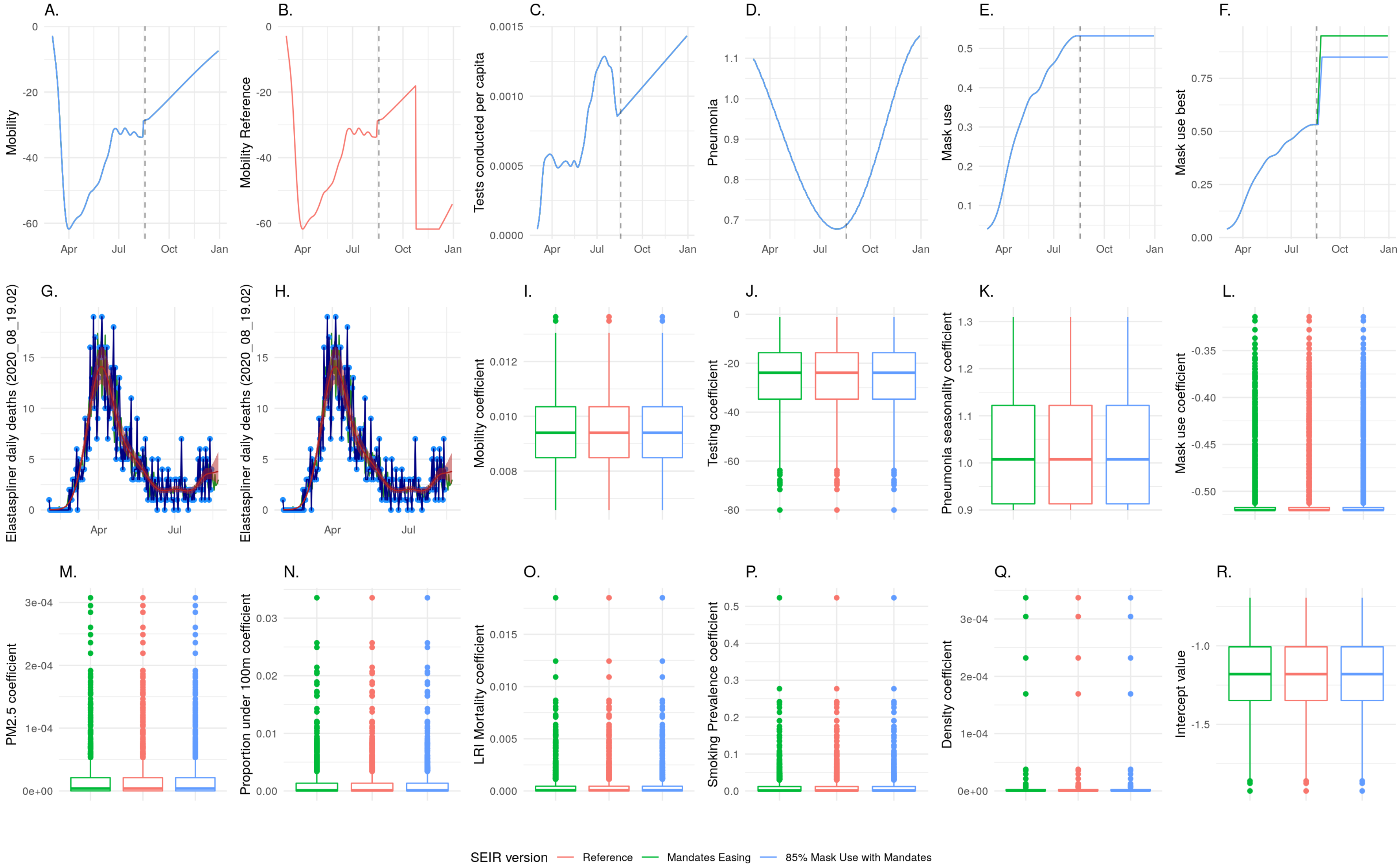

King and Snohomish Counties: Covariate fits and regression coefficients. A-F: Line plots showing predicted covariate time trends for **A)** mobility in the absence of additional mandates; **B)** mobility with additional mandates applied; **C)** diagnostic testing per capita; **D)** pneumonia seasonality; **E)** mask use per capita, and; **F)** mask use in a scenario where adherence increases to 85% of the population. **G-H**: COVID mortality data generated from reported daily deaths (blue); estimated based on reported hospitalizations (purple); estimated from reported cases (green); and via a spline fit through all available data types (red, 95% UI in pink). **I-R**: Box plots showing 1,000 draws of fixed effect coefficients in a multivariate regression fit to $log(beta)$.

109 Washington except for King, Snohomish, and Spokane Counties: SEIR fit comparison

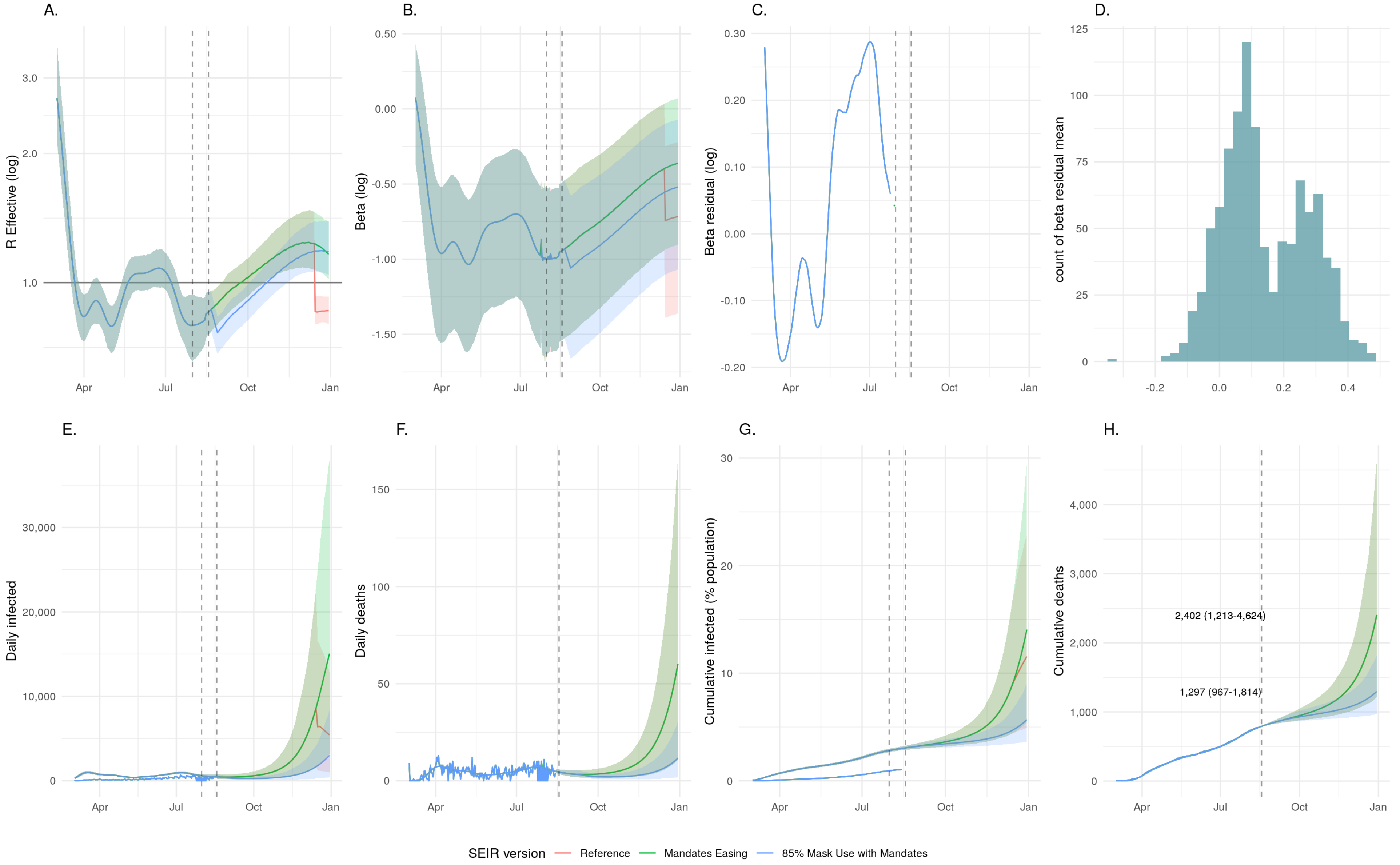

Washington except for King, Snohomish, and Spokane Counties: SEIR fit comparison. A: predicted R effective for each model through December 31. **B**: predicted SEIR *beta* parameter. **C**: residual of predicted *beta* and the observed value calculated directly from infection data over time. **D**: histogram of residual values for *beta*. Panels A, B, C, and D are all displayed in log space, reflecting the space in which the SEIR model is fit. **E**: predicted daily infections from each model through December 31. **F**: predicted daily deaths from each model through December 31. **G**: predicted cumulative infections through December 31, as a proportion of the total population. **H**: predicted cumulative deaths through December 31. In panels E, F, G, and H, reported death and infections are plotted alongside model predictions in

light blue.

110 Washington except for King, Snohomish, and Spokane Counties: Covariate fits and regression coefficients

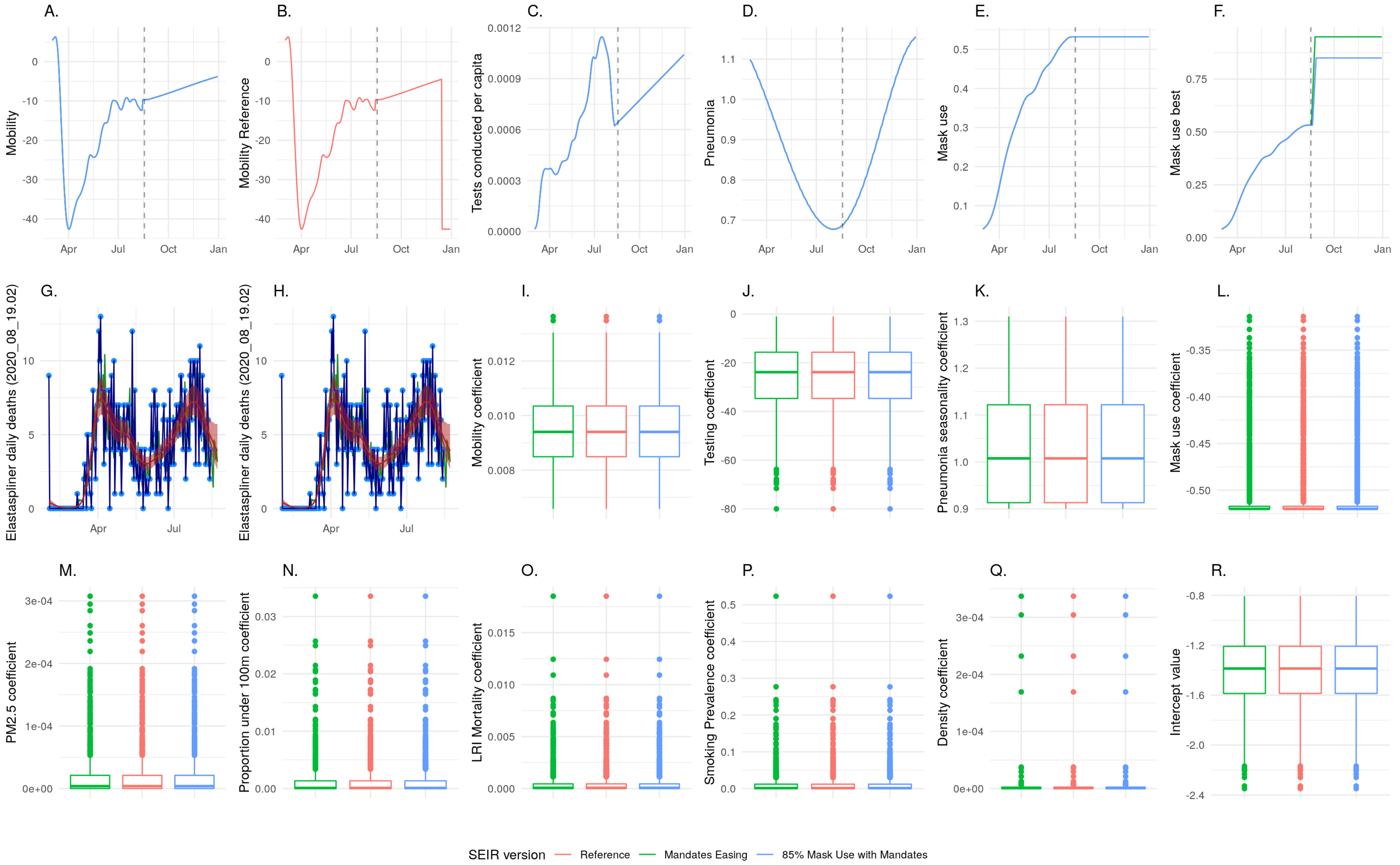

Washington except for King, Snohomish, and Spokane Counties: Covariate fits and regression coefficients. A-F: Line plots showing predicted covariate time trends for **A)** mobility in the absence of additional mandates; **B)** mobility with additional mandates applied; **C)** diagnostic testing per capita; **D)** pneumonia seasonality; **E)** mask use per capita, and; **F)** mask use in a scenario where adherence increases to 85% of the population. **G-H**: COVID mortality data generated from

reported daily deaths (blue); estimated based on reported hospitalizations (purple); estimated from reported cases (green); and via a spline fit through all available data types (red, 95% UI in pink). **I-R**: Box plots showing 1,000 draws of fixed effect coefficients in a multivariate regression fit to $log(beta)$.
