# Supplementary material for: Modeling COVID-19 scenarios for the United States
Source: Nat Med. 2020 Oct 23;27(1):94–105. doi: 10.1038/s41591-020-1132-9 (PMC7806509; doi:10.1038/s41591-020-1132-9)
Supplement: Supplementary file 6 — Appendix 4: detailed SEIR regression diagnostics. [file 41591_2020_1132_MOESM6_ESM.pdf]

Appendix 4:
Detailed SEIR Regression Diagnostics

Contents

1 Alabama: Detailed regression diagnostics **4**

2 Alaska: Detailed regression diagnostics **5**

3 Arizona: Detailed regression diagnostics **6**

4 Arkansas: Detailed regression diagnostics **7**

5 California: Detailed regression diagnostics **8**

6 Colorado: Detailed regression diagnostics **9**

7 Connecticut: Detailed regression diagnostics **10**

8 Delaware: Detailed regression diagnostics **11**

9 District of Columbia: Detailed regression diagnostics **12**

10 Florida: Detailed regression diagnostics **13**

11 Georgia: Detailed regression diagnostics **14**

12 Hawaii: Detailed regression diagnostics **15**

13 Idaho: Detailed regression diagnostics **16**

14 Illinois: Detailed regression diagnostics **17**

15 Indiana: Detailed regression diagnostics **18**

16 Iowa: Detailed regression diagnostics **19**

17 Kansas: Detailed regression diagnostics **20**

18 Kentucky: Detailed regression diagnostics **21**

19 Louisiana: Detailed regression diagnostics **22**

20 Maine: Detailed regression diagnostics **23**

21 Maryland: Detailed regression diagnostics **24**

22 Massachusetts: Detailed regression diagnostics **25**

23 Michigan: Detailed regression diagnostics **26**

24 Minnesota: Detailed regression diagnostics **27**

25 Mississippi: Detailed regression diagnostics **28**

26 Missouri: Detailed regression diagnostics **29**

27 Montana: Detailed regression diagnostics **30**

28 Nebraska: Detailed regression diagnostics **31**

29 Nevada: Detailed regression diagnostics **32**

30 New Hampshire: Detailed regression diagnostics **33**

31 New Jersey: Detailed regression diagnostics **34**

32 New Mexico: Detailed regression diagnostics **35**

33 New York: Detailed regression diagnostics **36**

34 North Carolina: Detailed regression diagnostics **37**

35 North Dakota: Detailed regression diagnostics **38**

36 Ohio: Detailed regression diagnostics **39**

37 Oklahoma: Detailed regression diagnostics **40**

38 Oregon: Detailed regression diagnostics **41**

39 Pennsylvania: Detailed regression diagnostics **42**

40 Rhode Island: Detailed regression diagnostics **43**

41 South Carolina: Detailed regression diagnostics **44**

42 South Dakota: Detailed regression diagnostics **45**

43 Tennessee: Detailed regression diagnostics **46**

44 Texas: Detailed regression diagnostics **47**

45 Utah: Detailed regression diagnostics **48**

46 Vermont: Detailed regression diagnostics **49**

47 Virginia: Detailed regression diagnostics **50**

48 West Virginia: Detailed regression diagnostics **51**

49 Wisconsin: Detailed regression diagnostics **52**

50 Wyoming: Detailed regression diagnostics **53**

51 Spokane County: Detailed regression diagnostics **54**

52 King and Snohomish Counties: Detailed regression diagnostics **55**

53 Washington except for King, Snohomish, and Spokane Counties: Detailed regression diagnostics **56**

1 Alabama: Detailed regression diagnostics

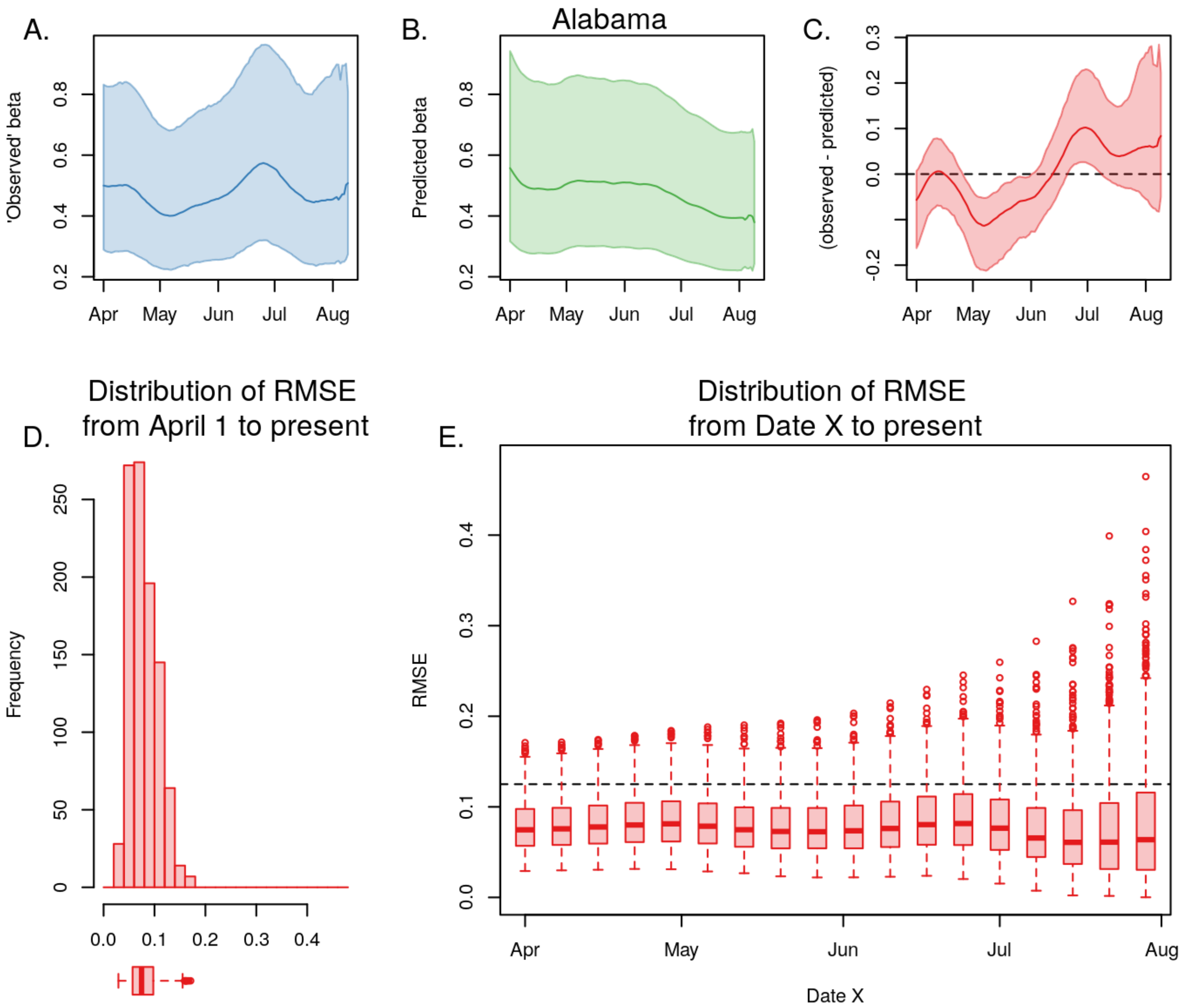

Alabama: Detailed regression diagnostics. A: The SEIR *beta* parameter calculated directly from past input data on infections. **B:** The *beta* parameter predicted using a multivariate regression across all locations. **C:** The difference between the directly-calculated and predicted values for *beta*. Mean and uncertainty interval are shown across 1,000 posterior predictive draws over time. **D:** Histogram and box plot showing the distribution of root mean squared error (RMSE) for *beta* when aggregated across all dates from April 1 to present. **E:** Box plots showing the RMSE for aggregates of *beta* from a given date to the present across 1,000 posterior predictive draws.

2 Alaska: Detailed regression diagnostics

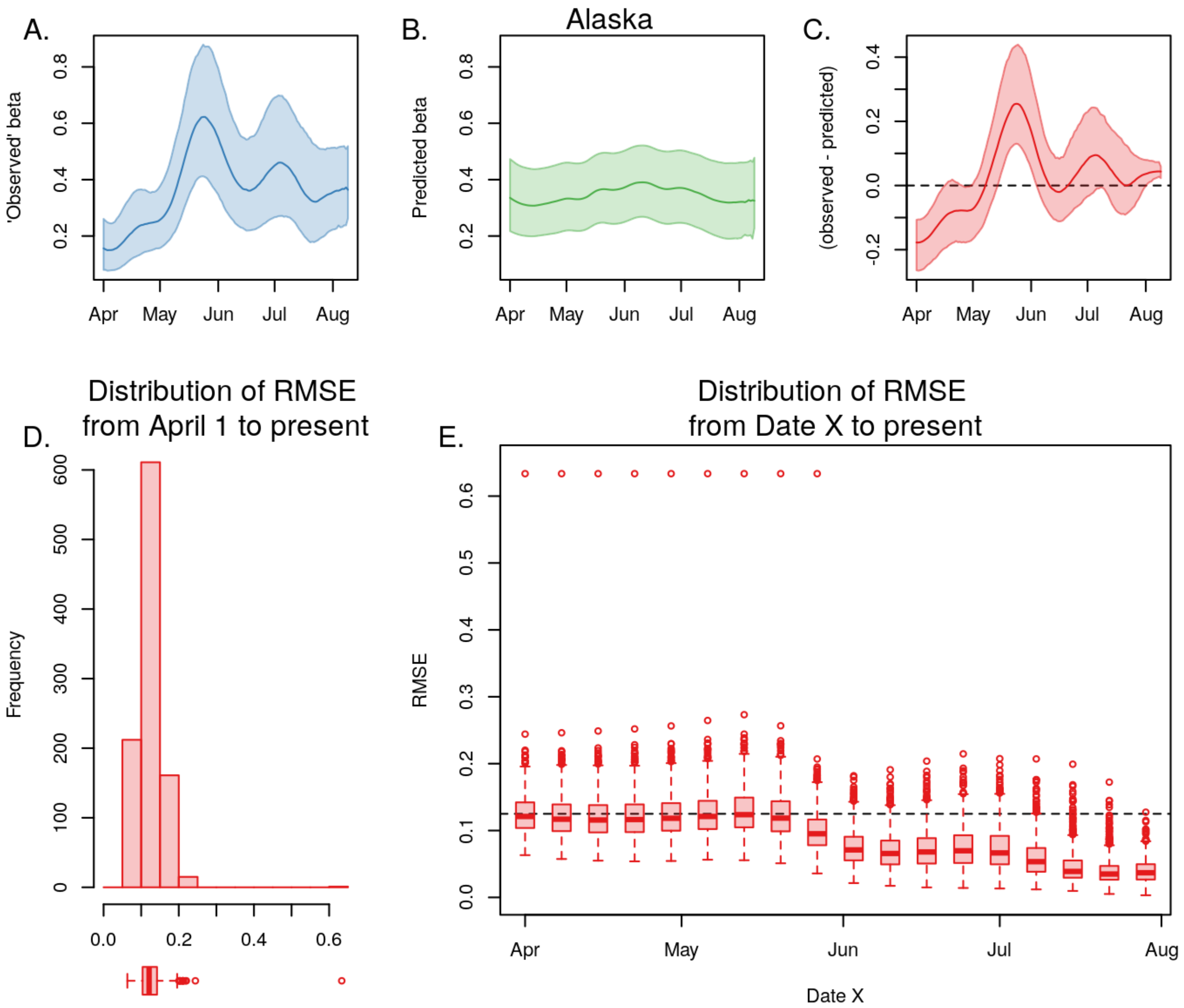

Alaska: Detailed regression diagnostics. **A:** The SEIR *beta* parameter calculated directly from past input data on infections. **B:** The *beta* parameter predicted using a multivariate regression across all locations. **C:** The difference between the directly-calculated and predicted values for *beta*. Mean and uncertainty interval are shown across 1,000 posterior predictive draws over time. **D:** Histogram and box plot showing the distribution of root mean squared error (RMSE) for *beta* when aggregated across all dates from April 1 to present. **E:** Box plots showing the RMSE for aggregates of *beta* from a given date to the present across 1,000 posterior predictive draws.

3 Arizona: Detailed regression diagnostics

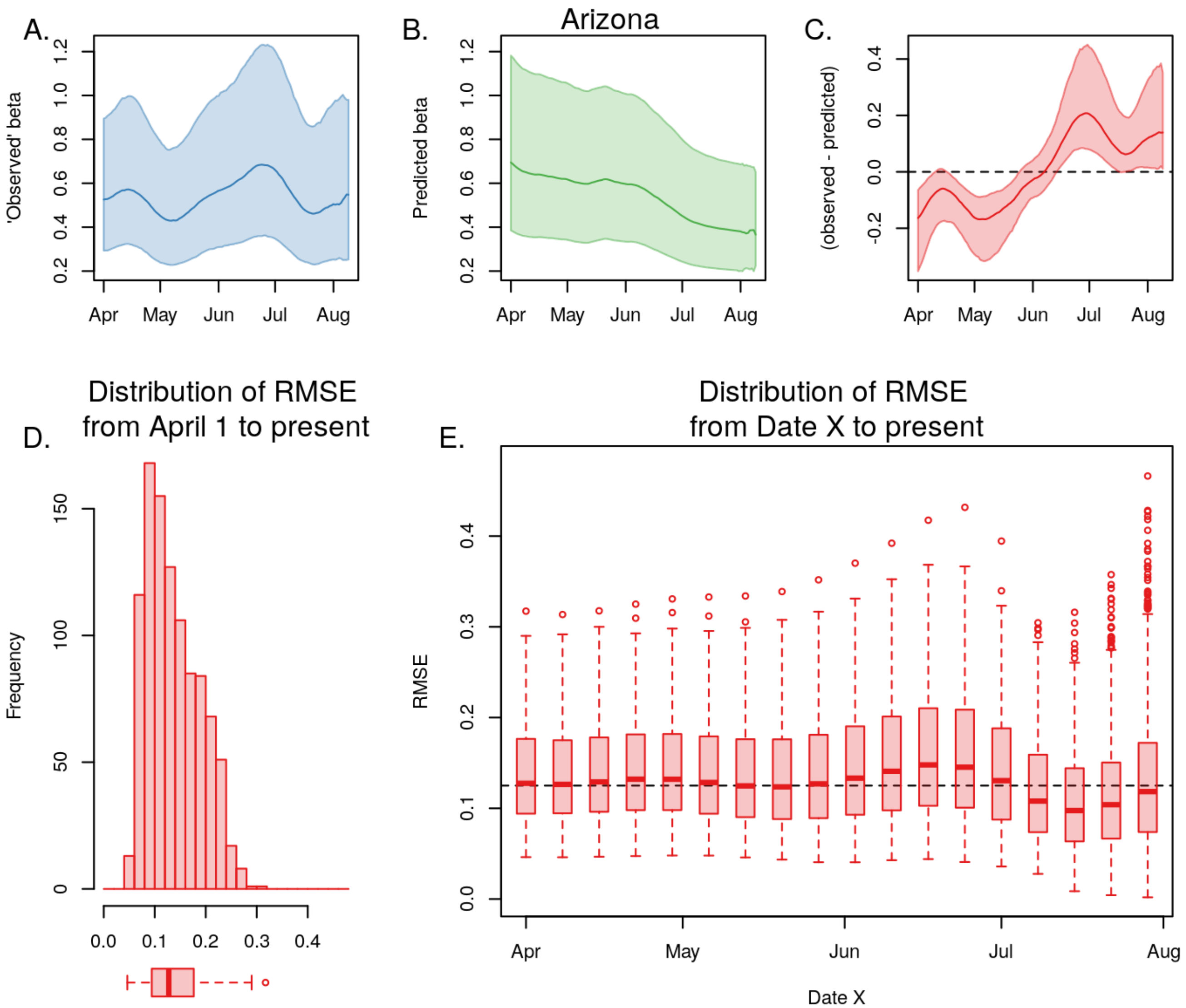

Arizona: Detailed regression diagnostics. **A:** The SEIR *beta* parameter calculated directly from past input data on infections. **B:** The *beta* parameter predicted using a multivariate regression across all locations. **C:** The difference between the directly-calculated and predicted values for *beta*. Mean and uncertainty interval are shown across 1,000 posterior predictive draws over time. **D:** Histogram and box plot showing the distribution of root mean squared error (RMSE) for *beta* when aggregated across all dates from April 1 to present. **E:** Box plots showing the RMSE for aggregates of *beta* from a given date to the present across 1,000 posterior predictive draws.

4 Arkansas: Detailed regression diagnostics

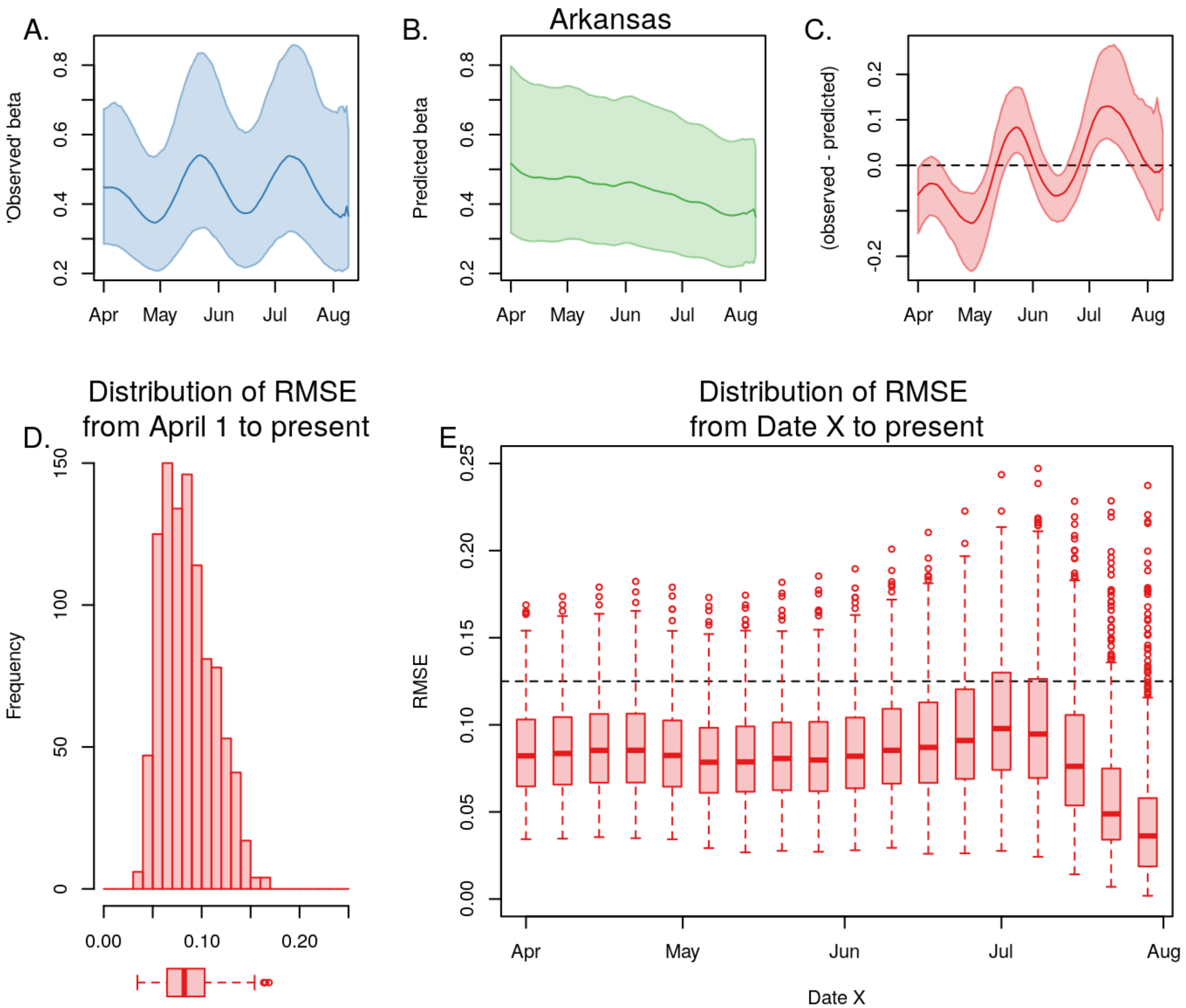

Arkansas: Detailed regression diagnostics. A: The SEIR *beta* parameter calculated directly from past input data on infections. **B:** The *beta* parameter predicted using a multivariate regression across all locations. **C:** The difference between the directly-calculated and predicted values for *beta*. Mean and uncertainty interval are shown across 1,000 posterior predictive draws over time. **D:** Histogram and box plot showing the distribution of root mean squared error (RMSE) for *beta* when aggregated across all dates from April 1 to present. **E:** Box plots showing the RMSE for aggregates of *beta* from a given date to the present across 1,000 posterior predictive draws.

5 California: Detailed regression diagnostics

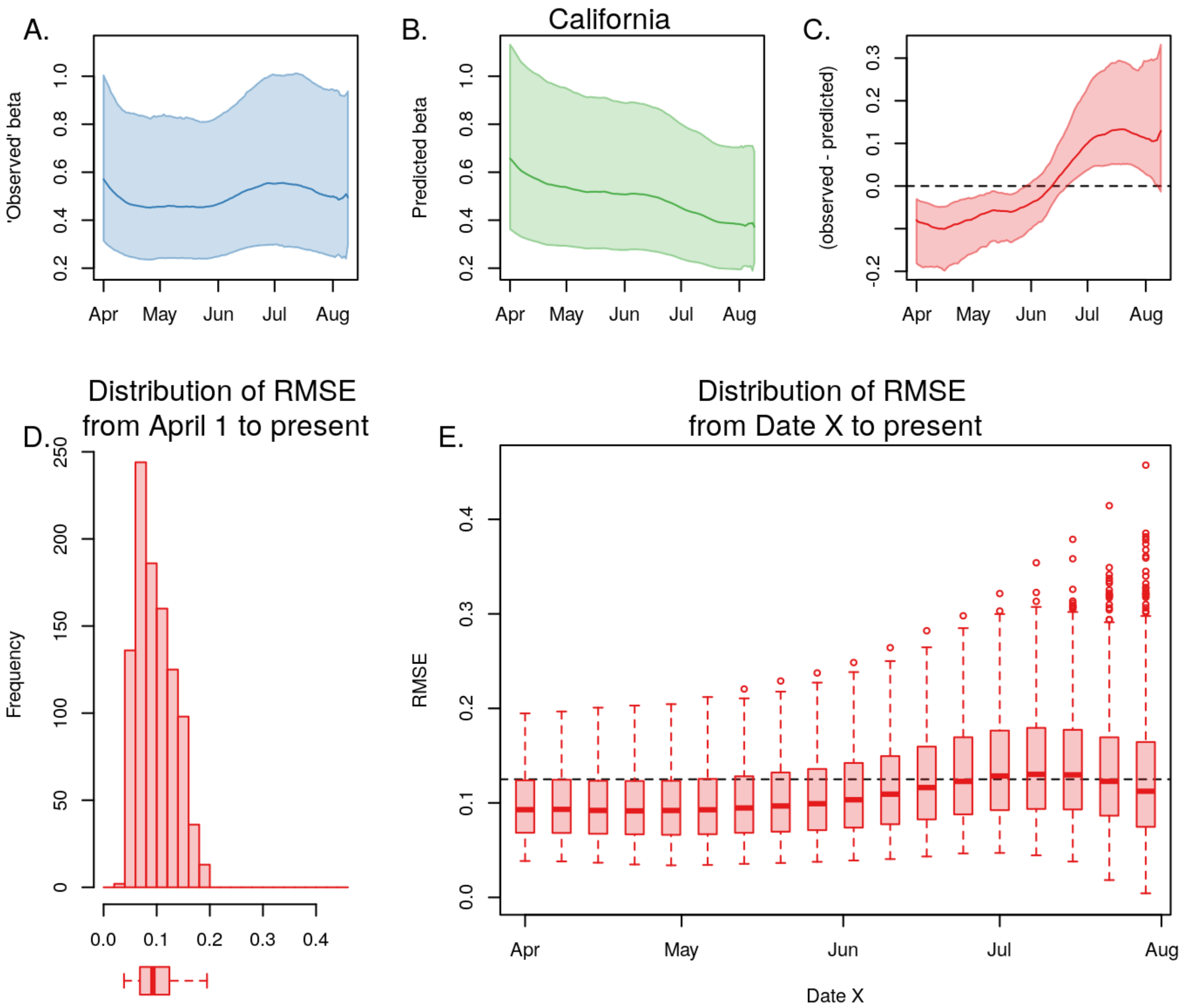

California: Detailed regression diagnostics. **A:** The SEIR *beta* parameter calculated directly from past input data on infections. **B:** The *beta* parameter predicted using a multivariate regression across all locations. **C:** The difference between the directly-calculated and predicted values for *beta*. Mean and uncertainty interval are shown across 1,000 posterior predictive draws over time. **D:** Histogram and box plot showing the distribution of root mean squared error (RMSE) for *beta* when aggregated across all dates from April 1 to present. **E:** Box plots showing the RMSE for aggregates of *beta* from a given date to the present across 1,000 posterior predictive draws.

6 Colorado: Detailed regression diagnostics

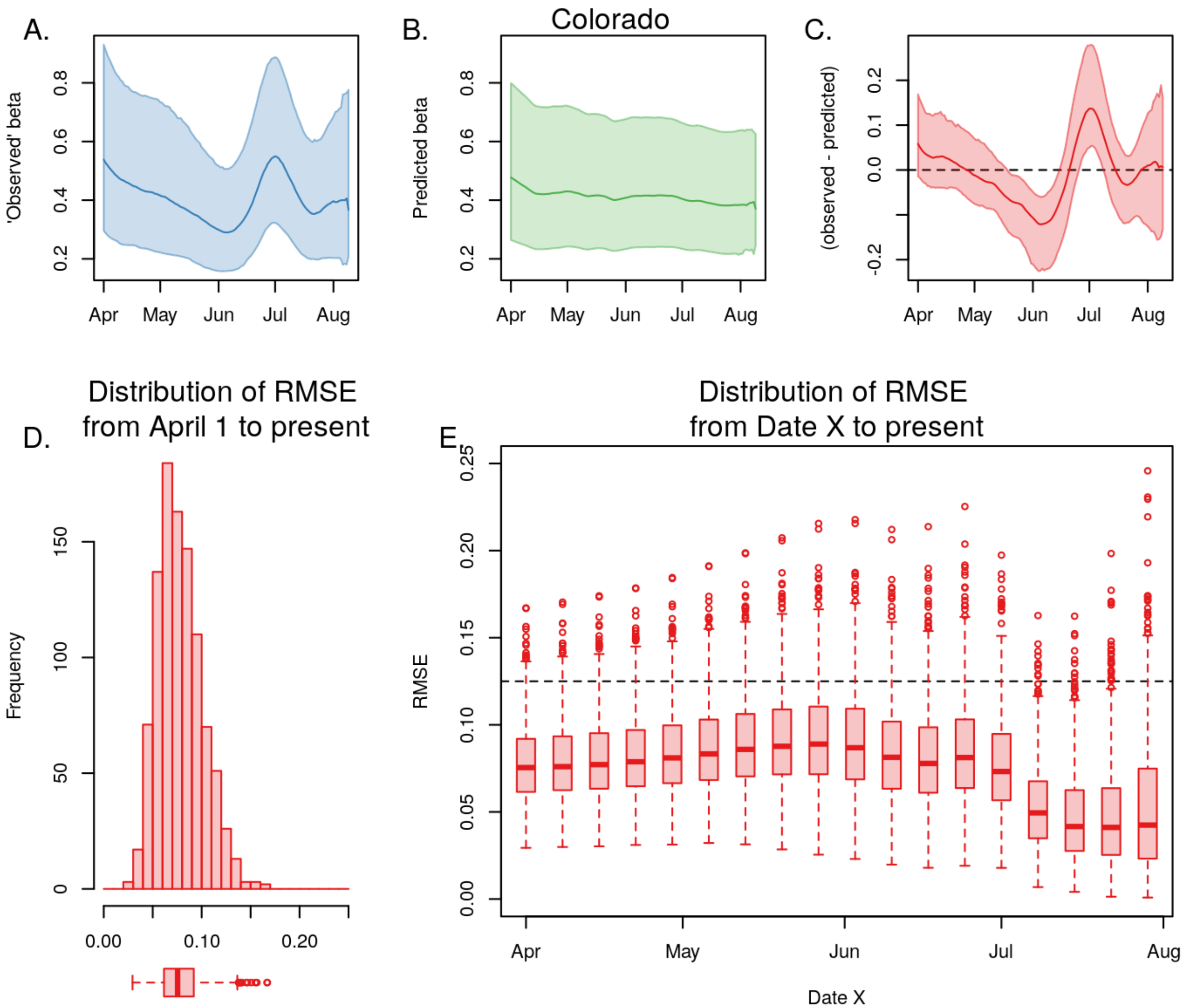

Colorado: Detailed regression diagnostics. A: The SEIR *beta* parameter calculated directly from past input data on infections. **B:** The *beta* parameter predicted using a multivariate regression across all locations. **C:** The difference between the directly-calculated and predicted values for *beta*. Mean and uncertainty interval are shown across 1,000 posterior predictive draws over time. **D:** Histogram and box plot showing the distribution of root mean squared error (RMSE) for *beta* when aggregated across all dates from April 1 to present. **E:** Box plots showing the RMSE for aggregates of *beta* from a given date to the present across 1,000 posterior predictive draws.

7 Connecticut: Detailed regression diagnostics

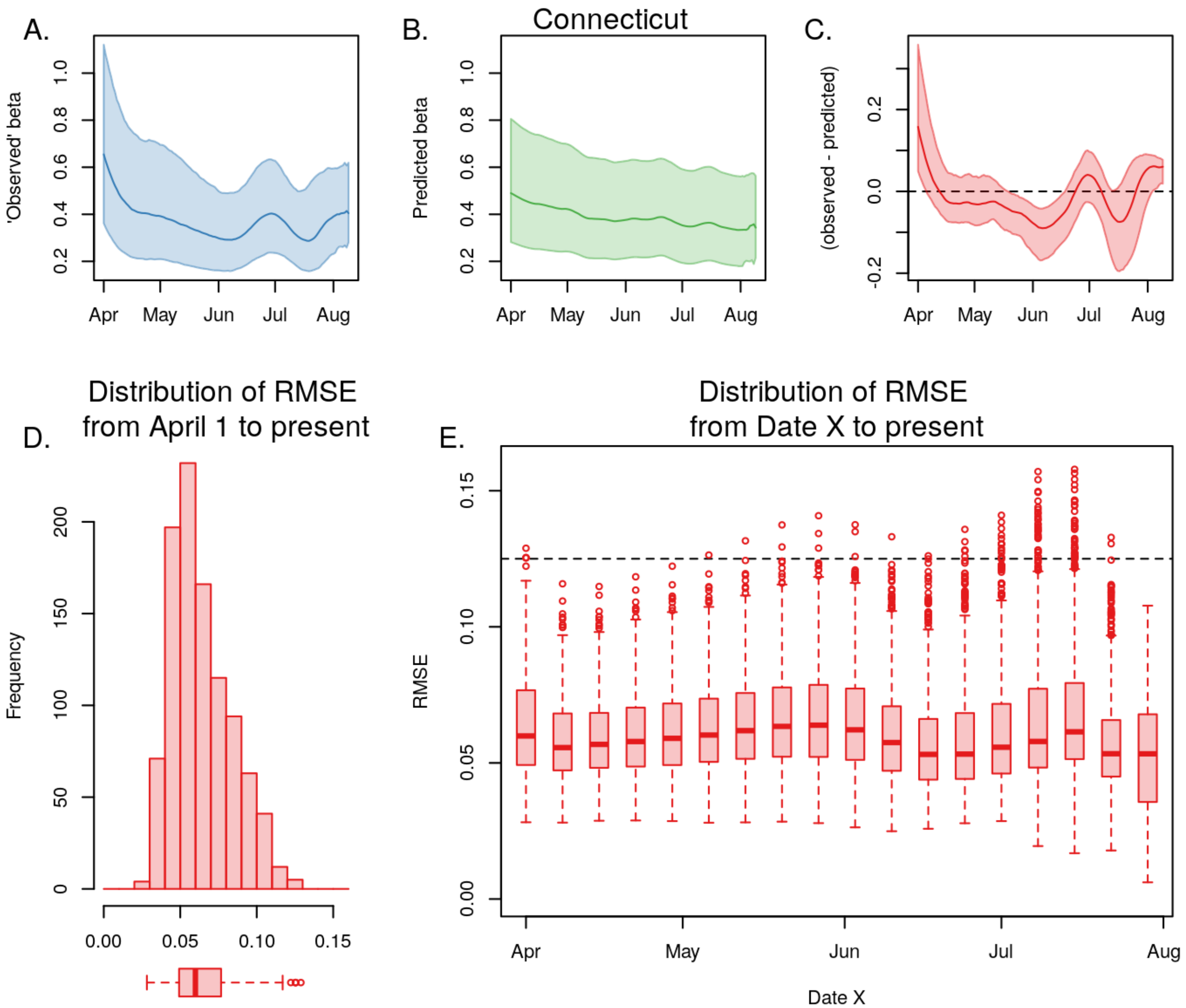

Connecticut: Detailed regression diagnostics. A: The SEIR *beta* parameter calculated directly from past input data on infections. **B:** The *beta* parameter predicted using a multivariate regression across all locations. **C:** The difference between the directly-calculated and predicted values for *beta*. Mean and uncertainty interval are shown across 1,000 posterior predictive draws over time. **D:** Histogram and box plot showing the distribution of root mean squared error (RMSE) for *beta* when aggregated across all dates from April 1 to present. **E:** Box plots showing the RMSE for aggregates of *beta* from a given date to the present across 1,000 posterior predictive draws.

8 Delaware: Detailed regression diagnostics

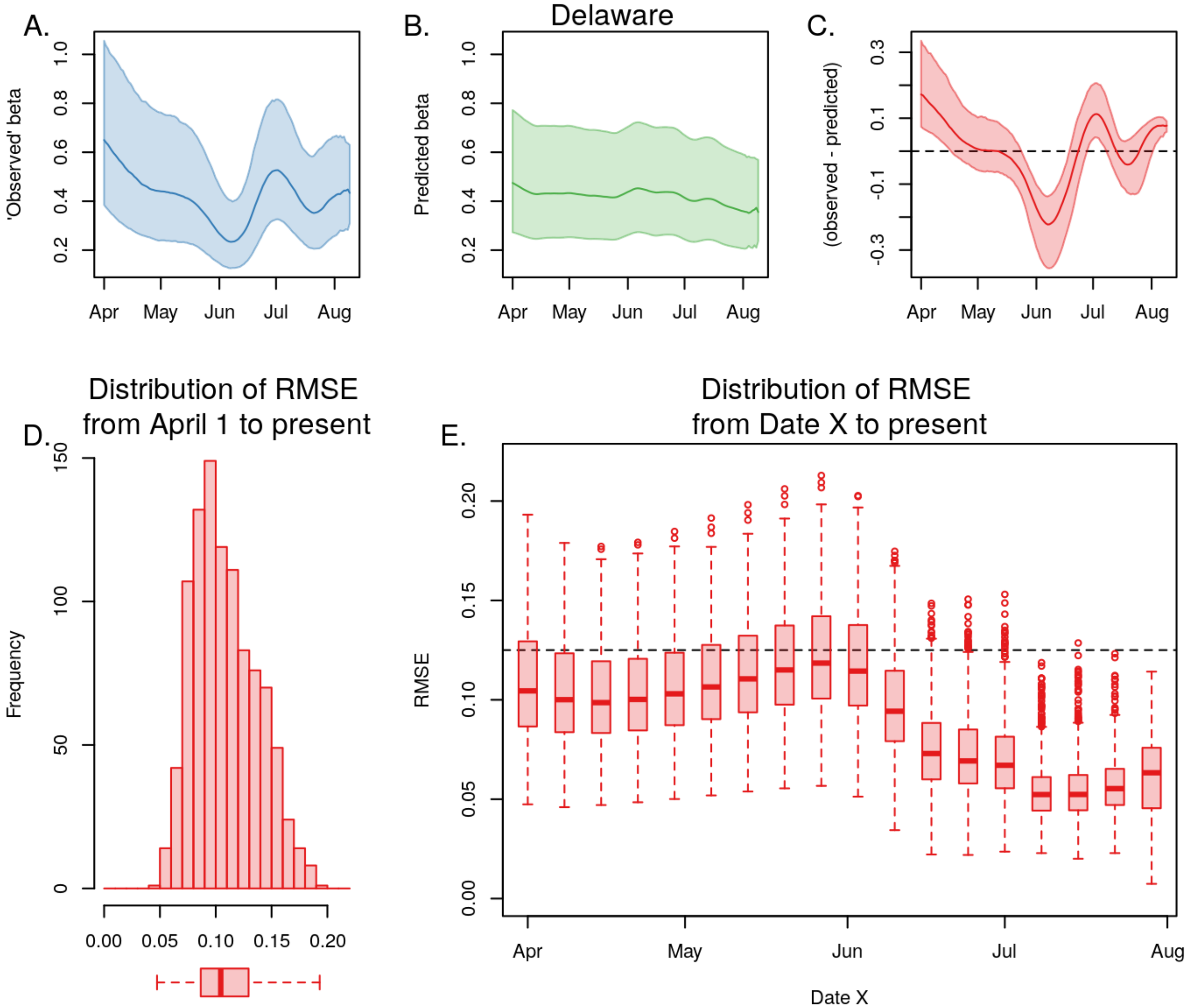

Delaware: Detailed regression diagnostics. A: The SEIR *beta* parameter calculated directly from past input data on infections. **B:** The *beta* parameter predicted using a multivariate regression across all locations. **C:** The difference between the directly-calculated and predicted values for *beta*. Mean and uncertainty interval are shown across 1,000 posterior predictive draws over time. **D:** Histogram and box plot showing the distribution of root mean squared error (RMSE) for *beta* when aggregated across all dates from April 1 to present. **E:** Box plots showing the RMSE for aggregates of *beta* from a given date to the present across 1,000 posterior predictive draws.

9 District of Columbia: Detailed regression diagnostics

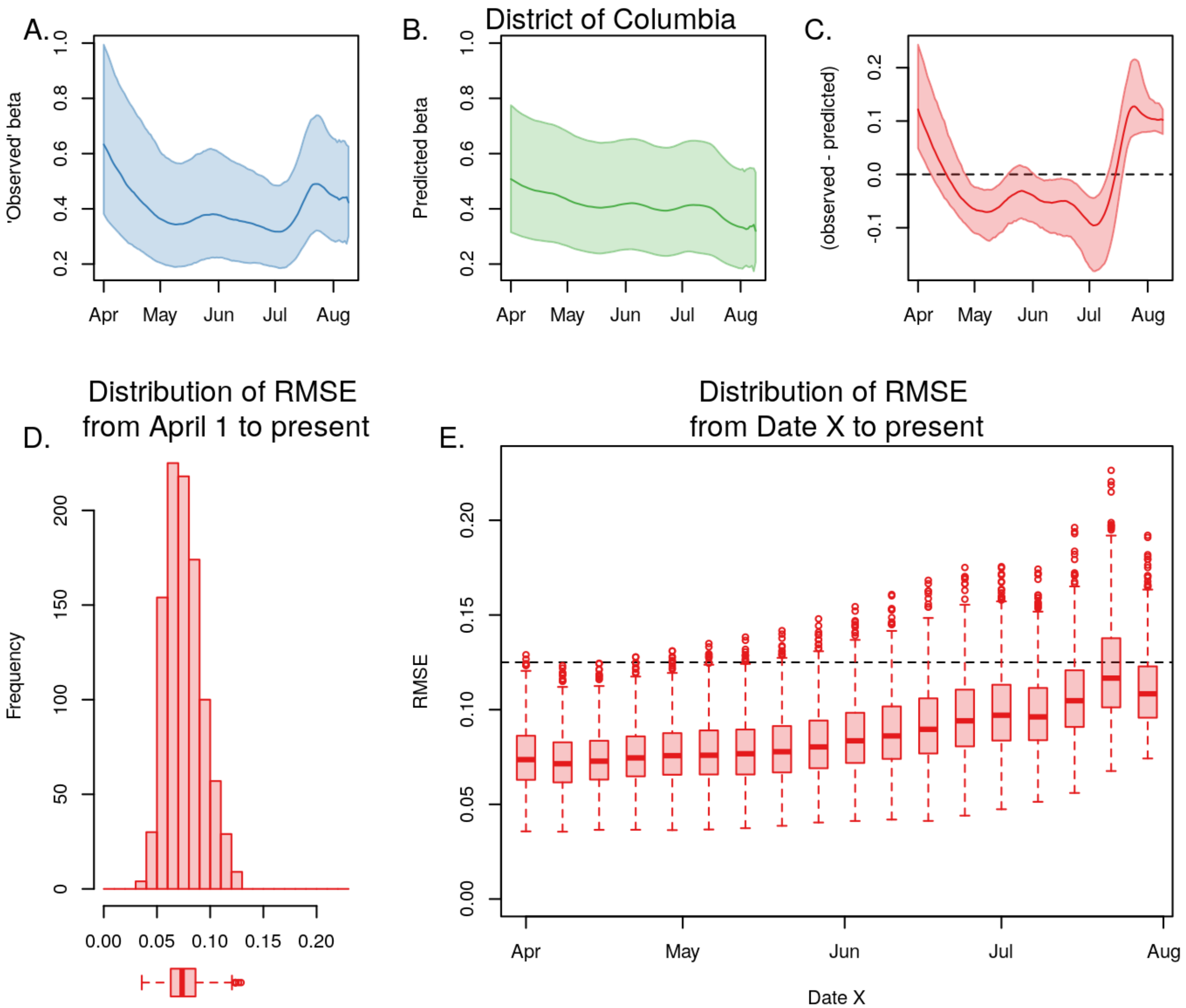

District of Columbia: Detailed regression diagnostics. A: The SEIR *beta* parameter calculated directly from past input data on infections. **B:** The *beta* parameter predicted using a multivariate regression across all locations. **C:** The difference between the directly-calculated and predicted values for *beta*. Mean and uncertainty interval are shown across 1,000 posterior predictive draws over time. **D:** Histogram and box plot showing the distribution of root mean squared error (RMSE) for *beta* when aggregated across all dates from April 1 to present. **E:** Box plots showing the RMSE for aggregates of *beta* from a given date to the present across 1,000 posterior predictive draws.

10 Florida: Detailed regression diagnostics

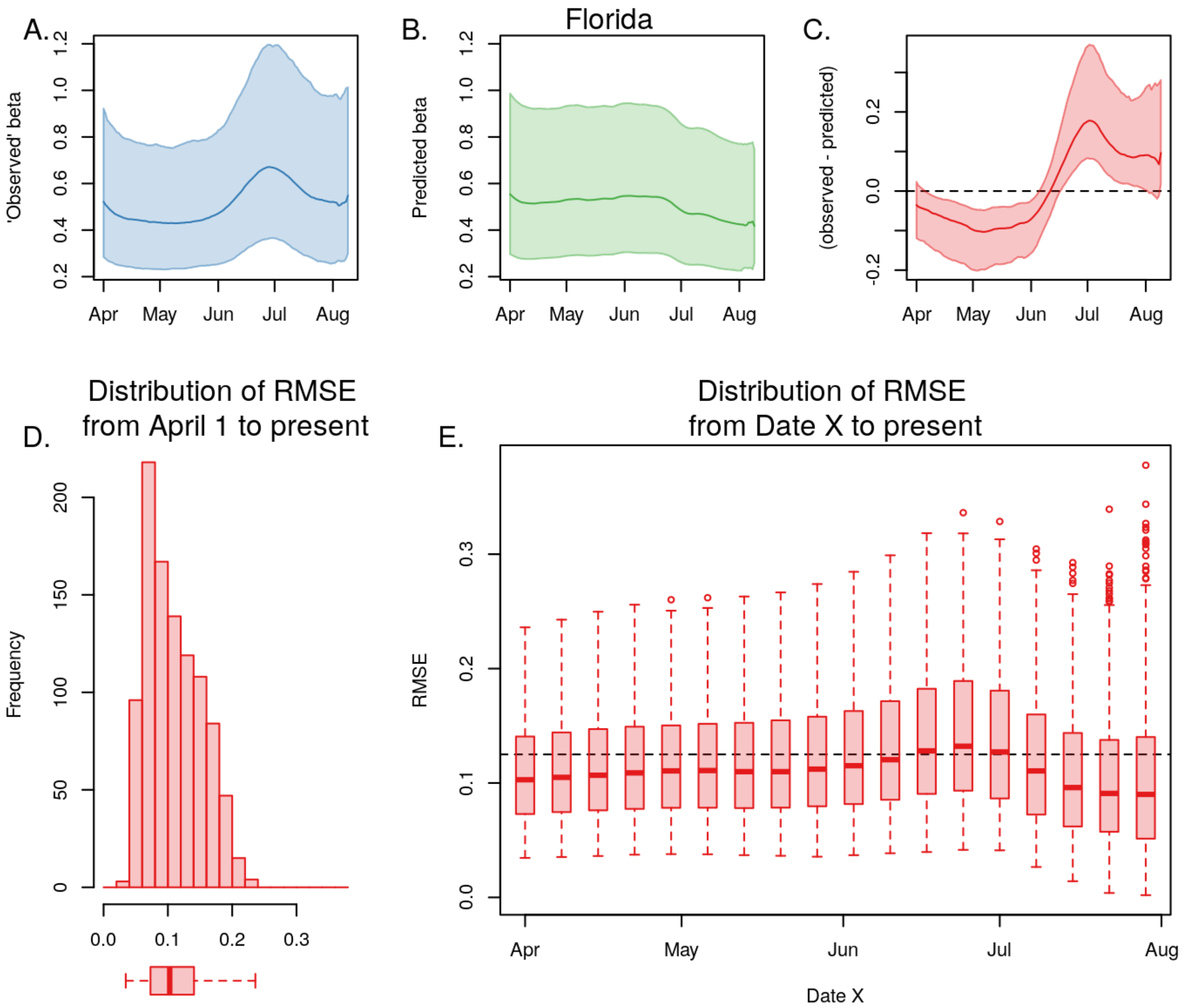

Florida: Detailed regression diagnostics. A: The SEIR *beta* parameter calculated directly from past input data on infections. **B:** The *beta* parameter predicted using a multivariate regression across all locations. **C:** The difference between the directly-calculated and predicted values for *beta*. Mean and uncertainty interval are shown across 1,000 posterior predictive draws over time. **D:** Histogram and box plot showing the distribution of root mean squared error (RMSE) for *beta* when aggregated across all dates from April 1 to present. **E:** Box plots showing the RMSE for aggregates of *beta* from a given date to the present across 1,000 posterior predictive draws.

11 Georgia: Detailed regression diagnostics

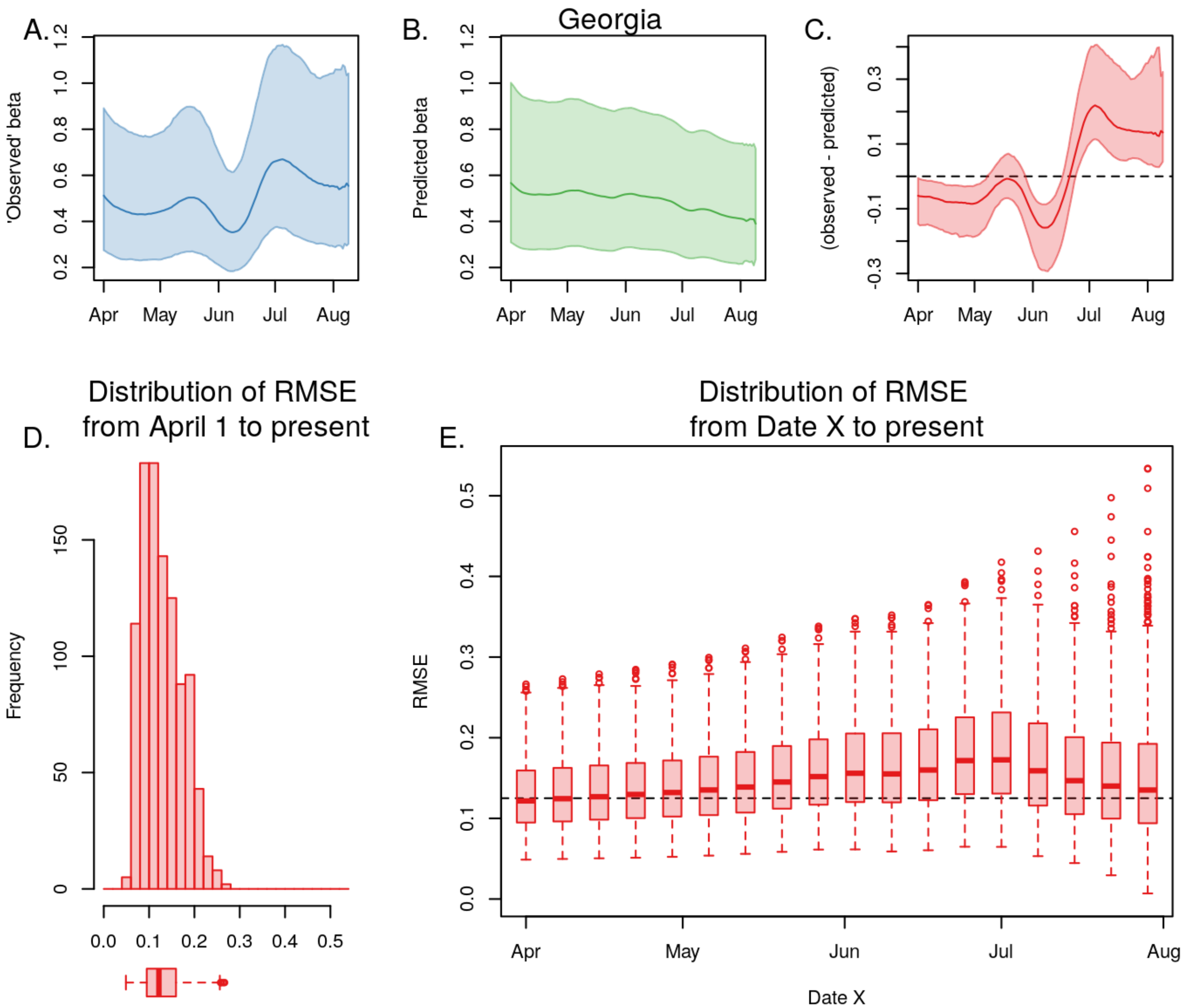

Georgia: Detailed regression diagnostics. A: The SEIR *beta* parameter calculated directly from past input data on infections. **B:** The *beta* parameter predicted using a multivariate regression across all locations. **C:** The difference between the directly-calculated and predicted values for *beta*. Mean and uncertainty interval are shown across 1,000 posterior predictive draws over time. **D:** Histogram and box plot showing the distribution of root mean squared error (RMSE) for *beta* when aggregated across all dates from April 1 to present. **E:** Box plots showing the RMSE for aggregates of *beta* from a given date to the present across 1,000 posterior predictive draws.

12 Hawaii: Detailed regression diagnostics

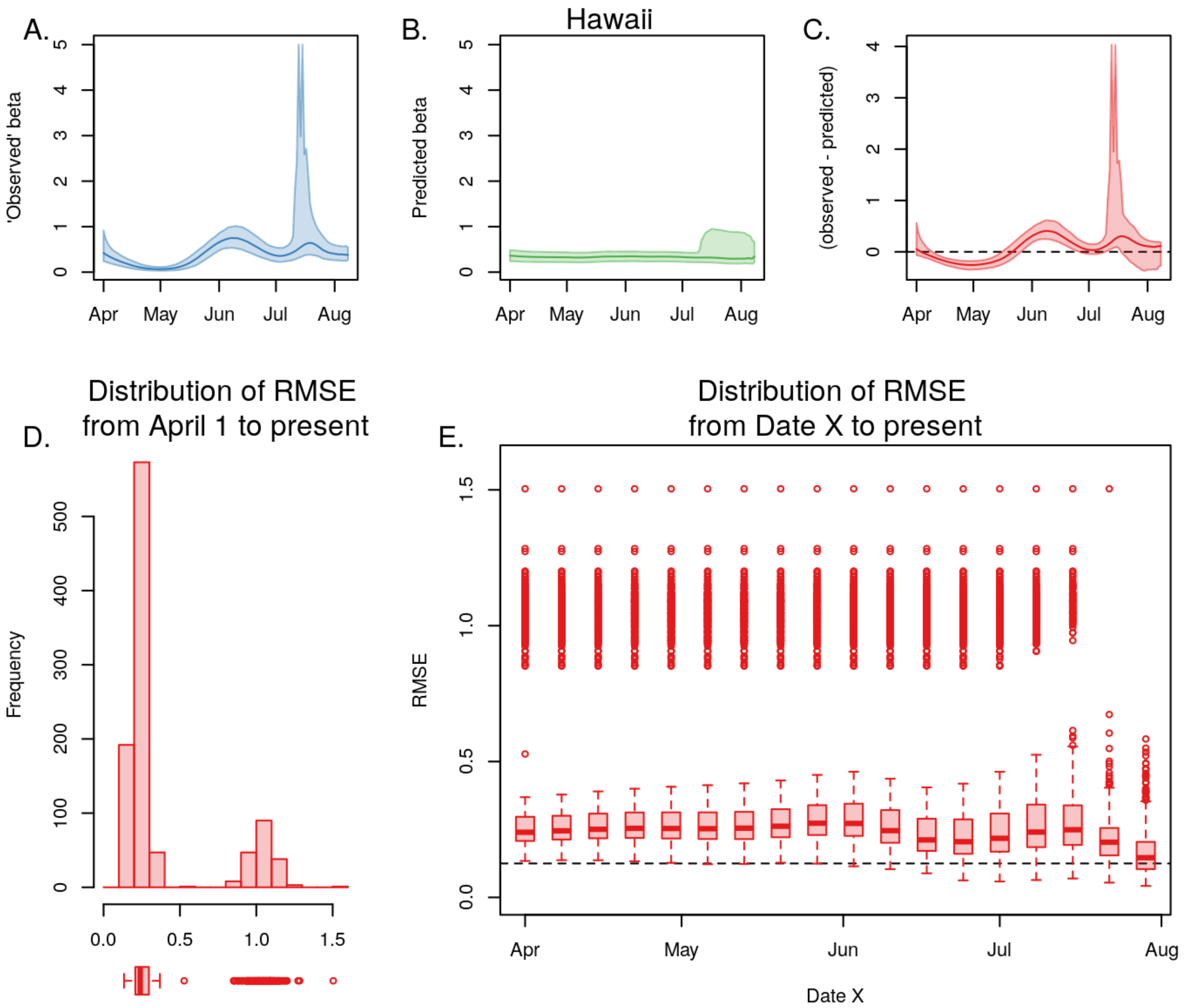

Hawaii: Detailed regression diagnostics. A: The SEIR *beta* parameter calculated directly from past input data on infections. **B:** The *beta* parameter predicted using a multivariate regression across all locations. **C:** The difference between the directly-calculated and predicted values for *beta*. Mean and uncertainty interval are shown across 1,000 posterior predictive draws over time. **D:** Histogram and box plot showing the distribution of root mean squared error (RMSE) for *beta* when aggregated across all dates from April 1 to present. **E:** Box plots showing the RMSE for aggregates of *beta* from a given date to the present across 1,000 posterior predictive draws.

13 Idaho: Detailed regression diagnostics

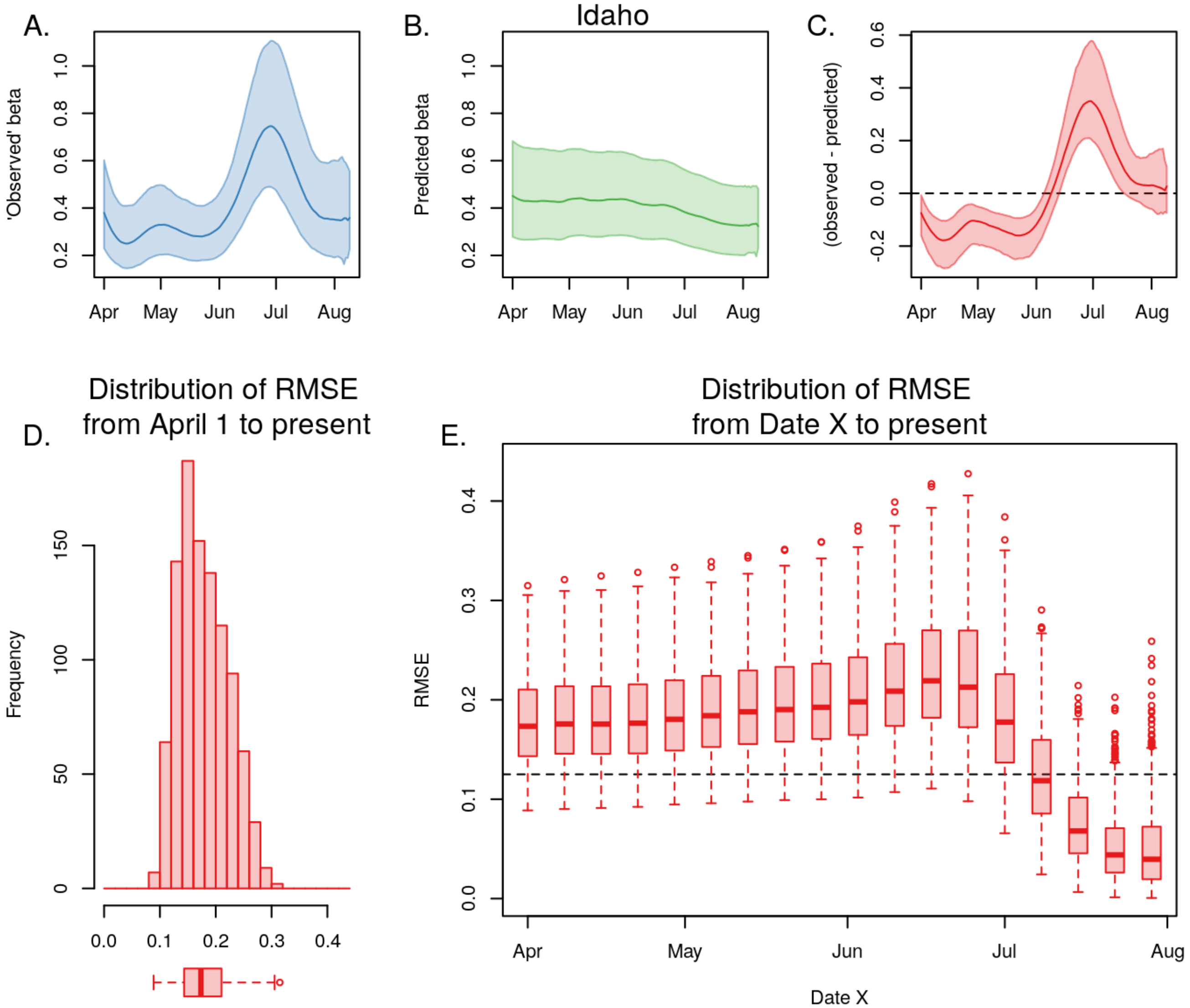

Idaho: Detailed regression diagnostics. **A:** The SEIR *beta* parameter calculated directly from past input data on infections. **B:** The *beta* parameter predicted using a multivariate regression across all locations. **C:** The difference between the directly-calculated and predicted values for *beta*. Mean and uncertainty interval are shown across 1,000 posterior predictive draws over time. **D:** Histogram and box plot showing the distribution of root mean squared error (RMSE) for *beta* when aggregated across all dates from April 1 to present. **E:** Box plots showing the RMSE for aggregates of *beta* from a given date to the present across 1,000 posterior predictive draws.

14 Illinois: Detailed regression diagnostics

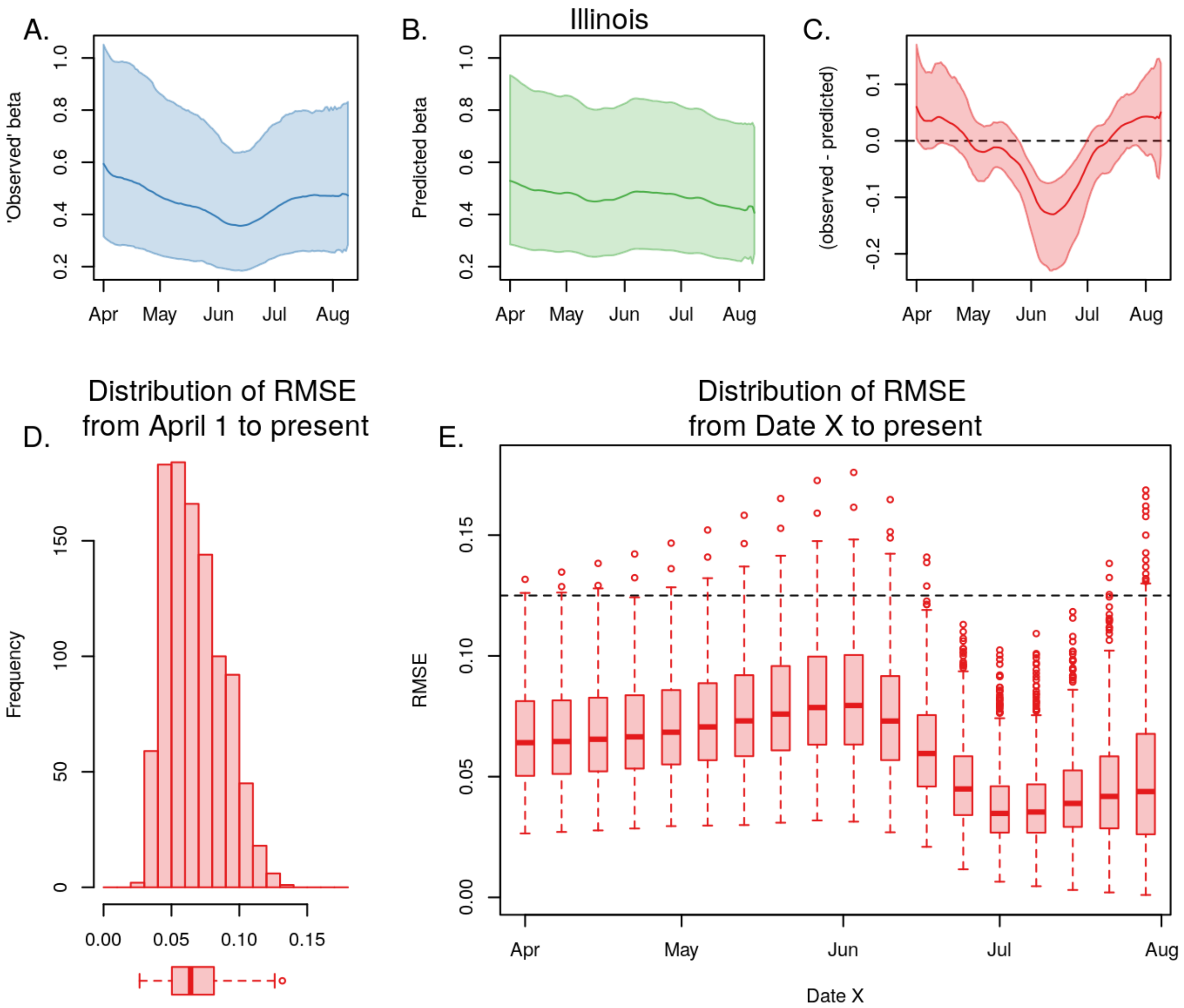

Illinois: Detailed regression diagnostics. **A:** The SEIR *beta* parameter calculated directly from past input data on infections. **B:** The *beta* parameter predicted using a multivariate regression across all locations. **C:** The difference between the directly-calculated and predicted values for *beta*. Mean and uncertainty interval are shown across 1,000 posterior predictive draws over time. **D:** Histogram and box plot showing the distribution of root mean squared error (RMSE) for *beta* when aggregated across all dates from April 1 to present. **E:** Box plots showing the RMSE for aggregates of *beta* from a given date to the present across 1,000 posterior predictive draws.

15 Indiana: Detailed regression diagnostics

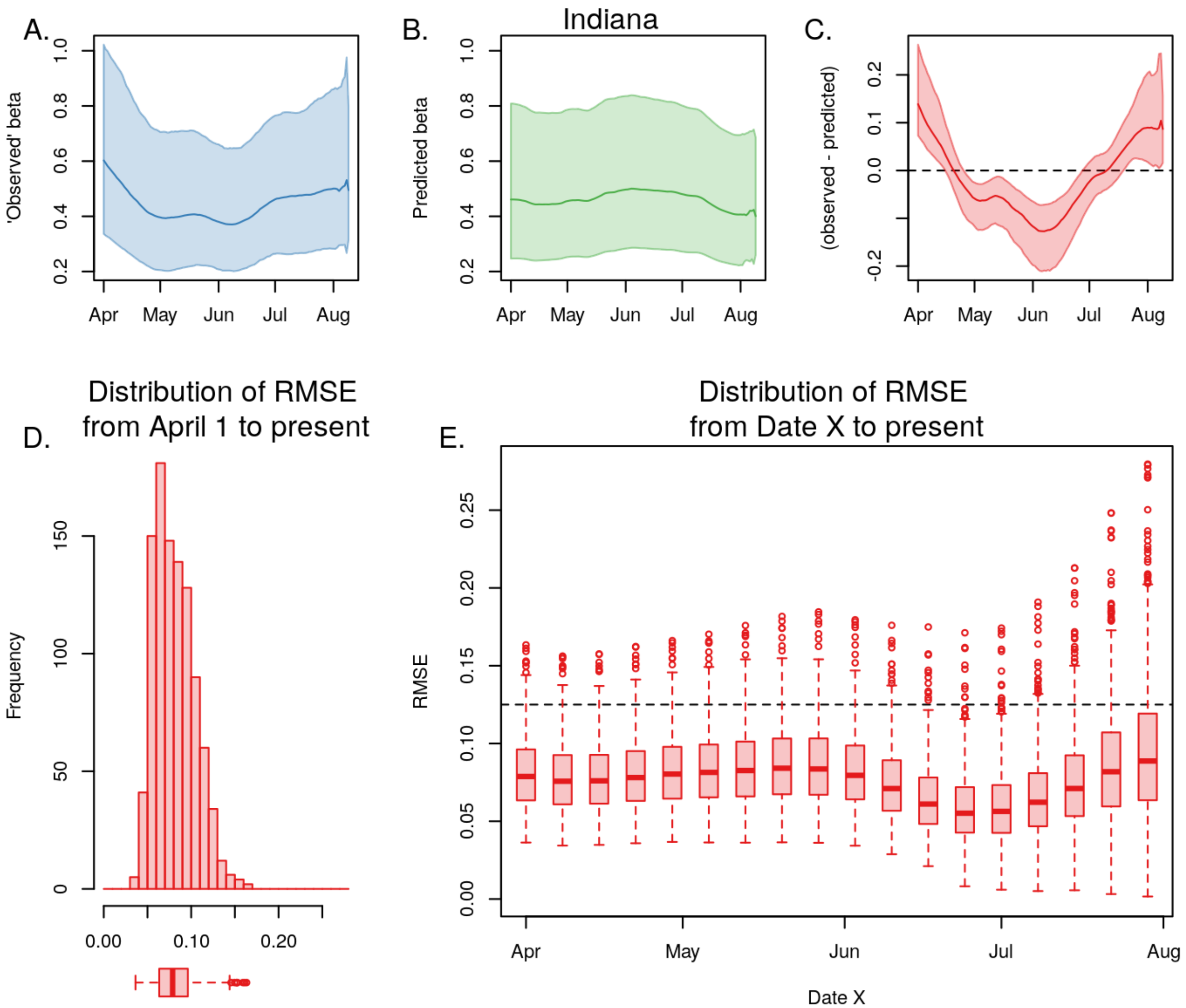

Indiana: Detailed regression diagnostics. A: The SEIR *beta* parameter calculated directly from past input data on infections. **B:** The *beta* parameter predicted using a multivariate regression across all locations. **C:** The difference between the directly-calculated and predicted values for *beta*. Mean and uncertainty interval are shown across 1,000 posterior predictive draws over time. **D:** Histogram and box plot showing the distribution of root mean squared error (RMSE) for *beta* when aggregated across all dates from April 1 to present. **E:** Box plots showing the RMSE for aggregates of *beta* from a given date to the present across 1,000 posterior predictive draws.

16 Iowa: Detailed regression diagnostics

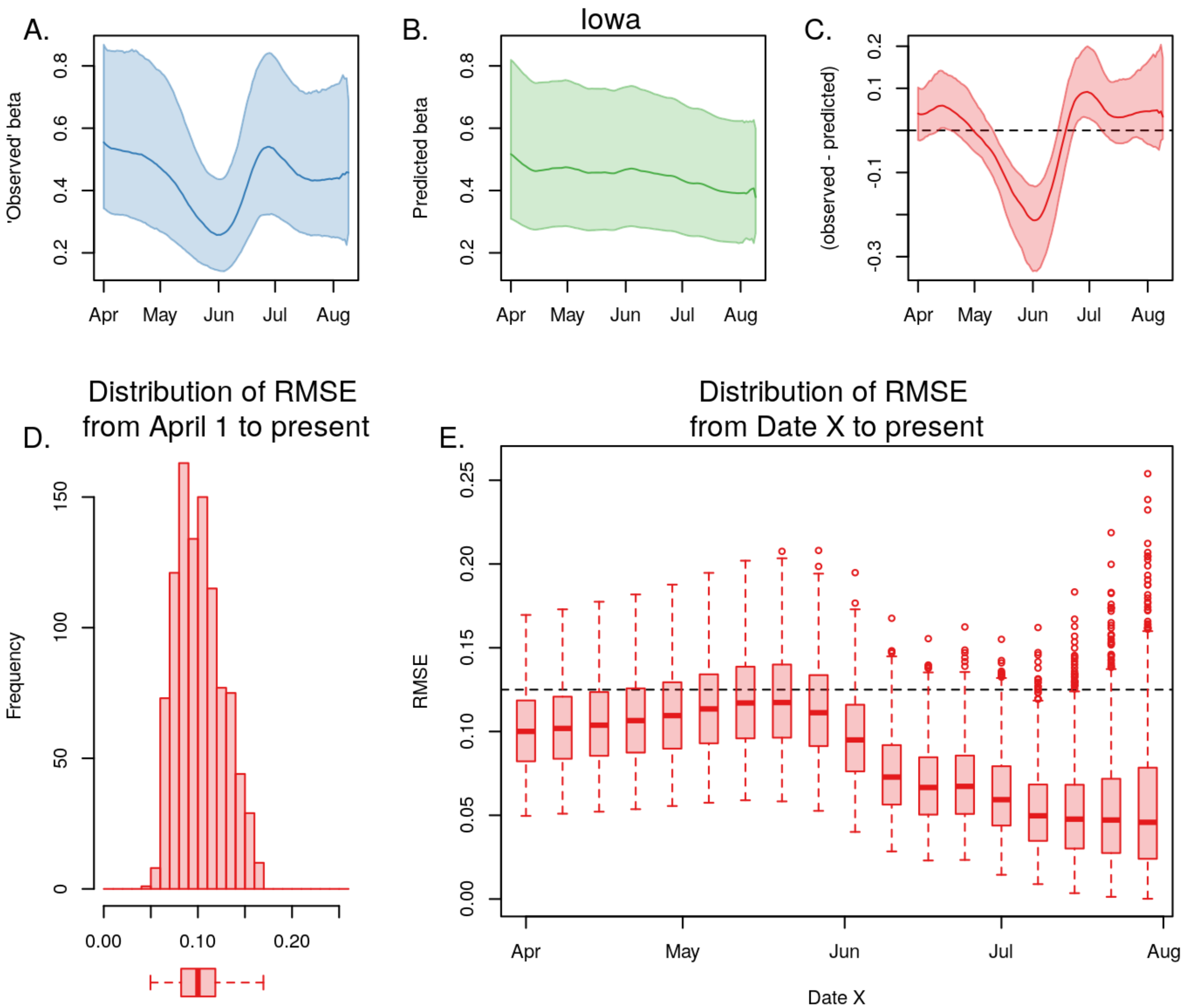

Iowa: Detailed regression diagnostics. **A:** The SEIR *beta* parameter calculated directly from past input data on infections. **B:** The *beta* parameter predicted using a multivariate regression across all locations. **C:** The difference between the directly-calculated and predicted values for *beta*. Mean and uncertainty interval are shown across 1,000 posterior predictive draws over time. **D:** Histogram and box plot showing the distribution of root mean squared error (RMSE) for *beta* when aggregated across all dates from April 1 to present. **E:** Box plots showing the RMSE for aggregates of *beta* from a given date to the present across 1,000 posterior predictive draws.

17 Kansas: Detailed regression diagnostics

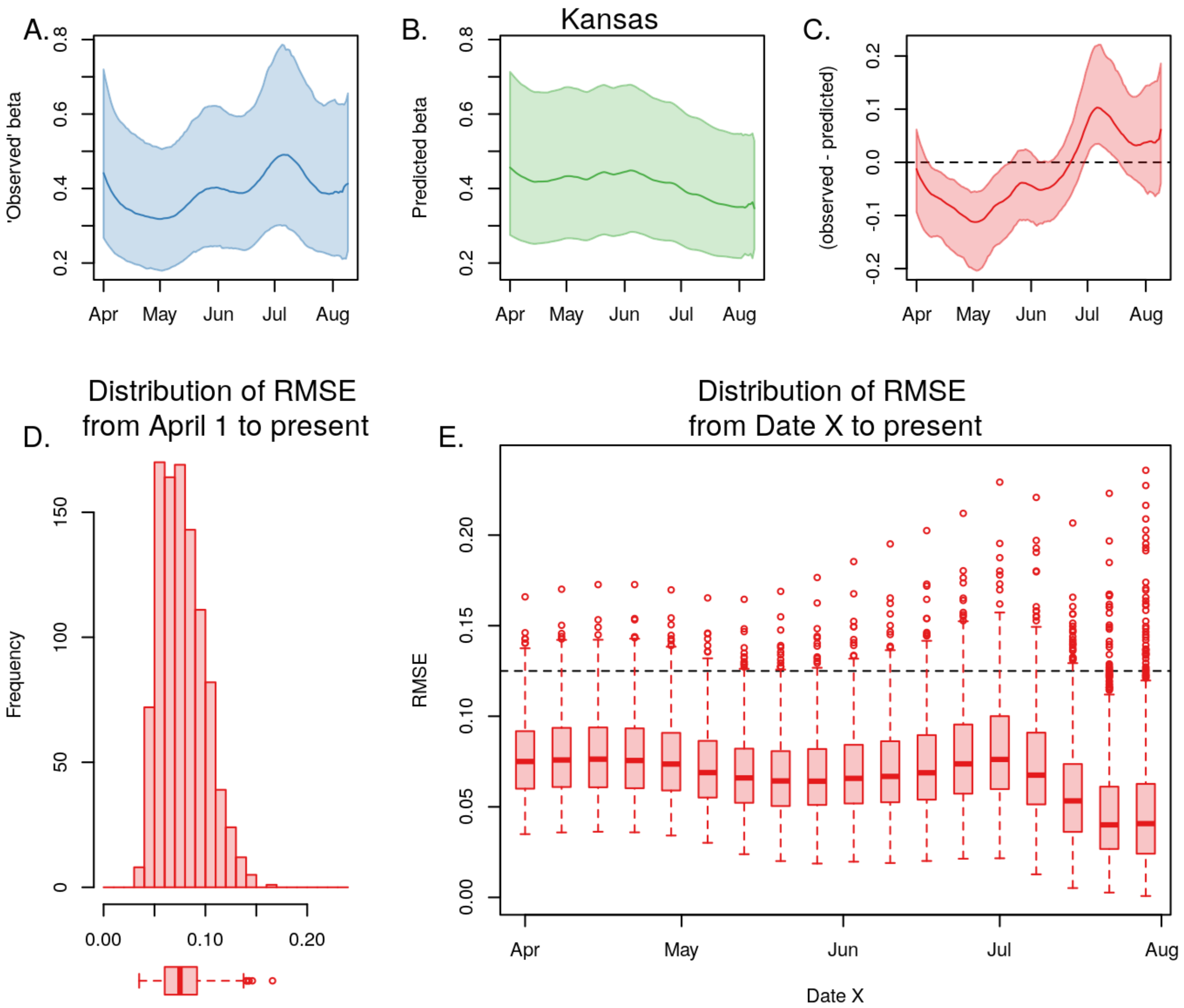

Kansas: Detailed regression diagnostics. **A:** The SEIR *beta* parameter calculated directly from past input data on infections. **B:** The *beta* parameter predicted using a multivariate regression across all locations. **C:** The difference between the directly-calculated and predicted values for *beta*. Mean and uncertainty interval are shown across 1,000 posterior predictive draws over time. **D:** Histogram and box plot showing the distribution of root mean squared error (RMSE) for *beta* when aggregated across all dates from April 1 to present. **E:** Box plots showing the RMSE for aggregates of *beta* from a given date to the present across 1,000 posterior predictive draws.

18 Kentucky: Detailed regression diagnostics

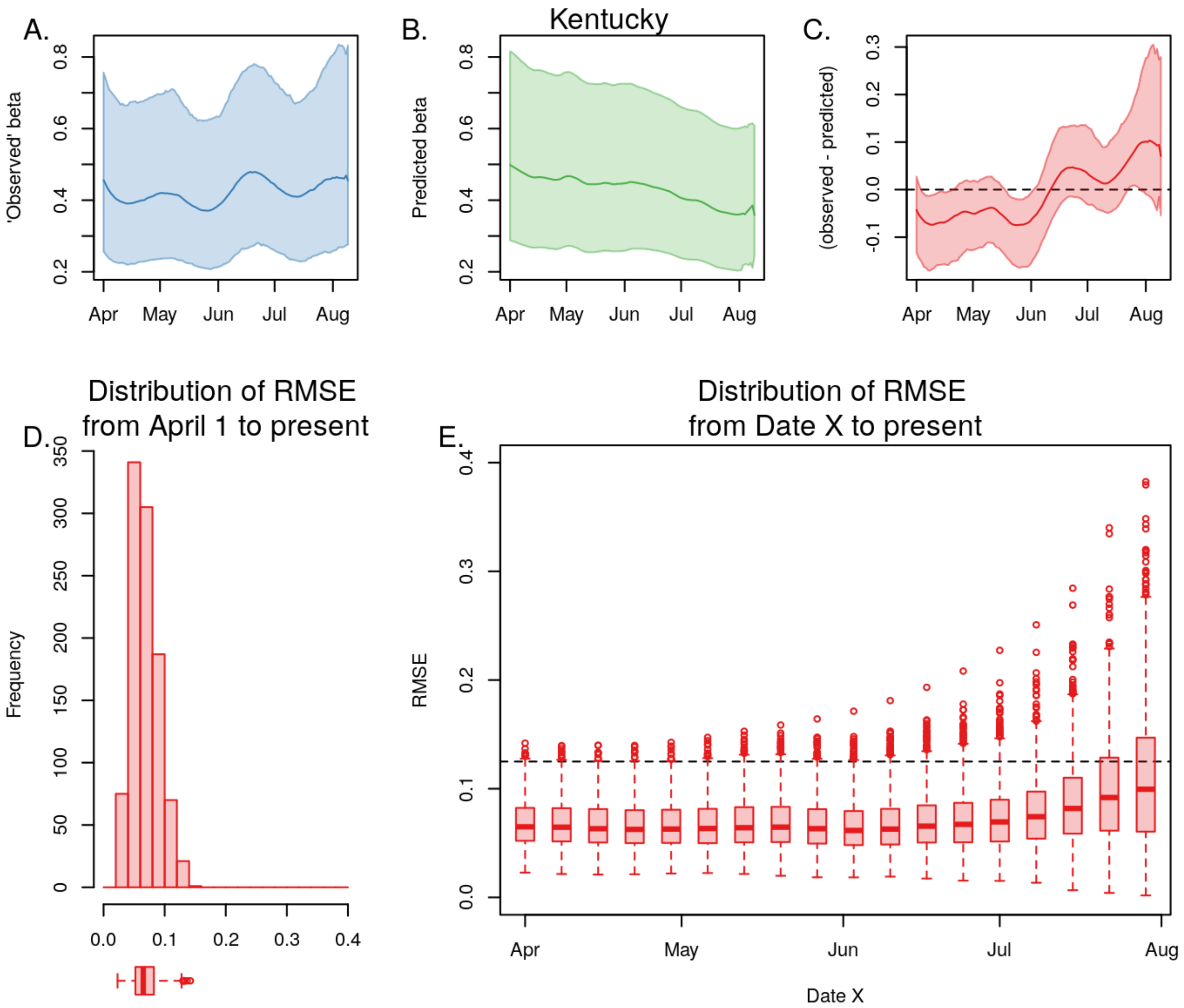

Kentucky: Detailed regression diagnostics. A: The SEIR *beta* parameter calculated directly from past input data on infections. **B:** The *beta* parameter predicted using a multivariate regression across all locations. **C:** The difference between the directly-calculated and predicted values for *beta*. Mean and uncertainty interval are shown across 1,000 posterior predictive draws over time. **D:** Histogram and box plot showing the distribution of root mean squared error (RMSE) for *beta* when aggregated across all dates from April 1 to present. **E:** Box plots showing the RMSE for aggregates of *beta* from a given date to the present across 1,000 posterior predictive draws.

19 Louisiana: Detailed regression diagnostics

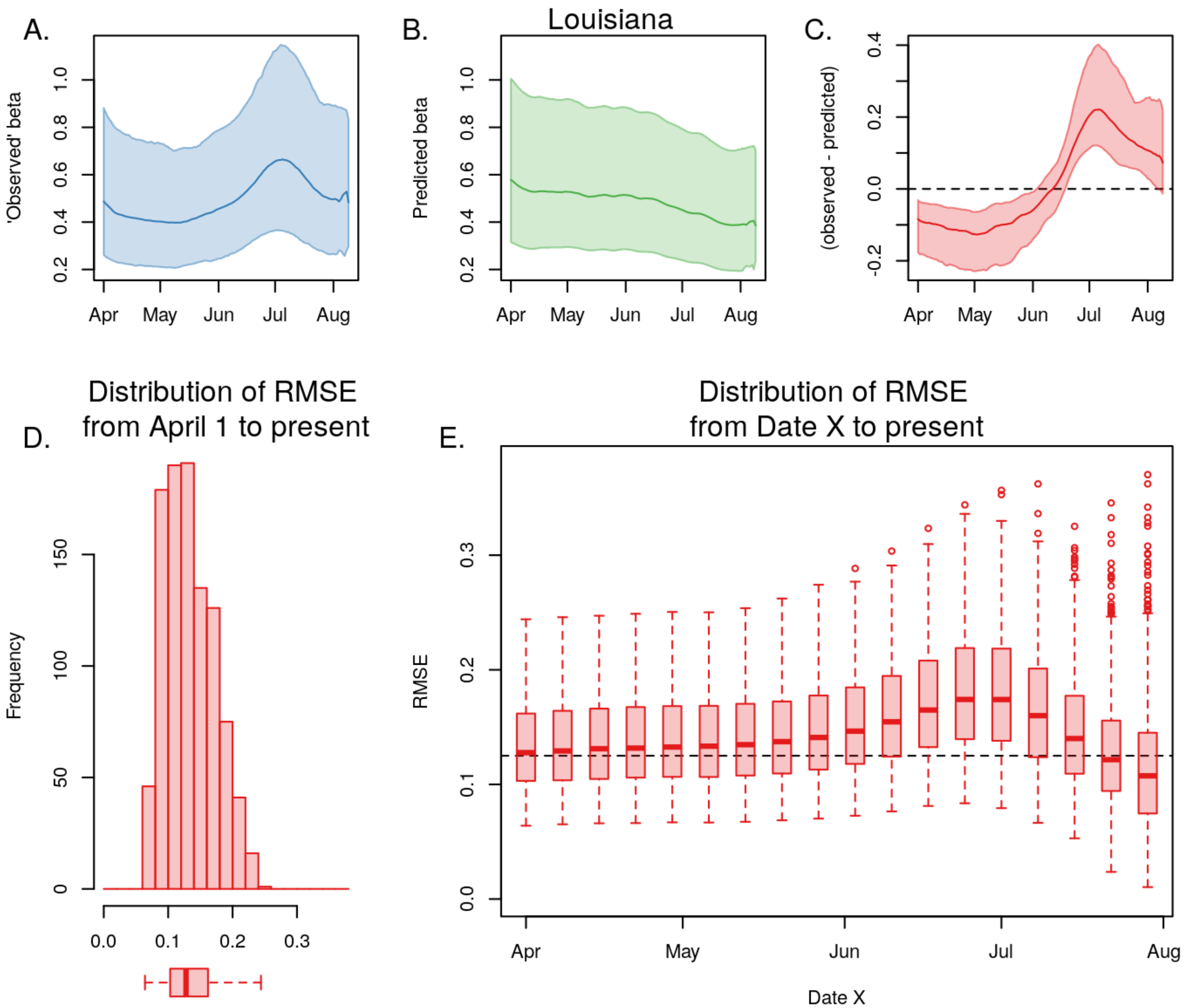

Louisiana: Detailed regression diagnostics. A: The SEIR *beta* parameter calculated directly from past input data on infections. **B:** The *beta* parameter predicted using a multivariate regression across all locations. **C:** The difference between the directly-calculated and predicted values for *beta*. Mean and uncertainty interval are shown across 1,000 posterior predictive draws over time. **D:** Histogram and box plot showing the distribution of root mean squared error (RMSE) for *beta* when aggregated across all dates from April 1 to present. **E:** Box plots showing the RMSE for aggregates of *beta* from a given date to the present across 1,000 posterior predictive draws.

20 Maine: Detailed regression diagnostics

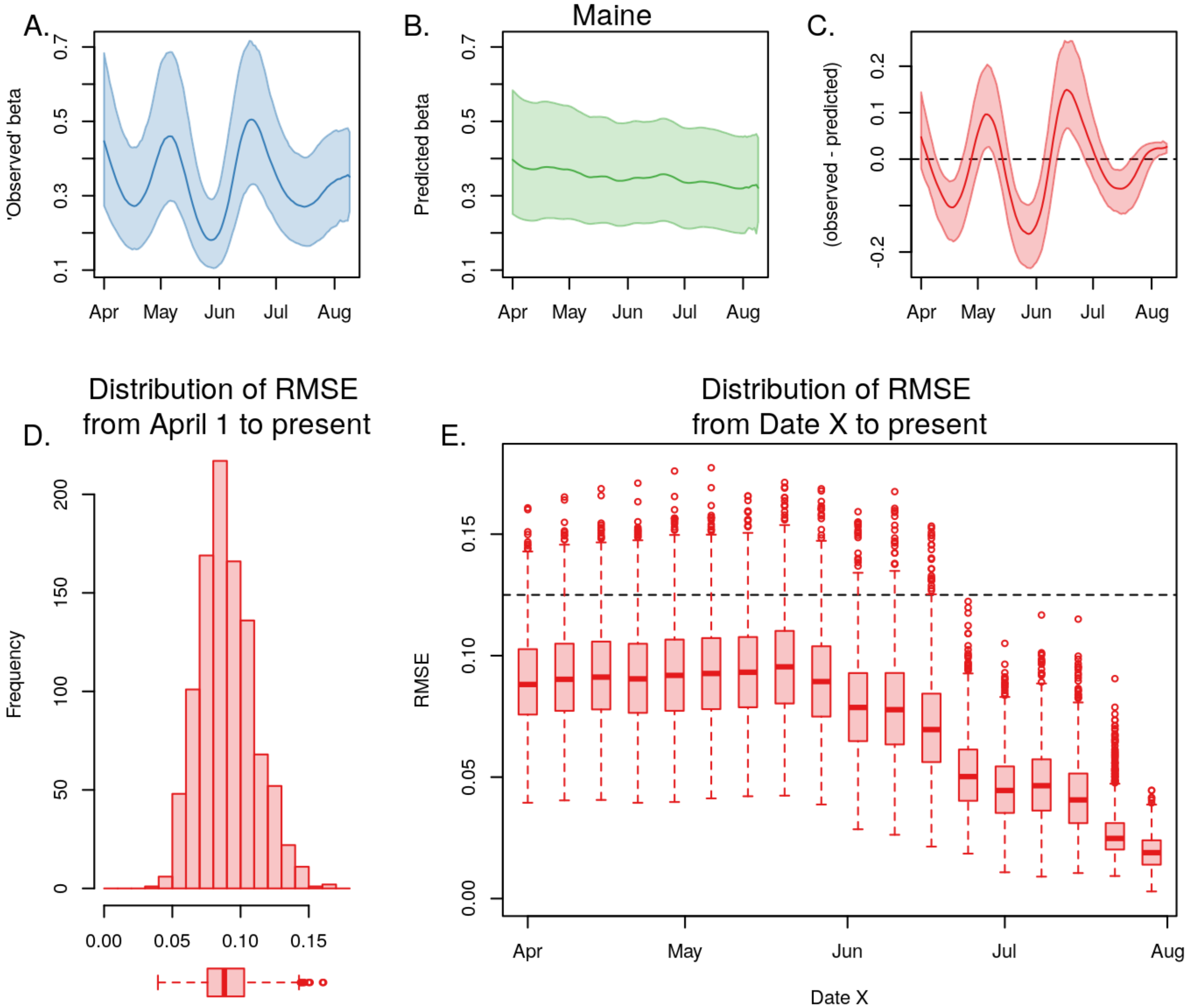

Maine: Detailed regression diagnostics. A: The SEIR *beta* parameter calculated directly from past input data on infections. **B:** The *beta* parameter predicted using a multivariate regression across all locations. **C:** The difference between the directly-calculated and predicted values for *beta*. Mean and uncertainty interval are shown across 1,000 posterior predictive draws over time. **D:** Histogram and box plot showing the distribution of root mean squared error (RMSE) for *beta* when aggregated across all dates from April 1 to present. **E:** Box plots showing the RMSE for aggregates of *beta* from a given date to the present across 1,000 posterior predictive draws.

21 Maryland: Detailed regression diagnostics

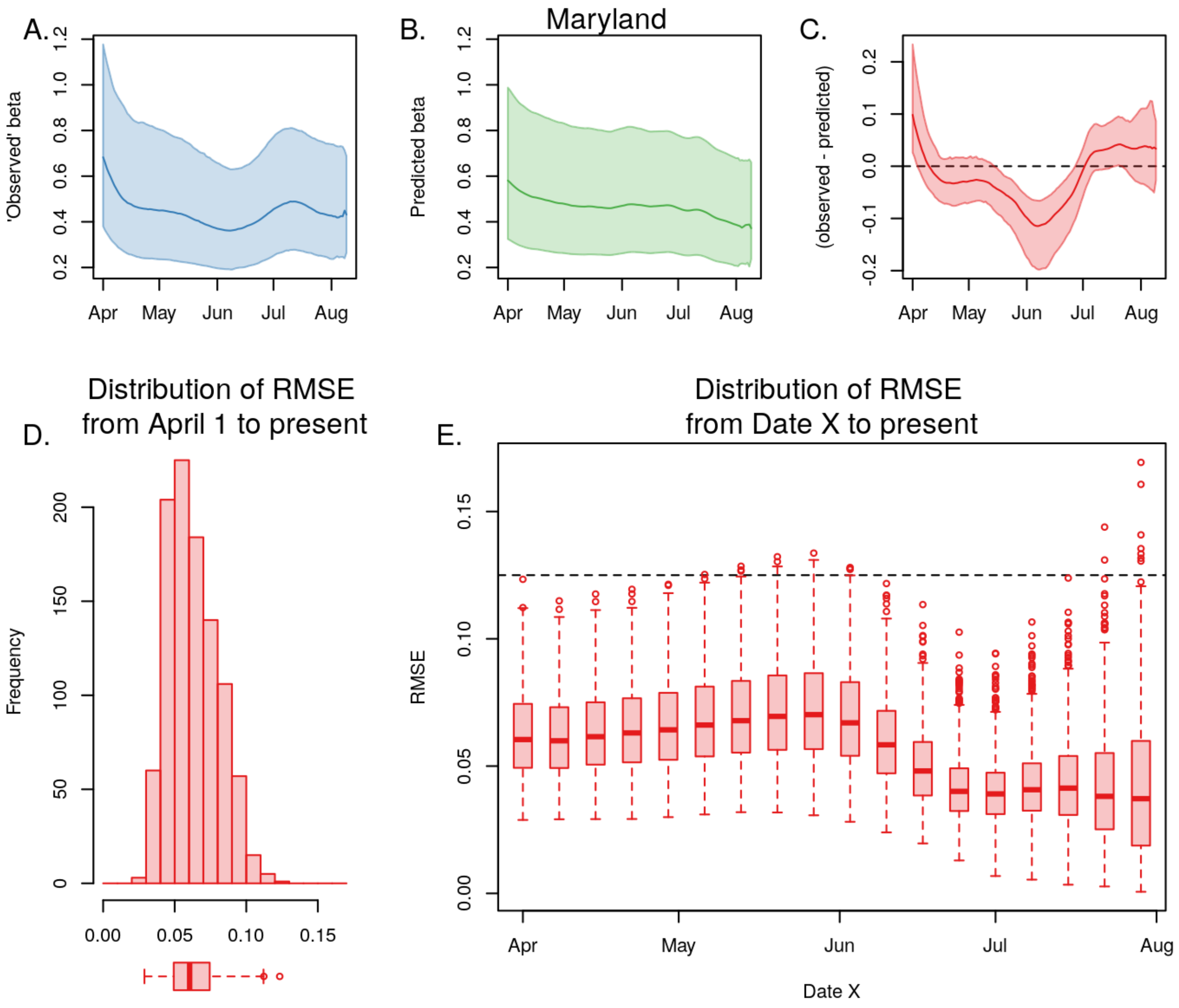

Maryland: Detailed regression diagnostics. A: The SEIR *beta* parameter calculated directly from past input data on infections. **B:** The *beta* parameter predicted using a multivariate regression across all locations. **C:** The difference between the directly-calculated and predicted values for *beta*. Mean and uncertainty interval are shown across 1,000 posterior predictive draws over time. **D:** Histogram and box plot showing the distribution of root mean squared error (RMSE) for *beta* when aggregated across all dates from April 1 to present. **E:** Box plots showing the RMSE for aggregates of *beta* from a given date to the present across 1,000 posterior predictive draws.

22 Massachusetts: Detailed regression diagnostics

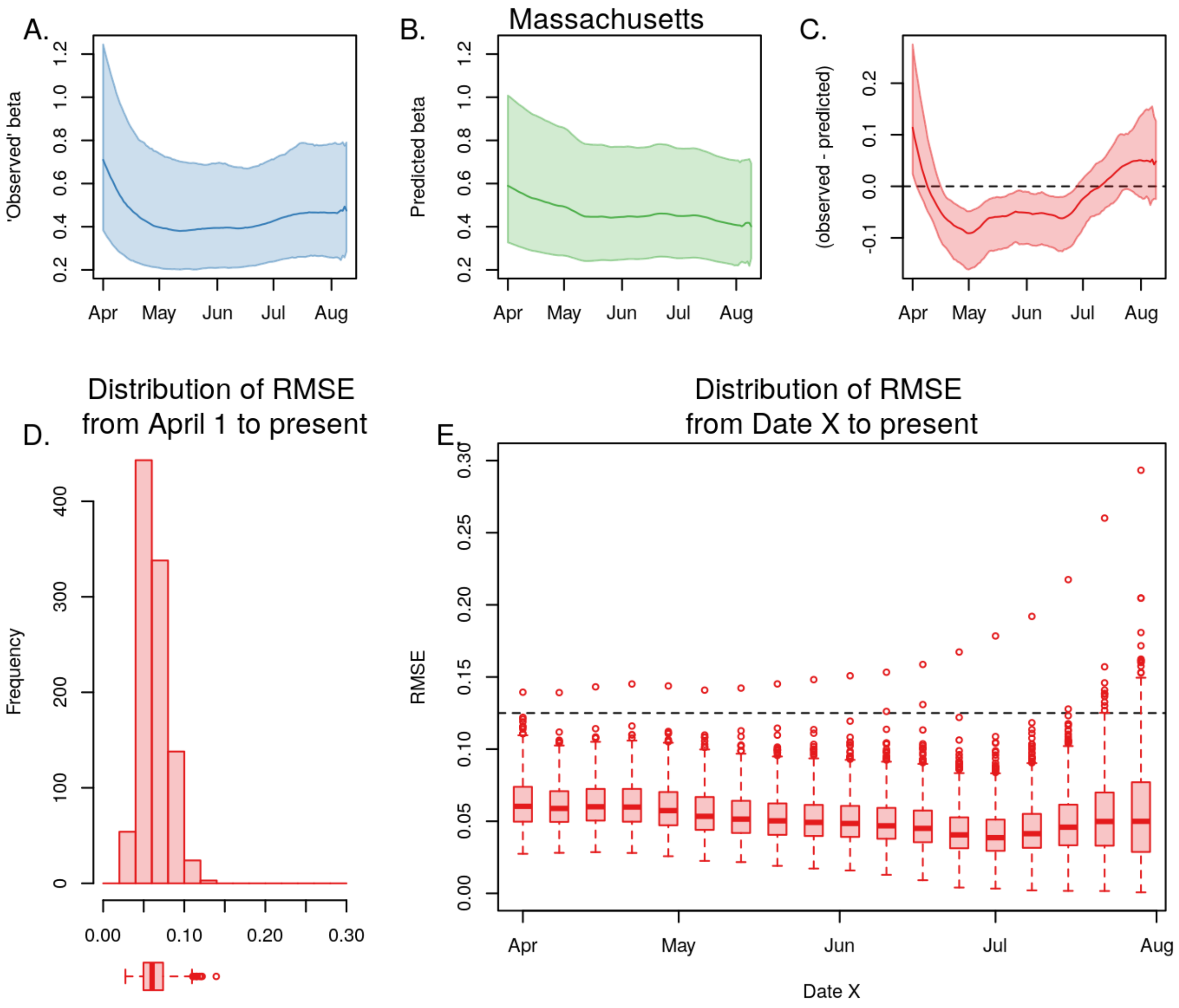

Massachusetts: Detailed regression diagnostics. **A:** The SEIR *beta* parameter calculated directly from past input data on infections. **B:** The *beta* parameter predicted using a multivariate regression across all locations. **C:** The difference between the directly-calculated and predicted values for *beta*. Mean and uncertainty interval are shown across 1,000 posterior predictive draws over time. **D:** Histogram and box plot showing the distribution of root mean squared error (RMSE) for *beta* when aggregated across all dates from April 1 to present. **E:** Box plots showing the RMSE for aggregates of *beta* from a given date to the present across 1,000 posterior predictive draws.

23 Michigan: Detailed regression diagnostics

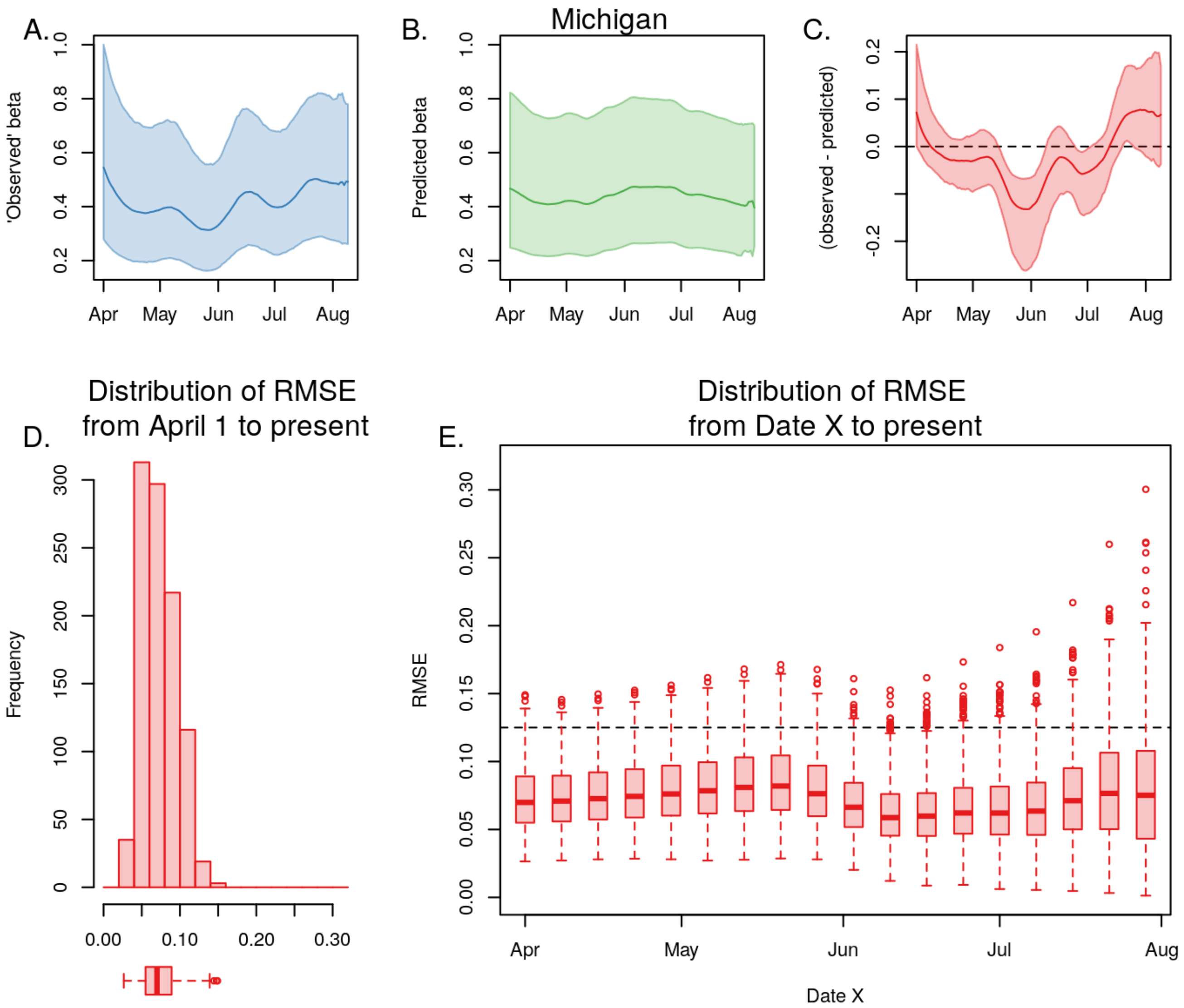

Michigan: Detailed regression diagnostics. A: The SEIR *beta* parameter calculated directly from past input data on infections. **B:** The *beta* parameter predicted using a multivariate regression across all locations. **C:** The difference between the directly-calculated and predicted values for *beta*. Mean and uncertainty interval are shown across 1,000 posterior predictive draws over time. **D:** Histogram and box plot showing the distribution of root mean squared error (RMSE) for *beta* when aggregated across all dates from April 1 to present. **E:** Box plots showing the RMSE for aggregates of *beta* from a given date to the present across 1,000 posterior predictive draws.

24 Minnesota: Detailed regression diagnostics

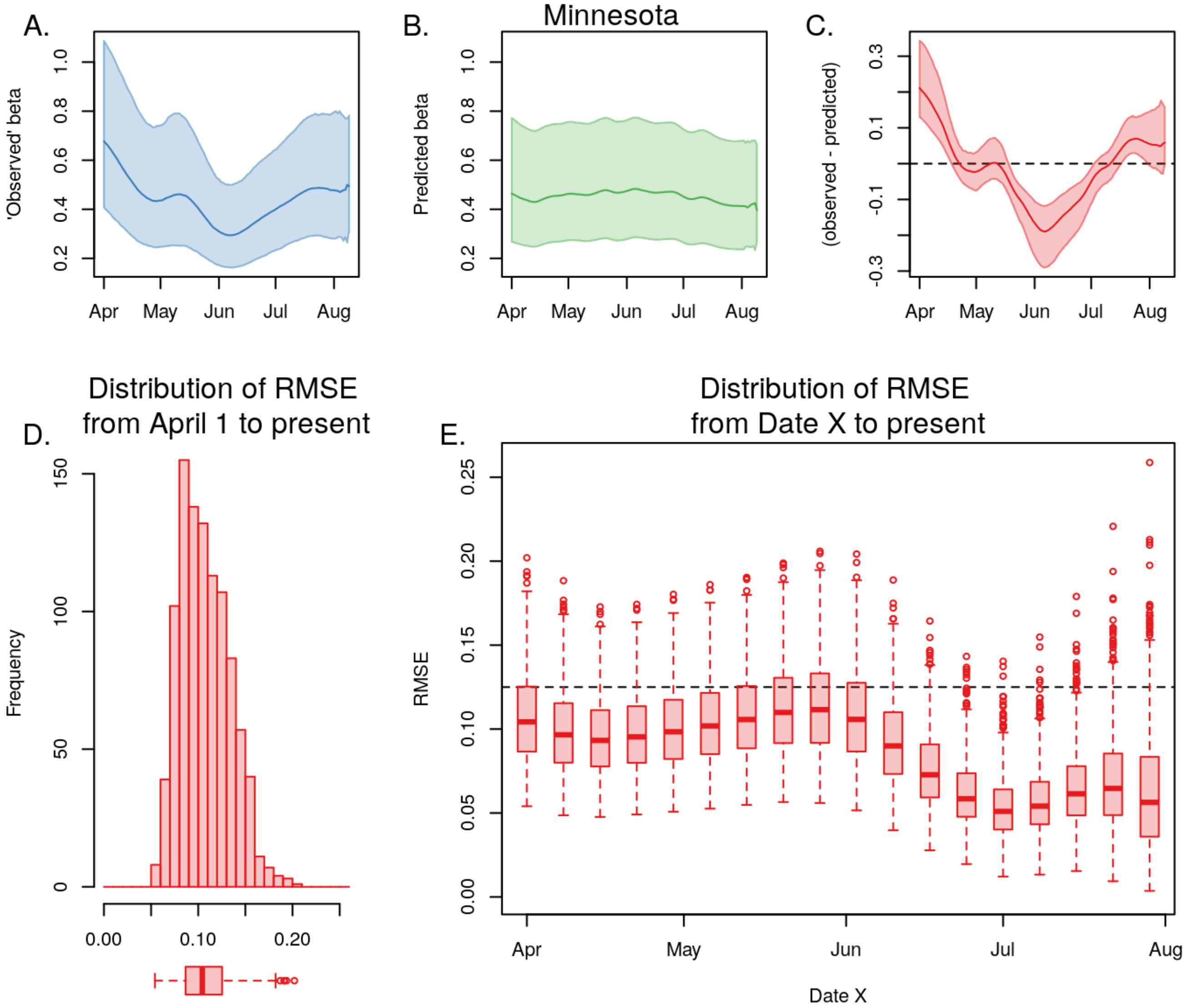

Minnesota: Detailed regression diagnostics. **A:** The SEIR *beta* parameter calculated directly from past input data on infections. **B:** The *beta* parameter predicted using a multivariate regression across all locations. **C:** The difference between the directly-calculated and predicted values for *beta*. Mean and uncertainty interval are shown across 1,000 posterior predictive draws over time. **D:** Histogram and box plot showing the distribution of root mean squared error (RMSE) for *beta* when aggregated across all dates from April 1 to present. **E:** Box plots showing the RMSE for aggregates of *beta* from a given date to the present across 1,000 posterior predictive draws.

25 Mississippi: Detailed regression diagnostics

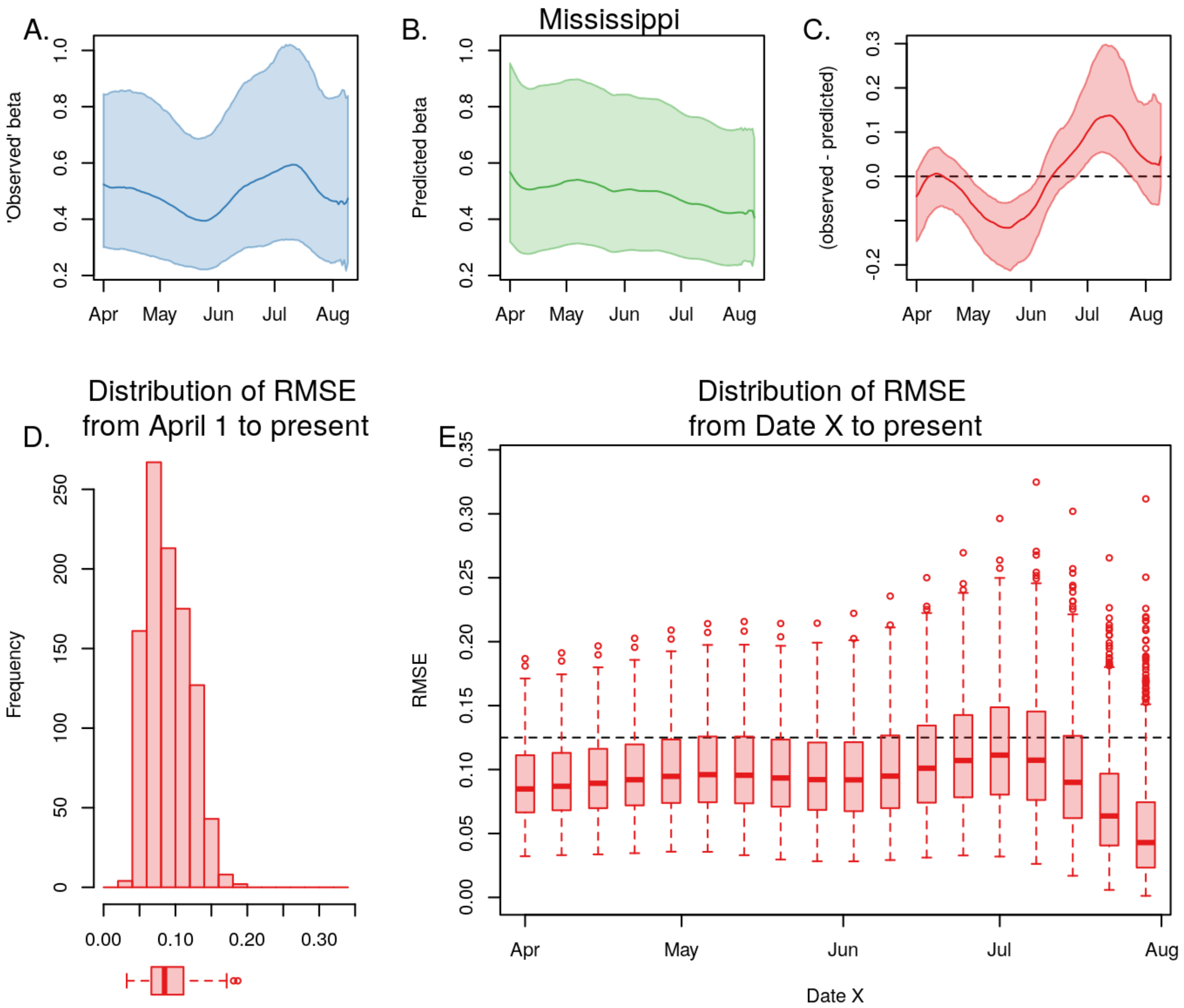

Mississippi: Detailed regression diagnostics. A: The SEIR *beta* parameter calculated directly from past input data on infections. **B:** The *beta* parameter predicted using a multivariate regression across all locations. **C:** The difference between the directly-calculated and predicted values for *beta*. Mean and uncertainty interval are shown across 1,000 posterior predictive draws over time. **D:** Histogram and box plot showing the distribution of root mean squared error (RMSE) for *beta* when aggregated across all dates from April 1 to present. **E:** Box plots showing the RMSE for aggregates of *beta* from a given date to the present across 1,000 posterior predictive draws.

26 Missouri: Detailed regression diagnostics

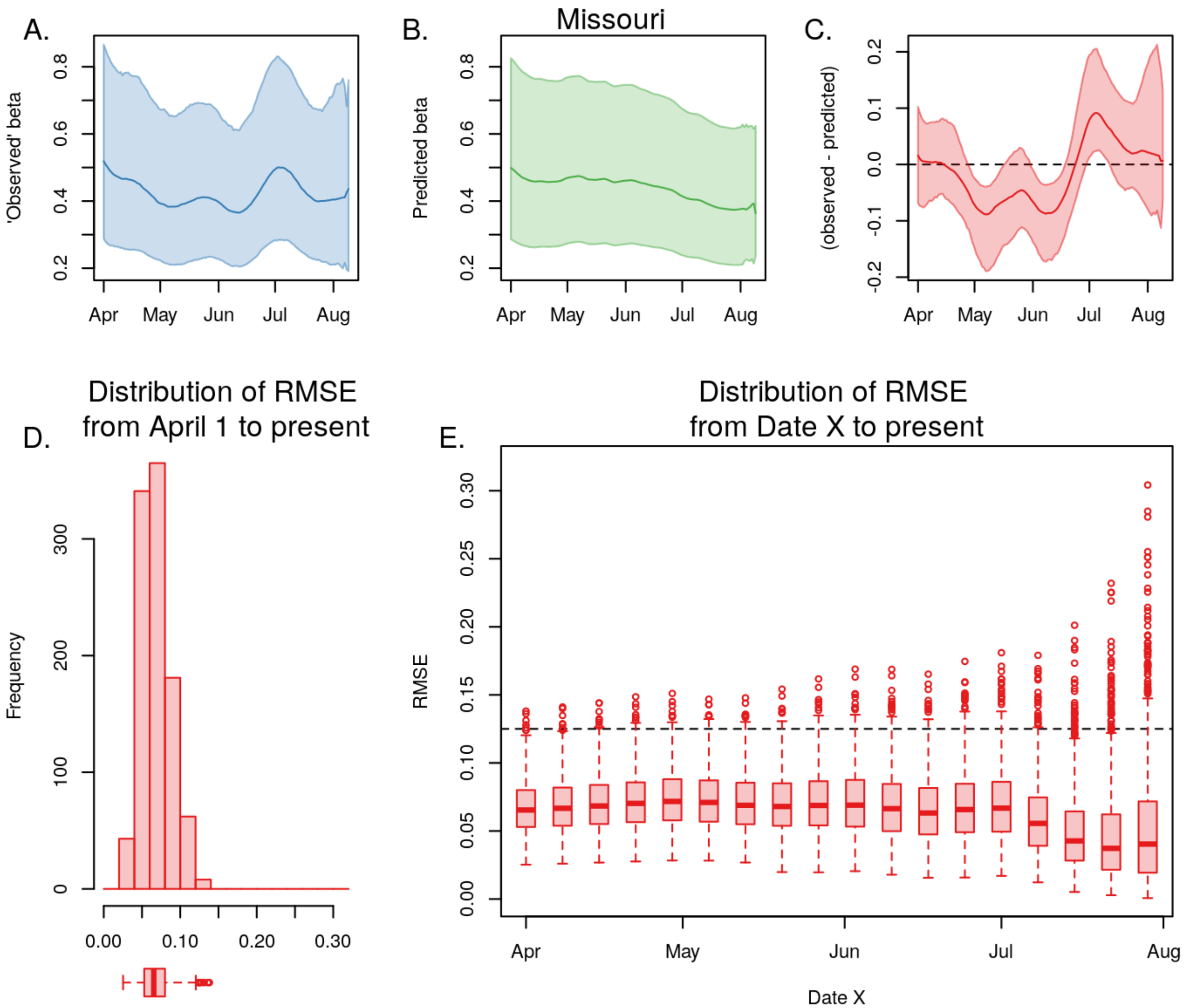

Missouri: Detailed regression diagnostics. A: The SEIR *beta* parameter calculated directly from past input data on infections. **B:** The *beta* parameter predicted using a multivariate regression across all locations. **C:** The difference between the directly-calculated and predicted values for *beta*. Mean and uncertainty interval are shown across 1,000 posterior predictive draws over time. **D:** Histogram and box plot showing the distribution of root mean squared error (RMSE) for *beta* when aggregated across all dates from April 1 to present. **E:** Box plots showing the RMSE for aggregates of *beta* from a given date to the present across 1,000 posterior predictive draws.

27 Montana: Detailed regression diagnostics

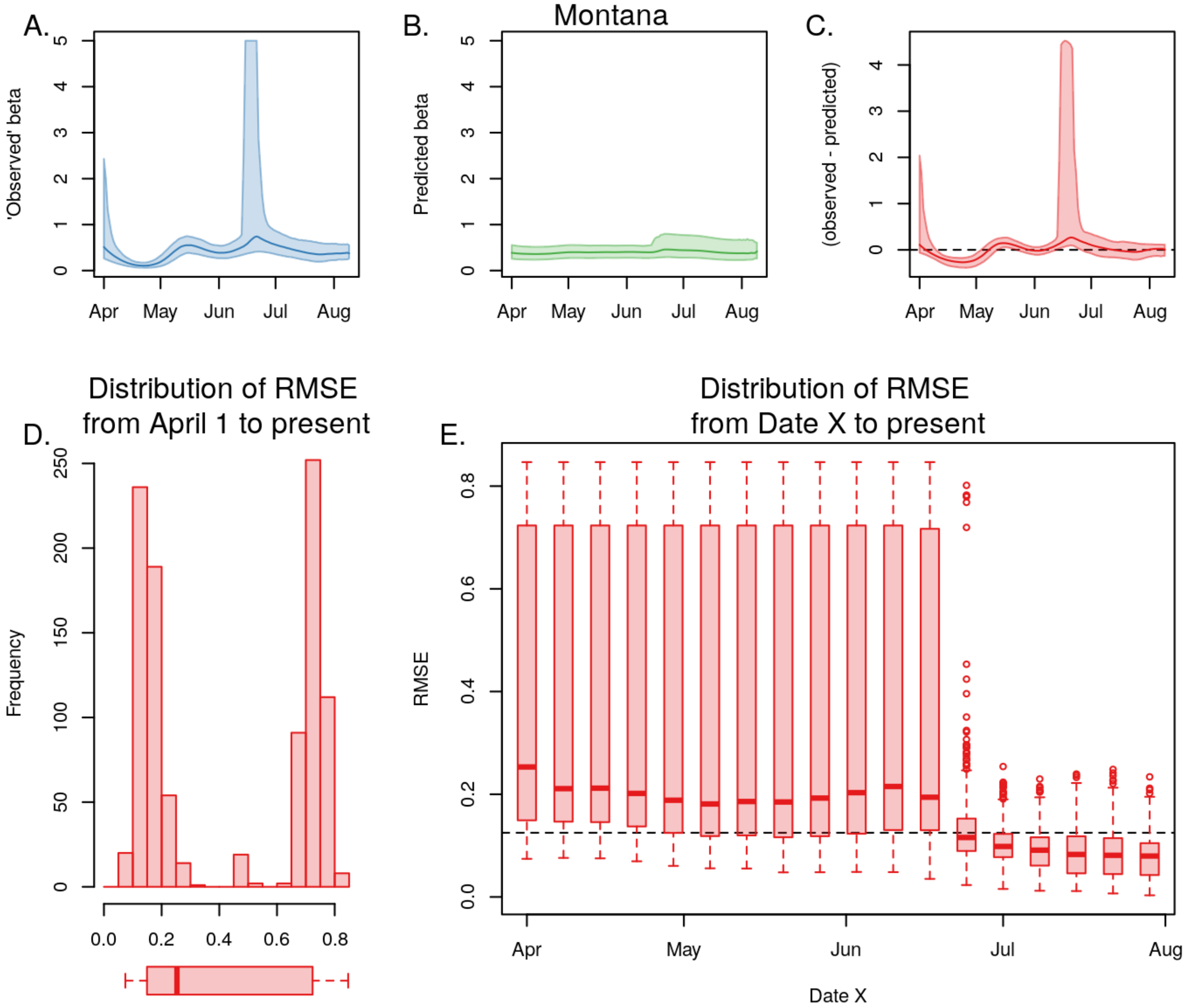

Montana: Detailed regression diagnostics. **A:** The SEIR *beta* parameter calculated directly from past input data on infections. **B:** The *beta* parameter predicted using a multivariate regression across all locations. **C:** The difference between the directly-calculated and predicted values for *beta*. Mean and uncertainty interval are shown across 1,000 posterior predictive draws over time. **D:** Histogram and box plot showing the distribution of root mean squared error (RMSE) for *beta* when aggregated across all dates from April 1 to present. **E:** Box plots showing the RMSE for aggregates of *beta* from a given date to the present across 1,000 posterior predictive draws.

28 Nebraska: Detailed regression diagnostics

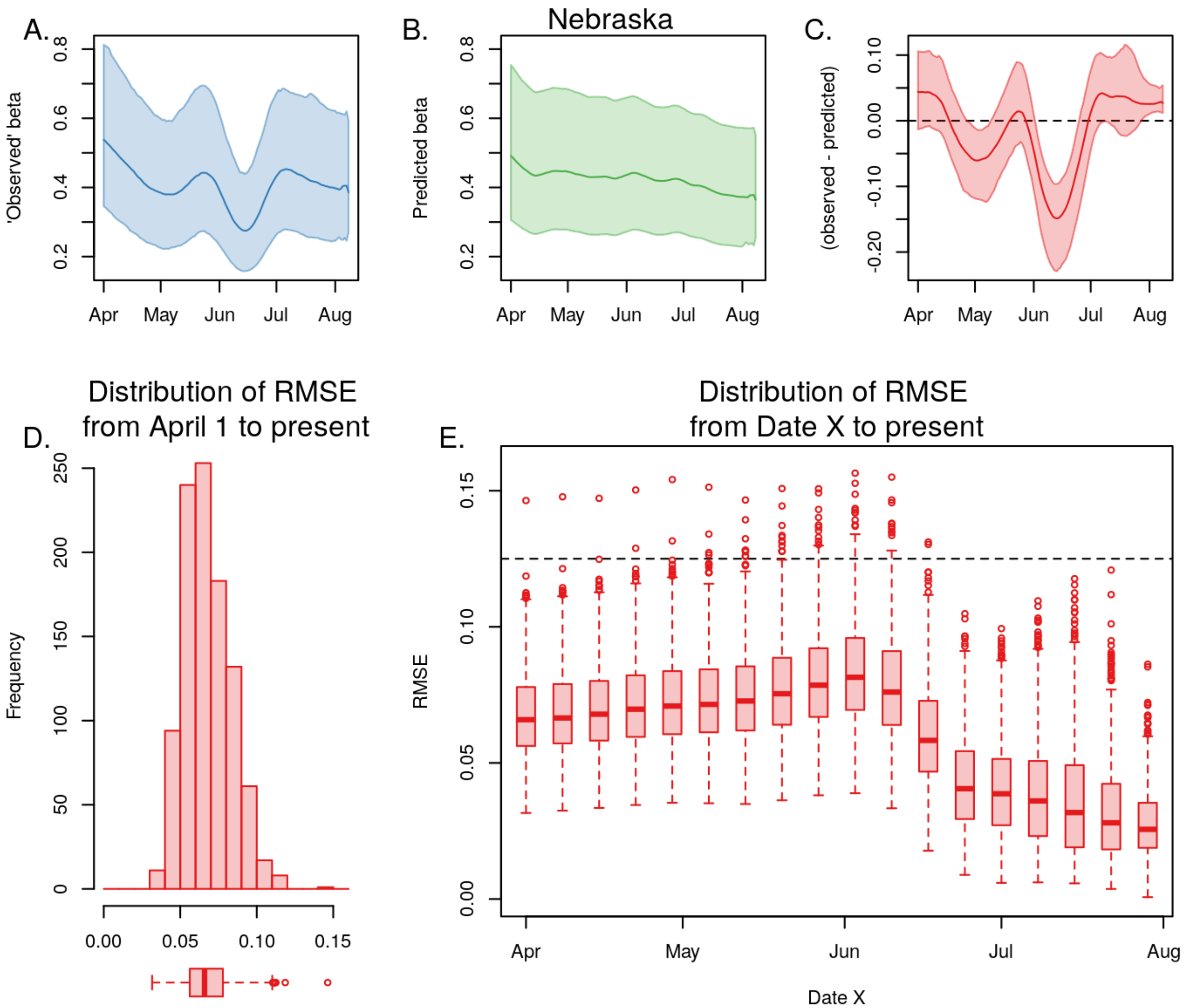

Nebraska: Detailed regression diagnostics. A: The SEIR *beta* parameter calculated directly from past input data on infections. **B:** The *beta* parameter predicted using a multivariate regression across all locations. **C:** The difference between the directly-calculated and predicted values for *beta*. Mean and uncertainty interval are shown across 1,000 posterior predictive draws over time. **D:** Histogram and box plot showing the distribution of root mean squared error (RMSE) for *beta* when aggregated across all dates from April 1 to present. **E:** Box plots showing the RMSE for aggregates of *beta* from a given date to the present across 1,000 posterior predictive draws.

29 Nevada: Detailed regression diagnostics

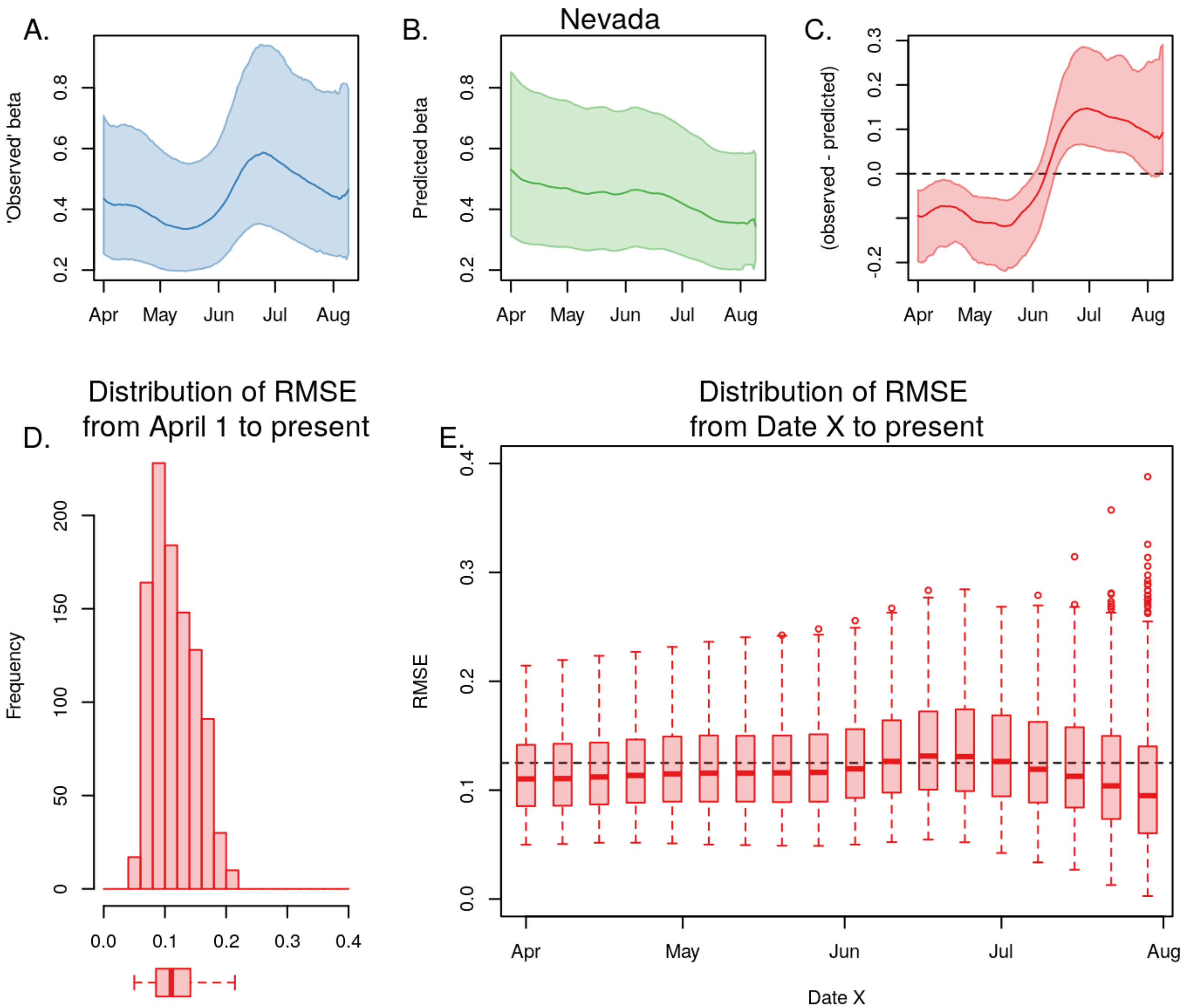

Nevada: Detailed regression diagnostics. **A:** The SEIR *beta* parameter calculated directly from past input data on infections. **B:** The *beta* parameter predicted using a multivariate regression across all locations. **C:** The difference between the directly-calculated and predicted values for *beta*. Mean and uncertainty interval are shown across 1,000 posterior predictive draws over time. **D:** Histogram and box plot showing the distribution of root mean squared error (RMSE) for *beta* when aggregated across all dates from April 1 to present. **E:** Box plots showing the RMSE for aggregates of *beta* from a given date to the present across 1,000 posterior predictive draws.

30 New Hampshire: Detailed regression diagnostics

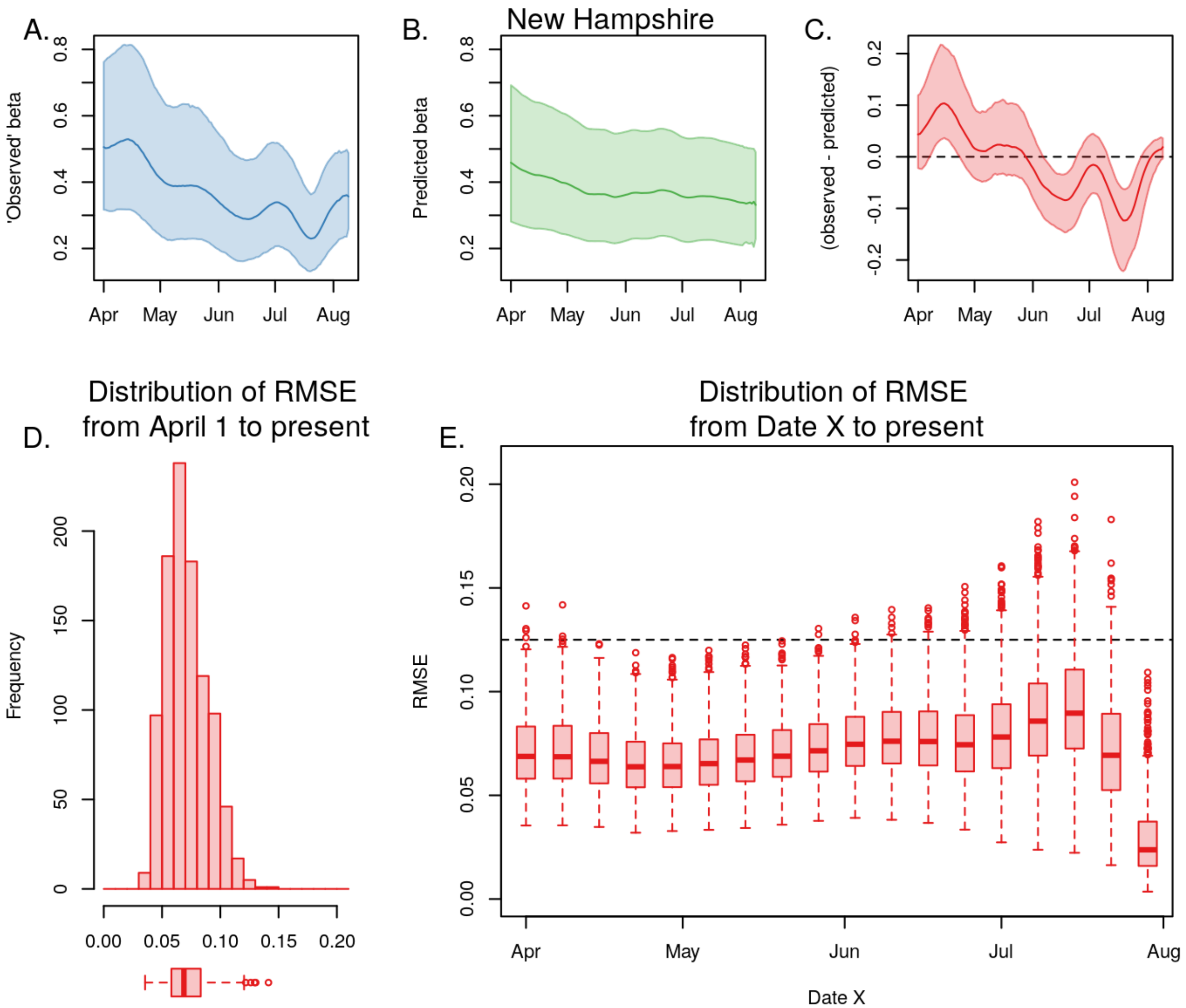

New Hampshire: Detailed regression diagnostics. A: The SEIR *beta* parameter calculated directly from past input data on infections. **B:** The *beta* parameter predicted using a multivariate regression across all locations. **C:** The difference between the directly-calculated and predicted values for *beta*. Mean and uncertainty interval are shown across 1,000 posterior predictive draws over time. **D:** Histogram and box plot showing the distribution of root mean squared error (RMSE) for *beta* when aggregated across all dates from April 1 to present. **E:** Box plots showing the RMSE for aggregates of *beta* from a given date to the present across 1,000 posterior predictive draws.

31 New Jersey: Detailed regression diagnostics

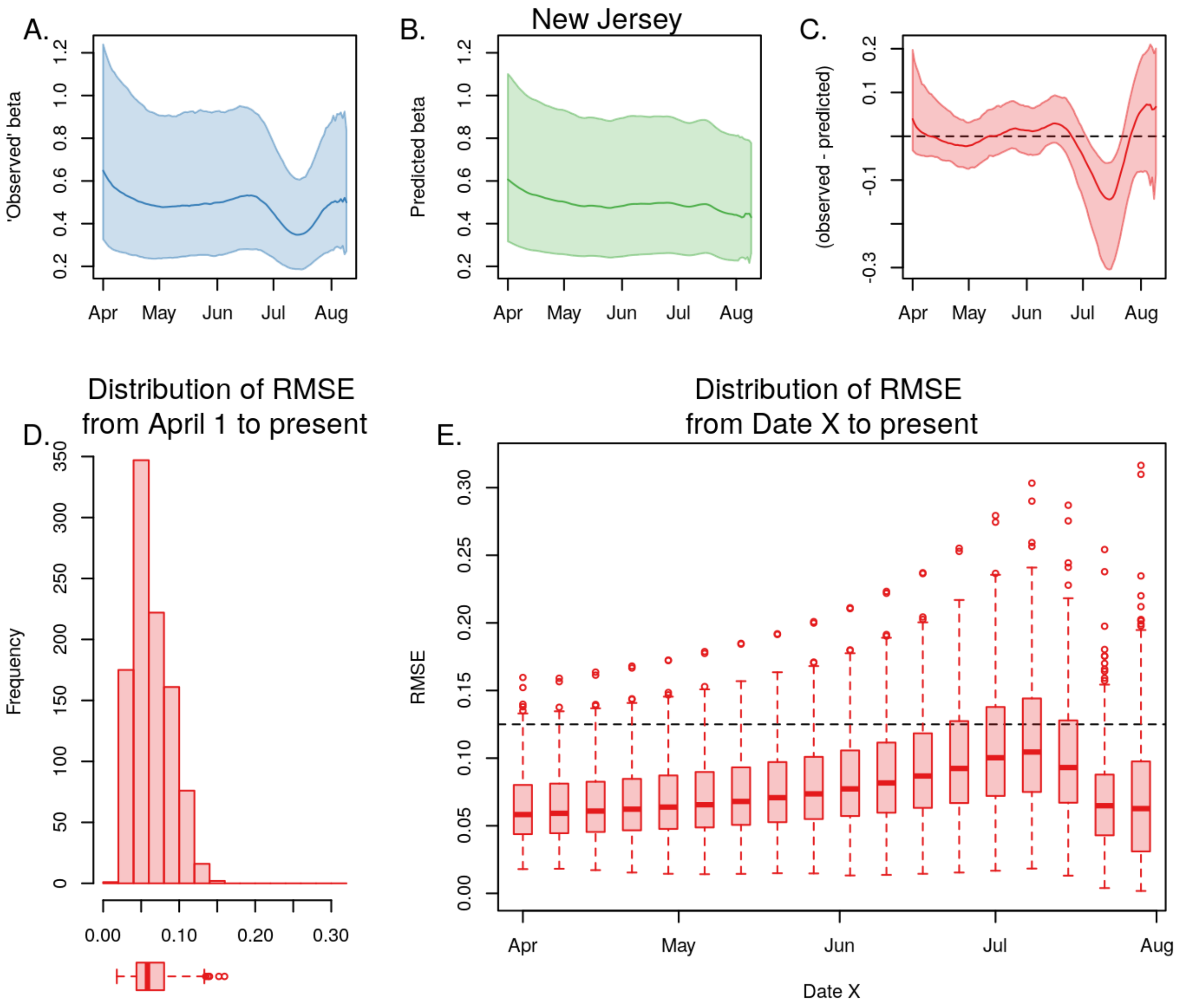

New Jersey: Detailed regression diagnostics. A: The SEIR *beta* parameter calculated directly from past input data on infections. **B:** The *beta* parameter predicted using a multivariate regression across all locations. **C:** The difference between the directly-calculated and predicted values for *beta*. Mean and uncertainty interval are shown across 1,000 posterior predictive draws over time. **D:** Histogram and box plot showing the distribution of root mean squared error (RMSE) for *beta* when aggregated across all dates from April 1 to present. **E:** Box plots showing the RMSE for aggregates of *beta* from a given date to the present across 1,000 posterior predictive draws.

32 New Mexico: Detailed regression diagnostics

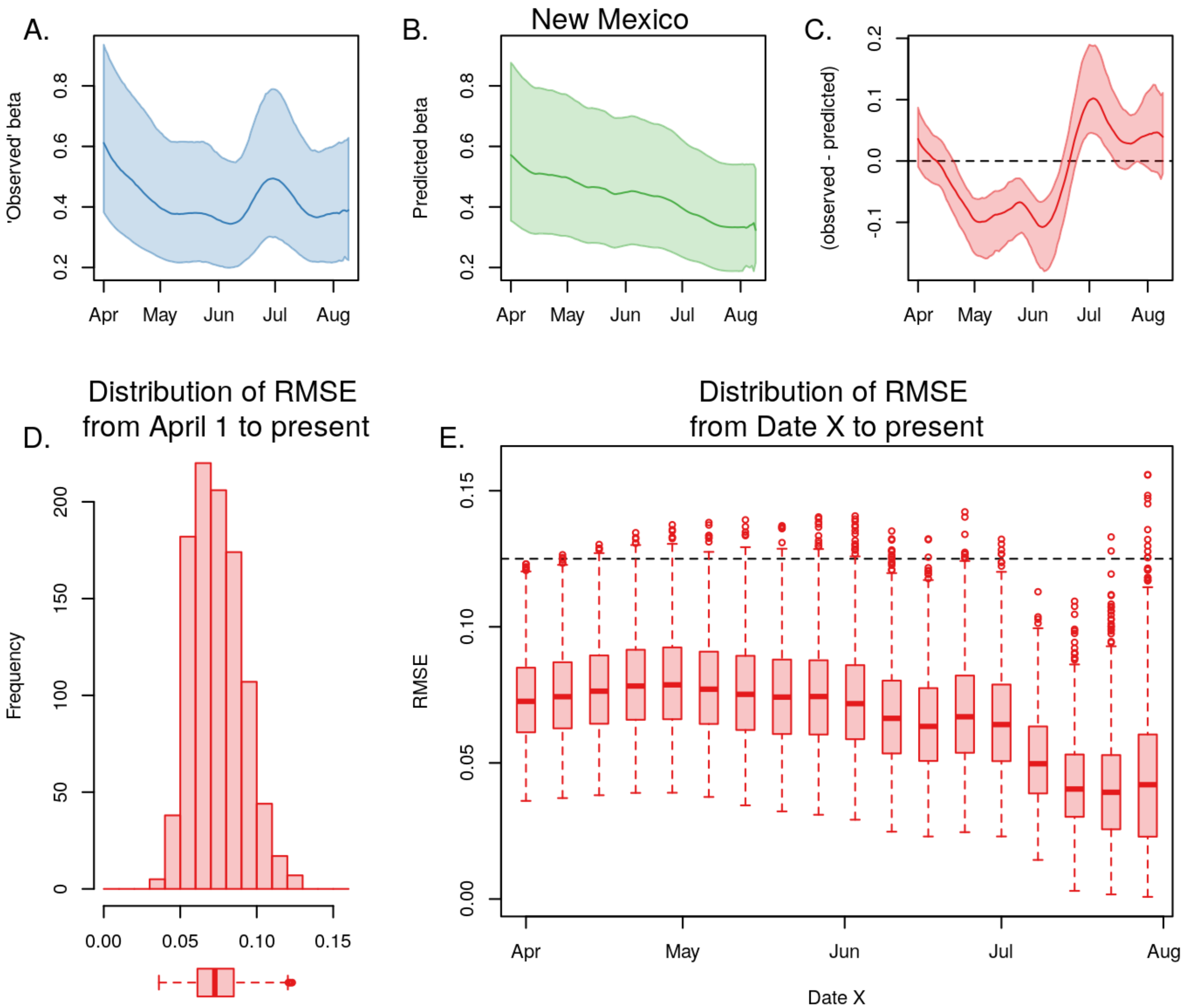

New Mexico: Detailed regression diagnostics. **A:** The SEIR *beta* parameter calculated directly from past input data on infections. **B:** The *beta* parameter predicted using a multivariate regression across all locations. **C:** The difference between the directly-calculated and predicted values for *beta*. Mean and uncertainty interval are shown across 1,000 posterior predictive draws over time. **D:** Histogram and box plot showing the distribution of root mean squared error (RMSE) for *beta* when aggregated across all dates from April 1 to present. **E:** Box plots showing the RMSE for aggregates of *beta* from a given date to the present across 1,000 posterior predictive draws.

33 New York: Detailed regression diagnostics

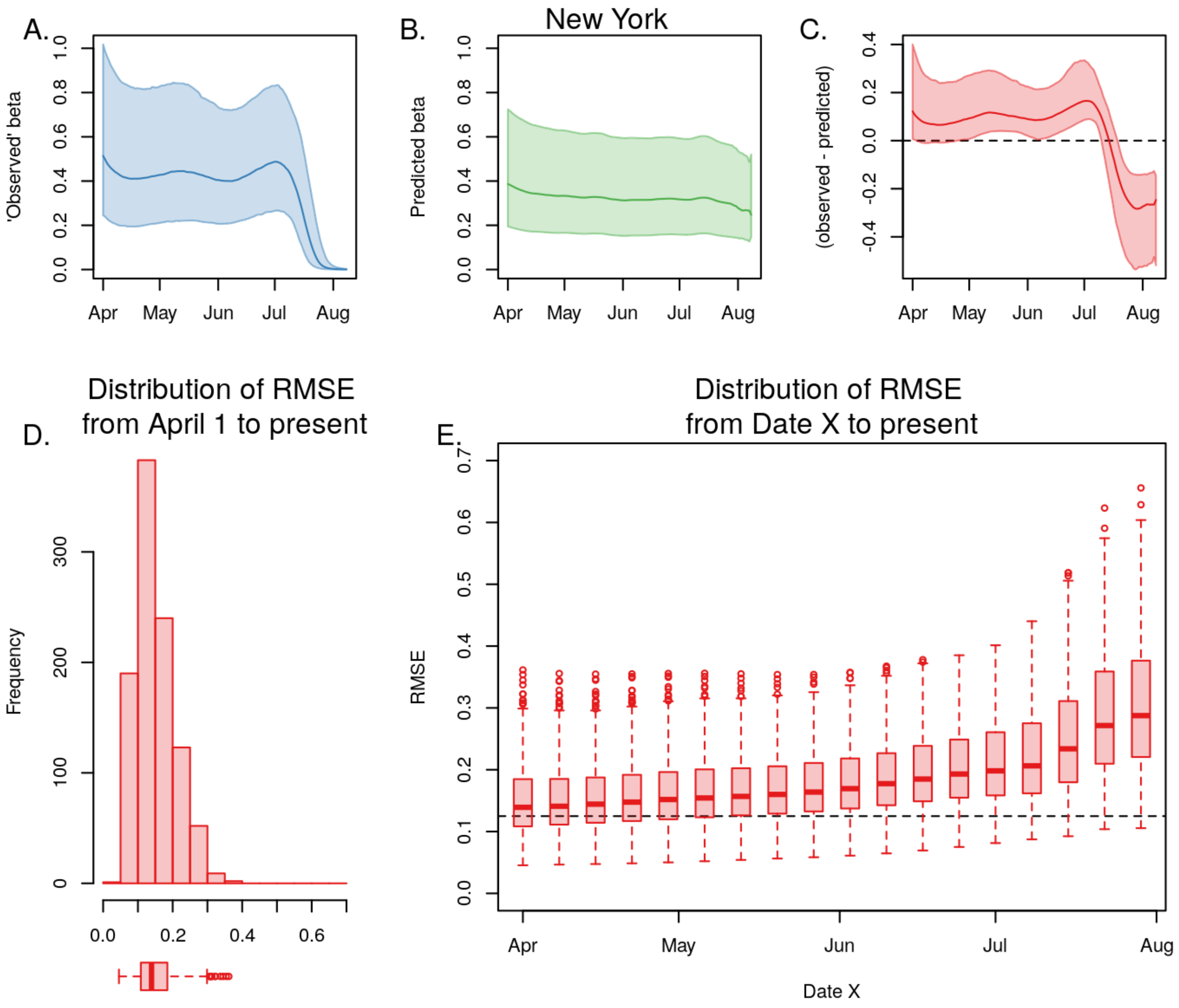

New York: Detailed regression diagnostics. A: The SEIR *beta* parameter calculated directly from past input data on infections. **B:** The *beta* parameter predicted using a multivariate regression across all locations. **C:** The difference between the directly-calculated and predicted values for *beta*. Mean and uncertainty interval are shown across 1,000 posterior predictive draws over time. **D:** Histogram and box plot showing the distribution of root mean squared error (RMSE) for *beta* when aggregated across all dates from April 1 to present. **E:** Box plots showing the RMSE for aggregates of *beta* from a given date to the present across 1,000 posterior predictive draws.

34 North Carolina: Detailed regression diagnostics

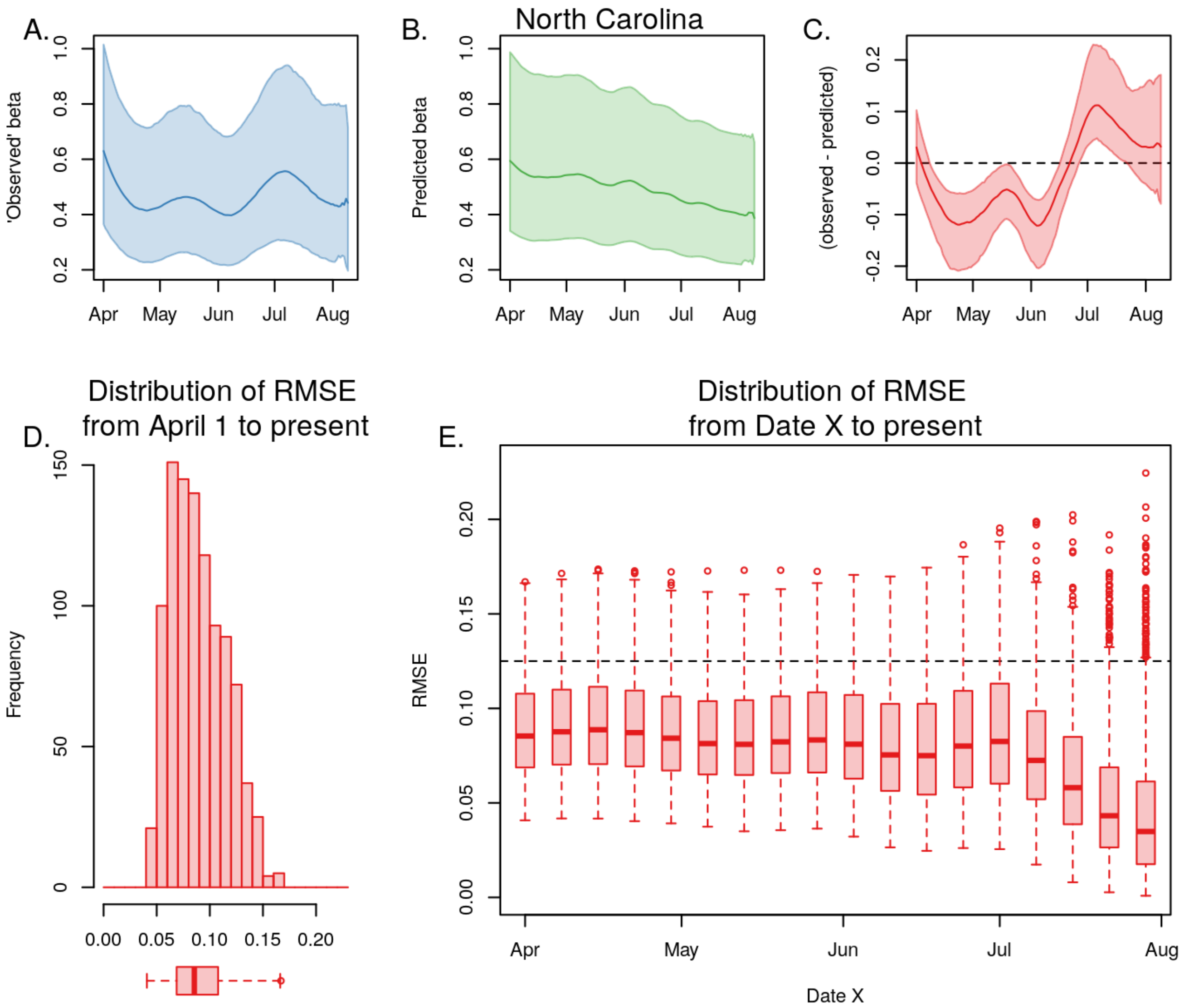

North Carolina: Detailed regression diagnostics. A: The SEIR *beta* parameter calculated directly from past input data on infections. **B:** The *beta* parameter predicted using a multivariate regression across all locations. **C:** The difference between the directly-calculated and predicted values for *beta*. Mean and uncertainty interval are shown across 1,000 posterior predictive draws over time. **D:** Histogram and box plot showing the distribution of root mean squared error (RMSE) for *beta* when aggregated across all dates from April 1 to present. **E:** Box plots showing the RMSE for aggregates of *beta* from a given date to the present across 1,000 posterior predictive draws.

35 North Dakota: Detailed regression diagnostics

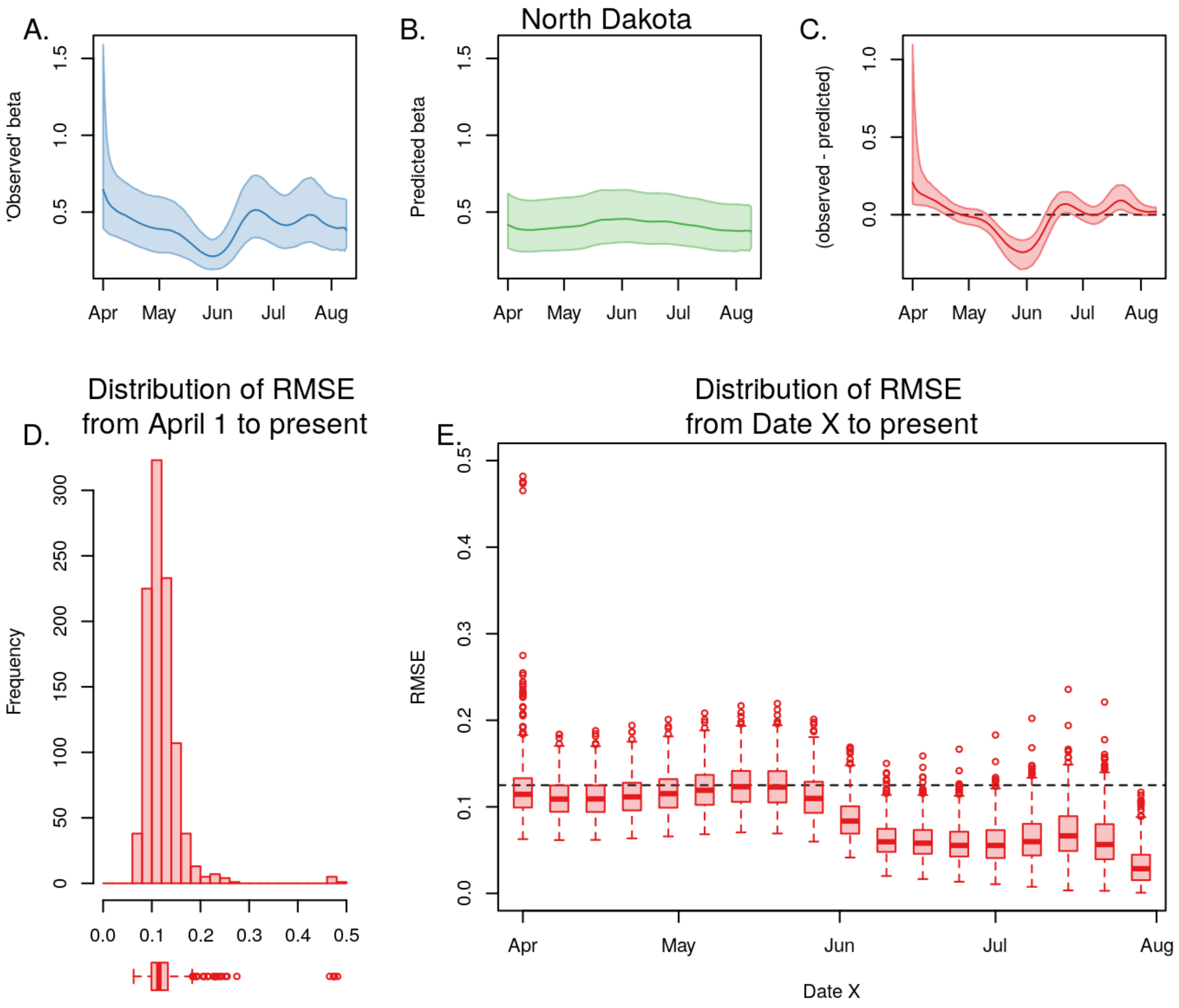

North Dakota: Detailed regression diagnostics. A: The SEIR *beta* parameter calculated directly from past input data on infections. **B:** The *beta* parameter predicted using a multivariate regression across all locations. **C:** The difference between the directly-calculated and predicted values for *beta*. Mean and uncertainty interval are shown across 1,000 posterior predictive draws over time. **D:** Histogram and box plot showing the distribution of root mean squared error (RMSE) for *beta* when aggregated across all dates from April 1 to present. **E:** Box plots showing the RMSE for aggregates of *beta* from a given date to the present across 1,000 posterior predictive draws.

36 Ohio: Detailed regression diagnostics

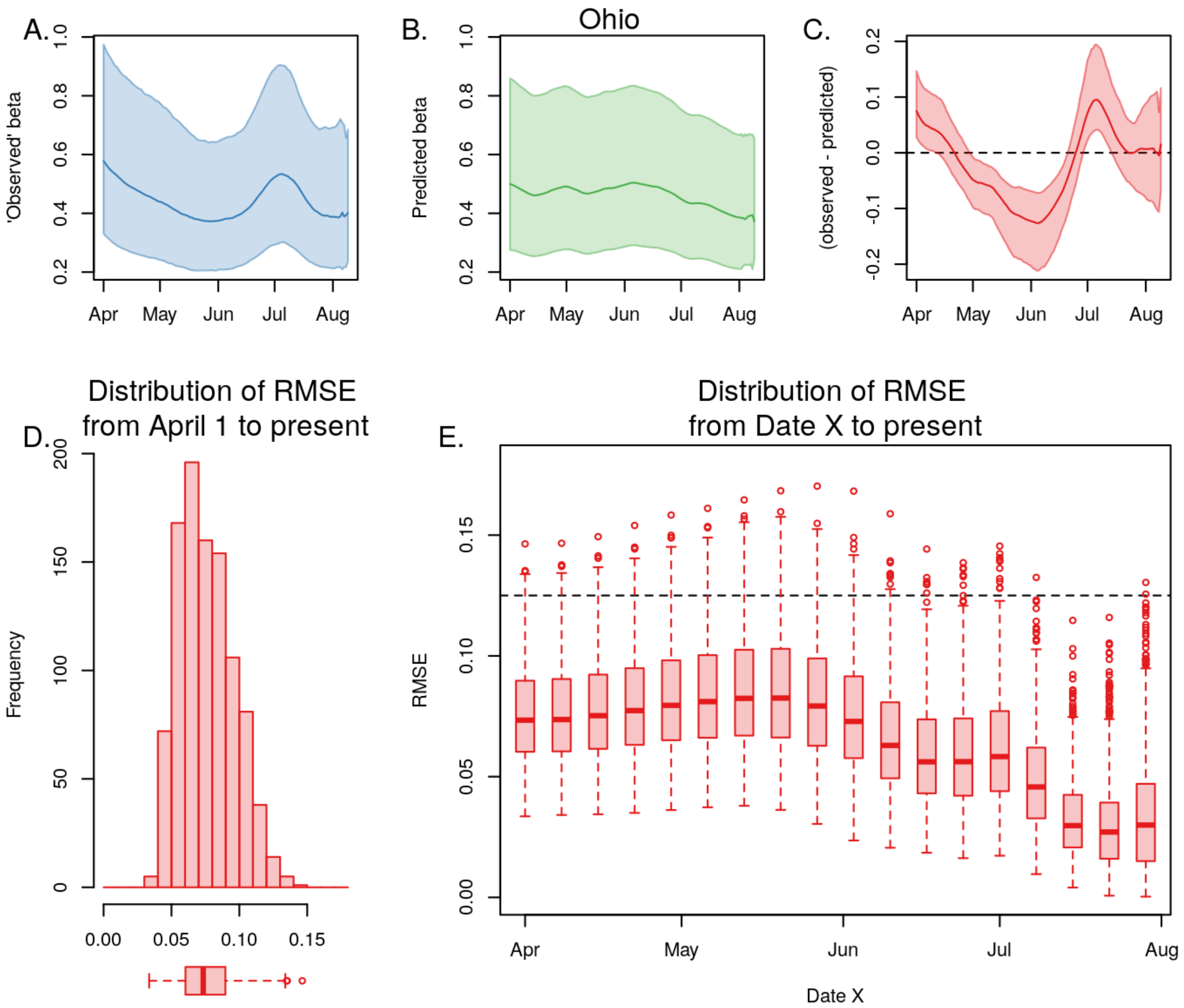

Ohio: Detailed regression diagnostics. **A:** The SEIR *beta* parameter calculated directly from past input data on infections. **B:** The *beta* parameter predicted using a multivariate regression across all locations. **C:** The difference between the directly-calculated and predicted values for *beta*. Mean and uncertainty interval are shown across 1,000 posterior predictive draws over time. **D:** Histogram and box plot showing the distribution of root mean squared error (RMSE) for *beta* when aggregated across all dates from April 1 to present. **E:** Box plots showing the RMSE for aggregates of *beta* from a given date to the present across 1,000 posterior predictive draws.

37 Oklahoma: Detailed regression diagnostics

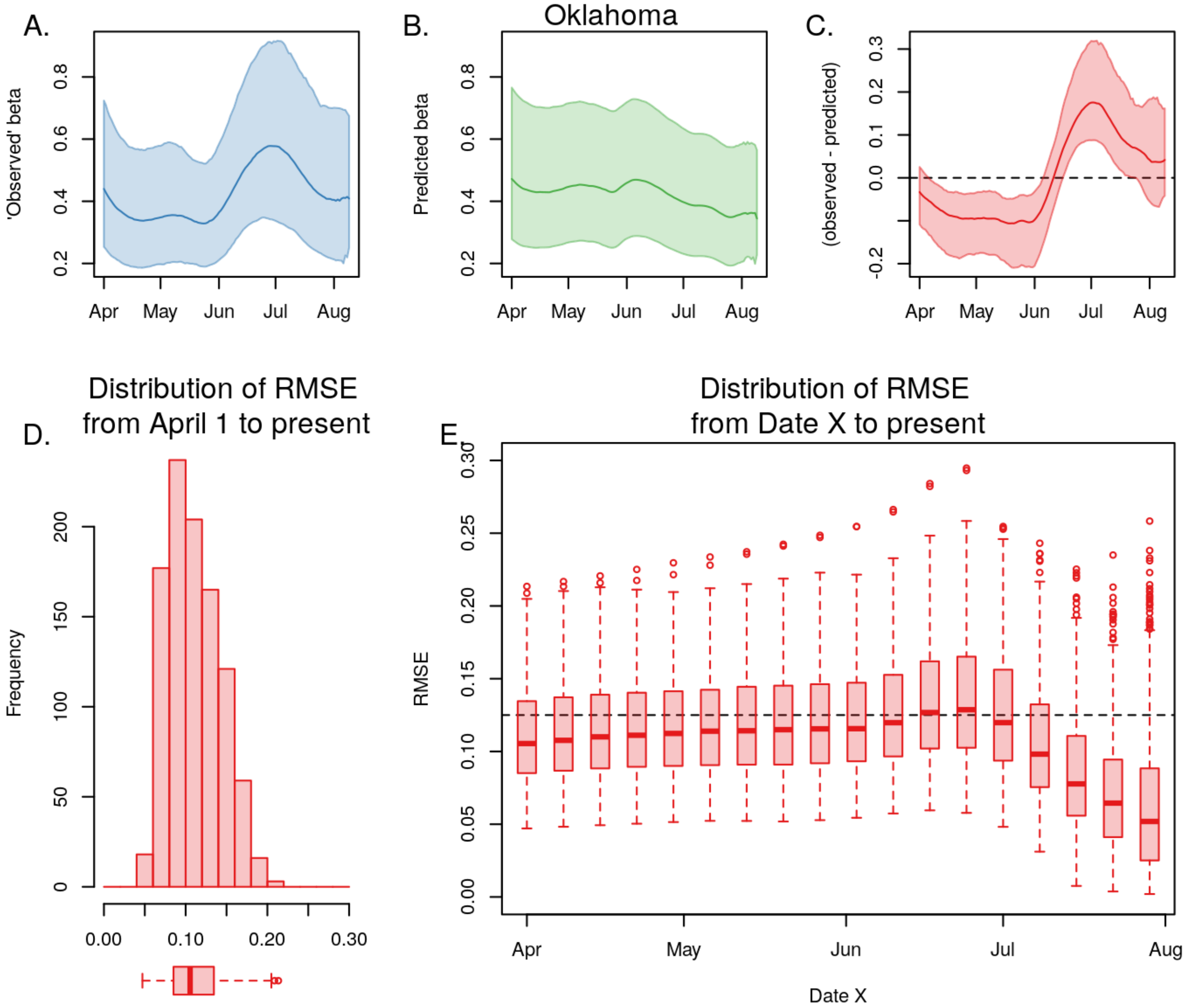

Oklahoma: Detailed regression diagnostics. A: The SEIR *beta* parameter calculated directly from past input data on infections. **B:** The *beta* parameter predicted using a multivariate regression across all locations. **C:** The difference between the directly-calculated and predicted values for *beta*. Mean and uncertainty interval are shown across 1,000 posterior predictive draws over time. **D:** Histogram and box plot showing the distribution of root mean squared error (RMSE) for *beta* when aggregated across all dates from April 1 to present. **E:** Box plots showing the RMSE for aggregates of *beta* from a given date to the present across 1,000 posterior predictive draws.

38 Oregon: Detailed regression diagnostics

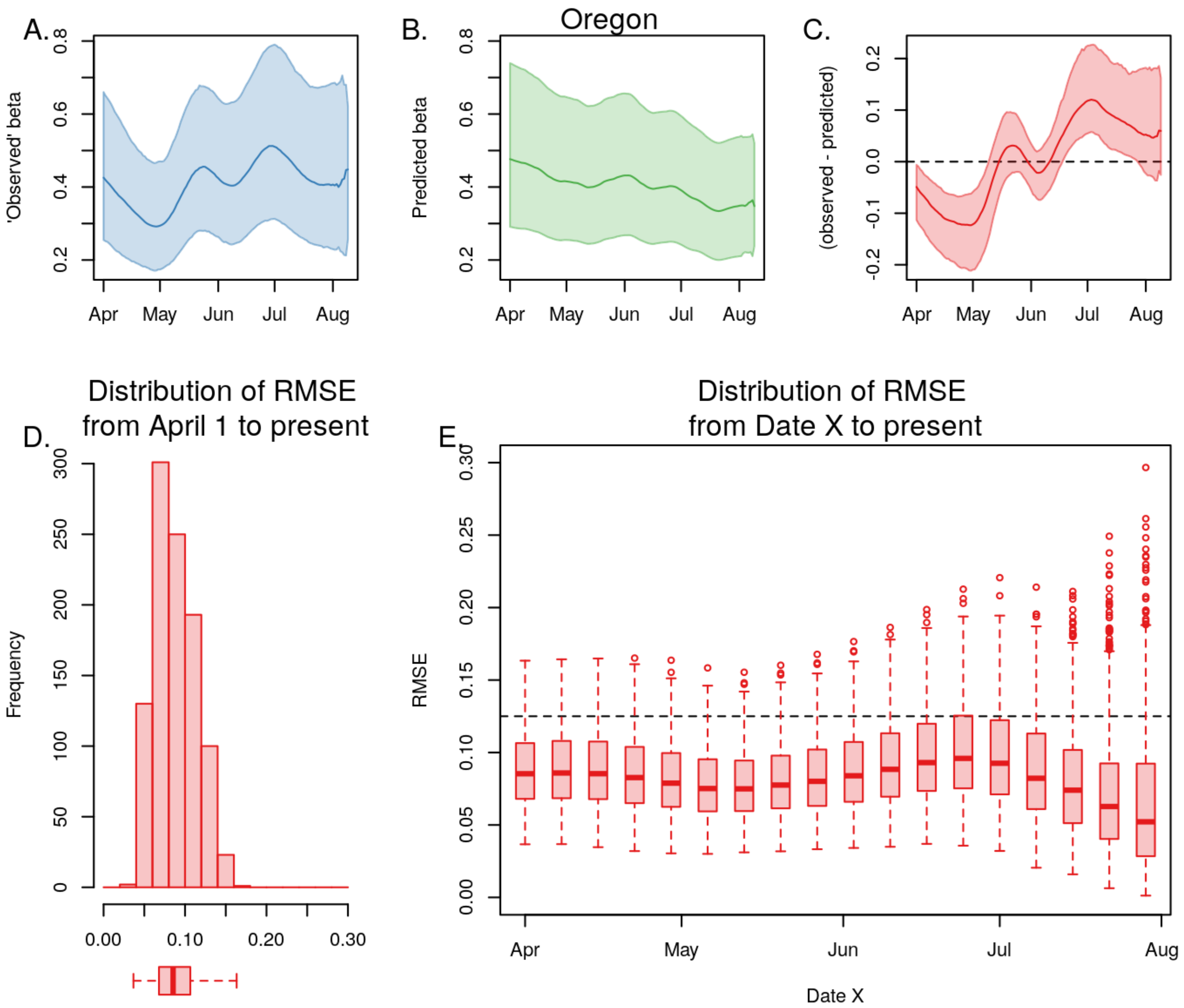

Oregon: Detailed regression diagnostics. A: The SEIR *beta* parameter calculated directly from past input data on infections. **B:** The *beta* parameter predicted using a multivariate regression across all locations. **C:** The difference between the directly-calculated and predicted values for *beta*. Mean and uncertainty interval are shown across 1,000 posterior predictive draws over time. **D:** Histogram and box plot showing the distribution of root mean squared error (RMSE) for *beta* when aggregated across all dates from April 1 to present. **E:** Box plots showing the RMSE for aggregates of *beta* from a given date to the present across 1,000 posterior predictive draws.

39 Pennsylvania: Detailed regression diagnostics

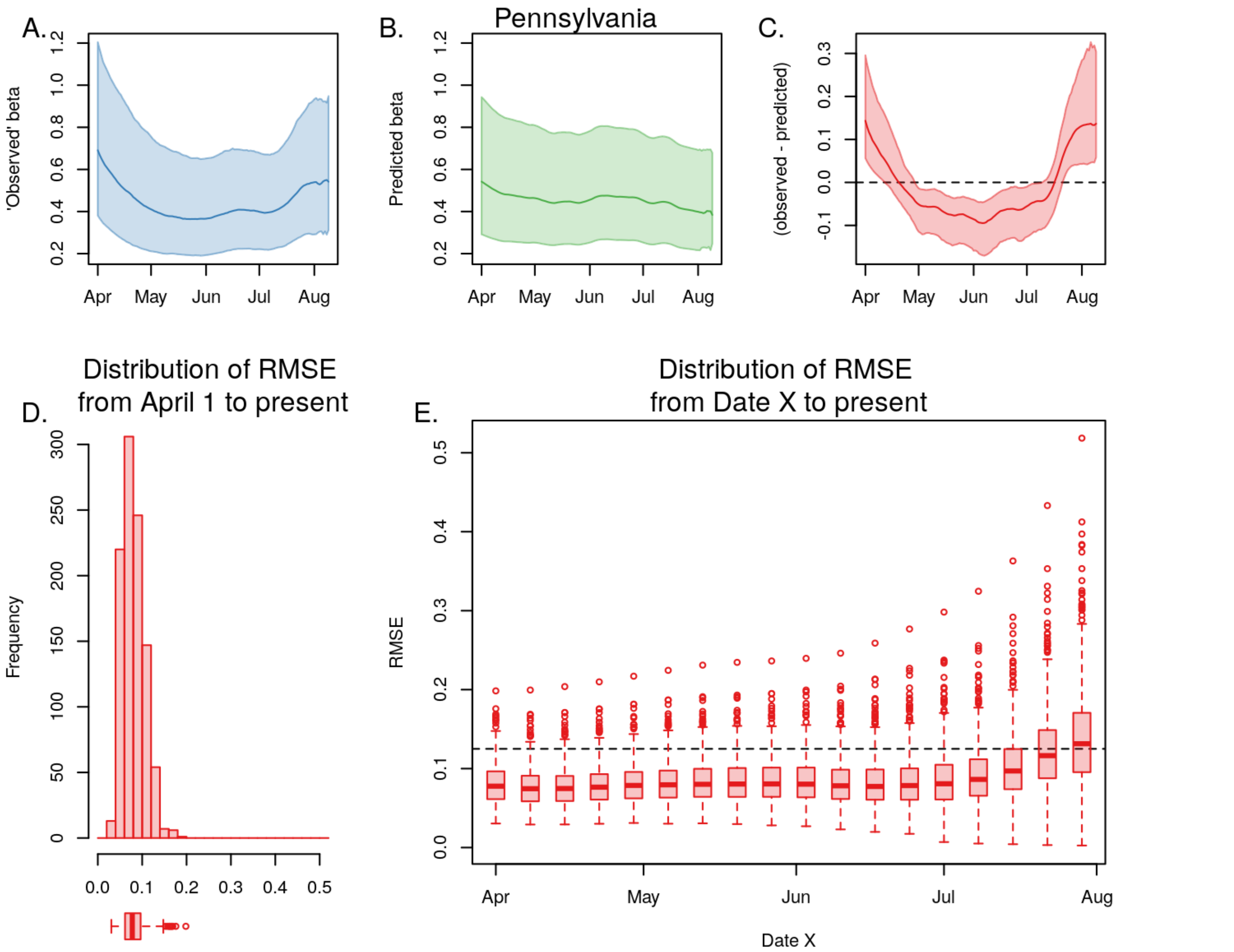

Pennsylvania: Detailed regression diagnostics. A: The SEIR *beta* parameter calculated directly from past input data on infections. **B:** The *beta* parameter predicted using a multivariate regression across all locations. **C:** The difference between the directly-calculated and predicted values for *beta*. Mean and uncertainty interval are shown across 1,000 posterior predictive draws over time. **D:** Histogram and box plot showing the distribution of root mean squared error (RMSE) for *beta* when aggregated across all dates from April 1 to present. **E:** Box plots showing the RMSE for aggregates of *beta* from a given date to the present across 1,000 posterior predictive draws.

40 Rhode Island: Detailed regression diagnostics

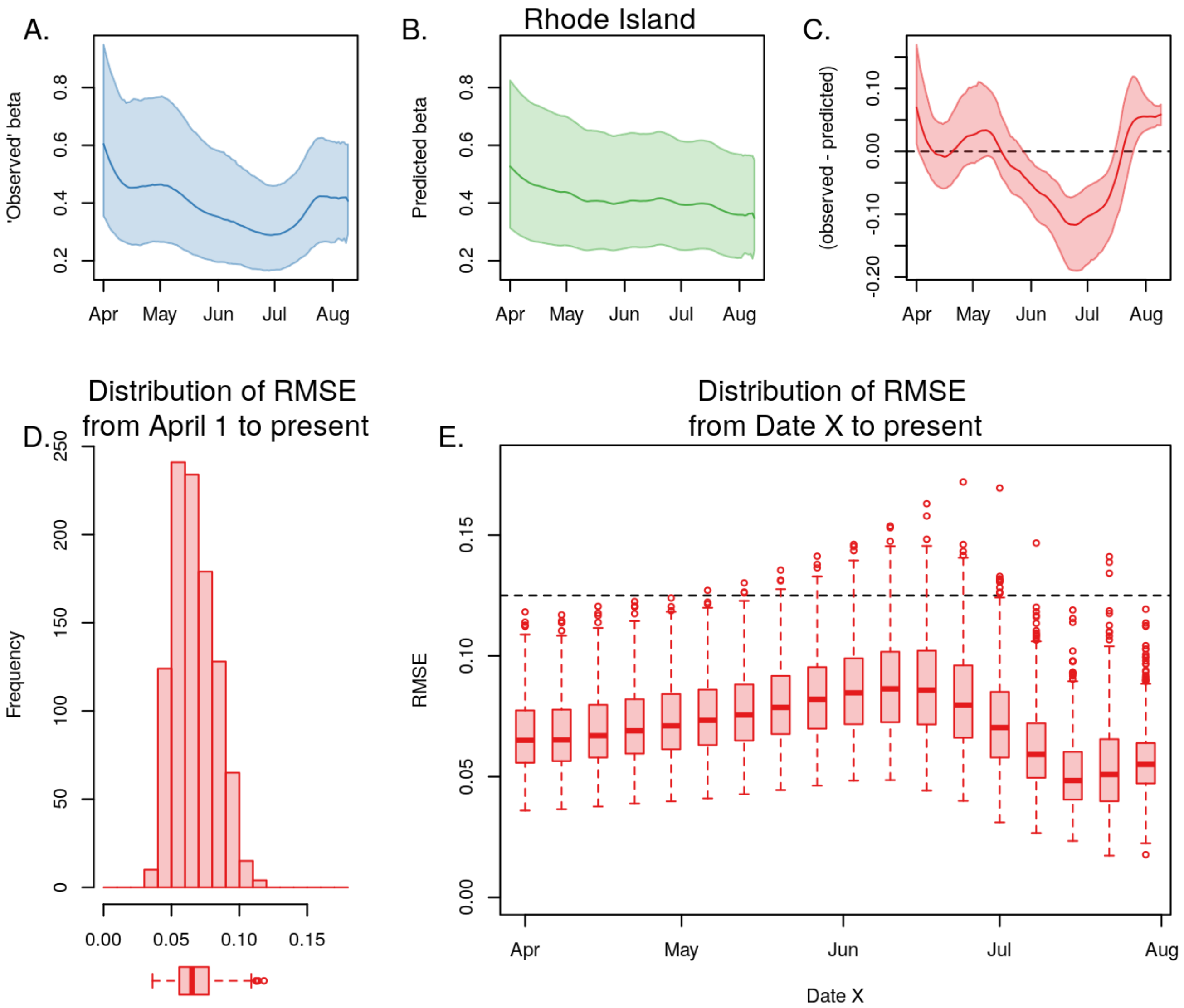

Rhode Island: Detailed regression diagnostics. A: The SEIR *beta* parameter calculated directly from past input data on infections. **B:** The *beta* parameter predicted using a multivariate regression across all locations. **C:** The difference between the directly-calculated and predicted values for *beta*. Mean and uncertainty interval are shown across 1,000 posterior predictive draws over time. **D:** Histogram and box plot showing the distribution of root mean squared error (RMSE) for *beta* when aggregated across all dates from April 1 to present. **E:** Box plots showing the RMSE for aggregates of *beta* from a given date to the present across 1,000 posterior predictive draws.

41 South Carolina: Detailed regression diagnostics

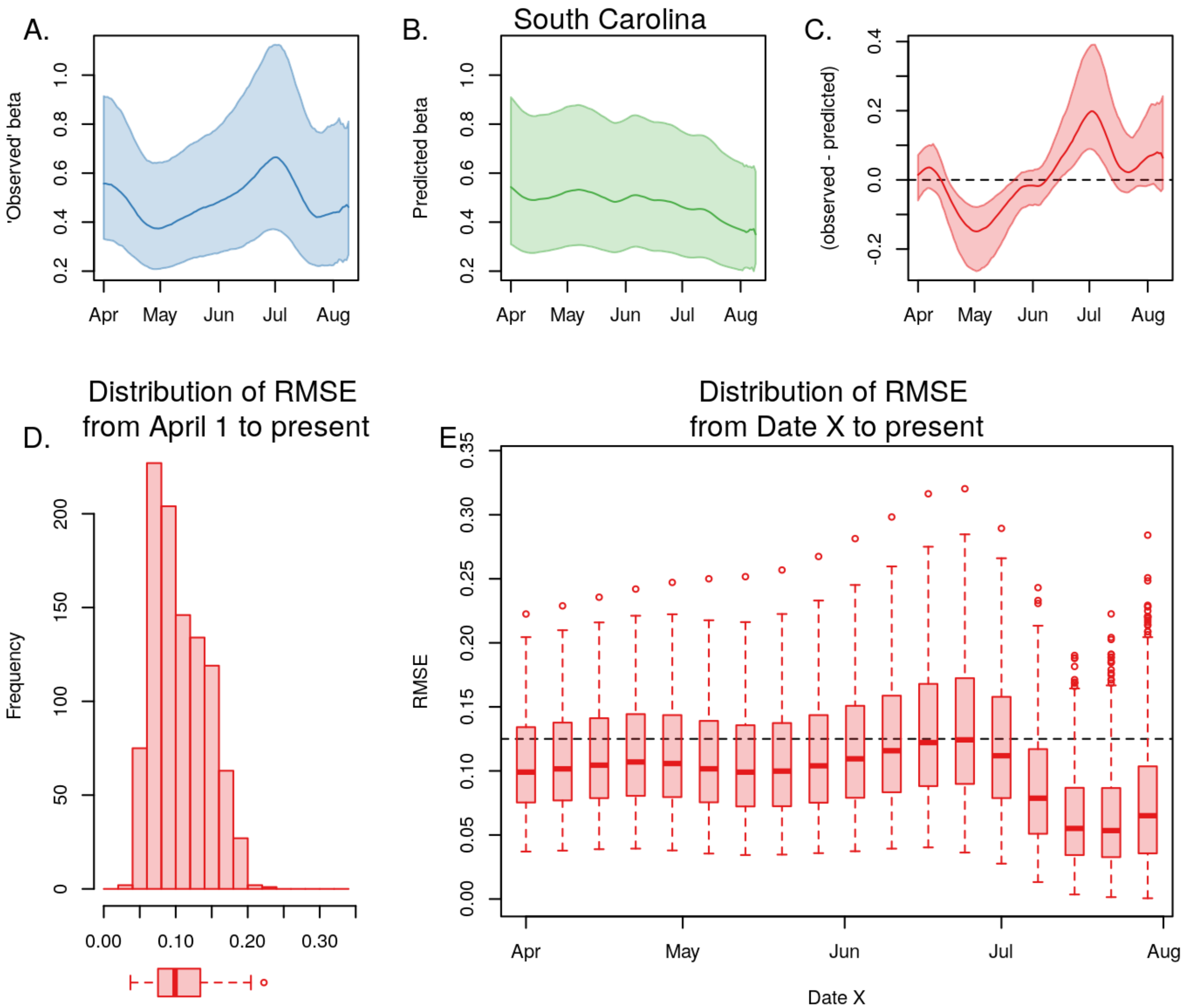

South Carolina: Detailed regression diagnostics. A: The SEIR *beta* parameter calculated directly from past input data on infections. **B:** The *beta* parameter predicted using a multivariate regression across all locations. **C:** The difference between the directly-calculated and predicted values for *beta*. Mean and uncertainty interval are shown across 1,000 posterior predictive draws over time. **D:** Histogram and box plot showing the distribution of root mean squared error (RMSE) for *beta* when aggregated across all dates from April 1 to present. **E:** Box plots showing the RMSE for aggregates of *beta* from a given date to the present across 1,000 posterior predictive draws.

42 South Dakota: Detailed regression diagnostics

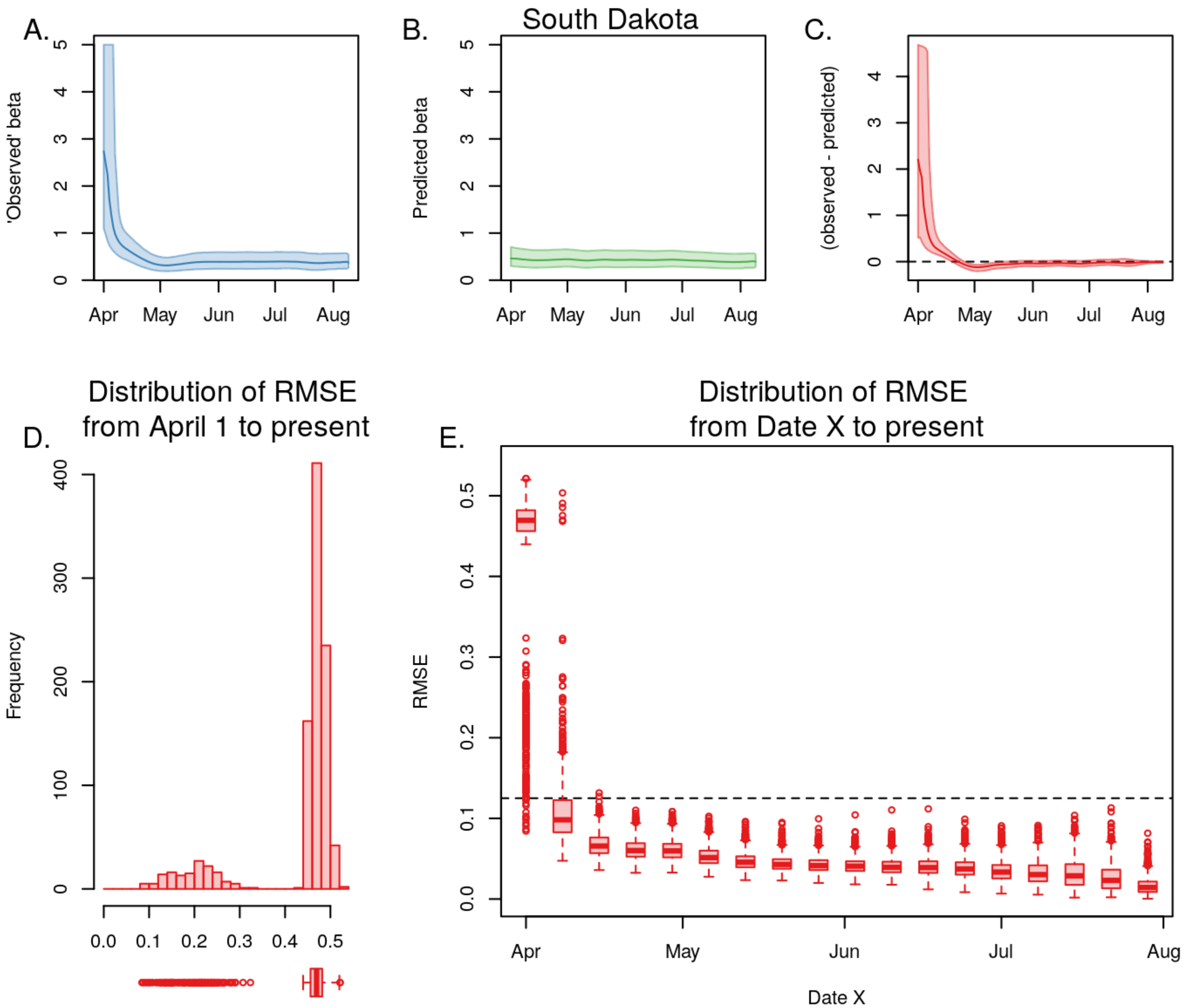

South Dakota: Detailed regression diagnostics. A: The SEIR *beta* parameter calculated directly from past input data on infections. **B:** The *beta* parameter predicted using a multivariate regression across all locations. **C:** The difference between the directly-calculated and predicted values for *beta*. Mean and uncertainty interval are shown across 1,000 posterior predictive draws over time. **D:** Histogram and box plot showing the distribution of root mean squared error (RMSE) for *beta* when aggregated across all dates from April 1 to present. **E:** Box plots showing the RMSE for aggregates of *beta* from a given date to the present across 1,000 posterior predictive draws.

43 Tennessee: Detailed regression diagnostics

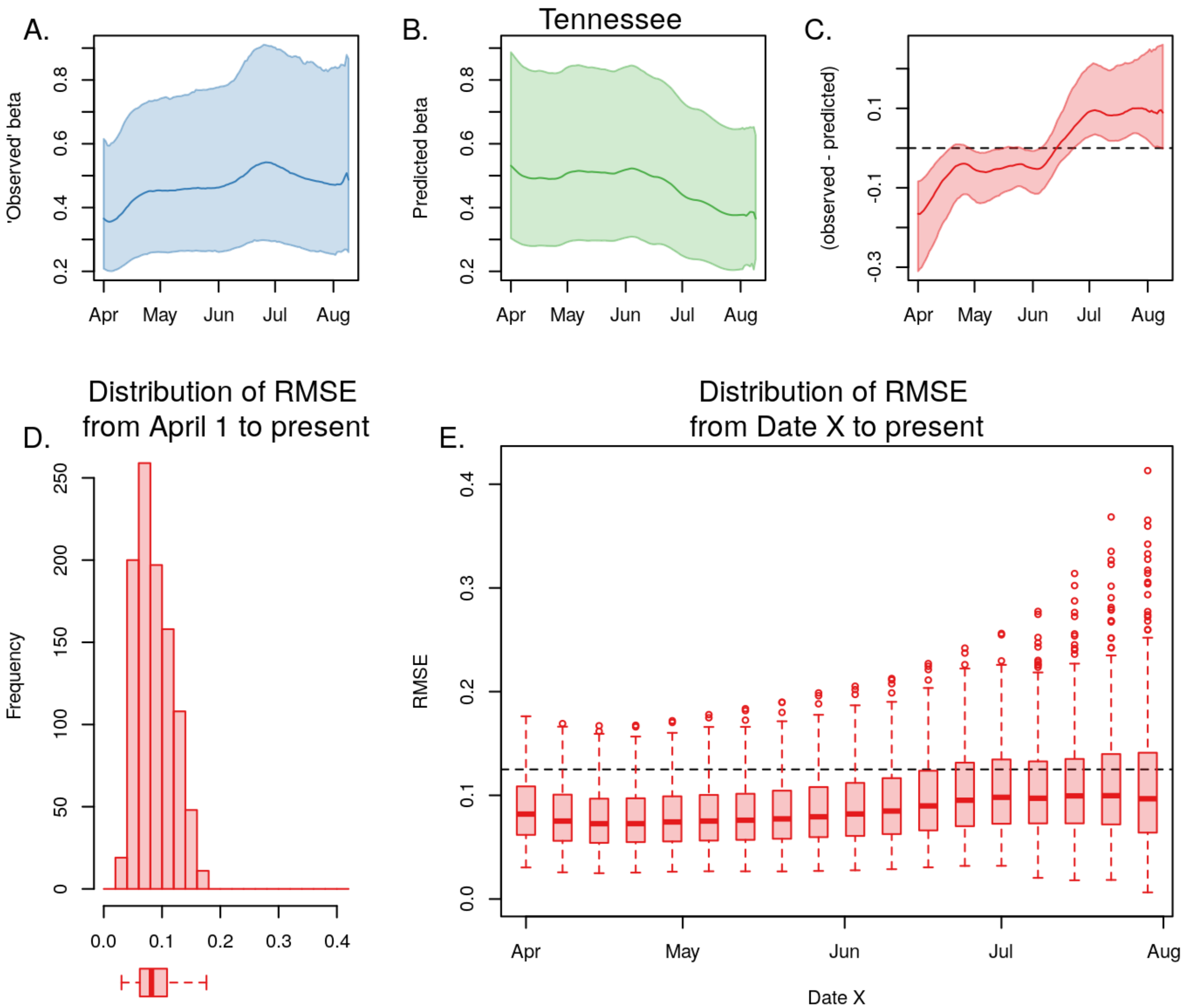

Tennessee: Detailed regression diagnostics. A: The SEIR *beta* parameter calculated directly from past input data on infections. **B:** The *beta* parameter predicted using a multivariate regression across all locations. **C:** The difference between the directly-calculated and predicted values for *beta*. Mean and uncertainty interval are shown across 1,000 posterior predictive draws over time. **D:** Histogram and box plot showing the distribution of root mean squared error (RMSE) for *beta* when aggregated across all dates from April 1 to present. **E:** Box plots showing the RMSE for aggregates of *beta* from a given date to the present across 1,000 posterior predictive draws.

44 Texas: Detailed regression diagnostics

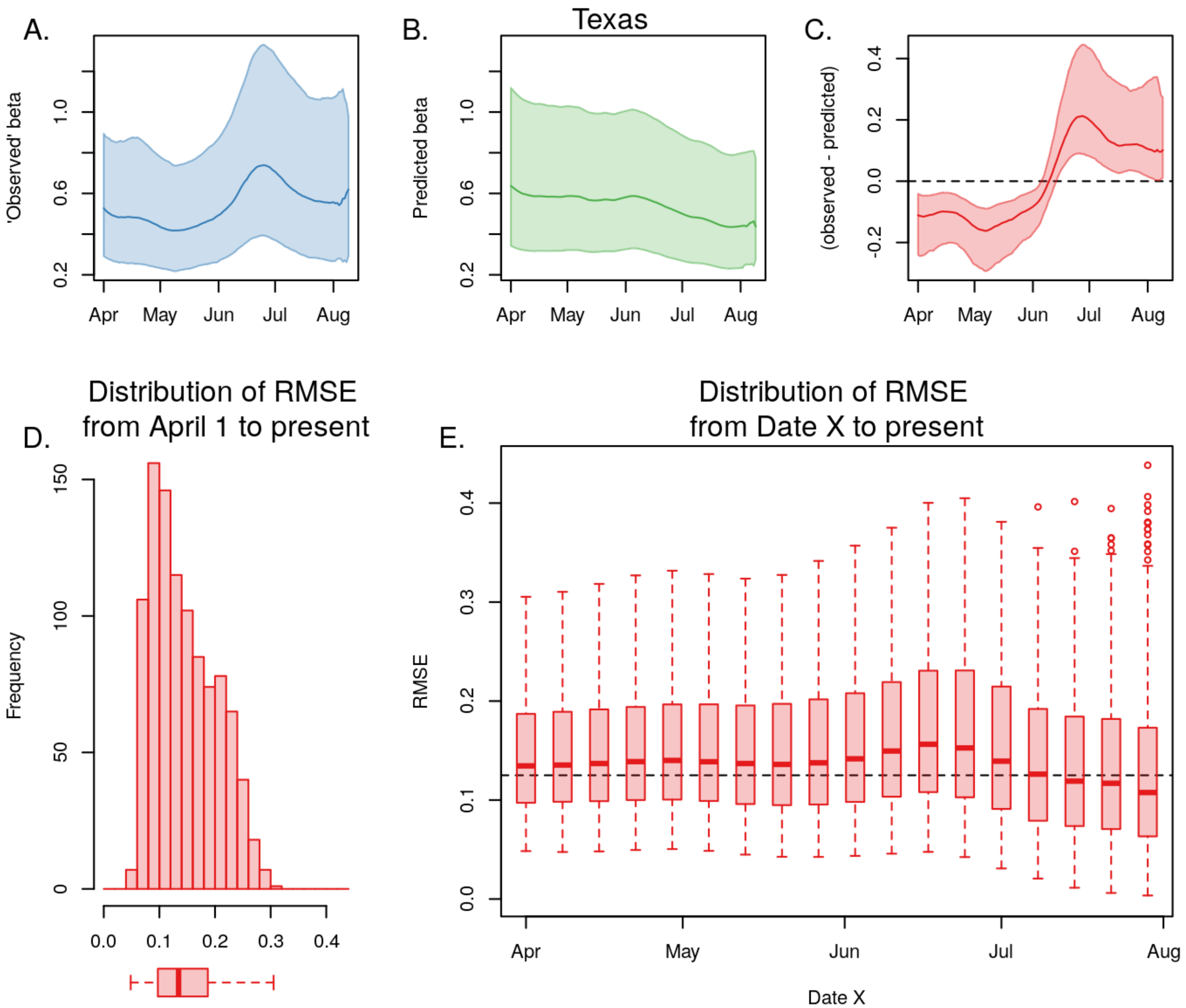

Texas: Detailed regression diagnostics. A: The SEIR *beta* parameter calculated directly from past input data on infections. **B:** The *beta* parameter predicted using a multivariate regression across all locations. **C:** The difference between the directly-calculated and predicted values for *beta*. Mean and uncertainty interval are shown across 1,000 posterior predictive draws over time. **D:** Histogram and box plot showing the distribution of root mean squared error (RMSE) for *beta* when aggregated across all dates from April 1 to present. **E:** Box plots showing the RMSE for aggregates of *beta* from a given date to the present across 1,000 posterior predictive draws.

45 Utah: Detailed regression diagnostics

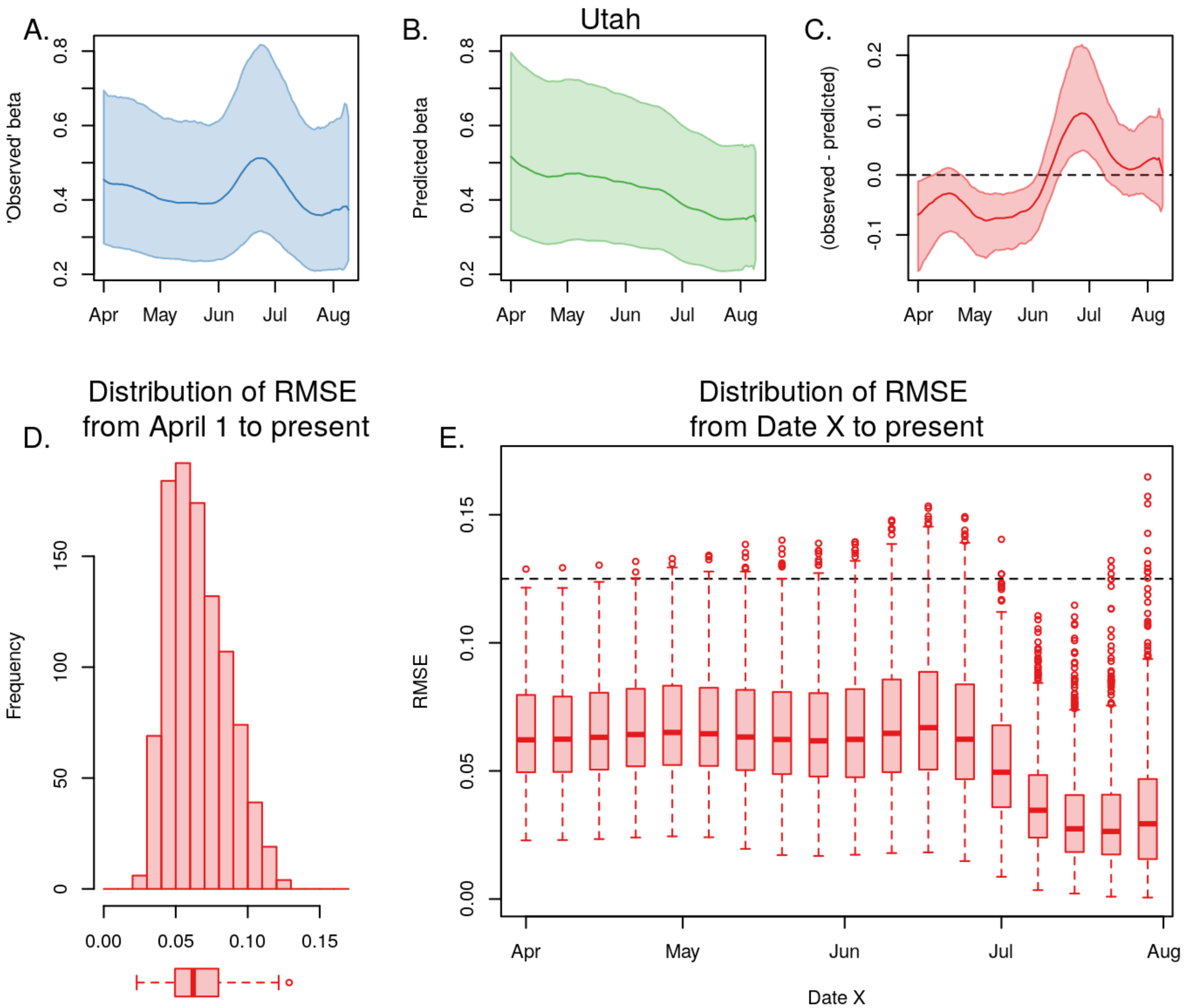

Utah: Detailed regression diagnostics. **A:** The SEIR *beta* parameter calculated directly from past input data on infections. **B:** The *beta* parameter predicted using a multivariate regression across all locations. **C:** The difference between the directly-calculated and predicted values for *beta*. Mean and uncertainty interval are shown across 1,000 posterior predictive draws over time. **D:** Histogram and box plot showing the distribution of root mean squared error (RMSE) for *beta* when aggregated across all dates from April 1 to present. **E:** Box plots showing the RMSE for aggregates of *beta* from a given date to the present across 1,000 posterior predictive draws.

46 Vermont: Detailed regression diagnostics

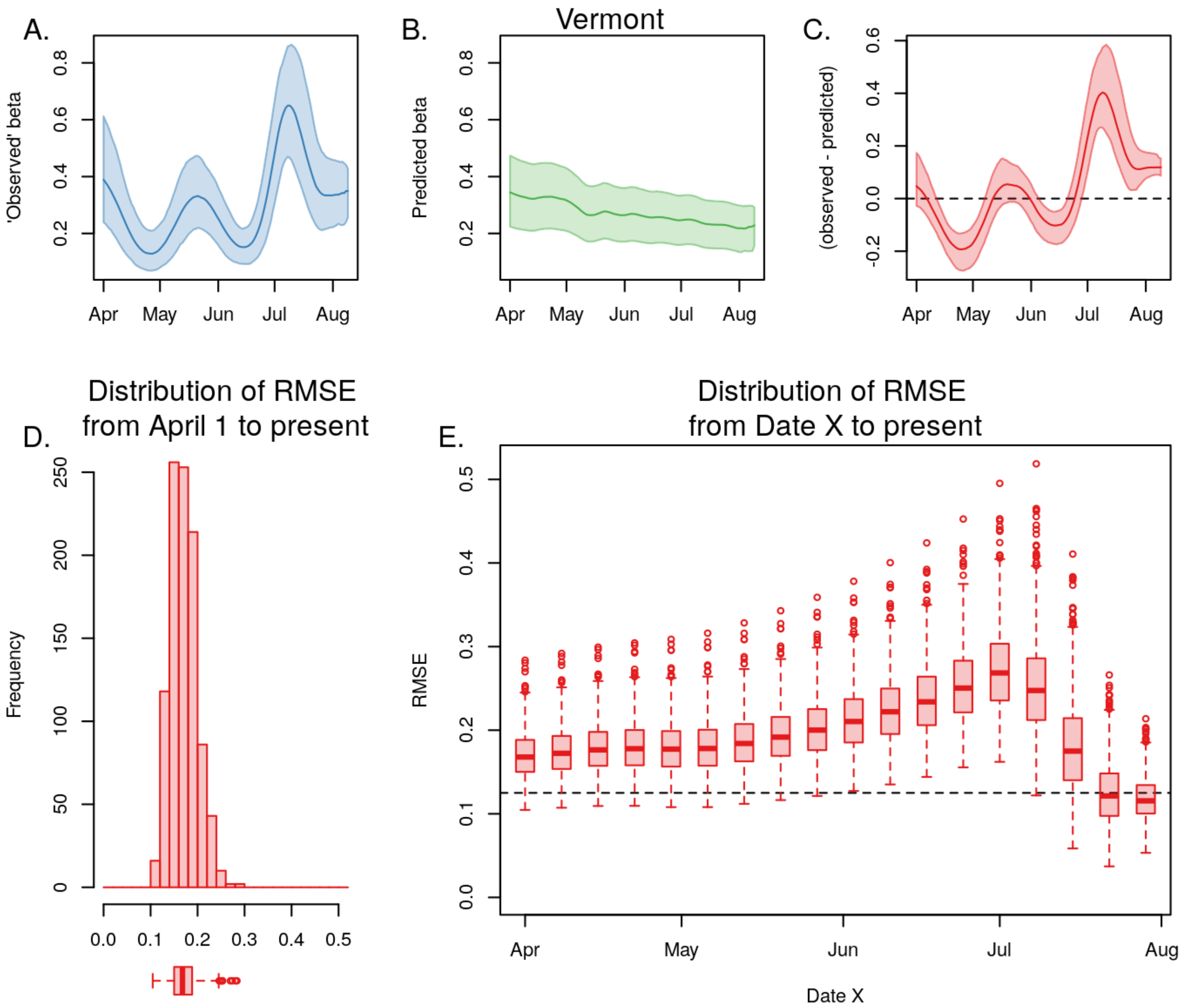

Vermont: Detailed regression diagnostics. A: The SEIR *beta* parameter calculated directly from past input data on infections. **B:** The *beta* parameter predicted using a multivariate regression across all locations. **C:** The difference between the directly-calculated and predicted values for *beta*. Mean and uncertainty interval are shown across 1,000 posterior predictive draws over time. **D:** Histogram and box plot showing the distribution of root mean squared error (RMSE) for *beta* when aggregated across all dates from April 1 to present. **E:** Box plots showing the RMSE for aggregates of *beta* from a given date to the present across 1,000 posterior predictive draws.

47 Virginia: Detailed regression diagnostics

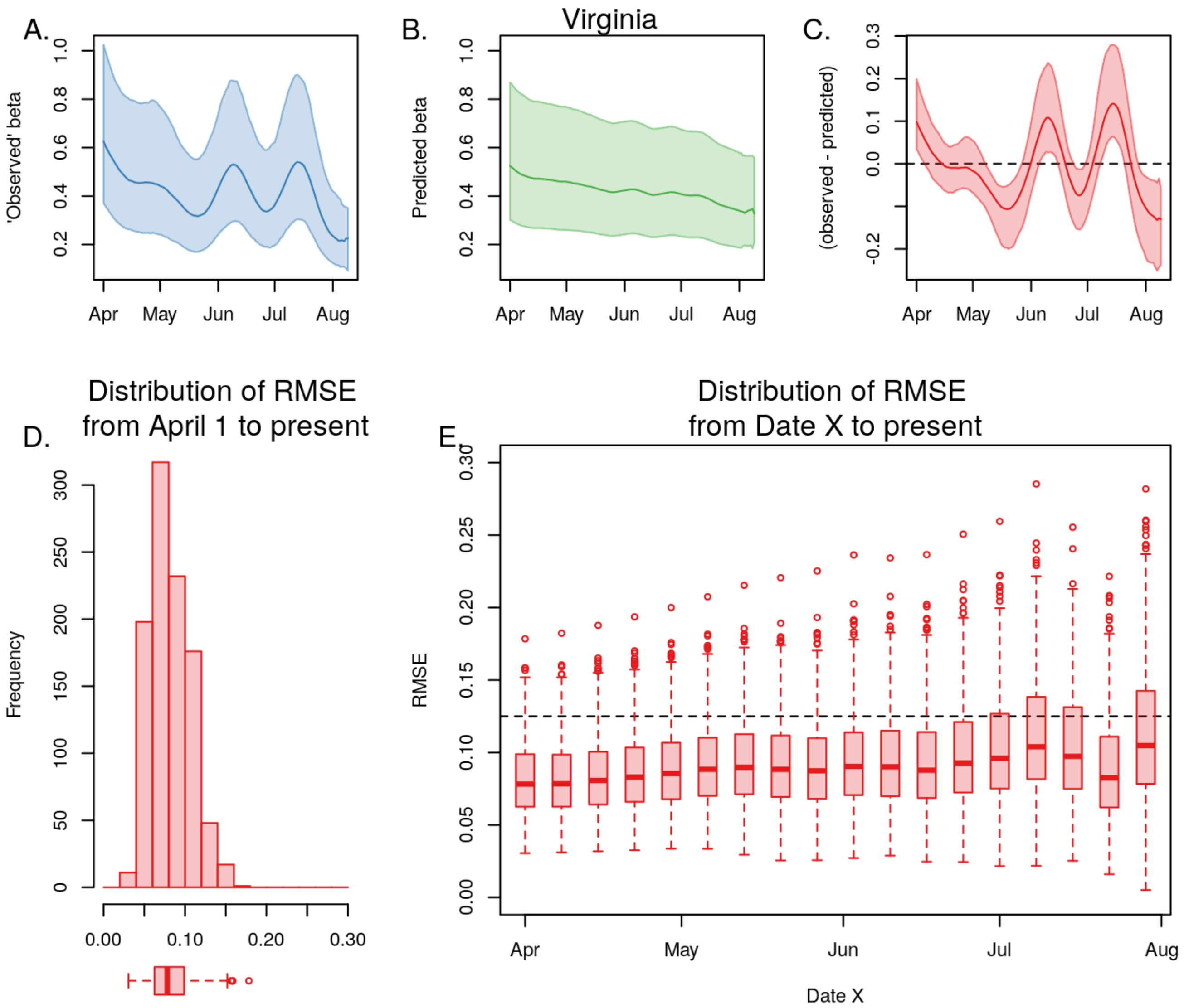

Virginia: Detailed regression diagnostics. **A:** The SEIR *beta* parameter calculated directly from past input data on infections. **B:** The *beta* parameter predicted using a multivariate regression across all locations. **C:** The difference between the directly-calculated and predicted values for *beta*. Mean and uncertainty interval are shown across 1,000 posterior predictive draws over time. **D:** Histogram and box plot showing the distribution of root mean squared error (RMSE) for *beta* when aggregated across all dates from April 1 to present. **E:** Box plots showing the RMSE for aggregates of *beta* from a given date to the present across 1,000 posterior predictive draws.

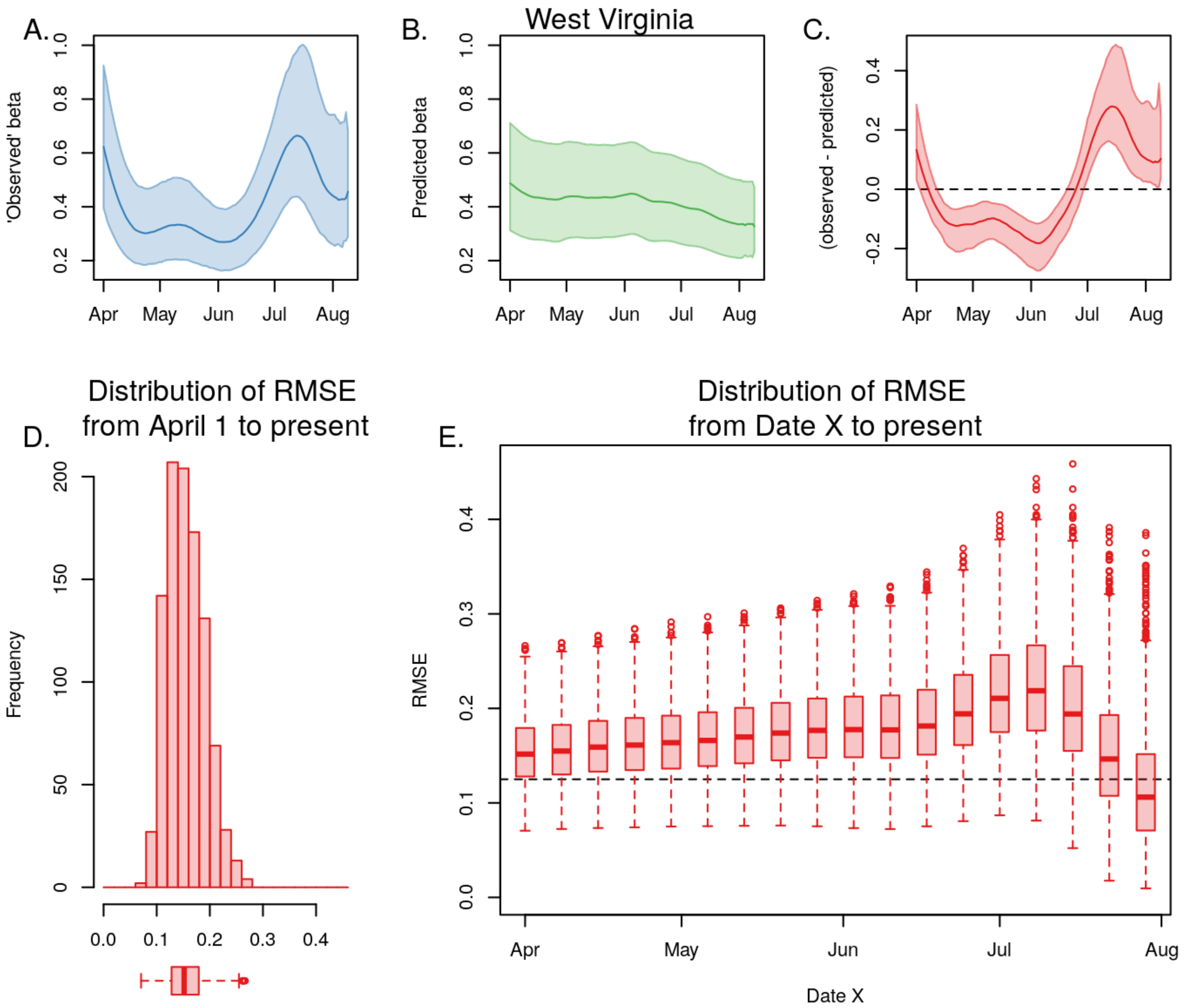

West Virginia: Detailed regression diagnostics. **A:** The SEIR *beta* parameter calculated directly from past input data on infections. **B:** The *beta* parameter predicted using a multivariate regression across all locations. **C:** The difference between the directly-calculated and predicted values for *beta*. Mean and uncertainty interval are shown across 1,000 posterior predictive draws over time. **D:** Histogram and box plot showing the distribution of root mean squared error (RMSE) for *beta* when aggregated across all dates from April 1 to present. **E:** Box plots showing the RMSE for aggregates of *beta* from a given date to the present across 1,000 posterior predictive draws.

49 Wisconsin: Detailed regression diagnostics

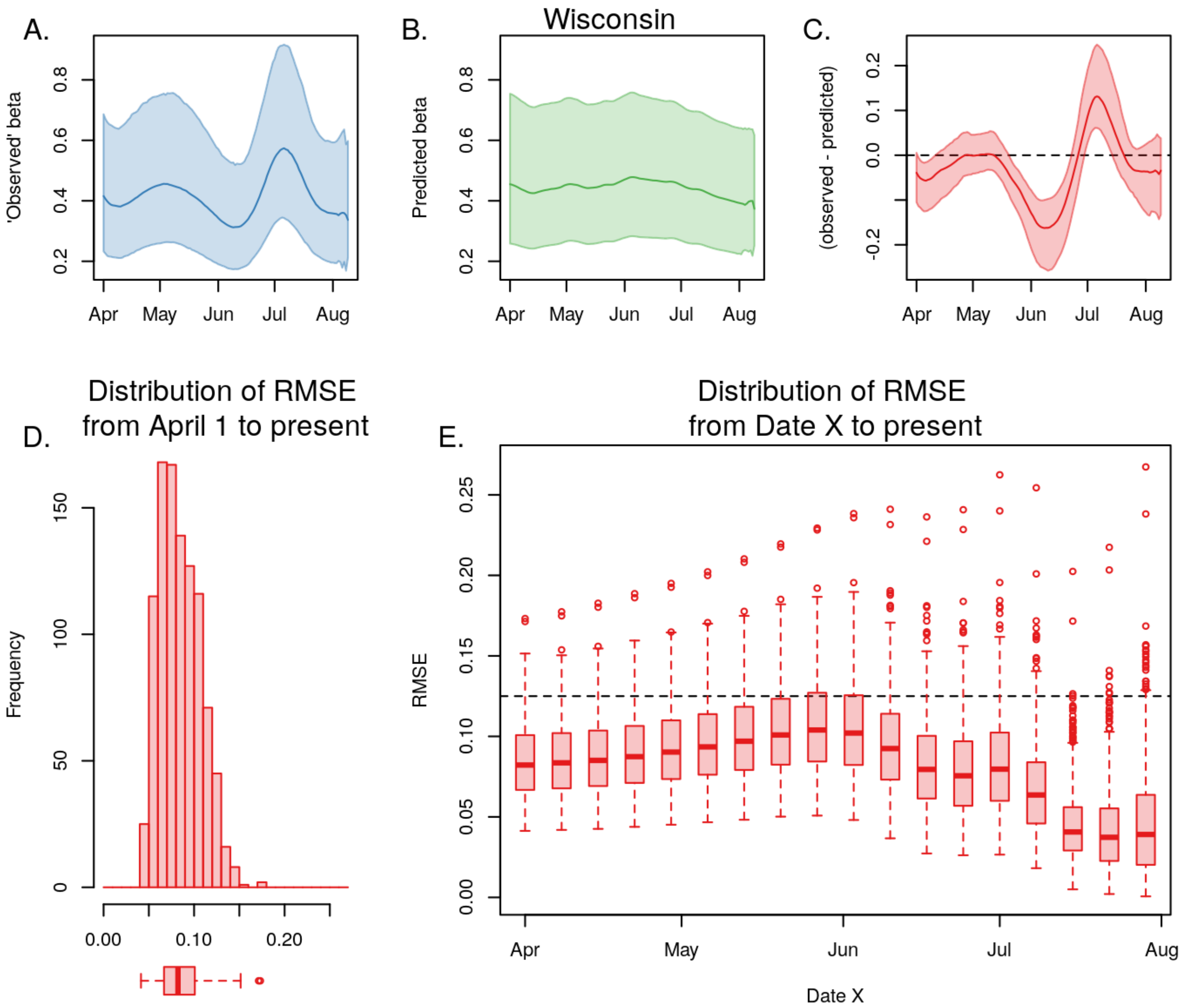

Wisconsin: Detailed regression diagnostics. A: The SEIR *beta* parameter calculated directly from past input data on infections. **B:** The *beta* parameter predicted using a multivariate regression across all locations. **C:** The difference between the directly-calculated and predicted values for *beta*. Mean and uncertainty interval are shown across 1,000 posterior predictive draws over time. **D:** Histogram and box plot showing the distribution of root mean squared error (RMSE) for *beta* when aggregated across all dates from April 1 to present. **E:** Box plots showing the RMSE for aggregates of *beta* from a given date to the present across 1,000 posterior predictive draws.

50 Wyoming: Detailed regression diagnostics

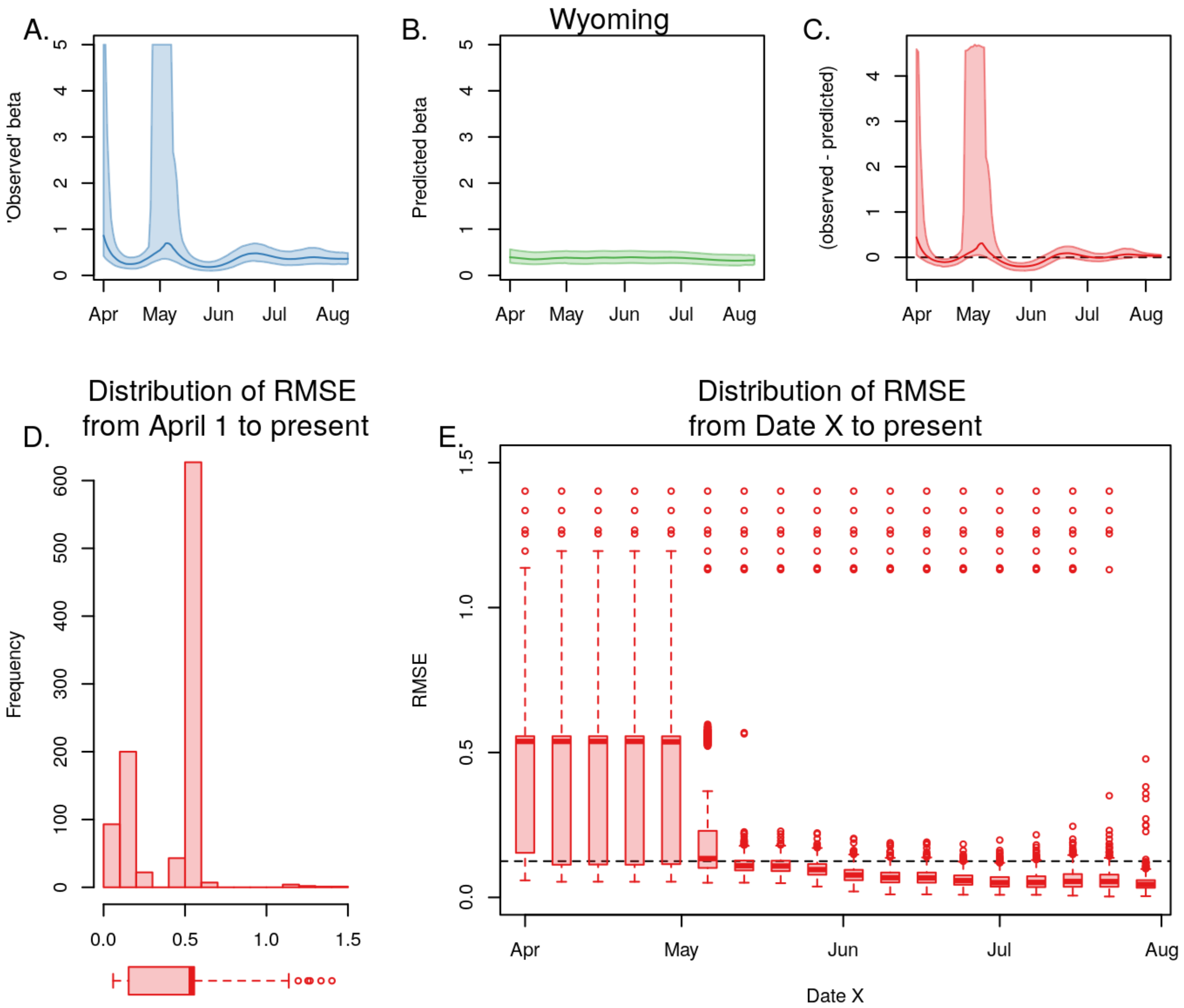

Wyoming: Detailed regression diagnostics. A: The SEIR *beta* parameter calculated directly from past input data on infections. **B:** The *beta* parameter predicted using a multivariate regression across all locations. **C:** The difference between the directly-calculated and predicted values for *beta*. Mean and uncertainty interval are shown across 1,000 posterior predictive draws over time. **D:** Histogram and box plot showing the distribution of root mean squared error (RMSE) for *beta* when aggregated across all dates from April 1 to present. **E:** Box plots showing the RMSE for aggregates of *beta* from a given date to the present across 1,000 posterior predictive draws.

51 Spokane County: Detailed regression diagnostics

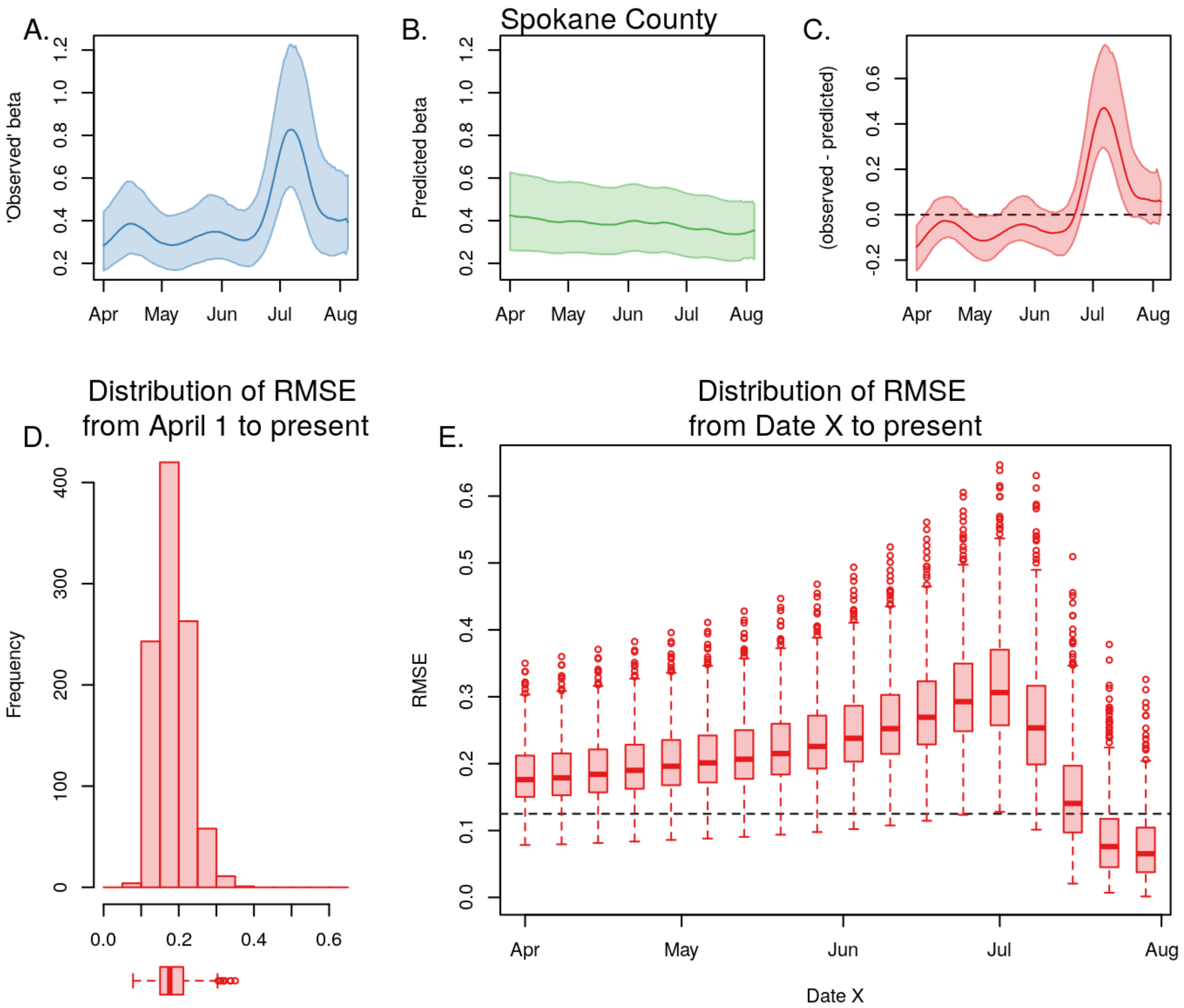

Spokane County: Detailed regression diagnostics. **A:** The SEIR *beta* parameter calculated directly from past input data on infections. **B:** The *beta* parameter predicted using a multivariate regression across all locations. **C:** The difference between the directly-calculated and predicted values for *beta*. Mean and uncertainty interval are shown across 1,000 posterior predictive draws over time. **D:** Histogram and box plot showing the distribution of root mean squared error (RMSE) for *beta* when aggregated across all dates from April 1 to present. **E:** Box plots showing the RMSE for aggregates of *beta* from a given date to the present across 1,000 posterior predictive draws.

52 King and Snohomish Counties: Detailed regression diagnostics

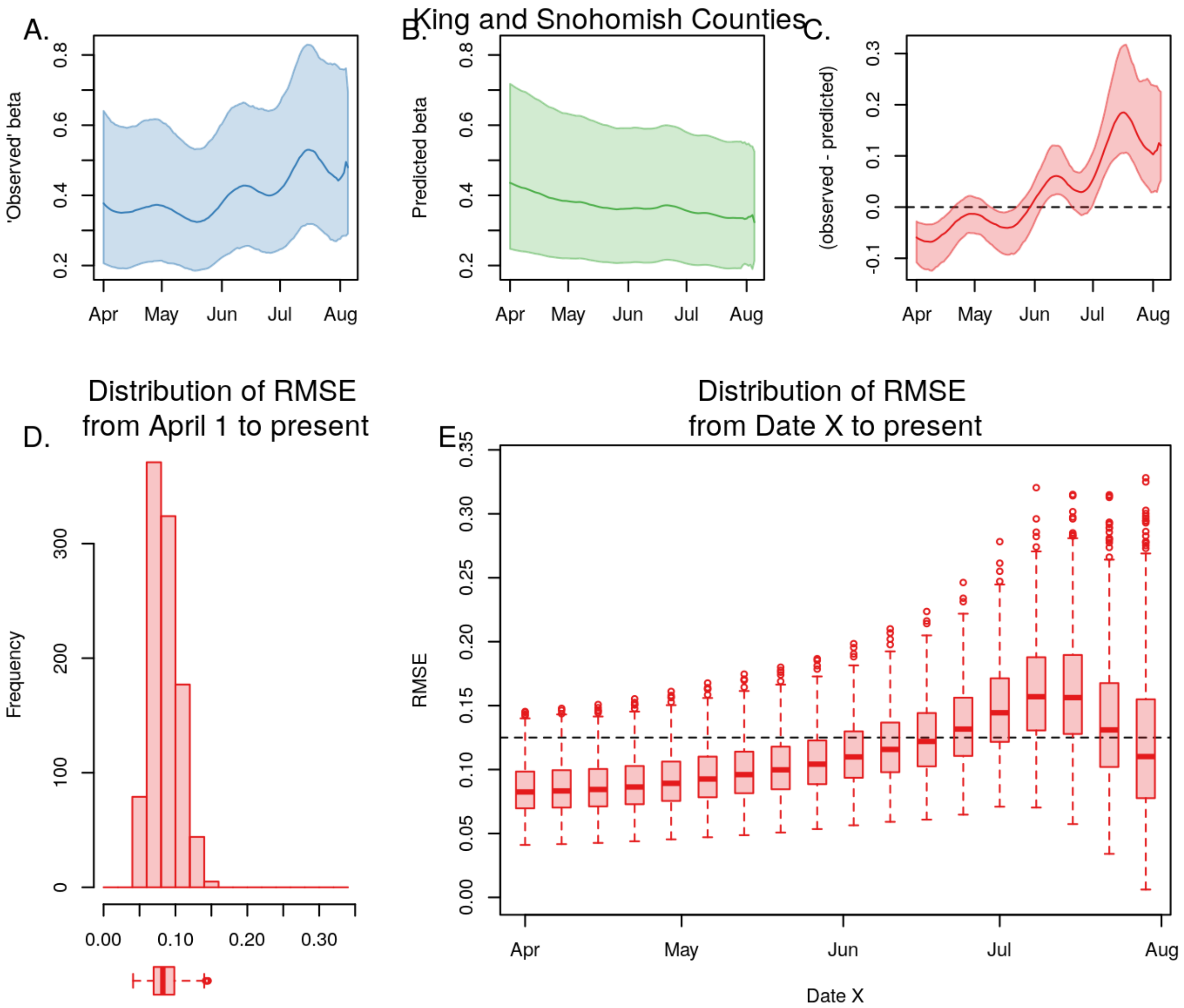

King and Snohomish Counties: Detailed regression diagnostics. A: The SEIR *beta* parameter calculated directly from past input data on infections. **B:** The *beta* parameter predicted using a multivariate regression across all locations. **C:** The difference between the directly-calculated and predicted values for *beta*. Mean and uncertainty interval are shown across 1,000 posterior predictive draws over time. **D:** Histogram and box plot showing the distribution of root mean squared error (RMSE) for *beta* when aggregated across all dates from April 1 to present. **E:** Box plots showing the RMSE for aggregates of *beta* from a given date to the present across 1,000 posterior predictive draws.

53 Washington except for King, Snohomish, and Spokane Counties: Detailed regression diagnostics

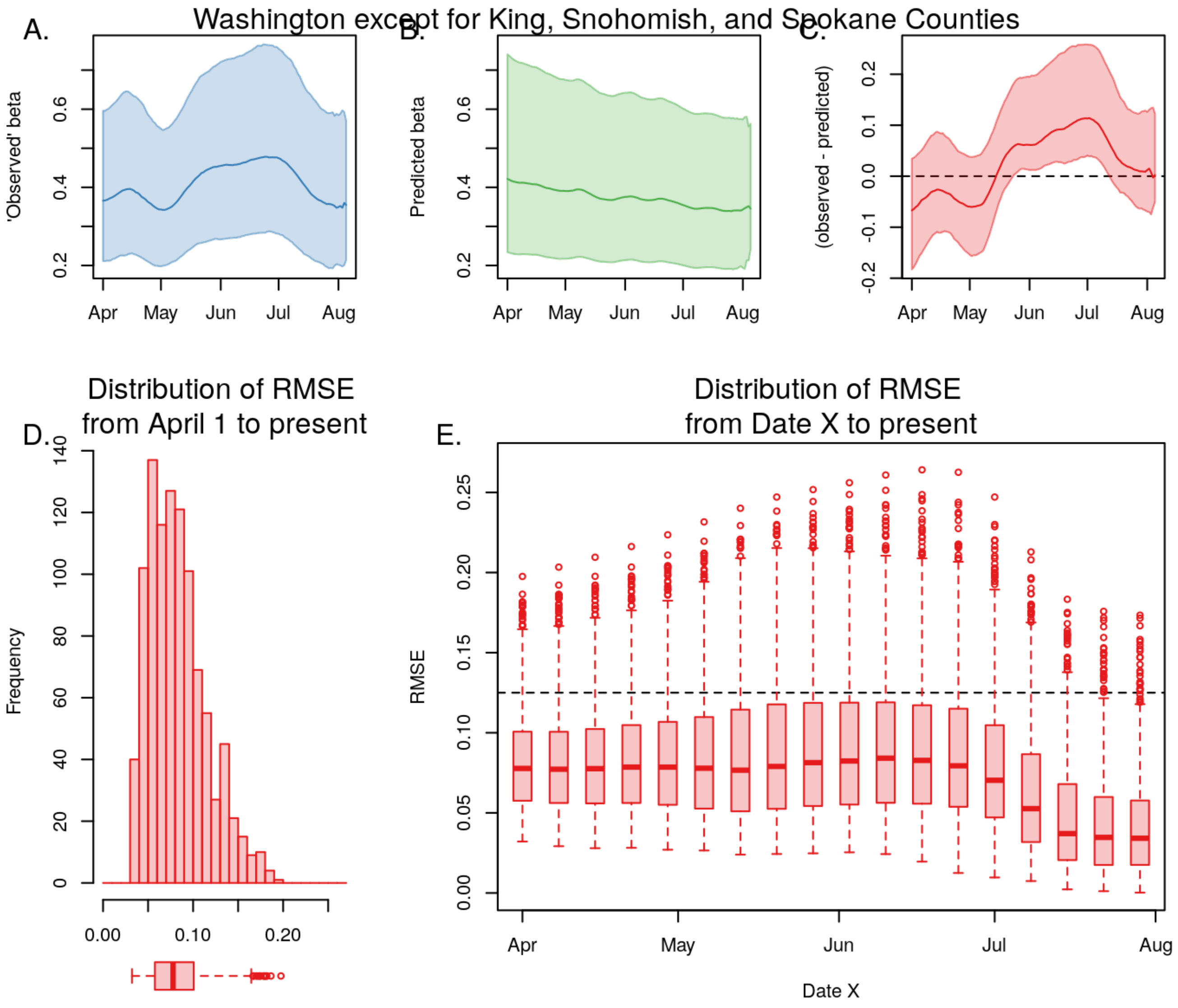

Washington except for King, Snohomish, and Spokane Counties: Detailed regression diagnostics. A: The SEIR *beta* parameter calculated directly from past input data on infections. **B:** The *beta* parameter predicted using a multivariate regression across all locations. **C:** The difference between the directly-calculated and predicted values for *beta*. Mean and uncertainty interval are shown across 1,000 posterior predictive draws over time. **D:** Histogram and box plot showing the distribution of root mean squared error (RMSE) for *beta* when aggregated across all dates from April 1 to present. **E:** Box plots showing the RMSE for aggregates of *beta* from a given date to the present across 1,000 posterior predictive draws.
