# Supplementary material for: Modeling COVID-19 scenarios for the United States
Source: Nat Med. 2020 Oct 23;27(1):94–105. doi: 10.1038/s41591-020-1132-9 (PMC7806509; doi:10.1038/s41591-020-1132-9)
Supplement: Supplementary file 7 — Appendix 5: spatial distribution of selected covariates. [file 41591_2020_1132_MOESM7_ESM.pdf]

Appendix 5:
Spatial Distribution of Select Covariates

Contents

1 PM 2.5 Air Pollution **3**

2 Lower Respiratory Infection Mortality **4**

3 Pneumonia Seasonality **5**

4 Smoking Prevalence **6**

5 Temperature **7**

1 PM 2.5 Air Pollution

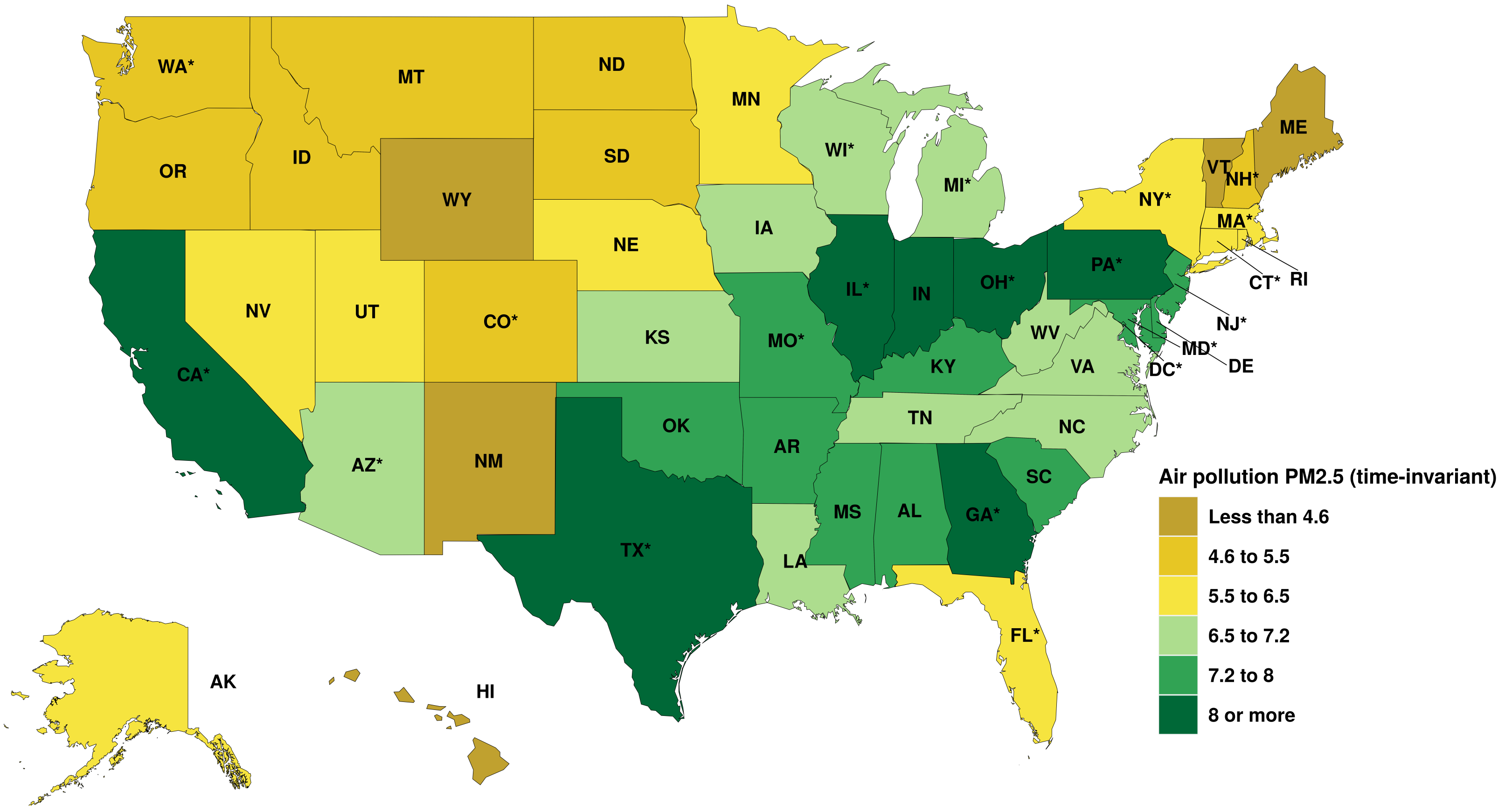

Map of PM 2.5 Air Pollution This map shows the estimated concentration of PM 2.5 air pollution (units: micograms per cubic meter) by US state. For more information, see Section 3 of the Supplementary Appendix.

2 Lower Respiratory Infection Mortality

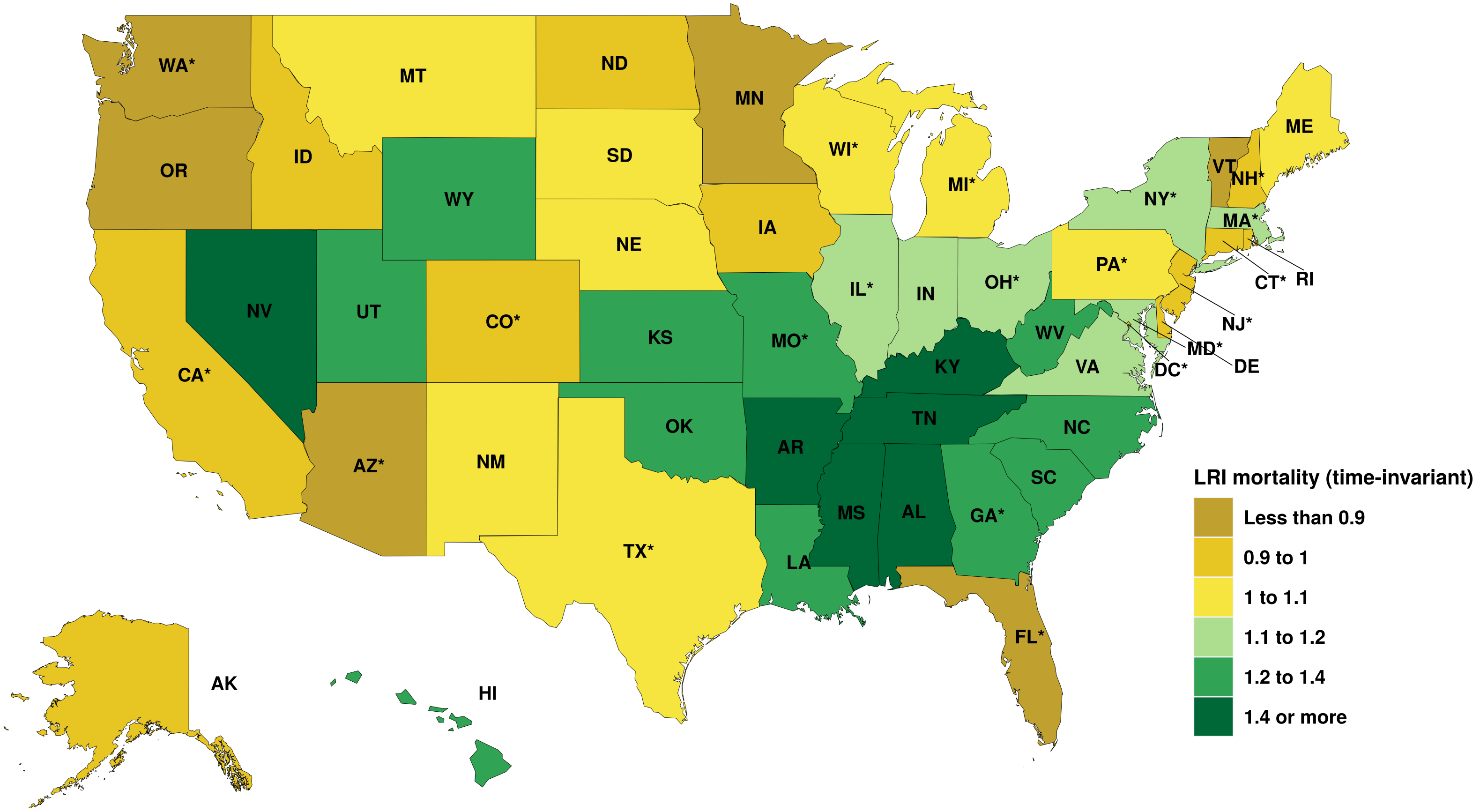

Map of Lower Respiratory Infection Mortality This map shows estimates of the age-standardized mortality rate (per 100,000 population) from lower respiratory infections in age groups 15 and over by US state. For more information, see Section 3 of the Supplementary Appendix.

3 Pneumonia Seasonality

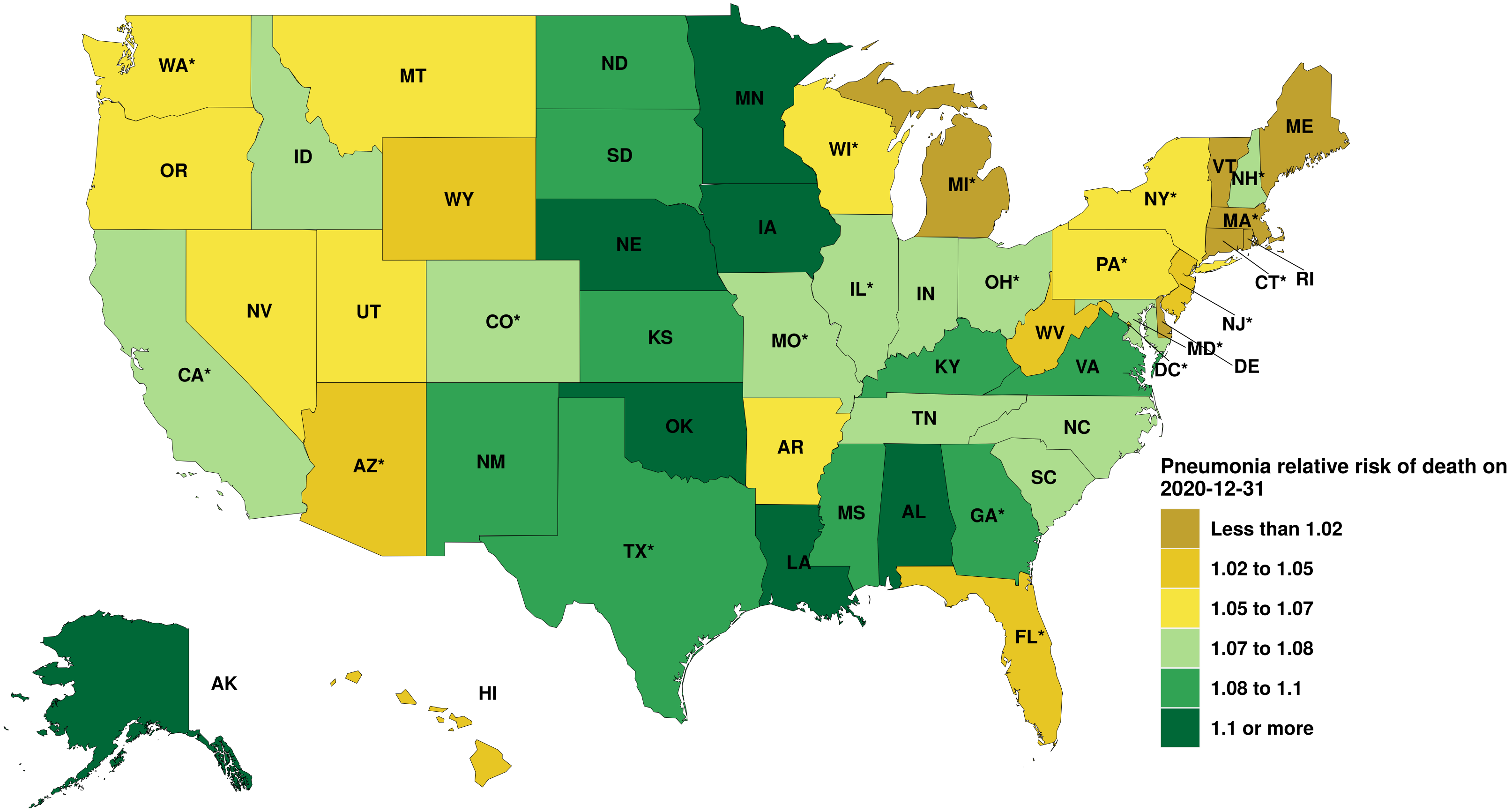

Map of Pneumonia Seasonality (Snapshot) This map shows a snapshot of estimated pneumonia seasonality by US state on December 31, 2020. This metric is calculated as the risk of death from pneumonia on a particular week of the year, divided by the average risk of death from pneumonia across all weeks. For more information, see Section 3 of the Supplementary Appendix.

4 Smoking Prevalence

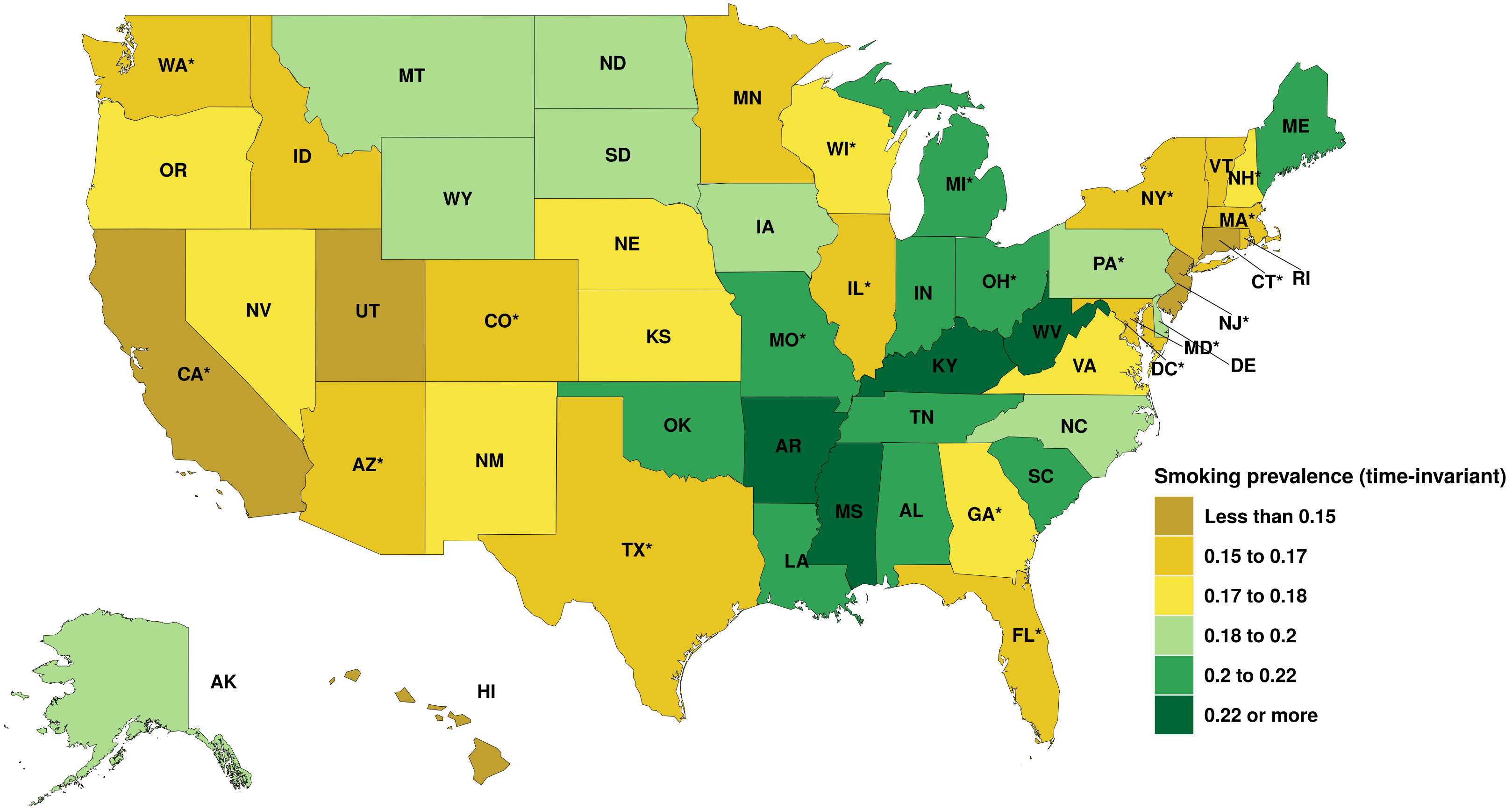

Map of Smoking Prevalence This map shows estimates of age-standardized prevalence of tobacco smoking among the population aged 15 and above by US state in 2019. For more information, see Section 3 of the Supplementary Appendix.

5 Temperature

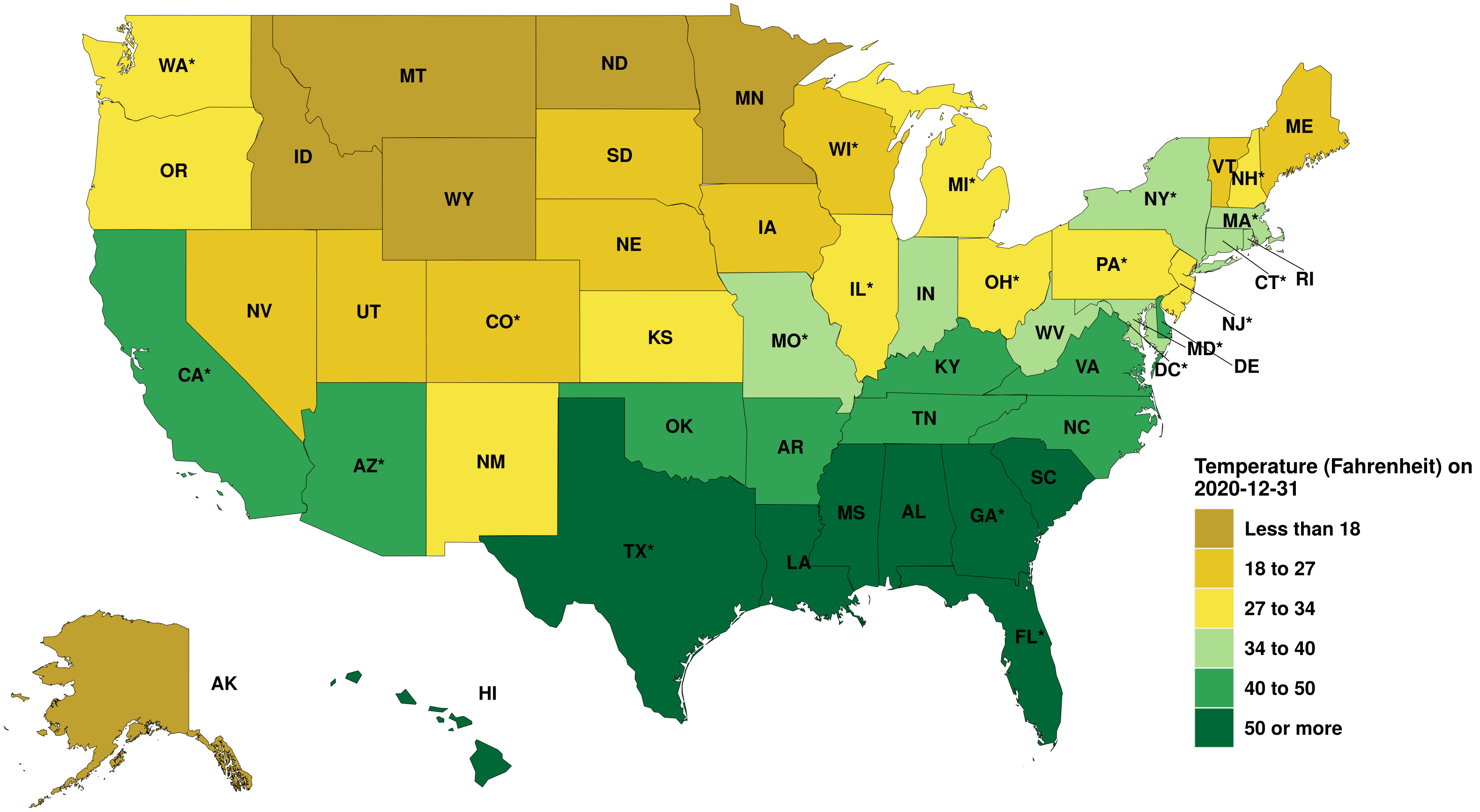

Map of Temperature (Snapshot) This map shows an estimate of the average temperature (in Fahrenheit) by state on December 31, 2020. For more information, see Section 3 of the Supplementary Appendix.
